# SARS-CoV-2-induced IgG repertoires shape T cell responses, microRNA regulation, and autoreactivity according to disease severity

**DOI:** 10.1016/j.jtauto.2026.100392

**Published:** 2026-08-03

**Authors:** Fabio da Ressureição Sgnotto, Nicolle Rakanidis Machado, Lais Alves do Nascimento, Beatriz Oliveira Fagundes, Isabella Siuffi Bergamasco, Sabri Saeed Sanabani, Maria Notomi Sato, Jefferson Russo Victor

**Affiliations:** a Post Graduation Program in Health Sciences, Santo Amaro University (UNISA), São Paulo, Brazil; b Laboratory of Medical Investigation LIM-56, Division of Dermatology, Medical School, University of São Paulo, São Paulo, Brazil; c Department of Dermatology, University Medical Center Groningen, Groningen, the Netherlands; d Laboratory of Medical Investigation LIM-03, Clinics Hospital, Medical School, University of São Paulo, São Paulo, Brazil; e Medical School, Santo Amaro University (UNISA), Sao Paulo, Brazil

**Keywords:** SARS-CoV-2, COVID-19, IgG repertoire, CD4^+^ T cells, CD8^+^ T cells, Cytokines, microRNAs, Autoreactivity, Immune modulation, Disease severity

## Abstract

SARS-CoV-2 infection can generate IgG repertoires with functions beyond viral neutralization. We purified IgG from non-exposed healthy controls, moderate and severe COVID-19 patients, and IVIg, and assessed their effects on healthy-donor PBMCs. COVID-19 IgG bound CD4^+^ and CD8^+^ T cells without inducing apoptosis. Severe-COVID IgG reduced Treg frequencies, enhanced IFN-γ production, and expanded autoreactivity toward proteins linked to proteostasis, whereas moderate-COVID IgG promoted CD8^+^ IL-22 and selective miRNA downregulation. These findings identify disease-severity-associated antibody-mediated immune modulation *in vitro* and motivate patient-resolved longitudinal validation.

## 1 Main text

The immune response to SARS-CoV-2 is usually interpreted through the coordinated activity of antiviral T cells and antibodies. CD4^+^ and CD8^+^ T cells participate in viral recognition, effector differentiation, and memory formation during COVID-19 [1,2]. Broad SARS-CoV-2-specific T-cell responses have also been detected in convalescent individuals and are considered relevant to protection and immune durability [[3], [4], [5]]. In parallel, IgG antibodies are commonly evaluated as markers of exposure, neutralizing activity, or Fc-dependent antiviral function. However, a growing body of work indicates that polyclonal IgG repertoires can also behave as direct immunomodulators, particularly when they contain idiotypes capable of interacting with immune-cell targets. This principle has been observed in allergic and atopic settings [6,7], in studies of thymic and peripheral lymphocyte modulation [[8], [9], [10], [11], [12], [13]], and in dermatological inflammation [[14], [15], [16], [17]]. Related observations in viral infections further support the concept that disease-associated IgG can influence cytokine profiles independently of the original pathogen [18,19].

This possibility is particularly relevant to COVID-19 because the disease has been repeatedly associated with autoantibody generation and post-acute immune dysregulation. Reviews of convalescent cohorts have emphasized that autoantibodies may persist after infection and may contribute to symptoms in a subset of patients [20,21]. Proteome-wide studies have also shown that SARS-CoV-2-induced IgG idiotypes can recognize numerous human proteins, suggesting that infection may reshape the antibody repertoire beyond classical antiviral specificity [22]. A subsequent mapping study indicated that these IgG repertoires may target proteins expressed across peripheral lymphoid and myeloid cell types, but direct membrane interaction and functional consequences for targeted cells remained insufficiently defined [23]. The present Short Communication therefore reports completed experimental work testing whether IgG repertoires from moderate and severe COVID-19 patients directly interact with human CD4^+^ and CD8^+^ T cells, modulate their functional profile, and differ in autoreactive breadth according to disease severity.

Serum samples were obtained from 79 R T-PCR-confirmed COVID-19 patients recruited between May and July 2020. Patients with hospitalized pneumonia who did not require high-flow oxygen or non-invasive ventilation were classified as moderate COVID-19 (COVID-Mod; n = 39), whereas patients requiring high-flow oxygen therapy or non-invasive ventilation were classified as severe COVID-19 (COVID-Sev; n = 40). Pre-pandemic sera from 40 healthy individuals collected in 2019 were used as non-exposed healthy controls. IgG was purified from pooled sera from each group and compared with commercial IVIg. The use of pooled IgG was intentional: the goal was not to identify patient-specific biomarkers, but to test whether disease-severity-associated antibody repertoires could impose reproducible functional effects under standardized culture conditions. Healthy-donor PBMCs were exposed to purified IgG at 100 μg/mL for three days, and CD4^+^ and CD8^+^ T-cell phenotypes and intracellular cytokines were evaluated by flow cytometry. Short incubations with Zenon-labeled IgG were used to test direct membrane binding and Annexin V staining. SARS-CoV-2 epitope recognition was assessed using an infectious-disease epitope microarray, small-RNA profiles were generated from IgG-exposed PBMCs, and candidate autoantibody targets were mapped using HuProt human proteome microarrays focused on proteins annotated as expressed in CD4^+^ or CD8^+^ T cells (Supplementary Table 1). Flow-cytometry procedures followed previously established protocols from our group [13,[24], [25], [26]].

Replicate structure and variability were defined according to the relevant experimental unit. Each study-group condition was represented by a single pooled IgG preparation comprising sera from 40 non-exposed healthy controls, 39 patients with moderate COVID-19 (COVID-Mod), or 40 patients with severe COVID-19 (COVID-Sev), whereas IVIg was represented by one commercial preparation. Thus, the IgG preparations represented group-level antibody-repertoire conditions rather than patient-level biological replicates. Flow-cytometric phenotyping, cytokine-production, and membrane-binding assays were performed using PBMCs from 12 independent healthy donors across six experiments. All IgG conditions were tested within each donor, allowing matched comparisons, and individual PBMC donors constituted the biological replicates for these cellular assays. For small-RNA profiling, equal amounts of RNA from 20 independent healthy PBMC donors, evaluated across ten experiments, were combined to generate one RNA pool for each exposure condition. Each condition-specific RNA pool was evaluated in at least three sequencing replicates, which represented technical rather than donor-level biological replication. The infectious-disease epitope and HuProt microarrays were performed using the same group-specific pooled IgG preparations employed in the cellular assays. Where technical replicates were available, they were averaged before statistical analysis. Because serum pooling collapses patient-level variability, clinical correlations and estimates of inter-individual heterogeneity among patients could not be performed.

A further consideration is that the present experimental design separates antibody-mediated effects from ongoing infection. Patient sera were collected during acute COVID-19, but the functional assays used PBMCs from healthy donors tested under identical culture conditions. This approach reduces confounding by patient-specific inflammation, lymphopenia, corticosteroid exposure, or tissue damage, and focuses the analysis on the information carried by purified IgG. For Journal of Translational Autoimmunity readers, this distinction is important because it places the work within post-infectious immune regulation rather than within conventional viral immunology alone. The experimental question was not whether SARS-CoV-2-specific T cells are altered during infection, but whether IgG repertoires elicited during infection can subsequently influence human lymphocytes and recognize self-proteins. This design therefore complements studies of acute COVID-19 immunophenotyping and tests a potential mechanism of humoral immune modulation whose persistence and clinical relevance require longitudinal validation.

COVID-Mod and COVID-Sev IgG clearly recognized SARS-CoV-2 epitopes, whereas IVIg and non-exposed healthy-control IgG showed no detectable reactivity (Supplementary Fig. 2). This confirmed that the patient-derived preparations retained disease-related antiviral antibody features. In PBMC cultures, IgG exposure did not broadly remodel the distribution of central memory, effector memory, tissue-resident memory, or TemRA subsets among CD4^+^ or CD8^+^ T cells; gating strategy shown in Supplementary Fig. 1. Nevertheless, severe-COVID IgG selectively reduced the frequency of CD4^+^CD25^+^CD127-regulatory T cells (Tregs), indicating a focused effect on a population central to immune tolerance and control of excessive inflammation. This observation is consistent with the known relevance of Tregs in human immune-mediated disease [27], but it also resonates with reports describing profound Treg perturbations in COVID-19 [28]. Other studies have suggested that SARS-CoV-2 infection alters the regulatory/cytotoxic balance in CD4^+^ responses [29], whereas single-clonotype analyses indicate that expanded Tregs are not necessarily driven by dominant SARS-CoV-2 antigen specificity [30]. These findings support the possibility that non-viral, repertoire-level antibody interactions participate in the regulatory T-cell landscape associated with COVID-19.

**Fig. 1:**
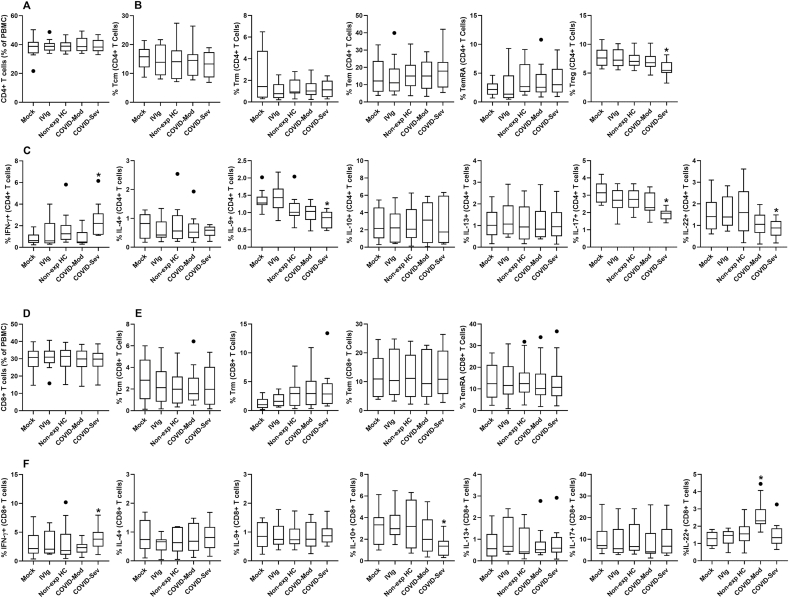
**Frequency, phenotype, and intracellular cytokine production of cultured CD4^+^ and CD8^+^ T cells.** PBMCs from healthy donors were cultured without IgG (Mock) or in the presence of IVIg or purified IgG pooled from non-exposed healthy controls (Non-exp HC), patients with moderate COVID-19 (COVID-Mod), or patients with severe COVID-19 (COVID-Sev). The panels show the viability, memory and regulatory phenotypes, and intracellular cytokine production of CD4^+^ and CD8^+^ T cells after culture. Twelve independent healthy PBMC donors served as biological replicates (n = 12), with all experimental conditions evaluated within each donor. Box plots show the median and interquartile range; whiskers indicate the minimum and maximum values, and dots denote outliers. *p ≤ 0.05 compared with the Mock, IVIg, and Non-exp HC conditions.

The cytokine data indicated that severe-COVID IgG promoted a proinflammatory T-cell profile. In CD4^+^ T cells, COVID-Sev IgG increased IFN-γ production while reducing IL-9, IL-17 A, and IL-22 production. In CD8^+^ T cells, COVID-Sev IgG increased IFN-γ and reduced IL-10. Increased IFN-γ has been associated with mortality risk in moderate and severe COVID-19 [31], and experimental work has shown that IFN-γ can synergize with TNF-alpha to drive inflammatory cell death during cytokine shock [32]. The simultaneous reduction in IL-9 is also relevant because a reduced IL-9/IFN-γ balance has been proposed as a marker of unfavorable COVID-19 progression [33]. In contrast, COVID-Mod IgG selectively increased IL-22-producing CD8^+^ T cells. IL-22 is not simply a proinflammatory mediator; it can support epithelial repair and tissue-protective responses in respiratory viral injury [34,35]. Increased IL-22-producing CD8^+^ T cells have also been described in less complicated COVID-19 courses [36]. Thus, the cytokine profile induced by severe-COVID IgG appears compatible with inflammatory amplification, whereas the moderate-COVID IgG profile suggests a more balanced regulatory/tissue-protective imprint (Fig. 1).

Small-RNA profiling provided an additional layer of evidence that disease-severity-associated IgG repertoires can influence immune-cell regulatory states. Across the experimental conditions, 49 known small RNAs were differentially detected, including tRNAs, snoRNAs, and miRNAs. A cluster of snoRNAs was downregulated in both COVID-IgG conditions compared with control IgG conditions, suggesting that some post-transcriptional effects may be shared after SARS-CoV-2-induced antibody responses. More selectively, nine miRNAs were downregulated in PBMCs exposed to COVID-Mod IgG but not COVID-Sev IgG: hsa-miR-99b, miR-26a-1, miR-26a-2, miR-155, let-7g, miR-146b, miR-142, miR-126, and miR-22. This signature is biologically plausible. The miR-99b/let-7e/miR-125a cluster regulates suppressive antigen-presenting-cell programs [37], whereas miR-155 is linked to PI3K/AKT signaling in SARS-CoV-2-infected cells [38]. miR-146-family regulation has been associated with inflammatory parameters in COVID-19 [39], and differential miRNA expression in peripheral blood has been reported in infected patients [40].

**Fig. 2:**
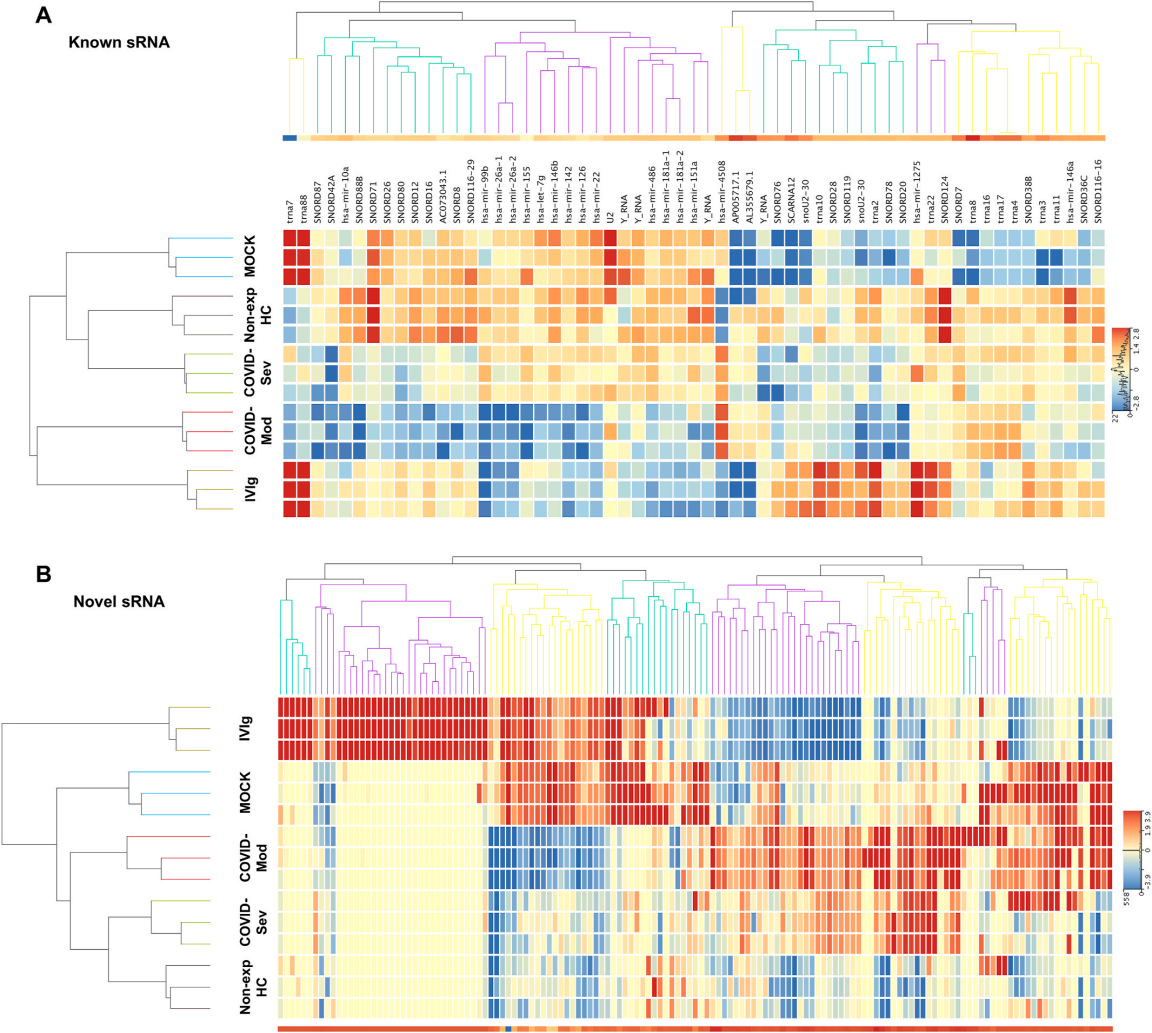
**COVID-19 IgG repertoires induce distinct small-RNA profiles in PBMCs.** Heatmaps show the relative abundance patterns of known (A) and novel (B) small RNAs following exposure to Mock, IVIg, non-exposed healthy-control IgG (Non-exp HC), moderate COVID-19 IgG (COVID-Mod), or severe COVID-19 IgG (COVID-Sev). For each exposure condition, PBMCs from 20 independent healthy donors were cultured individually, after which equal amounts of RNA from all donors were combined to generate one condition-specific RNA pool. Each pooled sample was analyzed using three library-preparation/sequencing replicates, which are displayed as rows in the heatmaps and represent technical rather than donor-level biological replicates. Columns represent individual small RNAs.

The observation that COVID-Mod, but not COVID-Sev, IgG induced this miRNA pattern suggests that antibody repertoires from moderate disease may engage compensatory post-transcriptional programs that are absent or attenuated in severe disease (Fig. 2).

Several of the miRNAs selectively altered by COVID-Mod IgG are also connected to pathways relevant to endothelial function, viral-entry biology, and immune homeostasis. miR-142 can target TIM-1 in endothelial cells, a molecule discussed in the context of viral infections including COVID-19 [41]. miR-126 has been associated with COVID-19 severity and inflammatory mRNA targets in patient serum [42]. let-7g and miR-22 have been characterized in lung cancer or pulmonary infection models, but their broader regulatory functions in cell proliferation, survival, and immune activity remain relevant to inflammatory disease biology [[43], [44], [45]]. These associations should not be overinterpreted as direct mechanistic proof, particularly because the small-RNA component of the present study used pooled material. Because this exploratory design does not estimate patient-level variance or permit clinical correlations, the miRNA signature should be regarded as hypothesis-generating. Nevertheless, the data indicate that IgG repertoires generated under different clinical severities can impose distinct regulatory signatures on PBMCs, supporting the broader premise that antibodies may influence immune programming beyond neutralization.

**Fig. 3:**
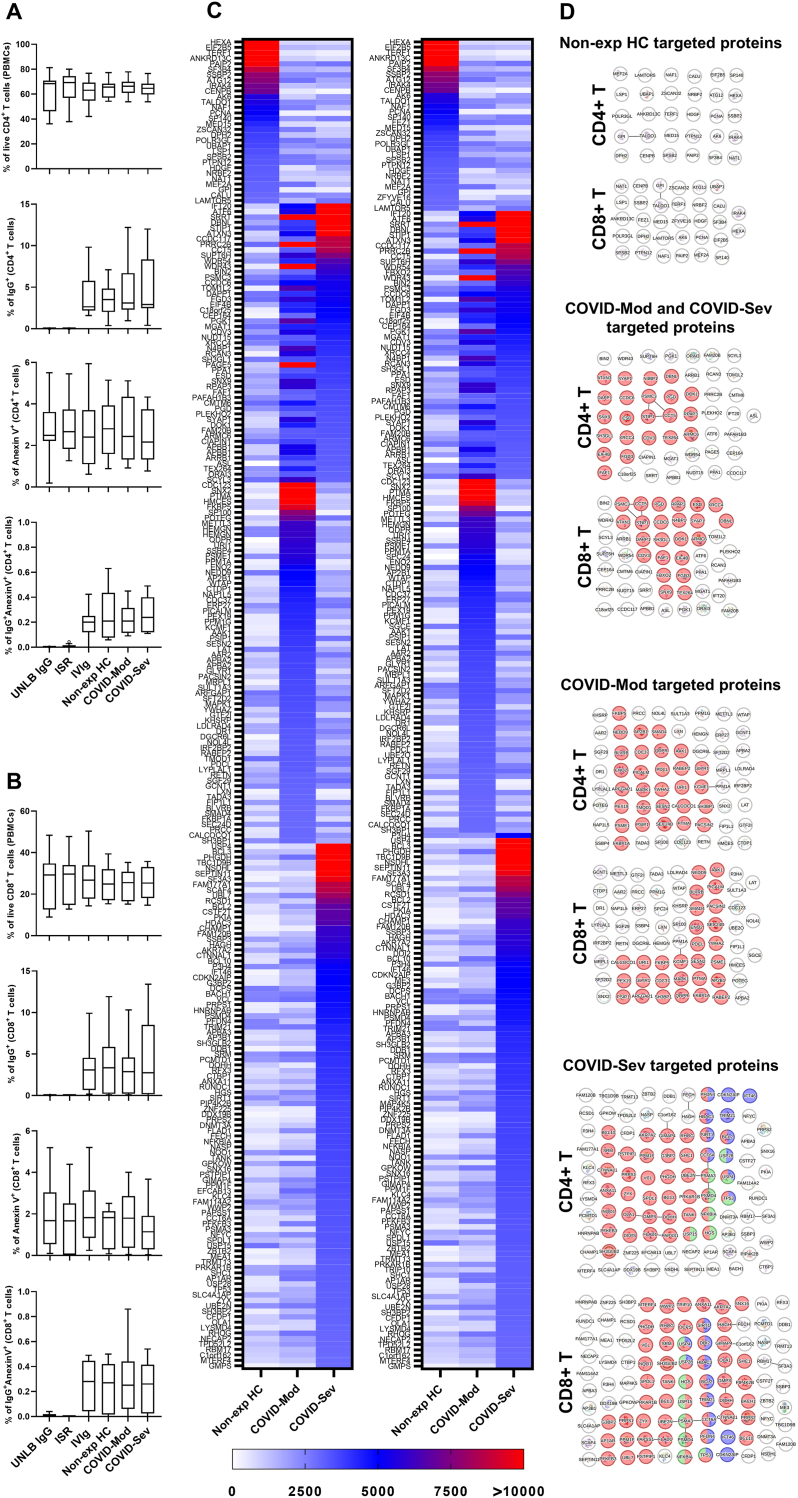
**COVID-19 IgG exhibits membrane binding and expanded reactivity against proteins expressed by CD4^+^ and CD8^+^ T cells.** Panels A and B show IgG membrane binding and Annexin V staining in CD4^+^ and CD8^+^ T cells, respectively. These cellular assays were performed using PBMCs from 12 independent healthy donors, which served as biological replicates (n = 12), with all experimental conditions evaluated within each donor in a matched design. Panel C shows HuProt proteome-microarray reactivity against proteins annotated as expressed in CD4^+^ or CD8^+^ T cells, whereas panel D presents STRING interaction networks of proteins recognized by IgG pooled from non-exposed healthy controls (Non-exp HC), patients with moderate COVID-19 (COVID-Mod), or patients with severe COVID-19 (COVID-Sev). The HuProt assays used the same group-specific pooled IgG preparations employed in the cellular experiments and therefore represent group-level antibody-repertoire.

Direct membrane-binding assays showed that approximately 3% of viable CD4^+^ and CD8^+^ T cells displayed IgG binding after a 30-min incubation. Importantly, early apoptosis remained uniformly low, and the overlap between IgG binding and Annexin V positivity was minimal. Therefore, membrane interaction could not be explained by nonspecific binding to dying cells (Fig. 3A and B). This distinction is important because severe COVID-19 has been associated with lymphocyte dysfunction and depletion, including alterations in individuals recovering from infection [46]. In the present system, purified IgG did not induce a broad apoptotic effect. Instead, the evidence supports a model in which a limited fraction of viable T cells is directly recognized by IgG, potentially creating sufficient signaling or receptor-proximal perturbation to alter cytokine output without overt cytotoxicity. This interpretation is consistent with prior work showing that patient-derived IgG can modulate immune-cell cytokine profiles while preserving viability in several disease contexts [10,12].

Proteome-wide mapping revealed that COVID-19 IgG repertoires recognized numerous proteins annotated as expressed in CD4^+^ and CD8^+^ T cells. For CD4^+^ T-cell-expressed proteins, 50 targets were shared by COVID-Mod and COVID-Sev IgG, whereas 64 were exclusive to COVID-Mod IgG and 96 were exclusive to COVID-Sev IgG. For CD8^+^ T-cell-expressed proteins, 50 targets were shared, 67 were exclusive to COVID-Mod IgG, and 99 were exclusive to COVID-Sev IgG; the complete list of targeted proteins is provided in Supplementary Table 2. STRING analyses showed limited sequence homology among targets, arguing against a simple model based only on cross-reactive peptide similarity. Instead, enrichment patterns suggested convergence on cytosolic localization, with severe-COVID IgG additionally enriching proteins involved in regulation of protein stability and ubiquitin-specific processing proteases (Fig. 3C and D). This profile is compatible with reports linking severe COVID-19 to autoantibodies against immune and non-immune targets [47,48] and with broader evidence that autoimmunity may represent a component of post-COVID syndrome [49], including persistent symptoms associated with autoantibody signatures [21]. The same framework includes diverse functional autoantibodies and new-onset IgG autoantibodies in infected or hospitalized patients [50,51]. Importantly, SARS-CoV-2-associated autoreactivity is not restricted to IgG: Pascolini et al. described three patients with COVID-19-associated immune thrombocytopenia in whom IgM anti-platelet autoantibodies were detected, providing a clinically relevant example of infection-associated autoreactivity against a blood-cell target [52]. These observations place the T-cell-expressed targets identified here within a multispecific, multi-isotype spectrum of COVID-19-associated autoreactivity.

The enrichment of protein-stability and ubiquitin-related targets is of particular interest for autoimmunity-oriented interpretation. The ubiquitin-proteasome system regulates protein turnover, antigen processing, cellular stress responses, and immune activation. Perturbation of these pathways may influence how intracellular proteins are processed, presented, or exposed to the immune system during severe infection. Although the present study does not establish causality between IgG recognition of these targets and clinical outcomes, the association between severe-COVID IgG and proteostasis-related autoreactivity supports the idea that severe infection leaves a broader antibody-mediated immunological scar. Such a scar may persist independently of acute viral replication and may contribute to prolonged immune dysregulation. This interpretation aligns with the broader literature describing autoantibodies in life-threatening COVID-19 [47], clinical autoimmune features in severe disease [48], and autoantibody associations in post-COVID manifestations [49].

The pattern observed here also helps reconcile two apparently conflicting views of anti-SARS-CoV-2 humoral immunity. On one hand, antibodies are essential components of antiviral defense and may indicate effective adaptive immunity. On the other hand, some antibody specificities induced during infection may participate in immune deviation, particularly when they recognize self-proteins or alter regulatory lymphocyte circuits. The present data do not imply that all COVID-19 IgG is pathogenic. Rather, they suggest that the repertoire generated during severe disease contains a larger autoreactive component and can bias T cells toward inflammatory cytokine production. This distinction is clinically meaningful because post-infectious autoimmunity is unlikely to depend on a single autoantibody specificity in all patients.

It may instead reflect a composite repertoire in which multiple low-frequency specificities converge on immune regulation, tissue repair, or intracellular stress pathways. Such a repertoire-level model is compatible with the heterogeneous clinical presentation of post-COVID manifestations and with the absence of a universal autoantibody marker across all affected individuals.

The study has limitations that should be considered when interpreting the findings. First, each clinical group was represented by a pooled IgG preparation, which prevents patient-level estimates of variability, associations with clinical characteristics, and assessment of inter-individual heterogeneity. The pooled small-RNA design likewise precludes donor-resolved validation; culture or technical replicates cannot substitute for patient-level biological replication. Second, the functional readouts were obtained after a short-term, 72-h *in vitro* exposure of healthy-donor PBMCs. Samples were collected during acute disease, and no longitudinal, convalescent, or long-COVID cohort was evaluated. Therefore, the present data neither establish persistence *in vivo* nor link the IgG effects causally to post-COVID phenotypes. Third, the protein microarray identifies candidate IgG targets but does not demonstrate that each target is accessible at the cell surface or functionally involved in cytokine modulation. Fourth, the study does not resolve whether the observed effects arise from SARS-CoV-2-specific or other IgG fractions, Fab- or Fc-dependent interactions, IgG subclass composition, Fc glycosylation, aggregation, immune complexes, or Fcγ-receptor engagement. Despite these limitations, the integrated design is a strength. SARS-CoV-2 epitope recognition established the disease-related nature of the IgG preparations; flow cytometry demonstrated functional modulation of T cells; membrane-binding assays excluded apoptosis as the main explanation for IgG interaction; small-RNA profiling identified severity-linked regulatory signatures; and proteome arrays revealed broader autoreactivity in severe disease. The convergence of these independent layers supports the central conclusion more strongly than any single assay alone.

In summary, SARS-CoV-2 infection generates IgG repertoires with disease-severity-associated functional properties *in vitro*. Severe-COVID IgG was characterized by selective Treg reduction, enhanced IFN-γ production by CD4^+^ and CD8^+^ T cells, suppression of selected CD4^+^ cytokines, and broader autoreactivity toward T-cell-expressed proteins linked to proteostasis. Moderate-COVID IgG induced a more balanced profile, including CD8^+^ IL-22 production and selective miRNA downregulation. These findings support an autoimmunity-relevant model in which infection-associated IgG repertoires may actively modulate cellular immunity and post-transcriptional regulation, while not establishing persistence or clinical causality. The work also highlights why antibody studies in COVID-19 should move beyond measuring antiviral binding or neutralization alone. Functional repertoire assays may reveal disease-associated properties that are invisible to conventional serology, particularly when antibody effects depend on immune-cell targets rather than viral antigens. Future patient-resolved and longitudinal studies should compare acute and convalescent samples, stratify post-acute clinical phenotypes, and determine whether the observed effects persist or identify individuals at risk of autoimmune sequelae. Mechanistic studies should use depletion or affinity purification of SARS-CoV-2-specific IgG, F (ab')2 and Fc fragments, Fcγ-receptor blockade, subclass and Fc-glycan profiling, aggregate and immune-complex assessment, and orthogonal validation of candidate targets.

## Ethics approval and consent to participate

The study was approved by the ethics committees at the School of Medicine and the Clinics Hospital of the University of Sao Paulo under registrations 3361622.7.0000.0068, 70823623.0.0000.0068, 3.983.577, and 5.264.948. Written informed consent was obtained from all participants.

## Funding

This study was funded by grants from the Laboratory of Medical Investigation-56, Medical School, University of Sao Paulo, Sao Paulo, Brazil (LIM-56 HC-10.13039/501100005640FMUSP), the 10.13039/501100003593National Council for Scientific and Technological Development (CNPq; grant no. 402406/2024-9), and Sao Paulo Research Foundation (FAPESP; grant no. 2021/08225-8).

## CRediT authorship contribution statement

**Fabio da Ressureição Sgnotto:** Investigation, Methodology. **Nicolle Rakanidis Machado:** Investigation, Methodology. **Lais Alves do Nascimento:** Methodology. **Beatriz Oliveira Fagundes:** Methodology. **Isabella Siuffi Bergamasco:** Methodology. **Sabri Saeed Sanabani:** Methodology, Writing – review & editing.

## Declaration of competing interest

The authors declare the following financial interests/personal relationships which may be considered as potential competing interests: Jefferson Russo Victor reports financial support was provided by State of Sao Paulo Research Foundation. If there are other authors, they declare that they have no known competing financial interests or personal relationships that could have appeared to influence the work reported in this paper.

## Data Availability

The data supporting the findings of this study are available from the corresponding author (JRV) upon reasonable request. Supplementary materials are publicly available at Zenodo: https://doi.org/10.5281/zenodo.18674702.
