# Supplementary material for: SARS-CoV-2-induced IgG repertoires shape T cell responses, microRNA regulation, and autoreactivity according to disease severity
Source: J Transl Autoimmun. 2026 Aug 3;13:100392. doi: 10.1016/j.jtauto.2026.100392 (PMC13476528; doi:10.1016/j.jtauto.2026.100392)
Supplement: Supplementary file 1 [file mmc1.docx]

**IVIg**

**N-exp HCs COVID-Mod COVID-Sev**

# SARS-Cov-2

**Epitope ID (IEDB)**

**> 4000**

**3000**

**2000**

**1000**

**0**

**Supplementary Figure S2: IgG Recognition of SARS-CoV-2 Epitopes Across Donor Groups.** This figure summarizes data from Infectious Disease Epitope Microarray profiling performed with serum or purified IgG from four groups: pooled intravenous immunoglobulin (IVIg, derived from thousands of donors), 40 healthy individuals unexposed to SARS-CoV-2 (N-exp HCs), 39 patients with moderate COVID-19 (COVID-Mod), and 40 patients with severe COVID-19 (COVID-Sev). Heatmaps depict IgG reactivity across the complete panel of SARS-CoV-2 epitopes catalogued in the IEDB resource. The epitopes are organized by their source proteins, presented from left to right as follows: membrane glycoprotein, nucleocapsid phosphoprotein, nucleoprotein, and spike glycoprotein. Reactivity values are expressed in arbitrary units (A.U.), calculated as the ratio of sample signal intensity to background signal intensity for each epitope.
