# Supplementary material for: SARS-CoV-2-induced IgG repertoires shape T cell responses, microRNA regulation, and autoreactivity according to disease severity
Source: J Transl Autoimmun. 2026 Aug 3;13:100392. doi: 10.1016/j.jtauto.2026.100392 (PMC13476528; doi:10.1016/j.jtauto.2026.100392)
Supplement: Supplementary file 2 [file mmc2.docx]

**CD4+** **T** **cells** **phenotypes:**

**Treg:** Lympho+CD4+CD25+CD127-**TemRA:** Lympho+CD4+CD45RA+CCR7-**Tcm:** Lympho+CD4+CD45R0+CCR7+ **Tem:** Lympho+CD4+CD45R0+CCR7-

**Trm:** Lympho+CD4+CD45R0+CCR7-CD69+

**CD8+** **T** **cells** **phenotypes:**

**TemRA:** Lympho+CD8+CD45RA+CCR7-**Tcm:** Lympho+CD8+CD45R0+CCR7+ **Tem:** Lympho+CD8+CD45R0+CCR7-

**Trm:** Lympho+CD8+CD45R0+CCR7-CD69+

**Supplementary** **Figure** **S1.** **Gating** **strategy** **used** **to** **identify** **CD4⁺ and** **CD8⁺ T-cell** **populations.** Arrows indicate the sequential gating steps applied to define each phenotype within both CD4⁺ and CD8⁺ subsets. Abbreviations: Treg, CD4⁺ regulatory T cells; TemRA, effector memory T cells re-expressing CD45RA; Tcm, central memory T cells; Tem, effector memory T cells; Trm, tissue-resident memory T cells.
