# Supplementary material for: SARS-CoV-2-induced IgG repertoires shape T cell responses, microRNA regulation, and autoreactivity according to disease severity
Source: J Transl Autoimmun. 2026 Aug 3;13:100392. doi: 10.1016/j.jtauto.2026.100392 (PMC13476528; doi:10.1016/j.jtauto.2026.100392)
Supplement: Supplementary file 3 [file mmc3.docx]

| **Supplementary Table 2. Full list of proteins targeted by each evaluated IgG formulation in relation to CD4⁺ and CD8⁺ T cells.** This dataset corresponds to the proteins illustrated in Figure 3C. | | | | |
| --- | --- | --- | --- | --- |
| **CD4+ T cells targeted proteins** | |  | **CD8+ T cells Targeted proteins** | |
| **IgG formulation** | **Acronym of the targeted protein** |  | **IgG formulation** | **Acronym of the targeted protein** |
| **Non-exp HC** | HEXA |  | **Non-exp HC** | HEXA |
|  | EIF2B5 |  |  | EIF2B5 |
|  | TERF1 |  |  | TERF1 |
|  | ANKRD13C |  |  | ANKRD13C |
|  | PAIP2 |  |  | PAIP2 |
|  | SF3B4 |  |  | SF3B4 |
|  | SSBP2 |  |  | SSBP2 |
|  | ATG12 |  |  | ATG12 |
|  | IRAK4 |  |  | IRAK4 |
|  | CENPB |  |  | CENPB |
|  | AK6 |  |  | AK6 |
|  | TALDO1 |  |  | TALDO1 |
|  | NAF1 |  |  | NAF1 |
|  | PCNA |  |  | PCNA |
|  | SP140 |  |  | SP140 |
|  | MED15 |  |  | FEZ1 |
|  | ZSCAN32 |  |  | MED15 |
|  | DPH2 |  |  | ZSCAN32 |
|  | POLR3GL |  |  | DPH2 |
|  | UBAP1 |  |  | POLR3GL |
|  | LSP1 |  |  | UBAP1 |
|  | SPSB2 |  |  | LSP1 |
|  | PTPN12 |  |  | SPSB2 |
|  | HDGF |  |  | PTPN12 |
|  | NRBF2 |  |  | HDGF |
|  | NAT1 |  |  | NRBF2 |
|  | MEF2A |  |  | NAT1 |
|  | GPI |  |  | MEF2A |
|  | CALU |  |  | GPI |
|  | LAMTOR5 |  |  | ZFYVE16 |
| **Both COVID-Mod and COVID-Sev goups** | IFT20 |  |  | CALU |
|  | ATF6 |  |  | LAMTOR5 |
|  | SRRT |  | **Both COVID-Mod and COVID-Sev goups** | IFT20 |
|  | DBNL |  |  | ATF6 |
|  | STIP1 |  |  | SRRT |
|  | ATXN3 |  |  | DBNL |
|  | CCDC117 |  |  | STIP1 |
|  | PRRC2B |  |  | ATXN3 |
|  | CCT5 |  |  | CCDC117 |
|  | SUPT6H |  |  | PRRC2B |
|  | WDR54 |  |  | CCT5 |
|  | WDR43 |  |  | SUPT6H |
|  | BIN2 |  |  | WDR54 |
|  | PSMC3 |  |  | FBXO2 |
|  | CCDC6 |  |  | WDR43 |
|  | TOM1L2 |  |  | BIN2 |
|  | DAPP1 |  |  | PSMC3 |
|  | FGD3 |  |  | CCDC6 |
|  | EIF4B |  |  | TOM1L2 |
|  | C18orf25 |  |  | DAPP1 |
|  | CEP164 |  |  | FGD3 |
|  | PGK1 |  |  | EIF4B |
|  | MGAT1 |  |  | C18orf25 |
|  | CDV3 |  |  | CEP164 |
|  | NUDT15 |  |  | PGK1 |
|  | XRCC4 |  |  | MGAT1 |
|  | N4BP1 |  |  | CDV3 |
|  | RCAN3 |  |  | NUDT15 |
|  | SH3GL1 |  |  | XRCC4 |
|  | PAGE5 |  |  | N4BP1 |
|  | PPA1 |  |  | RCAN3 |
|  | ESD |  |  | SH3GL1 |
|  | SNX9 |  |  | PPA1 |
|  | RPAP3 |  |  | ESD |
|  | FAF1 |  |  | SNX9 |
|  | PAFAH1B3 |  |  | RPAP3 |
|  | CMTM6 |  |  | FAF1 |
|  | PGD |  |  | PAFAH1B3 |
|  | PLEKHO2 |  |  | CMTM6 |
|  | SYAP1 |  |  | PGD |
|  | DOK1 |  |  | PLEKHO2 |
|  | FAM20B |  |  | SYAP1 |
|  | ARMC6 |  |  | DOK1 |
|  | CIAPIN1 |  |  | FAM20B |
|  | APBB1 |  |  | ARMC6 |
|  | APBB1 |  |  | CIAPIN1 |
|  | ARRB1 |  |  | APBB1 |
|  | ASL |  |  | ARRB1 |
|  | TEX264 |  |  | ASL |
|  | ORAI3 |  |  | TEX264 |
|  | SCYL3 |  |  | ORAI3 |
| **COVID-Mod** | CDC123 |  |  | SCYL3 |
|  | SNX2 |  | **COVID-Mod** | CDC123 |
|  | PTMA |  |  | SNX2 |
|  | HMCES |  |  | PTMA |
|  | FKBP5 |  |  | HMCES |
|  | SP100 |  |  | FKBP5 |
|  | POTEG |  |  | SP100 |
|  | METTL3 |  |  | POTEG |
|  | HEMGN |  |  | METTL3 |
|  | HEMGN |  |  | HEMGN |
|  | QDPR |  |  | QDPR |
|  | URI1 |  |  | URI1 |
|  | SSBP4 |  |  | SSBP4 |
|  | PSME1 |  |  | PSME1 |
|  | PPM1A |  |  | PPM1A |
|  | ENO2 |  |  | SPC24 |
|  | NEDD9 |  |  | ENO2 |
|  | AP2B1 |  |  | NEDD9 |
|  | WTAP |  |  | AP2B1 |
|  | CTDP1 |  |  | WTAP |
|  | NAP1L5 |  |  | CTDP1 |
|  | CDC37 |  |  | NAP1L5 |
|  | ERP27 |  |  | CDC37 |
|  | PICALM |  |  | ERP27 |
|  | PEX19 |  |  | PICALM |
|  | PPM1G |  |  | PEX19 |
|  | KCMF1 |  |  | PPM1G |
|  | AAK1 |  |  | KCMF1 |
|  | PSIP1 |  |  | SGCE |
|  | SESN2 |  |  | AAK1 |
|  | LAT |  |  | PSIP1 |
|  | AAR2 |  |  | SESN2 |
|  | APBA2 |  |  | LAT |
|  | APBA2 |  |  | AAR2 |
|  | GLYR1 |  |  | APBA2 |
|  | PACSIN2 |  |  | GLYR1 |
|  | MRPL1 |  |  | PACSIN2 |
|  | SULT1A3 |  |  | MRPL1 |
|  | ARFGAP1 |  |  | SULT1A3 |
|  | SFT2D2 |  |  | ARFGAP1 |
|  | MAPK1 |  |  | SFT2D2 |
|  | YWHAZ |  |  | MAPK1 |
|  | GTF2I |  |  | YWHAZ |
|  | KHSRP |  |  | GTF2I |
|  | LDLRAD4 |  |  | KHSRP |
|  | DR1 |  |  | LDLRAD4 |
|  | DGCR6L |  |  | DR1 |
|  | NOL4L |  |  | DGCR6L |
|  | IRF2BP2 |  |  | NOL4L |
|  | RABEP2 |  |  | IRF2BP2 |
|  | TMOD1 |  |  | RABEP2 |
|  | PDCL |  |  | PDCL |
|  | LYPLAL1 |  |  | UBE2O |
|  | RETN |  |  | LYPLAL1 |
|  | SGF29 |  |  | RETN |
|  | GCNT1 |  |  | SGF29 |
|  | LXN |  |  | GCNT1 |
|  | TADA3 |  |  | LXN |
|  | FIP1L1 |  |  | TADA3 |
|  | BLVRB |  |  | FIP1L1 |
|  | SMAD4 |  |  | BLVRB |
|  | FKBP1A |  |  | SMAD4 |
|  | SEC24D |  |  | FKBP1A |
|  | PRCC |  |  | SEC24D |
|  | CALCOCO1 |  |  | PRCC |
|  | SH3BP1 |  |  | CALCOCO1 |
| **COVID-Sev** | USP4 |  |  | SH3BP1 |
|  | BCL3 |  |  | P3H4 |
|  | PHGDH |  | **COVID-Sev** | USP4 |
|  | TBC1D9B |  |  | BCL3 |
|  | NSDHL |  |  | PHGDH |
|  | SEPTIN11 |  |  | TBC1D9B |
|  | SF3A3 |  |  | NSDHL |
|  | FAM177A1 |  |  | SEPTIN11 |
|  | SCAF4 |  |  | SF3A3 |
|  | UBL7 |  |  | FAM177A1 |
|  | RCSD1 |  |  | SCAF4 |
|  | BCL2 |  |  | UBL7 |
|  | CSTF2T |  |  | RCSD1 |
|  | PKIA |  |  | BCL2 |
|  | HDAC3 |  |  | CSTF2T |
|  | CHAMP1 |  |  | PKIA |
|  | FAM120B |  |  | HDAC3 |
|  | SSBP3 |  |  | CHAMP1 |
|  | HAGH |  |  | FAM120B |
|  | AKR7A2 |  |  | SSBP3 |
|  | CTNNAL1 |  |  | HAGH |
|  | BCL10 |  |  | AKR7A2 |
|  | P3H4 |  |  | CTNNAL1 |
|  | IFT46 |  |  | DDI2 |
|  | CDKN2AIP |  |  | BCL10 |
|  | G3BP2 |  |  | P3H4 |
|  | DCPS |  |  | IFT46 |
|  | BACH1 |  |  | CDKN2AIP |
|  | VCL |  |  | ME3 |
|  | PRPS1 |  |  | G3BP2 |
|  | HNRNPAB |  |  | DCPS |
|  | PSMD4 |  |  | BACH1 |
|  | PFDN4 |  |  | VCL |
|  | TRIM21 |  |  | PRPS1 |
|  | APBA3 |  |  | HNRNPAB |
|  | AP3B1 |  |  | PSMD4 |
|  | SH3GLB2 |  |  | PFDN4 |
|  | DDB1 |  |  | TRIM21 |
|  | SRM |  |  | APBA3 |
|  | PCMTD1 |  |  | AP3B1 |
|  | DOHH |  |  | SH3GLB2 |
|  | RFX3 |  |  | DDB1 |
|  | CTBP1 |  |  | SRM |
|  | ANXA11 |  |  | PCMTD1 |
|  | RUNDC1 |  |  | DOHH |
|  | HGS |  |  | RFX3 |
|  | SIRT1 |  |  | CTBP1 |
|  | PIP4K2B |  |  | ANXA11 |
|  | ZNF225 |  |  | RUNDC1 |
|  | DDX19B |  |  | HGS |
|  | PRPS2 |  |  | SIRT1 |
|  | DNMT3A |  |  | MAP4K5 |
|  | FLAD1 |  |  | PIP4K2B |
|  | FECH |  |  | ZNF225 |
|  | NFKBIA |  |  | DDX19B |
|  | NASP |  |  | PRPS2 |
|  | NQO1 |  |  | DNMT3A |
|  | TANK |  |  | FLAD1 |
|  | GPKOW |  |  | FECH |
|  | SNX16 |  |  | NFKBIA |
|  | PSTPIP1 |  |  | NASP |
|  | GIMAP4 |  |  | NQO1 |
|  | PPM1F |  |  | TANK |
|  | EFCAB13 |  |  | GPKOW |
|  | KLC4 |  |  | SNX16 |
|  | FAM114A2 |  |  | PSTPIP1 |
|  | WWP2 |  |  | GIMAP4 |
|  | PAPSS1 |  |  | PPM1F |
|  | CCT6A |  |  | KLC4 |
|  | PFKFB3 |  |  | FAM114A2 |
|  | PSMA3 |  |  | WWP2 |
|  | NFYC |  |  | PAPSS1 |
|  | SPDL1 |  |  | CCT6A |
|  | USP15 |  |  | PFKFB3 |
|  | ZBTB2 |  |  | PSMA3 |
|  | MEA1 |  |  | NFYC |
|  | TRMT13 |  |  | SPDL1 |
|  | PRKAR1B |  |  | USP15 |
|  | SHC1 |  |  | ZBTB2 |
|  | AP1AR |  |  | MEA1 |
|  | USP28 |  |  | TRMT13 |
|  | TP53 |  |  | PRKAR1B |
|  | SLC4A1AP |  |  | TRIP10 |
|  | ZYX |  |  | SHC1 |
|  | UBE2N |  |  | AP1AR |
|  | SH3BP2 |  |  | USP28 |
|  | CFDP1 |  |  | TP53 |
|  | OLA1 |  |  | SLC4A1AP |
|  | LYSMD4 |  |  | ZYX |
|  | RHOG |  |  | UBE2N |
|  | NECAP2 |  |  | SH3BP2 |
|  | TPD52L2 |  |  | CFDP1 |
|  | RBM17 |  |  | OLA1 |
|  | C1orf162 |  |  | LYSMD4 |
|  | MTERF4 |  |  | RHOG |
|  | GMPS |  |  | NECAP2 |
|  |  |  |  | TPD52L2 |
|  |  |  |  | RBM17 |
|  |  |  |  | C1orf162 |
|  |  |  |  | MTERF4 |
|  |  |  |  | GMPS |
