# Supplementary material for: SARS-CoV-2-induced IgG repertoires shape T cell responses, microRNA regulation, and autoreactivity according to disease severity
Source: J Transl Autoimmun. 2026 Aug 3;13:100392. doi: 10.1016/j.jtauto.2026.100392 (PMC13476528; doi:10.1016/j.jtauto.2026.100392)
Supplement: Supplementary file 4 [file mmc4.docx]

**Supplementary Table 1.** Full list of all proteins evaluated that are expressed in CD4⁺ (page 1) and CD8⁺ (page 182) T cells, including their acronyms, full names, and UniProt IDs.

| **CD4+ T cells evaluated proteins** | | |
| --- | --- | --- |
| **Acronym** | **Protein name** | **UniprotID** |
| AAAS | Aladin WD repeat nucleoporin | Q9NRG9 |
| AACS | Acetoacetyl-CoA synthetase | Q86V21 |
| AAGAB | Alpha and gamma adaptin binding protein | Q6PD74 |
| AAK1 | AP2 associated kinase 1 | Q2M2I8 |
| AAMDC | Adipogenesis associated Mth938 domain containing | Q9H7C9 |
| AAMP | Angio associated migratory cell protein | Q13685 |
| AAR2 | AAR2 splicing factor | Q9Y312 |
| AARSD1 | Alanyl-tRNA synthetase domain containing 1 | Q9BTE6 |
| AASDHPPT | Aminoadipate-semialdehyde dehydrogenase-phosphopantetheinyl transferase | Q9NRN7 |
| AATF | Apoptosis antagonizing transcription factor | Q9NY61 |
| ABAT | 4-aminobutyrate aminotransferase | P80404 |
| ABCA7 | ATP binding cassette subfamily A member 7 | Q8IZY2 |
| ABCB7 | ATP binding cassette subfamily B member 7 | O75027 |
| ABCC10 | ATP binding cassette subfamily C member 10 | Q5T3U5 |
| ABCC4 | ATP binding cassette subfamily C member 4 | O15439 |
| ABCC5 | ATP binding cassette subfamily C member 5 | O15440 |
| ABCD1 | ATP binding cassette subfamily D member 1 | P33897 |
| ABCD3 | ATP binding cassette subfamily D member 3 | P28288 |
| ABCD4 | ATP binding cassette subfamily D member 4 | O14678 |
| ABCE1 | ATP binding cassette subfamily E member 1 | P61221 |
| ABCF1 | ATP binding cassette subfamily F member 1 | Q8NE71 |
| ABCF2 | ATP binding cassette subfamily F member 2 | Q9UG63 |
| ABCF3 | ATP binding cassette subfamily F member 3 | Q9NUQ8 |
| ABCG1 | ATP binding cassette subfamily G member 1 | P45844 |
| ABHD10 | Abhydrolase domain containing 10, depalmitoylase | Q9NUJ1 |
| ABHD11 | Abhydrolase domain containing 11 | Q8NFV4 |
| ABHD12 | Abhydrolase domain containing 12, lysophospholipase | Q8N2K0 |
| ABHD13 | Abhydrolase domain containing 13 | Q7L211 |
| ABHD14A | Abhydrolase domain containing 14A | Q9BUJ0 |
| ABHD14B | Abhydrolase domain containing 14B | Q96IU4 |
| ABHD15 | Abhydrolase domain containing 15 | Q6UXT9 |
| ABHD16A | Abhydrolase domain containing 16A, phospholipase | O95870 |
| ABHD17A | Abhydrolase domain containing 17A, depalmitoylase | Q96GS6 |
| ABHD17B | Abhydrolase domain containing 17B, depalmitoylase | Q5VST6 |
| ABHD18 | Abhydrolase domain containing 18 | Q0P651 |
| ABHD2 | Abhydrolase domain containing 2, acylglycerol lipase | P08910 |
| ABHD3 | Abhydrolase domain containing 3, phospholipase | Q8WU67 |
| ABHD4 | Abhydrolase domain containing 4, N-acyl phospholipase B | Q8TB40 |
| ABHD5 | Abhydrolase domain containing 5, lysophosphatidic acid acyltransferase | Q8WTS1 |
| ABHD6 | Abhydrolase domain containing 6, acylglycerol lipase | Q9BV23 |
| ABHD8 | Abhydrolase domain containing 8 | Q96I13 |
| ABI1 | Abl interactor 1 | Q8IZP0 |
| ABI2 | Abl interactor 2 | Q9NYB9 |
| ABI3 | ABI family member 3 | Q9P2A4 |
| ABLIM1 | Actin binding LIM protein 1 | O14639 |
| ABRACL | ABRA C-terminal like | Q9P1F3 |
| ABT1 | Activator of basal transcription 1 | Q9ULW3 |
| ABTB1 | Ankyrin repeat and BTB domain containing 1 | Q969K4 |
| ACAA1 | Acetyl-CoA acyltransferase 1 | P09110 |
| ACAA2 | Acetyl-CoA acyltransferase 2 | P42765 |
| ACAD10 | Acyl-CoA dehydrogenase family member 10 | Q6JQN1 |
| ACAD8 | Acyl-CoA dehydrogenase family member 8 | Q9UKU7 |
| ACAD9 | Acyl-CoA dehydrogenase family member 9 | Q9H845 |
| ACADM | Acyl-CoA dehydrogenase medium chain | P11310 |
| ACADS | Acyl-CoA dehydrogenase short chain | P16219 |
| ACADSB | Acyl-CoA dehydrogenase short/branched chain | P45954 |
| ACADVL | Acyl-CoA dehydrogenase very long chain | P49748 |
| ACAP1 | ArfGAP with coiled-coil, ankyrin repeat and PH domains 1 | Q15027 |
| ACAP2 | ArfGAP with coiled-coil, ankyrin repeat and PH domains 2 | Q15057 |
| ACAT1 | Acetyl-CoA acetyltransferase 1 | P24752 |
| ACAT2 | Acetyl-CoA acetyltransferase 2 | Q9BWD1 |
| ACBD3 | Acyl-CoA binding domain containing 3 | Q9H3P7 |
| ACBD4 | Acyl-CoA binding domain containing 4 | Q8NC06 |
| ACBD5 | Acyl-CoA binding domain containing 5 | Q5T8D3 |
| ACBD6 | Acyl-CoA binding domain containing 6 | Q9BR61 |
| ACCS | 1-aminocyclopropane-1-carboxylate synthase homolog (inactive) | Q96QU6 |
| ACD | ACD shelterin complex subunit and telomerase recruitment factor | Q96AP0 |
| ACKR3 | Atypical chemokine receptor 3 | P25106 |
| ACO1 | Aconitase 1 | P21399 |
| ACO2 | Aconitase 2 | Q99798 |
| ACOT1 | Acyl-CoA thioesterase 1 | Q86TX2 |
| ACOT13 | Acyl-CoA thioesterase 13 | Q9NPJ3 |
| ACOT2 | Acyl-CoA thioesterase 2 | P49753 |
| ACOT4 | Acyl-CoA thioesterase 4 | Q8N9L9 |
| ACOT7 | Acyl-CoA thioesterase 7 | O00154 |
| ACOT8 | Acyl-CoA thioesterase 8 | O14734 |
| ACOT9 | Acyl-CoA thioesterase 9 | Q9Y305 |
| ACOX1 | Acyl-CoA oxidase 1 | Q15067 |
| ACOX3 | Acyl-CoA oxidase 3, pristanoyl | O15254 |
| ACP1 | Acid phosphatase 1 | P24666 |
| ACP2 | Acid phosphatase 2, lysosomal | P11117 |
| ACP5 | Acid phosphatase 5, tartrate resistant | P13686 |
| ACP6 | Acid phosphatase 6, lysophosphatidic | Q9NPH0 |
| ACSF2 | Acyl-CoA synthetase family member 2 | Q96CM8 |
| ACSF3 | Acyl-CoA synthetase family member 3 | Q4G176 |
| ACSL3 | Acyl-CoA synthetase long chain family member 3 | O95573 |
| ACSL4 | Acyl-CoA synthetase long chain family member 4 | O60488 |
| ACSL5 | Acyl-CoA synthetase long chain family member 5 | Q9ULC5 |
| ACSL6 | Acyl-CoA synthetase long chain family member 6 | Q9UKU0 |
| ACSM3 | Acyl-CoA synthetase medium chain family member 3 | Q53FZ2 |
| ACSS1 | Acyl-CoA synthetase short chain family member 1 | Q9NUB1 |
| ACSS2 | Acyl-CoA synthetase short chain family member 2 | Q9NR19 |
| ACTA1 | Actin alpha 1, skeletal muscle | P68133 |
| ACTA2 | Actin alpha 2, smooth muscle | P62736 |
| ACTB | Actin beta | P60709 |
| ACTG1 | Actin gamma 1 | P63261 |
| ACTL6A | Actin like 6A | O96019 |
| ACTN1 | Actinin alpha 1 | P12814 |
| ACTN4 | Actinin alpha 4 | O43707 |
| ACTR10 | Actin related protein 10 | Q9NZ32 |
| ACTR1A | Actin related protein 1A | P61163 |
| ACTR1B | Actin related protein 1B | P42025 |
| ACTR2 | Actin related protein 2 | P61160 |
| ACTR3 | Actin related protein 3 | P61158 |
| ACTR3B | Actin related protein 3B | Q9P1U1 |
| ACTR6 | Actin related protein 6 | Q9GZN1 |
| ACTR8 | Actin related protein 8 | Q9H981 |
| ACVR1 | Activin A receptor type 1 | Q04771 |
| ACVR2A | Activin A receptor type 2A | P27037 |
| ACY1 | Aminoacylase 1 | Q03154 |
| ACYP1 | Acylphosphatase 1 | P07311 |
| ACYP2 | Acylphosphatase 2 | P14621 |
| ADA | Adenosine deaminase | P00813 |
| ADAL | Adenosine deaminase like | Q6DHV7 |
| ADAM15 | ADAM metallopeptidase domain 15 | Q13444 |
| ADAM19 | ADAM metallopeptidase domain 19 | Q9H013 |
| ADAP1 | ArfGAP with dual PH domains 1 | O75689 |
| ADAR | Adenosine deaminase RNA specific | P55265 |
| ADAT1 | Adenosine deaminase tRNA specific 1 | Q9BUB4 |
| ADAT2 | Adenosine deaminase tRNA specific 2 | Q7Z6V5 |
| ADAT3 | Adenosine deaminase tRNA specific 3 |  |
| ADCK1 | AarF domain containing kinase 1 | Q86TW2 |
| ADCK2 | AarF domain containing kinase 2 | Q7Z695 |
| ADD1 | Adducin 1 | P35611 |
| ADGRG5 | Adhesion G protein-coupled receptor G5 | Q8IZF4 |
| ADH5 | Alcohol dehydrogenase 5 (class III), chi polypeptide | P11766 |
| ADI1 | Acireductone dioxygenase 1 | Q9BV57 |
| ADIPOR1 | Adiponectin receptor 1 | Q96A54 |
| ADIRF | Adipogenesis regulatory factor | Q15847 |
| ADK | Adenosine kinase | P55263 |
| ADNP | Activity dependent neuroprotector homeobox | Q9H2P0 |
| ADNP2 | ADNP homeobox 2 | Q6IQ32 |
| ADORA2A | Adenosine A2a receptor | P29274 |
| ADPGK | ADP dependent glucokinase | Q9BRR6 |
| ADPRH | ADP-ribosylarginine hydrolase | P54922 |
| ADPRM | ADP-ribose/CDP-alcohol diphosphatase, manganese dependent | Q3LIE5 |
| ADRB2 | Adrenoceptor beta 2 | P07550 |
| ADRM1 | ADRM1 26S proteasome ubiquitin receptor | Q16186 |
| ADSL | Adenylosuccinate lyase | P30566 |
| ADTRP | Androgen dependent TFPI regulating protein | Q96IZ2 |
| AEBP2 | AE binding protein 2 | Q6ZN18 |
| AEN | Apoptosis enhancing nuclease | Q8WTP8 |
| AFAP1L2 | Actin filament associated protein 1 like 2 | Q8N4X5 |
| AFF1 | ALF transcription elongation factor 1 | P51825 |
| AFF4 | ALF transcription elongation factor 4 | Q9UHB7 |
| AFG3L2 | AFG3 like matrix AAA peptidase subunit 2 | Q9Y4W6 |
| AFMID | Arylformamidase | Q63HM1 |
| AFTPH | Aftiphilin | Q6ULP2 |
| AGA | Aspartylglucosaminidase | P20933 |
| AGAP2 | ArfGAP with GTPase domain, ankyrin repeat and PH domain 2 | Q99490 |
| AGAP3 | ArfGAP with GTPase domain, ankyrin repeat and PH domain 3 | Q96P47 |
| AGBL3 | AGBL carboxypeptidase 3 | Q8NEM8 |
| AGBL5 | AGBL carboxypeptidase 5 | Q8NDL9 |
| AGFG1 | ArfGAP with FG repeats 1 | P52594 |
| AGFG2 | ArfGAP with FG repeats 2 | O95081 |
| AGGF1 | Angiogenic factor with G-patch and FHA domains 1 | Q8N302 |
| AGK | Acylglycerol kinase | Q53H12 |
| AGL | Amylo-alpha-1, 6-glucosidase, 4-alpha-glucanotransferase | P35573 |
| AGMAT | Agmatinase | Q9BSE5 |
| AGPAT1 | 1-acylglycerol-3-phosphate O-acyltransferase 1 | Q99943 |
| AGPAT2 | 1-acylglycerol-3-phosphate O-acyltransferase 2 | O15120 |
| AGPAT3 | 1-acylglycerol-3-phosphate O-acyltransferase 3 | Q9NRZ7 |
| AGPAT4 | 1-acylglycerol-3-phosphate O-acyltransferase 4 | Q9NRZ5 |
| AGPAT5 | 1-acylglycerol-3-phosphate O-acyltransferase 5 | Q9NUQ2 |
| AGTPBP1 | ATP/GTP binding carboxypeptidase 1 | Q9UPW5 |
| AGTRAP | Angiotensin II receptor associated protein | Q6RW13 |
| AHCY | Adenosylhomocysteinase | P23526 |
| AHCYL1 | Adenosylhomocysteinase like 1 | O43865 |
| AHI1 | Abelson helper integration site 1 | Q8N157 |
| AHNAK | AHNAK nucleoprotein | Q09666 |
| AHSA1 | Activator of HSP90 ATPase activity 1 | O95433 |
| AIDA | Axin interactor, dorsalization associated | Q96BJ3 |
| AIF1 | Allograft inflammatory factor 1 | P55008 |
| AIFM1 | Apoptosis inducing factor mitochondria associated 1 | O95831 |
| AIFM2 | Apoptosis inducing factor mitochondria associated 2 | Q9BRQ8 |
| AIG1 | Androgen induced 1 | Q9NVV5 |
| AIM2 | Absent in melanoma 2 | O14862 |
| AIMP1 | Aminoacyl tRNA synthetase complex interacting multifunctional protein 1 | Q12904 |
| AIMP2 | Aminoacyl tRNA synthetase complex interacting multifunctional protein 2 | Q13155 |
| AK2 | Adenylate kinase 2 | P54819 |
| AK3 | Adenylate kinase 3 | Q9UIJ7 |
| AK5 | Adenylate kinase 5 | Q9Y6K8 |
| AK6 | Adenylate kinase 6 | Q9Y3D8 |
| AK9 | Adenylate kinase 9 | Q5TCS8 |
| AKAP1 | A-kinase anchoring protein 1 | Q92667 |
| AKAP10 | A-kinase anchoring protein 10 | O43572 |
| AKAP13 | A-kinase anchoring protein 13 | Q12802 |
| AKAP7 | A-kinase anchoring protein 7 | O43687, Q9P0M2 |
| AKAP8 | A-kinase anchoring protein 8 | O43823 |
| AKAP8L | A-kinase anchoring protein 8 like | Q9ULX6 |
| AKIP1 | A-kinase interacting protein 1 | Q9NQ31 |
| AKIRIN2 | Akirin 2 | Q53H80 |
| AKNA | AT-hook transcription factor | Q7Z591 |
| AKR1A1 | Aldo-keto reductase family 1 member A1 | P14550 |
| AKR1B1 | Aldo-keto reductase family 1 member B | P15121 |
| AKR1C3 | Aldo-keto reductase family 1 member C3 | P42330 |
| AKR7A2 | Aldo-keto reductase family 7 member A2 | O43488 |
| AKT1 | AKT serine/threonine kinase 1 | P31749 |
| AKT2 | AKT serine/threonine kinase 2 | P31751 |
| AKTIP | AKT interacting protein | Q9H8T0 |
| ALAD | Aminolevulinate dehydratase | P13716 |
| ALAS1 | 5'-aminolevulinate synthase 1 | P13196 |
| ALDH16A1 | Aldehyde dehydrogenase 16 family member A1 | Q8IZ83 |
| ALDH18A1 | Aldehyde dehydrogenase 18 family member A1 | P54886 |
| ALDH1B1 | Aldehyde dehydrogenase 1 family member B1 | P30837 |
| ALDH3A2 | Aldehyde dehydrogenase 3 family member A2 | P51648 |
| ALDH5A1 | Aldehyde dehydrogenase 5 family member A1 | P51649 |
| ALDH6A1 | Aldehyde dehydrogenase 6 family member A1 | Q02252 |
| ALDH9A1 | Aldehyde dehydrogenase 9 family member A1 | P49189 |
| ALDOA | Aldolase, fructose-bisphosphate A | P04075 |
| ALDOC | Aldolase, fructose-bisphosphate C | P09972 |
| ALG1 | ALG1 chitobiosyldiphosphodolichol beta-mannosyltransferase | Q9BT22 |
| ALG10 | ALG10 alpha-1,2-glucosyltransferase | Q5BKT4 |
| ALG12 | ALG12 alpha-1,6-mannosyltransferase | Q9BV10 |
| ALG13 | ALG13 UDP-N-acetylglucosaminyltransferase subunit | Q9NP73 |
| ALG2 | ALG2 alpha-1,3/1,6-mannosyltransferase | Q9H553 |
| ALG3 | ALG3 alpha-1,3- mannosyltransferase | Q92685 |
| ALG5 | ALG5 dolichyl-phosphate beta-glucosyltransferase | Q9Y673 |
| ALG8 | ALG8 alpha-1,3-glucosyltransferase | Q9BVK2 |
| ALG9 | ALG9 alpha-1,2-mannosyltransferase | Q9H6U8 |
| ALKBH1 | AlkB homolog 1, histone H2A dioxygenase | Q13686 |
| ALKBH2 | AlkB homolog 2, alpha-ketoglutarate dependent dioxygenase | Q6NS38 |
| ALKBH3 | AlkB homolog 3, alpha-ketoglutarate dependent dioxygenase | Q96Q83 |
| ALKBH4 | AlkB homolog 4, lysine demethylase | Q9NXW9 |
| ALKBH5 | AlkB homolog 5, RNA demethylase | Q6P6C2 |
| ALKBH7 | AlkB homolog 7 | Q9BT30 |
| ALKBH8 | AlkB homolog 8, tRNA methyltransferase | Q96BT7 |
| ALOX5 | Arachidonate 5-lipoxygenase | P09917 |
| ALOX5AP | Arachidonate 5-lipoxygenase activating protein | P20292 |
| ALS2 | Alsin Rho guanine nucleotide exchange factor ALS2 | Q96Q42 |
| ALYREF | Aly/REF export factor |  |
| AMACR | Alpha-methylacyl-CoA racemase | Q9UHK6 |
| AMBRA1 | Autophagy and beclin 1 regulator 1 | Q9C0C7 |
| AMD1 | Adenosylmethionine decarboxylase 1 | P17707 |
| AMDHD2 | Amidohydrolase domain containing 2 | Q9Y303 |
| AMIGO1 | Adhesion molecule with Ig like domain 1 | Q86WK6 |
| AMIGO2 | Adhesion molecule with Ig like domain 2 | Q86SJ2 |
| AMMECR1 | AMMECR nuclear protein 1 | Q9Y4X0 |
| AMMECR1L | AMMECR1 like | Q6DCA0 |
| AMN1 | Antagonist of mitotic exit network 1 homolog | Q8IY45 |
| AMPD2 | Adenosine monophosphate deaminase 2 | Q01433 |
| AMPD3 | Adenosine monophosphate deaminase 3 | Q01432 |
| AMZ2 | Archaelysin family metallopeptidase 2 | Q86W34 |
| ANAPC10 | Anaphase promoting complex subunit 10 | Q9UM13 |
| ANAPC11 | Anaphase promoting complex subunit 11 | Q9NYG5 |
| ANAPC13 | Anaphase promoting complex subunit 13 | Q9BS18 |
| ANAPC15 | Anaphase promoting complex subunit 15 | P60006 |
| ANAPC16 | Anaphase promoting complex subunit 16 | Q96DE5 |
| ANAPC5 | Anaphase promoting complex subunit 5 | Q9UJX4 |
| ANG | Angiogenin | P03950 |
| ANGEL1 | Angel homolog 1 | Q9UNK9 |
| ANGEL2 | Angel homolog 2 | Q5VTE6 |
| ANK3 | Ankyrin 3 | Q12955 |
| ANKDD1A | Ankyrin repeat and death domain containing 1A | Q495B1 |
| ANKFY1 | Ankyrin repeat and FYVE domain containing 1 | Q9P2R3 |
| ANKH | ANKH inorganic pyrophosphate transport regulator | Q9HCJ1 |
| ANKHD1 | Ankyrin repeat and KH domain containing 1 | Q8IWZ3 |
| ANKLE2 | Ankyrin repeat and LEM domain containing 2 | Q86XL3 |
| ANKMY1 | Ankyrin repeat and MYND domain containing 1 | Q9P2S6 |
| ANKMY2 | Ankyrin repeat and MYND domain containing 2 | Q8IV38 |
| ANKRA2 | Ankyrin repeat family A member 2 | Q9H9E1 |
| ANKRD10 | Ankyrin repeat domain 10 | Q9NXR5 |
| ANKRD12 | Ankyrin repeat domain 12 | Q6UB98 |
| ANKRD13A | Ankyrin repeat domain 13A | Q8IZ07 |
| ANKRD13C | Ankyrin repeat domain 13C | Q8N6S4 |
| ANKRD13D | Ankyrin repeat domain 13D | Q6ZTN6 |
| ANKRD17 | Ankyrin repeat domain 17 | O75179 |
| ANKRD27 | Ankyrin repeat domain 27 | Q96NW4 |
| ANKRD36 | Ankyrin repeat domain 36 | A6QL64 |
| ANKRD37 | Ankyrin repeat domain 37 | Q7Z713 |
| ANKRD39 | Ankyrin repeat domain 39 | Q53RE8 |
| ANKRD40 | Ankyrin repeat domain 40 | Q6AI12 |
| ANKRD42 | Ankyrin repeat domain 42 | Q8N9B4 |
| ANKRD44 | Ankyrin repeat domain 44 | Q8N8A2 |
| ANKRD46 | Ankyrin repeat domain 46 | Q86W74 |
| ANKRD49 | Ankyrin repeat domain 49 | Q8WVL7 |
| ANKRD54 | Ankyrin repeat domain 54 | Q6NXT1 |
| ANKS1B | Ankyrin repeat and sterile alpha motif domain containing 1B | Q7Z6G8 |
| ANKZF1 | Ankyrin repeat and zinc finger peptidyl tRNA hydrolase 1 | Q9H8Y5 |
| ANO10 | Anoctamin 10 | Q9NW15 |
| ANO6 | Anoctamin 6 | Q4KMQ2 |
| ANP32A | Acidic nuclear phosphoprotein 32 family member A | P39687 |
| ANP32B | Acidic nuclear phosphoprotein 32 family member B | Q92688 |
| ANP32E | Acidic nuclear phosphoprotein 32 family member E | Q9BTT0 |
| ANTXR2 | ANTXR cell adhesion molecule 2 | P58335 |
| ANXA1 | Annexin A1 | P04083 |
| ANXA11 | Annexin A11 | P50995 |
| ANXA2 | Annexin A2 | P07355 |
| ANXA2R | Annexin A2 receptor | Q3ZCQ2 |
| ANXA4 | Annexin A4 | P09525 |
| ANXA5 | Annexin A5 | P08758 |
| ANXA6 | Annexin A6 | P08133 |
| ANXA7 | Annexin A7 | P20073 |
| AOAH | Acyloxyacyl hydrolase | P28039 |
| AOPEP | Aminopeptidase O (putative) | Q8N6M6 |
| AP1AR | Adaptor related protein complex 1 associated regulatory protein | Q63HQ0 |
| AP1B1 | Adaptor related protein complex 1 subunit beta 1 | Q10567 |
| AP1G1 | Adaptor related protein complex 1 subunit gamma 1 | O43747 |
| AP1S1 | Adaptor related protein complex 1 subunit sigma 1 | P61966 |
| AP1S2 | Adaptor related protein complex 1 subunit sigma 2 | P56377 |
| AP2A1 | Adaptor related protein complex 2 subunit alpha 1 | O95782 |
| AP2B1 | Adaptor related protein complex 2 subunit beta 1 | P63010 |
| AP2M1 | Adaptor related protein complex 2 subunit mu 1 | Q96CW1 |
| AP2S1 | Adaptor related protein complex 2 subunit sigma 1 | P53680 |
| AP3B1 | Adaptor related protein complex 3 subunit beta 1 | O00203 |
| AP3D1 | Adaptor related protein complex 3 subunit delta 1 | O14617 |
| AP3M1 | Adaptor related protein complex 3 subunit mu 1 | Q9Y2T2 |
| AP3M2 | Adaptor related protein complex 3 subunit mu 2 | P53677 |
| AP3S1 | Adaptor related protein complex 3 subunit sigma 1 | Q92572 |
| AP3S2 | Adaptor related protein complex 3 subunit sigma 2 | P59780 |
| AP4B1 | Adaptor related protein complex 4 subunit beta 1 | Q9Y6B7 |
| AP4M1 | Adaptor related protein complex 4 subunit mu 1 | O00189 |
| AP4S1 | Adaptor related protein complex 4 subunit sigma 1 | Q9Y587 |
| AP5M1 | Adaptor related protein complex 5 subunit mu 1 | Q9H0R1 |
| AP5S1 | Adaptor related protein complex 5 subunit sigma 1 | Q9NUS5 |
| AP5Z1 | Adaptor related protein complex 5 subunit zeta 1 | O43299 |
| APAF1 | Apoptotic peptidase activating factor 1 | O14727 |
| APBA2 | Amyloid beta precursor protein binding family A member 2 | Q99767 |
| APBA3 | Amyloid beta precursor protein binding family A member 3 | O96018 |
| APBB1 | Amyloid beta precursor protein binding family B member 1 | O00213 |
| APBB1IP | Amyloid beta precursor protein binding family B member 1 interacting protein | Q7Z5R6 |
| APC | APC regulator of WNT signaling pathway | P25054 |
| APEH | Acylaminoacyl-peptide hydrolase | P13798 |
| APEX1 | Apurinic/apyrimidinic endodeoxyribonuclease 1 | P27695 |
| APEX2 | Apurinic/apyrimidinic endodeoxyribonuclease 2 | Q9UBZ4 |
| APH1A | Aph-1 homolog A, gamma-secretase subunit | Q96BI3 |
| APH1B | Aph-1 homolog B, gamma-secretase subunit | Q8WW43 |
| API5 | Apoptosis inhibitor 5 | Q9BZZ5 |
| APIP | APAF1 interacting protein | Q96GX9 |
| APLP2 | Amyloid beta precursor like protein 2 | Q06481 |
| APMAP | Adipocyte plasma membrane associated protein | Q9HDC9 |
| APOBEC2 | Apolipoprotein B mRNA editing enzyme catalytic subunit 2 | Q9Y235 |
| APOBEC3C | Apolipoprotein B mRNA editing enzyme catalytic subunit 3C | Q9NRW3 |
| APOBEC3D | Apolipoprotein B mRNA editing enzyme catalytic subunit 3D | Q96AK3 |
| APOBEC3F | Apolipoprotein B mRNA editing enzyme catalytic subunit 3F | Q8IUX4 |
| APOBEC3G | Apolipoprotein B mRNA editing enzyme catalytic subunit 3G | Q9HC16 |
| APOBEC3H | Apolipoprotein B mRNA editing enzyme catalytic subunit 3H | Q6NTF7 |
| APOL1 | Apolipoprotein L1 | O14791 |
| APOL2 | Apolipoprotein L2 | Q9BQE5 |
| APOL3 | Apolipoprotein L3 | O95236 |
| APOL6 | Apolipoprotein L6 | Q9BWW8 |
| APOO | Apolipoprotein O | Q9BUR5 |
| APOOL | Apolipoprotein O like | Q6UXV4 |
| APPBP2 | Amyloid beta precursor protein binding protein 2 | Q92624 |
| APPL1 | Adaptor protein, phosphotyrosine interacting with PH domain and leucine zipper 1 | Q9UKG1 |
| APPL2 | Adaptor protein, phosphotyrosine interacting with PH domain and leucine zipper 2 | Q8NEU8 |
| APRT | Adenine phosphoribosyltransferase | P07741 |
| APTX | Aprataxin | Q7Z2E3 |
| ARAF | A-Raf proto-oncogene, serine/threonine kinase | P10398 |
| ARAP1 | ArfGAP with RhoGAP domain, ankyrin repeat and PH domain 1 | Q96P48 |
| ARAP2 | ArfGAP with RhoGAP domain, ankyrin repeat and PH domain 2 | Q8WZ64 |
| ARCN1 | Archain 1 | P48444 |
| AREL1 | Apoptosis resistant E3 ubiquitin protein ligase 1 | O15033 |
| ARF1 | ADP ribosylation factor 1 | P84077 |
| ARF3 | ADP ribosylation factor 3 | P61204 |
| ARF4 | ADP ribosylation factor 4 | P18085 |
| ARF5 | ADP ribosylation factor 5 | P84085 |
| ARF6 | ADP ribosylation factor 6 | P62330 |
| ARFGAP1 | ADP ribosylation factor GTPase activating protein 1 | Q8N6T3 |
| ARFGAP2 | ADP ribosylation factor GTPase activating protein 2 | Q8N6H7 |
| ARFGAP3 | ADP ribosylation factor GTPase activating protein 3 | Q9NP61 |
| ARFGEF1 | ADP ribosylation factor guanine nucleotide exchange factor 1 | Q9Y6D6 |
| ARFIP1 | ADP ribosylation factor interacting protein 1 | P53367 |
| ARFIP2 | ADP ribosylation factor interacting protein 2 | P53365 |
| ARFRP1 | ADP ribosylation factor related protein 1 | Q13795 |
| ARGLU1 | Arginine and glutamate rich 1 | Q9NWB6 |
| ARHGAP1 | Rho GTPase activating protein 1 | Q07960 |
| ARHGAP12 | Rho GTPase activating protein 12 | Q8IWW6 |
| ARHGAP15 | Rho GTPase activating protein 15 | Q53QZ3 |
| ARHGAP17 | Rho GTPase activating protein 17 | Q68EM7 |
| ARHGAP18 | Rho GTPase activating protein 18 | Q8N392 |
| ARHGAP19 | Rho GTPase activating protein 19 | Q14CB8 |
| ARHGAP25 | Rho GTPase activating protein 25 | P42331 |
| ARHGAP26 | Rho GTPase activating protein 26 | Q9UNA1 |
| ARHGAP4 | Rho GTPase activating protein 4 | P98171 |
| ARHGAP9 | Rho GTPase activating protein 9 | Q9BRR9 |
| ARHGDIA | Rho GDP dissociation inhibitor alpha | P52565 |
| ARHGDIB | Rho GDP dissociation inhibitor beta | P52566 |
| ARHGEF1 | Rho guanine nucleotide exchange factor 1 | Q92888 |
| ARHGEF2 | Rho/Rac guanine nucleotide exchange factor 2 | Q92974 |
| ARHGEF3 | Rho guanine nucleotide exchange factor 3 | Q9NR81 |
| ARHGEF4 | Rho guanine nucleotide exchange factor 4 | Q9NR80 |
| ARHGEF7 | Rho guanine nucleotide exchange factor 7 | Q14155 |
| ARHGEF9 | Cdc42 guanine nucleotide exchange factor 9 | O43307 |
| ARID2 | AT-rich interaction domain 2 | Q68CP9 |
| ARID3B | AT-rich interaction domain 3B | Q8IVW6 |
| ARID5A | AT-rich interaction domain 5A | Q03989 |
| ARID5B | AT-rich interaction domain 5B | Q14865 |
| ARIH2 | Ariadne RBR E3 ubiquitin protein ligase 2 | O95376 |
| ARL1 | ADP ribosylation factor like GTPase 1 | P40616 |
| ARL13B | ADP ribosylation factor like GTPase 13B | Q3SXY8 |
| ARL14EP | ADP ribosylation factor like GTPase 14 effector protein | Q8N8R7 |
| ARL15 | ADP ribosylation factor like GTPase 15 | Q9NXU5 |
| ARL16 | ADP ribosylation factor like GTPase 16 |  |
| ARL17A | ADP ribosylation factor like GTPase 17A | Q8IVW1 |
| ARL17B | ADP ribosylation factor like GTPase 17B | Q8IVW1 |
| ARL2 | ADP ribosylation factor like GTPase 2 | P36404 |
| ARL2BP | ADP ribosylation factor like GTPase 2 binding protein | Q9Y2Y0 |
| ARL3 | ADP ribosylation factor like GTPase 3 | P36405 |
| ARL4A | ADP ribosylation factor like GTPase 4A | P40617 |
| ARL4C | ADP ribosylation factor like GTPase 4C | P56559 |
| ARL4D | ADP ribosylation factor like GTPase 4D | P49703 |
| ARL5A | ADP ribosylation factor like GTPase 5A | Q9Y689 |
| ARL6 | ADP ribosylation factor like GTPase 6 | Q9H0F7 |
| ARL6IP1 | ADP ribosylation factor like GTPase 6 interacting protein 1 | Q15041 |
| ARL6IP4 | ADP ribosylation factor like GTPase 6 interacting protein 4 | Q66PJ3 |
| ARL6IP5 | ADP ribosylation factor like GTPase 6 interacting protein 5 | O75915 |
| ARL6IP6 | ADP ribosylation factor like GTPase 6 interacting protein 6 | Q8N6S5 |
| ARL8A | ADP ribosylation factor like GTPase 8A | Q96BM9 |
| ARL8B | ADP ribosylation factor like GTPase 8B | Q9NVJ2 |
| ARMC1 | Armadillo repeat containing 1 | Q9NVT9 |
| ARMC10 | Armadillo repeat containing 10 | Q8N2F6 |
| ARMC2 | Armadillo repeat containing 2 | Q8NEN0 |
| ARMC5 | Armadillo repeat containing 5 | Q96C12 |
| ARMC6 | Armadillo repeat containing 6 | Q6NXE6 |
| ARMC7 | Armadillo repeat containing 7 | Q9H6L4 |
| ARMC8 | Armadillo repeat containing 8 | Q8IUR7 |
| ARMCX1 | Armadillo repeat containing X-linked 1 | Q9P291 |
| ARMCX2 | Armadillo repeat containing X-linked 2 | Q7L311 |
| ARMCX3 | Armadillo repeat containing X-linked 3 | Q9UH62 |
| ARMCX5 | Armadillo repeat containing X-linked 5 | Q6P1M9 |
| ARMCX6 | Armadillo repeat containing X-linked 6 | Q7L4S7 |
| ARNT | Aryl hydrocarbon receptor nuclear translocator | P27540 |
| ARPC1A | Actin related protein 2/3 complex subunit 1A | Q92747 |
| ARPC1B | Actin related protein 2/3 complex subunit 1B | O15143 |
| ARPC3 | Actin related protein 2/3 complex subunit 3 | O15145 |
| ARPC4 | Actin related protein 2/3 complex subunit 4 | P59998 |
| ARPC5 | Actin related protein 2/3 complex subunit 5 | O15511 |
| ARPC5L | Actin related protein 2/3 complex subunit 5 like | Q9BPX5 |
| ARPP19 | CAMP regulated phosphoprotein 19 | P56211 |
| ARRB1 | Arrestin beta 1 | P49407 |
| ARRB2 | Arrestin beta 2 | P32121 |
| ARRDC1 | Arrestin domain containing 1 | Q8N5I2 |
| ARRDC2 | Arrestin domain containing 2 | Q8TBH0 |
| ARRDC3 | Arrestin domain containing 3 | Q96B67 |
| ARSA | Arylsulfatase A | P15289 |
| ARSD | Arylsulfatase D | P51689 |
| ARSG | Arylsulfatase G | Q96EG1 |
| ARSK | Arylsulfatase family member K | Q6UWY0 |
| ARV1 | ARV1 homolog, fatty acid homeostasis modulator | Q9H2C2 |
| ASAH1 | N-acylsphingosine amidohydrolase 1 | Q13510 |
| ASAH2B | N-acylsphingosine amidohydrolase 2B | P0C7U1 |
| ASB1 | Ankyrin repeat and SOCS box containing 1 | Q9Y576 |
| ASB13 | Ankyrin repeat and SOCS box containing 13 | Q8WXK3 |
| ASB16 | Ankyrin repeat and SOCS box containing 16 | Q96NS5 |
| ASB2 | Ankyrin repeat and SOCS box containing 2 | Q96Q27 |
| ASB3 | Ankyrin repeat and SOCS box containing 3 | Q9Y575 |
| ASB6 | Ankyrin repeat and SOCS box containing 6 | Q9NWX5 |
| ASB7 | Ankyrin repeat and SOCS box containing 7 | Q9H672 |
| ASB8 | Ankyrin repeat and SOCS box containing 8 | Q9H765 |
| ASCC1 | Activating signal cointegrator 1 complex subunit 1 | Q8N9N2 |
| ASCC2 | Activating signal cointegrator 1 complex subunit 2 | Q9H1I8 |
| ASCC3 | Activating signal cointegrator 1 complex subunit 3 | Q8N3C0 |
| ASCL2 | Achaete-scute family bHLH transcription factor 2 | Q99929 |
| ASF1A | Anti-silencing function 1A histone chaperone | Q9Y294 |
| ASF1B | Anti-silencing function 1B histone chaperone | Q9NVP2 |
| ASH2L | ASH2 like, histone lysine methyltransferase complex subunit | Q9UBL3 |
| ASL | Argininosuccinate lyase | P04424 |
| ASMTL | Acetylserotonin O-methyltransferase like | O95671 |
| ASNS | Asparagine synthetase (glutamine-hydrolyzing) | P08243 |
| ASNSD1 | Asparagine synthetase domain containing 1 | Q9NWL6 |
| ASPH | Aspartate beta-hydroxylase | Q12797 |
| ASPSCR1 | ASPSCR1 tether for SLC2A4, UBX domain containing | Q9BZE9 |
| ASRGL1 | Asparaginase and isoaspartyl peptidase 1 | Q7L266 |
| ASTE1 | Asteroid homolog 1 | Q2TB18 |
| ASXL1 | ASXL transcriptional regulator 1 | Q8IXJ9 |
| ASXL2 | ASXL transcriptional regulator 2 | Q76L83 |
| ATAD2 | ATPase family AAA domain containing 2 | Q6PL18 |
| ATAD3A | ATPase family AAA domain containing 3A | Q9NVI7 |
| ATE1 | Arginyltransferase 1 | O95260 |
| ATF1 | Activating transcription factor 1 | P18846 |
| ATF2 | Activating transcription factor 2 | P15336 |
| ATF4 | Activating transcription factor 4 | P18848 |
| ATF5 | Activating transcription factor 5 | Q9Y2D1 |
| ATF6 | Activating transcription factor 6 | P18850 |
| ATF6B | Activating transcription factor 6 beta | Q99941 |
| ATF7 | Activating transcription factor 7 | P17544 |
| ATF7IP2 | Activating transcription factor 7 interacting protein 2 | Q5U623 |
| ATG10 | Autophagy related 10 | Q9H0Y0 |
| ATG101 | Autophagy related 101 | Q9BSB4 |
| ATG12 | Autophagy related 12 | O94817 |
| ATG13 | Autophagy related 13 | O75143 |
| ATG14 | Autophagy related 14 | Q6ZNE5 |
| ATG16L1 | Autophagy related 16 like 1 | Q676U5 |
| ATG3 | Autophagy related 3 | Q9NT62 |
| ATG4A | Autophagy related 4A cysteine peptidase | Q8WYN0 |
| ATG4B | Autophagy related 4B cysteine peptidase | Q9Y4P1 |
| ATG4C | Autophagy related 4C cysteine peptidase | Q96DT6 |
| ATG4D | Autophagy related 4D cysteine peptidase | Q86TL0 |
| ATG5 | Autophagy related 5 | Q9H1Y0 |
| ATG7 | Autophagy related 7 | O95352 |
| ATG9A | Autophagy related 9A | Q7Z3C6 |
| ATIC | 5-aminoimidazole-4-carboxamide ribonucleotide formyltransferase/IMP cyclohydrolase | P31939 |
| ATL2 | Atlastin GTPase 2 | Q8NHH9 |
| ATL3 | Atlastin GTPase 3 | Q6DD88 |
| ATM | ATM serine/threonine kinase | Q13315 |
| ATMIN | ATM interactor | O43313 |
| ATOX1 | Antioxidant 1 copper chaperone | O00244 |
| ATP11B | ATPase phospholipid transporting 11B (putative) | Q9Y2G3 |
| ATP13A1 | ATPase 13A1 | Q9HD20 |
| ATP13A2 | ATPase cation transporting 13A2 | Q9NQ11 |
| ATP1A1 | ATPase Na+/K+ transporting subunit alpha 1 | P05023 |
| ATP1B1 | ATPase Na+/K+ transporting subunit beta 1 | P05026 |
| ATP1B3 | ATPase Na+/K+ transporting subunit beta 3 | P54709 |
| ATP2A3 | ATPase sarcoplasmic/endoplasmic reticulum Ca2+ transporting 3 | Q93084 |
| ATP2B4 | ATPase plasma membrane Ca2+ transporting 4 | P23634 |
| ATP2C1 | ATPase secretory pathway Ca2+ transporting 1 | P98194 |
| ATP6AP1 | ATPase H+ transporting accessory protein 1 | Q15904 |
| ATP6AP2 | ATPase H+ transporting accessory protein 2 | O75787 |
| ATP6V0A2 | ATPase H+ transporting V0 subunit a2 | Q9Y487 |
| ATP6V0B | ATPase H+ transporting V0 subunit b | Q99437 |
| ATP6V0C | ATPase H+ transporting V0 subunit c | P27449 |
| ATP6V0D1 | ATPase H+ transporting V0 subunit d1 | P61421 |
| ATP6V0E1 | ATPase H+ transporting V0 subunit e1 | O15342 |
| ATP6V0E2 | ATPase H+ transporting V0 subunit e2 | Q8NHE4 |
| ATP6V1B2 | ATPase H+ transporting V1 subunit B2 | P21281 |
| ATP6V1C1 | ATPase H+ transporting V1 subunit C1 | P21283 |
| ATP6V1D | ATPase H+ transporting V1 subunit D | Q9Y5K8 |
| ATP6V1E1 | ATPase H+ transporting V1 subunit E1 | P36543 |
| ATP6V1E2 | ATPase H+ transporting V1 subunit E2 | Q96A05 |
| ATP6V1F | ATPase H+ transporting V1 subunit F | Q16864 |
| ATP6V1G1 | ATPase H+ transporting V1 subunit G1 | O75348 |
| ATP6V1H | ATPase H+ transporting V1 subunit H | Q9UI12 |
| ATP9B | ATPase phospholipid transporting 9B (putative) | O43861 |
| ATPAF1 | ATP synthase mitochondrial F1 complex assembly factor 1 | Q5TC12 |
| ATPAF2 | ATP synthase mitochondrial F1 complex assembly factor 2 | Q8N5M1 |
| ATRAID | All-trans retinoic acid induced differentiation factor | Q6UW56 |
| ATRX | ATRX chromatin remodeler | P46100 |
| ATXN10 | Ataxin 10 | Q9UBB4 |
| ATXN3 | Ataxin 3 | P54252 |
| ATXN7 | Ataxin 7 | O15265 |
| ATXN7L1 | Ataxin 7 like 1 | Q9ULK2 |
| ATXN7L3 | Ataxin 7 like 3 | Q14CW9 |
| AUH | AU RNA binding methylglutaconyl-CoA hydratase | Q13825 |
| AUP1 | AUP1 lipid droplet regulating VLDL assembly factor | Q9Y679 |
| AURKA | Aurora kinase A | O14965 |
| AURKAIP1 | Aurora kinase A interacting protein 1 | Q9NWT8 |
| AURKB | Aurora kinase B | Q96GD4 |
| AUTS2 | Activator of transcription and developmental regulator AUTS2 | Q8WXX7 |
| AVL9 | AVL9 cell migration associated | Q8NBF6 |
| AVPI1 | Arginine vasopressin induced 1 | Q5T686 |
| AZI2 | 5-azacytidine induced 2 | Q9H6S1 |
| AZIN1 | Antizyme inhibitor 1 | O14977 |
| AZIN2 | Antizyme inhibitor 2 | Q96A70 |
| B2M | Beta-2-microglobulin | P61769 |
| B3GALT2 | Beta-1,3-galactosyltransferase 2 | O43825 |
| B3GALT4 | Beta-1,3-galactosyltransferase 4 | O96024 |
| B3GALT6 | Beta-1,3-galactosyltransferase 6 | Q96L58 |
| B3GAT3 | Beta-1,3-glucuronyltransferase 3 | O94766 |
| B3GLCT | Beta 3-glucosyltransferase | Q6Y288 |
| B3GNT2 | UDP-GlcNAc:betaGal beta-1,3-N-acetylglucosaminyltransferase 2 | Q9NY97 |
| B3GNT8 | UDP-GlcNAc:betaGal beta-1,3-N-acetylglucosaminyltransferase 8 | Q7Z7M8 |
| B4GALT1 | Beta-1,4-galactosyltransferase 1 | P15291 |
| B4GALT3 | Beta-1,4-galactosyltransferase 3 | O60512 |
| B4GALT4 | Beta-1,4-galactosyltransferase 4 | O60513 |
| B4GALT5 | Beta-1,4-galactosyltransferase 5 | O43286 |
| B4GALT7 | Beta-1,4-galactosyltransferase 7 | Q9UBV7 |
| B4GAT1 | Beta-1,4-glucuronyltransferase 1 | O43505 |
| B9D2 | B9 domain containing 2 | Q9BPU9 |
| BABAM1 | BRISC and BRCA1 A complex member 1 | Q9NWV8 |
| BACH1 | BTB domain and CNC homolog 1 | O14867 |
| BACH2 | BTB domain and CNC homolog 2 | Q9BYV9 |
| BAD | BCL2 associated agonist of cell death | Q92934 |
| BAG1 | BAG cochaperone 1 | Q99933 |
| BAG2 | BAG cochaperone 2 | O95816 |
| BAG3 | BAG cochaperone 3 | O95817 |
| BAG4 | BAG cochaperone 4 | O95429 |
| BAG5 | BAG cochaperone 5 | Q9UL15 |
| BAG6 | BAG cochaperone 6 | P46379 |
| BAHD1 | Bromo adjacent homology domain containing 1 | Q8TBE0 |
| BAIAP2 | BAR/IMD domain containing adaptor protein 2 | Q9UQB8 |
| BAK1 | BCL2 antagonist/killer 1 | Q16611 |
| BANF1 | BAF nuclear assembly factor 1 | O75531 |
| BANP | BTG3 associated nuclear protein | Q8N9N5 |
| BAP1 | BRCA1 associated protein 1 | Q92560 |
| BATF | Basic leucine zipper ATF-like transcription factor | Q16520 |
| BAX | BCL2 associated X, apoptosis regulator | Q07812 |
| BAZ2A | Bromodomain adjacent to zinc finger domain 2A | Q9UIF9 |
| BAZ2B | Bromodomain adjacent to zinc finger domain 2B | Q9UIF8 |
| BBS1 | Bardet-Biedl syndrome 1 | Q8NFJ9 |
| BBS10 | Bardet-Biedl syndrome 10 | Q8TAM1 |
| BBS12 | Bardet-Biedl syndrome 12 | Q6ZW61 |
| BBS2 | Bardet-Biedl syndrome 2 | Q9BXC9 |
| BBS4 | Bardet-Biedl syndrome 4 | Q96RK4 |
| BBS7 | Bardet-Biedl syndrome 7 | Q8IWZ6 |
| BBX | BBX high mobility group box domain containing | Q8WY36 |
| BCAP29 | B cell receptor associated protein 29 | Q9UHQ4 |
| BCAP31 | B cell receptor associated protein 31 | P51572 |
| BCAS2 | BCAS2 pre-mRNA processing factor | O75934 |
| BCAS3 | BCAS3 microtubule associated cell migration factor | Q9H6U6 |
| BCAS4 | Breast carcinoma amplified sequence 4 | Q8TDM0 |
| BCAT2 | Branched chain amino acid transaminase 2 | O15382 |
| BCCIP | BRCA2 and CDKN1A interacting protein | Q9P287 |
| BCDIN3D | BCDIN3 domain containing RNA methyltransferase | Q7Z5W3 |
| BCKDHA | Branched chain keto acid dehydrogenase E1 subunit alpha | P12694 |
| BCKDHB | Branched chain keto acid dehydrogenase E1 subunit beta | P21953 |
| BCKDK | Branched chain keto acid dehydrogenase kinase | O14874 |
| BCL10 | BCL10 immune signaling adaptor | O95999 |
| BCL11A | BAF chromatin remodeling complex subunit BCL11A | Q9H165 |
| BCL11B | BAF chromatin remodeling complex subunit BCL11B | Q9C0K0 |
| BCL2 | BCL2 apoptosis regulator | P10415 |
| BCL2A1 | BCL2 related protein A1 | Q16548 |
| BCL2L1 | BCL2 like 1 | Q07817 |
| BCL2L11 | BCL2 like 11 | O43521 |
| BCL2L12 | BCL2 like 12 | Q9HB09 |
| BCL2L13 | BCL2 like 13 | Q9BXK5 |
| BCL3 | BCL3 transcription coactivator | P20749 |
| BCL6 | BCL6 transcription repressor | P41182 |
| BCL7B | BAF chromatin remodeling complex subunit BCL7B | Q9BQE9 |
| BCL7C | BAF chromatin remodeling complex subunit BCL7C | Q8WUZ0 |
| BCLAF1 | BCL2 associated transcription factor 1 | Q9NYF8 |
| BCOR | BCL6 corepressor | Q6W2J9 |
| BCS1L | BCS1 homolog, ubiquinol-cytochrome c reductase complex chaperone | Q9Y276 |
| BDH1 | 3-hydroxybutyrate dehydrogenase 1 | Q02338 |
| BDH2 | 3-hydroxybutyrate dehydrogenase 2 | Q9BUT1 |
| BECN1 | Beclin 1 | Q14457 |
| BEND5 | BEN domain containing 5 | Q7L4P6 |
| BET1 | Bet1 golgi vesicular membrane trafficking protein | O15155 |
| BET1L | Bet1 golgi vesicular membrane trafficking protein like | Q9NYM9 |
| BEX2 | Brain expressed X-linked 2 | Q9BXY8 |
| BEX4 | Brain expressed X-linked 4 | Q9NWD9 |
| BEX5 | Brain expressed X-linked 5 | Q5H9J7 |
| BFAR | Bifunctional apoptosis regulator | Q9NZS9 |
| BHLHE40 | Basic helix-loop-helix family member e40 | O14503 |
| BICD2 | BICD cargo adaptor 2 | Q8TD16 |
| BID | BH3 interacting domain death agonist | P55957 |
| BIK | BCL2 interacting killer | Q13323 |
| BIN1 | Bridging integrator 1 | O00499 |
| BIN2 | Bridging integrator 2 | Q9UBW5 |
| BIN3 | Bridging integrator 3 | Q9NQY0 |
| BIRC2 | Baculoviral IAP repeat containing 2 | Q13490 |
| BIRC3 | Baculoviral IAP repeat containing 3 | Q13489 |
| BIRC5 | Baculoviral IAP repeat containing 5 | O15392 |
| BIVM | Basic, immunoglobulin-like variable motif containing | Q86UB2 |
| BLCAP | BLCAP apoptosis inducing factor | P62952 |
| BLK | BLK proto-oncogene, Src family tyrosine kinase | P51451 |
| BLMH | Bleomycin hydrolase | Q13867 |
| BLOC1S1 | Biogenesis of lysosomal organelles complex 1 subunit 1 | P78537 |
| BLOC1S2 | Biogenesis of lysosomal organelles complex 1 subunit 2 | Q6QNY1 |
| BLOC1S4 | Biogenesis of lysosomal organelles complex 1 subunit 4 | Q9NUP1 |
| BLOC1S5 | Biogenesis of lysosomal organelles complex 1 subunit 5 | Q8TDH9 |
| BLOC1S6 | Biogenesis of lysosomal organelles complex 1 subunit 6 | Q9UL45 |
| BLVRA | Biliverdin reductase A | P53004 |
| BLVRB | Biliverdin reductase B | P30043 |
| BLZF1 | Basic leucine zipper nuclear factor 1 | Q9H2G9 |
| BMI1 | BMI1 proto-oncogene, polycomb ring finger | P35226 |
| BNIP1 | BCL2 interacting protein 1 | Q12981 |
| BNIP2 | BCL2 interacting protein 2 | Q12982 |
| BNIP3 | BCL2 interacting protein 3 | Q12983 |
| BNIP3L | BCL2 interacting protein 3 like | O60238 |
| BOD1 | Biorientation of chromosomes in cell division 1 | Q96IK1 |
| BOK | BCL2 family apoptosis regulator BOK | Q9UMX3 |
| BOLA1 | BolA family member 1 | Q9Y3E2 |
| BOLA2 | BolA family member 2 | Q9H3K6 |
| BOLA3 | BolA family member 3 | Q53S33 |
| BOP1 | BOP1 ribosomal biogenesis factor | Q14137 |
| BORA | BORA aurora kinase A activator | Q6PGQ7 |
| BPGM | Bisphosphoglycerate mutase | P07738 |
| BPHL | Biphenyl hydrolase like | Q86WA6 |
| BPNT1 | 3'(2'), 5'-bisphosphate nucleotidase 1 | O95861 |
| BRAF | B-Raf proto-oncogene, serine/threonine kinase | P15056 |
| BRAT1 | BRCA1 associated ATM activator 1 | Q6PJG6 |
| BRCA1 | BRCA1 DNA repair associated | P38398 |
| BRCC3 | BRCA1/BRCA2-containing complex subunit 3 | P46736 |
| BRD1 | Bromodomain containing 1 | O95696 |
| BRD2 | Bromodomain containing 2 | P25440 |
| BRD3 | Bromodomain containing 3 | Q15059 |
| BRD4 | Bromodomain containing 4 | O60885 |
| BRD7 | Bromodomain containing 7 | Q9NPI1 |
| BRD9 | Bromodomain containing 9 | Q9H8M2 |
| BRF1 | BRF1 RNA polymerase III transcription initiation factor subunit | Q92994 |
| BRF2 | BRF2 RNA polymerase III transcription initiation factor subunit | Q9HAW0 |
| BRI3 | Brain protein I3 | O95415 |
| BRIX1 | Biogenesis of ribosomes BRX1 | Q8TDN6 |
| BRK1 | BRICK1 subunit of SCAR/WAVE actin nucleating complex | Q8WUW1 |
| BRMS1 | BRMS1 transcriptional repressor and anoikis regulator | Q9HCU9 |
| BRMS1L | BRMS1 like transcriptional repressor | Q5PSV4 |
| BROX | BRO1 domain and CAAX motif containing | Q5VW32 |
| BRPF1 | Bromodomain and PHD finger containing 1 | P55201 |
| BRWD1 | Bromodomain and WD repeat domain containing 1 | Q9NSI6 |
| BSCL2 | BSCL2 lipid droplet biogenesis associated, seipin | Q96G97 |
| BSDC1 | BSD domain containing 1 | Q9NW68 |
| BSG | Basigin (Ok blood group) | P35613 |
| BST2 | Bone marrow stromal cell antigen 2 | Q10589 |
| BTBD1 | BTB domain containing 1 | Q9H0C5 |
| BTBD10 | BTB domain containing 10 | Q9BSF8 |
| BTBD2 | BTB domain containing 2 | Q9BX70 |
| BTBD9 | BTB domain containing 9 | Q96Q07 |
| BTD | Biotinidase | P43251 |
| BTF3 | Basic transcription factor 3 | P20290 |
| BTF3L4 | Basic transcription factor 3 like 4 | Q96K17 |
| BTG1 | BTG anti-proliferation factor 1 | P62324 |
| BTG2 | BTG anti-proliferation factor 2 | P78543 |
| BTG3 | BTG anti-proliferation factor 3 | Q14201 |
| BTLA | B and T lymphocyte associated | Q7Z6A9 |
| BTN2A1 | Butyrophilin subfamily 2 member A1 | Q7KYR7 |
| BTN2A2 | Butyrophilin subfamily 2 member A2 | Q8WVV5 |
| BTN3A1 | Butyrophilin subfamily 3 member A1 | O00481 |
| BTN3A2 | Butyrophilin subfamily 3 member A2 | P78410 |
| BTN3A3 | Butyrophilin subfamily 3 member A3 | O00478 |
| BTRC | Beta-transducin repeat containing E3 ubiquitin protein ligase | Q9Y297 |
| BUB3 | BUB3 mitotic checkpoint protein | O43684 |
| BUD13 | BUD13 homolog | Q9BRD0 |
| BUD31 | BUD31 homolog | P41223 |
| BYSL | Bystin like | Q13895 |
| BZW1 | Basic leucine zipper and W2 domains 1 | Q7L1Q6 |
| BZW2 | Basic leucine zipper and W2 domains 2 | Q9Y6E2 |
| C10orf88 | Chromosome 10 open reading frame 88 | Q9H8K7 |
| C11orf1 | Chromosome 11 open reading frame 1 | Q9H5F2 |
| C11orf54 | Chromosome 11 open reading frame 54 | Q9H0W9 |
| C11orf68 | Chromosome 11 open reading frame 68 | Q9H3H3 |
| C11orf71 | Chromosome 11 open reading frame 71 | Q6IPW1 |
| C11orf98 | Chromosome 11 open reading frame 98 | E9PRG8 |
| C12orf4 | Chromosome 12 open reading frame 4 | Q9NQ89 |
| C12orf42 | Chromosome 12 open reading frame 42 | Q96LP6 |
| C12orf43 | Chromosome 12 open reading frame 43 | Q96C57 |
| C12orf57 | Chromosome 12 open reading frame 57 | Q99622 |
| C12orf60 | Chromosome 12 open reading frame 60 | Q5U649 |
| C14orf119 | Chromosome 14 open reading frame 119 | Q9NWQ9 |
| C14orf28 | Chromosome 14 open reading frame 28 | Q4W4Y0 |
| C14orf93 | Chromosome 14 open reading frame 93 | Q9H972 |
| C16orf54 | Chromosome 16 open reading frame 54 | Q6UWD8 |
| C16orf74 | Chromosome 16 open reading frame 74 | Q96GX8 |
| C16orf87 | Chromosome 16 open reading frame 87 | Q6PH81 |
| C17orf50 | Chromosome 17 open reading frame 50 | Q8WW18 |
| C17orf58 | Chromosome 17 open reading frame 58 | Q2M2W7 |
| C17orf75 | Chromosome 17 open reading frame 75 | Q9HAS0 |
| C17orf80 | Chromosome 17 open reading frame 80 | Q9BSJ5 |
| C18orf21 | Chromosome 18 open reading frame 21 | Q32NC0 |
| C18orf25 | Chromosome 18 open reading frame 25 | Q96B23 |
| C18orf32 | Chromosome 18 open reading frame 32 | Q8TCD1 |
| C19orf12 | Chromosome 19 open reading frame 12 | Q9NSK7 |
| C19orf25 | Chromosome 19 open reading frame 25 | Q9UFG5 |
| C19orf47 | Chromosome 19 open reading frame 47 | Q8N9M1 |
| C19orf53 | Chromosome 19 open reading frame 53 | Q9UNZ5 |
| C19orf54 | Chromosome 19 open reading frame 54 | Q5BKX5 |
| C19orf73 | Chromosome 19 open reading frame 73 | Q9NVV2 |
| C1D | C1D nuclear receptor corepressor | Q13901 |
| C1GALT1C1 | C1GALT1 specific chaperone 1 | Q96EU7 |
| C1orf109 | Chromosome 1 open reading frame 109 | Q9NX04 |
| C1orf131 | Chromosome 1 open reading frame 131 | Q8NDD1 |
| C1orf159 | Chromosome 1 open reading frame 159 | Q96HA4 |
| C1orf162 | Chromosome 1 open reading frame 162 | Q8NEQ5 |
| C1orf174 | Chromosome 1 open reading frame 174 | Q8IYL3 |
| C1orf198 | Chromosome 1 open reading frame 198 | Q9H425 |
| C1orf21 | Chromosome 1 open reading frame 21 | Q9H246 |
| C1orf43 | Chromosome 1 open reading frame 43 | Q9BWL3 |
| C1orf54 | Chromosome 1 open reading frame 54 | Q8WWF1 |
| C1orf74 | Chromosome 1 open reading frame 74 | Q96LT6 |
| C1QBP | Complement C1q binding protein | Q07021 |
| C20orf27 | Chromosome 20 open reading frame 27 | Q9GZN8 |
| C20orf96 | Chromosome 20 open reading frame 96 | Q9NUD7 |
| C22orf39 | Chromosome 22 open reading frame 39 | Q6P5X5 |
| C2orf15 | Chromosome 2 open reading frame 15 | Q8WU43 |
| C2orf42 | Chromosome 2 open reading frame 42 | Q9NWW7 |
| C2orf49 | Chromosome 2 open reading frame 49 | Q9BVC5 |
| C2orf68 | Chromosome 2 open reading frame 68 | Q2NKX9 |
| C2orf74 | Chromosome 2 open reading frame 74 | A8MZ97 |
| C2orf76 | Chromosome 2 open reading frame 76 | Q3KRA6 |
| C3AR1 | Complement C3a receptor 1 | Q16581 |
| C3orf14 | Chromosome 3 open reading frame 14 | Q9HBI5 |
| C3orf18 | Chromosome 3 open reading frame 18 | Q9UK00 |
| C3orf33 | Chromosome 3 open reading frame 33 | Q6P1S2 |
| C3orf38 | Chromosome 3 open reading frame 38 | Q5JPI3 |
| C3orf62 | Chromosome 3 open reading frame 62 | Q6ZUJ4 |
| C4orf33 | Chromosome 4 open reading frame 33 | Q8N1A6 |
| C4orf46 | Chromosome 4 open reading frame 46 | Q504U0 |
| C5orf22 | Chromosome 5 open reading frame 22 | Q49AR2 |
| C5orf24 | Chromosome 5 open reading frame 24 | Q7Z6I8 |
| C5orf34 | Chromosome 5 open reading frame 34 | Q96MH7 |
| C6orf226 | Chromosome 6 open reading frame 226 | Q5I0X4 |
| C6orf62 | Chromosome 6 open reading frame 62 | Q9GZU0 |
| C6orf89 | Chromosome 6 open reading frame 89 | Q6UWU4 |
| C7orf25 | Chromosome 7 open reading frame 25 | Q9BPX7 |
| C7orf50 | Chromosome 7 open reading frame 50 | Q9BRJ6 |
| C8orf33 | Chromosome 8 open reading frame 33 | Q9H7E9 |
| C8orf76 | Chromosome 8 open reading frame 76 | Q96K31 |
| C9orf72 | C9orf72-SMCR8 complex subunit | Q96LT7 |
| C9orf78 | Chromosome 9 open reading frame 78 | Q9NZ63 |
| C9orf85 | Chromosome 9 open reading frame 85 | Q96MD7 |
| CA11 | Carbonic anhydrase 11 | O75493 |
| CA5B | Carbonic anhydrase 5B | Q9Y2D0 |
| CA6 | Carbonic anhydrase 6 | P23280 |
| CAAP1 | Caspase activity and apoptosis inhibitor 1 | Q9H8G2 |
| CAB39 | Calcium binding protein 39 | Q9Y376 |
| CAB39L | Calcium binding protein 39 like | Q9H9S4 |
| CACNA2D4 | Calcium voltage-gated channel auxiliary subunit alpha2delta 4 | Q7Z3S7 |
| CACUL1 | CDK2 associated cullin domain 1 | Q86Y37 |
| CACYBP | Calcyclin binding protein | Q9HB71 |
| CADM1 | Cell adhesion molecule 1 | Q9BY67 |
| CALCOCO1 | Calcium binding and coiled-coil domain 1 | Q9P1Z2 |
| CALCOCO2 | Calcium binding and coiled-coil domain 2 | Q13137 |
| CALM1 | Calmodulin 1 | P0DP23 |
| CALR | Calreticulin | P27797 |
| CALU | Calumenin | O43852 |
| CAMK1D | Calcium/calmodulin dependent protein kinase ID | Q8IU85 |
| CAMK2D | Calcium/calmodulin dependent protein kinase II delta | Q13557 |
| CAMK2G | Calcium/calmodulin dependent protein kinase II gamma | Q13555 |
| CAMK4 | Calcium/calmodulin dependent protein kinase IV | Q16566 |
| CAMKK2 | Calcium/calmodulin dependent protein kinase kinase 2 | Q96RR4 |
| CAMKMT | Calmodulin-lysine N-methyltransferase | Q7Z624 |
| CAMLG | Calcium modulating ligand | P49069 |
| CAMTA1 | Calmodulin binding transcription activator 1 | Q9Y6Y1 |
| CAMTA2 | Calmodulin binding transcription activator 2 | O94983 |
| CAND1 | Cullin associated and neddylation dissociated 1 | Q86VP6 |
| CANT1 | Calcium activated nucleotidase 1 | Q8WVQ1 |
| CANX | Calnexin | P27824 |
| CAP1 | Cyclase associated actin cytoskeleton regulatory protein 1 | Q01518 |
| CAPG | Capping actin protein, gelsolin like | P40121 |
| CAPN1 | Calpain 1 | P07384 |
| CAPN10 | Calpain 10 | Q9HC96 |
| CAPN12 | Calpain 12 | Q6ZSI9 |
| CAPN2 | Calpain 2 | P17655 |
| CAPN7 | Calpain 7 | Q9Y6W3 |
| CAPNS1 | Calpain small subunit 1 | P04632 |
| CAPRIN1 | Cell cycle associated protein 1 | Q14444 |
| CAPS | Calcyphosine | Q13938 |
| CAPZA1 | Capping actin protein of muscle Z-line subunit alpha 1 | P52907 |
| CAPZA2 | Capping actin protein of muscle Z-line subunit alpha 2 | P47755 |
| CARD16 | Caspase recruitment domain family member 16 | Q5EG05 |
| CARD19 | Caspase recruitment domain family member 19 | Q96LW7 |
| CARD8 | Caspase recruitment domain family member 8 | Q9Y2G2 |
| CARF | Calcium responsive transcription factor | Q8N187 |
| CARHSP1 | Calcium regulated heat stable protein 1 | Q9Y2V2 |
| CARNMT1 | Carnosine N-methyltransferase 1 | Q8N4J0 |
| CARNS1 | Carnosine synthase 1 | A5YM72 |
| CASK | Calcium/calmodulin dependent serine protein kinase | O14936 |
| CASP1 | Caspase 1 | P29466 |
| CASP10 | Caspase 10 | Q92851 |
| CASP2 | Caspase 2 | P42575 |
| CASP3 | Caspase 3 | P42574 |
| CASP4 | Caspase 4 | P49662 |
| CASP6 | Caspase 6 | P55212 |
| CASP7 | Caspase 7 | P55210 |
| CASP8 | Caspase 8 | Q14790 |
| CASP8AP2 | Caspase 8 associated protein 2 |  |
| CASP9 | Caspase 9 | P55211 |
| CASS4 | Cas scaffold protein family member 4 | Q9NQ75 |
| CAST | Calpastatin | P20810 |
| CAT | Catalase | P04040 |
| CBFA2T2 | CBFA2/RUNX1 partner transcriptional co-repressor 2 | O43439 |
| CBFB | Core-binding factor subunit beta | Q13951 |
| CBL | Cbl proto-oncogene | P22681 |
| CBLB | Cbl proto-oncogene B | Q13191 |
| CBLL1 | Cbl proto-oncogene like 1 | Q75N03 |
| CBR1 | Carbonyl reductase 1 | P16152 |
| CBR3 | Carbonyl reductase 3 | O75828 |
| CBR4 | Carbonyl reductase 4 | Q8N4T8 |
| CBX1 | Chromobox 1 | P83916 |
| CBX3 | Chromobox 3 | Q13185 |
| CBX4 | Chromobox 4 | O00257 |
| CBX5 | Chromobox 5 | P45973 |
| CBX6 | Chromobox 6 | O95503 |
| CBX7 | Chromobox 7 | O95931 |
| CBX8 | Chromobox 8 | Q9HC52 |
| CBY1 | Chibby family member 1, beta catenin antagonist | Q9Y3M2 |
| CCAR2 | Cell cycle and apoptosis regulator 2 | Q8N163 |
| CCDC102A | Coiled-coil domain containing 102A | Q96A19 |
| CCDC106 | Coiled-coil domain containing 106 | Q9BWC9 |
| CCDC107 | Coiled-coil domain containing 107 | Q8WV48 |
| CCDC112 | Coiled-coil domain containing 112 | Q8NEF3 |
| CCDC115 | Coiled-coil domain containing 115 | Q96NT0 |
| CCDC117 | Coiled-coil domain containing 117 | Q8IWD4 |
| CCDC12 | Coiled-coil domain containing 12 | Q8WUD4 |
| CCDC121 | Coiled-coil domain containing 121 | Q6ZUS5 |
| CCDC124 | Coiled-coil domain containing 124 | Q96CT7 |
| CCDC126 | Coiled-coil domain containing 126 | Q96EE4 |
| CCDC127 | Coiled-coil domain containing 127 | Q96BQ5 |
| CCDC134 | Coiled-coil domain containing 134 | Q9H6E4 |
| CCDC137 | Coiled-coil domain containing 137 | Q6PK04 |
| CCDC138 | Coiled-coil domain containing 138 | Q96M89 |
| CCDC14 | Coiled-coil domain containing 14 | Q49A88 |
| CCDC141 | Coiled-coil domain containing 141 | Q6ZP82 |
| CCDC142 | Coiled-coil domain containing 142 | Q17RM4 |
| CCDC146 | Coiled-coil domain containing 146 | Q8IYE0 |
| CCDC167 | Coiled-coil domain containing 167 | Q9P0B6 |
| CCDC18 | Coiled-coil domain containing 18 | Q5T9S5 |
| CCDC186 | Coiled-coil domain containing 186 | Q7Z3E2 |
| CCDC191 | Coiled-coil domain containing 191 | Q8NCU4 |
| CCDC22 | Coiled-coil domain containing 22 | O60826 |
| CCDC25 | Coiled-coil domain containing 25 | Q86WR0 |
| CCDC28A | Coiled-coil domain containing 28A |  |
| CCDC28B | Coiled-coil domain containing 28B | Q9BUN5 |
| CCDC34 | Coiled-coil domain containing 34 | Q96HJ3 |
| CCDC43 | Coiled-coil domain containing 43 | Q96MW1 |
| CCDC47 | Coiled-coil domain containing 47 | Q96A33 |
| CCDC50 | Coiled-coil domain containing 50 | Q8IVM0 |
| CCDC51 | Coiled-coil domain containing 51 | Q96ER9 |
| CCDC57 | Coiled-coil domain containing 57 | Q2TAC2 |
| CCDC59 | Coiled-coil domain containing 59 | Q9P031 |
| CCDC6 | Coiled-coil domain containing 6 | Q16204 |
| CCDC65 | Coiled-coil domain containing 65 | Q8IXS2 |
| CCDC66 | Coiled-coil domain containing 66 | A2RUB6 |
| CCDC69 | Coiled-coil domain containing 69 | A6NI79 |
| CCDC7 | Coiled-coil domain containing 7 | Q96M83 |
| CCDC71 | Coiled-coil domain containing 71 | Q8IV32 |
| CCDC77 | Coiled-coil domain containing 77 | Q9BR77 |
| CCDC82 | Coiled-coil domain containing 82 | Q8N4S0 |
| CCDC85B | Coiled-coil domain containing 85B | Q15834 |
| CCDC86 | Coiled-coil domain containing 86 | Q9H6F5 |
| CCDC88C | Coiled-coil domain containing 88C | Q9P219 |
| CCDC9 | Coiled-coil domain containing 9 | Q9Y3X0 |
| CCDC90B | Coiled-coil domain containing 90B | Q9GZT6 |
| CCDC91 | Coiled-coil domain containing 91 | Q7Z6B0 |
| CCDC92 | Coiled-coil domain containing 92 | Q53HC0 |
| CCDC93 | Coiled-coil domain containing 93 | Q567U6 |
| CCDC97 | Coiled-coil domain containing 97 | Q96F63 |
| CCL28 | C-C motif chemokine ligand 28 | Q9NRJ3 |
| CCL3 | C-C motif chemokine ligand 3 | P10147 |
| CCL4 | C-C motif chemokine ligand 4 | P13236 |
| CCL5 | C-C motif chemokine ligand 5 | P13501 |
| CCM2 | CCM2 scaffold protein | Q9BSQ5 |
| CCNB1 | Cyclin B1 | P14635 |
| CCNB1IP1 | Cyclin B1 interacting protein 1 | Q9NPC3 |
| CCNB2 | Cyclin B2 | O95067 |
| CCNC | Cyclin C | P24863 |
| CCND2 | Cyclin D2 | P30279 |
| CCND3 | Cyclin D3 | P30281 |
| CCNDBP1 | Cyclin D1 binding protein 1 | O95273 |
| CCNE2 | Cyclin E2 | O96020 |
| CCNG1 | Cyclin G1 | P51959 |
| CCNG2 | Cyclin G2 | Q16589 |
| CCNH | Cyclin H | P51946 |
| CCNI | Cyclin I | Q14094 |
| CCNJ | Cyclin J | Q5T5M9 |
| CCNK | Cyclin K | O75909 |
| CCNL1 | Cyclin L1 | Q9UK58 |
| CCNL2 | Cyclin L2 | Q96S94 |
| CCNT1 | Cyclin T1 | O60563 |
| CCNY | Cyclin Y | Q8ND76 |
| CCP110 | Centriolar coiled-coil protein 110 | O43303 |
| CCR1 | C-C motif chemokine receptor 1 | P32246 |
| CCR10 | C-C motif chemokine receptor 10 | P46092 |
| CCR2 | C-C motif chemokine receptor 2 | P41597 |
| CCR3 | C-C motif chemokine receptor 3 | P51677 |
| CCR4 | C-C motif chemokine receptor 4 | P51679 |
| CCR5 | C-C motif chemokine receptor 5 | P51681 |
| CCR6 | C-C motif chemokine receptor 6 | P51684 |
| CCR7 | C-C motif chemokine receptor 7 | P32248 |
| CCR8 | C-C motif chemokine receptor 8 | P51685 |
| CCR9 | C-C motif chemokine receptor 9 | P51686 |
| CCRL2 | C-C motif chemokine receptor like 2 | O00421 |
| CCS | Copper chaperone for superoxide dismutase | O14618 |
| CCSER2 | Coiled-coil serine rich protein 2 | Q9H7U1 |
| CCT2 | Chaperonin containing TCP1 subunit 2 | P78371 |
| CCT3 | Chaperonin containing TCP1 subunit 3 | P49368 |
| CCT4 | Chaperonin containing TCP1 subunit 4 | P50991 |
| CCT5 | Chaperonin containing TCP1 subunit 5 | P48643 |
| CCT6A | Chaperonin containing TCP1 subunit 6A | P40227 |
| CCT6B | Chaperonin containing TCP1 subunit 6B | Q92526 |
| CCT7 | Chaperonin containing TCP1 subunit 7 | Q99832 |
| CCT8 | Chaperonin containing TCP1 subunit 8 | P50990 |
| CCZ1B | CCZ1 homolog B, vacuolar protein trafficking and biogenesis associated | P86790 |
| CD14 | CD14 molecule | P08571 |
| CD151 | CD151 molecule (Raph blood group) | P48509 |
| CD160 | CD160 molecule | O95971 |
| CD164 | CD164 molecule | Q04900 |
| CD2 | CD2 molecule | P06729 |
| CD200 | CD200 molecule | P41217 |
| CD200R1 | CD200 receptor 1 | Q8TD46 |
| CD226 | CD226 molecule | Q15762 |
| CD244 | CD244 molecule | Q9BZW8 |
| CD247 | CD247 molecule | P20963 |
| CD248 | CD248 molecule | Q9HCU0 |
| CD27 | CD27 molecule | P26842 |
| CD274 | CD274 molecule | Q9NZQ7 |
| CD28 | CD28 molecule | P10747 |
| CD2BP2 | CD2 cytoplasmic tail binding protein 2 | O95400 |
| CD300A | CD300a molecule | Q9UGN4 |
| CD320 | CD320 molecule | Q9NPF0 |
| CD37 | CD37 molecule | P11049 |
| CD38 | CD38 molecule | P28907 |
| CD3D | CD3 delta subunit of T-cell receptor complex | P04234 |
| CD3E | CD3 epsilon subunit of T-cell receptor complex | P07766 |
| CD40 | CD40 molecule | P25942 |
| CD40LG | CD40 ligand | P29965 |
| CD44 | CD44 molecule (Indian blood group) | P16070 |
| CD46 | CD46 molecule | P15529 |
| CD47 | CD47 molecule | Q08722 |
| CD48 | CD48 molecule | P09326 |
| CD5 | CD5 molecule | P06127 |
| CD52 | CD52 molecule | P31358 |
| CD53 | CD53 molecule | P19397 |
| CD55 | CD55 molecule (Cromer blood group) | P08174 |
| CD58 | CD58 molecule | P19256 |
| CD59 | CD59 molecule (CD59 blood group) | P13987 |
| CD6 | CD6 molecule | P30203 |
| CD63 | CD63 molecule | P08962 |
| CD68 | CD68 molecule | P34810 |
| CD69 | CD69 molecule | Q07108 |
| CD7 | CD7 molecule | P09564 |
| CD70 | CD70 molecule | P32970 |
| CD72 | CD72 molecule | P21854 |
| CD74 | CD74 molecule | P04233 |
| CD79A | CD79a molecule | P11912 |
| CD79B | CD79b molecule | P40259 |
| CD81 | CD81 molecule | P60033 |
| CD82 | CD82 molecule | P27701 |
| CD83 | CD83 molecule | Q01151 |
| CD84 | CD84 molecule | Q9UIB8 |
| CD8A | CD8a molecule | P01732 |
| CD8B | CD8b molecule | P10966 |
| CD9 | CD9 molecule | P21926 |
| CD96 | CD96 molecule | P40200 |
| CD99 | CD99 molecule (Xg blood group) | P14209 |
| CD99L2 | CD99 molecule like 2 | Q8TCZ2 |
| CDADC1 | Cytidine and dCMP deaminase domain containing 1 | Q9BWV3 |
| CDC123 | Cell division cycle 123 | O75794 |
| CDC14A | Cell division cycle 14A | Q9UNH5 |
| CDC14B | Cell division cycle 14B | O60729 |
| CDC16 | Cell division cycle 16 | Q13042 |
| CDC20 | Cell division cycle 20 | Q12834 |
| CDC23 | Cell division cycle 23 | Q9UJX2 |
| CDC25B | Cell division cycle 25B | P30305 |
| CDC26 | Cell division cycle 26 | Q8NHZ8 |
| CDC27 | Cell division cycle 27 | P30260 |
| CDC34 | Cell division cycle 34, ubiqiutin conjugating enzyme | P49427 |
| CDC37 | Cell division cycle 37, HSP90 cochaperone | Q16543 |
| CDC37L1 | Cell division cycle 37 like 1, HSP90 cochaperone | Q7L3B6 |
| CDC40 | Cell division cycle 40 | O60508 |
| CDC42 | Cell division cycle 42 | P60953 |
| CDC42EP3 | CDC42 effector protein 3 | Q9UKI2 |
| CDC42SE1 | CDC42 small effector 1 | Q9NRR8 |
| CDC42SE2 | CDC42 small effector 2 | Q9NRR3 |
| CDC5L | Cell division cycle 5 like | Q99459 |
| CDC6 | Cell division cycle 6 | Q99741 |
| CDC7 | Cell division cycle 7 | O00311 |
| CDC73 | Cell division cycle 73 | Q6P1J9 |
| CDCA4 | Cell division cycle associated 4 | Q9BXL8 |
| CDCA7 | Cell division cycle associated 7 | Q9BWT1 |
| CDCA7L | Cell division cycle associated 7 like | Q96GN5 |
| CDCA8 | Cell division cycle associated 8 | Q53HL2 |
| CDIP1 | Cell death inducing p53 target 1 | Q9H305 |
| CDIPT | CDP-diacylglycerol--inositol 3-phosphatidyltransferase | O14735 |
| CDK1 | Cyclin dependent kinase 1 | P06493 |
| CDK10 | Cyclin dependent kinase 10 | Q15131 |
| CDK11A | Cyclin dependent kinase 11A | Q9UQ88 |
| CDK11B | Cyclin dependent kinase 11B | P21127 |
| CDK12 | Cyclin dependent kinase 12 | Q9NYV4 |
| CDK13 | Cyclin dependent kinase 13 | Q14004 |
| CDK16 | Cyclin dependent kinase 16 | Q00536 |
| CDK17 | Cyclin dependent kinase 17 | Q00537 |
| CDK20 | Cyclin dependent kinase 20 | Q8IZL9 |
| CDK2AP2 | Cyclin dependent kinase 2 associated protein 2 | O75956 |
| CDK4 | Cyclin dependent kinase 4 | P11802 |
| CDK5 | Cyclin dependent kinase 5 | Q00535 |
| CDK5RAP1 | CDK5 regulatory subunit associated protein 1 | Q96SZ6 |
| CDK5RAP3 | CDK5 regulatory subunit associated protein 3 | Q96JB5 |
| CDK7 | Cyclin dependent kinase 7 | P50613 |
| CDK9 | Cyclin dependent kinase 9 | P50750 |
| CDKAL1 | CDK5 regulatory subunit associated protein 1 like 1 | Q5VV42 |
| CDKL1 | Cyclin dependent kinase like 1 | Q00532 |
| CDKL3 | Cyclin dependent kinase like 3 | Q8IVW4 |
| CDKN1B | Cyclin dependent kinase inhibitor 1B | P46527 |
| CDKN2A | Cyclin dependent kinase inhibitor 2A | P42771, Q8N726 |
| CDKN2AIP | CDKN2A interacting protein | Q9NXV6 |
| CDKN2AIPNL | CDKN2A interacting protein N-terminal like | Q96HQ2 |
| CDKN2C | Cyclin dependent kinase inhibitor 2C | P42773 |
| CDKN2D | Cyclin dependent kinase inhibitor 2D | P55273 |
| CDKN3 | Cyclin dependent kinase inhibitor 3 | Q16667 |
| CDPF1 | Cysteine rich DPF motif domain containing 1 | Q6NVV7 |
| CDR2 | Cerebellar degeneration related protein 2 | Q01850 |
| CDS2 | CDP-diacylglycerol synthase 2 | O95674 |
| CDV3 | CDV3 homolog | Q9UKY7 |
| CDYL | Chromodomain Y like | Q9Y232 |
| CEACAM21 | CEA cell adhesion molecule 21 | Q3KPI0 |
| CEBPD | CCAAT enhancer binding protein delta | P49716 |
| CEBPG | CCAAT enhancer binding protein gamma | P53567 |
| CEBPZ | CCAAT enhancer binding protein zeta | Q03701 |
| CELA1 | Chymotrypsin like elastase 1 | Q9UNI1 |
| CELF1 | CUGBP Elav-like family member 1 | Q92879 |
| CELF2 | CUGBP Elav-like family member 2 | O95319 |
| CENPB | Centromere protein B | P07199 |
| CENPH | Centromere protein H | Q9H3R5 |
| CENPK | Centromere protein K | Q9BS16 |
| CENPL | Centromere protein L | Q8N0S6 |
| CENPM | Centromere protein M | Q9NSP4 |
| CENPN | Centromere protein N | Q96H22 |
| CENPP | Centromere protein P | Q6IPU0 |
| CENPQ | Centromere protein Q | Q7L2Z9 |
| CENPT | Centromere protein T | Q96BT3 |
| CENPW | Centromere protein W | Q5EE01 |
| CEP120 | Centrosomal protein 120 | Q8N960 |
| CEP135 | Centrosomal protein 135 | Q66GS9 |
| CEP164 | Centrosomal protein 164 | Q9UPV0 |
| CEP19 | Centrosomal protein 19 | Q96LK0 |
| CEP250 | Centrosomal protein 250 | Q9BV73 |
| CEP41 | Centrosomal protein 41 | Q9BYV8 |
| CEP57 | Centrosomal protein 57 | Q86XR8 |
| CEP57L1 | Centrosomal protein 57 like 1 | Q8IYX8 |
| CEP63 | Centrosomal protein 63 | Q96MT8 |
| CEP70 | Centrosomal protein 70 | Q8NHQ1 |
| CEP72 | Centrosomal protein 72 | Q9P209 |
| CEP76 | Centrosomal protein 76 | Q8TAP6 |
| CEP83 | Centrosomal protein 83 | Q9Y592 |
| CEP85 | Centrosomal protein 85 | Q6P2H3 |
| CEP85L | Centrosomal protein 85 like | Q5SZL2 |
| CEP89 | Centrosomal protein 89 | Q96ST8 |
| CEP95 | Centrosomal protein 95 | Q96GE4 |
| CEPT1 | Choline/ethanolamine phosphotransferase 1 | Q9Y6K0 |
| CERCAM | Cerebral endothelial cell adhesion molecule | Q5T4B2 |
| CERK | Ceramide kinase | Q8TCT0 |
| CERS2 | Ceramide synthase 2 | Q96G23 |
| CERS4 | Ceramide synthase 4 | Q9HA82 |
| CES1 | Carboxylesterase 1 | P23141 |
| CES2 | Carboxylesterase 2 | O00748 |
| CETN2 | Centrin 2 | P41208 |
| CETN3 | Centrin 3 | O15182 |
| CFAP20 | Cilia and flagella associated protein 20 | Q9Y6A4 |
| CFAP36 | Cilia and flagella associated protein 36 | Q96G28 |
| CFAP97 | Cilia and flagella associated protein 97 | Q9P2B7 |
| CFD | Complement factor D | P00746 |
| CFDP1 | Craniofacial development protein 1 | Q9UEE9 |
| CFH | Complement factor H | P08603 |
| CFL1 | Cofilin 1 | P23528 |
| CFL2 | Cofilin 2 | Q9Y281 |
| CFLAR | CASP8 and FADD like apoptosis regulator | O15519 |
| CFP | Complement factor properdin | P27918 |
| CGGBP1 | CGG triplet repeat binding protein 1 | Q9UFW8 |
| CGRRF1 | Cell growth regulator with ring finger domain 1 | Q99675 |
| CHAC2 | ChaC glutathione specific gamma-glutamylcyclotransferase 2 | Q8WUX2 |
| CHAF1B | Chromatin assembly factor 1 subunit B | Q13112 |
| CHAMP1 | Chromosome alignment maintaining phosphoprotein 1 | Q96JM3 |
| CHCHD1 | Coiled-coil-helix-coiled-coil-helix domain containing 1 | Q96BP2 |
| CHCHD10 | Coiled-coil-helix-coiled-coil-helix domain containing 10 | Q8WYQ3 |
| CHCHD2 | Coiled-coil-helix-coiled-coil-helix domain containing 2 | Q9Y6H1 |
| CHCHD3 | Coiled-coil-helix-coiled-coil-helix domain containing 3 | Q9NX63 |
| CHCHD4 | Coiled-coil-helix-coiled-coil-helix domain containing 4 | Q8N4Q1 |
| CHCHD5 | Coiled-coil-helix-coiled-coil-helix domain containing 5 | Q9BSY4 |
| CHCHD6 | Coiled-coil-helix-coiled-coil-helix domain containing 6 | Q9BRQ6 |
| CHCHD7 | Coiled-coil-helix-coiled-coil-helix domain containing 7 | Q9BUK0 |
| CHD1 | Chromodomain helicase DNA binding protein 1 | O14646 |
| CHD2 | Chromodomain helicase DNA binding protein 2 | O14647 |
| CHD4 | Chromodomain helicase DNA binding protein 4 | Q14839 |
| CHEK1 | Checkpoint kinase 1 | O14757 |
| CHEK2 | Checkpoint kinase 2 | O96017 |
| CHERP | Calcium homeostasis endoplasmic reticulum protein | Q8IWX8 |
| CHFR | Checkpoint with forkhead and ring finger domains | Q96EP1 |
| CHI3L2 | Chitinase 3 like 2 | Q15782 |
| CHIC2 | Cysteine rich hydrophobic domain 2 | Q9UKJ5 |
| CHID1 | Chitinase domain containing 1 | Q9BWS9 |
| CHKB | Choline kinase beta | Q9Y259 |
| CHMP1A | Charged multivesicular body protein 1A | Q9HD42 |
| CHMP1B | Charged multivesicular body protein 1B | Q7LBR1 |
| CHMP2A | Charged multivesicular body protein 2A | O43633 |
| CHMP2B | Charged multivesicular body protein 2B | Q9UQN3 |
| CHMP3 | Charged multivesicular body protein 3 | Q9Y3E7 |
| CHMP4A | Charged multivesicular body protein 4A | Q9BY43 |
| CHMP5 | Charged multivesicular body protein 5 | Q9NZZ3 |
| CHMP6 | Charged multivesicular body protein 6 | Q96FZ7 |
| CHMP7 | Charged multivesicular body protein 7 | Q8WUX9 |
| CHN1 | Chimerin 1 | P15882 |
| CHORDC1 | Cysteine and histidine rich domain containing 1 | Q9UHD1 |
| CHP1 | Calcineurin like EF-hand protein 1 | Q99653 |
| CHPF | Chondroitin polymerizing factor | Q8IZ52 |
| CHPF2 | Chondroitin polymerizing factor 2 | Q9P2E5 |
| CHRAC1 | Chromatin accessibility complex subunit 1 | Q9NRG0 |
| CHRNB1 | Cholinergic receptor nicotinic beta 1 subunit | P11230 |
| CHST10 | Carbohydrate sulfotransferase 10 | O43529 |
| CHST11 | Carbohydrate sulfotransferase 11 | Q9NPF2 |
| CHST12 | Carbohydrate sulfotransferase 12 | Q9NRB3 |
| CHST14 | Carbohydrate sulfotransferase 14 | Q8NCH0 |
| CHST7 | Carbohydrate sulfotransferase 7 | Q9NS84 |
| CHTF8 | Chromosome transmission fidelity factor 8 | P0CG13 |
| CHTOP | Chromatin target of PRMT1 | Q9Y3Y2 |
| CHUK | Component of inhibitor of nuclear factor kappa B kinase complex | O15111 |
| CHURC1 | Churchill domain containing 1 | Q8WUH1 |
| CHURC1-FNTB | CHURC1-FNTB readthrough |  |
| CIAO1 | Cytosolic iron-sulfur assembly component 1 | O76071 |
| CIAPIN1 | Cytokine induced apoptosis inhibitor 1 | Q6FI81 |
| CIB1 | Calcium and integrin binding 1 | Q99828 |
| CIDEB | Cell death inducing DFFA like effector b | Q9UHD4 |
| CIITA | Class II major histocompatibility complex transactivator | P33076 |
| CINP | Cyclin dependent kinase 2 interacting protein | Q9BW66 |
| CIPC | CLOCK interacting pacemaker | Q9C0C6 |
| CIR1 | Corepressor interacting with RBPJ, CIR1 | Q86X95 |
| CIRBP | Cold inducible RNA binding protein | Q14011 |
| CISD1 | CDGSH iron sulfur domain 1 | Q9NZ45 |
| CISD2 | CDGSH iron sulfur domain 2 | Q8N5K1 |
| CISH | Cytokine inducible SH2 containing protein | Q9NSE2 |
| CITED2 | Cbp/p300 interacting transactivator with Glu/Asp rich carboxy-terminal domain 2 | Q99967 |
| CIZ1 | CDKN1A interacting zinc finger protein 1 | Q9ULV3 |
| CKAP2 | Cytoskeleton associated protein 2 | Q8WWK9 |
| CKAP4 | Cytoskeleton associated protein 4 | Q07065 |
| CKLF | Chemokine like factor | Q9UBR5 |
| CKS1B | CDC28 protein kinase regulatory subunit 1B | P61024 |
| CKS2 | CDC28 protein kinase regulatory subunit 2 | P33552 |
| CLASP2 | Cytoplasmic linker associated protein 2 | O75122 |
| CLASRP | CLK4 associating serine/arginine rich protein | Q8N2M8 |
| CLC | Charcot-Leyden crystal galectin | Q05315 |
| CLCC1 | Chloride channel CLIC like 1 | Q96S66 |
| CLCF1 | Cardiotrophin like cytokine factor 1 | Q9UBD9 |
| CLCN3 | Chloride voltage-gated channel 3 | P51790 |
| CLDN7 | Claudin 7 | O95471 |
| CLDND1 | Claudin domain containing 1 | Q9NY35 |
| CLDND2 | Claudin domain containing 2 | Q8NHS1 |
| CLEC11A | C-type lectin domain containing 11A | Q9Y240 |
| CLEC2B | C-type lectin domain family 2 member B | Q92478 |
| CLEC2D | C-type lectin domain family 2 member D | Q9UHP7 |
| CLEC4A | C-type lectin domain family 4 member A | Q9UMR7 |
| CLIC1 | Chloride intracellular channel 1 | O00299 |
| CLIC3 | Chloride intracellular channel 3 | O95833 |
| CLIC5 | Chloride intracellular channel 5 | Q9NZA1 |
| CLINT1 | Clathrin interactor 1 | Q14677 |
| CLIP4 | CAP-Gly domain containing linker protein family member 4 | Q8N3C7 |
| CLK1 | CDC like kinase 1 | P49759 |
| CLK2 | CDC like kinase 2 | P49760 |
| CLK3 | CDC like kinase 3 | P49761 |
| CLK4 | CDC like kinase 4 | Q9HAZ1 |
| CLN3 | CLN3 lysosomal/endosomal transmembrane protein, battenin | Q13286 |
| CLN5 | CLN5 intracellular trafficking protein | O75503 |
| CLN6 | CLN6 transmembrane ER protein | Q9NWW5 |
| CLN8 | CLN8 transmembrane ER and ERGIC protein | Q9UBY8 |
| CLP1 | Cleavage factor polyribonucleotide kinase subunit 1 | Q92989 |
| CLPB | Caseinolytic mitochondrial matrix peptidase chaperone subunit B | Q9H078 |
| CLPP | Caseinolytic mitochondrial matrix peptidase proteolytic subunit | Q16740 |
| CLPTM1 | CLPTM1 regulator of GABA type A receptor forward trafficking | O96005 |
| CLPTM1L | CLPTM1 like | Q96KA5 |
| CLSTN1 | Calsyntenin 1 | O94985 |
| CLSTN3 | Calsyntenin 3 | Q9BQT9 |
| CLTA | Clathrin light chain A | P09496 |
| CLTB | Clathrin light chain B | P09497 |
| CLU | Clusterin | P10909 |
| CLUAP1 | Clusterin associated protein 1 | Q96AJ1 |
| CLUH | Clustered mitochondria homolog | O75153 |
| CLYBL | Citramalyl-CoA lyase | Q8N0X4 |
| CMAS | Cytidine monophosphate N-acetylneuraminic acid synthetase | Q8NFW8 |
| CMC1 | C-X9-C motif containing 1 | Q7Z7K0 |
| CMC2 | C-X9-C motif containing 2 | Q9NRP2 |
| CMC4 | C-X9-C motif containing 4 | P56277 |
| CMKLR1 | Chemerin chemokine-like receptor 1 | Q99788 |
| CMPK1 | Cytidine/uridine monophosphate kinase 1 | P30085 |
| CMSS1 | Cms1 ribosomal small subunit homolog | Q9BQ75 |
| CMTM3 | CKLF like MARVEL transmembrane domain containing 3 | Q96MX0 |
| CMTM6 | CKLF like MARVEL transmembrane domain containing 6 | Q9NX76 |
| CMTM7 | CKLF like MARVEL transmembrane domain containing 7 | Q96FZ5 |
| CMTR1 | Cap methyltransferase 1 | Q8N1G2 |
| CMTR2 | Cap methyltransferase 2 | Q8IYT2 |
| CNBP | CCHC-type zinc finger nucleic acid binding protein | P62633 |
| CNDP2 | Carnosine dipeptidase 2 | Q96KP4 |
| CNEP1R1 | CTD nuclear envelope phosphatase 1 regulatory subunit 1 | Q8N9A8 |
| CNFN | Cornifelin | Q9BYD5 |
| CNIH1 | Cornichon family AMPA receptor auxiliary protein 1 | O95406 |
| CNIH4 | Cornichon family AMPA receptor auxiliary protein 4 | Q9P003 |
| CNN2 | Calponin 2 | Q99439 |
| CNN3 | Calponin 3 | Q15417 |
| CNNM3 | Cyclin and CBS domain divalent metal cation transport mediator 3 | Q8NE01 |
| CNOT10 | CCR4-NOT transcription complex subunit 10 | Q9H9A5 |
| CNOT11 | CCR4-NOT transcription complex subunit 11 | Q9UKZ1 |
| CNOT2 | CCR4-NOT transcription complex subunit 2 | Q9NZN8 |
| CNOT3 | CCR4-NOT transcription complex subunit 3 | O75175 |
| CNOT4 | CCR4-NOT transcription complex subunit 4 | O95628 |
| CNOT6 | CCR4-NOT transcription complex subunit 6 | Q9ULM6 |
| CNOT6L | CCR4-NOT transcription complex subunit 6 like | Q96LI5 |
| CNOT7 | CCR4-NOT transcription complex subunit 7 | Q9UIV1 |
| CNOT8 | CCR4-NOT transcription complex subunit 8 | Q9UFF9 |
| CNP | 2',3'-cyclic nucleotide 3' phosphodiesterase | P09543 |
| CNPPD1 | Cyclin Pas1/PHO80 domain containing 1 | Q9BV87 |
| CNPY2 | Canopy FGF signaling regulator 2 | Q9Y2B0 |
| CNPY3 | Canopy FGF signaling regulator 3 | Q9BT09 |
| CNPY4 | Canopy FGF signaling regulator 4 | Q8N129 |
| CNR2 | Cannabinoid receptor 2 | P34972 |
| CNST | Consortin, connexin sorting protein | Q6PJW8 |
| CNTROB | Centrobin, centriole duplication and spindle assembly protein | Q8N137 |
| COA1 | Cytochrome c oxidase assembly factor 1 | Q9GZY4 |
| COA3 | Cytochrome c oxidase assembly factor 3 | Q9Y2R0 |
| COA4 | Cytochrome c oxidase assembly factor 4 homolog | Q9NYJ1 |
| COA5 | Cytochrome c oxidase assembly factor 5 | Q86WW8 |
| COA6 | Cytochrome c oxidase assembly factor 6 | Q5JTJ3 |
| COA7 | Cytochrome c oxidase assembly factor 7 | Q96BR5 |
| COASY | Coenzyme A synthase | Q13057 |
| COG1 | Component of oligomeric golgi complex 1 | Q8WTW3 |
| COG2 | Component of oligomeric golgi complex 2 | Q14746 |
| COG3 | Component of oligomeric golgi complex 3 | Q96JB2 |
| COG4 | Component of oligomeric golgi complex 4 |  |
| COG5 | Component of oligomeric golgi complex 5 | Q9UP83 |
| COG6 | Component of oligomeric golgi complex 6 | Q9Y2V7 |
| COG7 | Component of oligomeric golgi complex 7 | P83436 |
| COIL | Coilin | P38432 |
| COL6A2 | Collagen type VI alpha 2 chain | P12110 |
| COLGALT1 | Collagen beta(1-O)galactosyltransferase 1 | Q8NBJ5 |
| COMMD1 | Copper metabolism domain containing 1 | Q8N668 |
| COMMD10 | COMM domain containing 10 | Q9Y6G5 |
| COMMD2 | COMM domain containing 2 | Q86X83 |
| COMMD3 | COMM domain containing 3 | Q9UBI1 |
| COMMD4 | COMM domain containing 4 | Q9H0A8 |
| COMMD5 | COMM domain containing 5 | Q9GZQ3 |
| COMMD6 | COMM domain containing 6 | Q7Z4G1 |
| COMMD7 | COMM domain containing 7 | Q86VX2 |
| COMMD8 | COMM domain containing 8 | Q9NX08 |
| COMMD9 | COMM domain containing 9 | Q9P000 |
| COMT | Catechol-O-methyltransferase | P21964 |
| COMTD1 | Catechol-O-methyltransferase domain containing 1 | Q86VU5 |
| COPB1 | COPI coat complex subunit beta 1 | P53618 |
| COPB2 | COPI coat complex subunit beta 2 | P35606 |
| COPE | COPI coat complex subunit epsilon | O14579 |
| COPG1 | COPI coat complex subunit gamma 1 | Q9Y678 |
| COPS2 | COP9 signalosome subunit 2 | P61201 |
| COPS3 | COP9 signalosome subunit 3 | Q9UNS2 |
| COPS5 | COP9 signalosome subunit 5 | Q92905 |
| COPS6 | COP9 signalosome subunit 6 | Q7L5N1 |
| COPS7A | COP9 signalosome subunit 7A | Q9UBW8 |
| COPS7B | COP9 signalosome subunit 7B | Q9H9Q2 |
| COPS8 | COP9 signalosome subunit 8 | Q99627 |
| COPZ1 | COPI coat complex subunit zeta 1 | P61923 |
| COQ10A | Coenzyme Q10A | Q96MF6 |
| COQ10B | Coenzyme Q10B | Q9H8M1 |
| COQ2 | Coenzyme Q2, polyprenyltransferase | Q96H96 |
| COQ3 | Coenzyme Q3, methyltransferase | Q9NZJ6 |
| COQ4 | Coenzyme Q4 | Q9Y3A0 |
| COQ5 | Coenzyme Q5, methyltransferase | Q5HYK3 |
| COQ6 | Coenzyme Q6, monooxygenase | Q9Y2Z9 |
| COQ7 | Coenzyme Q7, hydroxylase | Q99807 |
| COQ9 | Coenzyme Q9 | O75208 |
| CORO1A | Coronin 1A | P31146 |
| CORO1B | Coronin 1B | Q9BR76 |
| CORO1C | Coronin 1C | Q9ULV4 |
| CORO2A | Coronin 2A | Q92828 |
| CORO7 | Coronin 7 | P57737 |
| COTL1 | Coactosin like F-actin binding protein 1 | Q14019 |
| COX10 | Cytochrome c oxidase assembly factor heme A:farnesyltransferase COX10 | Q12887 |
| COX11 | Cytochrome c oxidase copper chaperone COX11 | Q9Y6N1 |
| COX14 | Cytochrome c oxidase assembly factor COX14 | Q96I36 |
| COX15 | Cytochrome c oxidase assembly homolog COX15 | Q7KZN9 |
| COX16 | Cytochrome c oxidase assembly factor COX16 | Q9P0S2 |
| COX17 | Cytochrome c oxidase copper chaperone COX17 | Q14061 |
| COX19 | Cytochrome c oxidase assembly factor COX19 | Q49B96 |
| COX20 | Cytochrome c oxidase assembly factor COX20 | Q5RI15 |
| COX4I1 | Cytochrome c oxidase subunit 4I1 | P13073 |
| COX5A | Cytochrome c oxidase subunit 5A | P20674 |
| COX5B | Cytochrome c oxidase subunit 5B | P10606 |
| COX6A1 | Cytochrome c oxidase subunit 6A1 | P12074 |
| COX6B1 | Cytochrome c oxidase subunit 6B1 | P14854 |
| COX6C | Cytochrome c oxidase subunit 6C | P09669 |
| COX7A2 | Cytochrome c oxidase subunit 7A2 | P14406 |
| COX7A2L | Cytochrome c oxidase subunit 7A2 like | O14548 |
| COX7B | Cytochrome c oxidase subunit 7B | P24311 |
| COX7C | Cytochrome c oxidase subunit 7C | P15954 |
| COX8A | Cytochrome c oxidase subunit 8A | P10176 |
| CPA5 | Carboxypeptidase A5 | Q8WXQ8 |
| CPB1 | Carboxypeptidase B1 | P15086 |
| CPD | Carboxypeptidase D | O75976 |
| CPEB1 | Cytoplasmic polyadenylation element binding protein 1 | Q9BZB8 |
| CPNE1 | Copine 1 | Q99829 |
| CPNE3 | Copine 3 | O75131 |
| CPNE7 | Copine 7 | Q9UBL6 |
| CPNE8 | Copine 8 | Q86YQ8 |
| CPPED1 | Calcineurin like phosphoesterase domain containing 1 | Q9BRF8 |
| CPQ | Carboxypeptidase Q | Q9Y646 |
| CPSF1 | Cleavage and polyadenylation specific factor 1 | Q10570 |
| CPSF2 | Cleavage and polyadenylation specific factor 2 | Q9P2I0 |
| CPSF3 | Cleavage and polyadenylation specific factor 3 | Q9UKF6 |
| CPSF4 | Cleavage and polyadenylation specific factor 4 | O95639 |
| CPSF6 | Cleavage and polyadenylation specific factor 6 | Q16630 |
| CPSF7 | Cleavage and polyadenylation specific factor 7 | Q8N684 |
| CPT1A | Carnitine palmitoyltransferase 1A | P50416 |
| CPT2 | Carnitine palmitoyltransferase 2 | P23786 |
| CPVL | Carboxypeptidase vitellogenic like | Q9H3G5 |
| CRACR2A | Calcium release activated channel regulator 2A | Q9BSW2 |
| CRADD | CASP2 and RIPK1 domain containing adaptor with death domain | P78560 |
| CRAT | Carnitine O-acetyltransferase | P43155 |
| CRB3 | Crumbs cell polarity complex component 3 | Q9BUF7 |
| CRBN | Cereblon | Q96SW2 |
| CRCP | CGRP receptor component | O75575 |
| CREB1 | CAMP responsive element binding protein 1 | P16220 |
| CREB3 | CAMP responsive element binding protein 3 | O43889 |
| CREB3L2 | CAMP responsive element binding protein 3 like 2 | Q70SY1 |
| CREB3L4 | CAMP responsive element binding protein 3 like 4 | Q8TEY5 |
| CREBL2 | CAMP responsive element binding protein like 2 | O60519 |
| CREBRF | CREB3 regulatory factor | Q8IUR6 |
| CREG1 | Cellular repressor of E1A stimulated genes 1 | O75629 |
| CRELD1 | Cysteine rich with EGF like domains 1 | Q96HD1 |
| CRELD2 | Cysteine rich with EGF like domains 2 | Q6UXH1 |
| CREM | CAMP responsive element modulator | Q03060 |
| CRIP1 | Cysteine rich protein 1 | P50238 |
| CRIP2 | Cysteine rich protein 2 | P52943 |
| CRIPT | CXXC repeat containing interactor of PDZ3 domain | Q9P021 |
| CRK | CRK proto-oncogene, adaptor protein | P46108 |
| CRKL | CRK like proto-oncogene, adaptor protein | P46109 |
| CRLF3 | Cytokine receptor like factor 3 | Q8IUI8 |
| CRNKL1 | Crooked neck pre-mRNA splicing factor 1 | Q9BZJ0 |
| CROT | Carnitine O-octanoyltransferase | Q9UKG9 |
| CRTAM | Cytotoxic and regulatory T cell molecule | O95727 |
| CRTAP | Cartilage associated protein | O75718 |
| CRTC2 | CREB regulated transcription coactivator 2 | Q53ET0 |
| CRTC3 | CREB regulated transcription coactivator 3 | Q6UUV7 |
| CRY1 | Cryptochrome circadian regulator 1 | Q16526 |
| CRY2 | Cryptochrome circadian regulator 2 | Q49AN0 |
| CRYL1 | Crystallin lambda 1 | Q9Y2S2 |
| CRYZ | Crystallin zeta | Q08257 |
| CRYZL1 | Crystallin zeta like 1 | O95825 |
| CS | Citrate synthase | O75390 |
| CSAD | Cysteine sulfinic acid decarboxylase | Q9Y600 |
| CSDE1 | Cold shock domain containing E1 | O75534 |
| CSE1L | Chromosome segregation 1 like | P55060 |
| CSF2RA | Colony stimulating factor 2 receptor subunit alpha | P15509 |
| CSGALNACT1 | Chondroitin sulfate N-acetylgalactosaminyltransferase 1 | Q8TDX6 |
| CSGALNACT2 | Chondroitin sulfate N-acetylgalactosaminyltransferase 2 | Q8N6G5 |
| CSK | C-terminal Src kinase | P41240 |
| CSNK1A1 | Casein kinase 1 alpha 1 | P48729 |
| CSNK1D | Casein kinase 1 delta | P48730 |
| CSNK1E | Casein kinase 1 epsilon | P49674 |
| CSNK1G1 | Casein kinase 1 gamma 1 | Q9HCP0 |
| CSNK1G2 | Casein kinase 1 gamma 2 | P78368 |
| CSNK1G3 | Casein kinase 1 gamma 3 | Q9Y6M4 |
| CSNK2A1 | Casein kinase 2 alpha 1 | P68400 |
| CSNK2A2 | Casein kinase 2 alpha 2 | P19784 |
| CSNK2B | Casein kinase 2 beta | P67870 |
| CSRNP2 | Cysteine and serine rich nuclear protein 2 | Q9H175 |
| CSRP1 | Cysteine and glycine rich protein 1 | P21291 |
| CST3 | Cystatin C | P01034 |
| CST7 | Cystatin F | O76096 |
| CSTB | Cystatin B | P04080 |
| CSTF1 | Cleavage stimulation factor subunit 1 | Q05048 |
| CSTF2 | Cleavage stimulation factor subunit 2 | P33240 |
| CSTF2T | Cleavage stimulation factor subunit 2 tau variant | Q9H0L4 |
| CSTF3 | Cleavage stimulation factor subunit 3 | Q12996 |
| CTBP1 | C-terminal binding protein 1 | Q13363 |
| CTBP2 | C-terminal binding protein 2 | P56545 |
| CTC1 | CST telomere replication complex component 1 | Q2NKJ3 |
| CTCF | CCCTC-binding factor | P49711 |
| CTDNEP1 | CTD nuclear envelope phosphatase 1 | O95476 |
| CTDP1 | CTD phosphatase subunit 1 | Q9Y5B0 |
| CTDSP1 | CTD small phosphatase 1 | Q9GZU7 |
| CTDSP2 | CTD small phosphatase 2 | O14595 |
| CTDSPL2 | CTD small phosphatase like 2 | Q05D32 |
| CTH | Cystathionine gamma-lyase | P32929 |
| CTNNA1 | Catenin alpha 1 | P35221 |
| CTNNAL1 | Catenin alpha like 1 | Q9UBT7 |
| CTNNB1 | Catenin beta 1 | P35222 |
| CTNNBIP1 | Catenin beta interacting protein 1 | Q9NSA3 |
| CTNS | Cystinosin, lysosomal cystine transporter | O60931 |
| CTR9 | CTR9 homolog, Paf1/RNA polymerase II complex component | Q6PD62 |
| CTSA | Cathepsin A | P10619 |
| CTSB | Cathepsin B | P07858 |
| CTSC | Cathepsin C | P53634 |
| CTSD | Cathepsin D | P07339 |
| CTSF | Cathepsin F | Q9UBX1 |
| CTSG | Cathepsin G | P08311 |
| CTSH | Cathepsin H | P09668 |
| CTSL | Cathepsin L | P07711 |
| CTSO | Cathepsin O | P43234 |
| CTSS | Cathepsin S | P25774 |
| CTSW | Cathepsin W | P56202 |
| CUL1 | Cullin 1 | Q13616 |
| CUL2 | Cullin 2 | Q13617 |
| CUL3 | Cullin 3 | Q13618 |
| CUL4A | Cullin 4A | Q13619 |
| CUL4B | Cullin 4B | Q13620 |
| CUL5 | Cullin 5 | Q93034 |
| CUTA | CutA divalent cation tolerance homolog | O60888 |
| CUTC | CutC copper transporter | Q9NTM9 |
| CUX1 | Cut like homeobox 1 | P39880, Q13948 |
| CWC15 | CWC15 spliceosome associated protein homolog | Q9P013 |
| CWC22 | CWC22 spliceosome associated protein homolog | Q9HCG8 |
| CWC25 | CWC25 spliceosome associated protein homolog | Q9NXE8 |
| CWF19L1 | CWF19 like cell cycle control factor 1 | Q69YN2 |
| CWF19L2 | CWF19 like cell cycle control factor 2 | Q2TBE0 |
| CX3CR1 | C-X3-C motif chemokine receptor 1 | P49238 |
| CXCL16 | C-X-C motif chemokine ligand 16 | Q9H2A7 |
| CXCR1 | C-X-C motif chemokine receptor 1 | P25024 |
| CXCR2 | C-X-C motif chemokine receptor 2 | P25025 |
| CXCR3 | C-X-C motif chemokine receptor 3 | P49682 |
| CXCR4 | C-X-C motif chemokine receptor 4 | P61073 |
| CXCR5 | C-X-C motif chemokine receptor 5 | P32302 |
| CXCR6 | C-X-C motif chemokine receptor 6 | O00574 |
| CXorf38 | Chromosome X open reading frame 38 | Q8TB03 |
| CXXC1 | CXXC finger protein 1 | Q9P0U4 |
| CXXC5 | CXXC finger protein 5 | Q7LFL8 |
| CYB561 | Cytochrome b561 | P49447 |
| CYB561A3 | Cytochrome b561 family member A3 | Q8NBI2 |
| CYB561D1 | Cytochrome b561 family member D1 | Q8N8Q1 |
| CYB561D2 | Cytochrome b561 family member D2 | O14569 |
| CYB5A | Cytochrome b5 type A | P00167 |
| CYB5B | Cytochrome b5 type B | O43169 |
| CYB5D1 | Cytochrome b5 domain containing 1 | Q6P9G0 |
| CYB5D2 | Cytochrome b5 domain containing 2 | Q8WUJ1 |
| CYB5R1 | Cytochrome b5 reductase 1 | Q9UHQ9 |
| CYB5R3 | Cytochrome b5 reductase 3 | P00387 |
| CYB5R4 | Cytochrome b5 reductase 4 | Q7L1T6 |
| CYBA | Cytochrome b-245 alpha chain | P13498 |
| CYC1 | Cytochrome c1 | P08574 |
| CYCS | Cytochrome c, somatic | P99999 |
| CYFIP1 | Cytoplasmic FMR1 interacting protein 1 | Q7L576 |
| CYFIP2 | Cytoplasmic FMR1 interacting protein 2 | Q96F07 |
| CYP20A1 | Cytochrome P450 family 20 subfamily A member 1 | Q6UW02 |
| CYP2J2 | Cytochrome P450 family 2 subfamily J member 2 | P51589 |
| CYP2R1 | Cytochrome P450 family 2 subfamily R member 1 | Q6VVX0 |
| CYP4F22 | Cytochrome P450 family 4 subfamily F member 22 | Q6NT55 |
| CYP4V2 | Cytochrome P450 family 4 subfamily V member 2 | Q6ZWL3 |
| CYP51A1 | Cytochrome P450 family 51 subfamily A member 1 | Q16850 |
| CYSTM1 | Cysteine rich transmembrane module containing 1 | Q9H1C7 |
| CYTH1 | Cytohesin 1 | Q15438 |
| CYTH2 | Cytohesin 2 | Q99418 |
| CYTH3 | Cytohesin 3 | O43739 |
| CYTH4 | Cytohesin 4 | Q9UIA0 |
| CYTIP | Cytohesin 1 interacting protein | O60759 |
| D2HGDH | D-2-hydroxyglutarate dehydrogenase | Q8N465 |
| DAAM1 | Dishevelled associated activator of morphogenesis 1 | Q9Y4D1 |
| DAB2 | DAB adaptor protein 2 | P98082 |
| DAD1 | Defender against cell death 1 | P61803 |
| DAGLB | Diacylglycerol lipase beta | Q8NCG7 |
| DALRD3 | DALR anticodon binding domain containing 3 | Q5D0E6 |
| DAP | Death associated protein | P51397 |
| DAP3 | Death associated protein 3 | P51398 |
| DAPK2 | Death associated protein kinase 2 | Q9UIK4 |
| DAPK3 | Death associated protein kinase 3 | O43293 |
| DAPP1 | Dual adaptor of phosphotyrosine and 3-phosphoinositides 1 | Q9UN19 |
| DARS2 | Aspartyl-tRNA synthetase 2, mitochondrial | Q6PI48 |
| DAXX | Death domain associated protein | Q9UER7 |
| DAZAP1 | DAZ associated protein 1 | Q96EP5 |
| DAZAP2 | DAZ associated protein 2 | Q15038 |
| DBI | Diazepam binding inhibitor, acyl-CoA binding protein | P07108 |
| DBN1 | Drebrin 1 | Q16643 |
| DBNDD2 | Dysbindin domain containing 2 | Q9BQY9 |
| DBNL | Drebrin like | Q9UJU6 |
| DBP | D-box binding PAR bZIP transcription factor | Q10586 |
| DBR1 | Debranching RNA lariats 1 | Q9UK59 |
| DBT | Dihydrolipoamide branched chain transacylase E2 | P11182 |
| DCAF11 | DDB1 and CUL4 associated factor 11 | Q8TEB1 |
| DCAF12 | DDB1 and CUL4 associated factor 12 | Q5T6F0 |
| DCAF4 | DDB1 and CUL4 associated factor 4 | Q8WV16 |
| DCAF7 | DDB1 and CUL4 associated factor 7 | P61962 |
| DCAF8 | DDB1 and CUL4 associated factor 8 | Q5TAQ9 |
| DCAKD | Dephospho-CoA kinase domain containing | Q8WVC6 |
| DCK | Deoxycytidine kinase | P27707 |
| DCLRE1A | DNA cross-link repair 1A | Q6PJP8 |
| DCLRE1B | DNA cross-link repair 1B | Q9H816 |
| DCLRE1C | DNA cross-link repair 1C | Q96SD1 |
| DCP1A | Decapping mRNA 1A | Q9NPI6 |
| DCP1B | Decapping mRNA 1B | Q8IZD4 |
| DCP2 | Decapping mRNA 2 | Q8IU60 |
| DCPS | Decapping enzyme, scavenger | Q96C86 |
| DCTD | DCMP deaminase | P32321 |
| DCTN1 | Dynactin subunit 1 | Q14203 |
| DCTN2 | Dynactin subunit 2 | Q13561 |
| DCTN3 | Dynactin subunit 3 | O75935 |
| DCTN4 | Dynactin subunit 4 | Q9UJW0 |
| DCTN6 | Dynactin subunit 6 | O00399 |
| DCTPP1 | DCTP pyrophosphatase 1 | Q9H773 |
| DCUN1D1 | Defective in cullin neddylation 1 domain containing 1 | Q96GG9 |
| DCUN1D2 | Defective in cullin neddylation 1 domain containing 2 | Q6PH85 |
| DCUN1D4 | Defective in cullin neddylation 1 domain containing 4 | Q92564 |
| DCUN1D5 | Defective in cullin neddylation 1 domain containing 5 | Q9BTE7 |
| DCXR | Dicarbonyl and L-xylulose reductase | Q7Z4W1 |
| DDA1 | DET1 and DDB1 associated 1 | Q9BW61 |
| DDAH2 | Dimethylarginine dimethylaminohydrolase 2 | O95865 |
| DDB1 | Damage specific DNA binding protein 1 | Q16531 |
| DDB2 | Damage specific DNA binding protein 2 | Q92466 |
| DDHD1 | DDHD domain containing 1 | Q8NEL9 |
| DDHD2 | DDHD domain containing 2 | O94830 |
| DDI2 | DNA damage inducible 1 homolog 2 | Q5TDH0 |
| DDIT3 | DNA damage inducible transcript 3 | P35638 |
| DDIT4 | DNA damage inducible transcript 4 | Q9NX09 |
| DDOST | Dolichyl-diphosphooligosaccharide--protein glycosyltransferase non-catalytic subunit | P39656 |
| DDRGK1 | DDRGK domain containing 1 | Q96HY6 |
| DDT | D-dopachrome tautomerase | P30046 |
| DDX1 | DEAD-box helicase 1 | Q92499 |
| DDX11 | DEAD/H-box helicase 11 | Q96FC9 |
| DDX17 | DEAD-box helicase 17 | Q92841 |
| DDX18 | DEAD-box helicase 18 | Q9NVP1 |
| DDX19B | DEAD-box helicase 19B | Q9UMR2 |
| DDX20 | DEAD-box helicase 20 | Q9UHI6 |
| DDX21 | DExD-box helicase 21 | Q9NR30 |
| DDX23 | DEAD-box helicase 23 | Q9BUQ8 |
| DDX24 | DEAD-box helicase 24 | Q9GZR7 |
| DDX27 | DEAD-box helicase 27 |  |
| DDX28 | DEAD-box helicase 28 | Q9NUL7 |
| DDX31 | DEAD-box helicase 31 | Q9H8H2 |
| DDX39A | DExD-box helicase 39A | O00148 |
| DDX39B | DExD-box helicase 39B | Q13838 |
| DDX3X | DEAD-box helicase 3 X-linked | O00571 |
| DDX3Y | DEAD-box helicase 3 Y-linked | O15523 |
| DDX41 | DEAD-box helicase 41 | Q9UJV9 |
| DDX42 | DEAD-box helicase 42 | Q86XP3 |
| DDX46 | DEAD-box helicase 46 | Q7L014 |
| DDX47 | DEAD-box helicase 47 | Q9H0S4 |
| DDX49 | DEAD-box helicase 49 | Q9Y6V7 |
| DDX5 | DEAD-box helicase 5 | P17844 |
| DDX50 | DExD-box helicase 50 | Q9BQ39 |
| DDX54 | DEAD-box helicase 54 | Q8TDD1 |
| DDX55 | DEAD-box helicase 55 | Q8NHQ9 |
| DDX56 | DEAD-box helicase 56 | Q9NY93 |
| DDX59 | DEAD-box helicase 59 | Q5T1V6 |
| DDX6 | DEAD-box helicase 6 | P26196 |
| DDX60 | DExD/H-box helicase 60 | Q8IY21 |
| DEAF1 | DEAF1 transcription factor | O75398 |
| DECR1 | 2,4-dienoyl-CoA reductase 1 | Q16698 |
| DECR2 | 2,4-dienoyl-CoA reductase 2 | Q9NUI1 |
| DEDD | Death effector domain containing | O75618 |
| DEDD2 | Death effector domain containing 2 | Q8WXF8 |
| DEF6 | DEF6 guanine nucleotide exchange factor | Q9H4E7 |
| DEFA1 | Defensin alpha 1 | P59665 |
| DEGS1 | Delta 4-desaturase, sphingolipid 1 | O15121 |
| DEGS2 | Delta 4-desaturase, sphingolipid 2 | Q6QHC5 |
| DEK | DEK proto-oncogene | P35659 |
| DENND1A | DENN domain containing 1A | Q8TEH3 |
| DENND1B | DENN domain containing 1B | Q6P3S1 |
| DENND1C | DENN domain containing 1C | Q8IV53 |
| DENND2D | DENN domain containing 2D | Q9H6A0 |
| DENND4B | DENN domain containing 4B | O75064 |
| DENND5A | DENN domain containing 5A | Q6IQ26 |
| DENND6A | DENN domain containing 6A | Q8IWF6 |
| DENR | Density regulated re-initiation and release factor | O43583 |
| DEPDC5 | DEP domain containing 5, GATOR1 subcomplex subunit | O75140 |
| DEPDC7 | DEP domain containing 7 | Q96QD5 |
| DERA | Deoxyribose-phosphate aldolase | Q9Y315 |
| DERL1 | Derlin 1 | Q9BUN8 |
| DERL2 | Derlin 2 | Q9GZP9 |
| DESI1 | Desumoylating isopeptidase 1 | Q6ICB0 |
| DESI2 | Desumoylating isopeptidase 2 | Q9BSY9 |
| DEXI | Dexi homolog | O95424 |
| DFFA | DNA fragmentation factor subunit alpha | O00273 |
| DFFB | DNA fragmentation factor subunit beta | O76075 |
| DGCR2 | DiGeorge syndrome critical region gene 2 | P98153 |
| DGCR6 | DiGeorge syndrome critical region gene 6 | Q14129 |
| DGCR6L | DiGeorge syndrome critical region gene 6 like | Q9BY27 |
| DGCR8 | DGCR8 microprocessor complex subunit | Q8WYQ5 |
| DGKA | Diacylglycerol kinase alpha | P23743 |
| DGKD | Diacylglycerol kinase delta | Q16760 |
| DGKQ | Diacylglycerol kinase theta | P52824 |
| DGKZ | Diacylglycerol kinase zeta | Q13574 |
| DGUOK | Deoxyguanosine kinase | Q16854 |
| DHCR24 | 24-dehydrocholesterol reductase | Q15392 |
| DHCR7 | 7-dehydrocholesterol reductase | Q9UBM7 |
| DHDDS | Dehydrodolichyl diphosphate synthase subunit | Q86SQ9 |
| DHDH | Dihydrodiol dehydrogenase | Q9UQ10 |
| DHODH | Dihydroorotate dehydrogenase (quinone) | Q02127 |
| DHPS | Deoxyhypusine synthase | P49366 |
| DHRS1 | Dehydrogenase/reductase 1 | Q96LJ7 |
| DHRS12 | Dehydrogenase/reductase 12 | A0PJE2 |
| DHRS13 | Dehydrogenase/reductase 13 | Q6UX07 |
| DHRS3 | Dehydrogenase/reductase 3 | O75911 |
| DHRS4 | Dehydrogenase/reductase 4 | Q9BTZ2 |
| DHRS4L2 | Dehydrogenase/reductase 4 like 2 | Q6PKH6 |
| DHRS7 | Dehydrogenase/reductase 7 | Q9Y394 |
| DHRS7B | Dehydrogenase/reductase 7B | Q6IAN0 |
| DHRSX | Dehydrogenase/reductase X-linked | Q8N5I4 |
| DHTKD1 | Dehydrogenase E1 and transketolase domain containing 1 | Q96HY7 |
| DHX16 | DEAH-box helicase 16 | O60231 |
| DHX29 | DExH-box helicase 29 | Q7Z478 |
| DHX30 | DExH-box helicase 30 | Q7L2E3 |
| DHX32 | DEAH-box helicase 32 (putative) | Q7L7V1 |
| DHX34 | DExH-box helicase 34 | Q14147 |
| DHX35 | DEAH-box helicase 35 | Q9H5Z1 |
| DHX36 | DEAH-box helicase 36 | Q9H2U1 |
| DHX38 | DEAH-box helicase 38 | Q92620 |
| DHX40 | DEAH-box helicase 40 | Q8IX18 |
| DHX58 | DExH-box helicase 58 | Q96C10 |
| DHX8 | DEAH-box helicase 8 | Q14562 |
| DIABLO | Diablo IAP-binding mitochondrial protein | Q9NR28 |
| DIDO1 | Death inducer-obliterator 1 | Q9BTC0 |
| DIMT1 | DIM1 rRNA methyltransferase and ribosome maturation factor | Q9UNQ2 |
| DIP2A | Disco interacting protein 2 homolog A | Q14689 |
| DIS3 | DIS3 homolog, exosome endoribonuclease and 3'-5' exoribonuclease | Q9Y2L1 |
| DIS3L | DIS3 like exosome 3'-5' exoribonuclease | Q8TF46 |
| DIS3L2 | DIS3 like 3'-5' exoribonuclease 2 | Q8IYB7 |
| DKC1 | Dyskerin pseudouridine synthase 1 | O60832 |
| DKK3 | Dickkopf WNT signaling pathway inhibitor 3 | Q9UBP4 |
| DLAT | Dihydrolipoamide S-acetyltransferase | P10515 |
| DLD | Dihydrolipoamide dehydrogenase | P09622 |
| DLGAP4 | DLG associated protein 4 | Q9Y2H0 |
| DLGAP5 | DLG associated protein 5 | Q15398 |
| DLL3 | Delta like canonical Notch ligand 3 | Q9NYJ7 |
| DLST | Dihydrolipoamide S-succinyltransferase | P36957 |
| DMAP1 | DNA methyltransferase 1 associated protein 1 | Q9NPF5 |
| DMKN | Dermokine | Q6E0U4 |
| DMTF1 | Cyclin D binding myb like transcription factor 1 | Q9Y222 |
| DMTN | Dematin actin binding protein | Q08495 |
| DMWD | DM1 locus, WD repeat containing | Q09019 |
| DNAJA1 | DnaJ heat shock protein family (Hsp40) member A1 | P31689 |
| DNAJA3 | DnaJ heat shock protein family (Hsp40) member A3 | Q96EY1 |
| DNAJA4 | DnaJ heat shock protein family (Hsp40) member A4 | Q8WW22 |
| DNAJB1 | DnaJ heat shock protein family (Hsp40) member B1 | P25685 |
| DNAJB11 | DnaJ heat shock protein family (Hsp40) member B11 | Q9UBS4 |
| DNAJB12 | DnaJ heat shock protein family (Hsp40) member B12 | Q9NXW2 |
| DNAJB14 | DnaJ heat shock protein family (Hsp40) member B14 | Q8TBM8 |
| DNAJB4 | DnaJ heat shock protein family (Hsp40) member B4 | Q9UDY4 |
| DNAJB6 | DnaJ heat shock protein family (Hsp40) member B6 | O75190 |
| DNAJB9 | DnaJ heat shock protein family (Hsp40) member B9 | Q9UBS3 |
| DNAJC10 | DnaJ heat shock protein family (Hsp40) member C10 | Q8IXB1 |
| DNAJC11 | DnaJ heat shock protein family (Hsp40) member C11 | Q9NVH1 |
| DNAJC14 | DnaJ heat shock protein family (Hsp40) member C14 | Q6Y2X3 |
| DNAJC15 | DnaJ heat shock protein family (Hsp40) member C15 | Q9Y5T4 |
| DNAJC16 | DnaJ heat shock protein family (Hsp40) member C16 | Q9Y2G8 |
| DNAJC17 | DnaJ heat shock protein family (Hsp40) member C17 | Q9NVM6 |
| DNAJC19 | DnaJ heat shock protein family (Hsp40) member C19 | Q96DA6 |
| DNAJC2 | DnaJ heat shock protein family (Hsp40) member C2 | Q99543 |
| DNAJC3 | DnaJ heat shock protein family (Hsp40) member C3 | Q13217 |
| DNAJC30 | DnaJ heat shock protein family (Hsp40) member C30 | Q96LL9 |
| DNAJC4 | DnaJ heat shock protein family (Hsp40) member C4 | Q9NNZ3 |
| DNAJC5 | DnaJ heat shock protein family (Hsp40) member C5 | Q9H3Z4 |
| DNAJC7 | DnaJ heat shock protein family (Hsp40) member C7 | Q99615 |
| DNAJC8 | DnaJ heat shock protein family (Hsp40) member C8 | O75937 |
| DNAL1 | Dynein axonemal light chain 1 | Q4LDG9 |
| DNAL4 | Dynein axonemal light chain 4 | O96015 |
| DNASE1L1 | Deoxyribonuclease 1 like 1 | P49184 |
| DNASE2 | Deoxyribonuclease 2, lysosomal | O00115 |
| DNM1L | Dynamin 1 like | O00429 |
| DNM2 | Dynamin 2 | P50570 |
| DNMT3A | DNA methyltransferase 3 alpha | Q9Y6K1 |
| DNPEP | Aspartyl aminopeptidase | Q9ULA0 |
| DNPH1 | 2'-deoxynucleoside 5'-phosphate N-hydrolase 1 | O43598 |
| DNTTIP1 | Deoxynucleotidyltransferase terminal interacting protein 1 | Q9H147 |
| DNTTIP2 | Deoxynucleotidyltransferase terminal interacting protein 2 | Q5QJE6 |
| DOCK2 | Dedicator of cytokinesis 2 | Q92608 |
| DOHH | Deoxyhypusine hydroxylase | Q9BU89 |
| DOK1 | Docking protein 1 | Q99704 |
| DOK2 | Docking protein 2 | O60496 |
| DOLK | Dolichol kinase | Q9UPQ8 |
| DOLPP1 | Dolichyldiphosphatase 1 | Q86YN1 |
| DPAGT1 | Dolichyl-phosphate N-acetylglucosaminephosphotransferase 1 | Q9H3H5 |
| DPCD | Deleted in primary ciliary dyskinesia homolog (mouse) | Q9BVM2 |
| DPEP2 | Dipeptidase 2 | Q9H4A9 |
| DPF2 | Double PHD fingers 2 | Q92785 |
| DPH1 | Diphthamide biosynthesis 1 | Q9BZG8 |
| DPH2 | Diphthamide biosynthesis 2 | Q9BQC3 |
| DPH3 | Diphthamide biosynthesis 3 | Q96FX2 |
| DPH5 | Diphthamide biosynthesis 5 | Q9H2P9 |
| DPH6 | Diphthamine biosynthesis 6 | Q7L8W6 |
| DPH7 | Diphthamide biosynthesis 7 | Q9BTV6 |
| DPM1 | Dolichyl-phosphate mannosyltransferase subunit 1, catalytic | O60762 |
| DPM2 | Dolichyl-phosphate mannosyltransferase subunit 2, regulatory | O94777 |
| DPM3 | Dolichyl-phosphate mannosyltransferase subunit 3, regulatory | Q9P2X0 |
| DPP3 | Dipeptidyl peptidase 3 | Q9NY33 |
| DPP4 | Dipeptidyl peptidase 4 | P27487 |
| DPP7 | Dipeptidyl peptidase 7 | Q9UHL4 |
| DPP8 | Dipeptidyl peptidase 8 | Q6V1X1 |
| DPP9 | Dipeptidyl peptidase 9 | Q86TI2 |
| DPY19L3 | Dpy-19 like C-mannosyltransferase 3 | Q6ZPD9 |
| DPY19L4 | Dpy-19 like 4 | Q7Z388 |
| DPY30 | Dpy-30 histone methyltransferase complex regulatory subunit | Q9C005 |
| DPYD | Dihydropyrimidine dehydrogenase | Q12882 |
| DPYSL2 | Dihydropyrimidinase like 2 | Q16555 |
| DR1 | Down-regulator of transcription 1 | Q01658 |
| DRAM1 | DNA damage regulated autophagy modulator 1 | Q8N682 |
| DRAP1 | DR1 associated protein 1 | Q14919 |
| DRG1 | Developmentally regulated GTP binding protein 1 | Q9Y295 |
| DRG2 | Developmentally regulated GTP binding protein 2 | P55039 |
| DSC1 | Desmocollin 1 | Q08554 |
| DSN1 | DSN1 component of MIS12 kinetochore complex | Q9H410 |
| DSTN | Destrin, actin depolymerizing factor | P60981 |
| DSTYK | Dual serine/threonine and tyrosine protein kinase | Q6XUX3 |
| DTD1 | D-aminoacyl-tRNA deacylase 1 | Q8TEA8 |
| DTD2 | D-aminoacyl-tRNA deacylase 2 | Q96FN9 |
| DTHD1 | Death domain containing 1 | Q6ZMT9 |
| DTNA | Dystrobrevin alpha | Q9Y4J8 |
| DTNB | Dystrobrevin beta | O60941 |
| DTNBP1 | Dystrobrevin binding protein 1 | Q96EV8 |
| DTWD1 | DTW domain containing 1 | Q8N5C7 |
| DTX2 | Deltex E3 ubiquitin ligase 2 | Q86UW9 |
| DTX3 | Deltex E3 ubiquitin ligase 3 | Q8N9I9 |
| DTX3L | Deltex E3 ubiquitin ligase 3L | Q8TDB6 |
| DTYMK | Deoxythymidylate kinase | P23919 |
| DUS1L | Dihydrouridine synthase 1 like | Q6P1R4 |
| DUS2 | Dihydrouridine synthase 2 | Q9NX74 |
| DUS3L | Dihydrouridine synthase 3 like | Q96G46 |
| DUSP10 | Dual specificity phosphatase 10 | Q9Y6W6 |
| DUSP11 | Dual specificity phosphatase 11 | O75319 |
| DUSP12 | Dual specificity phosphatase 12 | Q9UNI6 |
| DUSP18 | Dual specificity phosphatase 18 | Q8NEJ0 |
| DUSP2 | Dual specificity phosphatase 2 | Q05923 |
| DUSP22 | Dual specificity phosphatase 22 | Q9NRW4 |
| DUSP23 | Dual specificity phosphatase 23 | Q9BVJ7 |
| DUSP28 | Dual specificity phosphatase 28 | Q4G0W2 |
| DUSP3 | Dual specificity phosphatase 3 | P51452 |
| DUSP5 | Dual specificity phosphatase 5 | Q16690 |
| DUSP6 | Dual specificity phosphatase 6 | Q16828 |
| DUT | Deoxyuridine triphosphatase | P33316 |
| DVL2 | Dishevelled segment polarity protein 2 | O14641 |
| DXO | Decapping exoribonuclease | O77932 |
| DYM | Dymeclin | Q7RTS9 |
| DYNC1LI1 | Dynein cytoplasmic 1 light intermediate chain 1 | Q9Y6G9 |
| DYNC1LI2 | Dynein cytoplasmic 1 light intermediate chain 2 | O43237 |
| DYNC2LI1 | Dynein cytoplasmic 2 light intermediate chain 1 | Q8TCX1 |
| DYNLL1 | Dynein light chain LC8-type 1 | P63167 |
| DYNLRB1 | Dynein light chain roadblock-type 1 | Q9NP97 |
| DYNLRB2 | Dynein light chain roadblock-type 2 | Q8TF09 |
| DYNLT1 | Dynein light chain Tctex-type 1 | P63172 |
| DYNLT3 | Dynein light chain Tctex-type 3 | P51808 |
| DYRK1A | Dual specificity tyrosine phosphorylation regulated kinase 1A | Q13627 |
| DYRK1B | Dual specificity tyrosine phosphorylation regulated kinase 1B | Q9Y463 |
| DYRK2 | Dual specificity tyrosine phosphorylation regulated kinase 2 | Q92630 |
| DZIP3 | DAZ interacting zinc finger protein 3 | Q86Y13 |
| E2F3 | E2F transcription factor 3 | O00716 |
| E2F4 | E2F transcription factor 4 | Q16254 |
| E4F1 | E4F transcription factor 1 | Q66K89 |
| EAF1 | ELL associated factor 1 | Q96JC9 |
| EAF2 | ELL associated factor 2 | Q96CJ1 |
| EARS2 | Glutamyl-tRNA synthetase 2, mitochondrial | Q5JPH6 |
| EBAG9 | Estrogen receptor binding site associated antigen 9 | O00559 |
| EBLN2 | Endogenous Bornavirus like nucleoprotein 2 | Q6P2I7 |
| EBNA1BP2 | EBNA1 binding protein 2 | Q99848 |
| EBP | EBP cholestenol delta-isomerase | Q15125 |
| EBPL | EBP like | Q9BY08 |
| ECD | Ecdysoneless cell cycle regulator | O95905 |
| ECE1 | Endothelin converting enzyme 1 | P42892 |
| ECH1 | Enoyl-CoA hydratase 1 | Q13011 |
| ECHDC1 | Ethylmalonyl-CoA decarboxylase 1 | Q9NTX5 |
| ECHDC2 | Enoyl-CoA hydratase domain containing 2 | Q86YB7 |
| ECHS1 | Enoyl-CoA hydratase, short chain 1 | P30084 |
| ECI1 | Enoyl-CoA delta isomerase 1 | P42126 |
| ECI2 | Enoyl-CoA delta isomerase 2 | O75521 |
| ECSIT | ECSIT signaling integrator | Q9BQ95 |
| ECT2 | Epithelial cell transforming 2 | Q9H8V3 |
| EDAR | Ectodysplasin A receptor | Q9UNE0 |
| EDARADD | EDAR associated death domain | Q8WWZ3 |
| EDC3 | Enhancer of mRNA decapping 3 | Q96F86 |
| EDC4 | Enhancer of mRNA decapping 4 | Q6P2E9 |
| EDEM1 | ER degradation enhancing alpha-mannosidase like protein 1 | Q92611 |
| EDEM2 | ER degradation enhancing alpha-mannosidase like protein 2 | Q9BV94 |
| EDF1 | Endothelial differentiation related factor 1 | O60869 |
| EED | Embryonic ectoderm development | O75530 |
| EEF1A1 | Eukaryotic translation elongation factor 1 alpha 1 | P68104 |
| EEF1B2 | Eukaryotic translation elongation factor 1 beta 2 | P24534 |
| EEF1D | Eukaryotic translation elongation factor 1 delta | P29692 |
| EEF1E1 | Eukaryotic translation elongation factor 1 epsilon 1 | O43324 |
| EEF1G | Eukaryotic translation elongation factor 1 gamma | P26641 |
| EEF2 | Eukaryotic translation elongation factor 2 | P13639 |
| EEF2KMT | Eukaryotic elongation factor 2 lysine methyltransferase | Q96G04 |
| EEFSEC | Eukaryotic elongation factor, selenocysteine-tRNA specific | P57772 |
| EEPD1 | Endonuclease/exonuclease/phosphatase family domain containing 1 | Q7L9B9 |
| EFCAB11 | EF-hand calcium binding domain 11 | Q9BUY7 |
| EFCAB14 | EF-hand calcium binding domain 14 | O75071 |
| EFCAB7 | EF-hand calcium binding domain 7 | A8K855 |
| EFHD2 | EF-hand domain family member D2 | Q96C19 |
| EFNA1 | Ephrin A1 | P20827 |
| EFNA4 | Ephrin A4 | P52798 |
| EFR3A | EFR3 homolog A | Q14156 |
| EFTUD2 | Elongation factor Tu GTP binding domain containing 2 | Q15029 |
| EGLN2 | Egl-9 family hypoxia inducible factor 2 | Q96KS0 |
| EHD1 | EH domain containing 1 | Q9H4M9 |
| EHD3 | EH domain containing 3 | Q9NZN3 |
| EHHADH | Enoyl-CoA hydratase and 3-hydroxyacyl CoA dehydrogenase | Q08426 |
| EHMT1 | Euchromatic histone lysine methyltransferase 1 | Q9H9B1 |
| EI24 | EI24 autophagy associated transmembrane protein | O14681 |
| EID1 | EP300 interacting inhibitor of differentiation 1 | Q9Y6B2 |
| EID2B | EP300 interacting inhibitor of differentiation 2B | Q96D98 |
| EID3 | EP300 interacting inhibitor of differentiation 3 | Q8N140 |
| EIF1 | Eukaryotic translation initiation factor 1 | P41567 |
| EIF1AD | Eukaryotic translation initiation factor 1A domain containing | Q8N9N8 |
| EIF1AX | Eukaryotic translation initiation factor 1A X-linked | P47813 |
| EIF1AY | Eukaryotic translation initiation factor 1A Y-linked | O14602 |
| EIF1B | Eukaryotic translation initiation factor 1B | O60739 |
| EIF2A | Eukaryotic translation initiation factor 2A | Q9BY44 |
| EIF2AK1 | Eukaryotic translation initiation factor 2 alpha kinase 1 | Q9BQI3 |
| EIF2AK2 | Eukaryotic translation initiation factor 2 alpha kinase 2 | P19525 |
| EIF2AK4 | Eukaryotic translation initiation factor 2 alpha kinase 4 | Q9P2K8 |
| EIF2B1 | Eukaryotic translation initiation factor 2B subunit alpha | Q14232 |
| EIF2B2 | Eukaryotic translation initiation factor 2B subunit beta | P49770 |
| EIF2B3 | Eukaryotic translation initiation factor 2B subunit gamma | Q9NR50 |
| EIF2B4 | Eukaryotic translation initiation factor 2B subunit delta | Q9UI10 |
| EIF2B5 | Eukaryotic translation initiation factor 2B subunit epsilon | Q13144 |
| EIF2D | Eukaryotic translation initiation factor 2D | P41214 |
| EIF2S1 | Eukaryotic translation initiation factor 2 subunit alpha | P05198 |
| EIF2S2 | Eukaryotic translation initiation factor 2 subunit beta | P20042 |
| EIF2S3 | Eukaryotic translation initiation factor 2 subunit gamma | P41091 |
| EIF3C | Eukaryotic translation initiation factor 3 subunit C | Q99613 |
| EIF3D | Eukaryotic translation initiation factor 3 subunit D | O15371 |
| EIF3E | Eukaryotic translation initiation factor 3 subunit E | P60228 |
| EIF3F | Eukaryotic translation initiation factor 3 subunit F | O00303 |
| EIF3G | Eukaryotic translation initiation factor 3 subunit G | O75821 |
| EIF3H | Eukaryotic translation initiation factor 3 subunit H | O15372 |
| EIF3I | Eukaryotic translation initiation factor 3 subunit I | Q13347 |
| EIF3J | Eukaryotic translation initiation factor 3 subunit J | O75822 |
| EIF3K | Eukaryotic translation initiation factor 3 subunit K | Q9UBQ5 |
| EIF3L | Eukaryotic translation initiation factor 3 subunit L | Q9Y262 |
| EIF3M | Eukaryotic translation initiation factor 3 subunit M | Q7L2H7 |
| EIF4A1 | Eukaryotic translation initiation factor 4A1 | P60842 |
| EIF4A2 | Eukaryotic translation initiation factor 4A2 | Q14240 |
| EIF4A3 | Eukaryotic translation initiation factor 4A3 | P38919 |
| EIF4B | Eukaryotic translation initiation factor 4B | P23588 |
| EIF4E | Eukaryotic translation initiation factor 4E | P06730 |
| EIF4E2 | Eukaryotic translation initiation factor 4E family member 2 | O60573 |
| EIF4E3 | Eukaryotic translation initiation factor 4E family member 3 | Q8N5X7 |
| EIF4EBP1 | Eukaryotic translation initiation factor 4E binding protein 1 | Q13541 |
| EIF4EBP2 | Eukaryotic translation initiation factor 4E binding protein 2 | Q13542 |
| EIF4EBP3 | Eukaryotic translation initiation factor 4E binding protein 3 | O60516 |
| EIF4ENIF1 | Eukaryotic translation initiation factor 4E nuclear import factor 1 | Q9NRA8 |
| EIF4H | Eukaryotic translation initiation factor 4H | Q15056 |
| EIF5 | Eukaryotic translation initiation factor 5 | P55010 |
| EIF5A | Eukaryotic translation initiation factor 5A | P63241 |
| EIF5A2 | Eukaryotic translation initiation factor 5A2 | Q9GZV4 |
| EIF5B | Eukaryotic translation initiation factor 5B | O60841 |
| EIF6 | Eukaryotic translation initiation factor 6 | P56537 |
| ELAC1 | ElaC ribonuclease Z 1 | Q9H777 |
| ELAC2 | ElaC ribonuclease Z 2 | Q9BQ52 |
| ELAVL1 | ELAV like RNA binding protein 1 | Q15717 |
| ELF1 | E74 like ETS transcription factor 1 | P32519 |
| ELF2 | E74 like ETS transcription factor 2 | Q15723 |
| ELF4 | E74 like ETS transcription factor 4 | Q99607 |
| ELK1 | ETS transcription factor ELK1 | P19419 |
| ELK3 | ETS transcription factor ELK3 | P41970 |
| ELK4 | ETS transcription factor ELK4 | P28324 |
| ELL | Elongation factor for RNA polymerase II | P55199 |
| ELMO1 | Engulfment and cell motility 1 | Q92556 |
| ELMO2 | Engulfment and cell motility 2 | Q96JJ3 |
| ELMOD2 | ELMO domain containing 2 | Q8IZ81 |
| ELMOD3 | ELMO domain containing 3 | Q96FG2 |
| ELOF1 | Elongation factor 1 | P60002 |
| ELOVL1 | ELOVL fatty acid elongase 1 | Q9BW60 |
| ELOVL4 | ELOVL fatty acid elongase 4 | Q9GZR5 |
| ELOVL5 | ELOVL fatty acid elongase 5 | Q9NYP7 |
| ELOVL6 | ELOVL fatty acid elongase 6 | Q9H5J4 |
| ELP2 | Elongator acetyltransferase complex subunit 2 | Q6IA86 |
| ELP3 | Elongator acetyltransferase complex subunit 3 | Q9H9T3 |
| ELP4 | Elongator acetyltransferase complex subunit 4 | Q96EB1 |
| ELP5 | Elongator acetyltransferase complex subunit 5 | Q8TE02 |
| ELP6 | Elongator acetyltransferase complex subunit 6 | Q0PNE2 |
| EMB | Embigin | Q6PCB8 |
| EMC1 | ER membrane protein complex subunit 1 | Q8N766 |
| EMC10 | ER membrane protein complex subunit 10 | Q5UCC4 |
| EMC2 | ER membrane protein complex subunit 2 | Q15006 |
| EMC3 | ER membrane protein complex subunit 3 | Q9P0I2 |
| EMC4 | ER membrane protein complex subunit 4 | Q5J8M3 |
| EMC6 | ER membrane protein complex subunit 6 | Q9BV81 |
| EMC7 | ER membrane protein complex subunit 7 | Q9NPA0 |
| EMC8 | ER membrane protein complex subunit 8 | O43402 |
| EMC9 | ER membrane protein complex subunit 9 | Q9Y3B6 |
| EMD | Emerin | P50402 |
| EMG1 | EMG1 N1-specific pseudouridine methyltransferase | Q92979 |
| EMP3 | Epithelial membrane protein 3 | P54852 |
| ENC1 | Ectodermal-neural cortex 1 | O14682 |
| ENDOD1 | Endonuclease domain containing 1 | O94919 |
| ENDOV | Endonuclease V | Q8N8Q3 |
| ENG | Endoglin | P17813 |
| ENO1 | Enolase 1 | P06733 |
| ENO2 | Enolase 2 | P09104 |
| ENO3 | Enolase 3 | P13929 |
| ENOPH1 | Enolase-phosphatase 1 | Q9UHY7 |
| ENOSF1 | Enolase superfamily member 1 | Q7L5Y1 |
| ENOX2 | Ecto-NOX disulfide-thiol exchanger 2 | Q16206 |
| ENPP2 | Ectonucleotide pyrophosphatase/phosphodiesterase 2 | Q13822 |
| ENPP4 | Ectonucleotide pyrophosphatase/phosphodiesterase 4 | Q9Y6X5 |
| ENPP5 | Ectonucleotide pyrophosphatase/phosphodiesterase family member 5 | Q9UJA9 |
| ENSA | Endosulfine alpha | O43768 |
| ENTPD1 | Ectonucleoside triphosphate diphosphohydrolase 1 | P49961 |
| ENTPD4 | Ectonucleoside triphosphate diphosphohydrolase 4 | Q9Y227 |
| ENTPD5 | Ectonucleoside triphosphate diphosphohydrolase 5 (inactive) | O75356 |
| ENTPD6 | Ectonucleoside triphosphate diphosphohydrolase 6 | O75354 |
| ENY2 | ENY2 transcription and export complex 2 subunit | Q9NPA8 |
| EOGT | EGF domain specific O-linked N-acetylglucosamine transferase | Q5NDL2 |
| EOMES | Eomesodermin | O95936 |
| EP400 | E1A binding protein p400 | Q96L91 |
| EPB41 | Erythrocyte membrane protein band 4.1 | P11171 |
| EPB41L2 | Erythrocyte membrane protein band 4.1 like 2 | O43491 |
| EPB41L4A | Erythrocyte membrane protein band 4.1 like 4A | Q9HCS5 |
| EPC1 | Enhancer of polycomb homolog 1 | Q9H2F5 |
| EPHA1 | EPH receptor A1 | P21709 |
| EPHA4 | EPH receptor A4 | P54764 |
| EPHX2 | Epoxide hydrolase 2 | P34913 |
| EPHX4 | Epoxide hydrolase 4 | Q8IUS5 |
| EPM2AIP1 | EPM2A interacting protein 1 | Q7L775 |
| EPOR | Erythropoietin receptor | P19235 |
| EPS15L1 | Epidermal growth factor receptor pathway substrate 15 like 1 | Q9UBC2 |
| EPSTI1 | Epithelial stromal interaction 1 | Q96J88 |
| ERAL1 | Era like 12S mitochondrial rRNA chaperone 1 | O75616 |
| ERAP1 | Endoplasmic reticulum aminopeptidase 1 | Q9NZ08 |
| ERAP2 | Endoplasmic reticulum aminopeptidase 2 | Q6P179 |
| ERBB2 | Erb-b2 receptor tyrosine kinase 2 | P04626 |
| ERCC1 | ERCC excision repair 1, endonuclease non-catalytic subunit | P07992 |
| ERCC2 | ERCC excision repair 2, TFIIH core complex helicase subunit | P18074 |
| ERCC3 | ERCC excision repair 3, TFIIH core complex helicase subunit | P19447 |
| ERCC4 | ERCC excision repair 4, endonuclease catalytic subunit | Q92889 |
| ERCC5 | ERCC excision repair 5, endonuclease | P28715 |
| ERCC6L2 | ERCC excision repair 6 like 2 | Q5T890 |
| ERCC8 | ERCC excision repair 8, CSA ubiquitin ligase complex subunit | Q13216 |
| ERGIC1 | Endoplasmic reticulum-golgi intermediate compartment 1 | Q969X5 |
| ERGIC2 | ERGIC and golgi 2 | Q96RQ1 |
| ERGIC3 | ERGIC and golgi 3 | Q9Y282 |
| ERH | ERH mRNA splicing and mitosis factor | P84090 |
| ERI1 | Exoribonuclease 1 | Q8IV48 |
| ERI3 | ERI1 exoribonuclease family member 3 | O43414 |
| ERICH1 | Glutamate rich 1 | Q86X53 |
| ERICH6 | Glutamate rich 6 | Q7L0X2 |
| ERLEC1 | Endoplasmic reticulum lectin 1 | Q96DZ1 |
| ERLIN1 | ER lipid raft associated 1 | O75477 |
| ERLIN2 | ER lipid raft associated 2 | O94905 |
| ERMAP | Erythroblast membrane associated protein (Scianna blood group) | Q96PL5 |
| ERN1 | Endoplasmic reticulum to nucleus signaling 1 | O75460 |
| ERO1A | Endoplasmic reticulum oxidoreductase 1 alpha | Q96HE7 |
| ERP27 | Endoplasmic reticulum protein 27 | Q96DN0 |
| ERP29 | Endoplasmic reticulum protein 29 | P30040 |
| ERP44 | Endoplasmic reticulum protein 44 | Q9BS26 |
| ESCO1 | Establishment of sister chromatid cohesion N-acetyltransferase 1 | Q5FWF5 |
| ESD | Esterase D | P10768 |
| ESF1 | ESF1 nucleolar pre-rRNA processing protein homolog | Q9H501 |
| ESYT1 | Extended synaptotagmin 1 | Q9BSJ8 |
| ESYT2 | Extended synaptotagmin 2 | A0FGR8 |
| ETF1 | Eukaryotic translation termination factor 1 | P62495 |
| ETFA | Electron transfer flavoprotein subunit alpha | P13804 |
| ETFB | Electron transfer flavoprotein subunit beta | P38117 |
| ETFDH | Electron transfer flavoprotein dehydrogenase | Q16134 |
| ETHE1 | ETHE1 persulfide dioxygenase | O95571 |
| ETNK1 | Ethanolamine kinase 1 | Q9HBU6 |
| ETNK2 | Ethanolamine kinase 2 | Q9NVF9 |
| ETS1 | ETS proto-oncogene 1, transcription factor | P14921 |
| ETS2 | ETS proto-oncogene 2, transcription factor | P15036 |
| ETV2 | ETS variant transcription factor 2 | O00321 |
| ETV3 | ETS variant transcription factor 3 | P41162 |
| ETV6 | ETS variant transcription factor 6 | P41212 |
| ETV7 | ETS variant transcription factor 7 | Q9Y603 |
| EVA1C | Eva-1 homolog C | P58658 |
| EVI2A | Ecotropic viral integration site 2A | P22794 |
| EVI2B | Ecotropic viral integration site 2B | P34910 |
| EVL | Enah/Vasp-like | Q9UI08 |
| EWSR1 | EWS RNA binding protein 1 | Q01844 |
| EXD2 | Exonuclease 3'-5' domain containing 2 | Q9NVH0 |
| EXD3 | Exonuclease 3'-5' domain containing 3 | Q8N9H8 |
| EXO5 | Exonuclease 5 | Q9H790 |
| EXOC1 | Exocyst complex component 1 | Q9NV70 |
| EXOC3 | Exocyst complex component 3 | O60645 |
| EXOC5 | Exocyst complex component 5 | O00471 |
| EXOC6 | Exocyst complex component 6 | Q8TAG9 |
| EXOC7 | Exocyst complex component 7 | Q9UPT5 |
| EXOG | Exo/endonuclease G | Q9Y2C4 |
| EXOSC1 | Exosome component 1 | Q9Y3B2 |
| EXOSC10 | Exosome component 10 | Q01780 |
| EXOSC2 | Exosome component 2 | Q13868 |
| EXOSC3 | Exosome component 3 | Q9NQT5 |
| EXOSC4 | Exosome component 4 | Q9NPD3 |
| EXOSC5 | Exosome component 5 | Q9NQT4 |
| EXOSC7 | Exosome component 7 | Q15024 |
| EXOSC8 | Exosome component 8 | Q96B26 |
| EXT2 | Exostosin glycosyltransferase 2 | Q93063 |
| EXTL2 | Exostosin like glycosyltransferase 2 | Q9UBQ6 |
| EYA3 | EYA transcriptional coactivator and phosphatase 3 | Q99504 |
| EZH1 | Enhancer of zeste 1 polycomb repressive complex 2 subunit | Q92800 |
| EZH2 | Enhancer of zeste 2 polycomb repressive complex 2 subunit | Q15910 |
| EZR | Ezrin | P15311 |
| F11R | F11 receptor | Q9Y624 |
| F2R | Coagulation factor II thrombin receptor | P25116 |
| F2RL1 | F2R like trypsin receptor 1 | P55085 |
| F8 | Coagulation factor VIII | P00451 |
| F8A1 | Coagulation factor VIII associated 1 | P23610 |
| FAAH2 | Fatty acid amide hydrolase 2 | Q6GMR7 |
| FAAP24 | FA core complex associated protein 24 | Q9BTP7 |
| FABP5 | Fatty acid binding protein 5 | Q01469 |
| FADD | Fas associated via death domain | Q13158 |
| FADS1 | Fatty acid desaturase 1 | O60427 |
| FADS2 | Fatty acid desaturase 2 | O95864 |
| FADS3 | Fatty acid desaturase 3 | Q9Y5Q0 |
| FAF1 | Fas associated factor 1 | Q9UNN5 |
| FAF2 | Fas associated factor family member 2 | Q96CS3 |
| FAH | Fumarylacetoacetate hydrolase | P16930 |
| FAHD1 | Fumarylacetoacetate hydrolase domain containing 1 | Q6P587 |
| FAHD2A | Fumarylacetoacetate hydrolase domain containing 2A | Q96GK7 |
| FAHD2B | Fumarylacetoacetate hydrolase domain containing 2B | Q6P2I3 |
| FAIM | Fas apoptotic inhibitory molecule | Q9NVQ4 |
| FAM102A | Family with sequence similarity 102 member A | Q5T9C2 |
| FAM104B | Family with sequence similarity 104 member B | Q5XKR9 |
| FAM107B | Family with sequence similarity 107 member B | Q9H098 |
| FAM110A | Family with sequence similarity 110 member A | Q9BQ89 |
| FAM111A | FAM111 trypsin like peptidase A | Q96PZ2 |
| FAM114A2 | Family with sequence similarity 114 member A2 | Q9NRY5 |
| FAM117A | Family with sequence similarity 117 member A | Q9C073 |
| FAM118A | Family with sequence similarity 118 member A | Q9NWS6 |
| FAM118B | Family with sequence similarity 118 member B | Q9BPY3 |
| FAM120A | Family with sequence similarity 120A | Q9NZB2 |
| FAM120AOS | Family with sequence similarity 120A opposite strand | Q5T036 |
| FAM120B | Family with sequence similarity 120B | Q96EK7 |
| FAM131A | Family with sequence similarity 131 member A | Q6UXB0 |
| FAM133B | Family with sequence similarity 133 member B | Q5BKY9 |
| FAM136A | Family with sequence similarity 136 member A | Q96C01 |
| FAM13B | Family with sequence similarity 13 member B | Q9NYF5 |
| FAM153A | Family with sequence similarity 153 member A | Q9UHL3 |
| FAM156A | Family with sequence similarity 156 member A | Q8NDB6 |
| FAM161B | FAM161 centrosomal protein B | Q96MY7 |
| FAM162A | Family with sequence similarity 162 member A | Q96A26 |
| FAM168B | Family with sequence similarity 168 member B | A1KXE4 |
| FAM172A | Family with sequence similarity 172 member A | Q8WUF8 |
| FAM174A | Family with sequence similarity 174 member A | Q8TBP5 |
| FAM177A1 | Family with sequence similarity 177 member A1 | Q8N128 |
| FAM200A | Family with sequence similarity 200 member A | Q8TCP9 |
| FAM204A | Family with sequence similarity 204 member A | Q9H8W3 |
| FAM20B | FAM20B glycosaminoglycan xylosylkinase | O75063 |
| FAM210A | Family with sequence similarity 210 member A | Q96ND0 |
| FAM216A | Family with sequence similarity 216 member A | Q8WUB2 |
| FAM217B | Family with sequence similarity 217 member B | Q9NTX9 |
| FAM219B | Family with sequence similarity 219 member B | Q5XKK7 |
| FAM220A | Family with sequence similarity 220 member A | Q7Z4H9 |
| FAM221A | Family with sequence similarity 221 member A | A4D161 |
| FAM222B | Family with sequence similarity 222 member B | Q8WU58 |
| FAM227B | Family with sequence similarity 227 member B | Q96M60 |
| FAM234A | Family with sequence similarity 234 member A | Q9H0X4 |
| FAM234B | Family with sequence similarity 234 member B | A2RU67 |
| FAM24B | Family with sequence similarity 24 member B | Q8N5W8 |
| FAM3A | FAM3 metabolism regulating signaling molecule A | P98173 |
| FAM3C | FAM3 metabolism regulating signaling molecule C | Q92520 |
| FAM50A | Family with sequence similarity 50 member A | Q14320 |
| FAM50B | Family with sequence similarity 50 member B | Q9Y247 |
| FAM53B | Family with sequence similarity 53 member B | Q14153 |
| FAM53C | Family with sequence similarity 53 member C | Q9NYF3 |
| FAM76A | Family with sequence similarity 76 member A | Q8TAV0 |
| FAM76B | Family with sequence similarity 76 member B | Q5HYJ3 |
| FAM78A | Family with sequence similarity 78 member A | Q5JUQ0 |
| FAM89B | Family with sequence similarity 89 member B | Q8N5H3 |
| FAM8A1 | Family with sequence similarity 8 member A1 | Q9UBU6 |
| FAM98A | Family with sequence similarity 98 member A | Q8NCA5 |
| FAM98B | Family with sequence similarity 98 member B | Q52LJ0 |
| FAM98C | Family with sequence similarity 98 member C | Q17RN3 |
| FAN1 | FANCD2 and FANCI associated nuclease 1 | Q9Y2M0 |
| FANCA | FA complementation group A | O15360 |
| FANCB | FA complementation group B | Q8NB91 |
| FANCC | FA complementation group C | Q00597 |
| FANCF | FA complementation group F | Q9NPI8 |
| FANCG | FA complementation group G | O15287 |
| FANCI | FA complementation group I | Q9NVI1 |
| FANCL | FA complementation group L | Q9NW38 |
| FANCM | FA complementation group M | Q8IYD8 |
| FAR1 | Fatty acyl-CoA reductase 1 | Q8WVX9 |
| FARP2 | FERM, ARH/RhoGEF and pleckstrin domain protein 2 | O94887 |
| FARS2 | Phenylalanyl-tRNA synthetase 2, mitochondrial | O95363 |
| FARSA | Phenylalanyl-tRNA synthetase subunit alpha | Q9Y285 |
| FARSB | Phenylalanyl-tRNA synthetase subunit beta | Q9NSD9 |
| FAS | Fas cell surface death receptor | P25445 |
| FASLG | Fas ligand | P48023 |
| FASTK | Fas activated serine/threonine kinase | Q14296 |
| FASTKD2 | FAST kinase domains 2 | Q9NYY8 |
| FASTKD3 | FAST kinase domains 3 | Q14CZ7 |
| FASTKD5 | FAST kinase domains 5 | Q7L8L6 |
| FAU | FAU ubiquitin like and ribosomal protein S30 fusion | P62861 |
| FBL | Fibrillarin | P22087 |
| FBLN2 | Fibulin 2 | P98095 |
| FBLN5 | Fibulin 5 | Q9UBX5 |
| FBLN7 | Fibulin 7 | Q53RD9 |
| FBP1 | Fructose-bisphosphatase 1 | P09467 |
| FBXL12 | F-box and leucine rich repeat protein 12 | Q9NXK8 |
| FBXL15 | F-box and leucine rich repeat protein 15 | Q9H469 |
| FBXL18 | F-box and leucine rich repeat protein 18 | Q96ME1 |
| FBXL20 | F-box and leucine rich repeat protein 20 | Q96IG2 |
| FBXL3 | F-box and leucine rich repeat protein 3 | Q9UKT7 |
| FBXL4 | F-box and leucine rich repeat protein 4 | Q9UKA2 |
| FBXL5 | F-box and leucine rich repeat protein 5 | Q9UKA1 |
| FBXL8 | F-box and leucine rich repeat protein 8 | Q96CD0 |
| FBXO11 | F-box protein 11 | Q86XK2 |
| FBXO2 | F-box protein 2 | Q9UK22 |
| FBXO21 | F-box protein 21 | O94952 |
| FBXO22 | F-box protein 22 | Q8NEZ5 |
| FBXO25 | F-box protein 25 | Q8TCJ0 |
| FBXO28 | F-box protein 28 | Q9NVF7 |
| FBXO3 | F-box protein 3 | Q9UK99 |
| FBXO32 | F-box protein 32 | Q969P5 |
| FBXO33 | F-box protein 33 | Q7Z6M2 |
| FBXO34 | F-box protein 34 | Q9NWN3 |
| FBXO38 | F-box protein 38 | Q6PIJ6 |
| FBXO4 | F-box protein 4 | Q9UKT5 |
| FBXO42 | F-box protein 42 | Q6P3S6 |
| FBXO44 | F-box protein 44 | Q9H4M3 |
| FBXO5 | F-box protein 5 | Q9UKT4 |
| FBXO6 | F-box protein 6 | Q9NRD1 |
| FBXO7 | F-box protein 7 | Q9Y3I1 |
| FBXO8 | F-box protein 8 | Q9NRD0 |
| FBXO9 | F-box protein 9 | Q9UK97 |
| FBXW11 | F-box and WD repeat domain containing 11 | Q9UKB1 |
| FBXW2 | F-box and WD repeat domain containing 2 | Q9UKT8 |
| FBXW4 | F-box and WD repeat domain containing 4 | P57775 |
| FBXW5 | F-box and WD repeat domain containing 5 | Q969U6 |
| FBXW7 | F-box and WD repeat domain containing 7 | Q969H0 |
| FBXW9 | F-box and WD repeat domain containing 9 | Q5XUX1 |
| FCER1A | Fc epsilon receptor Ia | P12319 |
| FCER1G | Fc epsilon receptor Ig | P30273 |
| FCF1 | FCF1 rRNA-processing protein | Q9Y324 |
| FCGR2B | Fc gamma receptor IIb | P31994 |
| FCGR3A | Fc gamma receptor IIIa | P08637 |
| FCGR3B | Fc gamma receptor IIIb | O75015 |
| FCGRT | Fc gamma receptor and transporter | P55899 |
| FCHO1 | FCH and mu domain containing endocytic adaptor 1 | O14526 |
| FCHSD1 | FCH and double SH3 domains 1 | Q86WN1 |
| FCHSD2 | FCH and double SH3 domains 2 | O94868 |
| FCMR | Fc mu receptor | O60667 |
| FCN1 | Ficolin 1 | O00602 |
| FCRL3 | Fc receptor like 3 | Q96P31 |
| FDFT1 | Farnesyl-diphosphate farnesyltransferase 1 | P37268 |
| FDPS | Farnesyl diphosphate synthase | P14324 |
| FDX1 | Ferredoxin 1 | P10109 |
| FDXACB1 | Ferredoxin-fold anticodon binding domain containing 1 | Q9BRP7 |
| FDXR | Ferredoxin reductase | P22570 |
| FECH | Ferrochelatase | P22830 |
| FEM1A | Fem-1 homolog A | Q9BSK4 |
| FEM1B | Fem-1 homolog B | Q9UK73 |
| FEM1C | Fem-1 homolog C | Q96JP0 |
| FEN1 | Flap structure-specific endonuclease 1 | P39748 |
| FERMT3 | FERM domain containing kindlin 3 | Q86UX7 |
| FEZ1 | Fasciculation and elongation protein zeta 1 | Q99689 |
| FGD3 | FYVE, RhoGEF and PH domain containing 3 | Q5JSP0 |
| FGF23 | Fibroblast growth factor 23 | Q9GZV9 |
| FGFBP2 | Fibroblast growth factor binding protein 2 | Q9BYJ0 |
| FGFR1 | Fibroblast growth factor receptor 1 | P11362 |
| FGFR1OP2 | FGFR1 oncogene partner 2 | Q9NVK5 |
| FGGY | FGGY carbohydrate kinase domain containing | Q96C11 |
| FGL2 | Fibrinogen like 2 | Q14314 |
| FGR | FGR proto-oncogene, Src family tyrosine kinase | P09769 |
| FHIT | Fragile histidine triad diadenosine triphosphatase | P49789 |
| FHL1 | Four and a half LIM domains 1 | Q13642 |
| FHL2 | Four and a half LIM domains 2 | Q14192 |
| FHL3 | Four and a half LIM domains 3 | Q13643 |
| FIBP | FGF1 intracellular binding protein | O43427 |
| FICD | FIC domain protein adenylyltransferase | Q9BVA6 |
| FIG4 | FIG4 phosphoinositide 5-phosphatase | Q92562 |
| FIGNL1 | Fidgetin like 1 | Q6PIW4 |
| FIP1L1 | Factor interacting with PAPOLA and CPSF1 | Q6UN15 |
| FIS1 | Fission, mitochondrial 1 | Q9Y3D6 |
| FITM2 | Fat storage inducing transmembrane protein 2 | Q8N6M3 |
| FKBP11 | FKBP prolyl isomerase 11 | Q9NYL4 |
| FKBP1A | FKBP prolyl isomerase 1A | P62942 |
| FKBP2 | FKBP prolyl isomerase 2 | P26885 |
| FKBP3 | FKBP prolyl isomerase 3 | Q00688 |
| FKBP4 | FKBP prolyl isomerase 4 | Q02790 |
| FKBP5 | FKBP prolyl isomerase 5 | Q13451 |
| FKBP8 | FKBP prolyl isomerase 8 | Q14318 |
| FKBPL | FKBP prolyl isomerase like | Q9UIM3 |
| FKRP | Fukutin related protein | Q9H9S5 |
| FLAD1 | Flavin adenine dinucleotide synthetase 1 | Q8NFF5 |
| FLCN | Folliculin | Q8NFG4 |
| FLI1 | Fli-1 proto-oncogene, ETS transcription factor | Q01543 |
| FLII | FLII actin remodeling protein | Q13045 |
| FLOT1 | Flotillin 1 | O75955 |
| FLOT2 | Flotillin 2 | Q14254 |
| FLT3LG | Fms related receptor tyrosine kinase 3 ligand | P49771 |
| FLYWCH1 | FLYWCH-type zinc finger 1 | Q4VC44 |
| FLYWCH2 | FLYWCH family member 2 | Q96CP2 |
| FMO4 | Flavin containing dimethylaniline monoxygenase 4 | P31512 |
| FMO5 | Flavin containing dimethylaniline monoxygenase 5 | P49326 |
| FMR1 | Fragile X messenger ribonucleoprotein 1 | Q06787 |
| FN3KRP | Fructosamine 3 kinase related protein | Q9HA64 |
| FNBP4 | Formin binding protein 4 | Q8N3X1 |
| FNDC3A | Fibronectin type III domain containing 3A | Q9Y2H6 |
| FNDC3B | Fibronectin type III domain containing 3B | Q53EP0 |
| FNIP1 | Folliculin interacting protein 1 | Q8TF40 |
| FNTA | Farnesyltransferase, CAAX box, alpha | P49354 |
| FNTB | Farnesyltransferase, CAAX box, beta | P49356 |
| FOSL2 | FOS like 2, AP-1 transcription factor subunit | P15408 |
| FOXJ2 | Forkhead box J2 | Q9P0K8 |
| FOXJ3 | Forkhead box J3 | Q9UPW0 |
| FOXK1 | Forkhead box K1 | P85037 |
| FOXK2 | Forkhead box K2 | Q01167 |
| FOXN2 | Forkhead box N2 | P32314 |
| FOXN3 | Forkhead box N3 | O00409 |
| FOXO1 | Forkhead box O1 | Q12778 |
| FOXO3 | Forkhead box O3 | O43524 |
| FOXP1 | Forkhead box P1 | Q9H334 |
| FOXRED1 | FAD dependent oxidoreductase domain containing 1 | Q96CU9 |
| FPGS | Folylpolyglutamate synthase | Q05932 |
| FPGT | Fucose-1-phosphate guanylyltransferase | O14772 |
| FPR1 | Formyl peptide receptor 1 | P21462 |
| FRA10AC1 | FRA10A associated CGG repeat 1 | Q70Z53 |
| FRAT2 | FRAT regulator of WNT signaling pathway 2 | O75474 |
| FRG1 | FSHD region gene 1 | Q14331 |
| FRMD4B | FERM domain containing 4B | Q9Y2L6 |
| FRMD8 | FERM domain containing 8 | Q9BZ67 |
| FRS2 | Fibroblast growth factor receptor substrate 2 | Q8WU20 |
| FRYL | FRY like transcription coactivator | O94915 |
| FTCDNL1 | Formiminotransferase cyclodeaminase N-terminal like | E5RQL4 |
| FTH1 | Ferritin heavy chain 1 | P02794 |
| FTL | Ferritin light chain | P02792 |
| FTSJ1 | FtsJ RNA 2'-O-methyltransferase 1 | Q9UET6 |
| FTSJ3 | FtsJ RNA 2'-O-methyltransferase 3 | Q8IY81 |
| FUBP1 | Far upstream element binding protein 1 | Q96AE4 |
| FUBP3 | Far upstream element binding protein 3 | Q96I24 |
| FUCA1 | Alpha-L-fucosidase 1 | P04066 |
| FUCA2 | Alpha-L-fucosidase 2 | Q9BTY2 |
| FUNDC1 | FUN14 domain containing 1 | Q8IVP5 |
| FUNDC2 | FUN14 domain containing 2 | Q9BWH2 |
| FUOM | Fucose mutarotase | A2VDF0 |
| FUS | FUS RNA binding protein | P35637 |
| FUT10 | Fucosyltransferase 10 | Q6P4F1 |
| FUT11 | Fucosyltransferase 11 | Q495W5 |
| FUT7 | Fucosyltransferase 7 | Q11130 |
| FUT8 | Fucosyltransferase 8 | Q9BYC5 |
| FUZ | Fuzzy planar cell polarity protein | Q9BT04 |
| FXN | Frataxin | Q16595 |
| FXR1 | FMR1 autosomal homolog 1 | P51114 |
| FXR2 | FMR1 autosomal homolog 2 | P51116 |
| FXYD1 | FXYD domain containing ion transport regulator 1 | O00168 |
| FXYD2 | FXYD domain containing ion transport regulator 2 | P54710 |
| FXYD5 | FXYD domain containing ion transport regulator 5 | Q96DB9 |
| FXYD7 | FXYD domain containing ion transport regulator 7 | P58549 |
| FYCO1 | FYVE and coiled-coil domain autophagy adaptor 1 | Q9BQS8 |
| FYN | FYN proto-oncogene, Src family tyrosine kinase | P06241 |
| FYTTD1 | Forty-two-three domain containing 1 | Q96QD9 |
| FZD3 | Frizzled class receptor 3 | Q9NPG1 |
| G2E3 | G2/M-phase specific E3 ubiquitin protein ligase | Q7L622 |
| G3BP1 | G3BP stress granule assembly factor 1 | Q13283 |
| G3BP2 | G3BP stress granule assembly factor 2 | Q9UN86 |
| G6PC3 | Glucose-6-phosphatase catalytic subunit 3 | Q9BUM1 |
| G6PD | Glucose-6-phosphate dehydrogenase | P11413 |
| GAA | Alpha glucosidase | P10253 |
| GAB3 | GRB2 associated binding protein 3 | Q8WWW8 |
| GABARAP | GABA type A receptor-associated protein | O95166 |
| GABARAPL1 | GABA type A receptor associated protein like 1 | Q9H0R8 |
| GABARAPL2 | GABA type A receptor associated protein like 2 | P60520 |
| GABPA | GA binding protein transcription factor subunit alpha | Q06546 |
| GABPB1 | GA binding protein transcription factor subunit beta 1 | Q06547 |
| GABPB2 | GA binding protein transcription factor subunit beta 2 | Q8TAK5 |
| GADD45A | Growth arrest and DNA damage inducible alpha | P24522 |
| GADD45B | Growth arrest and DNA damage inducible beta | O75293 |
| GADD45GIP1 | GADD45G interacting protein 1 | Q8TAE8 |
| GAK | Cyclin G associated kinase | O14976 |
| GAL3ST4 | Galactose-3-O-sulfotransferase 4 | Q96RP7 |
| GALC | Galactosylceramidase | P54803 |
| GALE | UDP-galactose-4-epimerase | Q14376 |
| GALK1 | Galactokinase 1 | P51570 |
| GALK2 | Galactokinase 2 | Q01415 |
| GALM | Galactose mutarotase | Q96C23 |
| GALNS | Galactosamine (N-acetyl)-6-sulfatase | P34059 |
| GALNT1 | Polypeptide N-acetylgalactosaminyltransferase 1 | Q10472 |
| GALNT10 | Polypeptide N-acetylgalactosaminyltransferase 10 | Q86SR1 |
| GALNT2 | Polypeptide N-acetylgalactosaminyltransferase 2 | Q10471 |
| GALNT3 | Polypeptide N-acetylgalactosaminyltransferase 3 | Q14435 |
| GALNT4 | Polypeptide N-acetylgalactosaminyltransferase 4 | Q8N4A0 |
| GALNT6 | Polypeptide N-acetylgalactosaminyltransferase 6 | Q8NCL4 |
| GALNT7 | Polypeptide N-acetylgalactosaminyltransferase 7 | Q86SF2 |
| GALT | Galactose-1-phosphate uridylyltransferase | P07902 |
| GAMT | Guanidinoacetate N-methyltransferase | Q14353 |
| GANAB | Glucosidase II alpha subunit | Q14697 |
| GANC | Glucosidase alpha, neutral C | Q8TET4 |
| GAPDH | Glyceraldehyde-3-phosphate dehydrogenase | P04406 |
| GAPVD1 | GTPase activating protein and VPS9 domains 1 | Q14C86 |
| GAR1 | GAR1 ribonucleoprotein | Q9NY12 |
| GART | Phosphoribosylglycinamide formyltransferase, phosphoribosylglycinamide synthetase, phosphoribosylaminoimidazole synthetase | P22102 |
| GAS7 | Growth arrest specific 7 | O60861 |
| GATA3 | GATA binding protein 3 | P23771 |
| GATC | Glutamyl-tRNA amidotransferase subunit C | O43716 |
| GATM | Glycine amidinotransferase | P50440 |
| GBA2 | Glucosylceramidase beta 2 | Q9HCG7 |
| GBE1 | 1,4-alpha-glucan branching enzyme 1 | Q04446 |
| GBP1 | Guanylate binding protein 1 | P32455 |
| GBP2 | Guanylate binding protein 2 | P32456 |
| GBP3 | Guanylate binding protein 3 | Q9H0R5 |
| GBP4 | Guanylate binding protein 4 | Q96PP9 |
| GBP5 | Guanylate binding protein 5 | Q96PP8 |
| GCA | Grancalcin | P28676 |
| GCAT | Glycine C-acetyltransferase | O75600 |
| GCC1 | GRIP and coiled-coil domain containing 1 | Q96CN9 |
| GCDH | Glutaryl-CoA dehydrogenase | Q92947 |
| GCFC2 | GC-rich sequence DNA-binding factor 2 | P16383 |
| GCH1 | GTP cyclohydrolase 1 | P30793 |
| GCHFR | GTP cyclohydrolase I feedback regulator | P30047 |
| GCLC | Glutamate-cysteine ligase catalytic subunit | P48506 |
| GCLM | Glutamate-cysteine ligase modifier subunit | P48507 |
| GCNT1 | Glucosaminyl (N-acetyl) transferase 1 | Q02742 |
| GCNT4 | Glucosaminyl (N-acetyl) transferase 4 | Q9P109 |
| GCSAM | Germinal center associated signaling and motility | Q8N6F7 |
| GCSH | Glycine cleavage system protein H | P23434 |
| GDAP1 | Ganglioside induced differentiation associated protein 1 | Q8TB36 |
| GDAP2 | Ganglioside induced differentiation associated protein 2 | Q9NXN4 |
| GDE1 | Glycerophosphodiester phosphodiesterase 1 | Q9NZC3 |
| GDI1 | GDP dissociation inhibitor 1 | P31150 |
| GDI2 | GDP dissociation inhibitor 2 | P50395 |
| GDPGP1 | GDP-D-glucose phosphorylase 1 | Q6ZNW5 |
| GEMIN2 | Gem nuclear organelle associated protein 2 | O14893 |
| GEMIN4 | Gem nuclear organelle associated protein 4 | P57678 |
| GEMIN6 | Gem nuclear organelle associated protein 6 | Q8WXD5 |
| GEMIN7 | Gem nuclear organelle associated protein 7 | Q9H840 |
| GEMIN8 | Gem nuclear organelle associated protein 8 | Q9NWZ8 |
| GET4 | Guided entry of tail-anchored proteins factor 4 | Q7L5D6 |
| GFER | Growth factor, augmenter of liver regeneration | P55789 |
| GFI1 | Growth factor independent 1 transcriptional repressor | Q99684 |
| GFM1 | G elongation factor mitochondrial 1 | Q96RP9 |
| GFM2 | GTP dependent ribosome recycling factor mitochondrial 2 | Q969S9 |
| GFOD2 | Glucose-fructose oxidoreductase domain containing 2 | Q3B7J2 |
| GFPT1 | Glutamine--fructose-6-phosphate transaminase 1 | Q06210 |
| GFPT2 | Glutamine-fructose-6-phosphate transaminase 2 | O94808 |
| GGA1 | Golgi associated, gamma adaptin ear containing, ARF binding protein 1 | Q9UJY5 |
| GGA2 | Golgi associated, gamma adaptin ear containing, ARF binding protein 2 | Q9UJY4 |
| GGA3 | Golgi associated, gamma adaptin ear containing, ARF binding protein 3 | Q9NZ52 |
| GGACT | Gamma-glutamylamine cyclotransferase | Q9BVM4 |
| GGCT | Gamma-glutamylcyclotransferase | O75223 |
| GGCX | Gamma-glutamyl carboxylase | P38435 |
| GGH | Gamma-glutamyl hydrolase | Q92820 |
| GGPS1 | Geranylgeranyl diphosphate synthase 1 | O95749 |
| GGT1 | Gamma-glutamyltransferase 1 | P19440 |
| GGT7 | Gamma-glutamyltransferase 7 | Q9UJ14 |
| GGTLC2 | Gamma-glutamyltransferase light chain 2 | Q14390 |
| GHDC | GH3 domain containing | Q8N2G8 |
| GHITM | Growth hormone inducible transmembrane protein | Q9H3K2 |
| GID8 | GID complex subunit 8 homolog | Q9NWU2 |
| GIGYF1 | GRB10 interacting GYF protein 1 | O75420 |
| GIGYF2 | GRB10 interacting GYF protein 2 | Q6Y7W6 |
| GIMAP1 | GTPase, IMAP family member 1 | Q8WWP7 |
| GIMAP2 | GTPase, IMAP family member 2 | Q9UG22 |
| GIMAP4 | GTPase, IMAP family member 4 | Q9NUV9 |
| GIMAP5 | GTPase, IMAP family member 5 | Q96F15 |
| GIMAP6 | GTPase, IMAP family member 6 | Q6P9H5 |
| GIMAP7 | GTPase, IMAP family member 7 | Q8NHV1 |
| GIMAP8 | GTPase, IMAP family member 8 | Q8ND71 |
| GIN1 | Gypsy retrotransposon integrase 1 | Q9NXP7 |
| GINM1 | Glycosylated integral membrane protein 1 | Q9NU53 |
| GINS2 | GINS complex subunit 2 | Q9Y248 |
| GINS3 | GINS complex subunit 3 | Q9BRX5 |
| GINS4 | GINS complex subunit 4 | Q9BRT9 |
| GIPC1 | GIPC PDZ domain containing family member 1 | O14908 |
| GIT1 | GIT ArfGAP 1 | Q9Y2X7 |
| GIT2 | GIT ArfGAP 2 | Q14161 |
| GK | Glycerol kinase | P32189 |
| GLA | Galactosidase alpha | P06280 |
| GLB1 | Galactosidase beta 1 | P16278 |
| GLB1L2 | Galactosidase beta 1 like 2 | Q8IW92 |
| GLB1L3 | Galactosidase beta 1 like 3 | Q8NCI6 |
| GLE1 | GLE1 RNA export mediator | Q53GS7 |
| GLIPR1 | GLI pathogenesis related 1 | P48060 |
| GLIPR2 | GLI pathogenesis related 2 | Q9H4G4 |
| GLMN | Glomulin, FKBP associated protein | Q92990 |
| GLMP | Glycosylated lysosomal membrane protein | Q8WWB7 |
| GLO1 | Glyoxalase I | Q04760 |
| GLOD4 | Glyoxalase domain containing 4 | Q9HC38 |
| GLRX | Glutaredoxin | P35754 |
| GLRX2 | Glutaredoxin 2 | Q9NS18 |
| GLRX3 | Glutaredoxin 3 | O76003 |
| GLRX5 | Glutaredoxin 5 | Q86SX6 |
| GLS2 | Glutaminase 2 | Q9UI32 |
| GLT8D1 | Glycosyltransferase 8 domain containing 1 | Q68CQ7 |
| GLTP | Glycolipid transfer protein | Q9NZD2 |
| GLUD1 | Glutamate dehydrogenase 1 | P00367 |
| GLUL | Glutamate-ammonia ligase | P15104 |
| GLYCTK | Glycerate kinase | Q8IVS8 |
| GLYR1 | Glyoxylate reductase 1 homolog | Q49A26 |
| GM2A | Ganglioside GM2 activator | P17900 |
| GMCL1 | Germ cell-less 1, spermatogenesis associated | Q96IK5 |
| GMDS | GDP-mannose 4,6-dehydratase | O60547 |
| GMEB1 | Glucocorticoid modulatory element binding protein 1 | Q9Y692 |
| GMEB2 | Glucocorticoid modulatory element binding protein 2 | Q9UKD1 |
| GMFB | Glia maturation factor beta | P60983 |
| GMFG | Glia maturation factor gamma | O60234 |
| GMIP | GEM interacting protein | Q9P107 |
| GMNN | Geminin DNA replication inhibitor | O75496 |
| GMPPA | GDP-mannose pyrophosphorylase A | Q96IJ6 |
| GMPPB | GDP-mannose pyrophosphorylase B | Q9Y5P6 |
| GMPR2 | Guanosine monophosphate reductase 2 | Q9P2T1 |
| GMPS | Guanine monophosphate synthase | P49915 |
| GNA13 | G protein subunit alpha 13 | Q14344 |
| GNAI2 | G protein subunit alpha i2 | P04899 |
| GNAI3 | G protein subunit alpha i3 | P08754 |
| GNAQ | G protein subunit alpha q | P50148 |
| GNAS | GNAS complex locus | O95467, P63092, Q5JWF2 |
| GNB1 | G protein subunit beta 1 | P62873 |
| GNB1L | G protein subunit beta 1 like | Q9BYB4 |
| GNB2 | G protein subunit beta 2 | P62879 |
| GNB5 | G protein subunit beta 5 | O14775 |
| GNE | Glucosamine (UDP-N-acetyl)-2-epimerase/N-acetylmannosamine kinase | Q9Y223 |
| GNG10 | G protein subunit gamma 10 | P50151 |
| GNG2 | G protein subunit gamma 2 | P59768 |
| GNG5 | G protein subunit gamma 5 | P63218 |
| GNGT2 | G protein subunit gamma transducin 2 | O14610 |
| GNL1 | G protein nucleolar 1 (putative) | P36915 |
| GNL2 | G protein nucleolar 2 | Q13823 |
| GNL3 | G protein nucleolar 3 | Q9BVP2 |
| GNL3L | G protein nucleolar 3 like | Q9NVN8 |
| GNLY | Granulysin | P22749 |
| GNPAT | Glyceronephosphate O-acyltransferase | O15228 |
| GNPDA1 | Glucosamine-6-phosphate deaminase 1 | P46926 |
| GNPDA2 | Glucosamine-6-phosphate deaminase 2 | Q8TDQ7 |
| GNPTAB | N-acetylglucosamine-1-phosphate transferase subunits alpha and beta | Q3T906 |
| GNPTG | N-acetylglucosamine-1-phosphate transferase subunit gamma | Q9UJJ9 |
| GNS | Glucosamine (N-acetyl)-6-sulfatase | P15586 |
| GOLGA1 | Golgin A1 | Q92805 |
| GOLGA2 | Golgin A2 | Q08379 |
| GOLGA4 | Golgin A4 | Q13439 |
| GOLGA5 | Golgin A5 | Q8TBA6 |
| GOLGA7 | Golgin A7 | Q7Z5G4 |
| GOLPH3 | Golgi phosphoprotein 3 | Q9H4A6 |
| GOLPH3L | Golgi phosphoprotein 3 like | Q9H4A5 |
| GOLT1B | Golgi transport 1B | Q9Y3E0 |
| GON4L | Gon-4 like | Q3T8J9 |
| GOPC | Golgi associated PDZ and coiled-coil motif containing | Q9HD26 |
| GORAB | Golgin, RAB6 interacting | Q5T7V8 |
| GORASP1 | Golgi reassembly stacking protein 1 | Q9BQQ3 |
| GORASP2 | Golgi reassembly stacking protein 2 | Q9H8Y8 |
| GOSR1 | Golgi SNAP receptor complex member 1 | O95249 |
| GOSR2 | Golgi SNAP receptor complex member 2 | O14653 |
| GOT1 | Glutamic-oxaloacetic transaminase 1 | P17174 |
| GOT2 | Glutamic-oxaloacetic transaminase 2 | P00505 |
| GPA33 | Glycoprotein A33 | Q99795 |
| GPAA1 | Glycosylphosphatidylinositol anchor attachment 1 | O43292 |
| GPANK1 | G-patch domain and ankyrin repeats 1 | O95872 |
| GPAT4 | Glycerol-3-phosphate acyltransferase 4 | Q86UL3 |
| GPATCH1 | G-patch domain containing 1 | Q9BRR8 |
| GPATCH11 | G-patch domain containing 11 |  |
| GPATCH2 | G-patch domain containing 2 | Q9NW75 |
| GPATCH2L | G-patch domain containing 2 like | Q9NWQ4 |
| GPATCH3 | G-patch domain containing 3 | Q96I76 |
| GPATCH4 | G-patch domain containing 4 (gene/pseudogene) |  |
| GPBP1 | GC-rich promoter binding protein 1 | Q86WP2 |
| GPBP1L1 | GC-rich promoter binding protein 1 like 1 | Q9HC44 |
| GPC3 | Glypican 3 | P51654 |
| GPCPD1 | Glycerophosphocholine phosphodiesterase 1 | Q9NPB8 |
| GPD1L | Glycerol-3-phosphate dehydrogenase 1 like | Q8N335 |
| GPHN | Gephyrin | Q9NQX3 |
| GPI | Glucose-6-phosphate isomerase | P06744 |
| GPKOW | G-patch domain and KOW motifs | Q92917 |
| GPN1 | GPN-loop GTPase 1 | Q9HCN4 |
| GPN2 | GPN-loop GTPase 2 | Q9H9Y4 |
| GPN3 | GPN-loop GTPase 3 | Q9UHW5 |
| GPR107 | G protein-coupled receptor 107 | Q5VW38 |
| GPR108 | G protein-coupled receptor 108 | Q9NPR9 |
| GPR132 | G protein-coupled receptor 132 | Q9UNW8 |
| GPR137 | G protein-coupled receptor 137 | Q96N19 |
| GPR137C | G protein-coupled receptor 137C | Q8N3F9 |
| GPR141 | G protein-coupled receptor 141 | Q7Z602 |
| GPR146 | G protein-coupled receptor 146 | Q96CH1 |
| GPR15 | G protein-coupled receptor 15 | P49685 |
| GPR155 | G protein-coupled receptor 155 | Q7Z3F1 |
| GPR160 | G protein-coupled receptor 160 | Q9UJ42 |
| GPR171 | G protein-coupled receptor 171 | O14626 |
| GPR18 | G protein-coupled receptor 18 | Q14330 |
| GPR183 | G protein-coupled receptor 183 | P32249 |
| GPR65 | G protein-coupled receptor 65 | Q8IYL9 |
| GPR68 | G protein-coupled receptor 68 | Q15743 |
| GPR89A | G protein-coupled receptor 89A | B7ZAQ6 |
| GPRIN3 | GPRIN family member 3 | Q6ZVF9 |
| GPS1 | G protein pathway suppressor 1 | Q13098 |
| GPS2 | G protein pathway suppressor 2 | Q13227 |
| GPSM3 | G protein signaling modulator 3 | Q9Y4H4 |
| GPX7 | Glutathione peroxidase 7 | Q96SL4 |
| GRAMD1A | GRAM domain containing 1A | Q96CP6 |
| GRAMD1B | GRAM domain containing 1B | Q3KR37 |
| GRAP2 | GRB2 related adaptor protein 2 | O75791 |
| GRB2 | Growth factor receptor bound protein 2 | P62993 |
| GRHPR | Glyoxylate and hydroxypyruvate reductase | Q9UBQ7 |
| GRINA | Glutamate ionotropic receptor NMDA type subunit associated protein 1 | Q7Z429 |
| GRIPAP1 | GRIP1 associated protein 1 | Q4V328 |
| GRK5 | G protein-coupled receptor kinase 5 | P34947 |
| GRK6 | G protein-coupled receptor kinase 6 | P43250 |
| GRN | Granulin precursor | P28799 |
| GRPEL1 | GrpE like 1, mitochondrial | Q9HAV7 |
| GRPEL2 | GrpE like 2, mitochondrial | Q8TAA5 |
| GRWD1 | Glutamate rich WD repeat containing 1 | Q9BQ67 |
| GSDMD | Gasdermin D | P57764 |
| GSK3B | Glycogen synthase kinase 3 beta | P49841 |
| GSKIP | GSK3B interacting protein | Q9P0R6 |
| GSN | Gelsolin | P06396 |
| GSPT1 | G1 to S phase transition 1 | P15170 |
| GSPT2 | G1 to S phase transition 2 | Q8IYD1 |
| GSR | Glutathione-disulfide reductase | P00390 |
| GSS | Glutathione synthetase | P48637 |
| GSTA4 | Glutathione S-transferase alpha 4 | O15217 |
| GSTCD | Glutathione S-transferase C-terminal domain containing | Q8NEC7 |
| GSTK1 | Glutathione S-transferase kappa 1 | Q9Y2Q3 |
| GSTM1 | Glutathione S-transferase mu 1 | P09488 |
| GSTM2 | Glutathione S-transferase mu 2 | P28161 |
| GSTM3 | Glutathione S-transferase mu 3 | P21266 |
| GSTM4 | Glutathione S-transferase mu 4 | Q03013 |
| GSTO1 | Glutathione S-transferase omega 1 | P78417 |
| GSTP1 | Glutathione S-transferase pi 1 | P09211 |
| GSTZ1 | Glutathione S-transferase zeta 1 | O43708 |
| GTDC1 | Glycosyltransferase like domain containing 1 | Q4AE62 |
| GTF2A1 | General transcription factor IIA subunit 1 | P52655 |
| GTF2A2 | General transcription factor IIA subunit 2 | P52657 |
| GTF2B | General transcription factor IIB | Q00403 |
| GTF2E1 | General transcription factor IIE subunit 1 | P29083 |
| GTF2E2 | General transcription factor IIE subunit 2 | P29084 |
| GTF2F1 | General transcription factor IIF subunit 1 | P35269 |
| GTF2F2 | General transcription factor IIF subunit 2 | P13984 |
| GTF2H1 | General transcription factor IIH subunit 1 | P32780 |
| GTF2H2 | General transcription factor IIH subunit 2 | Q13888 |
| GTF2H2C | GTF2H2 family member C | Q6P1K8 |
| GTF2H3 | General transcription factor IIH subunit 3 | Q13889 |
| GTF2H4 | General transcription factor IIH subunit 4 | Q92759 |
| GTF2H5 | General transcription factor IIH subunit 5 | Q6ZYL4 |
| GTF2I | General transcription factor IIi | P78347 |
| GTF2IRD2 | GTF2I repeat domain containing 2 | Q86UP8 |
| GTF3C2 | General transcription factor IIIC subunit 2 | Q8WUA4 |
| GTF3C3 | General transcription factor IIIC subunit 3 | Q9Y5Q9 |
| GTF3C5 | General transcription factor IIIC subunit 5 | Q9Y5Q8 |
| GTF3C6 | General transcription factor IIIC subunit 6 | Q969F1 |
| GTPBP1 | GTP binding protein 1 | O00178 |
| GTPBP2 | GTP binding protein 2 | Q9BX10 |
| GTPBP3 | GTP binding protein 3, mitochondrial | Q969Y2 |
| GTPBP4 | GTP binding protein 4 | Q9BZE4 |
| GTPBP6 | GTP binding protein 6 (putative) | O43824 |
| GTPBP8 | GTP binding protein 8 (putative) | Q8N3Z3 |
| GTSF1 | Gametocyte specific factor 1 | Q8WW33 |
| GUCD1 | Guanylyl cyclase domain containing 1 | Q96NT3 |
| GUF1 | GTP binding elongation factor GUF1 | Q8N442 |
| GUK1 | Guanylate kinase 1 | Q16774 |
| GUSB | Glucuronidase beta | P08236 |
| GYG1 | Glycogenin 1 | P46976 |
| GYPC | Glycophorin C (Gerbich blood group) | P04921 |
| GYS1 | Glycogen synthase 1 | P13807 |
| GZMA | Granzyme A | P12544 |
| GZMB | Granzyme B | P10144 |
| GZMH | Granzyme H | P20718 |
| GZMK | Granzyme K | P49863 |
| GZMM | Granzyme M | P51124 |
| H6PD | Hexose-6-phosphate dehydrogenase/glucose 1-dehydrogenase | O95479 |
| HABP4 | Hyaluronan binding protein 4 | Q5JVS0 |
| HACD1 | 3-hydroxyacyl-CoA dehydratase 1 | B0YJ81 |
| HACD2 | 3-hydroxyacyl-CoA dehydratase 2 | Q6Y1H2 |
| HACD3 | 3-hydroxyacyl-CoA dehydratase 3 | Q9P035 |
| HACD4 | 3-hydroxyacyl-CoA dehydratase 4 | Q5VWC8 |
| HACE1 | HECT domain and ankyrin repeat containing E3 ubiquitin protein ligase 1 | Q8IYU2 |
| HACL1 | 2-hydroxyacyl-CoA lyase 1 | Q9UJ83 |
| HADH | Hydroxyacyl-CoA dehydrogenase | Q16836 |
| HADHA | Hydroxyacyl-CoA dehydrogenase trifunctional multienzyme complex subunit alpha | P40939 |
| HADHB | Hydroxyacyl-CoA dehydrogenase trifunctional multienzyme complex subunit beta | P55084 |
| HAGH | Hydroxyacylglutathione hydrolase | Q16775 |
| HAGHL | Hydroxyacylglutathione hydrolase like | Q6PII5 |
| HAPLN3 | Hyaluronan and proteoglycan link protein 3 | Q96S86 |
| HARBI1 | Harbinger transposase derived 1 | Q96MB7 |
| HARS2 | Histidyl-tRNA synthetase 2, mitochondrial | P49590 |
| HAT1 | Histone acetyltransferase 1 | O14929 |
| HAUS1 | HAUS augmin like complex subunit 1 | Q96CS2 |
| HAUS2 | HAUS augmin like complex subunit 2 | Q9NVX0 |
| HAUS3 | HAUS augmin like complex subunit 3 | Q68CZ6 |
| HAUS4 | HAUS augmin like complex subunit 4 | Q9H6D7 |
| HAUS5 | HAUS augmin like complex subunit 5 | O94927 |
| HAUS6 | HAUS augmin like complex subunit 6 | Q7Z4H7 |
| HAUS7 | HAUS augmin like complex subunit 7 | Q99871 |
| HAUS8 | HAUS augmin like complex subunit 8 | Q9BT25 |
| HAVCR1 | Hepatitis A virus cellular receptor 1 | Q96D42 |
| HAVCR2 | Hepatitis A virus cellular receptor 2 | Q8TDQ0 |
| HAX1 | HCLS1 associated protein X-1 | O00165 |
| HBA1 | Hemoglobin subunit alpha 1 | P69905 |
| HBB | Hemoglobin subunit beta | P68871 |
| HBP1 | HMG-box transcription factor 1 | O60381 |
| HBS1L | HBS1 like translational GTPase | Q9Y450 |
| HCCS | Holocytochrome c synthase | P53701 |
| HCFC1 | Host cell factor C1 | P51610 |
| HCFC1R1 | Host cell factor C1 regulator 1 | Q9NWW0 |
| HCK | HCK proto-oncogene, Src family tyrosine kinase | P08631 |
| HCLS1 | Hematopoietic cell-specific Lyn substrate 1 | P14317 |
| HCST | Hematopoietic cell signal transducer | Q9UBK5 |
| HDAC1 | Histone deacetylase 1 | Q13547 |
| HDAC11 | Histone deacetylase 11 | Q96DB2 |
| HDAC3 | Histone deacetylase 3 | O15379 |
| HDAC6 | Histone deacetylase 6 | Q9UBN7 |
| HDAC8 | Histone deacetylase 8 | Q9BY41 |
| HDAC9 | Histone deacetylase 9 | Q9UKV0 |
| HDDC2 | HD domain containing 2 | Q7Z4H3 |
| HDDC3 | HD domain containing 3 | Q8N4P3 |
| HDGF | Heparin binding growth factor | P51858 |
| HDHD2 | Haloacid dehalogenase like hydrolase domain containing 2 | Q9H0R4 |
| HDHD3 | Haloacid dehalogenase like hydrolase domain containing 3 | Q9BSH5 |
| HDLBP | High density lipoprotein binding protein | Q00341 |
| HEATR1 | HEAT repeat containing 1 | Q9H583 |
| HEATR5A | HEAT repeat containing 5A | Q86XA9 |
| HEBP1 | Heme binding protein 1 | Q9NRV9 |
| HEBP2 | Heme binding protein 2 | Q9Y5Z4 |
| HECTD3 | HECT domain E3 ubiquitin protein ligase 3 | Q5T447 |
| HELB | DNA helicase B | Q8NG08 |
| HELLS | Helicase, lymphoid specific | Q9NRZ9 |
| HEMGN | Hemogen | Q9BXL5 |
| HEMK1 | HemK methyltransferase family member 1 | Q9Y5R4 |
| HENMT1 | HEN methyltransferase 1 | Q5T8I9 |
| HERC3 | HECT and RLD domain containing E3 ubiquitin protein ligase 3 | Q15034 |
| HERPUD1 | Homocysteine inducible ER protein with ubiquitin like domain 1 | Q15011 |
| HERPUD2 | HERPUD family member 2 | Q9BSE4 |
| HEXA | Hexosaminidase subunit alpha | P06865 |
| HEXB | Hexosaminidase subunit beta | P07686 |
| HEXIM1 | HEXIM P-TEFb complex subunit 1 | O94992 |
| HEXIM2 | HEXIM P-TEFb complex subunit 2 | Q96MH2 |
| HFE | Homeostatic iron regulator | Q30201 |
| HGS | Hepatocyte growth factor-regulated tyrosine kinase substrate | O14964 |
| HGSNAT | Heparan-alpha-glucosaminide N-acetyltransferase | Q68CP4 |
| HIBADH | 3-hydroxyisobutyrate dehydrogenase | P31937 |
| HIBCH | 3-hydroxyisobutyryl-CoA hydrolase | Q6NVY1 |
| HID1 | HID1 domain containing | Q8IV36 |
| HIF1A | Hypoxia inducible factor 1 subunit alpha | Q16665 |
| HIF1AN | Hypoxia inducible factor 1 subunit alpha inhibitor | Q9NWT6 |
| HIGD1A | HIG1 hypoxia inducible domain family member 1A | Q9Y241 |
| HIGD2A | HIG1 hypoxia inducible domain family member 2A | Q9BW72 |
| HILPDA | Hypoxia inducible lipid droplet associated | Q9Y5L2 |
| HINFP | Histone H4 transcription factor | Q9BQA5 |
| HINT1 | Histidine triad nucleotide binding protein 1 | P49773 |
| HINT2 | Histidine triad nucleotide binding protein 2 | Q9BX68 |
| HINT3 | Histidine triad nucleotide binding protein 3 | Q9NQE9 |
| HIP1R | Huntingtin interacting protein 1 related | O75146 |
| HIPK1 | Homeodomain interacting protein kinase 1 | Q86Z02 |
| HIPK3 | Homeodomain interacting protein kinase 3 | Q9H422 |
| HIRA | Histone cell cycle regulator | P54198 |
| HIRIP3 | HIRA interacting protein 3 | Q9BW71 |
| HIVEP2 | HIVEP zinc finger 2 | P31629 |
| HK1 | Hexokinase 1 | P19367 |
| HK2 | Hexokinase 2 | P52789 |
| HLA-A | Major histocompatibility complex, class I, A | P04439 |
| HLA-B | Major histocompatibility complex, class I, B | P01889 |
| HLA-DMB | Major histocompatibility complex, class II, DM beta | P28068 |
| HLA-DOA | Major histocompatibility complex, class II, DO alpha | P06340 |
| HLA-DPA1 | Major histocompatibility complex, class II, DP alpha 1 | P20036 |
| HLA-DPB1 | Major histocompatibility complex, class II, DP beta 1 | P04440 |
| HLA-DQA1 | Major histocompatibility complex, class II, DQ alpha 1 | P01909 |
| HLA-DQB1 | Major histocompatibility complex, class II, DQ beta 1 |  |
| HLA-DRA | Major histocompatibility complex, class II, DR alpha | P01903 |
| HLA-DRB1 | Major histocompatibility complex, class II, DR beta 1 | P01911 |
| HLA-DRB5 | Major histocompatibility complex, class II, DR beta 5 | Q30154 |
| HLA-E | Major histocompatibility complex, class I, E | P13747 |
| HLA-F | Major histocompatibility complex, class I, F | P30511 |
| HLCS | Holocarboxylase synthetase | P50747 |
| HLTF | Helicase like transcription factor | Q14527 |
| HM13 | Histocompatibility minor 13 | Q8TCT9 |
| HMBOX1 | Homeobox containing 1 | Q6NT76 |
| HMBS | Hydroxymethylbilane synthase | P08397 |
| HMCES | 5-hydroxymethylcytosine binding, ES cell specific | Q96FZ2 |
| HMG20A | High mobility group 20A | Q9NP66 |
| HMG20B | High mobility group 20B | Q9P0W2 |
| HMGA1 | High mobility group AT-hook 1 | P17096 |
| HMGB1 | High mobility group box 1 | P09429 |
| HMGB2 | High mobility group box 2 | P26583 |
| HMGCL | 3-hydroxy-3-methylglutaryl-CoA lyase | P35914 |
| HMGCR | 3-hydroxy-3-methylglutaryl-CoA reductase | P04035 |
| HMGN1 | High mobility group nucleosome binding domain 1 | P05114 |
| HMGN2 | High mobility group nucleosomal binding domain 2 | P05204 |
| HMGN3 | High mobility group nucleosomal binding domain 3 | Q15651 |
| HMGN4 | High mobility group nucleosomal binding domain 4 | O00479 |
| HMGN5 | High mobility group nucleosome binding domain 5 | P82970 |
| HMGXB4 | HMG-box containing 4 | Q9UGU5 |
| HMOX1 | Heme oxygenase 1 | P09601 |
| HMOX2 | Heme oxygenase 2 | P30519 |
| HNRNPA0 | Heterogeneous nuclear ribonucleoprotein A0 | Q13151 |
| HNRNPA1 | Heterogeneous nuclear ribonucleoprotein A1 | P09651 |
| HNRNPAB | Heterogeneous nuclear ribonucleoprotein A/B | Q99729 |
| HNRNPC | Heterogeneous nuclear ribonucleoprotein C | P07910 |
| HNRNPD | Heterogeneous nuclear ribonucleoprotein D | Q14103 |
| HNRNPDL | Heterogeneous nuclear ribonucleoprotein D like | O14979 |
| HNRNPF | Heterogeneous nuclear ribonucleoprotein F | P52597 |
| HNRNPH1 | Heterogeneous nuclear ribonucleoprotein H1 | P31943 |
| HNRNPH2 | Heterogeneous nuclear ribonucleoprotein H2 | P55795 |
| HNRNPH3 | Heterogeneous nuclear ribonucleoprotein H3 | P31942 |
| HNRNPK | Heterogeneous nuclear ribonucleoprotein K | P61978 |
| HNRNPLL | Heterogeneous nuclear ribonucleoprotein L like | Q8WVV9 |
| HNRNPR | Heterogeneous nuclear ribonucleoprotein R | O43390 |
| HNRNPU | Heterogeneous nuclear ribonucleoprotein U | Q00839 |
| HNRNPUL1 | Heterogeneous nuclear ribonucleoprotein U like 1 | Q9BUJ2 |
| HNRNPUL2 | Heterogeneous nuclear ribonucleoprotein U like 2 | Q1KMD3 |
| HOMEZ | Homeobox and leucine zipper encoding | Q8IX15 |
| HOOK1 | Hook microtubule tethering protein 1 | Q9UJC3 |
| HOOK2 | Hook microtubule tethering protein 2 | Q96ED9 |
| HOOK3 | Hook microtubule tethering protein 3 | Q86VS8 |
| HOPX | HOP homeobox | Q9BPY8 |
| HOXB2 | Homeobox B2 | P14652 |
| HP1BP3 | Heterochromatin protein 1 binding protein 3 | Q5SSJ5 |
| HPCAL1 | Hippocalcin like 1 | P37235 |
| HPGD | 15-hydroxyprostaglandin dehydrogenase | P15428 |
| HPRT1 | Hypoxanthine phosphoribosyltransferase 1 | P00492 |
| HPS1 | HPS1 biogenesis of lysosomal organelles complex 3 subunit 1 | Q92902 |
| HPS3 | HPS3 biogenesis of lysosomal organelles complex 2 subunit 1 | Q969F9 |
| HPS4 | HPS4 biogenesis of lysosomal organelles complex 3 subunit 2 | Q9NQG7 |
| HPS5 | HPS5 biogenesis of lysosomal organelles complex 2 subunit 2 | Q9UPZ3 |
| HPS6 | HPS6 biogenesis of lysosomal organelles complex 2 subunit 3 | Q86YV9 |
| HPSE | Heparanase | Q9Y251 |
| HRH2 | Histamine receptor H2 | P25021 |
| HS1BP3 | HCLS1 binding protein 3 | Q53T59 |
| HS2ST1 | Heparan sulfate 2-O-sulfotransferase 1 | Q7LGA3 |
| HS3ST3B1 | Heparan sulfate-glucosamine 3-sulfotransferase 3B1 | Q9Y662 |
| HSBP1 | Heat shock factor binding protein 1 | O75506 |
| HSCB | HscB mitochondrial iron-sulfur cluster cochaperone | Q8IWL3 |
| HSD11B1 | Hydroxysteroid 11-beta dehydrogenase 1 | P28845 |
| HSD11B1L | Hydroxysteroid 11-beta dehydrogenase 1 like | Q7Z5J1 |
| HSD17B1 | Hydroxysteroid 17-beta dehydrogenase 1 | P14061 |
| HSD17B10 | Hydroxysteroid 17-beta dehydrogenase 10 | Q99714 |
| HSD17B11 | Hydroxysteroid 17-beta dehydrogenase 11 | Q8NBQ5 |
| HSD17B12 | Hydroxysteroid 17-beta dehydrogenase 12 | Q53GQ0 |
| HSD17B14 | Hydroxysteroid 17-beta dehydrogenase 14 | Q9BPX1 |
| HSD17B4 | Hydroxysteroid 17-beta dehydrogenase 4 | P51659 |
| HSD17B7 | Hydroxysteroid 17-beta dehydrogenase 7 | P56937 |
| HSD17B8 | Hydroxysteroid 17-beta dehydrogenase 8 | Q92506 |
| HSDL1 | Hydroxysteroid dehydrogenase like 1 | Q3SXM5 |
| HSDL2 | Hydroxysteroid dehydrogenase like 2 | Q6YN16 |
| HSF1 | Heat shock transcription factor 1 | Q00613 |
| HSF2 | Heat shock transcription factor 2 | Q03933 |
| HSH2D | Hematopoietic SH2 domain containing | Q96JZ2 |
| HSP90AA1 | Heat shock protein 90 alpha family class A member 1 | P07900 |
| HSP90AB1 | Heat shock protein 90 alpha family class B member 1 | P08238 |
| HSP90B1 | Heat shock protein 90 beta family member 1 | P14625 |
| HSPA13 | Heat shock protein family A (Hsp70) member 13 | P48723 |
| HSPA14 | Heat shock protein family A (Hsp70) member 14 | Q0VDF9 |
| HSPA1A | Heat shock protein family A (Hsp70) member 1A | P0DMV8 |
| HSPA4 | Heat shock protein family A (Hsp70) member 4 | P34932 |
| HSPA5 | Heat shock protein family A (Hsp70) member 5 | P11021 |
| HSPA6 | Heat shock protein family A (Hsp70) member 6 | P17066 |
| HSPA8 | Heat shock protein family A (Hsp70) member 8 | P11142 |
| HSPA9 | Heat shock protein family A (Hsp70) member 9 | P38646 |
| HSPB1 | Heat shock protein family B (small) member 1 | P04792 |
| HSPB11 | Heat shock protein family B (small) member 11 | Q9Y547 |
| HSPBAP1 | HSPB1 associated protein 1 | Q96EW2 |
| HSPBP1 | HSPA (Hsp70) binding protein 1 | Q9NZL4 |
| HSPD1 | Heat shock protein family D (Hsp60) member 1 | P10809 |
| HSPE1 | Heat shock protein family E (Hsp10) member 1 | P61604 |
| HSPH1 | Heat shock protein family H (Hsp110) member 1 | Q92598 |
| HTATIP2 | HIV-1 Tat interactive protein 2 | Q9BUP3 |
| HTRA2 | HtrA serine peptidase 2 | O43464 |
| HUS1 | HUS1 checkpoint clamp component | O60921 |
| HVCN1 | Hydrogen voltage gated channel 1 | Q96D96 |
| HYAL2 | Hyaluronidase 2 | Q12891 |
| HYAL3 | Hyaluronidase 3 | O43820 |
| HYI | Hydroxypyruvate isomerase (putative) | Q5T013 |
| HYLS1 | HYLS1 centriolar and ciliogenesis associated | Q96M11 |
| HYOU1 | Hypoxia up-regulated 1 | Q9Y4L1 |
| HYPK | Huntingtin interacting protein K | Q9NX55 |
| IAH1 | Isoamyl acetate hydrolyzing esterase 1 (putative) | Q2TAA2 |
| IARS2 | Isoleucyl-tRNA synthetase 2, mitochondrial | Q9NSE4 |
| IBTK | Inhibitor of Bruton tyrosine kinase | Q9P2D0 |
| ICA1L | Islet cell autoantigen 1 like | Q8NDH6 |
| ICAM1 | Intercellular adhesion molecule 1 | P05362 |
| ICAM2 | Intercellular adhesion molecule 2 | P13598 |
| ICAM3 | Intercellular adhesion molecule 3 | P32942 |
| ICMT | Isoprenylcysteine carboxyl methyltransferase | O60725 |
| ICOS | Inducible T cell costimulator | Q9Y6W8 |
| ID2 | Inhibitor of DNA binding 2 | Q02363 |
| ID3 | Inhibitor of DNA binding 3 | Q02535 |
| IDE | Insulin degrading enzyme | P14735 |
| IDH1 | Isocitrate dehydrogenase (NADP(+)) 1 | O75874 |
| IDH2 | Isocitrate dehydrogenase (NADP(+)) 2 | P48735 |
| IDH3A | Isocitrate dehydrogenase (NAD(+)) 3 catalytic subunit alpha | P50213 |
| IDH3B | Isocitrate dehydrogenase (NAD(+)) 3 non-catalytic subunit beta | O43837 |
| IDH3G | Isocitrate dehydrogenase (NAD(+)) 3 non-catalytic subunit gamma | P51553 |
| IDI1 | Isopentenyl-diphosphate delta isomerase 1 | Q13907 |
| IDNK | IDNK gluconokinase | Q5T6J7 |
| IDS | Iduronate 2-sulfatase | P22304 |
| IER2 | Immediate early response 2 | Q9BTL4 |
| IER3IP1 | Immediate early response 3 interacting protein 1 | Q9Y5U9 |
| IER5 | Immediate early response 5 | Q5VY09 |
| IFFO1 | Intermediate filament family orphan 1 | Q0D2I5 |
| IFI16 | Interferon gamma inducible protein 16 | Q16666 |
| IFI27L1 | Interferon alpha inducible protein 27 like 1 | Q96BM0 |
| IFI27L2 | Interferon alpha inducible protein 27 like 2 | Q9H2X8 |
| IFI30 | IFI30 lysosomal thiol reductase | P13284 |
| IFI35 | Interferon induced protein 35 | P80217 |
| IFI44 | Interferon induced protein 44 | Q8TCB0 |
| IFI44L | Interferon induced protein 44 like | Q53G44 |
| IFI6 | Interferon alpha inducible protein 6 | P09912 |
| IFIH1 | Interferon induced with helicase C domain 1 | Q9BYX4 |
| IFIT1 | Interferon induced protein with tetratricopeptide repeats 1 | P09914 |
| IFIT2 | Interferon induced protein with tetratricopeptide repeats 2 | P09913 |
| IFIT3 | Interferon induced protein with tetratricopeptide repeats 3 | O14879 |
| IFIT5 | Interferon induced protein with tetratricopeptide repeats 5 | Q13325 |
| IFITM1 | Interferon induced transmembrane protein 1 | P13164 |
| IFITM2 | Interferon induced transmembrane protein 2 | Q01629 |
| IFITM3 | Interferon induced transmembrane protein 3 | Q01628 |
| IFNAR1 | Interferon alpha and beta receptor subunit 1 | P17181 |
| IFNAR2 | Interferon alpha and beta receptor subunit 2 | P48551 |
| IFNG | Interferon gamma | P01579 |
| IFNGR1 | Interferon gamma receptor 1 | P15260 |
| IFNGR2 | Interferon gamma receptor 2 | P38484 |
| IFNL1 | Interferon lambda 1 | Q8IU54 |
| IFNLR1 | Interferon lambda receptor 1 | Q8IU57 |
| IFRD1 | Interferon related developmental regulator 1 | O00458 |
| IFRD2 | Interferon related developmental regulator 2 | Q12894 |
| IFT140 | Intraflagellar transport 140 | Q96RY7 |
| IFT20 | Intraflagellar transport 20 | Q8IY31 |
| IFT22 | Intraflagellar transport 22 | Q9H7X7 |
| IFT27 | Intraflagellar transport 27 | Q9BW83 |
| IFT43 | Intraflagellar transport 43 | Q96FT9 |
| IFT46 | Intraflagellar transport 46 | Q9NQC8 |
| IFT52 | Intraflagellar transport 52 | Q9Y366 |
| IFT57 | Intraflagellar transport 57 | Q9NWB7 |
| IFT74 | Intraflagellar transport 74 | Q96LB3 |
| IFT88 | Intraflagellar transport 88 | Q13099 |
| IGBP1 | Immunoglobulin binding protein 1 | P78318 |
| IGFBP3 | Insulin like growth factor binding protein 3 | P17936 |
| IGFBP4 | Insulin like growth factor binding protein 4 | P22692 |
| IGFBP6 | Insulin like growth factor binding protein 6 | P24592 |
| IGFLR1 | IGF like family receptor 1 | Q9H665 |
| IGHA1 | Immunoglobulin heavy constant alpha 1 | P01876 |
| IGKC | Immunoglobulin kappa constant |  |
| IGLC2 | Immunoglobulin lambda constant 2 | P0DOY2 |
| IGSF6 | Immunoglobulin superfamily member 6 | O95976 |
| IGSF8 | Immunoglobulin superfamily member 8 | Q969P0 |
| IK | IK cytokine | Q13123 |
| IKBIP | IKBKB interacting protein | Q70UQ0 |
| IKBKB | Inhibitor of nuclear factor kappa B kinase subunit beta | O14920 |
| IKBKE | Inhibitor of nuclear factor kappa B kinase subunit epsilon | Q14164 |
| IKZF1 | IKAROS family zinc finger 1 | Q13422 |
| IKZF2 | IKAROS family zinc finger 2 | Q9UKS7 |
| IKZF3 | IKAROS family zinc finger 3 | Q9UKT9 |
| IKZF5 | IKAROS family zinc finger 5 | Q9H5V7 |
| IL10RA | Interleukin 10 receptor subunit alpha | Q13651 |
| IL10RB | Interleukin 10 receptor subunit beta | Q08334 |
| IL11RA | Interleukin 11 receptor subunit alpha | Q14626 |
| IL12RB1 | Interleukin 12 receptor subunit beta 1 | P42701 |
| IL15 | Interleukin 15 | P40933 |
| IL15RA | Interleukin 15 receptor subunit alpha | Q13261 |
| IL16 | Interleukin 16 | Q14005 |
| IL17RA | Interleukin 17 receptor A | Q96F46 |
| IL18BP | Interleukin 18 binding protein | O95998 |
| IL18R1 | Interleukin 18 receptor 1 | Q13478 |
| IL18RAP | Interleukin 18 receptor accessory protein | O95256 |
| IL1RAP | Interleukin 1 receptor accessory protein | Q9NPH3 |
| IL21R | Interleukin 21 receptor | Q9HBE5 |
| IL23A | Interleukin 23 subunit alpha | Q9NPF7 |
| IL23R | Interleukin 23 receptor | Q5VWK5 |
| IL24 | Interleukin 24 | Q13007 |
| IL27RA | Interleukin 27 receptor subunit alpha | Q6UWB1 |
| IL2RA | Interleukin 2 receptor subunit alpha | P01589 |
| IL2RB | Interleukin 2 receptor subunit beta | P14784 |
| IL2RG | Interleukin 2 receptor subunit gamma | P31785 |
| IL32 | Interleukin 32 | P24001 |
| IL4I1 | Interleukin 4 induced 1 | Q96RQ9 |
| IL4R | Interleukin 4 receptor | P24394 |
| IL6R | Interleukin 6 receptor | P08887 |
| IL6ST | Interleukin 6 cytokine family signal transducer | P40189 |
| IL7 | Interleukin 7 | P13232 |
| IL7R | Interleukin 7 receptor | P16871 |
| ILF2 | Interleukin enhancer binding factor 2 | Q12905 |
| ILF3 | Interleukin enhancer binding factor 3 | Q12906 |
| ILK | Integrin linked kinase | Q13418 |
| ILKAP | ILK associated serine/threonine phosphatase | Q9H0C8 |
| ILVBL | IlvB acetolactate synthase like | A1L0T0 |
| IMMP1L | Inner mitochondrial membrane peptidase subunit 1 | Q96LU5 |
| IMMP2L | Inner mitochondrial membrane peptidase subunit 2 | Q96T52 |
| IMMT | Inner membrane mitochondrial protein | Q16891 |
| IMP3 | IMP U3 small nucleolar ribonucleoprotein 3 | Q9NV31 |
| IMP4 | IMP U3 small nucleolar ribonucleoprotein 4 | Q96G21 |
| IMPA1 | Inositol monophosphatase 1 | P29218 |
| IMPACT | Impact RWD domain protein | Q9P2X3 |
| IMPDH1 | Inosine monophosphate dehydrogenase 1 | P20839 |
| IMPDH2 | Inosine monophosphate dehydrogenase 2 | P12268 |
| INF2 | Inverted formin 2 | Q27J81 |
| ING1 | Inhibitor of growth family member 1 | Q9UK53 |
| ING2 | Inhibitor of growth family member 2 | Q9H160 |
| ING3 | Inhibitor of growth family member 3 | Q9NXR8 |
| ING4 | Inhibitor of growth family member 4 | Q9UNL4 |
| ING5 | Inhibitor of growth family member 5 | Q8WYH8 |
| INIP | INTS3 and NABP interacting protein | Q9NRY2 |
| INO80B | INO80 complex subunit B | Q9C086 |
| INO80C | INO80 complex subunit C | Q6PI98 |
| INO80E | INO80 complex subunit E | Q8NBZ0 |
| INPP1 | Inositol polyphosphate-1-phosphatase | P49441 |
| INPP4A | Inositol polyphosphate-4-phosphatase type I A | Q96PE3 |
| INPP4B | Inositol polyphosphate-4-phosphatase type II B | O15327 |
| INPP5B | Inositol polyphosphate-5-phosphatase B | P32019 |
| INPP5D | Inositol polyphosphate-5-phosphatase D | Q92835 |
| INPP5F | Inositol polyphosphate-5-phosphatase F | Q9Y2H2 |
| INPP5K | Inositol polyphosphate-5-phosphatase K | Q9BT40 |
| INSIG1 | Insulin induced gene 1 | O15503 |
| INSIG2 | Insulin induced gene 2 | Q9Y5U4 |
| INSL3 | Insulin like 3 | P51460 |
| INTS10 | Integrator complex subunit 10 | Q9NVR2 |
| INTS12 | Integrator complex subunit 12 | Q96CB8 |
| INTS4 | Integrator complex subunit 4 | Q96HW7 |
| INTS5 | Integrator complex subunit 5 | Q6P9B9 |
| INTS6 | Integrator complex subunit 6 | Q9UL03 |
| INTS7 | Integrator complex subunit 7 | Q9NVH2 |
| INTS9 | Integrator complex subunit 9 | Q9NV88 |
| INVS | Inversin | Q9Y283 |
| IP6K1 | Inositol hexakisphosphate kinase 1 | Q92551 |
| IP6K2 | Inositol hexakisphosphate kinase 2 | Q9UHH9 |
| IPCEF1 | Interaction protein for cytohesin exchange factors 1 | Q8WWN9 |
| IPO11 | Importin 11 | Q9UI26 |
| IPO13 | Importin 13 | O94829 |
| IPO5 | Importin 5 | O00410 |
| IPO8 | Importin 8 | O15397 |
| IPPK | Inositol-pentakisphosphate 2-kinase | Q9H8X2 |
| IQCA1 | IQ motif containing with AAA domain 1 | Q86XH1 |
| IQCB1 | IQ motif containing B1 | Q15051 |
| IQCG | IQ motif containing G | Q9H095 |
| IQSEC1 | IQ motif and Sec7 domain ArfGEF 1 | Q6DN90 |
| IRAK2 | Interleukin 1 receptor associated kinase 2 | O43187 |
| IRAK4 | Interleukin 1 receptor associated kinase 4 | Q9NWZ3 |
| IREB2 | Iron responsive element binding protein 2 | P48200 |
| IRF1 | Interferon regulatory factor 1 | P10914 |
| IRF2 | Interferon regulatory factor 2 | P14316 |
| IRF2BP2 | Interferon regulatory factor 2 binding protein 2 | Q7Z5L9 |
| IRF3 | Interferon regulatory factor 3 | Q14653 |
| IRF4 | Interferon regulatory factor 4 | Q15306 |
| IRF7 | Interferon regulatory factor 7 | Q92985 |
| IRF8 | Interferon regulatory factor 8 | Q02556 |
| IRF9 | Interferon regulatory factor 9 | Q00978 |
| ISCA1 | Iron-sulfur cluster assembly 1 | Q9BUE6 |
| ISCA2 | Iron-sulfur cluster assembly 2 | Q86U28 |
| ISCU | Iron-sulfur cluster assembly enzyme | Q9H1K1 |
| ISG15 | ISG15 ubiquitin like modifier | P05161 |
| ISG20 | Interferon stimulated exonuclease gene 20 | Q96AZ6 |
| ISG20L2 | Interferon stimulated exonuclease gene 20 like 2 | Q9H9L3 |
| ISOC1 | Isochorismatase domain containing 1 | Q96CN7 |
| ISOC2 | Isochorismatase domain containing 2 | Q96AB3 |
| IST1 | IST1 factor associated with ESCRT-III | P53990 |
| ISY1 | ISY1 splicing factor homolog | Q9ULR0 |
| ISYNA1 | Inositol-3-phosphate synthase 1 | Q9NPH2 |
| ITCH | Itchy E3 ubiquitin protein ligase | Q96J02 |
| ITFG1 | Integrin alpha FG-GAP repeat containing 1 | Q8TB96 |
| ITFG2 | Integrin alpha FG-GAP repeat containing 2 | Q969R8 |
| ITGA5 | Integrin subunit alpha 5 | P08648 |
| ITGA6 | Integrin subunit alpha 6 | P23229 |
| ITGAE | Integrin subunit alpha E | P38570 |
| ITGB1 | Integrin subunit beta 1 | P05556 |
| ITGB1BP1 | Integrin subunit beta 1 binding protein 1 | O14713 |
| ITGB2 | Integrin subunit beta 2 | P05107 |
| ITGB3BP | Integrin subunit beta 3 binding protein | Q13352 |
| ITGB7 | Integrin subunit beta 7 | P26010 |
| ITK | IL2 inducible T cell kinase | Q08881 |
| ITM2A | Integral membrane protein 2A | O43736 |
| ITM2B | Integral membrane protein 2B | Q9Y287 |
| ITM2C | Integral membrane protein 2C | Q9NQX7 |
| ITPA | Inosine triphosphatase | Q9BY32 |
| ITPK1 | Inositol-tetrakisphosphate 1-kinase | Q13572 |
| ITPKB | Inositol-trisphosphate 3-kinase B | P27987 |
| ITPRIP | Inositol 1,4,5-trisphosphate receptor interacting protein | Q8IWB1 |
| IVD | Isovaleryl-CoA dehydrogenase | P26440 |
| IVNS1ABP | Influenza virus NS1A binding protein | Q9Y6Y0 |
| IZUMO4 | IZUMO family member 4 | Q1ZYL8 |
| JADE1 | Jade family PHD finger 1 | Q6IE81 |
| JADE2 | Jade family PHD finger 2 | Q9NQC1 |
| JAGN1 | Jagunal homolog 1 | Q8N5M9 |
| JAK1 | Janus kinase 1 | P23458 |
| JAK2 | Janus kinase 2 | O60674 |
| JAK3 | Janus kinase 3 | P52333 |
| JAKMIP1 | Janus kinase and microtubule interacting protein 1 | Q96N16 |
| JAKMIP2 | Janus kinase and microtubule interacting protein 2 | Q96AA8 |
| JAM3 | Junctional adhesion molecule 3 | Q9BX67 |
| JARID2 | Jumonji and AT-rich interaction domain containing 2 | Q92833 |
| JAZF1 | JAZF zinc finger 1 | Q86VZ6 |
| JCHAIN | Joining chain of multimeric IgA and IgM | P01591 |
| JKAMP | JNK1/MAPK8 associated membrane protein | Q9P055 |
| JMJD6 | Jumonji domain containing 6, arginine demethylase and lysine hydroxylase | Q6NYC1 |
| JMJD7 | Jumonji domain containing 7 | P0C870 |
| JMJD8 | Jumonji domain containing 8 | Q96S16 |
| JOSD1 | Josephin domain containing 1 | Q15040 |
| JOSD2 | Josephin domain containing 2 | Q8TAC2 |
| JRK | Jrk helix-turn-helix protein | O75564 |
| JTB | Jumping translocation breakpoint | O76095 |
| JUNB | JunB proto-oncogene, AP-1 transcription factor subunit | P17275 |
| JUND | JunD proto-oncogene, AP-1 transcription factor subunit | P17535 |
| KANSL3 | KAT8 regulatory NSL complex subunit 3 | Q9P2N6 |
| KAT2B | Lysine acetyltransferase 2B | Q92831 |
| KAT5 | Lysine acetyltransferase 5 | Q92993 |
| KAT7 | Lysine acetyltransferase 7 | O95251 |
| KAT8 | Lysine acetyltransferase 8 | Q9H7Z6 |
| KATNA1 | Katanin catalytic subunit A1 | O75449 |
| KATNAL1 | Katanin catalytic subunit A1 like 1 | Q9BW62 |
| KATNB1 | Katanin regulatory subunit B1 | Q9BVA0 |
| KATNBL1 | Katanin regulatory subunit B1 like 1 | Q9H079 |
| KBTBD3 | Kelch repeat and BTB domain containing 3 | Q8NAB2 |
| KBTBD4 | Kelch repeat and BTB domain containing 4 | Q9NVX7 |
| KBTBD6 | Kelch repeat and BTB domain containing 6 | Q86V97 |
| KBTBD7 | Kelch repeat and BTB domain containing 7 | Q8WVZ9 |
| KCMF1 | Potassium channel modulatory factor 1 | Q9P0J7 |
| KCNA3 | Potassium voltage-gated channel subfamily A member 3 | P22001 |
| KCNAB2 | Potassium voltage-gated channel subfamily A regulatory beta subunit 2 | Q13303 |
| KCNK6 | Potassium two pore domain channel subfamily K member 6 | Q9Y257 |
| KCNN4 | Potassium calcium-activated channel subfamily N member 4 | O15554 |
| KCNQ1 | Potassium voltage-gated channel subfamily Q member 1 | P51787 |
| KCTD10 | Potassium channel tetramerization domain containing 10 | Q9H3F6 |
| KCTD13 | Potassium channel tetramerization domain containing 13 | Q8WZ19 |
| KCTD18 | Potassium channel tetramerization domain containing 18 | Q6PI47 |
| KCTD21 | Potassium channel tetramerization domain containing 21 | Q4G0X4 |
| KCTD5 | Potassium channel tetramerization domain containing 5 | Q9NXV2 |
| KCTD6 | Potassium channel tetramerization domain containing 6 | Q8NC69 |
| KCTD7 | Potassium channel tetramerization domain containing 7 | Q96MP8 |
| KCTD9 | Potassium channel tetramerization domain containing 9 | Q7L273 |
| KDELR1 | KDEL endoplasmic reticulum protein retention receptor 1 | P24390 |
| KDELR2 | KDEL endoplasmic reticulum protein retention receptor 2 | P33947 |
| KDM1A | Lysine demethylase 1A | O60341 |
| KDM2A | Lysine demethylase 2A | Q9Y2K7 |
| KDM3A | Lysine demethylase 3A | Q9Y4C1 |
| KDM3B | Lysine demethylase 3B | Q7LBC6 |
| KDM4A | Lysine demethylase 4A | O75164 |
| KDM4C | Lysine demethylase 4C | Q9H3R0 |
| KDM5A | Lysine demethylase 5A | P29375 |
| KDM5C | Lysine demethylase 5C | P41229 |
| KDM5D | Lysine demethylase 5D | Q9BY66 |
| KDM6A | Lysine demethylase 6A | O15550 |
| KDM8 | Lysine demethylase 8 | Q8N371 |
| KDSR | 3-ketodihydrosphingosine reductase | Q06136 |
| KEAP1 | Kelch like ECH associated protein 1 | Q14145 |
| KHDC1 | KH domain containing 1 | Q4VXA5 |
| KHDRBS1 | KH RNA binding domain containing, signal transduction associated 1 | Q07666 |
| KHK | Ketohexokinase | P50053 |
| KHNYN | KH and NYN domain containing | O15037 |
| KHSRP | KH-type splicing regulatory protein | Q92945 |
| KIAA0040 | KIAA0040 | Q15053 |
| KIAA0319L | KIAA0319 like | Q8IZA0 |
| KIAA0586 | KIAA0586 | Q9BVV6 |
| KIAA0930 | KIAA0930 | Q6ICG6 |
| KIAA1143 | KIAA1143 | Q96AT1 |
| KIAA1191 | KIAA1191 | Q96A73 |
| KIAA1328 | KIAA1328 | Q86T90 |
| KIAA1586 | KIAA1586 | Q9HCI6 |
| KIAA2013 | KIAA2013 | Q8IYS2 |
| KIF16B | Kinesin family member 16B | Q96L93 |
| KIF20B | Kinesin family member 20B | Q96Q89 |
| KIF22 | Kinesin family member 22 | Q14807 |
| KIF2A | Kinesin family member 2A | O00139 |
| KIF2C | Kinesin family member 2C | Q99661 |
| KIF3A | Kinesin family member 3A | Q9Y496 |
| KIF3B | Kinesin family member 3B | O15066 |
| KIF9 | Kinesin family member 9 | Q9HAQ2 |
| KIFAP3 | Kinesin associated protein 3 | Q92845 |
| KIFC3 | Kinesin family member C3 | Q9BVG8 |
| KIN | Kin17 DNA and RNA binding protein | O60870 |
| KIZ | Kizuna centrosomal protein | Q2M2Z5 |
| KLC1 | Kinesin light chain 1 | Q07866 |
| KLC2 | Kinesin light chain 2 | Q9H0B6 |
| KLC4 | Kinesin light chain 4 | Q9NSK0 |
| KLF10 | KLF transcription factor 10 | Q13118 |
| KLF11 | KLF transcription factor 11 | O14901 |
| KLF12 | KLF transcription factor 12 | Q9Y4X4 |
| KLF13 | KLF transcription factor 13 | Q9Y2Y9 |
| KLF15 | KLF transcription factor 15 | Q9UIH9 |
| KLF2 | KLF transcription factor 2 | Q9Y5W3 |
| KLF3 | KLF transcription factor 3 | P57682 |
| KLF6 | KLF transcription factor 6 | Q99612 |
| KLF7 | KLF transcription factor 7 | O75840 |
| KLHDC1 | Kelch domain containing 1 | Q8N7A1 |
| KLHDC2 | Kelch domain containing 2 | Q9Y2U9 |
| KLHDC3 | Kelch domain containing 3 | Q9BQ90 |
| KLHDC4 | Kelch domain containing 4 | Q8TBB5 |
| KLHDC7B | Kelch domain containing 7B | Q96G42 |
| KLHL12 | Kelch like family member 12 | Q53G59 |
| KLHL18 | Kelch like family member 18 | O94889 |
| KLHL2 | Kelch like family member 2 | O95198 |
| KLHL20 | Kelch like family member 20 | Q9Y2M5 |
| KLHL22 | Kelch like family member 22 | Q53GT1 |
| KLHL23 | Kelch like family member 23 | Q8NBE8 |
| KLHL25 | Kelch like family member 25 | Q9H0H3 |
| KLHL26 | Kelch like family member 26 | Q53HC5 |
| KLHL32 | Kelch like family member 32 | Q96NJ5 |
| KLHL36 | Kelch like family member 36 | Q8N4N3 |
| KLHL42 | Kelch like family member 42 | Q9P2K6 |
| KLHL6 | Kelch like family member 6 | Q8WZ60 |
| KLHL7 | Kelch like family member 7 | Q8IXQ5 |
| KLHL8 | Kelch like family member 8 | Q9P2G9 |
| KLHL9 | Kelch like family member 9 | Q9P2J3 |
| KLRB1 | Killer cell lectin like receptor B1 | Q12918 |
| KLRC1 | Killer cell lectin like receptor C1 | P26715 |
| KLRC2 | Killer cell lectin like receptor C2 | P26717 |
| KLRC3 | Killer cell lectin like receptor C3 | Q07444 |
| KLRC4 | Killer cell lectin like receptor C4 | O43908 |
| KLRD1 | Killer cell lectin like receptor D1 | Q13241 |
| KLRF1 | Killer cell lectin like receptor F1 | Q9NZS2 |
| KLRG1 | Killer cell lectin like receptor G1 | Q96E93 |
| KLRK1 | Killer cell lectin like receptor K1 | P26718 |
| KMT2E | Lysine methyltransferase 2E (inactive) | Q8IZD2 |
| KNOP1 | Lysine rich nucleolar protein 1 | Q1ED39 |
| KNSTRN | Kinetochore localized astrin (SPAG5) binding protein | Q9Y448 |
| KPNA1 | Karyopherin subunit alpha 1 | P52294 |
| KPNA2 | Karyopherin subunit alpha 2 | P52292 |
| KPNA3 | Karyopherin subunit alpha 3 | O00505 |
| KPNA4 | Karyopherin subunit alpha 4 | O00629 |
| KPNA5 | Karyopherin subunit alpha 5 | O15131 |
| KPNA6 | Karyopherin subunit alpha 6 | O60684 |
| KPNB1 | Karyopherin subunit beta 1 | Q14974 |
| KPTN | Kaptin, actin binding protein | Q9Y664 |
| KRAS | KRAS proto-oncogene, GTPase | P01116 |
| KRBA2 | KRAB-A domain containing 2 | Q6ZNG9 |
| KRBOX4 | KRAB box domain containing 4 | Q5JUW0 |
| KRCC1 | Lysine rich coiled-coil 1 | Q9NPI7 |
| KRI1 | KRI1 homolog | Q8N9T8 |
| KRIT1 | KRIT1 ankyrin repeat containing | O00522 |
| KRR1 | KRR1 small subunit processome component homolog | Q13601 |
| KRT10 | Keratin 10 | P13645 |
| KRT18 | Keratin 18 | P05783 |
| KRTCAP2 | Keratinocyte associated protein 2 | Q8N6L1 |
| KRTCAP3 | Keratinocyte associated protein 3 | Q53RY4 |
| KTI12 | KTI12 chromatin associated homolog | Q96EK9 |
| KTN1 | Kinectin 1 | Q86UP2 |
| KXD1 | KxDL motif containing 1 | Q9BQD3 |
| L2HGDH | L-2-hydroxyglutarate dehydrogenase | Q9H9P8 |
| L3HYPDH | Trans-L-3-hydroxyproline dehydratase | Q96EM0 |
| L3MBTL2 | L3MBTL histone methyl-lysine binding protein 2 | Q969R5 |
| L3MBTL3 | L3MBTL histone methyl-lysine binding protein 3 | Q96JM7 |
| LACTB | Lactamase beta | P83111 |
| LACTB2 | Lactamase beta 2 | Q53H82 |
| LAG3 | Lymphocyte activating 3 | P18627 |
| LAGE3 | L antigen family member 3 | Q14657 |
| LAIR1 | Leukocyte associated immunoglobulin like receptor 1 | Q6GTX8 |
| LAIR2 | Leukocyte associated immunoglobulin like receptor 2 | Q6ISS4 |
| LAMP1 | Lysosomal associated membrane protein 1 | P11279 |
| LAMP2 | Lysosomal associated membrane protein 2 | P13473 |
| LAMP3 | Lysosomal associated membrane protein 3 | Q9UQV4 |
| LAMTOR1 | Late endosomal/lysosomal adaptor, MAPK and MTOR activator 1 | Q6IAA8 |
| LAMTOR2 | Late endosomal/lysosomal adaptor, MAPK and MTOR activator 2 | Q9Y2Q5 |
| LAMTOR3 | Late endosomal/lysosomal adaptor, MAPK and MTOR activator 3 | Q9UHA4 |
| LAMTOR4 | Late endosomal/lysosomal adaptor, MAPK and MTOR activator 4 | Q0VGL1 |
| LAMTOR5 | Late endosomal/lysosomal adaptor, MAPK and MTOR activator 5 | O43504 |
| LANCL1 | LanC like glutathione S-transferase 1 | O43813 |
| LANCL2 | LanC like glutathione S-transferase 2 | Q9NS86 |
| LAP3 | Leucine aminopeptidase 3 | P28838 |
| LAPTM4A | Lysosomal protein transmembrane 4 alpha | Q15012 |
| LAPTM5 | Lysosomal protein transmembrane 5 | Q13571 |
| LARP1 | La ribonucleoprotein 1, translational regulator | Q6PKG0 |
| LARP1B | La ribonucleoprotein 1B | Q659C4 |
| LARP4 | La ribonucleoprotein 4 | Q71RC2 |
| LARP4B | La ribonucleoprotein 4B | Q92615 |
| LARP7 | La ribonucleoprotein 7, transcriptional regulator | Q4G0J3 |
| LARS2 | Leucyl-tRNA synthetase 2, mitochondrial | Q15031 |
| LAS1L | LAS1 like ribosome biogenesis factor | Q9Y4W2 |
| LASP1 | LIM and SH3 protein 1 | Q14847 |
| LAT | Linker for activation of T cells | O43561 |
| LAT2 | Linker for activation of T cells family member 2 | Q9GZY6 |
| LATS1 | Large tumor suppressor kinase 1 | O95835 |
| LAX1 | Lymphocyte transmembrane adaptor 1 | Q8IWV1 |
| LBH | LBH regulator of WNT signaling pathway | Q53QV2 |
| LBHD1 | LBH domain containing 1 | Q9BQE6 |
| LBR | Lamin B receptor | Q14739 |
| LCK | LCK proto-oncogene, Src family tyrosine kinase | P06239 |
| LCMT1 | Leucine carboxyl methyltransferase 1 | Q9UIC8 |
| LCMT2 | Leucine carboxyl methyltransferase 2 | O60294 |
| LCOR | Ligand dependent nuclear receptor corepressor | Q96JN0 |
| LCORL | Ligand dependent nuclear receptor corepressor like | Q8N3X6 |
| LCP1 | Lymphocyte cytosolic protein 1 | P13796 |
| LCP2 | Lymphocyte cytosolic protein 2 | Q13094 |
| LDAH | Lipid droplet associated hydrolase | Q9H6V9 |
| LDB1 | LIM domain binding 1 | Q86U70 |
| LDHA | Lactate dehydrogenase A | P00338 |
| LDHB | Lactate dehydrogenase B | P07195 |
| LDLR | Low density lipoprotein receptor | P01130 |
| LDLRAD4 | Low density lipoprotein receptor class A domain containing 4 | O15165 |
| LDOC1 | LDOC1 regulator of NFKB signaling | O95751 |
| LEF1 | Lymphoid enhancer binding factor 1 | Q9UJU2 |
| LEMD3 | LEM domain containing 3 | Q9Y2U8 |
| LEPROT | Leptin receptor overlapping transcript | O15243 |
| LEPROTL1 | Leptin receptor overlapping transcript like 1 | O95214 |
| LETM1 | Leucine zipper and EF-hand containing transmembrane protein 1 | O95202 |
| LETM2 | Leucine zipper and EF-hand containing transmembrane protein 2 | Q2VYF4 |
| LETMD1 | LETM1 domain containing 1 | Q6P1Q0 |
| LEXM | Lymphocyte expansion molecule | Q3ZCV2 |
| LFNG | LFNG O-fucosylpeptide 3-beta-N-acetylglucosaminyltransferase | Q8NES3 |
| LGALS1 | Galectin 1 | P09382 |
| LGALS2 | Galectin 2 | P05162 |
| LGALS3 | Galectin 3 | P17931 |
| LGALS3BP | Galectin 3 binding protein | Q08380 |
| LGALS8 | Galectin 8 | O00214 |
| LGALS9 | Galectin 9 | O00182 |
| LGMN | Legumain | Q99538 |
| LGR6 | Leucine rich repeat containing G protein-coupled receptor 6 | Q9HBX8 |
| LHPP | Phospholysine phosphohistidine inorganic pyrophosphate phosphatase | Q9H008 |
| LIAS | Lipoic acid synthetase | O43766 |
| LIG1 | DNA ligase 1 | P18858 |
| LIG3 | DNA ligase 3 | P49916 |
| LIG4 | DNA ligase 4 | P49917 |
| LILRB1 | Leukocyte immunoglobulin like receptor B1 |  |
| LIM2 | Lens intrinsic membrane protein 2 | P55344 |
| LIMA1 | LIM domain and actin binding 1 | Q9UHB6 |
| LIMD2 | LIM domain containing 2 | Q9BT23 |
| LIME1 | Lck interacting transmembrane adaptor 1 | Q9H400 |
| LIMK1 | LIM domain kinase 1 | P53667 |
| LIMK2 | LIM domain kinase 2 | P53671 |
| LIMS1 | LIM zinc finger domain containing 1 | P48059 |
| LIN37 | Lin-37 DREAM MuvB core complex component | Q96GY3 |
| LIN52 | Lin-52 DREAM MuvB core complex component |  |
| LIN54 | Lin-54 DREAM MuvB core complex component | Q6MZP7 |
| LIN7B | Lin-7 homolog B, crumbs cell polarity complex component | Q9HAP6 |
| LIN7C | Lin-7 homolog C, crumbs cell polarity complex component | Q9NUP9 |
| LIN9 | Lin-9 DREAM MuvB core complex component | Q5TKA1 |
| LINS1 | Lines homolog 1 | Q8NG48 |
| LIPA | Lipase A, lysosomal acid type | P38571 |
| LIPC | Lipase C, hepatic type | P11150 |
| LIPT1 | Lipoyltransferase 1 | Q9Y234 |
| LITAF | Lipopolysaccharide induced TNF factor | Q99732 |
| LIX1L | Limb and CNS expressed 1 like | Q8IVB5 |
| LLGL2 | LLGL scribble cell polarity complex component 2 | Q6P1M3 |
| LLPH | LLP homolog, long-term synaptic facilitation factor | Q9BRT6 |
| LMAN1 | Lectin, mannose binding 1 | P49257 |
| LMAN2 | Lectin, mannose binding 2 | Q12907 |
| LMAN2L | Lectin, mannose binding 2 like | Q9H0V9 |
| LMBR1L | Limb development membrane protein 1 like | Q6UX01 |
| LMBRD1 | LMBR1 domain containing 1 | Q9NUN5 |
| LMBRD2 | LMBR1 domain containing 2 | Q68DH5 |
| LMF1 | Lipase maturation factor 1 | Q96S06 |
| LMF2 | Lipase maturation factor 2 | Q9BU23 |
| LMNA | Lamin A/C | P02545 |
| LMNB1 | Lamin B1 | P20700 |
| LMNB2 | Lamin B2 | Q03252 |
| LMO4 | LIM domain only 4 | P61968 |
| LNPEP | Leucyl and cystinyl aminopeptidase | Q9UIQ6 |
| LONP1 | Lon peptidase 1, mitochondrial | P36776 |
| LPAR2 | Lysophosphatidic acid receptor 2 | Q9HBW0 |
| LPAR5 | Lysophosphatidic acid receptor 5 | Q9H1C0 |
| LPAR6 | Lysophosphatidic acid receptor 6 | P43657 |
| LPCAT3 | Lysophosphatidylcholine acyltransferase 3 | Q6P1A2 |
| LPCAT4 | Lysophosphatidylcholine acyltransferase 4 | Q643R3 |
| LPGAT1 | Lysophosphatidylglycerol acyltransferase 1 | Q92604 |
| LPIN1 | Lipin 1 | Q14693 |
| LPIN2 | Lipin 2 | Q92539 |
| LPXN | Leupaxin | O60711 |
| LRCH1 | Leucine rich repeats and calponin homology domain containing 1 | Q9Y2L9 |
| LRCH3 | Leucine rich repeats and calponin homology domain containing 3 | Q96II8 |
| LRCH4 | Leucine rich repeats and calponin homology domain containing 4 | O75427 |
| LRP10 | LDL receptor related protein 10 | Q7Z4F1 |
| LRPAP1 | LDL receptor related protein associated protein 1 | P30533 |
| LRPPRC | Leucine rich pentatricopeptide repeat containing | P42704 |
| LRR1 | Leucine rich repeat protein 1 | Q96L50 |
| LRRC14 | Leucine rich repeat containing 14 | Q15048 |
| LRRC23 | Leucine rich repeat containing 23 | Q53EV4 |
| LRRC27 | Leucine rich repeat containing 27 | Q9C0I9 |
| LRRC28 | Leucine rich repeat containing 28 | Q86X40 |
| LRRC37B | Leucine rich repeat containing 37B | Q96QE4 |
| LRRC40 | Leucine rich repeat containing 40 | Q9H9A6 |
| LRRC42 | Leucine rich repeat containing 42 | Q9Y546 |
| LRRC45 | Leucine rich repeat containing 45 | Q96CN5 |
| LRRC47 | Leucine rich repeat containing 47 | Q8N1G4 |
| LRRC57 | Leucine rich repeat containing 57 | Q8N9N7 |
| LRRC58 | Leucine rich repeat containing 58 | Q96CX6 |
| LRRC59 | Leucine rich repeat containing 59 | Q96AG4 |
| LRRC61 | Leucine rich repeat containing 61 | Q9BV99 |
| LRRFIP1 | LRR binding FLII interacting protein 1 | Q32MZ4 |
| LRRFIP2 | LRR binding FLII interacting protein 2 | Q9Y608 |
| LRRIQ3 | Leucine rich repeats and IQ motif containing 3 | A6PVS8 |
| LRRN3 | Leucine rich repeat neuronal 3 | Q9H3W5 |
| LRSAM1 | Leucine rich repeat and sterile alpha motif containing 1 | Q6UWE0 |
| LRWD1 | Leucine rich repeats and WD repeat domain containing 1 | Q9UFC0 |
| LSG1 | Large 60S subunit nuclear export GTPase 1 | Q9H089 |
| LSM1 | LSM1 homolog, mRNA degradation associated | O15116 |
| LSM10 | LSM10, U7 small nuclear RNA associated | Q969L4 |
| LSM12 | LSM12 homolog | Q3MHD2 |
| LSM14A | LSM14A mRNA processing body assembly factor | Q8ND56 |
| LSM2 | LSM2 homolog, U6 small nuclear RNA and mRNA degradation associated | Q9Y333 |
| LSM3 | LSM3 homolog, U6 small nuclear RNA and mRNA degradation associated | P62310 |
| LSM4 | LSM4 homolog, U6 small nuclear RNA and mRNA degradation associated | Q9Y4Z0 |
| LSM5 | LSM5 homolog, U6 small nuclear RNA and mRNA degradation associated | Q9Y4Y9 |
| LSM6 | LSM6 homolog, U6 small nuclear RNA and mRNA degradation associated | P62312 |
| LSM7 | LSM7 homolog, U6 small nuclear RNA and mRNA degradation associated | Q9UK45 |
| LSM8 | LSM8 homolog, U6 small nuclear RNA associated | O95777 |
| LSP1 | Lymphocyte specific protein 1 | P33241 |
| LSR | Lipolysis stimulated lipoprotein receptor | Q86X29 |
| LSS | Lanosterol synthase | P48449 |
| LST1 | Leukocyte specific transcript 1 | O00453 |
| LTA4H | Leukotriene A4 hydrolase | P09960 |
| LTB | Lymphotoxin beta | Q06643 |
| LTB4R | Leukotriene B4 receptor | Q15722 |
| LTBP3 | Latent transforming growth factor beta binding protein 3 | Q9NS15 |
| LTBP4 | Latent transforming growth factor beta binding protein 4 | Q8N2S1 |
| LTC4S | Leukotriene C4 synthase | Q16873 |
| LTV1 | LTV1 ribosome biogenesis factor | Q96GA3 |
| LUC7L | LUC7 like | Q9NQ29 |
| LUC7L2 | LUC7 like 2, pre-mRNA splicing factor | Q9Y383 |
| LUC7L3 | LUC7 like 3 pre-mRNA splicing factor | O95232 |
| LUZP1 | Leucine zipper protein 1 | Q86V48 |
| LXN | Latexin | Q9BS40 |
| LY6E | Lymphocyte antigen 6 family member E | Q16553 |
| LY75 | Lymphocyte antigen 75 | O60449 |
| LY86 | Lymphocyte antigen 86 | O95711 |
| LY9 | Lymphocyte antigen 9 | Q9HBG7 |
| LY96 | Lymphocyte antigen 96 | Q9Y6Y9 |
| LYAR | Ly1 antibody reactive | Q9NX58 |
| LYPD3 | LY6/PLAUR domain containing 3 | O95274 |
| LYPLA1 | Lysophospholipase 1 | O75608 |
| LYPLA2 | Lysophospholipase 2 | O95372 |
| LYPLAL1 | Lysophospholipase like 1 | Q5VWZ2 |
| LYRM1 | LYR motif containing 1 | O43325 |
| LYRM2 | LYR motif containing 2 | Q9NU23 |
| LYRM4 | LYR motif containing 4 | Q9HD34 |
| LYRM7 | LYR motif containing 7 | Q5U5X0 |
| LYSMD1 | LysM domain containing 1 | Q96S90 |
| LYSMD2 | LysM domain containing 2 | Q8IV50 |
| LYSMD3 | LysM domain containing 3 | Q7Z3D4 |
| LYSMD4 | LysM domain containing 4 | Q5XG99 |
| LYST | Lysosomal trafficking regulator | Q99698 |
| LYZ | Lysozyme | P61626 |
| LZIC | Leucine zipper and CTNNBIP1 domain containing | Q8WZA0 |
| LZTS2 | Leucine zipper tumor suppressor 2 | Q9BRK4 |
| M6PR | Mannose-6-phosphate receptor, cation dependent | P20645 |
| MAD1L1 | Mitotic arrest deficient 1 like 1 | Q9Y6D9 |
| MAD2L1 | Mitotic arrest deficient 2 like 1 | Q13257 |
| MAD2L1BP | MAD2L1 binding protein | Q15013 |
| MAD2L2 | Mitotic arrest deficient 2 like 2 | Q9UI95 |
| MAEA | Macrophage erythroblast attacher, E3 ubiquitin ligase | Q7L5Y9 |
| MAF | MAF bZIP transcription factor | O75444 |
| MAF1 | MAF1 homolog, negative regulator of RNA polymerase III | Q9H063 |
| MAFF | MAF bZIP transcription factor F | Q9ULX9 |
| MAGED1 | MAGE family member D1 | Q9Y5V3 |
| MAGED2 | MAGE family member D2 | Q9UNF1 |
| MAGEF1 | MAGE family member F1 | Q9HAY2 |
| MAGEH1 | MAGE family member H1 | Q9H213 |
| MAGOH | Mago homolog, exon junction complex subunit | P61326 |
| MAGOHB | Mago homolog B, exon junction complex subunit | Q96A72 |
| MAGT1 | Magnesium transporter 1 | Q9H0U3 |
| MAK16 | MAK16 homolog | Q9BXY0 |
| MAL | Mal, T cell differentiation protein | P21145 |
| MALSU1 | Mitochondrial assembly of ribosomal large subunit 1 | Q96EH3 |
| MAML2 | Mastermind like transcriptional coactivator 2 | Q8IZL2 |
| MAN1A2 | Mannosidase alpha class 1A member 2 | O60476 |
| MAN1B1 | Mannosidase alpha class 1B member 1 | Q9UKM7 |
| MAN1C1 | Mannosidase alpha class 1C member 1 | Q9NR34 |
| MAN2B1 | Mannosidase alpha class 2B member 1 | O00754 |
| MAN2B2 | Mannosidase alpha class 2B member 2 | Q9Y2E5 |
| MAN2C1 | Mannosidase alpha class 2C member 1 | Q9NTJ4 |
| MANBAL | Mannosidase beta like | Q9NQG1 |
| MANEA | Mannosidase endo-alpha | Q5SRI9 |
| MANF | Mesencephalic astrocyte derived neurotrophic factor | P55145 |
| MAP1LC3A | Microtubule associated protein 1 light chain 3 alpha | Q9H492 |
| MAP1LC3B | Microtubule associated protein 1 light chain 3 beta | Q9GZQ8 |
| MAP1LC3B2 | Microtubule associated protein 1 light chain 3 beta 2 | A6NCE7 |
| MAP1S | Microtubule associated protein 1S | Q66K74 |
| MAP2K2 | Mitogen-activated protein kinase kinase 2 | P36507 |
| MAP2K3 | Mitogen-activated protein kinase kinase 3 | P46734 |
| MAP2K4 | Mitogen-activated protein kinase kinase 4 | P45985 |
| MAP2K5 | Mitogen-activated protein kinase kinase 5 | Q13163 |
| MAP2K6 | Mitogen-activated protein kinase kinase 6 | P52564 |
| MAP3K11 | Mitogen-activated protein kinase kinase kinase 11 | Q16584 |
| MAP3K13 | Mitogen-activated protein kinase kinase kinase 13 | O43283 |
| MAP3K14 | Mitogen-activated protein kinase kinase kinase 14 | Q99558 |
| MAP3K2 | Mitogen-activated protein kinase kinase kinase 2 | Q9Y2U5 |
| MAP3K5 | Mitogen-activated protein kinase kinase kinase 5 | Q99683 |
| MAP3K6 | Mitogen-activated protein kinase kinase kinase 6 | O95382 |
| MAP3K7 | Mitogen-activated protein kinase kinase kinase 7 | O43318 |
| MAP3K7CL | MAP3K7 C-terminal like | P57077 |
| MAP3K8 | Mitogen-activated protein kinase kinase kinase 8 | P41279 |
| MAP4 | Microtubule associated protein 4 | P27816 |
| MAP4K1 | Mitogen-activated protein kinase kinase kinase kinase 1 | Q92918 |
| MAP4K2 | Mitogen-activated protein kinase kinase kinase kinase 2 | Q12851 |
| MAP4K4 | Mitogen-activated protein kinase kinase kinase kinase 4 | O95819 |
| MAP4K5 | Mitogen-activated protein kinase kinase kinase kinase 5 | Q9Y4K4 |
| MAP9 | Microtubule associated protein 9 | Q49MG5 |
| MAPK1 | Mitogen-activated protein kinase 1 | P28482 |
| MAPK10 | Mitogen-activated protein kinase 10 | P53779 |
| MAPK13 | Mitogen-activated protein kinase 13 | O15264 |
| MAPK14 | Mitogen-activated protein kinase 14 | Q16539 |
| MAPK1IP1L | Mitogen-activated protein kinase 1 interacting protein 1 like | Q8NDC0 |
| MAPK3 | Mitogen-activated protein kinase 3 | P27361 |
| MAPK6 | Mitogen-activated protein kinase 6 | Q16659 |
| MAPK7 | Mitogen-activated protein kinase 7 | Q13164 |
| MAPK8 | Mitogen-activated protein kinase 8 | P45983 |
| MAPK9 | Mitogen-activated protein kinase 9 | P45984 |
| MAPKAP1 | MAPK associated protein 1 | Q9BPZ7 |
| MAPKAPK2 | MAPK activated protein kinase 2 | P49137 |
| MAPKAPK3 | MAPK activated protein kinase 3 | Q16644 |
| MAPKAPK5 | MAPK activated protein kinase 5 | Q8IW41 |
| MAPRE1 | Microtubule associated protein RP/EB family member 1 | Q15691 |
| MAPRE2 | Microtubule associated protein RP/EB family member 2 | Q15555 |
| MARCHF2 | Membrane associated ring-CH-type finger 2 | Q9P0N8 |
| MARCHF5 | Membrane associated ring-CH-type finger 5 | Q9NX47 |
| MARCHF6 | Membrane associated ring-CH-type finger 6 | O60337 |
| MARCHF8 | Membrane associated ring-CH-type finger 8 | Q5T0T0 |
| MARCHF9 | Membrane associated ring-CH-type finger 9 | Q86YJ5 |
| MARCKSL1 | MARCKS like 1 | P49006 |
| MARK2 | Microtubule affinity regulating kinase 2 | Q7KZI7 |
| MARK3 | Microtubule affinity regulating kinase 3 | P27448 |
| MARS2 | Methionyl-tRNA synthetase 2, mitochondrial | Q96GW9 |
| MAST3 | Microtubule associated serine/threonine kinase 3 | O60307 |
| MAST4 | Microtubule associated serine/threonine kinase family member 4 | O15021 |
| MAT2A | Methionine adenosyltransferase 2A | P31153 |
| MAT2B | Methionine adenosyltransferase 2B | Q9NZL9 |
| MATK | Megakaryocyte-associated tyrosine kinase | P42679 |
| MATR3 | Matrin 3 | P43243 |
| MATR3 | Matrin 3 |  |
| MAU2 | MAU2 sister chromatid cohesion factor | Q9Y6X3 |
| MAVS | Mitochondrial antiviral signaling protein | Q7Z434 |
| MAX | MYC associated factor X | P61244 |
| MB21D2 | Mab-21 domain containing 2 | Q8IYB1 |
| MBD1 | Methyl-CpG binding domain protein 1 | Q9UIS9 |
| MBD2 | Methyl-CpG binding domain protein 2 | Q9UBB5 |
| MBD4 | Methyl-CpG binding domain 4, DNA glycosylase | O95243 |
| MBIP | MAP3K12 binding inhibitory protein 1 | Q9NS73 |
| MBNL1 | Muscleblind like splicing regulator 1 | Q9NR56 |
| MBNL2 | Muscleblind like splicing regulator 2 | Q5VZF2 |
| MBNL3 | Muscleblind like splicing regulator 3 | Q9NUK0 |
| MBOAT7 | Membrane bound O-acyltransferase domain containing 7 | Q96N66 |
| MBP | Myelin basic protein | P02686 |
| MBTPS1 | Membrane bound transcription factor peptidase, site 1 | Q14703 |
| MBTPS2 | Membrane bound transcription factor peptidase, site 2 | O43462 |
| MCAT | Malonyl-CoA-acyl carrier protein transacylase | Q8IVS2 |
| MCCC1 | Methylcrotonyl-CoA carboxylase subunit 1 | Q96RQ3 |
| MCCC2 | Methylcrotonyl-CoA carboxylase subunit 2 | Q9HCC0 |
| MCEE | Methylmalonyl-CoA epimerase | Q96PE7 |
| MCFD2 | Multiple coagulation factor deficiency 2, ER cargo receptor complex subunit | Q8NI22 |
| MCL1 | MCL1 apoptosis regulator, BCL2 family member | Q07820 |
| MCM2 | Minichromosome maintenance complex component 2 | P49736 |
| MCM3 | Minichromosome maintenance complex component 3 | P25205 |
| MCM4 | Minichromosome maintenance complex component 4 | P33991 |
| MCM5 | Minichromosome maintenance complex component 5 | P33992 |
| MCM6 | Minichromosome maintenance complex component 6 | Q14566 |
| MCM7 | Minichromosome maintenance complex component 7 | P33993 |
| MCM9 | Minichromosome maintenance 9 homologous recombination repair factor | Q9NXL9 |
| MCOLN1 | Mucolipin TRP cation channel 1 | Q9GZU1 |
| MCOLN2 | Mucolipin TRP cation channel 2 | Q8IZK6 |
| MCPH1 | Microcephalin 1 | Q8NEM0 |
| MCRS1 | Microspherule protein 1 | Q96EZ8 |
| MCTP2 | Multiple C2 and transmembrane domain containing 2 | Q6DN12 |
| MCTS1 | MCTS1 re-initiation and release factor | Q9ULC4 |
| MCU | Mitochondrial calcium uniporter | Q8NE86 |
| MCUR1 | Mitochondrial calcium uniporter regulator 1 | Q96AQ8 |
| MDFIC | MyoD family inhibitor domain containing | Q9P1T7 |
| MDH1 | Malate dehydrogenase 1 | P40925 |
| MDH2 | Malate dehydrogenase 2 | P40926 |
| MDM1 | Mdm1 nuclear protein | Q8TC05 |
| MDM2 | MDM2 proto-oncogene | Q00987 |
| MDM4 | MDM4 regulator of p53 | O15151 |
| MDP1 | Magnesium dependent phosphatase 1 | Q86V88 |
| ME1 | Malic enzyme 1 | P48163 |
| ME3 | Malic enzyme 3 | Q16798 |
| MEA1 | Male-enhanced antigen 1 | Q16626 |
| MEAF6 | MYST/Esa1 associated factor 6 | Q9HAF1 |
| MECP2 | Methyl-CpG binding protein 2 | P51608 |
| MECR | Mitochondrial trans-2-enoyl-CoA reductase | Q9BV79 |
| MED1 | Mediator complex subunit 1 | Q15648 |
| MED10 | Mediator complex subunit 10 | Q9BTT4 |
| MED11 | Mediator complex subunit 11 | Q9P086 |
| MED12 | Mediator complex subunit 12 | Q93074 |
| MED15 | Mediator complex subunit 15 | Q96RN5 |
| MED16 | Mediator complex subunit 16 | Q9Y2X0 |
| MED17 | Mediator complex subunit 17 | Q9NVC6 |
| MED18 | Mediator complex subunit 18 | Q9BUE0 |
| MED19 | Mediator complex subunit 19 | A0JLT2 |
| MED20 | Mediator complex subunit 20 | Q9H944 |
| MED21 | Mediator complex subunit 21 | Q13503 |
| MED23 | Mediator complex subunit 23 | Q9ULK4 |
| MED24 | Mediator complex subunit 24 | O75448 |
| MED25 | Mediator complex subunit 25 | Q71SY5 |
| MED27 | Mediator complex subunit 27 | Q6P2C8 |
| MED28 | Mediator complex subunit 28 | Q9H204 |
| MED29 | Mediator complex subunit 29 | Q9NX70 |
| MED30 | Mediator complex subunit 30 | Q96HR3 |
| MED31 | Mediator complex subunit 31 | Q9Y3C7 |
| MED4 | Mediator complex subunit 4 | Q9NPJ6 |
| MED6 | Mediator complex subunit 6 | O75586 |
| MED7 | Mediator complex subunit 7 | O43513 |
| MED8 | Mediator complex subunit 8 | Q96G25 |
| MED9 | Mediator complex subunit 9 | Q9NWA0 |
| MEF2A | Myocyte enhancer factor 2A | Q02078 |
| MEF2D | Myocyte enhancer factor 2D | Q14814 |
| MEI1 | Meiotic double-stranded break formation protein 1 | Q5TIA1 |
| MEMO1 | Mediator of cell motility 1 | Q9Y316 |
| MEN1 | Menin 1 | O00255 |
| MEOX1 | Mesenchyme homeobox 1 | P50221 |
| MEPCE | Methylphosphate capping enzyme | Q7L2J0 |
| MEST | Mesoderm specific transcript | Q5EB52 |
| METAP1D | Methionyl aminopeptidase type 1D, mitochondrial | Q6UB28 |
| METAP2 | Methionyl aminopeptidase 2 | P50579 |
| METTL1 | Methyltransferase 1, tRNA methylguanosine | Q9UBP6 |
| METTL13 | Methyltransferase 13, eEF1A lysine and N-terminal methyltransferase | Q8N6R0 |
| METTL14 | Methyltransferase 14, N6-adenosine-methyltransferase subunit | Q9HCE5 |
| METTL15 | Methyltransferase like 15 | A6NJ78 |
| METTL16 | Methyltransferase 16, N6-methyladenosine | Q86W50 |
| METTL17 | Methyltransferase like 17 | Q9H7H0 |
| METTL18 | Methyltransferase like 18 | O95568 |
| METTL21A | Methyltransferase 21A, HSPA lysine | Q8WXB1 |
| METTL22 | Methyltransferase 22, Kin17 lysine | Q9BUU2 |
| METTL25 | Methyltransferase like 25 | Q8N6Q8 |
| METTL2A | Methyltransferase 2A, methylcytidine | Q96IZ6 |
| METTL2B | Methyltransferase 2B, methylcytidine | Q6P1Q9 |
| METTL3 | Methyltransferase 3, N6-adenosine-methyltransferase complex catalytic subunit | Q86U44 |
| METTL4 | Methyltransferase 4, N6-adenosine | Q8N3J2 |
| METTL5 | Methyltransferase 5, N6-adenosine | Q9NRN9 |
| METTL6 | Methyltransferase 6, methylcytidine | Q8TCB7 |
| METTL7A | Methyltransferase like 7A | Q9H8H3 |
| METTL8 | Methyltransferase 8, methylcytidine |  |
| METTL9 | Methyltransferase like 9 | Q9H1A3 |
| MEX3C | Mex-3 RNA binding family member C | Q5U5Q3 |
| MFAP1 | Microfibril associated protein 1 | P55081 |
| MFAP3 | Microfibril associated protein 3 | P55082 |
| MFF | Mitochondrial fission factor | Q9GZY8 |
| MFGE8 | Milk fat globule EGF and factor V/VIII domain containing | Q08431 |
| MFN1 | Mitofusin 1 | Q8IWA4 |
| MFN2 | Mitofusin 2 | O95140 |
| MFNG | MFNG O-fucosylpeptide 3-beta-N-acetylglucosaminyltransferase | O00587 |
| MFSD10 | Major facilitator superfamily domain containing 10 | Q14728 |
| MFSD11 | Major facilitator superfamily domain containing 11 | O43934 |
| MFSD12 | Major facilitator superfamily domain containing 12 | Q6NUT3 |
| MFSD13A | Major facilitator superfamily domain containing 13A | Q14CX5 |
| MFSD3 | Major facilitator superfamily domain containing 3 | Q96ES6 |
| MFSD5 | Major facilitator superfamily domain containing 5 | Q6N075 |
| MFSD8 | Major facilitator superfamily domain containing 8 | Q8NHS3 |
| MFSD9 | Major facilitator superfamily domain containing 9 | Q8NBP5 |
| MGAT1 | Alpha-1,3-mannosyl-glycoprotein 2-beta-N-acetylglucosaminyltransferase | P26572 |
| MGAT2 | Alpha-1,6-mannosyl-glycoprotein 2-beta-N-acetylglucosaminyltransferase | Q10469 |
| MGAT4A | Alpha-1,3-mannosyl-glycoprotein 4-beta-N-acetylglucosaminyltransferase A | Q9UM21 |
| MGAT5 | Alpha-1,6-mannosylglycoprotein 6-beta-N-acetylglucosaminyltransferase | Q09328 |
| MGLL | Monoglyceride lipase | Q99685 |
| MGME1 | Mitochondrial genome maintenance exonuclease 1 | Q9BQP7 |
| MGMT | O-6-methylguanine-DNA methyltransferase | P16455 |
| MGRN1 | Mahogunin ring finger 1 | O60291 |
| MGST2 | Microsomal glutathione S-transferase 2 | Q99735 |
| MGST3 | Microsomal glutathione S-transferase 3 | O14880 |
| MIA2 | MIA SH3 domain ER export factor 2 | Q96PC5 |
| MIB2 | MIB E3 ubiquitin protein ligase 2 | Q96AX9 |
| MICB | MHC class I polypeptide-related sequence B | Q29980 |
| MICU1 | Mitochondrial calcium uptake 1 | Q9BPX6 |
| MICU2 | Mitochondrial calcium uptake 2 | Q8IYU8 |
| MID1IP1 | MID1 interacting protein 1 | Q9NPA3 |
| MID2 | Midline 2 | Q9UJV3 |
| MIEF1 | Mitochondrial elongation factor 1 | Q9NQG6 |
| MIEF2 | Mitochondrial elongation factor 2 | Q96C03 |
| MIEN1 | Migration and invasion enhancer 1 | Q9BRT3 |
| MIER1 | MIER1 transcriptional regulator | Q8N108 |
| MIER2 | MIER family member 2 | Q8N344 |
| MIER3 | MIER family member 3 | Q7Z3K6 |
| MIF | Macrophage migration inhibitory factor | P14174 |
| MIF4GD | MIF4G domain containing | A9UHW6 |
| MIIP | Migration and invasion inhibitory protein | Q5JXC2 |
| MINPP1 | Multiple inositol-polyphosphate phosphatase 1 | Q9UNW1 |
| MIOS | Meiosis regulator for oocyte development | Q9NXC5 |
| MIPEP | Mitochondrial intermediate peptidase | Q99797 |
| MIS12 | MIS12 kinetochore complex component | Q9H081 |
| MIS18A | MIS18 kinetochore protein A | Q9NYP9 |
| MIS18BP1 | MIS18 binding protein 1 | Q6P0N0 |
| MITD1 | Microtubule interacting and trafficking domain containing 1 | Q8WV92 |
| MIXL1 | Mix paired-like homeobox | Q9H2W2 |
| MKKS | MKKS centrosomal shuttling protein | Q9NPJ1 |
| MKKS | MKKS centrosomal shuttling protein |  |
| MKKS | MKKS centrosomal shuttling protein |  |
| MKNK1 | MAPK interacting serine/threonine kinase 1 | Q9BUB5 |
| MKNK2 | MAPK interacting serine/threonine kinase 2 | Q9HBH9 |
| MKRN1 | Makorin ring finger protein 1 | Q9UHC7 |
| MKRN2 | Makorin ring finger protein 2 | Q9H000 |
| MLC1 | Modulator of VRAC current 1 | Q15049 |
| MLEC | Malectin | Q14165 |
| MLF1 | Myeloid leukemia factor 1 | P58340 |
| MLF2 | Myeloid leukemia factor 2 | Q15773 |
| MLH1 | MutL homolog 1 | P40692 |
| MLH3 | MutL homolog 3 | Q9UHC1 |
| MLKL | Mixed lineage kinase domain like pseudokinase | Q8NB16 |
| MLLT10 | MLLT10 histone lysine methyltransferase DOT1L cofactor | P55197 |
| MLLT11 | MLLT11 transcription factor 7 cofactor | Q13015 |
| MLLT3 | MLLT3 super elongation complex subunit | P42568 |
| MLST8 | MTOR associated protein, LST8 homolog | Q9BVC4 |
| MLX | MAX dimerization protein MLX | Q9UH92 |
| MLXIP | MLX interacting protein | Q9HAP2 |
| MMAA | Metabolism of cobalamin associated A | Q8IVH4 |
| MMAB | Metabolism of cobalamin associated B | Q96EY8 |
| MMADHC | Metabolism of cobalamin associated D | Q9H3L0 |
| MMD | Monocyte to macrophage differentiation associated | Q15546 |
| MMEL1 | Membrane metalloendopeptidase like 1 | Q495T6 |
| MMGT1 | Membrane magnesium transporter 1 | Q8N4V1 |
| MMP28 | Matrix metallopeptidase 28 | Q9H239 |
| MMS19 | MMS19 homolog, cytosolic iron-sulfur assembly component | Q96T76 |
| MNAT1 | MNAT1 component of CDK activating kinase | P51948 |
| MNDA | Myeloid cell nuclear differentiation antigen | P41218 |
| MOAP1 | Modulator of apoptosis 1 | Q96BY2 |
| MOB1A | MOB kinase activator 1A | Q9H8S9 |
| MOB2 | MOB kinase activator 2 | Q70IA6 |
| MOB3A | MOB kinase activator 3A | Q96BX8 |
| MOB3C | MOB kinase activator 3C | Q70IA8 |
| MOB4 | MOB family member 4, phocein | Q9Y3A3 |
| MOCS2 | Molybdenum cofactor synthesis 2 | O96007, O96033 |
| MOCS3 | Molybdenum cofactor synthesis 3 | O95396 |
| MOGS | Mannosyl-oligosaccharide glucosidase | Q13724 |
| MOK | MOK protein kinase | Q9UQ07 |
| MON1A | MON1 homolog A, secretory trafficking associated | Q86VX9 |
| MON1B | MON1 homolog B, secretory trafficking associated | Q7L1V2 |
| MORC2 | MORC family CW-type zinc finger 2 | Q9Y6X9 |
| MORF4L1 | Mortality factor 4 like 1 | Q9UBU8 |
| MORF4L2 | Mortality factor 4 like 2 | Q15014 |
| MORN2 | MORN repeat containing 2 | Q502X0 |
| MORN4 | MORN repeat containing 4 | Q8NDC4 |
| MOSPD1 | Motile sperm domain containing 1 | Q9UJG1 |
| MOSPD2 | Motile sperm domain containing 2 | Q8NHP6 |
| MOSPD3 | Motile sperm domain containing 3 | O75425 |
| MOV10 | Mov10 RISC complex RNA helicase | Q9HCE1 |
| MPC1 | Mitochondrial pyruvate carrier 1 | Q9Y5U8 |
| MPC2 | Mitochondrial pyruvate carrier 2 | O95563 |
| MPDU1 | Mannose-P-dolichol utilization defect 1 | O75352 |
| MPG | N-methylpurine DNA glycosylase | P29372 |
| MPHOSPH6 | M-phase phosphoprotein 6 | Q99547 |
| MPHOSPH9 | M-phase phosphoprotein 9 | Q99550 |
| MPI | Mannose phosphate isomerase | P34949 |
| MPLKIP | M-phase specific PLK1 interacting protein | Q8TAP9 |
| MPND | MPN domain containing | Q8N594 |
| MPP1 | MAGUK p55 scaffold protein 1 | Q00013 |
| MPP7 | MAGUK p55 scaffold protein 7 | Q5T2T1 |
| MPPE1 | Metallophosphoesterase 1 | Q53F39 |
| MPRIP | Myosin phosphatase Rho interacting protein | Q6WCQ1 |
| MPST | Mercaptopyruvate sulfurtransferase | P25325 |
| MPV17 | Mitochondrial inner membrane protein MPV17 | P39210 |
| MPV17L2 | MPV17 mitochondrial inner membrane protein like 2 | Q567V2 |
| MPZL1 | Myelin protein zero like 1 | O95297 |
| MPZL3 | Myelin protein zero like 3 | Q6UWV2 |
| MR1 | Major histocompatibility complex, class I-related | Q95460 |
| MREG | Melanoregulin | Q8N565 |
| MRFAP1 | Morf4 family associated protein 1 | Q9Y605 |
| MRFAP1L1 | Morf4 family associated protein 1 like 1 | Q96HT8 |
| MRGBP | MRG domain binding protein | Q9NV56 |
| MRI1 | Methylthioribose-1-phosphate isomerase 1 | Q9BV20 |
| MRM1 | Mitochondrial rRNA methyltransferase 1 | Q6IN84 |
| MRPL1 | Mitochondrial ribosomal protein L1 | Q9BYD6 |
| MRPL10 | Mitochondrial ribosomal protein L10 | Q7Z7H8 |
| MRPL11 | Mitochondrial ribosomal protein L11 | Q9Y3B7 |
| MRPL12 | Mitochondrial ribosomal protein L12 | P52815 |
| MRPL13 | Mitochondrial ribosomal protein L13 | Q9BYD1 |
| MRPL14 | Mitochondrial ribosomal protein L14 | Q6P1L8 |
| MRPL15 | Mitochondrial ribosomal protein L15 | Q9P015 |
| MRPL16 | Mitochondrial ribosomal protein L16 | Q9NX20 |
| MRPL17 | Mitochondrial ribosomal protein L17 | Q9NRX2 |
| MRPL18 | Mitochondrial ribosomal protein L18 | Q9H0U6 |
| MRPL19 | Mitochondrial ribosomal protein L19 | P49406 |
| MRPL2 | Mitochondrial ribosomal protein L2 | Q5T653 |
| MRPL20 | Mitochondrial ribosomal protein L20 | Q9BYC9 |
| MRPL21 | Mitochondrial ribosomal protein L21 | Q7Z2W9 |
| MRPL22 | Mitochondrial ribosomal protein L22 | Q9NWU5 |
| MRPL23 | Mitochondrial ribosomal protein L23 | Q16540 |
| MRPL24 | Mitochondrial ribosomal protein L24 | Q96A35 |
| MRPL27 | Mitochondrial ribosomal protein L27 | Q9P0M9 |
| MRPL28 | Mitochondrial ribosomal protein L28 | Q13084 |
| MRPL3 | Mitochondrial ribosomal protein L3 | P09001 |
| MRPL30 | Mitochondrial ribosomal protein L30 | Q8TCC3 |
| MRPL32 | Mitochondrial ribosomal protein L32 | Q9BYC8 |
| MRPL33 | Mitochondrial ribosomal protein L33 | O75394 |
| MRPL34 | Mitochondrial ribosomal protein L34 | Q9BQ48 |
| MRPL35 | Mitochondrial ribosomal protein L35 | Q9NZE8 |
| MRPL36 | Mitochondrial ribosomal protein L36 | Q9P0J6 |
| MRPL37 | Mitochondrial ribosomal protein L37 | Q9BZE1 |
| MRPL38 | Mitochondrial ribosomal protein L38 | Q96DV4 |
| MRPL39 | Mitochondrial ribosomal protein L39 | Q9NYK5 |
| MRPL4 | Mitochondrial ribosomal protein L4 | Q9BYD3 |
| MRPL40 | Mitochondrial ribosomal protein L40 | Q9NQ50 |
| MRPL41 | Mitochondrial ribosomal protein L41 | Q8IXM3 |
| MRPL42 | Mitochondrial ribosomal protein L42 | Q9Y6G3 |
| MRPL43 | Mitochondrial ribosomal protein L43 | Q8N983 |
| MRPL44 | Mitochondrial ribosomal protein L44 | Q9H9J2 |
| MRPL46 | Mitochondrial ribosomal protein L46 | Q9H2W6 |
| MRPL47 | Mitochondrial ribosomal protein L47 | Q9HD33 |
| MRPL48 | Mitochondrial ribosomal protein L48 | Q96GC5 |
| MRPL49 | Mitochondrial ribosomal protein L49 | Q13405 |
| MRPL50 | Mitochondrial ribosomal protein L50 | Q8N5N7 |
| MRPL51 | Mitochondrial ribosomal protein L51 | Q4U2R6 |
| MRPL52 | Mitochondrial ribosomal protein L52 | Q86TS9 |
| MRPL53 | Mitochondrial ribosomal protein L53 | Q96EL3 |
| MRPL54 | Mitochondrial ribosomal protein L54 | Q6P161 |
| MRPL55 | Mitochondrial ribosomal protein L55 | Q7Z7F7 |
| MRPL57 | Mitochondrial ribosomal protein L57 | Q9BQC6 |
| MRPL9 | Mitochondrial ribosomal protein L9 | Q9BYD2 |
| MRPS10 | Mitochondrial ribosomal protein S10 | P82664 |
| MRPS11 | Mitochondrial ribosomal protein S11 | P82912 |
| MRPS12 | Mitochondrial ribosomal protein S12 | O15235 |
| MRPS14 | Mitochondrial ribosomal protein S14 | O60783 |
| MRPS15 | Mitochondrial ribosomal protein S15 | P82914 |
| MRPS16 | Mitochondrial ribosomal protein S16 | Q9Y3D3 |
| MRPS17 | Mitochondrial ribosomal protein S17 | Q9Y2R5 |
| MRPS18A | Mitochondrial ribosomal protein S18A | Q9NVS2 |
| MRPS18B | Mitochondrial ribosomal protein S18B | Q9Y676 |
| MRPS18C | Mitochondrial ribosomal protein S18C | Q9Y3D5 |
| MRPS2 | Mitochondrial ribosomal protein S2 | Q9Y399 |
| MRPS21 | Mitochondrial ribosomal protein S21 | P82921 |
| MRPS22 | Mitochondrial ribosomal protein S22 | P82650 |
| MRPS23 | Mitochondrial ribosomal protein S23 | Q9Y3D9 |
| MRPS24 | Mitochondrial ribosomal protein S24 | Q96EL2 |
| MRPS25 | Mitochondrial ribosomal protein S25 | P82663 |
| MRPS26 | Mitochondrial ribosomal protein S26 | Q9BYN8 |
| MRPS27 | Mitochondrial ribosomal protein S27 | Q92552 |
| MRPS28 | Mitochondrial ribosomal protein S28 | Q9Y2Q9 |
| MRPS30 | Mitochondrial ribosomal protein S30 | Q9NP92 |
| MRPS31 | Mitochondrial ribosomal protein S31 | Q92665 |
| MRPS33 | Mitochondrial ribosomal protein S33 | Q9Y291 |
| MRPS34 | Mitochondrial ribosomal protein S34 | P82930 |
| MRPS35 | Mitochondrial ribosomal protein S35 | P82673 |
| MRPS36 | Mitochondrial ribosomal protein S36 | P82909 |
| MRPS5 | Mitochondrial ribosomal protein S5 | P82675 |
| MRPS6 | Mitochondrial ribosomal protein S6 | P82932 |
| MRPS7 | Mitochondrial ribosomal protein S7 | Q9Y2R9 |
| MRPS9 | Mitochondrial ribosomal protein S9 | P82933 |
| MRRF | Mitochondrial ribosome recycling factor | Q96E11 |
| MRS2 | Magnesium transporter MRS2 | Q9HD23 |
| MRTO4 | MRT4 homolog, ribosome maturation factor | Q9UKD2 |
| MS4A1 | Membrane spanning 4-domains A1 | P11836 |
| MS4A6A | Membrane spanning 4-domains A6A | Q9H2W1 |
| MSANTD3 | Myb/SANT DNA binding domain containing 3 | Q96H12 |
| MSANTD4 | Myb/SANT DNA binding domain containing 4 with coiled-coils | Q8NCY6 |
| MSC | Musculin | O60682 |
| MSH2 | MutS homolog 2 | P43246 |
| MSH6 | MutS homolog 6 | P52701 |
| MSL1 | MSL complex subunit 1 | Q68DK7 |
| MSL2 | MSL complex subunit 2 | Q9HCI7 |
| MSL3 | MSL complex subunit 3 | Q8N5Y2 |
| MSMO1 | Methylsterol monooxygenase 1 | Q15800 |
| MSN | Moesin | P26038 |
| MSRB1 | Methionine sulfoxide reductase B1 | Q9NZV6 |
| MSTO1 | Misato mitochondrial distribution and morphology regulator 1 | Q9BUK6 |
| MT1E | Metallothionein 1E | P04732 |
| MT1F | Metallothionein 1F | P04733 |
| MT1X | Metallothionein 1X | P80297 |
| MT2A | Metallothionein 2A | P02795 |
| MT3 | Metallothionein 3 | P25713 |
| MTA2 | Metastasis associated 1 family member 2 | O94776 |
| MTA3 | Metastasis associated 1 family member 3 | Q9BTC8 |
| MTAP | Methylthioadenosine phosphorylase | Q13126 |
| MTCH2 | Mitochondrial carrier 2 | Q9Y6C9 |
| MTDH | Metadherin | Q86UE4 |
| MTERF1 | Mitochondrial transcription termination factor 1 | Q99551 |
| MTERF2 | Mitochondrial transcription termination factor 2 | Q49AM1 |
| MTERF3 | Mitochondrial transcription termination factor 3 | Q96E29 |
| MTERF4 | Mitochondrial transcription termination factor 4 | Q7Z6M4 |
| MTF1 | Metal regulatory transcription factor 1 | Q14872 |
| MTF2 | Metal response element binding transcription factor 2 | Q9Y483 |
| MTFMT | Mitochondrial methionyl-tRNA formyltransferase | Q96DP5 |
| MTFP1 | Mitochondrial fission process 1 | Q9UDX5 |
| MTFR1L | Mitochondrial fission regulator 1 like | Q9H019 |
| MTG1 | Mitochondrial ribosome associated GTPase 1 | Q9BT17 |
| MTG2 | Mitochondrial ribosome associated GTPase 2 | Q9H4K7 |
| MTHFD1 | Methylenetetrahydrofolate dehydrogenase, cyclohydrolase and formyltetrahydrofolate synthetase 1 | P11586 |
| MTHFD2 | Methylenetetrahydrofolate dehydrogenase (NADP+ dependent) 2, methenyltetrahydrofolate cyclohydrolase | P13995 |
| MTHFR | Methylenetetrahydrofolate reductase | P42898 |
| MTHFS | Methenyltetrahydrofolate synthetase | P49914 |
| MTHFSD | Methenyltetrahydrofolate synthetase domain containing | Q2M296 |
| MTIF3 | Mitochondrial translational initiation factor 3 | Q9H2K0 |
| MTM1 | Myotubularin 1 | Q13496 |
| MTMR1 | Myotubularin related protein 1 | Q13613 |
| MTMR10 | Myotubularin related protein 10 | Q9NXD2 |
| MTMR12 | Myotubularin related protein 12 | Q9C0I1 |
| MTMR14 | Myotubularin related protein 14 | Q8NCE2 |
| MTMR2 | Myotubularin related protein 2 | Q13614 |
| MTMR3 | Myotubularin related protein 3 | Q13615 |
| MTMR4 | Myotubularin related protein 4 | Q9NYA4 |
| MTMR6 | Myotubularin related protein 6 | Q9Y217 |
| MTMR8 | Myotubularin related protein 8 | Q96EF0 |
| MTPAP | Mitochondrial poly(A) polymerase | Q9NVV4 |
| MTPN | Myotrophin | P58546 |
| MTRF1 | Mitochondrial translation release factor 1 | O75570 |
| MTRF1L | Mitochondrial translation release factor 1 like | Q9UGC7 |
| MTUS1 | Microtubule associated scaffold protein 1 | Q9ULD2 |
| MTX2 | Metaxin 2 | O75431 |
| MUL1 | Mitochondrial E3 ubiquitin protein ligase 1 | Q969V5 |
| MUS81 | MUS81 structure-specific endonuclease subunit | Q96NY9 |
| MUTYH | MutY DNA glycosylase | Q9UIF7 |
| MVB12A | Multivesicular body subunit 12A | Q96EY5 |
| MVB12B | Multivesicular body subunit 12B | Q9H7P6 |
| MVD | Mevalonate diphosphate decarboxylase | P53602 |
| MVK | Mevalonate kinase | Q03426 |
| MVP | Major vault protein | Q14764 |
| MX1 | MX dynamin like GTPase 1 | P20591 |
| MX2 | MX dynamin like GTPase 2 | P20592 |
| MXD1 | MAX dimerization protein 1 | Q05195 |
| MXD4 | MAX dimerization protein 4 | Q14582 |
| MXI1 | MAX interactor 1, dimerization protein | P50539 |
| MXRA8 | Matrix remodeling associated 8 | Q9BRK3 |
| MYADM | Myeloid associated differentiation marker | Q96S97 |
| MYBBP1A | MYB binding protein 1a | Q9BQG0 |
| MYC | MYC proto-oncogene, bHLH transcription factor | P01106 |
| MYCBP | MYC binding protein | Q99417 |
| MYD88 | MYD88 innate immune signal transduction adaptor | Q99836 |
| MYDGF | Myeloid derived growth factor | Q969H8 |
| MYH9 | Myosin heavy chain 9 | P35579 |
| MYL12A | Myosin light chain 12A | P19105 |
| MYL12B | Myosin light chain 12B | O14950 |
| MYL5 | Myosin light chain 5 | Q02045 |
| MYL6 | Myosin light chain 6 | P60660 |
| MYL6B | Myosin light chain 6B | P14649 |
| MYLIP | Myosin regulatory light chain interacting protein | Q8WY64 |
| MYNN | Myoneurin | Q9NPC7 |
| MYO19 | Myosin XIX | Q96H55 |
| MYO1F | Myosin IF | O00160 |
| MYO6 | Myosin VI | Q9UM54 |
| MZB1 | Marginal zone B and B1 cell specific protein | Q8WU39 |
| MZF1 | Myeloid zinc finger 1 | P28698 |
| MZT1 | Mitotic spindle organizing protein 1 | Q08AG7 |
| MZT2B | Mitotic spindle organizing protein 2B | Q6NZ67 |
| N4BP1 | NEDD4 binding protein 1 | O75113 |
| N4BP2L1 | NEDD4 binding protein 2 like 1 | Q5TBK1 |
| N4BP2L2 | NEDD4 binding protein 2 like 2 | Q92802 |
| N6AMT1 | N-6 adenine-specific DNA methyltransferase 1 | Q9Y5N5 |
| NAA10 | N-alpha-acetyltransferase 10, NatA catalytic subunit | P41227 |
| NAA15 | N-alpha-acetyltransferase 15, NatA auxiliary subunit | Q9BXJ9 |
| NAA20 | N-alpha-acetyltransferase 20, NatB catalytic subunit | P61599 |
| NAA25 | N-alpha-acetyltransferase 25, NatB auxiliary subunit | Q14CX7 |
| NAA30 | N-alpha-acetyltransferase 30, NatC catalytic subunit | Q147X3 |
| NAA38 | N-alpha-acetyltransferase 38, NatC auxiliary subunit | Q9BRA0 |
| NAA40 | N-alpha-acetyltransferase 40, NatD catalytic subunit | Q86UY6 |
| NAA50 | N-alpha-acetyltransferase 50, NatE catalytic subunit | Q9GZZ1 |
| NAA60 | N-alpha-acetyltransferase 60, NatF catalytic subunit | Q9H7X0 |
| NAAA | N-acylethanolamine acid amidase | Q02083 |
| NAALADL1 | N-acetylated alpha-linked acidic dipeptidase like 1 | Q9UQQ1 |
| NAB1 | NGFI-A binding protein 1 | Q13506 |
| NAB2 | NGFI-A binding protein 2 | Q15742 |
| NABP1 | Nucleic acid binding protein 1 | Q96AH0 |
| NABP2 | Nucleic acid binding protein 2 | Q9BQ15 |
| NACA | Nascent polypeptide associated complex subunit alpha | E9PAV3, Q13765 |
| NADK | NAD kinase | O95544 |
| NADK2 | NAD kinase 2, mitochondrial | Q4G0N4 |
| NADSYN1 | NAD synthetase 1 | Q6IA69 |
| NAE1 | NEDD8 activating enzyme E1 subunit 1 | Q13564 |
| NAF1 | Nuclear assembly factor 1 ribonucleoprotein | Q96HR8 |
| NAGA | Alpha-N-acetylgalactosaminidase | P17050 |
| NAGK | N-acetylglucosamine kinase | Q9UJ70 |
| NAGLU | N-acetyl-alpha-glucosaminidase | P54802 |
| NAMPT | Nicotinamide phosphoribosyltransferase | P43490 |
| NANP | N-acetylneuraminic acid phosphatase | Q8TBE9 |
| NANS | N-acetylneuraminate synthase | Q9NR45 |
| NAP1L1 | Nucleosome assembly protein 1 like 1 | P55209 |
| NAP1L2 | Nucleosome assembly protein 1 like 2 | Q9ULW6 |
| NAP1L3 | Nucleosome assembly protein 1 like 3 | Q99457 |
| NAP1L4 | Nucleosome assembly protein 1 like 4 | Q99733 |
| NAP1L5 | Nucleosome assembly protein 1 like 5 | Q96NT1 |
| NAPA | NSF attachment protein alpha | P54920 |
| NAPB | NSF attachment protein beta | Q9H115 |
| NAPEPLD | N-acyl phosphatidylethanolamine phospholipase D | Q6IQ20 |
| NAPG | NSF attachment protein gamma | Q99747 |
| NAPRT | Nicotinate phosphoribosyltransferase | Q6XQN6 |
| NARF | Nuclear prelamin A recognition factor | Q9UHQ1 |
| NARS2 | Asparaginyl-tRNA synthetase 2, mitochondrial | Q96I59 |
| NASP | Nuclear autoantigenic sperm protein | P49321 |
| NAT1 | N-acetyltransferase 1 | P18440 |
| NAT9 | N-acetyltransferase 9 (putative) | Q9BTE0 |
| NBPF15 | NBPF member 15 | Q8N660 |
| NBPF3 | NBPF member 3 | Q9H094 |
| NBPF9 | NBPF member 9 | P0DPF3 |
| NBR1 | NBR1 autophagy cargo receptor | Q14596 |
| NCALD | Neurocalcin delta | P61601 |
| NCAM1 | Neural cell adhesion molecule 1 | P13591 |
| NCAPG2 | Non-SMC condensin II complex subunit G2 | Q86XI2 |
| NCAPH | Non-SMC condensin I complex subunit H | Q15003 |
| NCAPH2 | Non-SMC condensin II complex subunit H2 | Q6IBW4 |
| NCBP1 | Nuclear cap binding protein subunit 1 | Q09161 |
| NCBP2 | Nuclear cap binding protein subunit 2 | P52298 |
| NCBP3 | Nuclear cap binding subunit 3 | Q53F19 |
| NCDN | Neurochondrin | Q9UBB6 |
| NCEH1 | Neutral cholesterol ester hydrolase 1 | Q6PIU2 |
| NCF1 | Neutrophil cytosolic factor 1 | P14598 |
| NCF2 | Neutrophil cytosolic factor 2 | P19878 |
| NCF4 | Neutrophil cytosolic factor 4 | Q15080 |
| NCK1 | NCK adaptor protein 1 | P16333 |
| NCK2 | NCK adaptor protein 2 | O43639 |
| NCKIPSD | NCK interacting protein with SH3 domain | Q9NZQ3 |
| NCL | Nucleolin | P19338 |
| NCLN | Nicalin | Q969V3 |
| NCOA1 | Nuclear receptor coactivator 1 | Q15788 |
| NCOA3 | Nuclear receptor coactivator 3 | Q9Y6Q9 |
| NCOA4 | Nuclear receptor coactivator 4 | Q13772 |
| NCOA5 | Nuclear receptor coactivator 5 | Q9HCD5 |
| NCOR1 | Nuclear receptor corepressor 1 | O75376 |
| NCR3 | Natural cytotoxicity triggering receptor 3 | O14931 |
| NCSTN | Nicastrin | Q92542 |
| NDC1 | NDC1 transmembrane nucleoporin | Q9BTX1 |
| NDC80 | NDC80 kinetochore complex component | O14777 |
| NDE1 | NudE neurodevelopment protein 1 | Q9NXR1 |
| NDEL1 | NudE neurodevelopment protein 1 like 1 | Q9GZM8 |
| NDFIP1 | Nedd4 family interacting protein 1 | Q9BT67 |
| NDFIP2 | Nedd4 family interacting protein 2 | Q9NV92 |
| NDOR1 | NADPH dependent diflavin oxidoreductase 1 | Q9UHB4 |
| NDRG1 | N-myc downstream regulated 1 | Q92597 |
| NDRG2 | NDRG family member 2 | Q9UN36 |
| NDRG3 | NDRG family member 3 | Q9UGV2 |
| NDST2 | N-deacetylase and N-sulfotransferase 2 | P52849 |
| NDUFA1 | NADH:ubiquinone oxidoreductase subunit A1 | O15239 |
| NDUFA10 | NADH:ubiquinone oxidoreductase subunit A10 | O95299 |
| NDUFA11 | NADH:ubiquinone oxidoreductase subunit A11 | Q86Y39 |
| NDUFA12 | NADH:ubiquinone oxidoreductase subunit A12 | Q9UI09 |
| NDUFA13 | NADH:ubiquinone oxidoreductase subunit A13 | Q9P0J0 |
| NDUFA2 | NADH:ubiquinone oxidoreductase subunit A2 | O43678 |
| NDUFA3 | NADH:ubiquinone oxidoreductase subunit A3 | O95167 |
| NDUFA4 | NDUFA4 mitochondrial complex associated | O00483 |
| NDUFA5 | NADH:ubiquinone oxidoreductase subunit A5 | Q16718 |
| NDUFA6 | NADH:ubiquinone oxidoreductase subunit A6 | P56556 |
| NDUFA7 | NADH:ubiquinone oxidoreductase subunit A7 | O95182 |
| NDUFA8 | NADH:ubiquinone oxidoreductase subunit A8 | P51970 |
| NDUFA9 | NADH:ubiquinone oxidoreductase subunit A9 | Q16795 |
| NDUFAB1 | NADH:ubiquinone oxidoreductase subunit AB1 | O14561 |
| NDUFAF1 | NADH:ubiquinone oxidoreductase complex assembly factor 1 | Q9Y375 |
| NDUFAF2 | NADH:ubiquinone oxidoreductase complex assembly factor 2 | Q8N183 |
| NDUFAF4 | NADH:ubiquinone oxidoreductase complex assembly factor 4 | Q9P032 |
| NDUFAF5 | NADH:ubiquinone oxidoreductase complex assembly factor 5 | Q5TEU4 |
| NDUFAF6 | NADH:ubiquinone oxidoreductase complex assembly factor 6 | Q330K2 |
| NDUFAF7 | NADH:ubiquinone oxidoreductase complex assembly factor 7 | Q7L592 |
| NDUFB1 | NADH:ubiquinone oxidoreductase subunit B1 | O75438 |
| NDUFB10 | NADH:ubiquinone oxidoreductase subunit B10 | O96000 |
| NDUFB11 | NADH:ubiquinone oxidoreductase subunit B11 | Q9NX14 |
| NDUFB2 | NADH:ubiquinone oxidoreductase subunit B2 | O95178 |
| NDUFB3 | NADH:ubiquinone oxidoreductase subunit B3 | O43676 |
| NDUFB4 | NADH:ubiquinone oxidoreductase subunit B4 | O95168 |
| NDUFB5 | NADH:ubiquinone oxidoreductase subunit B5 | O43674 |
| NDUFB6 | NADH:ubiquinone oxidoreductase subunit B6 | O95139 |
| NDUFB7 | NADH:ubiquinone oxidoreductase subunit B7 | P17568 |
| NDUFB8 | NADH:ubiquinone oxidoreductase subunit B8 | O95169 |
| NDUFC2 | NADH:ubiquinone oxidoreductase subunit C2 | O95298 |
| NDUFS1 | NADH:ubiquinone oxidoreductase core subunit S1 | P28331 |
| NDUFS2 | NADH:ubiquinone oxidoreductase core subunit S2 | O75306 |
| NDUFS3 | NADH:ubiquinone oxidoreductase core subunit S3 | O75489 |
| NDUFS4 | NADH:ubiquinone oxidoreductase subunit S4 | O43181 |
| NDUFS5 | NADH:ubiquinone oxidoreductase subunit S5 | O43920 |
| NDUFS6 | NADH:ubiquinone oxidoreductase subunit S6 | O75380 |
| NDUFS7 | NADH:ubiquinone oxidoreductase core subunit S7 | O75251 |
| NDUFS8 | NADH:ubiquinone oxidoreductase core subunit S8 | O00217 |
| NDUFV1 | NADH:ubiquinone oxidoreductase core subunit V1 | P49821 |
| NDUFV2 | NADH:ubiquinone oxidoreductase core subunit V2 | P19404 |
| NDUFV3 | NADH:ubiquinone oxidoreductase subunit V3 | P56181 |
| NECAP1 | NECAP endocytosis associated 1 | Q8NC96 |
| NECAP2 | NECAP endocytosis associated 2 | Q9NVZ3 |
| NEDD1 | NEDD1 gamma-tubulin ring complex targeting factor | Q8NHV4 |
| NEDD8 | NEDD8 ubiquitin like modifier | Q15843 |
| NEDD9 | Neural precursor cell expressed, developmentally down-regulated 9 | Q14511 |
| NEFL | Neurofilament light chain | P07196 |
| NEIL1 | Nei like DNA glycosylase 1 | Q96FI4 |
| NEIL2 | Nei like DNA glycosylase 2 | Q969S2 |
| NEK1 | NIMA related kinase 1 | Q96PY6 |
| NEK3 | NIMA related kinase 3 | P51956 |
| NEK6 | NIMA related kinase 6 | Q9HC98 |
| NEK7 | NIMA related kinase 7 | Q8TDX7 |
| NEK8 | NIMA related kinase 8 | Q86SG6 |
| NEK9 | NIMA related kinase 9 | Q8TD19 |
| NELFA | Negative elongation factor complex member A | Q9H3P2 |
| NELFB | Negative elongation factor complex member B | Q8WX92 |
| NELFCD | Negative elongation factor complex member C/D | Q8IXH7 |
| NELFE | Negative elongation factor complex member E | P18615 |
| NELL2 | Neural EGFL like 2 | Q99435 |
| NEMF | Nuclear export mediator factor | O60524 |
| NEMP1 | Nuclear envelope integral membrane protein 1 | O14524 |
| NENF | Neudesin neurotrophic factor | Q9UMX5 |
| NEPRO | Nucleolus and neural progenitor protein | Q6NW34 |
| NET1 | Neuroepithelial cell transforming 1 | Q7Z628 |
| NF2 | NF2, moesin-ezrin-radixin like (MERLIN) tumor suppressor | P35240 |
| NFATC1 | Nuclear factor of activated T cells 1 | O95644 |
| NFATC2 | Nuclear factor of activated T cells 2 | Q13469 |
| NFATC2IP | Nuclear factor of activated T cells 2 interacting protein | Q8NCF5 |
| NFATC3 | Nuclear factor of activated T cells 3 | Q12968 |
| NFE2L1 | NFE2 like bZIP transcription factor 1 | Q14494 |
| NFE2L2 | NFE2 like bZIP transcription factor 2 | Q16236 |
| NFIA | Nuclear factor I A | Q12857 |
| NFIC | Nuclear factor I C | P08651 |
| NFKB1 | Nuclear factor kappa B subunit 1 | P19838 |
| NFKBIA | NFKB inhibitor alpha | P25963 |
| NFKBIB | NFKB inhibitor beta | Q15653 |
| NFKBID | NFKB inhibitor delta | Q8NI38 |
| NFKBIE | NFKB inhibitor epsilon | O00221 |
| NFKBIZ | NFKB inhibitor zeta | Q9BYH8 |
| NFS1 | NFS1 cysteine desulfurase | Q9Y697 |
| NFU1 | NFU1 iron-sulfur cluster scaffold | Q9UMS0 |
| NFX1 | Nuclear transcription factor, X-box binding 1 | Q12986 |
| NFYA | Nuclear transcription factor Y subunit alpha | P23511 |
| NFYB | Nuclear transcription factor Y subunit beta | P25208 |
| NFYC | Nuclear transcription factor Y subunit gamma | Q13952 |
| NGDN | Neuroguidin | Q8NEJ9 |
| NGLY1 | N-glycanase 1 | Q96IV0 |
| NGRN | Neugrin, neurite outgrowth associated | Q9NPE2 |
| NHEJ1 | Non-homologous end joining factor 1 | Q9H9Q4 |
| NHLRC1 | NHL repeat containing E3 ubiquitin protein ligase 1 | Q6VVB1 |
| NHLRC3 | NHL repeat containing 3 | Q5JS37 |
| NHP2 | NHP2 ribonucleoprotein | Q9NX24 |
| NHSL2 | NHS like 2 | Q5HYW2 |
| NICN1 | Nicolin 1, tubulin polyglutamylase complex subunit | Q9BSH3 |
| NIF3L1 | NGG1 interacting factor 3 like 1 | Q9GZT8 |
| NIFK | Nucleolar protein interacting with the FHA domain of MKI67 | Q9BYG3 |
| NIN | Ninein | Q8N4C6 |
| NINJ1 | Ninjurin 1 | Q92982 |
| NINJ2 | Ninjurin 2 | Q9NZG7 |
| NIP7 | Nucleolar pre-rRNA processing protein NIP7 | Q9Y221 |
| NIPA2 | NIPA magnesium transporter 2 | Q8N8Q9 |
| NIPAL3 | NIPA like domain containing 3 | Q6P499 |
| NIPSNAP1 | Nipsnap homolog 1 | Q9BPW8 |
| NIPSNAP3A | Nipsnap homolog 3A | Q9UFN0 |
| NISCH | Nischarin | Q9Y2I1 |
| NIT1 | Nitrilase 1 | Q86X76 |
| NIT2 | Nitrilase family member 2 | Q9NQR4 |
| NKAP | NFKB activating protein | Q8N5F7 |
| NKG7 | Natural killer cell granule protein 7 | Q16617 |
| NKIRAS1 | NFKB inhibitor interacting Ras like 1 | Q9NYS0 |
| NKIRAS2 | NFKB inhibitor interacting Ras like 2 | Q9NYR9 |
| NKRF | NFKB repressing factor | O15226 |
| NKTR | Natural killer cell triggering receptor | P30414 |
| NKX3-1 | NK3 homeobox 1 | Q99801 |
| NLRC5 | NLR family CARD domain containing 5 | Q86WI3 |
| NLRP1 | NLR family pyrin domain containing 1 | Q9C000 |
| NLRP3 | NLR family pyrin domain containing 3 |  |
| NMB | Neuromedin B | P08949 |
| NMD3 | NMD3 ribosome export adaptor | Q96D46 |
| NME1 | NME/NM23 nucleoside diphosphate kinase 1 | P15531 |
| NME2 | NME/NM23 nucleoside diphosphate kinase 2 | P22392 |
| NME3 | NME/NM23 nucleoside diphosphate kinase 3 | Q13232 |
| NME4 | NME/NM23 nucleoside diphosphate kinase 4 | O00746 |
| NME6 | NME/NM23 nucleoside diphosphate kinase 6 | O75414 |
| NME7 | NME/NM23 family member 7 | Q9Y5B8 |
| NMI | N-myc and STAT interactor | Q13287 |
| NMNAT1 | Nicotinamide nucleotide adenylyltransferase 1 | Q9HAN9 |
| NMNAT3 | Nicotinamide nucleotide adenylyltransferase 3 | Q96T66 |
| NMRAL1 | NmrA like redox sensor 1 | Q9HBL8 |
| NMRK1 | Nicotinamide riboside kinase 1 | Q9NWW6 |
| NMT1 | N-myristoyltransferase 1 | P30419 |
| NMT2 | N-myristoyltransferase 2 | O60551 |
| NMUR1 | Neuromedin U receptor 1 | Q9HB89 |
| NOA1 | Nitric oxide associated 1 | Q8NC60 |
| NOB1 | NIN1 (RPN12) binding protein 1 homolog | Q9ULX3 |
| NOC3L | NOC3 like DNA replication regulator | Q8WTT2 |
| NOC4L | Nucleolar complex associated 4 homolog | Q9BVI4 |
| NOD1 | Nucleotide binding oligomerization domain containing 1 | Q9Y239 |
| NOG | Noggin | Q13253 |
| NOL12 | Nucleolar protein 12 | Q9UGY1 |
| NOL4L | Nucleolar protein 4 like | Q96MY1 |
| NOL6 | Nucleolar protein 6 | Q9H6R4 |
| NOL7 | Nucleolar protein 7 | Q9UMY1 |
| NOL9 | Nucleolar protein 9 | Q5SY16 |
| NOLC1 | Nucleolar and coiled-body phosphoprotein 1 | Q14978 |
| NOMO2 | NODAL modulator 2 | Q5JPE7 |
| NONO | Non-POU domain containing octamer binding | Q15233 |
| NOP10 | NOP10 ribonucleoprotein | Q9NPE3 |
| NOP14 | NOP14 nucleolar protein | P78316 |
| NOP16 | NOP16 nucleolar protein | Q9Y3C1 |
| NOP2 | NOP2 nucleolar protein | P46087 |
| NOP58 | NOP58 ribonucleoprotein | Q9Y2X3 |
| NOSIP | Nitric oxide synthase interacting protein | Q9Y314 |
| NPC2 | NPC intracellular cholesterol transporter 2 | P61916 |
| NPDC1 | Neural proliferation, differentiation and control 1 | Q9NQX5 |
| NPEPL1 | Aminopeptidase like 1 | Q8NDH3 |
| NPL | N-acetylneuraminate pyruvate lyase | Q9BXD5 |
| NPLOC4 | NPL4 homolog, ubiquitin recognition factor | Q8TAT6 |
| NPM1 | Nucleophosmin 1 | P06748 |
| NPM3 | Nucleophosmin/nucleoplasmin 3 | O75607 |
| NPRL2 | NPR2 like, GATOR1 complex subunit | Q8WTW4 |
| NPTN | Neuroplastin | Q9Y639 |
| NQO1 | NAD(P)H quinone dehydrogenase 1 | P15559 |
| NQO2 | N-ribosyldihydronicotinamide:quinone reductase 2 | P16083 |
| NR1D1 | Nuclear receptor subfamily 1 group D member 1 | P20393 |
| NR1H2 | Nuclear receptor subfamily 1 group H member 2 | P55055 |
| NR1H3 | Nuclear receptor subfamily 1 group H member 3 | Q13133 |
| NR2C1 | Nuclear receptor subfamily 2 group C member 1 | P13056 |
| NR2C2AP | Nuclear receptor 2C2 associated protein | Q86WQ0 |
| NR2F6 | Nuclear receptor subfamily 2 group F member 6 | P10588 |
| NR3C1 | Nuclear receptor subfamily 3 group C member 1 | P04150 |
| NR3C2 | Nuclear receptor subfamily 3 group C member 2 | P08235 |
| NRAS | NRAS proto-oncogene, GTPase | P01111 |
| NRBF2 | Nuclear receptor binding factor 2 | Q96F24 |
| NRBP1 | Nuclear receptor binding protein 1 | Q9UHY1 |
| NRCAM | Neuronal cell adhesion molecule | Q92823 |
| NREP | Neuronal regeneration related protein | Q16612 |
| NRF1 | Nuclear respiratory factor 1 | Q16656 |
| NRM | Nurim | Q8IXM6 |
| NRSN2 | Neurensin 2 | Q9GZP1 |
| NSA2 | NSA2 ribosome biogenesis factor | O95478 |
| NSDHL | NAD(P) dependent steroid dehydrogenase-like | Q15738 |
| NSF | N-ethylmaleimide sensitive factor, vesicle fusing ATPase | P46459 |
| NSFL1C | NSFL1 cofactor | Q9UNZ2 |
| NSG1 | Neuronal vesicle trafficking associated 1 | P42857 |
| NSL1 | NSL1 component of MIS12 kinetochore complex | Q96IY1 |
| NSMCE1 | NSE1 homolog, SMC5-SMC6 complex component | Q8WV22 |
| NSMCE2 | NSE2 (MMS21) homolog, SMC5-SMC6 complex SUMO ligase | Q96MF7 |
| NSMCE4A | NSE4 homolog A, SMC5-SMC6 complex component | Q9NXX6 |
| NSRP1 | Nuclear speckle splicing regulatory protein 1 | Q9H0G5 |
| NSUN3 | NOP2/Sun RNA methyltransferase 3 | Q9H649 |
| NSUN4 | NOP2/Sun RNA methyltransferase 4 | Q96CB9 |
| NSUN5 | NOP2/Sun RNA methyltransferase 5 | Q96P11 |
| NSUN6 | NOP2/Sun RNA methyltransferase 6 | Q8TEA1 |
| NT5C | 5', 3'-nucleotidase, cytosolic | Q8TCD5 |
| NT5C2 | 5'-nucleotidase, cytosolic II | P49902 |
| NT5C3A | 5'-nucleotidase, cytosolic IIIA | Q9H0P0 |
| NT5C3B | 5'-nucleotidase, cytosolic IIIB | Q969T7 |
| NT5DC1 | 5'-nucleotidase domain containing 1 | Q5TFE4 |
| NT5E | 5'-nucleotidase ecto | P21589 |
| NTAN1 | N-terminal asparagine amidase | Q96AB6 |
| NTHL1 | Nth like DNA glycosylase 1 | P78549 |
| NTMT1 | N-terminal Xaa-Pro-Lys N-methyltransferase 1 | Q9BV86 |
| NTPCR | Nucleoside-triphosphatase, cancer-related | Q9BSD7 |
| NUAK2 | NUAK family kinase 2 | Q9H093 |
| NUB1 | Negative regulator of ubiquitin like proteins 1 | Q9Y5A7 |
| NUBP1 | NUBP iron-sulfur cluster assembly factor 1, cytosolic | P53384 |
| NUBP2 | NUBP iron-sulfur cluster assembly factor 2, cytosolic | Q9Y5Y2 |
| NUBPL | NUBP iron-sulfur cluster assembly factor, mitochondrial | Q8TB37 |
| NUCB1 | Nucleobindin 1 | Q02818 |
| NUCKS1 | Nuclear casein kinase and cyclin dependent kinase substrate 1 | Q9H1E3 |
| NUDC | Nuclear distribution C, dynein complex regulator | Q9Y266 |
| NUDCD1 | NudC domain containing 1 | Q96RS6 |
| NUDCD2 | NudC domain containing 2 | Q8WVJ2 |
| NUDT1 | Nudix hydrolase 1 | P36639 |
| NUDT13 | Nudix hydrolase 13 | Q86X67 |
| NUDT14 | Nudix hydrolase 14 | O95848 |
| NUDT15 | Nudix hydrolase 15 | Q9NV35 |
| NUDT16 | Nudix hydrolase 16 | Q96DE0 |
| NUDT16L1 | Nudix hydrolase 16 like 1 | Q9BRJ7 |
| NUDT18 | Nudix hydrolase 18 | Q6ZVK8 |
| NUDT2 | Nudix hydrolase 2 | P50583 |
| NUDT21 | Nudix hydrolase 21 | O43809 |
| NUDT22 | Nudix hydrolase 22 | Q9BRQ3 |
| NUDT3 | Nudix hydrolase 3 | O95989 |
| NUDT4 | Nudix hydrolase 4 | Q9NZJ9 |
| NUDT5 | Nudix hydrolase 5 | Q9UKK9 |
| NUDT6 | Nudix hydrolase 6 | P53370 |
| NUDT7 | Nudix hydrolase 7 | P0C024 |
| NUDT8 | Nudix hydrolase 8 | Q8WV74 |
| NUDT9 | Nudix hydrolase 9 | Q9BW91 |
| NUF2 | NUF2 component of NDC80 kinetochore complex | Q9BZD4 |
| NUFIP2 | Nuclear FMR1 interacting protein 2 | Q7Z417 |
| NUGGC | Nuclear GTPase, germinal center associated | Q68CJ6 |
| NUMA1 | Nuclear mitotic apparatus protein 1 | Q14980 |
| NUMB | NUMB endocytic adaptor protein | P49757 |
| NUP107 | Nucleoporin 107 | P57740 |
| NUP133 | Nucleoporin 133 | Q8WUM0 |
| NUP160 | Nucleoporin 160 | Q12769 |
| NUP188 | Nucleoporin 188 | Q5SRE5 |
| NUP210 | Nucleoporin 210 | Q8TEM1 |
| NUP35 | Nucleoporin 35 | Q8NFH5 |
| NUP37 | Nucleoporin 37 | Q8NFH4 |
| NUP43 | Nucleoporin 43 | Q8NFH3 |
| NUP50 | Nucleoporin 50 | Q9UKX7 |
| NUP54 | Nucleoporin 54 | Q7Z3B4 |
| NUP58 | Nucleoporin 58 | Q9BVL2 |
| NUP62 | Nucleoporin 62 | P37198 |
| NUP85 | Nucleoporin 85 | Q9BW27 |
| NUP88 | Nucleoporin 88 | Q99567 |
| NUP98 | Nucleoporin 98 and 96 precursor | P52948 |
| NUS1 | NUS1 dehydrodolichyl diphosphate synthase subunit | Q96E22 |
| NUTF2 | Nuclear transport factor 2 | P61970 |
| NVL | Nuclear VCP like | O15381 |
| NXF1 | Nuclear RNA export factor 1 | Q9UBU9 |
| NXPE3 | Neurexophilin and PC-esterase domain family member 3 | Q969Y0 |
| NXT1 | Nuclear transport factor 2 like export factor 1 | Q9UKK6 |
| NXT2 | Nuclear transport factor 2 like export factor 2 | Q9NPJ8 |
| OARD1 | O-acyl-ADP-ribose deacylase 1 | Q9Y530 |
| OAS1 | 2'-5'-oligoadenylate synthetase 1 | P00973 |
| OAS2 | 2'-5'-oligoadenylate synthetase 2 | P29728 |
| OASL | 2'-5'-oligoadenylate synthetase like | Q15646 |
| OAT | Ornithine aminotransferase | P04181 |
| OCEL1 | Occludin/ELL domain containing 1 | Q9H607 |
| OCIAD1 | OCIA domain containing 1 | Q9NX40 |
| OCIAD2 | OCIA domain containing 2 | Q56VL3 |
| ODC1 | Ornithine decarboxylase 1 | P11926 |
| ODF2 | Outer dense fiber of sperm tails 2 | Q5BJF6 |
| ODF2L | Outer dense fiber of sperm tails 2 like | Q9ULJ1 |
| ODF3B | Outer dense fiber of sperm tails 3B | A8MYP8 |
| ODR4 | Odr-4 GPCR localization factor homolog | Q5SWX8 |
| OGDH | Oxoglutarate dehydrogenase | Q02218 |
| OGFOD1 | 2-oxoglutarate and iron dependent oxygenase domain containing 1 | Q8N543 |
| OGFOD2 | 2-oxoglutarate and iron dependent oxygenase domain containing 2 | Q6N063 |
| OGFOD3 | 2-oxoglutarate and iron dependent oxygenase domain containing 3 | Q6PK18 |
| OGG1 | 8-oxoguanine DNA glycosylase | O15527 |
| OGT | O-linked N-acetylglucosamine (GlcNAc) transferase | O15294 |
| OLA1 | Obg like ATPase 1 | Q9NTK5 |
| OLFM2 | Olfactomedin 2 | O95897 |
| OMA1 | OMA1 zinc metallopeptidase | Q96E52 |
| OPA1 | OPA1 mitochondrial dynamin like GTPase | O60313 |
| OPA3 | Outer mitochondrial membrane lipid metabolism regulator OPA3 | Q9H6K4 |
| OPTN | Optineurin | Q96CV9 |
| OR4D1 | Olfactory receptor family 4 subfamily D member 1 | Q15615 |
| ORAI1 | ORAI calcium release-activated calcium modulator 1 | Q96D31 |
| ORAI2 | ORAI calcium release-activated calcium modulator 2 | Q96SN7 |
| ORAI3 | ORAI calcium release-activated calcium modulator 3 | Q9BRQ5 |
| ORC2 | Origin recognition complex subunit 2 | Q13416 |
| ORC3 | Origin recognition complex subunit 3 | Q9UBD5 |
| ORC4 | Origin recognition complex subunit 4 | O43929 |
| ORC5 | Origin recognition complex subunit 5 | O43913 |
| ORMDL1 | ORMDL sphingolipid biosynthesis regulator 1 | Q9P0S3 |
| ORMDL2 | ORMDL sphingolipid biosynthesis regulator 2 | Q53FV1 |
| ORMDL3 | ORMDL sphingolipid biosynthesis regulator 3 | Q8N138 |
| OS9 | OS9 endoplasmic reticulum lectin | Q13438 |
| OSBPL11 | Oxysterol binding protein like 11 | Q9BXB4 |
| OSBPL3 | Oxysterol binding protein like 3 | Q9H4L5 |
| OSBPL5 | Oxysterol binding protein like 5 | Q9H0X9 |
| OSBPL8 | Oxysterol binding protein like 8 | Q9BZF1 |
| OSCP1 | Organic solute carrier partner 1 | Q8WVF1 |
| OSER1 | Oxidative stress responsive serine rich 1 | Q9NX31 |
| OSGEP | O-sialoglycoprotein endopeptidase | Q9NPF4 |
| OSGEPL1 | O-sialoglycoprotein endopeptidase like 1 | Q9H4B0 |
| OSGIN2 | Oxidative stress induced growth inhibitor family member 2 | Q9Y236 |
| OSM | Oncostatin M | P13725 |
| OSTC | Oligosaccharyltransferase complex non-catalytic subunit | Q9NRP0 |
| OSTF1 | Osteoclast stimulating factor 1 | Q92882 |
| OSTM1 | Osteoclastogenesis associated transmembrane protein 1 | Q86WC4 |
| OTUB1 | OTU deubiquitinase, ubiquitin aldehyde binding 1 | Q96FW1 |
| OTUD5 | OTU deubiquitinase 5 | Q96G74 |
| OTUD6B | OTU deubiquitinase 6B | Q8N6M0 |
| OVCA2 | OVCA2 serine hydrolase domain containing | Q8WZ82 |
| OXA1L | OXA1L mitochondrial inner membrane protein | Q15070 |
| OXCT1 | 3-oxoacid CoA-transferase 1 | P55809 |
| OXLD1 | Oxidoreductase like domain containing 1 | Q5BKU9 |
| OXNAD1 | Oxidoreductase NAD binding domain containing 1 | Q96HP4 |
| OXR1 | Oxidation resistance 1 | Q8N573 |
| OXSM | 3-oxoacyl-ACP synthase, mitochondrial | Q9NWU1 |
| OXSR1 | Oxidative stress responsive kinase 1 | O95747 |
| OXT | Oxytocin/neurophysin I prepropeptide | P01178 |
| P2RX4 | Purinergic receptor P2X 4 | Q99571 |
| P2RX5 | Purinergic receptor P2X 5 | Q93086 |
| P2RY10 | P2Y receptor family member 10 | O00398 |
| P2RY14 | Purinergic receptor P2Y14 | Q15391 |
| P2RY8 | P2Y receptor family member 8 | Q86VZ1 |
| P3H1 | Prolyl 3-hydroxylase 1 | Q32P28 |
| P3H4 | Prolyl 3-hydroxylase family member 4 (inactive) | Q92791 |
| P4HA1 | Prolyl 4-hydroxylase subunit alpha 1 | P13674 |
| P4HB | Prolyl 4-hydroxylase subunit beta | P07237 |
| P4HTM | Prolyl 4-hydroxylase, transmembrane | Q9NXG6 |
| PA2G4 | Proliferation-associated 2G4 | Q9UQ80 |
| PAAF1 | Proteasomal ATPase associated factor 1 | Q9BRP4 |
| PABPC1 | Poly(A) binding protein cytoplasmic 1 | P11940 |
| PABPC4 | Poly(A) binding protein cytoplasmic 4 | Q13310 |
| PABPN1 | Poly(A) binding protein nuclear 1 | Q86U42 |
| PACRGL | Parkin coregulated like | Q8N7B6 |
| PACS1 | Phosphofurin acidic cluster sorting protein 1 | Q6VY07 |
| PACSIN2 | Protein kinase C and casein kinase substrate in neurons 2 | Q9UNF0 |
| PADI4 | Peptidyl arginine deiminase 4 | Q9UM07 |
| PAF1 | PAF1 homolog, Paf1/RNA polymerase II complex component | Q8N7H5 |
| PAFAH1B1 | Platelet activating factor acetylhydrolase 1b regulatory subunit 1 | P43034 |
| PAFAH1B2 | Platelet activating factor acetylhydrolase 1b catalytic subunit 2 | P68402 |
| PAFAH1B3 | Platelet activating factor acetylhydrolase 1b catalytic subunit 3 | Q15102 |
| PAFAH2 | Platelet activating factor acetylhydrolase 2 | Q99487 |
| PAG1 | Phosphoprotein membrane anchor with glycosphingolipid microdomains 1 | Q9NWQ8 |
| PAGR1 | PAXIP1 associated glutamate rich protein 1 | Q9BTK6 |
| PAICS | Phosphoribosylaminoimidazole carboxylase and phosphoribosylaminoimidazolesuccinocarboxamide synthase | P22234 |
| PAIP1 | Poly(A) binding protein interacting protein 1 | Q9H074 |
| PAIP2 | Poly(A) binding protein interacting protein 2 | Q9BPZ3 |
| PAIP2B | Poly(A) binding protein interacting protein 2B | Q9ULR5 |
| PAK1 | P21 (RAC1) activated kinase 1 | Q13153 |
| PAK1IP1 | PAK1 interacting protein 1 | Q9NWT1 |
| PAK2 | P21 (RAC1) activated kinase 2 | Q13177 |
| PAK4 | P21 (RAC1) activated kinase 4 | O96013 |
| PAK6 | P21 (RAC1) activated kinase 6 | Q9NQU5 |
| PALB2 | Partner and localizer of BRCA2 | Q86YC2 |
| PAM | Peptidylglycine alpha-amidating monooxygenase | P19021 |
| PAM16 | Presequence translocase associated motor 16 | Q9Y3D7 |
| PANK2 | Pantothenate kinase 2 | Q9BZ23 |
| PANX1 | Pannexin 1 | Q96RD7 |
| PAPOLA | Poly(A) polymerase alpha | P51003 |
| PAPOLG | Poly(A) polymerase gamma | Q9BWT3 |
| PAPSS1 | 3'-phosphoadenosine 5'-phosphosulfate synthase 1 | O43252 |
| PAQR7 | Progestin and adipoQ receptor family member 7 | Q86WK9 |
| PAQR8 | Progestin and adipoQ receptor family member 8 | Q8TEZ7 |
| PARD6A | Par-6 family cell polarity regulator alpha | Q9NPB6 |
| PARD6B | Par-6 family cell polarity regulator beta | Q9BYG5 |
| PARG | Poly(ADP-ribose) glycohydrolase | Q86W56 |
| PARK7 | Parkinsonism associated deglycase | Q99497 |
| PARL | Presenilin associated rhomboid like | Q9H300 |
| PARN | Poly(A)-specific ribonuclease | O95453 |
| PARP1 | Poly(ADP-ribose) polymerase 1 | P09874 |
| PARP11 | Poly(ADP-ribose) polymerase family member 11 | Q9NR21 |
| PARP15 | Poly(ADP-ribose) polymerase family member 15 | Q460N3 |
| PARP16 | Poly(ADP-ribose) polymerase family member 16 | Q8N5Y8 |
| PARP3 | Poly(ADP-ribose) polymerase family member 3 | Q9Y6F1 |
| PARP8 | Poly(ADP-ribose) polymerase family member 8 | Q8N3A8 |
| PARS2 | Prolyl-tRNA synthetase 2, mitochondrial | Q7L3T8 |
| PARVG | Parvin gamma | Q9HBI0 |
| PASK | PAS domain containing serine/threonine kinase | Q96RG2 |
| PATL1 | PAT1 homolog 1, processing body mRNA decay factor | Q86TB9 |
| PATL2 | PAT1 homolog 2 | C9JE40 |
| PATZ1 | POZ/BTB and AT hook containing zinc finger 1 | Q9HBE1 |
| PBDC1 | Polysaccharide biosynthesis domain containing 1 | Q9BVG4 |
| PBX4 | PBX homeobox 4 | Q9BYU1 |
| PBXIP1 | PBX homeobox interacting protein 1 | Q96AQ6 |
| PCBD1 | Pterin-4 alpha-carbinolamine dehydratase 1 | P61457 |
| PCBD2 | Pterin-4 alpha-carbinolamine dehydratase 2 | Q9H0N5 |
| PCBP1 | Poly(rC) binding protein 1 | Q15365 |
| PCBP2 | Poly(rC) binding protein 2 | Q15366 |
| PCBP4 | Poly(rC) binding protein 4 | P57723 |
| PCCA | Propionyl-CoA carboxylase subunit alpha | P05165 |
| PCCB | Propionyl-CoA carboxylase subunit beta | P05166 |
| PCED1A | PC-esterase domain containing 1A | Q9H1Q7 |
| PCED1B | PC-esterase domain containing 1B | Q96HM7 |
| PCGF1 | Polycomb group ring finger 1 | Q9BSM1 |
| PCGF3 | Polycomb group ring finger 3 | Q3KNV8 |
| PCGF5 | Polycomb group ring finger 5 | Q86SE9 |
| PCGF6 | Polycomb group ring finger 6 | Q9BYE7 |
| PCID2 | PCI domain containing 2 | Q5JVF3 |
| PCIF1 | Phosphorylated CTD interacting factor 1 | Q9H4Z3 |
| PCK2 | Phosphoenolpyruvate carboxykinase 2, mitochondrial | Q16822 |
| PCM1 | Pericentriolar material 1 | Q15154 |
| PCMT1 | Protein-L-isoaspartate (D-aspartate) O-methyltransferase | P22061 |
| PCMTD1 | Protein-L-isoaspartate (D-aspartate) O-methyltransferase domain containing 1 | Q96MG8 |
| PCMTD2 | Protein-L-isoaspartate (D-aspartate) O-methyltransferase domain containing 2 | Q9NV79 |
| PCNA | Proliferating cell nuclear antigen | P12004 |
| PCNP | PEST proteolytic signal containing nuclear protein | Q8WW12 |
| PCSK1N | Proprotein convertase subtilisin/kexin type 1 inhibitor | Q9UHG2 |
| PCSK5 | Proprotein convertase subtilisin/kexin type 5 | Q92824 |
| PCSK7 | Proprotein convertase subtilisin/kexin type 7 | Q16549 |
| PCTP | Phosphatidylcholine transfer protein | Q9UKL6 |
| PCYOX1 | Prenylcysteine oxidase 1 | Q9UHG3 |
| PCYOX1L | Prenylcysteine oxidase 1 like | Q8NBM8 |
| PCYT1A | Phosphate cytidylyltransferase 1A, choline | P49585 |
| PCYT2 | Phosphate cytidylyltransferase 2, ethanolamine | Q99447 |
| PDAP1 | PDGFA associated protein 1 | Q13442 |
| PDCD1 | Programmed cell death 1 | Q15116 |
| PDCD10 | Programmed cell death 10 | Q9BUL8 |
| PDCD11 | Programmed cell death 11 | Q14690 |
| PDCD2 | Programmed cell death 2 | Q16342 |
| PDCD2L | Programmed cell death 2 like | Q9BRP1 |
| PDCD4 | Programmed cell death 4 | Q53EL6 |
| PDCD5 | Programmed cell death 5 | O14737 |
| PDCD6 | Programmed cell death 6 | O75340 |
| PDCD6IP | Programmed cell death 6 interacting protein | Q8WUM4 |
| PDCD7 | Programmed cell death 7 | Q8N8D1 |
| PDCL | Phosducin like | Q13371 |
| PDCL3 | Phosducin like 3 | Q9H2J4 |
| PDE12 | Phosphodiesterase 12 | Q6L8Q7 |
| PDE4A | Phosphodiesterase 4A | P27815 |
| PDE4DIP | Phosphodiesterase 4D interacting protein | Q5VU43 |
| PDE6B | Phosphodiesterase 6B | P35913 |
| PDE6D | Phosphodiesterase 6D | O43924 |
| PDE6G | Phosphodiesterase 6G | P18545 |
| PDE7A | Phosphodiesterase 7A | Q13946 |
| PDF | Peptide deformylase, mitochondrial | Q9HBH1 |
| PDGFA | Platelet derived growth factor subunit A | P04085 |
| PDGFB | Platelet derived growth factor subunit B | P01127 |
| PDGFD | Platelet derived growth factor D | Q9GZP0 |
| PDHA1 | Pyruvate dehydrogenase E1 subunit alpha 1 | P08559 |
| PDHB | Pyruvate dehydrogenase E1 subunit beta | P11177 |
| PDHX | Pyruvate dehydrogenase complex component X | O00330 |
| PDIA3 | Protein disulfide isomerase family A member 3 | P30101 |
| PDIA4 | Protein disulfide isomerase family A member 4 | P13667 |
| PDIA6 | Protein disulfide isomerase family A member 6 | Q15084 |
| PDIK1L | PDLIM1 interacting kinase 1 like | Q8N165 |
| PDK1 | Pyruvate dehydrogenase kinase 1 | Q15118 |
| PDK2 | Pyruvate dehydrogenase kinase 2 | Q15119 |
| PDK3 | Pyruvate dehydrogenase kinase 3 | Q15120 |
| PDLIM1 | PDZ and LIM domain 1 | O00151 |
| PDLIM2 | PDZ and LIM domain 2 | Q96JY6 |
| PDLIM5 | PDZ and LIM domain 5 | Q96HC4 |
| PDLIM7 | PDZ and LIM domain 7 | Q9NR12 |
| PDP1 | Pyruvate dehydrogenase phosphatase catalytic subunit 1 | Q9P0J1 |
| PDP2 | Pyruvate dehydrogenase phosphatase catalytic subunit 2 | Q9P2J9 |
| PDPK1 | 3-phosphoinositide dependent protein kinase 1 | O15530 |
| PDPR | Pyruvate dehydrogenase phosphatase regulatory subunit | Q8NCN5 |
| PDRG1 | P53 and DNA damage regulated 1 | Q9NUG6 |
| PDS5A | PDS5 cohesin associated factor A | Q29RF7 |
| PDS5B | PDS5 cohesin associated factor B | Q9NTI5 |
| PDSS2 | Decaprenyl diphosphate synthase subunit 2 | Q86YH6 |
| PDXDC1 | Pyridoxal dependent decarboxylase domain containing 1 | Q6P996 |
| PDZD11 | PDZ domain containing 11 | Q5EBL8 |
| PEA15 | Proliferation and apoptosis adaptor protein 15 | Q15121 |
| PECAM1 | Platelet and endothelial cell adhesion molecule 1 | P16284 |
| PECR | Peroxisomal trans-2-enoyl-CoA reductase | Q9BY49 |
| PEF1 | Penta-EF-hand domain containing 1 | Q9UBV8 |
| PELI1 | Pellino E3 ubiquitin protein ligase 1 | Q96FA3 |
| PELI3 | Pellino E3 ubiquitin protein ligase family member 3 | Q8N2H9 |
| PELO | Pelota mRNA surveillance and ribosome rescue factor | Q9BRX2 |
| PEMT | Phosphatidylethanolamine N-methyltransferase | Q9UBM1 |
| PEPD | Peptidase D | P12955 |
| PER2 | Period circadian regulator 2 | O15055 |
| PER3 | Period circadian regulator 3 | P56645 |
| PERP | P53 apoptosis effector related to PMP22 | Q96FX8 |
| PES1 | Pescadillo ribosomal biogenesis factor 1 | O00541 |
| PEX1 | Peroxisomal biogenesis factor 1 | O43933 |
| PEX10 | Peroxisomal biogenesis factor 10 | O60683 |
| PEX11A | Peroxisomal biogenesis factor 11 alpha | O75192 |
| PEX11B | Peroxisomal biogenesis factor 11 beta | O96011 |
| PEX12 | Peroxisomal biogenesis factor 12 | O00623 |
| PEX13 | Peroxisomal biogenesis factor 13 | Q92968 |
| PEX14 | Peroxisomal biogenesis factor 14 | O75381 |
| PEX16 | Peroxisomal biogenesis factor 16 | Q9Y5Y5 |
| PEX19 | Peroxisomal biogenesis factor 19 | P40855 |
| PEX2 | Peroxisomal biogenesis factor 2 | P28328 |
| PEX26 | Peroxisomal biogenesis factor 26 | Q7Z412 |
| PEX3 | Peroxisomal biogenesis factor 3 | P56589 |
| PEX5 | Peroxisomal biogenesis factor 5 | P50542 |
| PEX6 | Peroxisomal biogenesis factor 6 | Q13608 |
| PEX7 | Peroxisomal biogenesis factor 7 | O00628 |
| PFAS | Phosphoribosylformylglycinamidine synthase | O15067 |
| PFDN1 | Prefoldin subunit 1 | O60925 |
| PFDN2 | Prefoldin subunit 2 | Q9UHV9 |
| PFDN4 | Prefoldin subunit 4 | Q9NQP4 |
| PFDN5 | Prefoldin subunit 5 | Q99471 |
| PFDN6 | Prefoldin subunit 6 | O15212 |
| PFKFB3 | 6-phosphofructo-2-kinase/fructose-2,6-biphosphatase 3 | Q16875 |
| PFKL | Phosphofructokinase, liver type | P17858 |
| PFKM | Phosphofructokinase, muscle | P08237 |
| PFKP | Phosphofructokinase, platelet | Q01813 |
| PFN1 | Profilin 1 | P07737 |
| PFN2 | Profilin 2 | P35080 |
| PGAM1 | Phosphoglycerate mutase 1 | P18669 |
| PGAM5 | PGAM family member 5, mitochondrial serine/threonine protein phosphatase | Q96HS1 |
| PGAP2 | Post-GPI attachment to proteins 2 | Q9UHJ9 |
| PGAP3 | Post-GPI attachment to proteins phospholipase 3 | Q96FM1 |
| PGBD2 | PiggyBac transposable element derived 2 | Q6P3X8 |
| PGD | Phosphogluconate dehydrogenase | P52209 |
| PGGT1B | Protein geranylgeranyltransferase type I subunit beta | P53609 |
| PGK1 | Phosphoglycerate kinase 1 | P00558 |
| PGLS | 6-phosphogluconolactonase | O95336 |
| PGLYRP2 | Peptidoglycan recognition protein 2 | Q96PD5 |
| PGM1 | Phosphoglucomutase 1 | P36871 |
| PGM2 | Phosphoglucomutase 2 | Q96G03 |
| PGM2L1 | Phosphoglucomutase 2 like 1 | Q6PCE3 |
| PGM3 | Phosphoglucomutase 3 | O95394 |
| PGPEP1 | Pyroglutamyl-peptidase I | Q9NXJ5 |
| PGRMC1 | Progesterone receptor membrane component 1 | O00264 |
| PGRMC2 | Progesterone receptor membrane component 2 | O15173 |
| PGS1 | Phosphatidylglycerophosphate synthase 1 | Q32NB8 |
| PHACTR2 | Phosphatase and actin regulator 2 | O75167 |
| PHACTR4 | Phosphatase and actin regulator 4 | Q8IZ21 |
| PHAX | Phosphorylated adaptor for RNA export | Q9H814 |
| PHB2 | Prohibitin 2 | Q99623 |
| PHC1 | Polyhomeotic homolog 1 | P78364 |
| PHC2 | Polyhomeotic homolog 2 | Q8IXK0 |
| PHC3 | Polyhomeotic homolog 3 | Q8NDX5 |
| PHF1 | PHD finger protein 1 | O43189 |
| PHF10 | PHD finger protein 10 | Q8WUB8 |
| PHF11 | PHD finger protein 11 | Q9UIL8 |
| PHF13 | PHD finger protein 13 | Q86YI8 |
| PHF14 | PHD finger protein 14 | O94880 |
| PHF19 | PHD finger protein 19 | Q5T6S3 |
| PHF2 | PHD finger protein 2 | O75151 |
| PHF20 | PHD finger protein 20 | Q9BVI0 |
| PHF20L1 | PHD finger protein 20 like 1 | A8MW92 |
| PHF21A | PHD finger protein 21A | Q96BD5 |
| PHF23 | PHD finger protein 23 | Q9BUL5 |
| PHF3 | PHD finger protein 3 | Q92576 |
| PHF5A | PHD finger protein 5A | Q7RTV0 |
| PHF6 | PHD finger protein 6 | Q8IWS0 |
| PHF7 | PHD finger protein 7 | Q9BWX1 |
| PHF8 | PHD finger protein 8 | Q9UPP1 |
| PHGDH | Phosphoglycerate dehydrogenase | O43175 |
| PHKA2 | Phosphorylase kinase regulatory subunit alpha 2 | P46019 |
| PHKB | Phosphorylase kinase regulatory subunit beta | Q93100 |
| PHKG2 | Phosphorylase kinase catalytic subunit gamma 2 | P15735 |
| PHLDA1 | Pleckstrin homology like domain family A member 1 | Q8WV24 |
| PHOSPHO2 | Phosphatase, orphan 2 | Q8TCD6 |
| PHPT1 | Phosphohistidine phosphatase 1 | Q9NRX4 |
| PHTF1 | Putative homeodomain transcription factor 1 | Q9UMS5 |
| PHTF2 | Putative homeodomain transcription factor 2 | Q8N3S3 |
| PHYH | Phytanoyl-CoA 2-hydroxylase | O14832 |
| PHYHD1 | Phytanoyl-CoA dioxygenase domain containing 1 | Q5SRE7 |
| PHYKPL | 5-phosphohydroxy-L-lysine phospho-lyase | Q8IUZ5 |
| PI16 | Peptidase inhibitor 16 | Q6UXB8 |
| PI4K2A | Phosphatidylinositol 4-kinase type 2 alpha | Q9BTU6 |
| PI4K2B | Phosphatidylinositol 4-kinase type 2 beta | Q8TCG2 |
| PI4KA | Phosphatidylinositol 4-kinase alpha | P42356 |
| PI4KB | Phosphatidylinositol 4-kinase beta | Q9UBF8 |
| PIAS1 | Protein inhibitor of activated STAT 1 | O75925 |
| PIAS2 | Protein inhibitor of activated STAT 2 | O75928 |
| PIAS3 | Protein inhibitor of activated STAT 3 | Q9Y6X2 |
| PIAS4 | Protein inhibitor of activated STAT 4 | Q8N2W9 |
| PIBF1 | Progesterone immunomodulatory binding factor 1 | Q8WXW3 |
| PICALM | Phosphatidylinositol binding clathrin assembly protein | Q13492 |
| PICK1 | Protein interacting with PRKCA 1 | Q9NRD5 |
| PIEZO1 | Piezo type mechanosensitive ion channel component 1 | Q92508 |
| PIGA | Phosphatidylinositol glycan anchor biosynthesis class A | P37287 |
| PIGB | Phosphatidylinositol glycan anchor biosynthesis class B | Q92521 |
| PIGC | Phosphatidylinositol glycan anchor biosynthesis class C | Q92535 |
| PIGF | Phosphatidylinositol glycan anchor biosynthesis class F | Q07326 |
| PIGG | Phosphatidylinositol glycan anchor biosynthesis class G | Q5H8A4 |
| PIGH | Phosphatidylinositol glycan anchor biosynthesis class H | Q14442 |
| PIGK | Phosphatidylinositol glycan anchor biosynthesis class K | Q92643 |
| PIGL | Phosphatidylinositol glycan anchor biosynthesis class L | Q9Y2B2 |
| PIGM | Phosphatidylinositol glycan anchor biosynthesis class M | Q9H3S5 |
| PIGN | Phosphatidylinositol glycan anchor biosynthesis class N | O95427 |
| PIGO | Phosphatidylinositol glycan anchor biosynthesis class O | Q8TEQ8 |
| PIGP | Phosphatidylinositol glycan anchor biosynthesis class P | P57054 |
| PIGQ | Phosphatidylinositol glycan anchor biosynthesis class Q | Q9BRB3 |
| PIGS | Phosphatidylinositol glycan anchor biosynthesis class S | Q96S52 |
| PIGT | Phosphatidylinositol glycan anchor biosynthesis class T | Q969N2 |
| PIGU | Phosphatidylinositol glycan anchor biosynthesis class U | Q9H490 |
| PIGV | Phosphatidylinositol glycan anchor biosynthesis class V | Q9NUD9 |
| PIGX | Phosphatidylinositol glycan anchor biosynthesis class X | Q8TBF5 |
| PIH1D1 | PIH1 domain containing 1 | Q9NWS0 |
| PIK3AP1 | Phosphoinositide-3-kinase adaptor protein 1 | Q6ZUJ8 |
| PIK3C2A | Phosphatidylinositol-4-phosphate 3-kinase catalytic subunit type 2 alpha | O00443 |
| PIK3C3 | Phosphatidylinositol 3-kinase catalytic subunit type 3 | Q8NEB9 |
| PIK3CB | Phosphatidylinositol-4,5-bisphosphate 3-kinase catalytic subunit beta | P42338 |
| PIK3CD | Phosphatidylinositol-4,5-bisphosphate 3-kinase catalytic subunit delta | O00329 |
| PIK3CG | Phosphatidylinositol-4,5-bisphosphate 3-kinase catalytic subunit gamma | P48736 |
| PIK3IP1 | Phosphoinositide-3-kinase interacting protein 1 | Q96FE7 |
| PIK3R1 | Phosphoinositide-3-kinase regulatory subunit 1 | P27986 |
| PIK3R3 | Phosphoinositide-3-kinase regulatory subunit 3 | Q92569 |
| PIK3R4 | Phosphoinositide-3-kinase regulatory subunit 4 | Q99570 |
| PIK3R5 | Phosphoinositide-3-kinase regulatory subunit 5 | Q8WYR1 |
| PIKFYVE | Phosphoinositide kinase, FYVE-type zinc finger containing | Q9Y2I7 |
| PILRA | Paired immunoglobin like type 2 receptor alpha | Q9UKJ1 |
| PILRB | Paired immunoglobin like type 2 receptor beta | Q9UKJ0 |
| PIM1 | Pim-1 proto-oncogene, serine/threonine kinase | P11309 |
| PIM2 | Pim-2 proto-oncogene, serine/threonine kinase | Q9P1W9 |
| PIM3 | Pim-3 proto-oncogene, serine/threonine kinase | Q86V86 |
| PIN1 | Peptidylprolyl cis/trans isomerase, NIMA-interacting 1 | Q13526 |
| PIN4 | Peptidylprolyl cis/trans isomerase, NIMA-interacting 4 | Q9Y237 |
| PINK1 | PTEN induced kinase 1 | Q9BXM7 |
| PINX1 | PIN2 (TERF1) interacting telomerase inhibitor 1 | Q96BK5 |
| PIP4K2A | Phosphatidylinositol-5-phosphate 4-kinase type 2 alpha | P48426 |
| PIP4K2B | Phosphatidylinositol-5-phosphate 4-kinase type 2 beta | P78356 |
| PIP4K2C | Phosphatidylinositol-5-phosphate 4-kinase type 2 gamma | Q8TBX8 |
| PIP5K1A | Phosphatidylinositol-4-phosphate 5-kinase type 1 alpha | Q99755 |
| PISD | Phosphatidylserine decarboxylase | Q9UG56 |
| PITHD1 | PITH domain containing 1 | Q9GZP4 |
| PITPNA | Phosphatidylinositol transfer protein alpha | Q00169 |
| PITPNB | Phosphatidylinositol transfer protein beta | P48739 |
| PITPNC1 | Phosphatidylinositol transfer protein cytoplasmic 1 | Q9UKF7 |
| PITPNM1 | Phosphatidylinositol transfer protein membrane associated 1 | O00562 |
| PITRM1 | Pitrilysin metallopeptidase 1 | Q5JRX3 |
| PJA2 | Praja ring finger ubiquitin ligase 2 | O43164 |
| PKIA | CAMP-dependent protein kinase inhibitor alpha | P61925 |
| PKIG | CAMP-dependent protein kinase inhibitor gamma | Q9Y2B9 |
| PKM | Pyruvate kinase M1/2 | P14618 |
| PKN1 | Protein kinase N1 | Q16512 |
| PKN2 | Protein kinase N2 | Q16513 |
| PKNOX1 | PBX/knotted 1 homeobox 1 | P55347 |
| PLA2G12A | Phospholipase A2 group XIIA | Q9BZM1 |
| PLA2G15 | Phospholipase A2 group XV | Q8NCC3 |
| PLA2G6 | Phospholipase A2 group VI | O60733 |
| PLAA | Phospholipase A2 activating protein | Q9Y263 |
| PLAC8 | Placenta associated 8 | Q9NZF1 |
| PLAG1 | PLAG1 zinc finger | Q6DJT9 |
| PLAGL1 | PLAG1 like zinc finger 1 | Q9UM63 |
| PLAGL2 | PLAG1 like zinc finger 2 | Q9UPG8 |
| PLAUR | Plasminogen activator, urokinase receptor | Q03405 |
| PLCB2 | Phospholipase C beta 2 | Q00722 |
| PLCD1 | Phospholipase C delta 1 | P51178 |
| PLCG2 | Phospholipase C gamma 2 | P16885 |
| PLCL2 | Phospholipase C like 2 | Q9UPR0 |
| PLCXD1 | Phosphatidylinositol specific phospholipase C X domain containing 1 | Q9NUJ7 |
| PLCXD2 | Phosphatidylinositol specific phospholipase C X domain containing 2 | Q0VAA5 |
| PLD3 | Phospholipase D family member 3 | Q8IV08 |
| PLD4 | Phospholipase D family member 4 | Q96BZ4 |
| PLD6 | Phospholipase D family member 6 | Q8N2A8 |
| PLEK | Pleckstrin | P08567 |
| PLEKHA1 | Pleckstrin homology domain containing A1 | Q9HB21 |
| PLEKHA2 | Pleckstrin homology domain containing A2 |  |
| PLEKHB1 | Pleckstrin homology domain containing B1 | Q9UF11 |
| PLEKHB2 | Pleckstrin homology domain containing B2 | Q96CS7 |
| PLEKHF1 | Pleckstrin homology and FYVE domain containing 1 | Q96S99 |
| PLEKHF2 | Pleckstrin homology and FYVE domain containing 2 | Q9H8W4 |
| PLEKHG3 | Pleckstrin homology and RhoGEF domain containing G3 | A1L390 |
| PLEKHJ1 | Pleckstrin homology domain containing J1 | Q9NW61 |
| PLEKHO1 | Pleckstrin homology domain containing O1 | Q53GL0 |
| PLEKHO2 | Pleckstrin homology domain containing O2 | Q8TD55 |
| PLGRKT | Plasminogen receptor with a C-terminal lysine | Q9HBL7 |
| PLIN2 | Perilipin 2 | Q99541 |
| PLIN3 | Perilipin 3 | O60664 |
| PLK3 | Polo like kinase 3 | Q9H4B4 |
| PLLP | Plasmolipin | Q9Y342 |
| PLOD1 | Procollagen-lysine,2-oxoglutarate 5-dioxygenase 1 | Q02809 |
| PLOD3 | Procollagen-lysine,2-oxoglutarate 5-dioxygenase 3 | O60568 |
| PLP2 | Proteolipid protein 2 | Q04941 |
| PLPP1 | Phospholipid phosphatase 1 | O14494 |
| PLPP2 | Phospholipid phosphatase 2 | O43688 |
| PLPP5 | Phospholipid phosphatase 5 | Q8NEB5 |
| PLPP6 | Phospholipid phosphatase 6 | Q8IY26 |
| PLRG1 | Pleiotropic regulator 1 | O43660 |
| PLSCR1 | Phospholipid scramblase 1 | O15162 |
| PLSCR3 | Phospholipid scramblase 3 | Q9NRY6 |
| PLTP | Phospholipid transfer protein | P55058 |
| PLXDC1 | Plexin domain containing 1 | Q8IUK5 |
| PLXNC1 | Plexin C1 | O60486 |
| PM20D1 | Peptidase M20 domain containing 1 | Q6GTS8 |
| PM20D2 | Peptidase M20 domain containing 2 | Q8IYS1 |
| PMAIP1 | Phorbol-12-myristate-13-acetate-induced protein 1 | Q13794 |
| PMEPA1 | Prostate transmembrane protein, androgen induced 1 | Q969W9 |
| PMF1 | Polyamine modulated factor 1 | Q6P1K2 |
| PML | PML nuclear body scaffold | P29590 |
| PMM1 | Phosphomannomutase 1 | Q92871 |
| PMM2 | Phosphomannomutase 2 | O15305 |
| PMPCA | Peptidase, mitochondrial processing subunit alpha | Q10713 |
| PMPCB | Peptidase, mitochondrial processing subunit beta | O75439 |
| PMS1 | PMS1 homolog 1, mismatch repair system component | P54277 |
| PMS2 | PMS1 homolog 2, mismatch repair system component | P54278 |
| PMVK | Phosphomevalonate kinase | Q15126 |
| PNKD | PNKD metallo-beta-lactamase domain containing | Q8N490 |
| PNKP | Polynucleotide kinase 3'-phosphatase | Q96T60 |
| PNMA1 | PNMA family member 1 | Q8ND90 |
| PNN | Pinin, desmosome associated protein | Q9H307 |
| PNO1 | Partner of NOB1 homolog | Q9NRX1 |
| PNP | Purine nucleoside phosphorylase | P00491 |
| PNPLA2 | Patatin like phospholipase domain containing 2 | Q96AD5 |
| PNPLA4 | Patatin like phospholipase domain containing 4 | P41247 |
| PNPLA6 | Patatin like phospholipase domain containing 6 | Q8IY17 |
| PNPLA8 | Patatin like phospholipase domain containing 8 | Q9NP80 |
| PNPO | Pyridoxamine 5'-phosphate oxidase | Q9NVS9 |
| PNPT1 | Polyribonucleotide nucleotidyltransferase 1 | Q8TCS8 |
| PNRC2 | Proline rich nuclear receptor coactivator 2 | Q9NPJ4 |
| POC1B | POC1 centriolar protein B | Q8TC44 |
| POC5 | POC5 centriolar protein | Q8NA72 |
| PODN | Podocan | Q7Z5L7 |
| POFUT1 | Protein O-fucosyltransferase 1 | Q9H488 |
| POGK | Pogo transposable element derived with KRAB domain | Q9P215 |
| POGLUT1 | Protein O-glucosyltransferase 1 | Q8NBL1 |
| POGZ | Pogo transposable element derived with ZNF domain | Q7Z3K3 |
| POLA2 | DNA polymerase alpha 2, accessory subunit | Q14181 |
| POLB | DNA polymerase beta | P06746 |
| POLD1 | DNA polymerase delta 1, catalytic subunit | P28340 |
| POLD2 | DNA polymerase delta 2, accessory subunit | P49005 |
| POLD3 | DNA polymerase delta 3, accessory subunit | Q15054 |
| POLD4 | DNA polymerase delta 4, accessory subunit | Q9HCU8 |
| POLDIP2 | DNA polymerase delta interacting protein 2 | Q9Y2S7 |
| POLDIP3 | DNA polymerase delta interacting protein 3 | Q9BY77 |
| POLE3 | DNA polymerase epsilon 3, accessory subunit | Q9NRF9 |
| POLE4 | DNA polymerase epsilon 4, accessory subunit | Q9NR33 |
| POLG2 | DNA polymerase gamma 2, accessory subunit | Q9UHN1 |
| POLH | DNA polymerase eta | Q9Y253 |
| POLI | DNA polymerase iota | Q9UNA4 |
| POLK | DNA polymerase kappa | Q9UBT6 |
| POLL | DNA polymerase lambda | Q9UGP5 |
| POLR1C | RNA polymerase I and III subunit C | O15160 |
| POLR1D | RNA polymerase I and III subunit D | P0DPB5, P0DPB6 |
| POLR1E | RNA polymerase I subunit E | Q9GZS1 |
| POLR2C | RNA polymerase II subunit C | P19387 |
| POLR2D | RNA polymerase II subunit D | O15514 |
| POLR2E | RNA polymerase II, I and III subunit E | P19388 |
| POLR2F | RNA polymerase II, I and III subunit F | P61218 |
| POLR2G | RNA polymerase II subunit G | P62487 |
| POLR2H | RNA polymerase II, I and III subunit H | P52434 |
| POLR2I | RNA polymerase II subunit I | P36954 |
| POLR2J | RNA polymerase II subunit J | P52435 |
| POLR2J2 | RNA polymerase II subunit J2 | Q9GZM3 |
| POLR2J3 | RNA polymerase II subunit J3 |  |
| POLR2J3 | RNA polymerase II subunit J3 |  |
| POLR2K | RNA polymerase II, I and III subunit K | P53803 |
| POLR2L | RNA polymerase II, I and III subunit L | P62875 |
| POLR2M | RNA polymerase II subunit M | P0CAP2, Q6EEV4 |
| POLR3B | RNA polymerase III subunit B | Q9NW08 |
| POLR3C | RNA polymerase III subunit C | Q9BUI4 |
| POLR3D | RNA polymerase III subunit D | P05423 |
| POLR3E | RNA polymerase III subunit E | Q9NVU0 |
| POLR3F | RNA polymerase III subunit F | Q9H1D9 |
| POLR3G | RNA polymerase III subunit G | O15318 |
| POLR3GL | RNA polymerase III subunit GL | Q9BT43 |
| POLR3H | RNA polymerase III subunit H | Q9Y535 |
| POLR3K | RNA polymerase III subunit K | Q9Y2Y1 |
| POLRMT | RNA polymerase mitochondrial | O00411 |
| POM121 | POM121 transmembrane nucleoporin | Q96HA1 |
| POMC | Proopiomelanocortin | P01189 |
| POMGNT2 | Protein O-linked mannose N-acetylglucosaminyltransferase 2 (beta 1,4-) | Q8NAT1 |
| POMK | Protein O-mannose kinase | Q9H5K3 |
| POMP | Proteasome maturation protein | Q9Y244 |
| POMT1 | Protein O-mannosyltransferase 1 | Q9Y6A1 |
| POMZP3 | POM121 and ZP3 fusion | Q6PJE2 |
| PON2 | Paraoxonase 2 | Q15165 |
| POP1 | POP1 homolog, ribonuclease P/MRP subunit | Q99575 |
| POP4 | POP4 homolog, ribonuclease P/MRP subunit | O95707 |
| POP5 | POP5 homolog, ribonuclease P/MRP subunit | Q969H6 |
| POP7 | POP7 homolog, ribonuclease P/MRP subunit | O75817 |
| POR | Cytochrome p450 oxidoreductase | P16435 |
| PORCN | Porcupine O-acyltransferase | Q9H237 |
| POT1 | Protection of telomeres 1 | Q9NUX5 |
| POTEG | POTE ankyrin domain family member G | Q6S5H5 |
| POU2F2 | POU class 2 homeobox 2 | P09086 |
| PPA1 | Inorganic pyrophosphatase 1 | Q15181 |
| PPA2 | Inorganic pyrophosphatase 2 | Q9H2U2 |
| PPAN | Peter pan homolog | Q9NQ55 |
| PPAN-P2RY11 | PPAN-P2RY11 readthrough |  |
| PPARD | Peroxisome proliferator activated receptor delta | Q03181 |
| PPAT | Phosphoribosyl pyrophosphate amidotransferase | Q06203 |
| PPCDC | Phosphopantothenoylcysteine decarboxylase | Q96CD2 |
| PPCS | Phosphopantothenoylcysteine synthetase | Q9HAB8 |
| PPDPF | Pancreatic progenitor cell differentiation and proliferation factor | Q9H3Y8 |
| PPFIA1 | PTPRF interacting protein alpha 1 | Q13136 |
| PPFIBP2 | PPFIA binding protein 2 | Q8ND30 |
| PPHLN1 | Periphilin 1 | Q8NEY8 |
| PPIA | Peptidylprolyl isomerase A | P62937 |
| PPIAL4A | Peptidylprolyl isomerase A like 4A | Q9Y536 |
| PPIAL4C | Peptidylprolyl isomerase A like 4C | A0A0B4J2A2 |
| PPIB | Peptidylprolyl isomerase B | P23284 |
| PPID | Peptidylprolyl isomerase D | Q08752 |
| PPIE | Peptidylprolyl isomerase E | Q9UNP9 |
| PPIF | Peptidylprolyl isomerase F | P30405 |
| PPIG | Peptidylprolyl isomerase G | Q13427 |
| PPIH | Peptidylprolyl isomerase H | O43447 |
| PPIL1 | Peptidylprolyl isomerase like 1 | Q9Y3C6 |
| PPIL2 | Peptidylprolyl isomerase like 2 | Q13356 |
| PPIL3 | Peptidylprolyl isomerase like 3 | Q9H2H8 |
| PPIL4 | Peptidylprolyl isomerase like 4 | Q8WUA2 |
| PPIP5K1 | Diphosphoinositol pentakisphosphate kinase 1 | Q6PFW1 |
| PPIP5K2 | Diphosphoinositol pentakisphosphate kinase 2 | O43314 |
| PPM1A | Protein phosphatase, Mg2+/Mn2+ dependent 1A | P35813 |
| PPM1B | Protein phosphatase, Mg2+/Mn2+ dependent 1B | O75688 |
| PPM1D | Protein phosphatase, Mg2+/Mn2+ dependent 1D | O15297 |
| PPM1F | Protein phosphatase, Mg2+/Mn2+ dependent 1F | P49593 |
| PPM1G | Protein phosphatase, Mg2+/Mn2+ dependent 1G | O15355 |
| PPM1K | Protein phosphatase, Mg2+/Mn2+ dependent 1K | Q8N3J5 |
| PPM1L | Protein phosphatase, Mg2+/Mn2+ dependent 1L | Q5SGD2 |
| PPM1M | Protein phosphatase, Mg2+/Mn2+ dependent 1M | Q96MI6 |
| PPM1N | Protein phosphatase, Mg2+/Mn2+ dependent 1N (putative) | Q8N819 |
| PPME1 | Protein phosphatase methylesterase 1 | Q9Y570 |
| PPOX | Protoporphyrinogen oxidase | P50336 |
| PPP1CA | Protein phosphatase 1 catalytic subunit alpha | P62136 |
| PPP1CB | Protein phosphatase 1 catalytic subunit beta | P62140 |
| PPP1CC | Protein phosphatase 1 catalytic subunit gamma | P36873 |
| PPP1R10 | Protein phosphatase 1 regulatory subunit 10 | Q96QC0 |
| PPP1R11 | Protein phosphatase 1 regulatory inhibitor subunit 11 | O60927 |
| PPP1R12A | Protein phosphatase 1 regulatory subunit 12A | O14974 |
| PPP1R12B | Protein phosphatase 1 regulatory subunit 12B | O60237 |
| PPP1R12C | Protein phosphatase 1 regulatory subunit 12C | Q9BZL4 |
| PPP1R14B | Protein phosphatase 1 regulatory inhibitor subunit 14B | Q96C90 |
| PPP1R15A | Protein phosphatase 1 regulatory subunit 15A | O75807 |
| PPP1R16B | Protein phosphatase 1 regulatory subunit 16B | Q96T49 |
| PPP1R18 | Protein phosphatase 1 regulatory subunit 18 | Q6NYC8 |
| PPP1R2 | Protein phosphatase 1 regulatory inhibitor subunit 2 | P41236 |
| PPP1R21 | Protein phosphatase 1 regulatory subunit 21 | Q6ZMI0 |
| PPP1R32 | Protein phosphatase 1 regulatory subunit 32 | Q7Z5V6 |
| PPP1R35 | Protein phosphatase 1 regulatory subunit 35 | Q8TAP8 |
| PPP1R3B | Protein phosphatase 1 regulatory subunit 3B | Q86XI6 |
| PPP1R7 | Protein phosphatase 1 regulatory subunit 7 | Q15435 |
| PPP1R8 | Protein phosphatase 1 regulatory subunit 8 | Q12972 |
| PPP2CA | Protein phosphatase 2 catalytic subunit alpha | P67775 |
| PPP2CB | Protein phosphatase 2 catalytic subunit beta | P62714 |
| PPP2R1A | Protein phosphatase 2 scaffold subunit Aalpha | P30153 |
| PPP2R1B | Protein phosphatase 2 scaffold subunit Abeta | P30154 |
| PPP2R2A | Protein phosphatase 2 regulatory subunit Balpha | P63151 |
| PPP2R2B | Protein phosphatase 2 regulatory subunit Bbeta | Q00005 |
| PPP2R3C | Protein phosphatase 2 regulatory subunit B''gamma | Q969Q6 |
| PPP2R5A | Protein phosphatase 2 regulatory subunit B'alpha | Q15172 |
| PPP2R5C | Protein phosphatase 2 regulatory subunit B'gamma | Q13362 |
| PPP2R5D | Protein phosphatase 2 regulatory subunit B'delta | Q14738 |
| PPP2R5E | Protein phosphatase 2 regulatory subunit B'epsilon | Q16537 |
| PPP3CA | Protein phosphatase 3 catalytic subunit alpha | Q08209 |
| PPP3CB | Protein phosphatase 3 catalytic subunit beta | P16298 |
| PPP3CC | Protein phosphatase 3 catalytic subunit gamma | P48454 |
| PPP4C | Protein phosphatase 4 catalytic subunit | P60510 |
| PPP4R1 | Protein phosphatase 4 regulatory subunit 1 | Q8TF05 |
| PPP4R2 | Protein phosphatase 4 regulatory subunit 2 | Q9NY27 |
| PPP4R3A | Protein phosphatase 4 regulatory subunit 3A | Q6IN85 |
| PPP5C | Protein phosphatase 5 catalytic subunit | P53041 |
| PPP6C | Protein phosphatase 6 catalytic subunit | O00743 |
| PPP6R2 | Protein phosphatase 6 regulatory subunit 2 | O75170 |
| PPRC1 | PPARG related coactivator 1 | Q5VV67 |
| PPT1 | Palmitoyl-protein thioesterase 1 | P50897 |
| PPT2 | Palmitoyl-protein thioesterase 2 | Q9UMR5 |
| PPWD1 | Peptidylprolyl isomerase domain and WD repeat containing 1 | Q96BP3 |
| PQBP1 | Polyglutamine binding protein 1 | O60828 |
| PRADC1 | Protease associated domain containing 1 | Q9BSG0 |
| PRAF2 | PRA1 domain family member 2 | O60831 |
| PRC1 | Protein regulator of cytokinesis 1 | O43663 |
| PRCC | Proline rich mitotic checkpoint control factor | Q92733 |
| PRCP | Prolylcarboxypeptidase | P42785 |
| PRDM1 | PR/SET domain 1 | O75626 |
| PRDM2 | PR/SET domain 2 | Q13029 |
| PRDM4 | PR/SET domain 4 | Q9UKN5 |
| PRDX1 | Peroxiredoxin 1 | Q06830 |
| PRDX2 | Peroxiredoxin 2 | P32119 |
| PRDX3 | Peroxiredoxin 3 | P30048 |
| PRDX4 | Peroxiredoxin 4 | Q13162 |
| PRDX5 | Peroxiredoxin 5 | P30044 |
| PRDX6 | Peroxiredoxin 6 | P30041 |
| PREB | Prolactin regulatory element binding | Q9HCU5 |
| PRELID1 | PRELI domain containing 1 | Q9Y255 |
| PRELID2 | PRELI domain containing 2 | Q8N945 |
| PRELID3B | PRELI domain containing 3B | Q9Y3B1 |
| PREP | Prolyl endopeptidase | P48147 |
| PREPL | Prolyl endopeptidase like | Q4J6C6 |
| PREX1 | Phosphatidylinositol-3,4,5-trisphosphate dependent Rac exchange factor 1 | Q8TCU6 |
| PRF1 | Perforin 1 | P14222 |
| PRICKLE3 | Prickle planar cell polarity protein 3 | O43900 |
| PRIM1 | DNA primase subunit 1 | P49642 |
| PRIM2 | DNA primase subunit 2 | P49643 |
| PRIMPOL | Primase and DNA directed polymerase | Q96LW4 |
| PRKAA1 | Protein kinase AMP-activated catalytic subunit alpha 1 | Q13131 |
| PRKAB1 | Protein kinase AMP-activated non-catalytic subunit beta 1 | Q9Y478 |
| PRKACB | Protein kinase cAMP-activated catalytic subunit beta | P22694 |
| PRKAG1 | Protein kinase AMP-activated non-catalytic subunit gamma 1 | P54619 |
| PRKAG2 | Protein kinase AMP-activated non-catalytic subunit gamma 2 | Q9UGJ0 |
| PRKAR1A | Protein kinase cAMP-dependent type I regulatory subunit alpha | P10644 |
| PRKAR1B | Protein kinase cAMP-dependent type I regulatory subunit beta | P31321 |
| PRKAR2A | Protein kinase cAMP-dependent type II regulatory subunit alpha | P13861 |
| PRKCB | Protein kinase C beta | P05771 |
| PRKCD | Protein kinase C delta | Q05655 |
| PRKCH | Protein kinase C eta | P24723 |
| PRKCI | Protein kinase C iota | P41743 |
| PRKCQ | Protein kinase C theta | Q04759 |
| PRKD2 | Protein kinase D2 | Q9BZL6 |
| PRKD3 | Protein kinase D3 | O94806 |
| PRKRA | Protein activator of interferon induced protein kinase EIF2AK2 | O75569 |
| PRKRIP1 | PRKR interacting protein 1 | Q9H875 |
| PRKX | Protein kinase cAMP-dependent X-linked catalytic subunit | P51817 |
| PRMT1 | Protein arginine methyltransferase 1 | Q99873 |
| PRMT2 | Protein arginine methyltransferase 2 | P55345 |
| PRMT3 | Protein arginine methyltransferase 3 | O60678 |
| PRMT5 | Protein arginine methyltransferase 5 | O14744 |
| PRMT6 | Protein arginine methyltransferase 6 | Q96LA8 |
| PRMT7 | Protein arginine methyltransferase 7 | Q9NVM4 |
| PRNP | Prion protein | P04156 |
| PROCR | Protein C receptor | Q9UNN8 |
| PROK2 | Prokineticin 2 | Q9HC23 |
| PROSER3 | Proline and serine rich 3 | Q2NL68 |
| PRPF18 | Pre-mRNA processing factor 18 | Q99633 |
| PRPF19 | Pre-mRNA processing factor 19 | Q9UMS4 |
| PRPF3 | Pre-mRNA processing factor 3 | O43395 |
| PRPF31 | Pre-mRNA processing factor 31 | Q8WWY3 |
| PRPF38A | Pre-mRNA processing factor 38A | Q8NAV1 |
| PRPF4 | Pre-mRNA processing factor 4 | O43172 |
| PRPF6 | Pre-mRNA processing factor 6 | O94906 |
| PRPS1 | Phosphoribosyl pyrophosphate synthetase 1 | P60891 |
| PRPS2 | Phosphoribosyl pyrophosphate synthetase 2 | P11908 |
| PRPSAP1 | Phosphoribosyl pyrophosphate synthetase associated protein 1 | Q14558 |
| PRPSAP2 | Phosphoribosyl pyrophosphate synthetase associated protein 2 | O60256 |
| PRR13 | Proline rich 13 | Q9NZ81 |
| PRR14 | Proline rich 14 | Q9BWN1 |
| PRR5 | Proline rich 5 | P85299 |
| PRR7 | Proline rich 7, synaptic | Q8TB68 |
| PRRC1 | Proline rich coiled-coil 1 | Q96M27 |
| PRRC2B | Proline rich coiled-coil 2B | Q5JSZ5 |
| PRRT3 | Proline rich transmembrane protein 3 | Q5FWE3 |
| PRSS21 | Serine protease 21 | Q9Y6M0 |
| PRSS35 | Serine protease 35 | Q8N3Z0 |
| PRSS57 | Serine protease 57 | Q6UWY2 |
| PSAP | Prosaposin | P07602 |
| PSAT1 | Phosphoserine aminotransferase 1 | Q9Y617 |
| PSD4 | Pleckstrin and Sec7 domain containing 4 | Q8NDX1 |
| PSEN1 | Presenilin 1 | P49768 |
| PSEN2 | Presenilin 2 | P49810 |
| PSENEN | Presenilin enhancer, gamma-secretase subunit | Q9NZ42 |
| PSIP1 | PC4 and SFRS1 interacting protein 1 | O75475 |
| PSKH1 | Protein serine kinase H1 | P11801 |
| PSMA1 | Proteasome 20S subunit alpha 1 | P25786 |
| PSMA2 | Proteasome 20S subunit alpha 2 | P25787 |
| PSMA3 | Proteasome 20S subunit alpha 3 | P25788 |
| PSMA4 | Proteasome 20S subunit alpha 4 | P25789 |
| PSMA5 | Proteasome 20S subunit alpha 5 | P28066 |
| PSMA6 | Proteasome 20S subunit alpha 6 | P60900 |
| PSMA7 | Proteasome 20S subunit alpha 7 | O14818 |
| PSMA8 | Proteasome 20S subunit alpha 8 | Q8TAA3 |
| PSMB1 | Proteasome 20S subunit beta 1 | P20618 |
| PSMB10 | Proteasome 20S subunit beta 10 | P40306 |
| PSMB2 | Proteasome 20S subunit beta 2 | P49721 |
| PSMB3 | Proteasome 20S subunit beta 3 | P49720 |
| PSMB4 | Proteasome 20S subunit beta 4 | P28070 |
| PSMB5 | Proteasome 20S subunit beta 5 | P28074 |
| PSMB6 | Proteasome 20S subunit beta 6 | P28072 |
| PSMB7 | Proteasome 20S subunit beta 7 | Q99436 |
| PSMB8 | Proteasome 20S subunit beta 8 | P28062 |
| PSMB9 | Proteasome 20S subunit beta 9 | P28065 |
| PSMC1 | Proteasome 26S subunit, ATPase 1 | P62191 |
| PSMC2 | Proteasome 26S subunit, ATPase 2 | P35998 |
| PSMC3 | Proteasome 26S subunit, ATPase 3 | P17980 |
| PSMC4 | Proteasome 26S subunit, ATPase 4 | P43686 |
| PSMC5 | Proteasome 26S subunit, ATPase 5 | P62195 |
| PSMC6 | Proteasome 26S subunit, ATPase 6 | P62333 |
| PSMD10 | Proteasome 26S subunit, non-ATPase 10 | O75832 |
| PSMD11 | Proteasome 26S subunit, non-ATPase 11 | O00231 |
| PSMD12 | Proteasome 26S subunit, non-ATPase 12 | O00232 |
| PSMD13 | Proteasome 26S subunit, non-ATPase 13 | Q9UNM6 |
| PSMD14 | Proteasome 26S subunit, non-ATPase 14 | O00487 |
| PSMD2 | Proteasome 26S subunit ubiquitin receptor, non-ATPase 2 | Q13200 |
| PSMD3 | Proteasome 26S subunit, non-ATPase 3 | O43242 |
| PSMD4 | Proteasome 26S subunit ubiquitin receptor, non-ATPase 4 | P55036 |
| PSMD5 | Proteasome 26S subunit, non-ATPase 5 | Q16401 |
| PSMD7 | Proteasome 26S subunit, non-ATPase 7 | P51665 |
| PSMD8 | Proteasome 26S subunit, non-ATPase 8 | P48556 |
| PSMD9 | Proteasome 26S subunit, non-ATPase 9 | O00233 |
| PSME1 | Proteasome activator subunit 1 | Q06323 |
| PSME2 | Proteasome activator subunit 2 | Q9UL46 |
| PSME3 | Proteasome activator subunit 3 | P61289 |
| PSMF1 | Proteasome inhibitor subunit 1 | Q92530 |
| PSMG1 | Proteasome assembly chaperone 1 | O95456 |
| PSMG2 | Proteasome assembly chaperone 2 | Q969U7 |
| PSMG3 | Proteasome assembly chaperone 3 | Q9BT73 |
| PSPC1 | Paraspeckle component 1 | Q8WXF1 |
| PSPH | Phosphoserine phosphatase | P78330 |
| PSTK | Phosphoseryl-tRNA kinase | Q8IV42 |
| PSTPIP1 | Proline-serine-threonine phosphatase interacting protein 1 | O43586 |
| PSTPIP2 | Proline-serine-threonine phosphatase interacting protein 2 | Q9H939 |
| PTBP1 | Polypyrimidine tract binding protein 1 | P26599 |
| PTBP3 | Polypyrimidine tract binding protein 3 | O95758 |
| PTCD1 | Pentatricopeptide repeat domain 1 | O75127 |
| PTCD2 | Pentatricopeptide repeat domain 2 | Q8WV60 |
| PTCD3 | Pentatricopeptide repeat domain 3 | Q96EY7 |
| PTCH1 | Patched 1 | Q13635 |
| PTDSS1 | Phosphatidylserine synthase 1 | P48651 |
| PTEN | Phosphatase and tensin homolog | P60484 |
| PTER | Phosphotriesterase related | Q96BW5 |
| PTGDR | Prostaglandin D2 receptor | Q13258 |
| PTGDS | Prostaglandin D2 synthase | P41222 |
| PTGER2 | Prostaglandin E receptor 2 | P43116 |
| PTGER4 | Prostaglandin E receptor 4 | P35408 |
| PTGES2 | Prostaglandin E synthase 2 | Q9H7Z7 |
| PTGES3 | Prostaglandin E synthase 3 | Q15185 |
| PTGR2 | Prostaglandin reductase 2 | Q8N8N7 |
| PTK2 | Protein tyrosine kinase 2 | Q05397 |
| PTK2B | Protein tyrosine kinase 2 beta | Q14289 |
| PTK7 | Protein tyrosine kinase 7 (inactive) | Q13308 |
| PTMA | Prothymosin alpha | P06454 |
| PTMS | Parathymosin | P20962 |
| PTOV1 | PTOV1 extended AT-hook containing adaptor protein | Q86YD1 |
| PTP4A1 | Protein tyrosine phosphatase 4A1 | Q93096 |
| PTP4A2 | Protein tyrosine phosphatase 4A2 | Q12974 |
| PTP4A3 | Protein tyrosine phosphatase 4A3 | O75365 |
| PTPMT1 | Protein tyrosine phosphatase mitochondrial 1 | Q8WUK0 |
| PTPN1 | Protein tyrosine phosphatase non-receptor type 1 | P18031 |
| PTPN11 | Protein tyrosine phosphatase non-receptor type 11 | Q06124 |
| PTPN12 | Protein tyrosine phosphatase non-receptor type 12 | Q05209 |
| PTPN18 | Protein tyrosine phosphatase non-receptor type 18 | Q99952 |
| PTPN2 | Protein tyrosine phosphatase non-receptor type 2 | P17706 |
| PTPN22 | Protein tyrosine phosphatase non-receptor type 22 | Q9Y2R2 |
| PTPN4 | Protein tyrosine phosphatase non-receptor type 4 | P29074 |
| PTPN6 | Protein tyrosine phosphatase non-receptor type 6 | P29350 |
| PTPN7 | Protein tyrosine phosphatase non-receptor type 7 | P35236 |
| PTPN9 | Protein tyrosine phosphatase non-receptor type 9 | P43378 |
| PTPRA | Protein tyrosine phosphatase receptor type A | P18433 |
| PTPRC | Protein tyrosine phosphatase receptor type C | P08575 |
| PTPRCAP | Protein tyrosine phosphatase receptor type C associated protein | Q14761 |
| PTPRE | Protein tyrosine phosphatase receptor type E | P23469 |
| PTPRJ | Protein tyrosine phosphatase receptor type J | Q12913 |
| PTPRN2 | Protein tyrosine phosphatase receptor type N2 | Q92932 |
| PTPRO | Protein tyrosine phosphatase receptor type O | Q16827 |
| PTRH1 | Peptidyl-tRNA hydrolase 1 homolog | Q86Y79 |
| PTRH2 | Peptidyl-tRNA hydrolase 2 | Q9Y3E5 |
| PTRHD1 | Peptidyl-tRNA hydrolase domain containing 1 | Q6GMV3 |
| PTS | 6-pyruvoyltetrahydropterin synthase | Q03393 |
| PTTG1 | PTTG1 regulator of sister chromatid separation, securin | O95997 |
| PTTG1IP | PTTG1 interacting protein | P53801 |
| PUDP | Pseudouridine 5'-phosphatase | Q08623 |
| PUF60 | Poly(U) binding splicing factor 60 | Q9UHX1 |
| PUM1 | Pumilio RNA binding family member 1 | Q14671 |
| PUM2 | Pumilio RNA binding family member 2 | Q8TB72 |
| PUM3 | Pumilio RNA binding family member 3 | Q15397 |
| PURA | Purine rich element binding protein A | Q00577 |
| PUS1 | Pseudouridine synthase 1 | Q9Y606 |
| PUS3 | Pseudouridine synthase 3 | Q9BZE2 |
| PUS7L | Pseudouridine synthase 7 like | Q9H0K6 |
| PUSL1 | Pseudouridine synthase like 1 | Q8N0Z8 |
| PVRIG | PVR related immunoglobulin domain containing | Q6DKI7 |
| PWP1 | PWP1 homolog, endonuclein | Q13610 |
| PWP2 | PWP2 small subunit processome component | Q15269 |
| PXK | PX domain containing serine/threonine kinase like | Q7Z7A4 |
| PXMP4 | Peroxisomal membrane protein 4 | Q9Y6I8 |
| PXN | Paxillin | P49023 |
| PXYLP1 | 2-phosphoxylose phosphatase 1 | Q8TE99 |
| PYCARD | PYD and CARD domain containing | Q9ULZ3 |
| PYCR1 | Pyrroline-5-carboxylate reductase 1 | P32322 |
| PYCR2 | Pyrroline-5-carboxylate reductase 2 | Q96C36 |
| PYGB | Glycogen phosphorylase B | P11216 |
| PYGO2 | Pygopus family PHD finger 2 | Q9BRQ0 |
| PYHIN1 | Pyrin and HIN domain family member 1 | Q6K0P9 |
| PYM1 | PYM homolog 1, exon junction complex associated factor | Q9BRP8 |
| PYROXD1 | Pyridine nucleotide-disulphide oxidoreductase domain 1 | Q8WU10 |
| PYROXD2 | Pyridine nucleotide-disulphide oxidoreductase domain 2 | Q8N2H3 |
| PYURF | PIGY upstream open reading frame | Q96I23 |
| QDPR | Quinoid dihydropteridine reductase | P09417 |
| QKI | QKI, KH domain containing RNA binding | Q96PU8 |
| QRICH1 | Glutamine rich 1 | Q2TAL8 |
| QRSL1 | Glutaminyl-tRNA amidotransferase subunit QRSL1 | Q9H0R6 |
| QSOX1 | Quiescin sulfhydryl oxidase 1 | O00391 |
| QTRT1 | Queuine tRNA-ribosyltransferase catalytic subunit 1 | Q9BXR0 |
| R3HCC1L | R3H domain and coiled-coil containing 1 like | Q7Z5L2 |
| R3HDM1 | R3H domain containing 1 | Q15032 |
| R3HDM4 | R3H domain containing 4 | Q96D70 |
| RAB10 | RAB10, member RAS oncogene family | P61026 |
| RAB11A | RAB11A, member RAS oncogene family | P62491 |
| RAB11B | RAB11B, member RAS oncogene family | Q15907 |
| RAB11FIP1 | RAB11 family interacting protein 1 | Q6WKZ4 |
| RAB11FIP4 | RAB11 family interacting protein 4 | Q86YS3 |
| RAB12 | RAB12, member RAS oncogene family |  |
| RAB14 | RAB14, member RAS oncogene family | P61106 |
| RAB18 | RAB18, member RAS oncogene family | Q9NP72 |
| RAB1A | RAB1A, member RAS oncogene family | P62820 |
| RAB1B | RAB1B, member RAS oncogene family | Q9H0U4 |
| RAB20 | RAB20, member RAS oncogene family | Q9NX57 |
| RAB22A | RAB22A, member RAS oncogene family | Q9UL26 |
| RAB24 | RAB24, member RAS oncogene family | Q969Q5 |
| RAB25 | RAB25, member RAS oncogene family | P57735 |
| RAB27A | RAB27A, member RAS oncogene family | P51159 |
| RAB27B | RAB27B, member RAS oncogene family | O00194 |
| RAB28 | RAB28, member RAS oncogene family | P51157 |
| RAB29 | RAB29, member RAS oncogene family | O14966 |
| RAB2A | RAB2A, member RAS oncogene family | P61019 |
| RAB2B | RAB2B, member RAS oncogene family | Q8WUD1 |
| RAB30 | RAB30, member RAS oncogene family | Q15771 |
| RAB33A | RAB33A, member RAS oncogene family | Q14088 |
| RAB33B | RAB33B, member RAS oncogene family | Q9H082 |
| RAB34 | RAB34, member RAS oncogene family | P0DI83, Q9BZG1 |
| RAB35 | RAB35, member RAS oncogene family | Q15286 |
| RAB37 | RAB37, member RAS oncogene family | Q96AX2 |
| RAB38 | RAB38, member RAS oncogene family | P57729 |
| RAB39B | RAB39B, member RAS oncogene family | Q96DA2 |
| RAB3A | RAB3A, member RAS oncogene family | P20336 |
| RAB3D | RAB3D, member RAS oncogene family | O95716 |
| RAB3IP | RAB3A interacting protein | Q96QF0 |
| RAB40B | RAB40B, member RAS oncogene family | Q12829 |
| RAB40C | RAB40C, member RAS oncogene family | Q96S21 |
| RAB43 | RAB43, member RAS oncogene family | Q86YS6 |
| RAB4A | RAB4A, member RAS oncogene family | P20338 |
| RAB4B | RAB4B, member RAS oncogene family | P61018 |
| RAB5A | RAB5A, member RAS oncogene family | P20339 |
| RAB5B | RAB5B, member RAS oncogene family | P61020 |
| RAB5C | RAB5C, member RAS oncogene family | P51148 |
| RAB6B | RAB6B, member RAS oncogene family | Q9NRW1 |
| RAB7A | RAB7A, member RAS oncogene family | P51149 |
| RAB8A | RAB8A, member RAS oncogene family | P61006 |
| RAB8B | RAB8B, member RAS oncogene family | Q92930 |
| RAB9A | RAB9A, member RAS oncogene family | P51151 |
| RABAC1 | Rab acceptor 1 | Q9UI14 |
| RABEP1 | Rabaptin, RAB GTPase binding effector protein 1 | Q15276 |
| RABEP2 | Rabaptin, RAB GTPase binding effector protein 2 | Q9H5N1 |
| RABEPK | Rab9 effector protein with kelch motifs | Q7Z6M1 |
| RABGAP1 | RAB GTPase activating protein 1 | Q9Y3P9 |
| RABGAP1L | RAB GTPase activating protein 1 like | B7ZAP0, Q5R372 |
| RABGEF1 | RAB guanine nucleotide exchange factor 1 | Q9UJ41 |
| RABGGTA | Rab geranylgeranyltransferase subunit alpha | Q92696 |
| RABGGTB | Rab geranylgeranyltransferase subunit beta | P53611 |
| RABIF | RAB interacting factor | P47224 |
| RABL2A | RAB, member of RAS oncogene family like 2A | Q9UBK7 |
| RABL2B | RAB, member of RAS oncogene family like 2B | Q9UNT1 |
| RABL3 | RAB, member of RAS oncogene family like 3 | Q5HYI8 |
| RABL6 | RAB, member RAS oncogene family like 6 | Q3YEC7 |
| RAC1 | Rac family small GTPase 1 | P63000 |
| RAC2 | Rac family small GTPase 2 | P15153 |
| RACGAP1 | Rac GTPase activating protein 1 | Q9H0H5 |
| RACK1 | Receptor for activated C kinase 1 | P63244 |
| RAD1 | RAD1 checkpoint DNA exonuclease | O60671 |
| RAD17 | RAD17 checkpoint clamp loader component | O75943 |
| RAD18 | RAD18 E3 ubiquitin protein ligase | Q9NS91 |
| RAD21 | RAD21 cohesin complex component | O60216 |
| RAD23A | RAD23 homolog A, nucleotide excision repair protein | P54725 |
| RAD23B | RAD23 homolog B, nucleotide excision repair protein | P54727 |
| RAD51 | RAD51 recombinase | Q06609 |
| RAD51AP1 | RAD51 associated protein 1 | Q96B01 |
| RAD51B | RAD51 paralog B | O15315 |
| RAD51C | RAD51 paralog C | O43502 |
| RAD51D | RAD51 paralog D | O75771 |
| RAD52 | RAD52 homolog, DNA repair protein | P43351 |
| RAD54B | RAD54 homolog B | Q9Y620 |
| RAD54L2 | RAD54 like 2 | Q9Y4B4 |
| RAE1 | Ribonucleic acid export 1 | P78406 |
| RAF1 | Raf-1 proto-oncogene, serine/threonine kinase | P04049 |
| RAI2 | Retinoic acid induced 2 | Q9Y5P3 |
| RALA | RAS like proto-oncogene A | P11233 |
| RALB | RAS like proto-oncogene B | P11234 |
| RALBP1 | RalA binding protein 1 | Q15311 |
| RALGAPB | Ral GTPase activating protein non-catalytic subunit beta | Q86X10 |
| RALGDS | Ral guanine nucleotide dissociation stimulator | Q12967 |
| RALY | RALY heterogeneous nuclear ribonucleoprotein | Q9UKM9 |
| RAN | RAN, member RAS oncogene family | P62826 |
| RANBP1 | RAN binding protein 1 | P43487 |
| RANBP3 | RAN binding protein 3 | Q9H6Z4 |
| RANBP6 | RAN binding protein 6 | O60518 |
| RANGAP1 | Ran GTPase activating protein 1 | P46060 |
| RANGRF | RAN guanine nucleotide release factor | Q9HD47 |
| RAP1A | RAP1A, member of RAS oncogene family | P62834 |
| RAP1B | RAP1B, member of RAS oncogene family | P61224 |
| RAP1GDS1 | Rap1 GTPase-GDP dissociation stimulator 1 | P52306 |
| RAP2A | RAP2A, member of RAS oncogene family | P10114 |
| RAP2B | RAP2B, member of RAS oncogene family | P61225 |
| RAP2C | RAP2C, member of RAS oncogene family | Q9Y3L5 |
| RAPGEF1 | Rap guanine nucleotide exchange factor 1 | Q13905 |
| RAPGEF6 | Rap guanine nucleotide exchange factor 6 | Q8TEU7 |
| RARA | Retinoic acid receptor alpha | P10276 |
| RARG | Retinoic acid receptor gamma | P13631 |
| RARS2 | Arginyl-tRNA synthetase 2, mitochondrial | Q5T160 |
| RASA1 | RAS p21 protein activator 1 | P20936 |
| RASA2 | RAS p21 protein activator 2 | Q15283 |
| RASA3 | RAS p21 protein activator 3 | Q14644 |
| RASA4 | RAS p21 protein activator 4 | O43374 |
| RASAL3 | RAS protein activator like 3 | Q86YV0 |
| RASEF | RAS and EF-hand domain containing | Q8IZ41 |
| RASGEF1A | RasGEF domain family member 1A | Q8N9B8 |
| RASGEF1B | RasGEF domain family member 1B | Q0VAM2 |
| RASGRP1 | RAS guanyl releasing protein 1 | O95267 |
| RASGRP2 | RAS guanyl releasing protein 2 | Q7LDG7 |
| RASSF1 | Ras association domain family member 1 | Q9NS23 |
| RASSF2 | Ras association domain family member 2 | P50749 |
| RASSF3 | Ras association domain family member 3 | Q86WH2 |
| RASSF4 | Ras association domain family member 4 | Q9H2L5 |
| RASSF5 | Ras association domain family member 5 | Q8WWW0 |
| RASSF7 | Ras association domain family member 7 | Q02833 |
| RB1 | RB transcriptional corepressor 1 | P06400 |
| RB1CC1 | RB1 inducible coiled-coil 1 | Q8TDY2 |
| RBBP4 | RB binding protein 4, chromatin remodeling factor | Q09028 |
| RBBP5 | RB binding protein 5, histone lysine methyltransferase complex subunit | Q15291 |
| RBBP6 | RB binding protein 6, ubiquitin ligase | Q7Z6E9 |
| RBBP7 | RB binding protein 7, chromatin remodeling factor | Q16576 |
| RBBP8 | RB binding protein 8, endonuclease | Q99708 |
| RBBP9 | RB binding protein 9, serine hydrolase | O75884 |
| RBCK1 | RANBP2-type and C3HC4-type zinc finger containing 1 | Q9BYM8 |
| RBFA | Ribosome binding factor A | Q8N0V3 |
| RBKS | Ribokinase | Q9H477 |
| RBL1 | RB transcriptional corepressor like 1 | P28749 |
| RBL2 | RB transcriptional corepressor like 2 | Q08999 |
| RBM10 | RNA binding motif protein 10 | P98175 |
| RBM12 | RNA binding motif protein 12 | Q9NTZ6 |
| RBM14 | RNA binding motif protein 14 | Q96PK6 |
| RBM15 | RNA binding motif protein 15 | Q96T37 |
| RBM15B | RNA binding motif protein 15B | Q8NDT2 |
| RBM17 | RNA binding motif protein 17 | Q96I25 |
| RBM18 | RNA binding motif protein 18 | Q96H35 |
| RBM19 | RNA binding motif protein 19 | Q9Y4C8 |
| RBM22 | RNA binding motif protein 22 | Q9NW64 |
| RBM23 | RNA binding motif protein 23 | Q86U06 |
| RBM25 | RNA binding motif protein 25 | P49756 |
| RBM28 | RNA binding motif protein 28 | Q9NW13 |
| RBM3 | RNA binding motif protein 3 | P98179 |
| RBM33 | RNA binding motif protein 33 | Q96EV2 |
| RBM34 | RNA binding motif protein 34 | P42696 |
| RBM38 | RNA binding motif protein 38 | Q9H0Z9 |
| RBM39 | RNA binding motif protein 39 | Q14498 |
| RBM4 | RNA binding motif protein 4 | Q9BWF3 |
| RBM41 | RNA binding motif protein 41 | Q96IZ5 |
| RBM42 | RNA binding motif protein 42 | Q9BTD8 |
| RBM43 | RNA binding motif protein 43 | Q6ZSC3 |
| RBM45 | RNA binding motif protein 45 | Q8IUH3 |
| RBM48 | RNA binding motif protein 48 | Q5RL73 |
| RBM4B | RNA binding motif protein 4B | Q9BQ04 |
| RBM5 | RNA binding motif protein 5 | P52756 |
| RBM6 | RNA binding motif protein 6 | P78332 |
| RBM7 | RNA binding motif protein 7 | Q9Y580 |
| RBM8A | RNA binding motif protein 8A | Q9Y5S9 |
| RBMS1 | RNA binding motif single stranded interacting protein 1 | P29558 |
| RBMS2 | RNA binding motif single stranded interacting protein 2 | Q15434 |
| RBMX | RNA binding motif protein X-linked | P38159 |
| RBMX2 | RNA binding motif protein X-linked 2 | Q9Y388 |
| RBPJ | Recombination signal binding protein for immunoglobulin kappa J region | Q06330 |
| RBSN | Rabenosyn, RAB effector | Q9H1K0 |
| RBX1 | Ring-box 1 | P62877 |
| RC3H2 | Ring finger and CCCH-type domains 2 | Q9HBD1 |
| RCAN2 | Regulator of calcineurin 2 | Q14206 |
| RCAN3 | RCAN family member 3 | Q9UKA8 |
| RCBTB1 | RCC1 and BTB domain containing protein 1 | Q8NDN9 |
| RCBTB2 | RCC1 and BTB domain containing protein 2 | O95199 |
| RCC1 | Regulator of chromosome condensation 1 | P18754 |
| RCE1 | Ras converting CAAX endopeptidase 1 | Q9Y256 |
| RCHY1 | Ring finger and CHY zinc finger domain containing 1 | Q96PM5 |
| RCL1 | RNA terminal phosphate cyclase like 1 | Q9Y2P8 |
| RCN1 | Reticulocalbin 1 | Q15293 |
| RCN2 | Reticulocalbin 2 | Q14257 |
| RCN3 | Reticulocalbin 3 | Q96D15 |
| RCOR3 | REST corepressor 3 | Q9P2K3 |
| RCSD1 | RCSD domain containing 1 | Q6JBY9 |
| RDH11 | Retinol dehydrogenase 11 | Q8TC12 |
| RDH13 | Retinol dehydrogenase 13 | Q8NBN7 |
| RDH14 | Retinol dehydrogenase 14 | Q9HBH5 |
| RDM1 | RAD52 motif containing 1 | Q8NG50 |
| RDX | Radixin | P35241 |
| REC8 | REC8 meiotic recombination protein | O95072 |
| RECK | Reversion inducing cysteine rich protein with kazal motifs | O95980 |
| RECQL | RecQ like helicase | P46063 |
| RECQL5 | RecQ like helicase 5 | O94762 |
| REEP3 | Receptor accessory protein 3 | Q6NUK4 |
| REEP4 | Receptor accessory protein 4 | Q9H6H4 |
| REEP5 | Receptor accessory protein 5 | Q00765 |
| REEP6 | Receptor accessory protein 6 | Q96HR9 |
| REL | REL proto-oncogene, NF-kB subunit | Q04864 |
| RELA | RELA proto-oncogene, NF-kB subunit | Q04206 |
| RELB | RELB proto-oncogene, NF-kB subunit | Q01201 |
| RELL1 | RELT like 1 | Q8IUW5 |
| RELL2 | RELT like 2 | Q8NC24 |
| RELT | RELT TNF receptor | Q969Z4 |
| REPIN1 | Replication initiator 1 | Q9BWE0 |
| REPS1 | RALBP1 associated Eps domain containing 1 | Q96D71 |
| RER1 | Retention in endoplasmic reticulum sorting receptor 1 | O15258 |
| RERE | Arginine-glutamic acid dipeptide repeats | Q9P2R6 |
| REST | RE1 silencing transcription factor | Q13127 |
| RETN | Resistin | Q9HD89 |
| RETSAT | Retinol saturase | Q6NUM9 |
| REV3L | REV3 like, DNA directed polymerase zeta catalytic subunit | O60673 |
| REXO2 | RNA exonuclease 2 | Q9Y3B8 |
| REXO4 | REX4 homolog, 3'-5' exonuclease | Q9GZR2 |
| RFC1 | Replication factor C subunit 1 | P35251 |
| RFC2 | Replication factor C subunit 2 | P35250 |
| RFC3 | Replication factor C subunit 3 | P40938 |
| RFC4 | Replication factor C subunit 4 | P35249 |
| RFC5 | Replication factor C subunit 5 | P40937 |
| RFESD | Rieske Fe-S domain containing | Q8TAC1 |
| RFFL | Ring finger and FYVE like domain containing E3 ubiquitin protein ligase | Q8WZ73 |
| RFK | Riboflavin kinase | Q969G6 |
| RFT1 | RFT1 homolog | Q96AA3 |
| RFTN1 | Raftlin, lipid raft linker 1 | Q14699 |
| RFWD3 | Ring finger and WD repeat domain 3 | Q6PCD5 |
| RFX3 | Regulatory factor X3 | P48380 |
| RFX5 | Regulatory factor X5 | P48382 |
| RFXANK | Regulatory factor X associated ankyrin containing protein | O14593 |
| RGCC | Regulator of cell cycle | Q9H4X1 |
| RGL4 | Ral guanine nucleotide dissociation stimulator like 4 | Q8IZJ4 |
| RGMB | Repulsive guidance molecule BMP co-receptor b | Q6NW40 |
| RGPD5 | RANBP2 like and GRIP domain containing 5 | Q99666 |
| RGS1 | Regulator of G protein signaling 1 | Q08116 |
| RGS10 | Regulator of G protein signaling 10 | O43665 |
| RGS14 | Regulator of G protein signaling 14 | O43566 |
| RGS18 | Regulator of G protein signaling 18 | Q9NS28 |
| RGS19 | Regulator of G protein signaling 19 | P49795 |
| RGS2 | Regulator of G protein signaling 2 | P41220 |
| RGS3 | Regulator of G protein signaling 3 | P49796 |
| RHBDD1 | Rhomboid domain containing 1 | Q8TEB9 |
| RHBDD2 | Rhomboid domain containing 2 | Q6NTF9 |
| RHBDD3 | Rhomboid domain containing 3 | Q9Y3P4 |
| RHBDF2 | Rhomboid 5 homolog 2 | Q6PJF5 |
| RHEB | Ras homolog, mTORC1 binding | Q15382 |
| RHEBL1 | RHEB like 1 | Q8TAI7 |
| RHNO1 | RAD9-HUS1-RAD1 interacting nuclear orphan 1 | Q9BSD3 |
| RHOA | Ras homolog family member A | P61586 |
| RHOBTB3 | Rho related BTB domain containing 3 | O94955 |
| RHOC | Ras homolog family member C | P08134 |
| RHOG | Ras homolog family member G | P84095 |
| RHOH | Ras homolog family member H | Q15669 |
| RHOT1 | Ras homolog family member T1 | Q8IXI2 |
| RHOT2 | Ras homolog family member T2 | Q8IXI1 |
| RIC8A | RIC8 guanine nucleotide exchange factor A | Q9NPQ8 |
| RICTOR | RPTOR independent companion of MTOR complex 2 | Q6R327 |
| RILPL2 | Rab interacting lysosomal protein like 2 | Q969X0 |
| RIMKLB | Ribosomal modification protein rimK like family member B | Q9ULI2 |
| RING1 | Ring finger protein 1 | Q06587 |
| RINL | Ras and Rab interactor like | Q6ZS11 |
| RINT1 | RAD50 interactor 1 | Q6NUQ1 |
| RIOK1 | RIO kinase 1 | Q9BRS2 |
| RIOK2 | RIO kinase 2 | Q9BVS4 |
| RIOK3 | RIO kinase 3 | O14730 |
| RIPK1 | Receptor interacting serine/threonine kinase 1 | Q13546 |
| RIPK2 | Receptor interacting serine/threonine kinase 2 | O43353 |
| RIPK3 | Receptor interacting serine/threonine kinase 3 | Q9Y572 |
| RIT1 | Ras like without CAAX 1 | Q92963 |
| RITA1 | RBPJ interacting and tubulin associated 1 | Q96K30 |
| RLF | RLF zinc finger | Q13129 |
| RLIM | Ring finger protein, LIM domain interacting | Q9NVW2 |
| RLN2 | Relaxin 2 | P04090 |
| RMDN3 | Regulator of microtubule dynamics 3 | Q96TC7 |
| RMI1 | RecQ mediated genome instability 1 | Q9H9A7 |
| RMND1 | Required for meiotic nuclear division 1 homolog | Q9NWS8 |
| RMND5A | Required for meiotic nuclear division 5 homolog A | Q9H871 |
| RMND5B | Required for meiotic nuclear division 5 homolog B | Q96G75 |
| RNASE6 | Ribonuclease A family member k6 | Q93091 |
| RNASEH1 | Ribonuclease H1 | O60930 |
| RNASEH2A | Ribonuclease H2 subunit A | O75792 |
| RNASEH2B | Ribonuclease H2 subunit B | Q5TBB1 |
| RNASEH2C | Ribonuclease H2 subunit C | Q8TDP1 |
| RNASEK | Ribonuclease K | Q6P5S7 |
| RNASEL | Ribonuclease L | Q05823 |
| RNASET2 | Ribonuclease T2 | O00584 |
| RNF10 | Ring finger protein 10 | Q8N5U6 |
| RNF103 | Ring finger protein 103 | O00237 |
| RNF11 | Ring finger protein 11 | Q9Y3C5 |
| RNF111 | Ring finger protein 111 | Q6ZNA4 |
| RNF113A | Ring finger protein 113A | O15541 |
| RNF114 | Ring finger protein 114 | Q9Y508 |
| RNF115 | Ring finger protein 115 | Q9Y4L5 |
| RNF121 | Ring finger protein 121 | Q9H920 |
| RNF122 | Ring finger protein 122 | Q9H9V4 |
| RNF123 | Ring finger protein 123 | Q5XPI4 |
| RNF125 | Ring finger protein 125 | Q96EQ8 |
| RNF126 | Ring finger protein 126 | Q9BV68 |
| RNF13 | Ring finger protein 13 | O43567 |
| RNF135 | Ring finger protein 135 | Q8IUD6 |
| RNF138 | Ring finger protein 138 | Q8WVD3 |
| RNF139 | Ring finger protein 139 | Q8WU17 |
| RNF14 | Ring finger protein 14 | Q9UBS8 |
| RNF141 | Ring finger protein 141 | Q8WVD5 |
| RNF144A | Ring finger protein 144A | P50876 |
| RNF144B | Ring finger protein 144B | Q7Z419 |
| RNF149 | Ring finger protein 149 | Q8NC42 |
| RNF157 | Ring finger protein 157 | Q96PX1 |
| RNF166 | Ring finger protein 166 | Q96A37 |
| RNF167 | Ring finger protein 167 | Q9H6Y7 |
| RNF168 | Ring finger protein 168 | Q8IYW5 |
| RNF170 | Ring finger protein 170 | Q96K19 |
| RNF180 | Ring finger protein 180 | Q86T96 |
| RNF181 | Ring finger protein 181 | Q9P0P0 |
| RNF185 | Ring finger protein 185 | Q96GF1 |
| RNF19A | Ring finger protein 19A, RBR E3 ubiquitin protein ligase | Q9NV58 |
| RNF2 | Ring finger protein 2 | Q99496 |
| RNF20 | Ring finger protein 20 | Q5VTR2 |
| RNF213 | Ring finger protein 213 | Q63HN8 |
| RNF215 | Ring finger protein 215 | Q9Y6U7 |
| RNF216 | Ring finger protein 216 | Q9NWF9 |
| RNF220 | Ring finger protein 220 | Q5VTB9 |
| RNF24 | Ring finger protein 24 | Q9Y225 |
| RNF25 | Ring finger protein 25 | Q96BH1 |
| RNF26 | Ring finger protein 26 | Q9BY78 |
| RNF34 | Ring finger protein 34 | Q969K3 |
| RNF38 | Ring finger protein 38 | Q9H0F5 |
| RNF4 | Ring finger protein 4 | P78317 |
| RNF40 | Ring finger protein 40 | O75150 |
| RNF41 | Ring finger protein 41 | Q9H4P4 |
| RNF5 | Ring finger protein 5 | Q99942 |
| RNF6 | Ring finger protein 6 | Q9Y252 |
| RNF7 | Ring finger protein 7 | Q9UBF6 |
| RNF8 | Ring finger protein 8 | O76064 |
| RNFT1 | Ring finger protein, transmembrane 1 | Q5M7Z0 |
| RNGTT | RNA guanylyltransferase and 5'-phosphatase | O60942 |
| RNH1 | Ribonuclease/angiogenin inhibitor 1 | P13489 |
| RNLS | Renalase, FAD dependent amine oxidase | Q5VYX0 |
| RNMT | RNA guanine-7 methyltransferase | O43148 |
| RNPC3 | RNA binding region (RNP1, RRM) containing 3 | Q96LT9 |
| RNPEP | Arginyl aminopeptidase | Q9H4A4 |
| RNPEPL1 | Arginyl aminopeptidase like 1 | Q9HAU8 |
| RNPS1 | RNA binding protein with serine rich domain 1 | Q15287 |
| ROMO1 | Reactive oxygen species modulator 1 | P60602 |
| ROPN1L | Rhophilin associated tail protein 1 like | Q96C74 |
| RORA | RAR related orphan receptor A | P35398 |
| RP2 | RP2 activator of ARL3 GTPase | O75695 |
| RP9 | RP9 pre-mRNA splicing factor | Q8TA86 |
| RPA1 | Replication protein A1 | P27694 |
| RPA2 | Replication protein A2 | P15927 |
| RPA3 | Replication protein A3 | P35244 |
| RPAIN | RPA interacting protein | Q86UA6 |
| RPAP1 | RNA polymerase II associated protein 1 | Q9BWH6 |
| RPAP2 | RNA polymerase II associated protein 2 | Q8IXW5 |
| RPAP3 | RNA polymerase II associated protein 3 | Q9H6T3 |
| RPE | Ribulose-5-phosphate-3-epimerase | Q96AT9 |
| RPF1 | Ribosome production factor 1 homolog | Q9H9Y2 |
| RPF2 | Ribosome production factor 2 homolog | Q9H7B2 |
| RPL10 | Ribosomal protein L10 | P27635 |
| RPL10A | Ribosomal protein L10a | P62906 |
| RPL11 | Ribosomal protein L11 | P62913 |
| RPL12 | Ribosomal protein L12 | P30050 |
| RPL13 | Ribosomal protein L13 | P26373 |
| RPL13A | Ribosomal protein L13a | P40429 |
| RPL14 | Ribosomal protein L14 | P50914 |
| RPL15 | Ribosomal protein L15 | P61313 |
| RPL17 | Ribosomal protein L17 | P18621 |
| RPL18 | Ribosomal protein L18 | Q07020 |
| RPL18A | Ribosomal protein L18a | Q02543 |
| RPL19 | Ribosomal protein L19 | P84098 |
| RPL21 | Ribosomal protein L21 | P46778 |
| RPL22 | Ribosomal protein L22 | P35268 |
| RPL22L1 | Ribosomal protein L22 like 1 | Q6P5R6 |
| RPL23 | Ribosomal protein L23 | P62829 |
| RPL23A | Ribosomal protein L23a | P62750 |
| RPL24 | Ribosomal protein L24 | P83731 |
| RPL26 | Ribosomal protein L26 | P61254 |
| RPL26L1 | Ribosomal protein L26 like 1 | Q9UNX3 |
| RPL27 | Ribosomal protein L27 | P61353 |
| RPL27A | Ribosomal protein L27a | P46776 |
| RPL28 | Ribosomal protein L28 | P46779 |
| RPL29 | Ribosomal protein L29 | P47914 |
| RPL3 | Ribosomal protein L3 | P39023 |
| RPL30 | Ribosomal protein L30 | P62888 |
| RPL31 | Ribosomal protein L31 | P62899 |
| RPL32 | Ribosomal protein L32 | P62910 |
| RPL34 | Ribosomal protein L34 | P49207 |
| RPL35 | Ribosomal protein L35 | P42766 |
| RPL35A | Ribosomal protein L35a | P18077 |
| RPL36 | Ribosomal protein L36 | Q9Y3U8 |
| RPL36A | Ribosomal protein L36a | P83881 |
| RPL36AL | Ribosomal protein L36a like | Q969Q0 |
| RPL37 | Ribosomal protein L37 | P61927 |
| RPL37A | Ribosomal protein L37a | P61513 |
| RPL38 | Ribosomal protein L38 | P63173 |
| RPL39 | Ribosomal protein L39 | P62891 |
| RPL39L | Ribosomal protein L39 like | Q96EH5 |
| RPL4 | Ribosomal protein L4 | P36578 |
| RPL6 | Ribosomal protein L6 | Q02878 |
| RPL7 | Ribosomal protein L7 | P18124 |
| RPL7A | Ribosomal protein L7a | P62424 |
| RPL7L1 | Ribosomal protein L7 like 1 | Q6DKI1 |
| RPL8 | Ribosomal protein L8 | P62917 |
| RPL9 | Ribosomal protein L9 | P32969 |
| RPLP0 | Ribosomal protein lateral stalk subunit P0 | P05388 |
| RPLP1 | Ribosomal protein lateral stalk subunit P1 | P05386 |
| RPLP2 | Ribosomal protein lateral stalk subunit P2 | P05387 |
| RPN2 | Ribophorin II | P04844 |
| RPP14 | Ribonuclease P/MRP subunit p14 | O95059 |
| RPP21 | Ribonuclease P/MRP subunit p21 | Q9H633 |
| RPP25 | Ribonuclease P and MRP subunit p25 | Q9BUL9 |
| RPP25L | Ribonuclease P/MRP subunit p25 like | Q8N5L8 |
| RPP30 | Ribonuclease P/MRP subunit p30 | P78346 |
| RPP38 | Ribonuclease P/MRP subunit p38 | P78345 |
| RPP40 | Ribonuclease P/MRP subunit p40 | O75818 |
| RPRD1A | Regulation of nuclear pre-mRNA domain containing 1A | Q96P16 |
| RPRD1B | Regulation of nuclear pre-mRNA domain containing 1B | Q9NQG5 |
| RPRD2 | Regulation of nuclear pre-mRNA domain containing 2 | Q5VT52 |
| RPS10 | Ribosomal protein S10 | P46783 |
| RPS11 | Ribosomal protein S11 | P62280 |
| RPS12 | Ribosomal protein S12 | P25398 |
| RPS13 | Ribosomal protein S13 | P62277 |
| RPS14 | Ribosomal protein S14 | P62263 |
| RPS15 | Ribosomal protein S15 | P62841 |
| RPS15A | Ribosomal protein S15a | P62244 |
| RPS16 | Ribosomal protein S16 | P62249 |
| RPS17 | Ribosomal protein S17 | P08708 |
| RPS18 | Ribosomal protein S18 | P62269 |
| RPS19 | Ribosomal protein S19 | P39019 |
| RPS19BP1 | Ribosomal protein S19 binding protein 1 | Q86WX3 |
| RPS2 | Ribosomal protein S2 | P15880 |
| RPS20 | Ribosomal protein S20 | P60866 |
| RPS21 | Ribosomal protein S21 | P63220 |
| RPS24 | Ribosomal protein S24 | P62847 |
| RPS25 | Ribosomal protein S25 | P62851 |
| RPS26 | Ribosomal protein S26 | P62854 |
| RPS27 | Ribosomal protein S27 | P42677 |
| RPS27A | Ribosomal protein S27a | P62979 |
| RPS27L | Ribosomal protein S27 like | Q71UM5 |
| RPS28 | Ribosomal protein S28 | P62857 |
| RPS29 | Ribosomal protein S29 | P62273 |
| RPS3 | Ribosomal protein S3 | P23396 |
| RPS3A | Ribosomal protein S3A | P61247 |
| RPS4X | Ribosomal protein S4 X-linked | P62701 |
| RPS4Y1 | Ribosomal protein S4 Y-linked 1 | P22090 |
| RPS5 | Ribosomal protein S5 | P46782 |
| RPS6 | Ribosomal protein S6 | P62753 |
| RPS6KA1 | Ribosomal protein S6 kinase A1 | Q15418 |
| RPS6KA3 | Ribosomal protein S6 kinase A3 | P51812 |
| RPS6KA5 | Ribosomal protein S6 kinase A5 | O75582 |
| RPS6KB1 | Ribosomal protein S6 kinase B1 | P23443 |
| RPS6KB2 | Ribosomal protein S6 kinase B2 | Q9UBS0 |
| RPS7 | Ribosomal protein S7 | P62081 |
| RPS8 | Ribosomal protein S8 | P62241 |
| RPS9 | Ribosomal protein S9 | P46781 |
| RPSA | Ribosomal protein SA | P08865 |
| RPUSD1 | RNA pseudouridine synthase domain containing 1 | Q9UJJ7 |
| RPUSD2 | RNA pseudouridine synthase domain containing 2 | Q8IZ73 |
| RPUSD3 | RNA pseudouridine synthase D3 | Q6P087 |
| RPUSD4 | RNA pseudouridine synthase D4 | Q96CM3 |
| RRAGA | Ras related GTP binding A | Q7L523 |
| RRAGB | Ras related GTP binding B | Q5VZM2 |
| RRAGC | Ras related GTP binding C | Q9HB90 |
| RRAS2 | RAS related 2 | P62070 |
| RREB1 | Ras responsive element binding protein 1 | Q92766 |
| RRM1 | Ribonucleotide reductase catalytic subunit M1 | P23921 |
| RRM2B | Ribonucleotide reductase regulatory TP53 inducible subunit M2B | Q7LG56 |
| RRP1 | Ribosomal RNA processing 1 | P56182 |
| RRP12 | Ribosomal RNA processing 12 homolog | Q5JTH9 |
| RRP15 | Ribosomal RNA processing 15 homolog | Q9Y3B9 |
| RRP36 | Ribosomal RNA processing 36 | Q96EU6 |
| RRP7A | Ribosomal RNA processing 7 homolog A | Q9Y3A4 |
| RRP8 | Ribosomal RNA processing 8 | O43159 |
| RRP9 | Ribosomal RNA processing 9, U3 small nucleolar RNA binding protein | O43818 |
| RRS1 | Ribosome biogenesis regulator 1 homolog | Q15050 |
| RSAD1 | Radical S-adenosyl methionine domain containing 1 | Q9HA92 |
| RSAD2 | Radical S-adenosyl methionine domain containing 2 | Q8WXG1 |
| RSBN1 | Round spermatid basic protein 1 | Q5VWQ0 |
| RSBN1L | Round spermatid basic protein 1 like | Q6PCB5 |
| RSF1 | Remodeling and spacing factor 1 | Q96T23 |
| RSL1D1 | Ribosomal L1 domain containing 1 | O76021 |
| RSL24D1 | Ribosomal L24 domain containing 1 | Q9UHA3 |
| RSPH3 | Radial spoke head 3 |  |
| RSPRY1 | Ring finger and SPRY domain containing 1 | Q96DX4 |
| RSRC1 | Arginine and serine rich coiled-coil 1 | Q96IZ7 |
| RSRC2 | Arginine and serine rich coiled-coil 2 | Q7L4I2 |
| RSRP1 | Arginine and serine rich protein 1 | Q9BUV0 |
| RSU1 | Ras suppressor protein 1 | Q15404 |
| RTCA | RNA 3'-terminal phosphate cyclase | O00442 |
| RTCB | RNA 2',3'-cyclic phosphate and 5'-OH ligase | Q9Y3I0 |
| RTF1 | RTF1 homolog, Paf1/RNA polymerase II complex component | Q92541 |
| RTN3 | Reticulon 3 | O95197 |
| RTN4 | Reticulon 4 | Q9NQC3 |
| RTN4IP1 | Reticulon 4 interacting protein 1 | Q8WWV3 |
| RTN4R | Reticulon 4 receptor | Q9BZR6 |
| RTP4 | Receptor transporter protein 4 | Q96DX8 |
| RUBCN | Rubicon autophagy regulator | Q92622 |
| RUFY1 | RUN and FYVE domain containing 1 | Q96T51 |
| RUFY2 | RUN and FYVE domain containing 2 | Q8WXA3 |
| RUFY3 | RUN and FYVE domain containing 3 | Q7L099 |
| RUNDC1 | RUN domain containing 1 | Q96C34 |
| RUNX1 | RUNX family transcription factor 1 | Q01196 |
| RUNX2 | RUNX family transcription factor 2 | Q13950 |
| RUNX3 | RUNX family transcription factor 3 | Q13761 |
| RUSC1 | RUN and SH3 domain containing 1 | Q9BVN2 |
| RUVBL1 | RuvB like AAA ATPase 1 | Q9Y265 |
| RUVBL2 | RuvB like AAA ATPase 2 | Q9Y230 |
| RWDD1 | RWD domain containing 1 | Q9H446 |
| RWDD2A | RWD domain containing 2A | Q9UIY3 |
| RWDD3 | RWD domain containing 3 | Q9Y3V2 |
| RWDD4 | RWD domain containing 4 | Q6NW29 |
| RXRA | Retinoid X receptor alpha | P19793 |
| RXRB | Retinoid X receptor beta | P28702 |
| RYK | Receptor like tyrosine kinase | P34925 |
| S100A10 | S100 calcium binding protein A10 | P60903 |
| S100A11 | S100 calcium binding protein A11 | P31949 |
| S100A12 | S100 calcium binding protein A12 | P80511 |
| S100A2 | S100 calcium binding protein A2 | P29034 |
| S100A4 | S100 calcium binding protein A4 | P26447 |
| S100A6 | S100 calcium binding protein A6 | P06703 |
| S100A8 | S100 calcium binding protein A8 | P05109 |
| S100A9 | S100 calcium binding protein A9 | P06702 |
| S100B | S100 calcium binding protein B | P04271 |
| S100P | S100 calcium binding protein P | P25815 |
| S1PR1 | Sphingosine-1-phosphate receptor 1 | P21453 |
| S1PR2 | Sphingosine-1-phosphate receptor 2 | O95136 |
| S1PR4 | Sphingosine-1-phosphate receptor 4 | O95977 |
| S1PR5 | Sphingosine-1-phosphate receptor 5 | Q9H228 |
| SAAL1 | Serum amyloid A like 1 | Q96ER3 |
| SACM1L | SAC1 like phosphatidylinositide phosphatase | Q9NTJ5 |
| SAE1 | SUMO1 activating enzyme subunit 1 | Q9UBE0 |
| SAFB | Scaffold attachment factor B | Q15424 |
| SAFB2 | Scaffold attachment factor B2 | Q14151 |
| SALL2 | Spalt like transcription factor 2 | Q9Y467 |
| SAMD1 | Sterile alpha motif domain containing 1 |  |
| SAMD3 | Sterile alpha motif domain containing 3 | Q8N6K7 |
| SAMD8 | Sterile alpha motif domain containing 8 | Q96LT4 |
| SAMHD1 | SAM and HD domain containing deoxynucleoside triphosphate triphosphohydrolase 1 | Q9Y3Z3 |
| SAMM50 | SAMM50 sorting and assembly machinery component | Q9Y512 |
| SAMSN1 | SAM domain, SH3 domain and nuclear localization signals 1 | Q9NSI8 |
| SAP130 | Sin3A associated protein 130 | Q9H0E3 |
| SAP18 | Sin3A associated protein 18 | O00422 |
| SAP30BP | SAP30 binding protein | Q9UHR5 |
| SAR1A | Secretion associated Ras related GTPase 1A | Q9NR31 |
| SAR1B | Secretion associated Ras related GTPase 1B | Q9Y6B6 |
| SARAF | Store-operated calcium entry associated regulatory factor | Q96BY9 |
| SARNP | SAP domain containing ribonucleoprotein | P82979 |
| SARS2 | Seryl-tRNA synthetase 2, mitochondrial | Q9NP81 |
| SART1 | Spliceosome associated factor 1, recruiter of U4/U6.U5 tri-snRNP | O43290 |
| SART3 | Spliceosome associated factor 3, U4/U6 recycling protein | Q15020 |
| SASH3 | SAM and SH3 domain containing 3 | O75995 |
| SAT1 | Spermidine/spermine N1-acetyltransferase 1 | P21673 |
| SAT2 | Spermidine/spermine N1-acetyltransferase family member 2 | Q96F10 |
| SATB1 | SATB homeobox 1 | Q01826 |
| SAYSD1 | SAYSVFN motif domain containing 1 | Q9NPB0 |
| SBDS | SBDS ribosome maturation factor | Q9Y3A5 |
| SBF1 | SET binding factor 1 | O95248 |
| SBK1 | SH3 domain binding kinase 1 | Q52WX2 |
| SC5D | Sterol-C5-desaturase | O75845 |
| SCAF11 | SR-related CTD associated factor 11 | Q99590 |
| SCAF4 | SR-related CTD associated factor 4 | O95104 |
| SCAF8 | SR-related CTD associated factor 8 | Q9UPN6 |
| SCAMP1 | Secretory carrier membrane protein 1 | O15126 |
| SCAMP2 | Secretory carrier membrane protein 2 | O15127 |
| SCAMP3 | Secretory carrier membrane protein 3 | O14828 |
| SCAMP4 | Secretory carrier membrane protein 4 | Q969E2 |
| SCAND1 | SCAN domain containing 1 | P57086 |
| SCAP | SREBF chaperone | Q12770 |
| SCAPER | S-phase cyclin A associated protein in the ER | Q9BY12 |
| SCARB2 | Scavenger receptor class B member 2 | Q14108 |
| SCCPDH | Saccharopine dehydrogenase (putative) | Q8NBX0 |
| SCD | Stearoyl-CoA desaturase | O00767 |
| SCFD1 | Sec1 family domain containing 1 | Q8WVM8 |
| SCFD2 | Sec1 family domain containing 2 | Q8WU76 |
| SCGB3A1 | Secretoglobin family 3A member 1 | Q96QR1 |
| SCIMP | SLP adaptor and CSK interacting membrane protein | Q6UWF3 |
| SCLT1 | Sodium channel and clathrin linker 1 | Q96NL6 |
| SCLY | Selenocysteine lyase | Q96I15 |
| SCMH1 | Scm polycomb group protein homolog 1 | Q96GD3 |
| SCML1 | Scm polycomb group protein like 1 | Q9UN30 |
| SCML4 | Scm polycomb group protein like 4 | Q8N228 |
| SCNM1 | Sodium channel modifier 1 | Q9BWG6 |
| SCO1 | Synthesis of cytochrome C oxidase 1 | O75880 |
| SCO2 | Synthesis of cytochrome C oxidase 2 | O43819 |
| SCOC | Short coiled-coil protein | Q9UIL1 |
| SCP2 | Sterol carrier protein 2 | P22307 |
| SCPEP1 | Serine carboxypeptidase 1 | Q9HB40 |
| SCRN1 | Secernin 1 | Q12765 |
| SCRN2 | Secernin 2 | Q96FV2 |
| SCRN3 | Secernin 3 | Q0VDG4 |
| SCYL1 | SCY1 like pseudokinase 1 | Q96KG9 |
| SCYL2 | SCY1 like pseudokinase 2 | Q6P3W7 |
| SCYL3 | SCY1 like pseudokinase 3 | Q8IZE3 |
| SDCBP | Syndecan binding protein | O00560 |
| SDCBP2 | Syndecan binding protein 2 | Q9H190 |
| SDCCAG8 | SHH signaling and ciliogenesis regulator SDCCAG8 | Q86SQ7 |
| SDE2 | SDE2 telomere maintenance homolog | Q6IQ49 |
| SDF2 | Stromal cell derived factor 2 | Q99470 |
| SDF2L1 | Stromal cell derived factor 2 like 1 | Q9HCN8 |
| SDF4 | Stromal cell derived factor 4 |  |
| SDHA | Succinate dehydrogenase complex flavoprotein subunit A | P31040 |
| SDHAF1 | Succinate dehydrogenase complex assembly factor 1 | A6NFY7 |
| SDHAF2 | Succinate dehydrogenase complex assembly factor 2 | Q9NX18 |
| SDHAF3 | Succinate dehydrogenase complex assembly factor 3 | Q9NRP4 |
| SDHAF4 | Succinate dehydrogenase complex assembly factor 4 | Q5VUM1 |
| SDHB | Succinate dehydrogenase complex iron sulfur subunit B | P21912 |
| SDHC | Succinate dehydrogenase complex subunit C | Q99643 |
| SDHD | Succinate dehydrogenase complex subunit D | O14521 |
| SDR39U1 | Short chain dehydrogenase/reductase family 39U member 1 | Q9NRG7 |
| SDR42E1 | Short chain dehydrogenase/reductase family 42E, member 1 | Q8WUS8 |
| SEC11A | SEC11 homolog A, signal peptidase complex subunit | P67812 |
| SEC13 | SEC13 homolog, nuclear pore and COPII coat complex component | P55735 |
| SEC14L2 | SEC14 like lipid binding 2 | O76054 |
| SEC22B | SEC22 homolog B, vesicle trafficking protein | O75396 |
| SEC22C | SEC22 homolog C, vesicle trafficking protein | Q9BRL7 |
| SEC23A | SEC23 homolog A, COPII coat complex component | Q15436 |
| SEC23B | SEC23 homolog B, COPII coat complex component | Q15437 |
| SEC23IP | SEC23 interacting protein | Q9Y6Y8 |
| SEC24C | SEC24 homolog C, COPII coat complex component | P53992 |
| SEC24D | SEC24 homolog D, COPII coat complex component | O94855 |
| SEC31A | SEC31 homolog A, COPII coat complex component | O94979 |
| SEC61A1 | SEC61 translocon subunit alpha 1 | P61619 |
| SEC61B | SEC61 translocon subunit beta | P60468 |
| SEC61G | SEC61 translocon subunit gamma | P60059 |
| SEC62 | SEC62 homolog, preprotein translocation factor | Q99442 |
| SEC63 | SEC63 homolog, protein translocation regulator | Q9UGP8 |
| SECISBP2 | SECIS binding protein 2 | Q96T21 |
| SECISBP2L | SECIS binding protein 2 like | Q93073 |
| SEH1L | SEH1 like nucleoporin | Q96EE3 |
| SEL1L3 | SEL1L family member 3 | Q68CR1 |
| SELL | Selectin L | P14151 |
| SELPLG | Selectin P ligand | Q14242 |
| SEMA4A | Semaphorin 4A | Q9H3S1 |
| SEMA4D | Semaphorin 4D | Q92854 |
| SEMA4F | Ssemaphorin 4F | O95754 |
| SENP1 | SUMO specific peptidase 1 | Q9P0U3 |
| SENP2 | SUMO specific peptidase 2 | Q9HC62 |
| SENP5 | SUMO specific peptidase 5 | Q96HI0 |
| SENP6 | SUMO specific peptidase 6 | Q9GZR1 |
| SENP7 | SUMO specific peptidase 7 | Q9BQF6 |
| SENP8 | SUMO peptidase family member, NEDD8 specific | Q96LD8 |
| SEPHS1 | Selenophosphate synthetase 1 | P49903 |
| SEPTIN1 | Septin 1 | Q8WYJ6 |
| SEPTIN11 | Septin 11 | Q9NVA2 |
| SEPTIN2 | Septin 2 | Q15019 |
| SEPTIN4 | Septin 4 | O43236 |
| SEPTIN6 | Septin 6 | Q14141 |
| SEPTIN7 | Septin 7 | Q16181 |
| SEPTIN8 | Septin 8 | Q92599 |
| SEPTIN9 | Septin 9 | Q9UHD8 |
| SERAC1 | Serine active site containing 1 | Q96JX3 |
| SERBP1 | SERPINE1 mRNA binding protein 1 | Q8NC51 |
| SERF1A | Small EDRK-rich factor 1A | O75920 |
| SERF2 | Small EDRK-rich factor 2 | P84101 |
| SERGEF | Secretion regulating guanine nucleotide exchange factor | Q9UGK8 |
| SERINC1 | Serine incorporator 1 | Q9NRX5 |
| SERINC3 | Serine incorporator 3 | Q13530 |
| SERINC5 | Serine incorporator 5 | Q86VE9 |
| SERP1 | Stress associated endoplasmic reticulum protein 1 | Q9Y6X1 |
| SERPINA1 | Serpin family A member 1 | P01009 |
| SERPINB1 | Serpin family B member 1 | P30740 |
| SERPINB6 | Serpin family B member 6 | P35237 |
| SERPINB8 | Serpin family B member 8 | P50452 |
| SERPINB9 | Serpin family B member 9 | P50453 |
| SERPINE2 | Serpin family E member 2 | P07093 |
| SERPINF1 | Serpin family F member 1 | P36955 |
| SERPINH1 | Serpin family H member 1 | P50454 |
| SERTAD1 | SERTA domain containing 1 | Q9UHV2 |
| SERTAD2 | SERTA domain containing 2 | Q14140 |
| SERTAD3 | SERTA domain containing 3 | Q9UJW9 |
| SESN2 | Sestrin 2 | P58004 |
| SET | SET nuclear proto-oncogene | Q01105 |
| SETD3 | SET domain containing 3, actin histidine methyltransferase | Q86TU7 |
| SETD4 | SET domain containing 4 | Q9NVD3 |
| SETD6 | SET domain containing 6, protein lysine methyltransferase | Q8TBK2 |
| SETD7 | SET domain containing 7, histone lysine methyltransferase | Q8WTS6 |
| SETDB1 | SET domain bifurcated histone lysine methyltransferase 1 | Q15047 |
| SETDB2 | SET domain bifurcated histone lysine methyltransferase 2 | Q96T68 |
| SETMAR | SET domain and mariner transposase fusion gene | Q53H47 |
| SF1 | Splicing factor 1 | Q15637 |
| SF3A1 | Splicing factor 3a subunit 1 | Q15459 |
| SF3A2 | Splicing factor 3a subunit 2 | Q15428 |
| SF3A3 | Splicing factor 3a subunit 3 | Q12874 |
| SF3B2 | Splicing factor 3b subunit 2 | Q13435 |
| SF3B3 | Splicing factor 3b subunit 3 | Q15393 |
| SF3B4 | Splicing factor 3b subunit 4 | Q15427 |
| SF3B5 | Splicing factor 3b subunit 5 | Q9BWJ5 |
| SF3B6 | Splicing factor 3b subunit 6 | Q9Y3B4 |
| SFMBT1 | Scm like with four mbt domains 1 | Q9UHJ3 |
| SFPQ | Splicing factor proline and glutamine rich | P23246 |
| SFR1 | SWI5 dependent homologous recombination repair protein 1 | Q86XK3 |
| SFRP5 | Secreted frizzled related protein 5 | Q5T4F7 |
| SFSWAP | Splicing factor SWAP | Q12872 |
| SFT2D1 | SFT2 domain containing 1 | Q8WV19 |
| SFT2D2 | SFT2 domain containing 2 | O95562 |
| SFXN1 | Sideroflexin 1 | Q9H9B4 |
| SFXN2 | Sideroflexin 2 | Q96NB2 |
| SFXN3 | Sideroflexin 3 | Q9BWM7 |
| SFXN4 | Sideroflexin 4 | Q6P4A7 |
| SGCB | Sarcoglycan beta | Q16585 |
| SGCE | Sarcoglycan epsilon | O43556 |
| SGF29 | SAGA complex associated factor 29 | Q96ES7 |
| SGK1 | Serum/glucocorticoid regulated kinase 1 | O00141 |
| SGK3 | Serum/glucocorticoid regulated kinase family member 3 | Q96BR1 |
| SGPL1 | Sphingosine-1-phosphate lyase 1 | O95470 |
| SGSH | N-sulfoglucosamine sulfohydrolase | P51688 |
| SGSM3 | Small G protein signaling modulator 3 | Q96HU1 |
| SGTA | Small glutamine rich tetratricopeptide repeat co-chaperone alpha | O43765 |
| SGTB | Small glutamine rich tetratricopeptide repeat co-chaperone beta | Q96EQ0 |
| SH2B1 | SH2B adaptor protein 1 | Q9NRF2 |
| SH2D1A | SH2 domain containing 1A | O60880 |
| SH2D1B | SH2 domain containing 1B | O14796 |
| SH2D2A | SH2 domain containing 2A | Q9NP31 |
| SH2D3A | SH2 domain containing 3A | Q9BRG2 |
| SH2D3C | SH2 domain containing 3C | Q8N5H7 |
| SH3BGR | SH3 domain binding glutamate rich protein | P55822 |
| SH3BGRL | SH3 domain binding glutamate rich protein like | O75368 |
| SH3BGRL2 | SH3 domain binding glutamate rich protein like 2 | Q9UJC5 |
| SH3BGRL3 | SH3 domain binding glutamate rich protein like 3 | Q9H299 |
| SH3BP1 | SH3 domain binding protein 1 | Q9Y3L3 |
| SH3BP2 | SH3 domain binding protein 2 | P78314 |
| SH3BP5 | SH3 domain binding protein 5 | O60239 |
| SH3GL1 | SH3 domain containing GRB2 like 1, endophilin A2 | Q99961 |
| SH3GLB1 | SH3 domain containing GRB2 like, endophilin B1 | Q9Y371 |
| SH3GLB2 | SH3 domain containing GRB2 like, endophilin B2 | Q9NR46 |
| SH3TC1 | SH3 domain and tetratricopeptide repeats 1 | Q8TE82 |
| SH3YL1 | SH3 and SYLF domain containing 1 | Q96HL8 |
| SHARPIN | SHANK associated RH domain interactor | Q9H0F6 |
| SHC1 | SHC adaptor protein 1 | P29353 |
| SHISA2 | Shisa family member 2 | Q6UWI4 |
| SHISA5 | Shisa family member 5 | Q8N114 |
| SHKBP1 | SH3KBP1 binding protein 1 | Q8TBC3 |
| SHMT1 | Serine hydroxymethyltransferase 1 | P34896 |
| SHMT2 | Serine hydroxymethyltransferase 2 | P34897 |
| SHOC2 | SHOC2 leucine rich repeat scaffold protein | Q9UQ13 |
| SHPK | Sedoheptulokinase | Q9UHJ6 |
| SHQ1 | SHQ1, H/ACA ribonucleoprotein assembly factor | Q6PI26 |
| SIAE | Sialic acid acetylesterase | Q9HAT2 |
| SIAH1 | Siah E3 ubiquitin protein ligase 1 | Q8IUQ4 |
| SIAH2 | Siah E3 ubiquitin protein ligase 2 | O43255 |
| SIGIRR | Single Ig and TIR domain containing | Q6IA17 |
| SIGLEC9 | Sialic acid binding Ig like lectin 9 | Q9Y336 |
| SIGMAR1 | Sigma non-opioid intracellular receptor 1 | Q99720 |
| SIKE1 | Suppressor of IKBKE 1 | Q9BRV8 |
| SIL1 | SIL1 nucleotide exchange factor | Q9H173 |
| SIMC1 | SUMO interacting motifs containing 1 | Q8NDZ2 |
| SIN3A | SIN3 transcription regulator family member A | Q96ST3 |
| SIN3B | SIN3 transcription regulator family member B | O75182 |
| SIRPB1 | Signal regulatory protein beta 1 | O00241, Q5TFQ8 |
| SIRPG | Signal regulatory protein gamma | Q9P1W8 |
| SIRT1 | Sirtuin 1 | Q96EB6 |
| SIRT2 | Sirtuin 2 | Q8IXJ6 |
| SIRT3 | Sirtuin 3 | Q9NTG7 |
| SIRT4 | Sirtuin 4 | Q9Y6E7 |
| SIRT5 | Sirtuin 5 | Q9NXA8 |
| SIRT6 | Sirtuin 6 | Q8N6T7 |
| SIRT7 | Sirtuin 7 | Q9NRC8 |
| SIVA1 | SIVA1 apoptosis inducing factor | O15304 |
| SKA2 | Spindle and kinetochore associated complex subunit 2 | Q8WVK7 |
| SKAP1 | Src kinase associated phosphoprotein 1 | Q86WV1 |
| SKAP2 | Src kinase associated phosphoprotein 2 | O75563 |
| SKIL | SKI like proto-oncogene | P12757 |
| SKP1 | S-phase kinase associated protein 1 | P63208 |
| SLA | Src like adaptor | Q13239 |
| SLA2 | Src like adaptor 2 | Q9H6Q3 |
| SLAIN1 | SLAIN motif family member 1 | Q8ND83 |
| SLAIN2 | SLAIN motif family member 2 | Q9P270 |
| SLAMF1 | Signaling lymphocytic activation molecule family member 1 | Q13291 |
| SLAMF6 | SLAM family member 6 | Q96DU3 |
| SLAMF7 | SLAM family member 7 | Q9NQ25 |
| SLAMF8 | SLAM family member 8 | Q9P0V8 |
| SLBP | Stem-loop binding protein | Q14493 |
| SLC10A3 | Solute carrier family 10 member 3 | P09131 |
| SLC10A7 | Solute carrier family 10 member 7 | Q0GE19 |
| SLC11A2 | Solute carrier family 11 member 2 | P49281 |
| SLC12A6 | Solute carrier family 12 member 6 | Q9UHW9 |
| SLC12A7 | Solute carrier family 12 member 7 | Q9Y666 |
| SLC14A1 | Solute carrier family 14 member 1 (Kidd blood group) | Q13336 |
| SLC16A1 | Solute carrier family 16 member 1 | P53985 |
| SLC16A10 | Solute carrier family 16 member 10 | Q8TF71 |
| SLC16A11 | Solute carrier family 16 member 11 |  |
| SLC16A3 | Solute carrier family 16 member 3 | O15427 |
| SLC16A7 | Solute carrier family 16 member 7 | O60669 |
| SLC17A5 | Solute carrier family 17 member 5 | Q9NRA2 |
| SLC19A1 | Solute carrier family 19 member 1 | P41440 |
| SLC19A2 | Solute carrier family 19 member 2 | O60779 |
| SLC1A4 | Solute carrier family 1 member 4 | P43007 |
| SLC1A5 | Solute carrier family 1 member 5 | Q15758 |
| SLC20A1 | Solute carrier family 20 member 1 | Q8WUM9 |
| SLC20A2 | Solute carrier family 20 member 2 | Q08357 |
| SLC22A18 | Solute carrier family 22 member 18 | Q96BI1 |
| SLC22A5 | Solute carrier family 22 member 5 | O76082 |
| SLC25A1 | Solute carrier family 25 member 1 | P53007 |
| SLC25A11 | Solute carrier family 25 member 11 | Q02978 |
| SLC25A12 | Solute carrier family 25 member 12 | O75746 |
| SLC25A13 | Solute carrier family 25 member 13 | Q9UJS0 |
| SLC25A14 | Solute carrier family 25 member 14 | O95258 |
| SLC25A15 | Solute carrier family 25 member 15 | Q9Y619 |
| SLC25A17 | Solute carrier family 25 member 17 | O43808 |
| SLC25A19 | Solute carrier family 25 member 19 | Q9HC21 |
| SLC25A20 | Solute carrier family 25 member 20 | O43772 |
| SLC25A22 | Solute carrier family 25 member 22 | Q9H936 |
| SLC25A23 | Solute carrier family 25 member 23 | Q9BV35 |
| SLC25A25 | Solute carrier family 25 member 25 | Q6KCM7 |
| SLC25A26 | Solute carrier family 25 member 26 | Q70HW3 |
| SLC25A28 | Solute carrier family 25 member 28 | Q96A46 |
| SLC25A3 | Solute carrier family 25 member 3 | Q00325 |
| SLC25A32 | Solute carrier family 25 member 32 | Q9H2D1 |
| SLC25A35 | Solute carrier family 25 member 35 | Q3KQZ1 |
| SLC25A36 | Solute carrier family 25 member 36 | Q96CQ1 |
| SLC25A37 | Solute carrier family 25 member 37 | Q9NYZ2 |
| SLC25A38 | Solute carrier family 25 member 38 | Q96DW6 |
| SLC25A39 | Solute carrier family 25 member 39 | Q9BZJ4 |
| SLC25A4 | Solute carrier family 25 member 4 | P12235 |
| SLC25A40 | Solute carrier family 25 member 40 | Q8TBP6 |
| SLC25A42 | Solute carrier family 25 member 42 | Q86VD7 |
| SLC25A43 | Solute carrier family 25 member 43 | Q8WUT9 |
| SLC25A44 | Solute carrier family 25 member 44 | Q96H78 |
| SLC25A45 | Solute carrier family 25 member 45 | Q8N413 |
| SLC25A46 | Solute carrier family 25 member 46 | Q96AG3 |
| SLC25A5 | Solute carrier family 25 member 5 | P05141 |
| SLC25A51 | Solute carrier family 25 member 51 | Q9H1U9 |
| SLC25A6 | Solute carrier family 25 member 6 | P12236 |
| SLC26A6 | Solute carrier family 26 member 6 | Q9BXS9 |
| SLC27A1 | Solute carrier family 27 member 1 | Q6PCB7 |
| SLC27A3 | Solute carrier family 27 member 3 | Q5K4L6 |
| SLC27A5 | Solute carrier family 27 member 5 | Q9Y2P5 |
| SLC29A1 | Solute carrier family 29 member 1 (Augustine blood group) | Q99808 |
| SLC29A2 | Solute carrier family 29 member 2 | Q14542 |
| SLC29A3 | Solute carrier family 29 member 3 | Q9BZD2 |
| SLC2A1 | Solute carrier family 2 member 1 | P11166 |
| SLC2A3 | Solute carrier family 2 member 3 | P11169 |
| SLC2A6 | Solute carrier family 2 member 6 | Q9UGQ3 |
| SLC2A8 | Solute carrier family 2 member 8 | Q9NY64 |
| SLC30A4 | Solute carrier family 30 member 4 | O14863 |
| SLC30A5 | Solute carrier family 30 member 5 | Q8TAD4 |
| SLC30A6 | Solute carrier family 30 member 6 | Q6NXT4 |
| SLC30A7 | Solute carrier family 30 member 7 | Q8NEW0 |
| SLC30A9 | Solute carrier family 30 member 9 | Q6PML9 |
| SLC31A1 | Solute carrier family 31 member 1 | O15431 |
| SLC31A2 | Solute carrier family 31 member 2 | O15432 |
| SLC33A1 | Solute carrier family 33 member 1 | O00400 |
| SLC35A1 | Solute carrier family 35 member A1 | P78382 |
| SLC35A2 | Solute carrier family 35 member A2 | P78381 |
| SLC35A3 | Solute carrier family 35 member A3 | Q9Y2D2 |
| SLC35A4 | Solute carrier family 35 member A4 | L0R6Q1, Q96G79 |
| SLC35A5 | Solute carrier family 35 member A5 | Q9BS91 |
| SLC35B1 | Solute carrier family 35 member B1 | P78383 |
| SLC35B2 | Solute carrier family 35 member B2 | Q8TB61 |
| SLC35B3 | Solute carrier family 35 member B3 | Q9H1N7 |
| SLC35B4 | Solute carrier family 35 member B4 | Q969S0 |
| SLC35C1 | Solute carrier family 35 member C1 | Q96A29 |
| SLC35C2 | Solute carrier family 35 member C2 | Q9NQQ7 |
| SLC35D1 | Solute carrier family 35 member D1 | Q9NTN3 |
| SLC35D2 | Solute carrier family 35 member D2 | Q76EJ3 |
| SLC35E2B | Solute carrier family 35 member E2B | P0CK96 |
| SLC35F2 | Solute carrier family 35 member F2 | Q8IXU6 |
| SLC35F6 | Solute carrier family 35 member F6 | Q8N357 |
| SLC35G2 | Solute carrier family 35 member G2 | Q8TBE7 |
| SLC36A1 | Solute carrier family 36 member 1 | Q7Z2H8 |
| SLC36A4 | Solute carrier family 36 member 4 | Q6YBV0 |
| SLC37A1 | Solute carrier family 37 member 1 | P57057 |
| SLC37A3 | Solute carrier family 37 member 3 | Q8NCC5 |
| SLC37A4 | Solute carrier family 37 member 4 |  |
| SLC38A1 | Solute carrier family 38 member 1 | Q9H2H9 |
| SLC38A10 | Solute carrier family 38 member 10 | Q9HBR0 |
| SLC38A2 | Solute carrier family 38 member 2 | Q96QD8 |
| SLC38A5 | Solute carrier family 38 member 5 | Q8WUX1 |
| SLC38A6 | Solute carrier family 38 member 6 | Q8IZM9 |
| SLC38A7 | Solute carrier family 38 member 7 | Q9NVC3 |
| SLC39A1 | Solute carrier family 39 member 1 | Q9NY26 |
| SLC39A10 | Solute carrier family 39 member 10 | Q9ULF5 |
| SLC39A11 | Solute carrier family 39 member 11 | Q8N1S5 |
| SLC39A13 | Solute carrier family 39 member 13 | Q96H72 |
| SLC39A14 | Solute carrier family 39 member 14 | Q15043 |
| SLC39A3 | Solute carrier family 39 member 3 | Q9BRY0 |
| SLC39A6 | Solute carrier family 39 member 6 | Q13433 |
| SLC39A7 | Solute carrier family 39 member 7 | Q92504 |
| SLC39A8 | Solute carrier family 39 member 8 | Q9C0K1 |
| SLC39A9 | Solute carrier family 39 member 9 | Q9NUM3 |
| SLC3A2 | Solute carrier family 3 member 2 | P08195 |
| SLC40A1 | Solute carrier family 40 member 1 | Q9NP59 |
| SLC41A1 | Solute carrier family 41 member 1 | Q8IVJ1 |
| SLC41A3 | Solute carrier family 41 member 3 | Q96GZ6 |
| SLC43A1 | Solute carrier family 43 member 1 | O75387 |
| SLC43A3 | Solute carrier family 43 member 3 | Q8NBI5 |
| SLC44A2 | Solute carrier family 44 member 2 | Q8IWA5 |
| SLC46A3 | Solute carrier family 46 member 3 | Q7Z3Q1 |
| SLC48A1 | Solute carrier family 48 member 1 | Q6P1K1 |
| SLC4A1AP | Solute carrier family 4 member 1 adaptor protein | Q9BWU0 |
| SLC4A5 | Solute carrier family 4 member 5 | Q9BY07 |
| SLC4A7 | Solute carrier family 4 member 7 | Q9Y6M7 |
| SLC50A1 | Solute carrier family 50 member 1 | Q9BRV3 |
| SLC5A6 | Solute carrier family 5 member 6 | Q9Y289 |
| SLC6A16 | Solute carrier family 6 member 16 | Q9GZN6 |
| SLC6A6 | Solute carrier family 6 member 6 | P31641 |
| SLC7A6 | Solute carrier family 7 member 6 | Q92536 |
| SLC7A6OS | Solute carrier family 7 member 6 opposite strand | Q96CW6 |
| SLC7A8 | Solute carrier family 7 member 8 | Q9UHI5 |
| SLC8B1 | Solute carrier family 8 member B1 | Q6J4K2 |
| SLC9A3R1 | SLC9A3 regulator 1 | O14745 |
| SLC9A6 | Solute carrier family 9 member A6 | Q92581 |
| SLC9B2 | Solute carrier family 9 member B2 | Q86UD5 |
| SLCO3A1 | Solute carrier organic anion transporter family member 3A1 | Q9UIG8 |
| SLCO4C1 | Solute carrier organic anion transporter family member 4C1 | Q6ZQN7 |
| SLF1 | SMC5-SMC6 complex localization factor 1 | Q9BQI6 |
| SLF2 | SMC5-SMC6 complex localization factor 2 | Q8IX21 |
| SLFN11 | Schlafen family member 11 | Q7Z7L1 |
| SLFN5 | Schlafen family member 5 | Q08AF3 |
| SLIRP | SRA stem-loop interacting RNA binding protein | Q9GZT3 |
| SLK | STE20 like kinase | Q9H2G2 |
| SLTM | SAFB like transcription modulator | Q9NWH9 |
| SLU7 | SLU7 homolog, splicing factor | O95391 |
| SLX1A | SLX1 homolog A, structure-specific endonuclease subunit | Q9BQ83 |
| SMAD2 | SMAD family member 2 | Q15796 |
| SMAD3 | SMAD family member 3 | P84022 |
| SMAD4 | SMAD family member 4 | Q13485 |
| SMAD5 | SMAD family member 5 | Q99717 |
| SMAGP | Small cell adhesion glycoprotein | Q0VAQ4 |
| SMAP1 | Small ArfGAP 1 | Q8IYB5 |
| SMAP2 | Small ArfGAP2 | Q8WU79 |
| SMARCA2 | SWI/SNF related, matrix associated, actin dependent regulator of chromatin, subfamily a, member 2 | P51531 |
| SMARCA4 | SWI/SNF related, matrix associated, actin dependent regulator of chromatin, subfamily a, member 4 | P51532 |
| SMARCA5 | SWI/SNF related, matrix associated, actin dependent regulator of chromatin, subfamily a, member 5 | O60264 |
| SMARCAD1 | SWI/SNF-related, matrix-associated actin-dependent regulator of chromatin, subfamily a, containing DEAD/H box 1 | Q9H4L7 |
| SMARCAL1 | SWI/SNF related, matrix associated, actin dependent regulator of chromatin, subfamily a like 1 | Q9NZC9 |
| SMARCB1 | SWI/SNF related, matrix associated, actin dependent regulator of chromatin, subfamily b, member 1 | Q12824 |
| SMARCC1 | SWI/SNF related, matrix associated, actin dependent regulator of chromatin subfamily c member 1 | Q92922 |
| SMARCC2 | SWI/SNF related, matrix associated, actin dependent regulator of chromatin subfamily c member 2 | Q8TAQ2 |
| SMARCD1 | SWI/SNF related, matrix associated, actin dependent regulator of chromatin, subfamily d, member 1 | Q96GM5 |
| SMARCE1 | SWI/SNF related, matrix associated, actin dependent regulator of chromatin, subfamily e, member 1 | Q969G3 |
| SMC1A | Structural maintenance of chromosomes 1A | Q14683 |
| SMC4 | Structural maintenance of chromosomes 4 | Q9NTJ3 |
| SMC5 | Structural maintenance of chromosomes 5 | Q8IY18 |
| SMC6 | Structural maintenance of chromosomes 6 | Q96SB8 |
| SMCO4 | Single-pass membrane protein with coiled-coil domains 4 | Q9NRQ5 |
| SMDT1 | Single-pass membrane protein with aspartate rich tail 1 | Q9H4I9 |
| SMG5 | SMG5 nonsense mediated mRNA decay factor | Q9UPR3 |
| SMG6 | SMG6 nonsense mediated mRNA decay factor | Q86US8 |
| SMG9 | SMG9 nonsense mediated mRNA decay factor | Q9H0W8 |
| SMIM12 | Small integral membrane protein 12 | Q96EX1 |
| SMIM14 | Small integral membrane protein 14 | Q96QK8 |
| SMIM19 | Small integral membrane protein 19 | Q96E16 |
| SMIM24 | Small integral membrane protein 24 | O75264 |
| SMIM3 | Small integral membrane protein 3 | Q9BZL3 |
| SMIM8 | Small integral membrane protein 8 | Q96KF7 |
| SMN1 | Survival of motor neuron 1, telomeric | Q16637 |
| SMNDC1 | Survival motor neuron domain containing 1 | O75940 |
| SMPD1 | Sphingomyelin phosphodiesterase 1 | P17405 |
| SMPD2 | Sphingomyelin phosphodiesterase 2 | O60906 |
| SMS | Spermine synthase | P52788 |
| SMU1 | SMU1 DNA replication regulator and spliceosomal factor | Q2TAY7 |
| SMUG1 | Single-strand-selective monofunctional uracil-DNA glycosylase 1 | Q53HV7 |
| SMURF2 | SMAD specific E3 ubiquitin protein ligase 2 | Q9HAU4 |
| SMYD2 | SET and MYND domain containing 2 | Q9NRG4 |
| SMYD3 | SET and MYND domain containing 3 | Q9H7B4 |
| SMYD4 | SET and MYND domain containing 4 | Q8IYR2 |
| SNAI3 | Snail family transcriptional repressor 3 | Q3KNW1 |
| SNAP23 | Synaptosome associated protein 23 | O00161 |
| SNAP29 | Synaptosome associated protein 29 | O95721 |
| SNAP47 | Synaptosome associated protein 47 | Q5SQN1 |
| SNAPC1 | Small nuclear RNA activating complex polypeptide 1 | Q16533 |
| SNAPC2 | Small nuclear RNA activating complex polypeptide 2 | Q13487 |
| SNAPC5 | Small nuclear RNA activating complex polypeptide 5 | O75971 |
| SNAPIN | SNAP associated protein | O95295 |
| SND1 | Staphylococcal nuclease and tudor domain containing 1 | Q7KZF4 |
| SNF8 | SNF8 subunit of ESCRT-II | Q96H20 |
| SNIP1 | Smad nuclear interacting protein 1 | Q8TAD8 |
| SNN | Stannin | O75324 |
| SNPH | Syntaphilin | O15079 |
| SNRK | SNF related kinase | Q9NRH2 |
| SNRNP200 | Small nuclear ribonucleoprotein U5 subunit 200 | O75643 |
| SNRNP25 | Small nuclear ribonucleoprotein U11/U12 subunit 25 | Q9BV90 |
| SNRNP27 | Small nuclear ribonucleoprotein U4/U6.U5 subunit 27 | Q8WVK2 |
| SNRNP35 | Small nuclear ribonucleoprotein U11/U12 subunit 35 | Q16560 |
| SNRNP40 | Small nuclear ribonucleoprotein U5 subunit 40 | Q96DI7 |
| SNRNP48 | Small nuclear ribonucleoprotein U11/U12 subunit 48 | Q6IEG0 |
| SNRNP70 | Small nuclear ribonucleoprotein U1 subunit 70 | P08621 |
| SNRPA | Small nuclear ribonucleoprotein polypeptide A | P09012 |
| SNRPA1 | Small nuclear ribonucleoprotein polypeptide A' | P09661 |
| SNRPB | Small nuclear ribonucleoprotein polypeptides B and B1 | P14678 |
| SNRPB2 | Small nuclear ribonucleoprotein polypeptide B2 | P08579 |
| SNRPC | Small nuclear ribonucleoprotein polypeptide C | P09234 |
| SNRPD1 | Small nuclear ribonucleoprotein D1 polypeptide | P62314 |
| SNRPD2 | Small nuclear ribonucleoprotein D2 polypeptide | P62316 |
| SNRPD3 | Small nuclear ribonucleoprotein D3 polypeptide | P62318 |
| SNRPE | Small nuclear ribonucleoprotein polypeptide E | P62304 |
| SNRPF | Small nuclear ribonucleoprotein polypeptide F | P62306 |
| SNRPG | Small nuclear ribonucleoprotein polypeptide G | P62308 |
| SNRPN | Small nuclear ribonucleoprotein polypeptide N | P63162 |
| SNTB2 | Syntrophin beta 2 | Q13425 |
| SNU13 | Small nuclear ribonucleoprotein 13 | P55769 |
| SNUPN | Snurportin 1 | O95149 |
| SNURF | SNRPN upstream open reading frame | Q9Y675 |
| SNW1 | SNW domain containing 1 | Q13573 |
| SNX1 | Sorting nexin 1 | Q13596 |
| SNX10 | Sorting nexin 10 | Q9Y5X0 |
| SNX11 | Sorting nexin 11 | Q9Y5W9 |
| SNX12 | Sorting nexin 12 | Q9UMY4 |
| SNX14 | Sorting nexin 14 | Q9Y5W7 |
| SNX15 | Sorting nexin 15 | Q9NRS6 |
| SNX16 | Sorting nexin 16 | P57768 |
| SNX17 | Sorting nexin 17 | Q15036 |
| SNX19 | Sorting nexin 19 | Q92543 |
| SNX2 | Sorting nexin 2 | O60749 |
| SNX20 | Sorting nexin 20 | Q7Z614 |
| SNX29 | Sorting nexin 29 | Q8TEQ0 |
| SNX3 | Sorting nexin 3 | O60493 |
| SNX4 | Sorting nexin 4 | O95219 |
| SNX5 | Sorting nexin 5 | Q9Y5X3 |
| SNX6 | Sorting nexin 6 | Q9UNH7 |
| SNX8 | Sorting nexin 8 | Q9Y5X2 |
| SNX9 | Sorting nexin 9 | Q9Y5X1 |
| SOAT1 | Sterol O-acyltransferase 1 | P35610 |
| SOCS1 | Suppressor of cytokine signaling 1 | O15524 |
| SOCS2 | Suppressor of cytokine signaling 2 | O14508 |
| SOCS5 | Suppressor of cytokine signaling 5 | O75159 |
| SOD1 | Superoxide dismutase 1 | P00441 |
| SOD2 | Superoxide dismutase 2 | P04179 |
| SORBS3 | Sorbin and SH3 domain containing 3 | O60504 |
| SORD | Sorbitol dehydrogenase | Q00796 |
| SOS1 | SOS Ras/Rac guanine nucleotide exchange factor 1 | Q07889 |
| SOX4 | SRY-box transcription factor 4 | Q06945 |
| SP1 | Sp1 transcription factor | P08047 |
| SP100 | SP100 nuclear antigen | P23497 |
| SP110 | SP110 nuclear body protein | Q9HB58 |
| SP140 | SP140 nuclear body protein | Q13342 |
| SP140L | SP140 nuclear body protein like | Q9H930 |
| SP2 | Sp2 transcription factor | Q02086 |
| SP3 | Sp3 transcription factor | Q02447 |
| SP4 | Sp4 transcription factor | Q02446 |
| SPAG16 | Sperm associated antigen 16 | Q8N0X2 |
| SPAG7 | Sperm associated antigen 7 | O75391 |
| SPAST | Spastin | Q9UBP0 |
| SPATA2 | Spermatogenesis associated 2 | Q9UM82 |
| SPATA20 | Spermatogenesis associated 20 | Q8TB22 |
| SPATA24 | Spermatogenesis associated 24 | Q86W54 |
| SPATA2L | Spermatogenesis associated 2 like | Q8IUW3 |
| SPATA33 | Spermatogenesis associated 33 | Q96N06 |
| SPATA5L1 | Spermatogenesis associated 5 like 1 | Q9BVQ7 |
| SPATA7 | Spermatogenesis associated 7 | Q9P0W8 |
| SPATC1L | Spermatogenesis and centriole associated 1 like | Q9H0A9 |
| SPATS2L | Spermatogenesis associated serine rich 2 like | Q9NUQ6 |
| SPC24 | SPC24 component of NDC80 kinetochore complex | Q8NBT2 |
| SPCS1 | Signal peptidase complex subunit 1 | Q9Y6A9 |
| SPCS2 | Signal peptidase complex subunit 2 | Q15005 |
| SPCS3 | Signal peptidase complex subunit 3 | P61009 |
| SPDL1 | Spindle apparatus coiled-coil protein 1 | Q96EA4 |
| SPECC1 | Sperm antigen with calponin homology and coiled-coil domains 1 | Q5M775 |
| SPECC1L | Sperm antigen with calponin homology and coiled-coil domains 1 like | Q69YQ0 |
| SPEF2 | Sperm flagellar 2 | Q9C093 |
| SPG21 | SPG21 abhydrolase domain containing, maspardin | Q9NZD8 |
| SPG7 | SPG7 matrix AAA peptidase subunit, paraplegin | Q9UQ90 |
| SPHK2 | Sphingosine kinase 2 | Q9NRA0 |
| SPICE1 | Spindle and centriole associated protein 1 | Q8N0Z3 |
| SPIDR | Scaffold protein involved in DNA repair | Q14159 |
| SPIN1 | Spindlin 1 | Q9Y657 |
| SPIN2B | Spindlin family member 2B | Q9BPZ2 |
| SPINK2 | Serine peptidase inhibitor Kazal type 2 | P20155 |
| SPINT1 | Serine peptidase inhibitor, Kunitz type 1 | O43278 |
| SPINT2 | Serine peptidase inhibitor, Kunitz type 2 | O43291 |
| SPN | Sialophorin | P16150 |
| SPNS1 | Sphingolipid transporter 1 (putative) | Q9H2V7 |
| SPNS3 | Sphingolipid transporter 3 (putative) | Q6ZMD2 |
| SPOCK2 | SPARC (osteonectin), cwcv and kazal like domains proteoglycan 2 | Q92563 |
| SPON2 | Spondin 2 | Q9BUD6 |
| SPOP | Speckle type BTB/POZ protein | O43791 |
| SPPL2B | Signal peptide peptidase like 2B | Q8TCT7 |
| SPPL3 | Signal peptide peptidase like 3 | Q8TCT6 |
| SPRTN | SprT-like N-terminal domain | Q9H040 |
| SPRY1 | Sprouty RTK signaling antagonist 1 | O43609 |
| SPRYD7 | SPRY domain containing 7 | Q5W111 |
| SPSB2 | SplA/ryanodine receptor domain and SOCS box containing 2 | Q99619 |
| SPSB3 | SplA/ryanodine receptor domain and SOCS box containing 3 | Q6PJ21 |
| SPTLC1 | Serine palmitoyltransferase long chain base subunit 1 | O15269 |
| SPTLC2 | Serine palmitoyltransferase long chain base subunit 2 | O15270 |
| SPTSSA | Serine palmitoyltransferase small subunit A | Q969W0 |
| SPTY2D1 | SPT2 chromatin protein domain containing 1 | Q68D10 |
| SQLE | Squalene epoxidase | Q14534 |
| SQSTM1 | Sequestosome 1 | Q13501 |
| SRA1 | Steroid receptor RNA activator 1 | Q9HD15 |
| SRBD1 | S1 RNA binding domain 1 | Q8N5C6 |
| SRD5A3 | Steroid 5 alpha-reductase 3 | Q9H8P0 |
| SREBF2 | Sterol regulatory element binding transcription factor 2 | Q12772 |
| SREK1 | Splicing regulatory glutamic acid and lysine rich protein 1 | Q8WXA9 |
| SREK1IP1 | SREK1 interacting protein 1 | Q8N9Q2 |
| SRFBP1 | Serum response factor binding protein 1 | Q8NEF9 |
| SRGN | Serglycin | P10124 |
| SRI | Sorcin | P30626 |
| SRM | Spermidine synthase | P19623 |
| SRP14 | Signal recognition particle 14 | P37108 |
| SRP19 | Signal recognition particle 19 | P09132 |
| SRP54 | Signal recognition particle 54 | P61011 |
| SRP68 | Signal recognition particle 68 | Q9UHB9 |
| SRP72 | Signal recognition particle 72 | O76094 |
| SRP9 | Signal recognition particle 9 | P49458 |
| SRPK1 | SRSF protein kinase 1 | Q96SB4 |
| SRPK2 | SRSF protein kinase 2 | P78362 |
| SRPRA | SRP receptor subunit alpha | P08240 |
| SRPRB | SRP receptor subunit beta | Q9Y5M8 |
| SRR | Serine racemase | Q9GZT4 |
| SRRD | SRR1 domain containing | Q9UH36 |
| SRRM1 | Serine and arginine repetitive matrix 1 | Q8IYB3 |
| SRRT | Serrate, RNA effector molecule | Q9BXP5 |
| SRSF1 | Serine and arginine rich splicing factor 1 | Q07955 |
| SRSF10 | Serine and arginine rich splicing factor 10 | O75494 |
| SRSF11 | Serine and arginine rich splicing factor 11 | Q05519 |
| SRSF2 | Serine and arginine rich splicing factor 2 | Q01130 |
| SRSF3 | Serine and arginine rich splicing factor 3 | P84103 |
| SRSF4 | Serine and arginine rich splicing factor 4 | Q08170 |
| SRSF5 | Serine and arginine rich splicing factor 5 | Q13243 |
| SRSF6 | Serine and arginine rich splicing factor 6 | Q13247 |
| SRSF7 | Serine and arginine rich splicing factor 7 | Q16629 |
| SRSF8 | Serine and arginine rich splicing factor 8 | Q9BRL6 |
| SRSF9 | Serine and arginine rich splicing factor 9 | Q13242 |
| SRXN1 | Sulfiredoxin 1 | Q9BYN0 |
| SS18 | SS18 subunit of BAF chromatin remodeling complex | Q15532 |
| SS18L2 | SS18 like 2 | Q9UHA2 |
| SSB | Small RNA binding exonuclease protection factor La | P05455 |
| SSBP1 | Single stranded DNA binding protein 1 | Q04837 |
| SSBP2 | Single stranded DNA binding protein 2 | P81877 |
| SSBP3 | Single stranded DNA binding protein 3 | Q9BWW4 |
| SSBP4 | Single stranded DNA binding protein 4 | Q9BWG4 |
| SSH2 | Slingshot protein phosphatase 2 | Q76I76 |
| SSH3 | Slingshot protein phosphatase 3 | Q8TE77 |
| SSNA1 | SS nuclear autoantigen 1 | O43805 |
| SSR1 | Signal sequence receptor subunit 1 | P43307 |
| SSR2 | Signal sequence receptor subunit 2 | P43308 |
| SSR3 | Signal sequence receptor subunit 3 | Q9UNL2 |
| SSR4 | Signal sequence receptor subunit 4 | P51571 |
| SSRP1 | Structure specific recognition protein 1 | Q08945 |
| SSU72 | SSU72 homolog, RNA polymerase II CTD phosphatase | Q9NP77 |
| SSX2IP | SSX family member 2 interacting protein | Q9Y2D8 |
| ST3GAL1 | ST3 beta-galactoside alpha-2,3-sialyltransferase 1 | Q11201 |
| ST3GAL2 | ST3 beta-galactoside alpha-2,3-sialyltransferase 2 | Q16842 |
| ST3GAL3 | ST3 beta-galactoside alpha-2,3-sialyltransferase 3 | Q11203 |
| ST3GAL4 | ST3 beta-galactoside alpha-2,3-sialyltransferase 4 | Q11206 |
| ST6GAL1 | ST6 beta-galactoside alpha-2,6-sialyltransferase 1 | P15907 |
| ST6GALNAC3 | ST6 N-acetylgalactosaminide alpha-2,6-sialyltransferase 3 | Q8NDV1 |
| ST6GALNAC4 | ST6 N-acetylgalactosaminide alpha-2,6-sialyltransferase 4 | Q9H4F1 |
| ST6GALNAC6 | ST6 N-acetylgalactosaminide alpha-2,6-sialyltransferase 6 | Q969X2 |
| ST7 | Suppression of tumorigenicity 7 | Q9NRC1 |
| ST7L | Suppression of tumorigenicity 7 like | Q8TDW4 |
| ST8SIA4 | ST8 alpha-N-acetyl-neuraminide alpha-2,8-sialyltransferase 4 | Q92187 |
| STAG2 | Stromal antigen 2 | Q8N3U4 |
| STAM | Signal transducing adaptor molecule | Q92783 |
| STAM2 | Signal transducing adaptor molecule 2 | O75886 |
| STAMBP | STAM binding protein | O95630 |
| STAP2 | Signal transducing adaptor family member 2 | Q9UGK3 |
| STARD10 | StAR related lipid transfer domain containing 10 | Q9Y365 |
| STARD3 | StAR related lipid transfer domain containing 3 | Q14849 |
| STARD3NL | STARD3 N-terminal like | O95772 |
| STARD4 | StAR related lipid transfer domain containing 4 | Q96DR4 |
| STARD5 | StAR related lipid transfer domain containing 5 | Q9NSY2 |
| STARD7 | StAR related lipid transfer domain containing 7 | Q9NQZ5 |
| STAT1 | Signal transducer and activator of transcription 1 | P42224 |
| STAT2 | Signal transducer and activator of transcription 2 | P52630 |
| STAT3 | Signal transducer and activator of transcription 3 | P40763 |
| STAT4 | Signal transducer and activator of transcription 4 | Q14765 |
| STAT5A | Signal transducer and activator of transcription 5A | P42229 |
| STAT5B | Signal transducer and activator of transcription 5B | P51692 |
| STAT6 | Signal transducer and activator of transcription 6 | P42226 |
| STAU1 | Staufen double-stranded RNA binding protein 1 | O95793 |
| STAU2 | Staufen double-stranded RNA binding protein 2 | Q9NUL3 |
| STBD1 | Starch binding domain 1 | O95210 |
| STIM1 | Stromal interaction molecule 1 | Q13586 |
| STIP1 | Stress induced phosphoprotein 1 | P31948 |
| STK11 | Serine/threonine kinase 11 | Q15831 |
| STK11IP | Serine/threonine kinase 11 interacting protein | Q8N1F8 |
| STK16 | Serine/threonine kinase 16 | O75716 |
| STK17A | Serine/threonine kinase 17a | Q9UEE5 |
| STK17B | Serine/threonine kinase 17b | O94768 |
| STK19 | Serine/threonine kinase 19 | P49842 |
| STK24 | Serine/threonine kinase 24 | Q9Y6E0 |
| STK25 | Serine/threonine kinase 25 | O00506 |
| STK26 | Serine/threonine kinase 26 | Q9P289 |
| STK3 | Serine/threonine kinase 3 | Q13188 |
| STK32C | Serine/threonine kinase 32C | Q86UX6 |
| STK38 | Serine/threonine kinase 38 | Q15208 |
| STK38L | Serine/threonine kinase 38 like | Q9Y2H1 |
| STK39 | Serine/threonine kinase 39 | Q9UEW8 |
| STK40 | Serine/threonine kinase 40 | Q8N2I9 |
| STMN1 | Stathmin 1 | P16949 |
| STMN3 | Stathmin 3 | Q9NZ72 |
| STOM | Stomatin | P27105 |
| STOML1 | Stomatin like 1 | Q9UBI4 |
| STOML2 | Stomatin like 2 | Q9UJZ1 |
| STPG1 | Sperm tail PG-rich repeat containing 1 | Q5TH74 |
| STRADA | STE20 related adaptor alpha | Q7RTN6 |
| STRADB | STE20 related adaptor beta | Q9C0K7 |
| STRAP | Serine/threonine kinase receptor associated protein | Q9Y3F4 |
| STRBP | Spermatid perinuclear RNA binding protein | Q96SI9 |
| STRIP1 | Striatin interacting protein 1 | Q5VSL9 |
| STT3A | STT3 oligosaccharyltransferase complex catalytic subunit A | P46977 |
| STT3B | STT3 oligosaccharyltransferase complex catalytic subunit B | Q8TCJ2 |
| STUB1 | STIP1 homology and U-box containing protein 1 | Q9UNE7 |
| STX10 | Syntaxin 10 | O60499 |
| STX11 | Syntaxin 11 | O75558 |
| STX12 | Syntaxin 12 | Q86Y82 |
| STX16 | Syntaxin 16 | O14662 |
| STX17 | Syntaxin 17 | P56962 |
| STX18 | Syntaxin 18 | Q9P2W9 |
| STX3 | Syntaxin 3 | Q13277 |
| STX4 | Syntaxin 4 | Q12846 |
| STX5 | Syntaxin 5 | Q13190 |
| STX6 | Syntaxin 6 | O43752 |
| STX7 | Syntaxin 7 | O15400 |
| STX8 | Syntaxin 8 | Q9UNK0 |
| STXBP2 | Syntaxin binding protein 2 | Q15833 |
| STXBP3 | Syntaxin binding protein 3 | O00186 |
| STYK1 | Serine/threonine/tyrosine kinase 1 | Q6J9G0 |
| STYX | Serine/threonine/tyrosine interacting protein | Q8WUJ0 |
| STYXL1 | Serine/threonine/tyrosine interacting like 1 | Q9Y6J8 |
| SUB1 | SUB1 regulator of transcription | P53999 |
| SUCLG1 | Succinate-CoA ligase GDP/ADP-forming subunit alpha | P53597 |
| SUCLG2 | Succinate-CoA ligase GDP-forming subunit beta | Q96I99 |
| SUDS3 | SDS3 homolog, SIN3A corepressor complex component | Q9H7L9 |
| SUFU | SUFU negative regulator of hedgehog signaling | Q9UMX1 |
| SUGP1 | SURP and G-patch domain containing 1 | Q8IWZ8 |
| SUGP2 | SURP and G-patch domain containing 2 | Q8IX01 |
| SUGT1 | SGT1 homolog, MIS12 kinetochore complex assembly cochaperone | Q9Y2Z0 |
| SULT1A1 | Sulfotransferase family 1A member 1 | P50225 |
| SULT1A3 | Sulfotransferase family 1A member 3 | P0DMM9 |
| SULT1B1 | Sulfotransferase family 1B member 1 | O43704 |
| SUMF1 | Sulfatase modifying factor 1 | Q8NBK3 |
| SUMF2 | Sulfatase modifying factor 2 | Q8NBJ7 |
| SUMO1 | Small ubiquitin like modifier 1 | P63165 |
| SUMO2 | Small ubiquitin like modifier 2 | P61956 |
| SUMO3 | Small ubiquitin like modifier 3 | P55854 |
| SUN1 | Sad1 and UNC84 domain containing 1 | O94901 |
| SUN2 | Sad1 and UNC84 domain containing 2 | Q9UH99 |
| SUPT20H | SPT20 homolog, SAGA complex component | Q8NEM7 |
| SUPT3H | SPT3 homolog, SAGA and STAGA complex component | O75486 |
| SUPT4H1 | SPT4 homolog, DSIF elongation factor subunit | P63272 |
| SUPT5H | SPT5 homolog, DSIF elongation factor subunit | O00267 |
| SUPT6H | SPT6 homolog, histone chaperone and transcription elongation factor | Q7KZ85 |
| SUPV3L1 | Suv3 like RNA helicase | Q8IYB8 |
| SURF1 | SURF1 cytochrome c oxidase assembly factor | Q15526 |
| SURF2 | Surfeit 2 | Q15527 |
| SURF4 | Surfeit 4 | O15260 |
| SURF6 | Surfeit 6 | O75683 |
| SUSD3 | Sushi domain containing 3 | Q96L08 |
| SUSD4 | Sushi domain containing 4 | Q5VX71 |
| SUSD6 | Sushi domain containing 6 | Q92537 |
| SUV39H1 | SUV39H1 histone lysine methyltransferase | O43463 |
| SUV39H2 | SUV39H2 histone lysine methyltransferase | Q9H5I1 |
| SVBP | Small vasohibin binding protein | Q8N300 |
| SVIL | Supervillin | O95425 |
| SWAP70 | Switching B cell complex subunit SWAP70 | Q9UH65 |
| SWSAP1 | SWIM-type zinc finger 7 associated protein 1 | Q6NVH7 |
| SWT1 | SWT1 RNA endoribonuclease homolog | Q5T5J6 |
| SYAP1 | Synapse associated protein 1 | Q96A49 |
| SYF2 | SYF2 pre-mRNA splicing factor | O95926 |
| SYMPK | Symplekin scaffold protein | Q92797 |
| SYNCRIP | Synaptotagmin binding cytoplasmic RNA interacting protein | O60506 |
| SYNE2 | Spectrin repeat containing nuclear envelope protein 2 | Q8WXH0 |
| SYNGR1 | Synaptogyrin 1 | O43759 |
| SYNGR2 | Synaptogyrin 2 | O43760 |
| SYNJ2BP | Synaptojanin 2 binding protein | P57105 |
| SYPL1 | Synaptophysin like 1 | Q16563 |
| SYS1 | SYS1 golgi trafficking protein | Q8N2H4 |
| SYT11 | Synaptotagmin 11 | Q9BT88 |
| SYTL1 | Synaptotagmin like 1 | Q8IYJ3 |
| SYTL2 | Synaptotagmin like 2 | Q9HCH5 |
| SYTL3 | Synaptotagmin like 3 | Q4VX76 |
| SYVN1 | Synoviolin 1 | Q86TM6 |
| SZRD1 | SUZ RNA binding domain containing 1 | Q7Z422 |
| TAB1 | TGF-beta activated kinase 1 (MAP3K7) binding protein 1 | Q15750 |
| TAB2 | TGF-beta activated kinase 1 (MAP3K7) binding protein 2 | Q9NYJ8 |
| TACC1 | Transforming acidic coiled-coil containing protein 1 | O75410 |
| TACC3 | Transforming acidic coiled-coil containing protein 3 | Q9Y6A5 |
| TACO1 | Translational activator of cytochrome c oxidase I | Q9BSH4 |
| TADA1 | Transcriptional adaptor 1 | Q96BN2 |
| TADA2A | Transcriptional adaptor 2A |  |
| TADA2B | Transcriptional adaptor 2B | Q86TJ2 |
| TADA3 | Transcriptional adaptor 3 | O75528 |
| TAF1 | TATA-box binding protein associated factor 1 | P21675 |
| TAF10 | TATA-box binding protein associated factor 10 | Q12962 |
| TAF11 | TATA-box binding protein associated factor 11 | Q15544 |
| TAF12 | TATA-box binding protein associated factor 12 | Q16514 |
| TAF13 | TATA-box binding protein associated factor 13 | Q15543 |
| TAF1A | TATA-box binding protein associated factor, RNA polymerase I subunit A | Q15573 |
| TAF1B | TATA-box binding protein associated factor, RNA polymerase I subunit B | Q53T94 |
| TAF1C | TATA-box binding protein associated factor, RNA polymerase I subunit C | Q15572 |
| TAF1D | TATA-box binding protein associated factor, RNA polymerase I subunit D | Q9H5J8 |
| TAF2 | TATA-box binding protein associated factor 2 | Q6P1X5 |
| TAF3 | TATA-box binding protein associated factor 3 | Q5VWG9 |
| TAF6 | TATA-box binding protein associated factor 6 | P49848 |
| TAF7 | TATA-box binding protein associated factor 7 | Q15545 |
| TAF8 | TATA-box binding protein associated factor 8 | Q7Z7C8 |
| TAF9 | TATA-box binding protein associated factor 9 | Q16594 |
| TAF9B | TATA-box binding protein associated factor 9b | Q9HBM6 |
| TAGAP | T cell activation RhoGTPase activating protein | Q8N103 |
| TAGLN2 | Transgelin 2 | P37802 |
| TALDO1 | Transaldolase 1 | P37837 |
| TAMM41 | TAM41 mitochondrial translocator assembly and maintenance homolog | Q96BW9 |
| TANGO2 | Transport and golgi organization 2 homolog | Q6ICL3 |
| TANK | TRAF family member associated NFKB activator | Q92844 |
| TAOK1 | TAO kinase 1 | Q7L7X3 |
| TAOK3 | TAO kinase 3 | Q9H2K8 |
| TAP1 | Transporter 1, ATP binding cassette subfamily B member | Q03518 |
| TAP2 | Transporter 2, ATP binding cassette subfamily B member | Q03519 |
| TAPBP | TAP binding protein | O15533 |
| TAPBPL | TAP binding protein like | Q9BX59 |
| TARBP2 | TARBP2 subunit of RISC loading complex | Q15633 |
| TARDBP | TAR DNA binding protein | Q13148 |
| TARP | TCR gamma alternate reading frame protein |  |
| TASP1 | Taspase 1 | Q9H6P5 |
| TATDN1 | TatD DNase domain containing 1 | Q6P1N9 |
| TATDN3 | TatD DNase domain containing 3 | Q17R31 |
| TAX1BP1 | Tax1 binding protein 1 | Q86VP1 |
| TAX1BP3 | Tax1 binding protein 3 | O14907 |
| TBC1D10A | TBC1 domain family member 10A | Q9BXI6 |
| TBC1D10C | TBC1 domain family member 10C | Q8IV04 |
| TBC1D13 | TBC1 domain family member 13 | Q9NVG8 |
| TBC1D15 | TBC1 domain family member 15 | Q8TC07 |
| TBC1D19 | TBC1 domain family member 19 | Q8N5T2 |
| TBC1D2 | TBC1 domain family member 2 | Q9BYX2 |
| TBC1D20 | TBC1 domain family member 20 | Q96BZ9 |
| TBC1D22A | TBC1 domain family member 22A | Q8WUA7 |
| TBC1D22B | TBC1 domain family member 22B | Q9NU19 |
| TBC1D23 | TBC1 domain family member 23 | Q9NUY8 |
| TBC1D25 | TBC1 domain family member 25 | Q3MII6 |
| TBC1D4 | TBC1 domain family member 4 | O60343 |
| TBC1D5 | TBC1 domain family member 5 | Q92609 |
| TBC1D7 | TBC1 domain family member 7 | Q9P0N9 |
| TBC1D9B | TBC1 domain family member 9B | Q66K14 |
| TBCA | Tubulin folding cofactor A | O75347 |
| TBCB | Tubulin folding cofactor B | Q99426 |
| TBCC | Tubulin folding cofactor C | Q15814 |
| TBCCD1 | TBCC domain containing 1 | Q9NVR7 |
| TBCD | Tubulin folding cofactor D | Q9BTW9 |
| TBCE | Tubulin folding cofactor E | Q15813 |
| TBCK | TBC1 domain containing kinase | Q8TEA7 |
| TBK1 | TANK binding kinase 1 | Q9UHD2 |
| TBL1X | Transducin beta like 1 X-linked | O60907 |
| TBL1XR1 | TBL1X/Y related 1 | Q9BZK7 |
| TBL2 | Transducin beta like 2 | Q9Y4P3 |
| TBL3 | Transducin beta like 3 | Q12788 |
| TBP | TATA-box binding protein | P20226 |
| TBPL1 | TATA-box binding protein like 1 | P62380 |
| TBRG1 | Transforming growth factor beta regulator 1 | Q3YBR2 |
| TBRG4 | Transforming growth factor beta regulator 4 | Q969Z0 |
| TBX21 | T-box transcription factor 21 | Q9UL17 |
| TBXAS1 | Thromboxane A synthase 1 | P24557 |
| TC2N | Tandem C2 domains, nuclear | Q8N9U0 |
| TCAF1 | TRPM8 channel associated factor 1 | Q9Y4C2 |
| TCAF2 | TRPM8 channel associated factor 2 | A6NFQ2 |
| TCAIM | T cell activation inhibitor, mitochondrial | Q8N3R3 |
| TCEA1 | Transcription elongation factor A1 | P23193 |
| TCEA2 | Transcription elongation factor A2 | Q15560 |
| TCEA3 | Transcription elongation factor A3 | O75764 |
| TCEAL1 | Transcription elongation factor A like 1 | Q15170 |
| TCEAL3 | Transcription elongation factor A like 3 | Q969E4 |
| TCEAL4 | Transcription elongation factor A like 4 | Q96EI5 |
| TCEAL8 | Transcription elongation factor A like 8 | Q8IYN2 |
| TCEANC | Transcription elongation factor A N-terminal and central domain containing | Q8N8B7 |
| TCEANC2 | Transcription elongation factor A N-terminal and central domain containing 2 | Q96MN5 |
| TCF12 | Transcription factor 12 | Q99081 |
| TCF19 | Transcription factor 19 | Q9Y242 |
| TCF25 | Transcription factor 25 | Q9BQ70 |
| TCF3 | Transcription factor 3 | P15923 |
| TCF7 | Transcription factor 7 | P36402 |
| TCF7L2 | Transcription factor 7 like 2 | Q9NQB0 |
| TCHP | Trichoplein keratin filament binding | Q9BT92 |
| TCIRG1 | T cell immune regulator 1, ATPase H+ transporting V0 subunit a3 | Q13488 |
| TCOF1 | Treacle ribosome biogenesis factor 1 | Q13428 |
| TCP1 | T-complex 1 | P17987 |
| TCP11L1 | T-complex 11 like 1 | Q9NUJ3 |
| TCP11L2 | T-complex 11 like 2 | Q8N4U5 |
| TCTA | T cell leukemia translocation altered | P57738 |
| TCTN1 | Tectonic family member 1 | Q2MV58 |
| TCTN2 | Tectonic family member 2 | Q96GX1 |
| TCTN3 | Tectonic family member 3 | Q6NUS6 |
| TDG | Thymine DNA glycosylase | Q13569 |
| TDP1 | Tyrosyl-DNA phosphodiesterase 1 | Q9NUW8 |
| TDP2 | Tyrosyl-DNA phosphodiesterase 2 | O95551 |
| TDRD3 | Tudor domain containing 3 | Q9H7E2 |
| TDRD7 | Tudor domain containing 7 | Q8NHU6 |
| TDRKH | Tudor and KH domain containing | Q9Y2W6 |
| TECPR1 | Tectonin beta-propeller repeat containing 1 | Q7Z6L1 |
| TECR | Trans-2,3-enoyl-CoA reductase | Q9NZ01 |
| TEFM | Transcription elongation factor, mitochondrial | Q96QE5 |
| TELO2 | Telomere maintenance 2 | Q9Y4R8 |
| TEN1 | TEN1 subunit of CST complex | Q86WV5 |
| TEPP | Testis, prostate and placenta expressed | Q6URK8 |
| TERF1 | Telomeric repeat binding factor 1 | P54274 |
| TERF2 | Telomeric repeat binding factor 2 | Q15554 |
| TERF2IP | TERF2 interacting protein | Q9NYB0 |
| TES | Testin LIM domain protein | Q9UGI8 |
| TESC | Tescalcin | Q96BS2 |
| TESK1 | Testis associated actin remodelling kinase 1 | Q15569 |
| TESK2 | Testis associated actin remodelling kinase 2 | Q96S53 |
| TESPA1 | Thymocyte expressed, positive selection associated 1 | A2RU30 |
| TEX10 | Testis expressed 10 | Q9NXF1 |
| TEX2 | Testis expressed 2 | Q8IWB9 |
| TEX261 | Testis expressed 261 | Q6UWH6 |
| TEX264 | Testis expressed 264, ER-phagy receptor | Q9Y6I9 |
| TEX30 | Testis expressed 30 | Q5JUR7 |
| TFAM | Transcription factor A, mitochondrial | Q00059 |
| TFB1M | Transcription factor B1, mitochondrial | Q8WVM0 |
| TFB2M | Transcription factor B2, mitochondrial | Q9H5Q4 |
| TFCP2 | Transcription factor CP2 | Q12800 |
| TFDP1 | Transcription factor Dp-1 | Q14186 |
| TFDP2 | Transcription factor Dp-2 | Q14188 |
| TFEB | Transcription factor EB | P19484 |
| TFG | Trafficking from ER to golgi regulator | Q92734 |
| TFIP11 | Tuftelin interacting protein 11 | Q9UBB9 |
| TFPT | TCF3 fusion partner | P0C1Z6 |
| TFRC | Transferrin receptor | P02786 |
| TGDS | TDP-glucose 4,6-dehydratase | O95455 |
| TGFA | Transforming growth factor alpha | P01135 |
| TGFBI | Transforming growth factor beta induced | Q15582 |
| TGFBR1 | Transforming growth factor beta receptor 1 | P36897 |
| TGFBR2 | Transforming growth factor beta receptor 2 | P37173 |
| TGFBR3 | Transforming growth factor beta receptor 3 | Q03167 |
| TGFBRAP1 | Transforming growth factor beta receptor associated protein 1 | Q8WUH2 |
| TGIF1 | TGFB induced factor homeobox 1 | Q15583 |
| TGIF2 | TGFB induced factor homeobox 2 | Q9GZN2 |
| TGOLN2 | Trans-golgi network protein 2 | O43493 |
| TGS1 | Trimethylguanosine synthase 1 | Q96RS0 |
| THADA | THADA armadillo repeat containing | Q6YHU6 |
| THAP1 | THAP domain containing 1 | Q9NVV9 |
| THAP11 | THAP domain containing 11 | Q96EK4 |
| THAP2 | THAP domain containing 2 | Q9H0W7 |
| THAP3 | THAP domain containing 3 | Q8WTV1 |
| THAP4 | THAP domain containing 4 | Q8WY91 |
| THAP5 | THAP domain containing 5 | Q7Z6K1 |
| THAP6 | THAP domain containing 6 | Q8TBB0 |
| THAP7 | THAP domain containing 7 | Q9BT49 |
| THAP8 | THAP domain containing 8 | Q8NA92 |
| THAP9 | THAP domain containing 9 | Q9H5L6 |
| THEM4 | Thioesterase superfamily member 4 | Q5T1C6 |
| THEM6 | Thioesterase superfamily member 6 | Q8WUY1 |
| THEMIS | Thymocyte selection associated | Q8N1K5 |
| THEMIS2 | Thymocyte selection associated family member 2 | Q5TEJ8 |
| THG1L | TRNA-histidine guanylyltransferase 1 like | Q9NWX6 |
| THNSL1 | Threonine synthase like 1 | Q8IYQ7 |
| THOC1 | THO complex subunit 1 | Q96FV9 |
| THOC3 | THO complex subunit 3 | Q96J01 |
| THOC5 | THO complex subunit 5 | Q13769 |
| THOC6 | THO complex subunit 6 | Q86W42 |
| THOC7 | THO complex subunit 7 | Q6I9Y2 |
| THOP1 | Thimet oligopeptidase 1 | P52888 |
| THRA | Thyroid hormone receptor alpha | P10827 |
| THRAP3 | Thyroid hormone receptor associated protein 3 | Q9Y2W1 |
| THTPA | Thiamine triphosphatase | Q9BU02 |
| THUMPD1 | THUMP domain containing 1 | Q9NXG2 |
| THUMPD3 | THUMP domain containing 3 | Q9BV44 |
| THYN1 | Thymocyte nuclear protein 1 | Q9P016 |
| TIA1 | TIA1 cytotoxic granule associated RNA binding protein | P31483 |
| TIAL1 | TIA1 cytotoxic granule associated RNA binding protein like 1 | Q01085 |
| TIFA | TRAF interacting protein with forkhead associated domain | Q96CG3 |
| TIGD1 | Tigger transposable element derived 1 | Q96MW7 |
| TIGD6 | Tigger transposable element derived 6 | Q17RP2 |
| TIGD7 | Tigger transposable element derived 7 | Q6NT04 |
| TIGIT | T cell immunoreceptor with Ig and ITIM domains | Q495A1 |
| TIMD4 | T cell immunoglobulin and mucin domain containing 4 | Q96H15 |
| TIMELESS | Timeless circadian regulator | Q9UNS1 |
| TIMM10 | Translocase of inner mitochondrial membrane 10 | P62072 |
| TIMM10B | Translocase of inner mitochondrial membrane 10B | Q9Y5J6 |
| TIMM13 | Translocase of inner mitochondrial membrane 13 | Q9Y5L4 |
| TIMM17A | Translocase of inner mitochondrial membrane 17A | Q99595 |
| TIMM17B | Translocase of inner mitochondrial membrane 17B | O60830 |
| TIMM21 | Translocase of inner mitochondrial membrane 21 | Q9BVV7 |
| TIMM23 | Translocase of inner mitochondrial membrane 23 | O14925 |
| TIMM44 | Translocase of inner mitochondrial membrane 44 | O43615 |
| TIMM50 | Translocase of inner mitochondrial membrane 50 | Q3ZCQ8 |
| TIMM8A | Translocase of inner mitochondrial membrane 8A | O60220 |
| TIMM8B | Translocase of inner mitochondrial membrane 8 homolog B | Q9Y5J9 |
| TIMM9 | Translocase of inner mitochondrial membrane 9 | Q9Y5J7 |
| TIMMDC1 | Translocase of inner mitochondrial membrane domain containing 1 | Q9NPL8 |
| TIMP1 | TIMP metallopeptidase inhibitor 1 | P01033 |
| TINF2 | TERF1 interacting nuclear factor 2 | Q9BSI4 |
| TIPARP | TCDD inducible poly(ADP-ribose) polymerase | Q7Z3E1 |
| TIPIN | TIMELESS interacting protein | Q9BVW5 |
| TIPRL | TOR signaling pathway regulator | O75663 |
| TIRAP | TIR domain containing adaptor protein | P58753 |
| TK1 | Thymidine kinase 1 | P04183 |
| TK2 | Thymidine kinase 2 | O00142 |
| TKFC | Triokinase and FMN cyclase | Q3LXA3 |
| TKT | Transketolase | P29401 |
| TKTL1 | Transketolase like 1 | P51854 |
| TLE3 | TLE family member 3, transcriptional corepressor | Q04726 |
| TLE4 | TLE family member 4, transcriptional corepressor | Q04727 |
| TLK1 | Tousled like kinase 1 | Q9UKI8 |
| TLK2 | Tousled like kinase 2 | Q86UE8 |
| TLR1 | Toll like receptor 1 | Q15399 |
| TLR3 | Toll like receptor 3 | O15455 |
| TM2D1 | TM2 domain containing 1 | Q9BX74 |
| TM2D2 | TM2 domain containing 2 | Q9BX73 |
| TM2D3 | TM2 domain containing 3 | Q9BRN9 |
| TM4SF19 | Transmembrane 4 L six family member 19 | Q96DZ7 |
| TM7SF2 | Transmembrane 7 superfamily member 2 | O76062 |
| TM9SF1 | Transmembrane 9 superfamily member 1 | O15321 |
| TM9SF2 | Transmembrane 9 superfamily member 2 | Q99805 |
| TM9SF4 | Transmembrane 9 superfamily member 4 | Q92544 |
| TMA16 | Translation machinery associated 16 homolog | Q96EY4 |
| TMA7 | Translation machinery associated 7 homolog | Q9Y2S6 |
| TMBIM1 | Transmembrane BAX inhibitor motif containing 1 | Q969X1 |
| TMBIM4 | Transmembrane BAX inhibitor motif containing 4 | Q9HC24 |
| TMBIM6 | Transmembrane BAX inhibitor motif containing 6 | P55061 |
| TMC6 | Transmembrane channel like 6 | Q7Z403 |
| TMCC1 | Transmembrane and coiled-coil domain family 1 | O94876 |
| TMCC3 | Transmembrane and coiled-coil domain family 3 | Q9ULS5 |
| TMCO1 | Transmembrane and coiled-coil domains 1 | Q9UM00 |
| TMCO3 | Transmembrane and coiled-coil domains 3 | Q6UWJ1 |
| TMCO4 | Transmembrane and coiled-coil domains 4 | Q5TGY1 |
| TMCO6 | Transmembrane and coiled-coil domains 6 | Q96DC7 |
| TMED1 | Transmembrane p24 trafficking protein 1 | Q13445 |
| TMED10 | Transmembrane p24 trafficking protein 10 | P49755 |
| TMED2 | Transmembrane p24 trafficking protein 2 | Q15363 |
| TMED3 | Transmembrane p24 trafficking protein 3 | Q9Y3Q3 |
| TMED4 | Transmembrane p24 trafficking protein 4 | Q7Z7H5 |
| TMED5 | Transmembrane p24 trafficking protein 5 | Q9Y3A6 |
| TMED7 | Transmembrane p24 trafficking protein 7 | Q9Y3B3 |
| TMED9 | Transmembrane p24 trafficking protein 9 | Q9BVK6 |
| TMEM101 | Transmembrane protein 101 | Q96IK0 |
| TMEM104 | Transmembrane protein 104 | Q8NE00 |
| TMEM106A | Transmembrane protein 106A | Q96A25 |
| TMEM106B | Transmembrane protein 106B | Q9NUM4 |
| TMEM106C | Transmembrane protein 106C | Q9BVX2 |
| TMEM107 | Transmembrane protein 107 | Q6UX40 |
| TMEM109 | Transmembrane protein 109 | Q9BVC6 |
| TMEM11 | Transmembrane protein 11 | P17152 |
| TMEM115 | Transmembrane protein 115 | Q12893 |
| TMEM116 | Transmembrane protein 116 | Q8NCL8 |
| TMEM117 | Transmembrane protein 117 | Q9H0C3 |
| TMEM120A | Transmembrane protein 120A | Q9BXJ8 |
| TMEM123 | Transmembrane protein 123 | Q8N131 |
| TMEM126A | Transmembrane protein 126A | Q9H061 |
| TMEM126B | Transmembrane protein 126B | Q8IUX1 |
| TMEM128 | Transmembrane protein 128 | Q5BJH2 |
| TMEM129 | Transmembrane protein 129, E3 ubiquitin ligase | A0AVI4 |
| TMEM134 | Transmembrane protein 134 | Q9H6X4 |
| TMEM135 | Transmembrane protein 135 | Q86UB9 |
| TMEM138 | Transmembrane protein 138 | Q9NPI0 |
| TMEM140 | Transmembrane protein 140 | Q9NV12 |
| TMEM141 | Transmembrane protein 141 | Q96I45 |
| TMEM143 | Transmembrane protein 143 | Q96AN5 |
| TMEM147 | Transmembrane protein 147 | Q9BVK8 |
| TMEM14A | Transmembrane protein 14A | Q9Y6G1 |
| TMEM14B | Transmembrane protein 14B | Q9NUH8 |
| TMEM14C | Transmembrane protein 14C | Q9P0S9 |
| TMEM150A | Transmembrane protein 150A | Q86TG1 |
| TMEM156 | Transmembrane protein 156 | Q8N614 |
| TMEM160 | Transmembrane protein 160 | Q9NX00 |
| TMEM161A | Transmembrane protein 161A | Q9NX61 |
| TMEM161B | Transmembrane protein 161B | Q8NDZ6 |
| TMEM167A | Transmembrane protein 167A | Q8TBQ9 |
| TMEM167B | Transmembrane protein 167B | Q9NRX6 |
| TMEM170A | Transmembrane protein 170A | Q8WVE7 |
| TMEM171 | Transmembrane protein 171 | Q8WVE6 |
| TMEM175 | Transmembrane protein 175 | Q9BSA9 |
| TMEM177 | Transmembrane protein 177 | Q53S58 |
| TMEM179B | Transmembrane protein 179B | Q7Z7N9 |
| TMEM18 | Transmembrane protein 18 | Q96B42 |
| TMEM182 | Transmembrane protein 182 | Q6ZP80 |
| TMEM183A | Transmembrane protein 183A | Q8IXX5 |
| TMEM184B | Transmembrane protein 184B | Q9Y519 |
| TMEM184C | Transmembrane protein 184C | Q9NVA4 |
| TMEM185A | Transmembrane protein 185A | Q8NFB2 |
| TMEM186 | Transmembrane protein 186 | Q96B77 |
| TMEM187 | Transmembrane protein 187 | Q14656 |
| TMEM19 | Transmembrane protein 19 | Q96HH6 |
| TMEM192 | Transmembrane protein 192 | Q8IY95 |
| TMEM199 | Transmembrane protein 199 | Q8N511 |
| TMEM203 | Transmembrane protein 203 | Q969S6 |
| TMEM204 | Transmembrane protein 204 | Q9BSN7 |
| TMEM205 | Transmembrane protein 205 | Q6UW68 |
| TMEM208 | Transmembrane protein 208 | Q9BTX3 |
| TMEM209 | Transmembrane protein 209 | Q96SK2 |
| TMEM214 | Transmembrane protein 214 | Q6NUQ4 |
| TMEM216 | Transmembrane protein 216 | Q9P0N5 |
| TMEM218 | Transmembrane protein 218 | A2RU14 |
| TMEM219 | Transmembrane protein 219 | Q86XT9 |
| TMEM220 | Transmembrane protein 220 | Q6QAJ8 |
| TMEM222 | Transmembrane protein 222 | Q9H0R3 |
| TMEM223 | Transmembrane protein 223 | A0PJW6 |
| TMEM229B | Transmembrane protein 229B | Q8NBD8 |
| TMEM230 | Transmembrane protein 230 | Q96A57 |
| TMEM234 | Transmembrane protein 234 | Q8WY98 |
| TMEM241 | Transmembrane protein 241 | Q24JQ0 |
| TMEM242 | Transmembrane protein 242 | Q9NWH2 |
| TMEM243 | Transmembrane protein 243 | Q9BU79 |
| TMEM248 | Transmembrane protein 248 | Q9NWD8 |
| TMEM25 | Transmembrane protein 25 | Q86YD3 |
| TMEM254 | Transmembrane protein 254 | Q8TBM7 |
| TMEM255A | Transmembrane protein 255A | Q5JRV8 |
| TMEM256 | Transmembrane protein 256 | Q8N2U0 |
| TMEM258 | Transmembrane protein 258 | P61165 |
| TMEM259 | Transmembrane protein 259 | Q4ZIN3 |
| TMEM263 | Transmembrane protein 263 | Q8WUH6 |
| TMEM30A | Transmembrane protein 30A | Q9NV96 |
| TMEM33 | Transmembrane protein 33 | P57088 |
| TMEM38B | Transmembrane protein 38B | Q9NVV0 |
| TMEM39A | Transmembrane protein 39A | Q9NV64 |
| TMEM39B | Transmembrane protein 39B | Q9GZU3 |
| TMEM41A | Transmembrane protein 41A | Q96HV5 |
| TMEM41B | Transmembrane protein 41B | Q5BJD5 |
| TMEM42 | Transmembrane protein 42 | Q69YG0 |
| TMEM43 | Transmembrane protein 43 | Q9BTV4 |
| TMEM50A | Transmembrane protein 50A | O95807 |
| TMEM50B | Transmembrane protein 50B | P56557 |
| TMEM53 | Transmembrane protein 53 | Q6P2H8 |
| TMEM59 | Transmembrane protein 59 | Q9BXS4 |
| TMEM60 | Transmembrane protein 60 | Q9H2L4 |
| TMEM62 | Transmembrane protein 62 | Q0P6H9 |
| TMEM63A | Transmembrane protein 63A | O94886 |
| TMEM68 | Transmembrane protein 68 | Q96MH6 |
| TMEM70 | Transmembrane protein 70 | Q9BUB7 |
| TMEM71 | Transmembrane protein 71 | Q6P5X7 |
| TMEM79 | Transmembrane protein 79 | Q9BSE2 |
| TMEM80 | Transmembrane protein 80 | Q96HE8 |
| TMEM81 | Transmembrane protein 81 | Q6P7N7 |
| TMEM87A | Transmembrane protein 87A | Q8NBN3 |
| TMEM9 | Transmembrane protein 9 | Q9P0T7 |
| TMEM91 | Transmembrane protein 91 | Q6ZNR0 |
| TMEM97 | Transmembrane protein 97 | Q5BJF2 |
| TMEM9B | TMEM9 domain family member B | Q9NQ34 |
| TMF1 | TATA element modulatory factor 1 | P82094 |
| TMIGD2 | Transmembrane and immunoglobulin domain containing 2 | Q96BF3 |
| TMLHE | Trimethyllysine hydroxylase, epsilon | Q9NVH6 |
| TMOD2 | Tropomodulin 2 | Q9NZR1 |
| TMOD3 | Tropomodulin 3 | Q9NYL9 |
| TMPO | Thymopoietin | P42166, P42167 |
| TMPRSS3 | Transmembrane serine protease 3 | P57727 |
| TMSB10 | Thymosin beta 10 | P63313 |
| TMSB4X | Thymosin beta 4 X-linked | P62328 |
| TMTC3 | Transmembrane O-mannosyltransferase targeting cadherins 3 | Q6ZXV5 |
| TMTC4 | Transmembrane O-mannosyltransferase targeting cadherins 4 | Q5T4D3 |
| TMUB1 | Transmembrane and ubiquitin like domain containing 1 | Q9BVT8 |
| TMUB2 | Transmembrane and ubiquitin like domain containing 2 | Q71RG4 |
| TMX1 | Thioredoxin related transmembrane protein 1 | Q9H3N1 |
| TMX2 | Thioredoxin related transmembrane protein 2 | Q9Y320 |
| TMX3 | Thioredoxin related transmembrane protein 3 | Q96JJ7 |
| TMX4 | Thioredoxin related transmembrane protein 4 | Q9H1E5 |
| TNF | Tumor necrosis factor | P01375 |
| TNFAIP3 | TNF alpha induced protein 3 | P21580 |
| TNFAIP8 | TNF alpha induced protein 8 | O95379 |
| TNFAIP8L1 | TNF alpha induced protein 8 like 1 | Q8WVP5 |
| TNFAIP8L2 | TNF alpha induced protein 8 like 2 | Q6P589 |
| TNFRSF10A | TNF receptor superfamily member 10a | O00220 |
| TNFRSF10B | TNF receptor superfamily member 10b | O14763 |
| TNFRSF10D | TNF receptor superfamily member 10d | Q9UBN6 |
| TNFRSF12A | TNF receptor superfamily member 12A | Q9NP84 |
| TNFRSF14 | TNF receptor superfamily member 14 | Q92956 |
| TNFRSF18 | TNF receptor superfamily member 18 | Q9Y5U5 |
| TNFRSF1A | TNF receptor superfamily member 1A | P19438 |
| TNFRSF1B | TNF receptor superfamily member 1B | P20333 |
| TNFRSF25 | TNF receptor superfamily member 25 | Q93038 |
| TNFRSF9 | TNF receptor superfamily member 9 | Q07011 |
| TNFSF10 | TNF superfamily member 10 | P50591 |
| TNFSF12 | TNF superfamily member 12 | O43508 |
| TNFSF13 | TNF superfamily member 13 | O75888 |
| TNFSF13B | TNF superfamily member 13b | Q9Y275 |
| TNFSF14 | TNF superfamily member 14 | O43557 |
| TNFSF8 | TNF superfamily member 8 | P32971 |
| TNIP1 | TNFAIP3 interacting protein 1 | Q15025 |
| TNIP2 | TNFAIP3 interacting protein 2 | Q8NFZ5 |
| TNIP3 | TNFAIP3 interacting protein 3 | Q96KP6 |
| TNK1 | Tyrosine kinase non receptor 1 | Q13470 |
| TNPO1 | Transportin 1 | Q92973 |
| TNPO3 | Transportin 3 | Q9Y5L0 |
| TNRC6C | Trinucleotide repeat containing adaptor 6C | Q9HCJ0 |
| TOB1 | Transducer of ERBB2, 1 | P50616 |
| TOB2 | Transducer of ERBB2, 2 | Q14106 |
| TOE1 | Target of EGR1, exonuclease | Q96GM8 |
| TOLLIP | Toll interacting protein | Q9H0E2 |
| TOM1 | Target of myb1 membrane trafficking protein | O60784 |
| TOM1L2 | Target of myb1 like 2 membrane trafficking protein | Q6ZVM7 |
| TOMM20 | Translocase of outer mitochondrial membrane 20 | Q15388 |
| TOMM22 | Translocase of outer mitochondrial membrane 22 | Q9NS69 |
| TOMM34 | Translocase of outer mitochondrial membrane 34 | Q15785 |
| TOMM40 | Translocase of outer mitochondrial membrane 40 | O96008 |
| TOMM40L | Translocase of outer mitochondrial membrane 40 like | Q969M1 |
| TOMM5 | Translocase of outer mitochondrial membrane 5 | Q8N4H5 |
| TOMM6 | Translocase of outer mitochondrial membrane 6 | Q96B49 |
| TOMM7 | Translocase of outer mitochondrial membrane 7 | Q9P0U1 |
| TOP1MT | DNA topoisomerase I mitochondrial | Q969P6 |
| TOP3B | DNA topoisomerase III beta | O95985 |
| TOR1A | Torsin family 1 member A | O14656 |
| TOR1AIP1 | Torsin 1A interacting protein 1 | Q5JTV8 |
| TOR1AIP2 | Torsin 1A interacting protein 2 | Q8NFQ8, Q9H496 |
| TOR2A | Torsin family 2 member A | Q5JU69, Q8N2E6 |
| TOR3A | Torsin family 3 member A | Q9H497 |
| TOX | Thymocyte selection associated high mobility group box | O94900 |
| TOX4 | TOX high mobility group box family member 4 | O94842 |
| TP53 | Tumor protein p53 | P04637 |
| TP53I13 | Tumor protein p53 inducible protein 13 | Q8NBR0 |
| TP53INP1 | Tumor protein p53 inducible nuclear protein 1 | Q96A56 |
| TP53RK | TP53 regulating kinase | Q96S44 |
| TPCN1 | Two pore segment channel 1 | Q9ULQ1 |
| TPCN2 | Two pore segment channel 2 | Q8NHX9 |
| TPD52 | Tumor protein D52 | P55327 |
| TPD52L2 | TPD52 like 2 | O43399 |
| TPGS1 | Tubulin polyglutamylase complex subunit 1 | Q6ZTW0 |
| TPGS2 | Tubulin polyglutamylase complex subunit 2 | Q68CL5 |
| TPI1 | Triosephosphate isomerase 1 | P60174 |
| TPK1 | Thiamin pyrophosphokinase 1 | Q9H3S4 |
| TPM1 | Tropomyosin 1 | P09493 |
| TPM3 | Tropomyosin 3 | P06753 |
| TPM4 | Tropomyosin 4 | P67936 |
| TPMT | Thiopurine S-methyltransferase | P51580 |
| TPP1 | Tripeptidyl peptidase 1 | O14773 |
| TPP2 | Tripeptidyl peptidase 2 | P29144 |
| TPR | Translocated promoter region, nuclear basket protein | P12270 |
| TPRA1 | Transmembrane protein adipocyte associated 1 | Q86W33 |
| TPRG1 | Tumor protein p63 regulated 1 | Q6ZUI0 |
| TPRG1L | Tumor protein p63 regulated 1 like | Q5T0D9 |
| TPRKB | TP53RK binding protein | Q9Y3C4 |
| TPST1 | Tyrosylprotein sulfotransferase 1 | O60507 |
| TPST2 | Tyrosylprotein sulfotransferase 2 | O60704 |
| TPT1 | Tumor protein, translationally-controlled 1 | P13693 |
| TRA2A | Transformer 2 alpha homolog | Q13595 |
| TRA2B | Transformer 2 beta homolog | P62995 |
| TRABD | TraB domain containing | Q9H4I3 |
| TRAC | T cell receptor alpha constant | P01848 |
| TRADD | TNFRSF1A associated via death domain | Q15628 |
| TRAF1 | TNF receptor associated factor 1 | Q13077 |
| TRAF2 | TNF receptor associated factor 2 | Q12933 |
| TRAF3 | TNF receptor associated factor 3 | Q13114 |
| TRAF3IP2 | TRAF3 interacting protein 2 | O43734 |
| TRAF3IP3 | TRAF3 interacting protein 3 | Q9Y228 |
| TRAF4 | TNF receptor associated factor 4 | Q9BUZ4 |
| TRAF5 | TNF receptor associated factor 5 | O00463 |
| TRAF6 | TNF receptor associated factor 6 | Q9Y4K3 |
| TRAF7 | TNF receptor associated factor 7 | Q6Q0C0 |
| TRAFD1 | TRAF-type zinc finger domain containing 1 | O14545 |
| TRAIP | TRAF interacting protein | Q9BWF2 |
| TRAK2 | Trafficking kinesin protein 2 | O60296 |
| TRAM1 | Translocation associated membrane protein 1 | Q15629 |
| TRAP1 | TNF receptor associated protein 1 | Q12931 |
| TRAPPC1 | Trafficking protein particle complex subunit 1 | Q9Y5R8 |
| TRAPPC10 | Trafficking protein particle complex subunit 10 | P48553 |
| TRAPPC11 | Trafficking protein particle complex subunit 11 | Q7Z392 |
| TRAPPC12 | Trafficking protein particle complex subunit 12 | Q8WVT3 |
| TRAPPC2 | Trafficking protein particle complex subunit 2 | P0DI81 |
| TRAPPC2L | Trafficking protein particle complex subunit 2L | Q9UL33 |
| TRAPPC3 | Trafficking protein particle complex subunit 3 | O43617 |
| TRAPPC4 | Trafficking protein particle complex subunit 4 | Q9Y296 |
| TRAPPC5 | Trafficking protein particle complex subunit 5 | Q8IUR0 |
| TRAPPC6A | Trafficking protein particle complex subunit 6A | O75865 |
| TRAPPC6B | Trafficking protein particle complex subunit 6B | Q86SZ2 |
| TRAPPC8 | Trafficking protein particle complex subunit 8 | Q9Y2L5 |
| TRAPPC9 | Trafficking protein particle complex subunit 9 | Q96Q05 |
| TRAT1 | T cell receptor associated transmembrane adaptor 1 | Q6PIZ9 |
| TRBC1 | T cell receptor beta constant 1 | P01850 |
| TRBC2 | T cell receptor beta constant 2 | A0A5B9 |
| TRDC | T cell receptor delta constant | B7Z8K6 |
| TRDMT1 | TRNA aspartic acid methyltransferase 1 | O14717 |
| TRERF1 | Transcriptional regulating factor 1 | Q96PN7 |
| TREX1 | Three prime repair exonuclease 1 | Q9NSU2 |
| TRGC1 | T cell receptor gamma constant 1 |  |
| TRGC2 | T cell receptor gamma constant 2 |  |
| TRGV3 | T cell receptor gamma variable 3 | P03979 |
| TRIAP1 | TP53 regulated inhibitor of apoptosis 1 | O43715 |
| TRIB2 | Tribbles pseudokinase 2 | Q92519 |
| TRIB3 | Tribbles pseudokinase 3 | Q96RU7 |
| TRIM11 | Tripartite motif containing 11 | Q96F44 |
| TRIM13 | Tripartite motif containing 13 | O60858 |
| TRIM14 | Tripartite motif containing 14 | Q14142 |
| TRIM16 | Tripartite motif containing 16 | O95361 |
| TRIM21 | Tripartite motif containing 21 | P19474 |
| TRIM22 | Tripartite motif containing 22 | Q8IYM9 |
| TRIM23 | Tripartite motif containing 23 | P36406 |
| TRIM25 | Tripartite motif containing 25 | Q14258 |
| TRIM26 | Tripartite motif containing 26 | Q12899 |
| TRIM27 | Tripartite motif containing 27 | P14373 |
| TRIM28 | Tripartite motif containing 28 | Q13263 |
| TRIM32 | Tripartite motif containing 32 | Q13049 |
| TRIM33 | Tripartite motif containing 33 | Q9UPN9 |
| TRIM34 | Tripartite motif containing 34 | Q9BYJ4 |
| TRIM35 | Tripartite motif containing 35 | Q9UPQ4 |
| TRIM37 | Tripartite motif containing 37 | O94972 |
| TRIM38 | Tripartite motif containing 38 | O00635 |
| TRIM39 | Tripartite motif containing 39 | Q9HCM9 |
| TRIM4 | Tripartite motif containing 4 | Q9C037 |
| TRIM41 | Tripartite motif containing 41 | Q8WV44 |
| TRIM44 | Tripartite motif containing 44 | Q96DX7 |
| TRIM47 | Tripartite motif containing 47 | Q96LD4 |
| TRIM5 | Tripartite motif containing 5 | Q9C035 |
| TRIM52 | Tripartite motif containing 52 | Q96A61 |
| TRIM56 | Tripartite motif containing 56 | Q9BRZ2 |
| TRIM59 | Tripartite motif containing 59 | Q8IWR1 |
| TRIM61 | Tripartite motif containing 61 | Q5EBN2 |
| TRIM62 | Tripartite motif containing 62 | Q9BVG3 |
| TRIM65 | Tripartite motif containing 65 | Q6PJ69 |
| TRIM68 | Tripartite motif containing 68 | Q6AZZ1 |
| TRIM8 | Tripartite motif containing 8 | Q9BZR9 |
| TRIOBP | TRIO and F-actin binding protein | Q9H2D6 |
| TRIP10 | Thyroid hormone receptor interactor 10 | Q15642 |
| TRIP4 | Thyroid hormone receptor interactor 4 | Q15650 |
| TRIT1 | TRNA isopentenyltransferase 1 | Q9H3H1 |
| TRMO | TRNA methyltransferase O | Q9BU70 |
| TRMT1 | TRNA methyltransferase 1 | Q9NXH9 |
| TRMT10B | TRNA methyltransferase 10B | Q6PF06 |
| TRMT10C | TRNA methyltransferase 10C, mitochondrial RNase P subunit | Q7L0Y3 |
| TRMT11 | TRNA methyltransferase 11 homolog | Q7Z4G4 |
| TRMT112 | TRNA methyltransferase activator subunit 11-2 | Q9UI30 |
| TRMT12 | TRNA methyltransferase 12 homolog | Q53H54 |
| TRMT13 | TRNA methyltransferase 13 homolog | Q9NUP7 |
| TRMT1L | TRNA methyltransferase 1 like | Q7Z2T5 |
| TRMT2A | TRNA methyltransferase 2 homolog A | Q8IZ69 |
| TRMT2B | TRNA methyltransferase 2 homolog B | Q96GJ1 |
| TRMT5 | TRNA methyltransferase 5 | Q32P41 |
| TRMT6 | TRNA methyltransferase 6 non-catalytic subunit | Q9UJA5 |
| TRMT61A | TRNA methyltransferase 61A | Q96FX7 |
| TRMT61B | TRNA methyltransferase 61B | Q9BVS5 |
| TRMU | TRNA mitochondrial 2-thiouridylase | O75648 |
| TRNAU1AP | TRNA selenocysteine 1 associated protein 1 | Q9NX07 |
| TRNT1 | TRNA nucleotidyl transferase 1 | Q96Q11 |
| TROAP | Trophinin associated protein | Q12815 |
| TRPC4AP | Transient receptor potential cation channel subfamily C member 4 associated protein | Q8TEL6 |
| TRPT1 | TRNA phosphotransferase 1 | Q86TN4 |
| TRPV2 | Transient receptor potential cation channel subfamily V member 2 | Q9Y5S1 |
| TRUB1 | TruB pseudouridine synthase family member 1 | Q8WWH5 |
| TRUB2 | TruB pseudouridine synthase family member 2 | O95900 |
| TSACC | TSSK6 activating cochaperone | Q96A04 |
| TSC1 | TSC complex subunit 1 | Q92574 |
| TSC2 | TSC complex subunit 2 | P49815 |
| TSC22D1 | TSC22 domain family member 1 | Q15714 |
| TSC22D2 | TSC22 domain family member 2 | O75157 |
| TSC22D3 | TSC22 domain family member 3 | Q99576 |
| TSC22D4 | TSC22 domain family member 4 | Q9Y3Q8 |
| TSEN15 | TRNA splicing endonuclease subunit 15 | Q8WW01 |
| TSEN2 | TRNA splicing endonuclease subunit 2 | Q8NCE0 |
| TSFM | Ts translation elongation factor, mitochondrial | P43897 |
| TSG101 | Tumor susceptibility 101 | Q99816 |
| TSHZ1 | Teashirt zinc finger homeobox 1 | Q6ZSZ6 |
| TSN | Translin | Q15631 |
| TSNAX | Translin associated factor X | Q99598 |
| TSPAN14 | Tetraspanin 14 | Q8NG11 |
| TSPAN15 | Tetraspanin 15 | O95858 |
| TSPAN17 | Tetraspanin 17 | Q96FV3 |
| TSPAN2 | Tetraspanin 2 | O60636 |
| TSPAN3 | Tetraspanin 3 | O60637 |
| TSPAN31 | Tetraspanin 31 | Q12999 |
| TSPAN32 | Tetraspanin 32 | Q96QS1 |
| TSPAN4 | Tetraspanin 4 | O14817 |
| TSPAN5 | Tetraspanin 5 | P62079 |
| TSPAN6 | Tetraspanin 6 | O43657 |
| TSPO | Translocator protein | P30536 |
| TSPYL1 | TSPY like 1 | Q9H0U9 |
| TSPYL2 | TSPY like 2 | Q9H2G4 |
| TSR1 | TSR1 ribosome maturation factor | Q2NL82 |
| TSR2 | TSR2 ribosome maturation factor | Q969E8 |
| TSSC4 | Tumor suppressing subtransferable candidate 4 | Q9Y5U2 |
| TSTD2 | Thiosulfate sulfurtransferase like domain containing 2 | Q5T7W7 |
| TTC1 | Tetratricopeptide repeat domain 1 | Q99614 |
| TTC12 | Tetratricopeptide repeat domain 12 | Q9H892 |
| TTC13 | Tetratricopeptide repeat domain 13 | Q8NBP0 |
| TTC14 | Tetratricopeptide repeat domain 14 | Q96N46 |
| TTC16 | Tetratricopeptide repeat domain 16 | Q8NEE8 |
| TTC17 | Tetratricopeptide repeat domain 17 | Q96AE7 |
| TTC27 | Tetratricopeptide repeat domain 27 | Q6P3X3 |
| TTC31 | Tetratricopeptide repeat domain 31 | Q49AM3 |
| TTC32 | Tetratricopeptide repeat domain 32 | Q5I0X7 |
| TTC33 | Tetratricopeptide repeat domain 33 | Q6PID6 |
| TTC38 | Tetratricopeptide repeat domain 38 | Q5R3I4 |
| TTC39B | Tetratricopeptide repeat domain 39B | Q5VTQ0 |
| TTC4 | Tetratricopeptide repeat domain 4 | O95801 |
| TTC5 | Tetratricopeptide repeat domain 5 | Q8N0Z6 |
| TTC9C | Tetratricopeptide repeat domain 9C | Q8N5M4 |
| TTF1 | Transcription termination factor 1 | Q15361 |
| TTF2 | Transcription termination factor 2 | Q9UNY4 |
| TTI2 | TELO2 interacting protein 2 | Q6NXR4 |
| TTLL1 | TTL family tubulin polyglutamylase complex subunit L1 | O95922 |
| TTLL12 | Tubulin tyrosine ligase like 12 | Q14166 |
| TTLL3 | Tubulin tyrosine ligase like 3 | Q9Y4R7 |
| TTLL4 | Tubulin tyrosine ligase like 4 | Q14679 |
| TTN | Titin | Q8WZ42 |
| TTPAL | Alpha tocopherol transfer protein like | Q9BTX7 |
| TTYH2 | Tweety family member 2 | Q9BSA4 |
| TUBA1A | Tubulin alpha 1a | Q71U36 |
| TUBA1B | Tubulin alpha 1b | P68363 |
| TUBA1C | Tubulin alpha 1c | Q9BQE3 |
| TUBA4A | Tubulin alpha 4a | P68366 |
| TUBB | Tubulin beta class I | P07437 |
| TUBB2A | Tubulin beta 2A class IIa | Q13885 |
| TUBB4A | Tubulin beta 4A class IVa | P04350 |
| TUBB4B | Tubulin beta 4B class IVb | P68371 |
| TUBD1 | Tubulin delta 1 | Q9UJT1 |
| TUBE1 | Tubulin epsilon 1 | Q9UJT0 |
| TUBG1 | Tubulin gamma 1 | P23258 |
| TUBGCP2 | Tubulin gamma complex associated protein 2 | Q9BSJ2 |
| TUBGCP3 | Tubulin gamma complex associated protein 3 | Q96CW5 |
| TUBGCP4 | Tubulin gamma complex associated protein 4 | Q9UGJ1 |
| TUBGCP5 | Tubulin gamma complex associated protein 5 | Q96RT8 |
| TUFM | Tu translation elongation factor, mitochondrial | P49411 |
| TULP3 | TUB like protein 3 | O75386 |
| TULP4 | TUB like protein 4 | Q9NRJ4 |
| TUSC2 | Tumor suppressor 2, mitochondrial calcium regulator | O75896 |
| TUT1 | Terminal uridylyl transferase 1, U6 snRNA-specific | Q9H6E5 |
| TVP23B | Trans-golgi network vesicle protein 23 homolog B | Q9NYZ1 |
| TVP23C | Trans-golgi network vesicle protein 23 homolog C | Q96ET8 |
| TWF1 | Twinfilin actin binding protein 1 | Q12792 |
| TWF2 | Twinfilin actin binding protein 2 | Q6IBS0 |
| TWSG1 | Twisted gastrulation BMP signaling modulator 1 | Q9GZX9 |
| TXK | TXK tyrosine kinase | P42681 |
| TXLNA | Taxilin alpha | P40222 |
| TXN | Thioredoxin | P10599 |
| TXN2 | Thioredoxin 2 | Q99757 |
| TXNDC11 | Thioredoxin domain containing 11 | Q6PKC3 |
| TXNDC12 | Thioredoxin domain containing 12 | O95881 |
| TXNDC15 | Thioredoxin domain containing 15 | Q96J42 |
| TXNDC17 | Thioredoxin domain containing 17 | Q9BRA2 |
| TXNDC9 | Thioredoxin domain containing 9 | O14530 |
| TXNIP | Thioredoxin interacting protein | Q9H3M7 |
| TXNL1 | Thioredoxin like 1 | O43396 |
| TXNL4A | Thioredoxin like 4A | P83876 |
| TXNL4B | Thioredoxin like 4B | Q9NX01 |
| TXNRD1 | Thioredoxin reductase 1 | Q16881 |
| TXNRD2 | Thioredoxin reductase 2 | Q9NNW7 |
| TYK2 | Tyrosine kinase 2 | P29597 |
| TYMP | Thymidine phosphorylase | P19971 |
| TYMS | Thymidylate synthetase | P04818 |
| TYROBP | Transmembrane immune signaling adaptor TYROBP | O43914 |
| TYSND1 | Trypsin like peroxisomal matrix peptidase 1 | Q2T9J0 |
| TYW1 | TRNA-yW synthesizing protein 1 homolog | Q9NV66 |
| TYW1B | TRNA-yW synthesizing protein 1 homolog B | Q6NUM6 |
| TYW3 | TRNA-yW synthesizing protein 3 homolog | Q6IPR3 |
| U2AF1 | U2 small nuclear RNA auxiliary factor 1 | Q01081 |
| U2AF1L4 | U2 small nuclear RNA auxiliary factor 1 like 4 | Q8WU68 |
| U2SURP | U2 snRNP associated SURP domain containing | O15042 |
| UAP1 | UDP-N-acetylglucosamine pyrophosphorylase 1 | Q16222 |
| UBA1 | Ubiquitin like modifier activating enzyme 1 | P22314 |
| UBA2 | Ubiquitin like modifier activating enzyme 2 | Q9UBT2 |
| UBA3 | Ubiquitin like modifier activating enzyme 3 | Q8TBC4 |
| UBA5 | Ubiquitin like modifier activating enzyme 5 | Q9GZZ9 |
| UBA52 | Ubiquitin A-52 residue ribosomal protein fusion product 1 | P62987 |
| UBA6 | Ubiquitin like modifier activating enzyme 6 | A0AVT1 |
| UBA7 | Ubiquitin like modifier activating enzyme 7 | P41226 |
| UBAC1 | UBA domain containing 1 | Q9BSL1 |
| UBAC2 | UBA domain containing 2 | Q8NBM4 |
| UBALD1 | UBA like domain containing 1 | Q8TB05 |
| UBALD2 | UBA like domain containing 2 | Q8IYN6 |
| UBAP1 | Ubiquitin associated protein 1 | Q9NZ09 |
| UBAP2 | Ubiquitin associated protein 2 | Q5T6F2 |
| UBASH3A | Ubiquitin associated and SH3 domain containing A | P57075 |
| UBASH3B | Ubiquitin associated and SH3 domain containing B | Q8TF42 |
| UBB | Ubiquitin B | P0CG47 |
| UBE2A | Ubiquitin conjugating enzyme E2 A | P49459 |
| UBE2B | Ubiquitin conjugating enzyme E2 B | P63146 |
| UBE2C | Ubiquitin conjugating enzyme E2 C | O00762 |
| UBE2D1 | Ubiquitin conjugating enzyme E2 D1 | P51668 |
| UBE2D2 | Ubiquitin conjugating enzyme E2 D2 | P62837 |
| UBE2D3 | Ubiquitin conjugating enzyme E2 D3 | P61077 |
| UBE2D4 | Ubiquitin conjugating enzyme E2 D4 (putative) | Q9Y2X8 |
| UBE2E1 | Ubiquitin conjugating enzyme E2 E1 | P51965 |
| UBE2E2 | Ubiquitin conjugating enzyme E2 E2 | Q96LR5 |
| UBE2E3 | Ubiquitin conjugating enzyme E2 E3 | Q969T4 |
| UBE2F | Ubiquitin conjugating enzyme E2 F (putative) | Q969M7 |
| UBE2G1 | Ubiquitin conjugating enzyme E2 G1 | P62253 |
| UBE2G2 | Ubiquitin conjugating enzyme E2 G2 | P60604 |
| UBE2H | Ubiquitin conjugating enzyme E2 H | P62256 |
| UBE2I | Ubiquitin conjugating enzyme E2 I | P63279 |
| UBE2J1 | Ubiquitin conjugating enzyme E2 J1 | Q9Y385 |
| UBE2J2 | Ubiquitin conjugating enzyme E2 J2 | Q8N2K1 |
| UBE2K | Ubiquitin conjugating enzyme E2 K | P61086 |
| UBE2L3 | Ubiquitin conjugating enzyme E2 L3 | P68036 |
| UBE2L6 | Ubiquitin conjugating enzyme E2 L6 | O14933 |
| UBE2M | Ubiquitin conjugating enzyme E2 M | P61081 |
| UBE2N | Ubiquitin conjugating enzyme E2 N | P61088 |
| UBE2O | Ubiquitin conjugating enzyme E2 O | Q9C0C9 |
| UBE2Q1 | Ubiquitin conjugating enzyme E2 Q1 | Q7Z7E8 |
| UBE2Q2 | Ubiquitin conjugating enzyme E2 Q2 | Q8WVN8 |
| UBE2R2 | Ubiquitin conjugating enzyme E2 R2 | Q712K3 |
| UBE2S | Ubiquitin conjugating enzyme E2 S | Q16763 |
| UBE2T | Ubiquitin conjugating enzyme E2 T | Q9NPD8 |
| UBE2V1 | Ubiquitin conjugating enzyme E2 V1 | Q13404 |
| UBE2V2 | Ubiquitin conjugating enzyme E2 V2 | Q15819 |
| UBE2W | Ubiquitin conjugating enzyme E2 W | Q96B02 |
| UBE2Z | Ubiquitin conjugating enzyme E2 Z | Q9H832 |
| UBE3A | Ubiquitin protein ligase E3A | Q05086 |
| UBE3B | Ubiquitin protein ligase E3B | Q7Z3V4 |
| UBE3C | Ubiquitin protein ligase E3C | Q15386 |
| UBE3D | Ubiquitin protein ligase E3D | Q7Z6J8 |
| UBE4A | Ubiquitination factor E4A | Q14139 |
| UBFD1 | Ubiquitin family domain containing 1 | O14562 |
| UBIAD1 | UbiA prenyltransferase domain containing 1 | Q9Y5Z9 |
| UBL3 | Ubiquitin like 3 | O95164 |
| UBL4A | Ubiquitin like 4A | P11441 |
| UBL5 | Ubiquitin like 5 | Q9BZL1 |
| UBL7 | Ubiquitin like 7 | Q96S82 |
| UBOX5 | U-box domain containing 5 | O94941 |
| UBP1 | Upstream binding protein 1 | Q9NZI7 |
| UBQLN1 | Ubiquilin 1 | Q9UMX0 |
| UBQLN2 | Ubiquilin 2 | Q9UHD9 |
| UBR2 | Ubiquitin protein ligase E3 component n-recognin 2 | Q8IWV8 |
| UBXN1 | UBX domain protein 1 | Q04323 |
| UBXN11 | UBX domain protein 11 | Q5T124 |
| UBXN2A | UBX domain protein 2A | P68543 |
| UBXN2B | UBX domain protein 2B | Q14CS0 |
| UBXN4 | UBX domain protein 4 | Q92575 |
| UBXN6 | UBX domain protein 6 | Q9BZV1 |
| UBXN7 | UBX domain protein 7 | O94888 |
| UBXN8 | UBX domain protein 8 | O00124 |
| UCHL3 | Ubiquitin C-terminal hydrolase L3 | P15374 |
| UCHL5 | Ubiquitin C-terminal hydrolase L5 | Q9Y5K5 |
| UCK1 | Uridine-cytidine kinase 1 | Q9HA47 |
| UCK2 | Uridine-cytidine kinase 2 | Q9BZX2 |
| UCKL1 | Uridine-cytidine kinase 1 like 1 | Q9NWZ5 |
| UCP2 | Uncoupling protein 2 | P55851 |
| UEVLD | UEV and lactate/malate dehyrogenase domains | Q8IX04 |
| UFC1 | Ubiquitin-fold modifier conjugating enzyme 1 | Q9Y3C8 |
| UFL1 | UFM1 specific ligase 1 | O94874 |
| UFM1 | Ubiquitin fold modifier 1 | P61960 |
| UFSP1 | UFM1 specific peptidase 1 (inactive) | Q6NVU6 |
| UFSP2 | UFM1 specific peptidase 2 | Q9NUQ7 |
| UGCG | UDP-glucose ceramide glucosyltransferase | Q16739 |
| UGDH | UDP-glucose 6-dehydrogenase | O60701 |
| UGGT1 | UDP-glucose glycoprotein glucosyltransferase 1 | Q9NYU2 |
| UGP2 | UDP-glucose pyrophosphorylase 2 | Q16851 |
| UHMK1 | U2AF homology motif kinase 1 | Q8TAS1 |
| UHRF1 | Ubiquitin like with PHD and ring finger domains 1 | Q96T88 |
| UHRF2 | Ubiquitin like with PHD and ring finger domains 2 | Q96PU4 |
| UIMC1 | Ubiquitin interaction motif containing 1 | Q96RL1 |
| ULBP2 | UL16 binding protein 2 | Q9BZM5 |
| ULK3 | Unc-51 like kinase 3 | Q6PHR2 |
| ULK4 | Unc-51 like kinase 4 | Q96C45 |
| UMPS | Uridine monophosphate synthetase | P11172 |
| UNC119 | Unc-119 lipid binding chaperone | Q13432 |
| UNC13D | Unc-13 homolog D | Q70J99 |
| UNC45A | Unc-45 myosin chaperone A | Q9H3U1 |
| UNC50 | Unc-50 inner nuclear membrane RNA binding protein | Q53HI1 |
| UNG | Uracil DNA glycosylase | P13051 |
| UPF3A | UPF3A regulator of nonsense mediated mRNA decay | Q9H1J1 |
| UPP1 | Uridine phosphorylase 1 | Q16831 |
| UPRT | Uracil phosphoribosyltransferase homolog | Q96BW1 |
| UQCC1 | Ubiquinol-cytochrome c reductase complex assembly factor 1 | Q9NVA1 |
| UQCC2 | Ubiquinol-cytochrome c reductase complex assembly factor 2 | Q9BRT2 |
| UQCR10 | Ubiquinol-cytochrome c reductase, complex III subunit X | Q9UDW1 |
| UQCR11 | Ubiquinol-cytochrome c reductase, complex III subunit XI | O14957 |
| UQCRB | Ubiquinol-cytochrome c reductase binding protein | P14927 |
| UQCRC1 | Ubiquinol-cytochrome c reductase core protein 1 | P31930 |
| UQCRC2 | Ubiquinol-cytochrome c reductase core protein 2 | P22695 |
| UQCRFS1 | Ubiquinol-cytochrome c reductase, Rieske iron-sulfur polypeptide 1 | P47985 |
| UQCRH | Ubiquinol-cytochrome c reductase hinge protein | P07919 |
| UQCRQ | Ubiquinol-cytochrome c reductase complex III subunit VII | O14949 |
| URB2 | URB2 ribosome biogenesis homolog | Q14146 |
| URGCP | Upregulator of cell proliferation | Q8TCY9 |
| URI1 | URI1 prefoldin like chaperone | O94763 |
| URM1 | Ubiquitin related modifier 1 | Q9BTM9 |
| UROD | Uroporphyrinogen decarboxylase | P06132 |
| UROS | Uroporphyrinogen III synthase | P10746 |
| USB1 | U6 snRNA biogenesis phosphodiesterase 1 | Q9BQ65 |
| USE1 | Unconventional SNARE in the ER 1 | Q9NZ43 |
| USF1 | Upstream transcription factor 1 | P22415 |
| USF2 | Upstream transcription factor 2, c-fos interacting | Q15853 |
| USO1 | USO1 vesicle transport factor | O60763 |
| USP1 | Ubiquitin specific peptidase 1 | O94782 |
| USP10 | Ubiquitin specific peptidase 10 | Q14694 |
| USP11 | Ubiquitin specific peptidase 11 | P51784 |
| USP13 | Ubiquitin specific peptidase 13 | Q92995 |
| USP14 | Ubiquitin specific peptidase 14 | P54578 |
| USP15 | Ubiquitin specific peptidase 15 | Q9Y4E8 |
| USP16 | Ubiquitin specific peptidase 16 | Q9Y5T5 |
| USP18 | Ubiquitin specific peptidase 18 | Q9UMW8 |
| USP19 | Ubiquitin specific peptidase 19 | O94966 |
| USP20 | Ubiquitin specific peptidase 20 | Q9Y2K6 |
| USP21 | Ubiquitin specific peptidase 21 | Q9UK80 |
| USP22 | Ubiquitin specific peptidase 22 | Q9UPT9 |
| USP24 | Ubiquitin specific peptidase 24 | Q9UPU5 |
| USP25 | Ubiquitin specific peptidase 25 | Q9UHP3 |
| USP28 | Ubiquitin specific peptidase 28 | Q96RU2 |
| USP3 | Ubiquitin specific peptidase 3 | Q9Y6I4 |
| USP30 | Ubiquitin specific peptidase 30 | Q70CQ3 |
| USP32 | Ubiquitin specific peptidase 32 | Q8NFA0 |
| USP33 | Ubiquitin specific peptidase 33 | Q8TEY7 |
| USP36 | Ubiquitin specific peptidase 36 | Q9P275 |
| USP38 | Ubiquitin specific peptidase 38 | Q8NB14 |
| USP39 | Ubiquitin specific peptidase 39 | Q53GS9 |
| USP4 | Ubiquitin specific peptidase 4 | Q13107 |
| USP42 | Ubiquitin specific peptidase 42 | Q9H9J4 |
| USP44 | Ubiquitin specific peptidase 44 | Q9H0E7 |
| USP45 | Ubiquitin specific peptidase 45 | Q70EL2 |
| USP47 | Ubiquitin specific peptidase 47 | Q96K76 |
| USP48 | Ubiquitin specific peptidase 48 | Q86UV5 |
| USP5 | Ubiquitin specific peptidase 5 | P45974 |
| USP7 | Ubiquitin specific peptidase 7 | Q93009 |
| USP8 | Ubiquitin specific peptidase 8 | P40818 |
| USP9X | Ubiquitin specific peptidase 9 X-linked | Q93008 |
| USPL1 | Ubiquitin specific peptidase like 1 | Q5W0Q7 |
| UTP14A | UTP14A small subunit processome component | Q9BVJ6 |
| UTP15 | UTP15 small subunit processome component | Q8TED0 |
| UTP18 | UTP18 small subunit processome component | Q9Y5J1 |
| UTP23 | UTP23 small subunit processome component | Q9BRU9 |
| UTP3 | UTP3 small subunit processome component | Q9NQZ2 |
| UTP4 | UTP4 small subunit processome component | Q969X6 |
| UTP6 | UTP6 small subunit processome component | Q9NYH9 |
| UTS2 | Urotensin 2 | O95399 |
| UVRAG | UV radiation resistance associated | Q9P2Y5 |
| UXS1 | UDP-glucuronate decarboxylase 1 | Q8NBZ7 |
| UXT | Ubiquitously expressed prefoldin like chaperone | Q9UBK9 |
| VAC14 | VAC14 component of PIKFYVE complex | Q08AM6 |
| VAMP1 | Vesicle associated membrane protein 1 | P23763 |
| VAMP2 | Vesicle associated membrane protein 2 | P63027 |
| VAMP3 | Vesicle associated membrane protein 3 | Q15836 |
| VAMP4 | Vesicle associated membrane protein 4 | O75379 |
| VAMP5 | Vesicle associated membrane protein 5 | O95183 |
| VAMP8 | Vesicle associated membrane protein 8 | Q9BV40 |
| VANGL1 | VANGL planar cell polarity protein 1 | Q8TAA9 |
| VAPA | VAMP associated protein A | Q9P0L0 |
| VAPB | VAMP associated protein B and C | O95292 |
| VASP | Vasodilator stimulated phosphoprotein | P50552 |
| VAT1 | Vesicle amine transport 1 | Q99536 |
| VAV3 | Vav guanine nucleotide exchange factor 3 | Q9UKW4 |
| VBP1 | VHL binding protein 1 | P61758 |
| VCL | Vinculin | P18206 |
| VCP | Valosin containing protein | P55072 |
| VDAC1 | Voltage dependent anion channel 1 | P21796 |
| VDAC2 | Voltage dependent anion channel 2 | P45880 |
| VDAC3 | Voltage dependent anion channel 3 | Q9Y277 |
| VEGFB | Vascular endothelial growth factor B | P49765 |
| VEZF1 | Vascular endothelial zinc finger 1 | Q14119 |
| VGLL4 | Vestigial like family member 4 | Q14135 |
| VHL | Von Hippel-Lindau tumor suppressor | P40337 |
| VILL | Villin like | O15195 |
| VIM | Vimentin | P08670 |
| VIPR1 | Vasoactive intestinal peptide receptor 1 | P32241 |
| VIT | Vitrin | Q6UXI7 |
| VKORC1 | Vitamin K epoxide reductase complex subunit 1 | Q9BQB6 |
| VKORC1L1 | Vitamin K epoxide reductase complex subunit 1 like 1 | Q8N0U8 |
| VMA21 | Vacuolar ATPase assembly factor VMA21 | Q3ZAQ7 |
| VMAC | Vimentin type intermediate filament associated coiled-coil protein | Q2NL98 |
| VMP1 | Vacuole membrane protein 1 | Q96GC9 |
| VNN2 | Vanin 2 | O95498 |
| VOPP1 | VOPP1 WW domain binding protein | Q96AW1 |
| VPS16 | VPS16 core subunit of CORVET and HOPS complexes | Q9H269 |
| VPS18 | VPS18 core subunit of CORVET and HOPS complexes | Q9P253 |
| VPS25 | Vacuolar protein sorting 25 homolog | Q9BRG1 |
| VPS26A | VPS26 retromer complex component A | O75436 |
| VPS26B | VPS26 retromer complex component B | Q4G0F5 |
| VPS29 | VPS29 retromer complex component | Q9UBQ0 |
| VPS33A | VPS33A core subunit of CORVET and HOPS complexes | Q96AX1 |
| VPS33B | VPS33B late endosome and lysosome associated | Q9H267 |
| VPS36 | Vacuolar protein sorting 36 homolog | Q86VN1 |
| VPS37A | VPS37A subunit of ESCRT-I | Q8NEZ2 |
| VPS37B | VPS37B subunit of ESCRT-I | Q9H9H4 |
| VPS39 | VPS39 subunit of HOPS complex | Q96JC1 |
| VPS41 | VPS41 subunit of HOPS complex | P49754 |
| VPS45 | Vacuolar protein sorting 45 homolog | Q9NRW7 |
| VPS4A | Vacuolar protein sorting 4 homolog A | Q9UN37 |
| VPS4B | Vacuolar protein sorting 4 homolog B | O75351 |
| VPS50 | VPS50 subunit of EARP/GARPII complex | Q96JG6 |
| VPS52 | VPS52 subunit of GARP complex | Q8N1B4 |
| VPS53 | VPS53 subunit of GARP complex | Q5VIR6 |
| VPS72 | Vacuolar protein sorting 72 homolog | Q15906 |
| VRK1 | VRK serine/threonine kinase 1 | Q99986 |
| VRK2 | VRK serine/threonine kinase 2 | Q86Y07 |
| VRK3 | VRK serine/threonine kinase 3 | Q8IV63 |
| VSIG1 | V-set and immunoglobulin domain containing 1 | Q86XK7 |
| VTA1 | Vesicle trafficking 1 | Q9NP79 |
| VTI1A | Vesicle transport through interaction with t-SNAREs 1A | Q96AJ9 |
| VTI1B | Vesicle transport through interaction with t-SNAREs 1B | Q9UEU0 |
| VWA5A | Von Willebrand factor A domain containing 5A | O00534 |
| VWA8 | Von Willebrand factor A domain containing 8 | A3KMH1 |
| WAC | WW domain containing adaptor with coiled-coil | Q9BTA9 |
| WARS2 | Tryptophanyl tRNA synthetase 2, mitochondrial | Q9UGM6 |
| WASF2 | WASP family member 2 | Q9Y6W5 |
| WASL | WASP like actin nucleation promoting factor | O00401 |
| WBP1 | WW domain binding protein 1 | Q96G27 |
| WBP11 | WW domain binding protein 11 | Q9Y2W2 |
| WBP1L | WW domain binding protein 1 like | Q9NX94 |
| WBP2 | WW domain binding protein 2 | Q969T9 |
| WBP4 | WW domain binding protein 4 | O75554 |
| WDFY1 | WD repeat and FYVE domain containing 1 | Q8IWB7 |
| WDFY2 | WD repeat and FYVE domain containing 2 | Q96P53 |
| WDHD1 | WD repeat and HMG-box DNA binding protein 1 | O75717 |
| WDR1 | WD repeat domain 1 | O75083 |
| WDR11 | WD repeat domain 11 | Q9BZH6 |
| WDR12 | WD repeat domain 12 | Q9GZL7 |
| WDR13 | WD repeat domain 13 | Q9H1Z4 |
| WDR18 | WD repeat domain 18 | Q9BV38 |
| WDR20 | WD repeat domain 20 | Q8TBZ3 |
| WDR24 | WD repeat domain 24 | Q96S15 |
| WDR25 | WD repeat domain 25 | Q64LD2 |
| WDR3 | WD repeat domain 3 | Q9UNX4 |
| WDR33 | WD repeat domain 33 | Q9C0J8 |
| WDR36 | WD repeat domain 36 |  |
| WDR37 | WD repeat domain 37 | Q9Y2I8 |
| WDR4 | WD repeat domain 4 | P57081 |
| WDR41 | WD repeat domain 41 | Q9HAD4 |
| WDR43 | WD repeat domain 43 | Q15061 |
| WDR45 | WD repeat domain 45 | Q9Y484 |
| WDR45B | WD repeat domain 45B | Q5MNZ6 |
| WDR47 | WD repeat domain 47 | O94967 |
| WDR48 | WD repeat domain 48 | Q8TAF3 |
| WDR5 | WD repeat domain 5 | P61964 |
| WDR53 | WD repeat domain 53 | Q7Z5U6 |
| WDR54 | WD repeat domain 54 | Q9H977 |
| WDR55 | WD repeat domain 55 | Q9H6Y2 |
| WDR59 | WD repeat domain 59 | Q6PJI9 |
| WDR5B | WD repeat domain 5B | Q86VZ2 |
| WDR6 | WD repeat domain 6 | Q9NNW5 |
| WDR70 | WD repeat domain 70 | Q9NW82 |
| WDR73 | WD repeat domain 73 | Q6P4I2 |
| WDR74 | WD repeat domain 74 | Q6RFH5 |
| WDR75 | WD repeat domain 75 | Q8IWA0 |
| WDR76 | WD repeat domain 76 | Q9H967 |
| WDR77 | WD repeat domain 77 | Q9BQA1 |
| WDR82 | WD repeat domain 82 | Q6UXN9 |
| WDR83 | WD repeat domain 83 | Q9BRX9 |
| WDR83OS | WD repeat domain 83 opposite strand | Q9Y284 |
| WDR89 | WD repeat domain 89 | Q96FK6 |
| WDR91 | WD repeat domain 91 | A4D1P6 |
| WDSUB1 | WD repeat, sterile alpha motif and U-box domain containing 1 | Q8N9V3 |
| WEE1 | WEE1 G2 checkpoint kinase | P30291 |
| WFS1 | Wolframin ER transmembrane glycoprotein | O76024 |
| WHAMM | WASP homolog associated with actin, golgi membranes and microtubules | Q8TF30 |
| WIPF1 | WAS/WASL interacting protein family member 1 | O43516 |
| WIPI1 | WD repeat domain, phosphoinositide interacting 1 | Q5MNZ9 |
| WIPI2 | WD repeat domain, phosphoinositide interacting 2 | Q9Y4P8 |
| WNK1 | WNK lysine deficient protein kinase 1 | Q9H4A3 |
| WNT1 | Wnt family member 1 | P04628 |
| WRAP53 | WD repeat containing antisense to TP53 | Q9BUR4 |
| WRAP73 | WD repeat containing, antisense to TP73 | Q9P2S5 |
| WRNIP1 | WRN helicase interacting protein 1 | Q96S55 |
| WSB1 | WD repeat and SOCS box containing 1 | Q9Y6I7 |
| WSB2 | WD repeat and SOCS box containing 2 | Q9NYS7 |
| WTAP | WT1 associated protein | Q15007 |
| WWC3 | WWC family member 3 | Q9ULE0 |
| WWOX | WW domain containing oxidoreductase | Q9NZC7 |
| WWP1 | WW domain containing E3 ubiquitin protein ligase 1 | Q9H0M0 |
| WWP2 | WW domain containing E3 ubiquitin protein ligase 2 | O00308 |
| XAB2 | XPA binding protein 2 | Q9HCS7 |
| XAF1 | XIAP associated factor 1 | Q6GPH4 |
| XBP1 | X-box binding protein 1 | P17861 |
| XCL1 | X-C motif chemokine ligand 1 | P47992 |
| XCL2 | X-C motif chemokine ligand 2 | Q9UBD3 |
| XIAP | X-linked inhibitor of apoptosis | P98170 |
| XKR8 | XK related 8 | Q9H6D3 |
| XKRX | XK related X-linked | Q6PP77 |
| XPA | XPA, DNA damage recognition and repair factor | P23025 |
| XPC | XPC complex subunit, DNA damage recognition and repair factor | Q01831 |
| XPNPEP1 | X-prolyl aminopeptidase 1 | Q9NQW7 |
| XPNPEP3 | X-prolyl aminopeptidase 3 | Q9NQH7 |
| XPO1 | Exportin 1 | O14980 |
| XPO4 | Exportin 4 | Q9C0E2 |
| XPO5 | Exportin 5 | Q9HAV4 |
| XPO7 | Exportin 7 | Q9UIA9 |
| XPOT | Exportin for tRNA | O43592 |
| XPR1 | Xenotropic and polytropic retrovirus receptor 1 | Q9UBH6 |
| XRCC1 | X-ray repair cross complementing 1 | P18887 |
| XRCC4 | X-ray repair cross complementing 4 | Q13426 |
| XRCC5 | X-ray repair cross complementing 5 | P13010 |
| XRCC6 | X-ray repair cross complementing 6 | P12956 |
| XRN1 | 5'-3' exoribonuclease 1 | Q8IZH2 |
| XRN2 | 5'-3' exoribonuclease 2 | Q9H0D6 |
| XRRA1 | X-ray radiation resistance associated 1 | Q6P2D8 |
| XXYLT1 | Xyloside xylosyltransferase 1 | Q8NBI6 |
| YAF2 | YY1 associated factor 2 | Q8IY57 |
| YARS2 | Tyrosyl-tRNA synthetase 2 | Q9Y2Z4 |
| YBEY | YbeY metalloendoribonuclease | P58557 |
| YBX1 | Y-box binding protein 1 | P67809 |
| YBX3 | Y-box binding protein 3 | P16989 |
| YEATS2 | YEATS domain containing 2 | Q9ULM3 |
| YEATS4 | YEATS domain containing 4 | O95619 |
| YES1 | YES proto-oncogene 1, Src family tyrosine kinase | P07947 |
| YIF1A | Yip1 interacting factor homolog A, membrane trafficking protein | O95070 |
| YIF1B | Yip1 interacting factor homolog B, membrane trafficking protein | Q5BJH7 |
| YIPF1 | Yip1 domain family member 1 | Q9Y548 |
| YIPF2 | Yip1 domain family member 2 | Q9BWQ6 |
| YIPF3 | Yip1 domain family member 3 | Q9GZM5 |
| YIPF4 | Yip1 domain family member 4 | Q9BSR8 |
| YIPF5 | Yip1 domain family member 5 | Q969M3 |
| YIPF6 | Yip1 domain family member 6 | Q96EC8 |
| YKT6 | YKT6 v-SNARE homolog | O15498 |
| YME1L1 | YME1 like 1 ATPase | Q96TA2 |
| YPEL1 | Yippee like 1 | O60688 |
| YPEL2 | Yippee like 2 | Q96QA6 |
| YPEL3 | Yippee like 3 | P61236 |
| YPEL5 | Yippee like 5 | P62699 |
| YRDC | YrdC N6-threonylcarbamoyltransferase domain containing | Q86U90 |
| YTHDC1 | YTH domain containing 1 | Q96MU7 |
| YTHDF1 | YTH N6-methyladenosine RNA binding protein 1 | Q9BYJ9 |
| YTHDF2 | YTH N6-methyladenosine RNA binding protein 2 | Q9Y5A9 |
| YTHDF3 | YTH N6-methyladenosine RNA binding protein 3 | Q7Z739 |
| YWHAB | Tyrosine 3-monooxygenase/tryptophan 5-monooxygenase activation protein beta | P31946 |
| YWHAE | Tyrosine 3-monooxygenase/tryptophan 5-monooxygenase activation protein epsilon | P62258 |
| YWHAG | Tyrosine 3-monooxygenase/tryptophan 5-monooxygenase activation protein gamma | P61981 |
| YWHAH | Tyrosine 3-monooxygenase/tryptophan 5-monooxygenase activation protein eta | Q04917 |
| YWHAQ | Tyrosine 3-monooxygenase/tryptophan 5-monooxygenase activation protein theta | P27348 |
| YWHAZ | Tyrosine 3-monooxygenase/tryptophan 5-monooxygenase activation protein zeta | P63104 |
| YY1 | YY1 transcription factor | P25490 |
| YY1AP1 | YY1 associated protein 1 | Q9H869 |
| ZAP70 | Zeta chain of T cell receptor associated protein kinase 70 | P43403 |
| ZBED1 | Zinc finger BED-type containing 1 | O96006 |
| ZBED5 | Zinc finger BED-type containing 5 | Q49AG3 |
| ZBP1 | Z-DNA binding protein 1 | Q9H171 |
| ZBTB1 | Zinc finger and BTB domain containing 1 | Q9Y2K1 |
| ZBTB11 | Zinc finger and BTB domain containing 11 | O95625 |
| ZBTB14 | Zinc finger and BTB domain containing 14 | O43829 |
| ZBTB16 | Zinc finger and BTB domain containing 16 | Q05516 |
| ZBTB17 | Zinc finger and BTB domain containing 17 | Q13105 |
| ZBTB2 | Zinc finger and BTB domain containing 2 | Q8N680 |
| ZBTB20 | Zinc finger and BTB domain containing 20 | Q9HC78 |
| ZBTB21 | Zinc finger and BTB domain containing 21 | Q9ULJ3 |
| ZBTB24 | Zinc finger and BTB domain containing 24 | O43167 |
| ZBTB25 | Zinc finger and BTB domain containing 25 | P24278 |
| ZBTB3 | Zinc finger and BTB domain containing 3 | Q9H5J0 |
| ZBTB33 | Zinc finger and BTB domain containing 33 | Q86T24 |
| ZBTB38 | Zinc finger and BTB domain containing 38 | Q8NAP3 |
| ZBTB4 | Zinc finger and BTB domain containing 4 | Q9P1Z0 |
| ZBTB40 | Zinc finger and BTB domain containing 40 | Q9NUA8 |
| ZBTB43 | Zinc finger and BTB domain containing 43 | O43298 |
| ZBTB44 | Zinc finger and BTB domain containing 44 | Q8NCP5 |
| ZBTB48 | Zinc finger and BTB domain containing 48 | P10074 |
| ZBTB49 | Zinc finger and BTB domain containing 49 | Q6ZSB9 |
| ZBTB6 | Zinc finger and BTB domain containing 6 | Q15916 |
| ZBTB8OS | Zinc finger and BTB domain containing 8 opposite strand | Q8IWT0 |
| ZBTB9 | Zinc finger and BTB domain containing 9 | Q96C00 |
| ZC3H10 | Zinc finger CCCH-type containing 10 | Q96K80 |
| ZC3H11A | Zinc finger CCCH-type containing 11A | O75152 |
| ZC3H12A | Zinc finger CCCH-type containing 12A | Q5D1E8 |
| ZC3H13 | Zinc finger CCCH-type containing 13 | Q5T200 |
| ZC3H14 | Zinc finger CCCH-type containing 14 | Q6PJT7 |
| ZC3H15 | Zinc finger CCCH-type containing 15 | Q8WU90 |
| ZC3H18 | Zinc finger CCCH-type containing 18 | Q86VM9 |
| ZC3H3 | Zinc finger CCCH-type containing 3 | Q8IXZ2 |
| ZC3H7A | Zinc finger CCCH-type containing 7A | Q8IWR0 |
| ZC3HAV1 | Zinc finger CCCH-type containing, antiviral 1 | Q7Z2W4 |
| ZC3HC1 | Zinc finger C3HC-type containing 1 | Q86WB0 |
| ZC4H2 | Zinc finger C4H2-type containing | Q9NQZ6 |
| ZCCHC10 | Zinc finger CCHC-type containing 10 | Q8TBK6 |
| ZCCHC12 | Zinc finger CCHC-type containing 12 | Q6PEW1 |
| ZCCHC17 | Zinc finger CCHC-type containing 17 | Q9NP64 |
| ZCCHC3 | Zinc finger CCHC-type containing 3 | Q9NUD5 |
| ZCCHC4 | Zinc finger CCHC-type containing 4 | Q9H5U6 |
| ZCCHC7 | Zinc finger CCHC-type containing 7 | Q8N3Z6 |
| ZCCHC8 | Zinc finger CCHC-type containing 8 | Q6NZY4 |
| ZCCHC9 | Zinc finger CCHC-type containing 9 | Q8N567 |
| ZCRB1 | Zinc finger CCHC-type and RNA binding motif containing 1 | Q8TBF4 |
| ZCWPW1 | Zinc finger CW-type and PWWP domain containing 1 | Q9H0M4 |
| ZDHHC12 | Zinc finger DHHC-type palmitoyltransferase 12 | Q96GR4 |
| ZDHHC13 | Zinc finger DHHC-type palmitoyltransferase 13 | Q8IUH4 |
| ZDHHC16 | Zinc finger DHHC-type palmitoyltransferase 16 | Q969W1 |
| ZDHHC17 | Zinc finger DHHC-type palmitoyltransferase 17 | Q8IUH5 |
| ZDHHC20 | Zinc finger DHHC-type palmitoyltransferase 20 | Q5W0Z9 |
| ZDHHC3 | Zinc finger DHHC-type palmitoyltransferase 3 | Q9NYG2 |
| ZDHHC4 | Zinc finger DHHC-type palmitoyltransferase 4 | Q9NPG8 |
| ZDHHC5 | Zinc finger DHHC-type palmitoyltransferase 5 | Q9C0B5 |
| ZDHHC6 | Zinc finger DHHC-type palmitoyltransferase 6 | Q9H6R6 |
| ZDHHC7 | Zinc finger DHHC-type palmitoyltransferase 7 | Q9NXF8 |
| ZDHHC9 | Zinc finger DHHC-type palmitoyltransferase 9 | Q9Y397 |
| ZEB2 | Zinc finger E-box binding homeobox 2 | O60315 |
| ZER1 | Zyg-11 related cell cycle regulator | Q7Z7L7 |
| ZFAND1 | Zinc finger AN1-type containing 1 | Q8TCF1 |
| ZFAND2A | Zinc finger AN1-type containing 2A | Q8N6M9 |
| ZFAND2B | Zinc finger AN1-type containing 2B | Q8WV99 |
| ZFAND3 | Zinc finger AN1-type containing 3 | Q9H8U3 |
| ZFAND5 | Zinc finger AN1-type containing 5 | O76080 |
| ZFAND6 | Zinc finger AN1-type containing 6 | Q6FIF0 |
| ZFC3H1 | Zinc finger C3H1-type containing | O60293 |
| ZFP1 | ZFP1 zinc finger protein | Q6P2D0 |
| ZFP14 | ZFP14 zinc finger protein | Q9HCL3 |
| ZFP3 | ZFP3 zinc finger protein | Q96NJ6 |
| ZFP30 | ZFP30 zinc finger protein | Q9Y2G7 |
| ZFP36 | ZFP36 ring finger protein | P26651 |
| ZFP36L1 | ZFP36 ring finger protein like 1 | Q07352 |
| ZFP36L2 | ZFP36 ring finger protein like 2 | P47974 |
| ZFP64 | ZFP64 zinc finger protein | Q9NTW7 |
| ZFP69B | ZFP69 zinc finger protein B | Q9UJL9 |
| ZFP82 | ZFP82 zinc finger protein | Q8N141 |
| ZFP91 | ZFP91 zinc finger protein, atypical E3 ubiquitin ligase | Q96JP5 |
| ZFPL1 | Zinc finger protein like 1 | O95159 |
| ZFY | Zinc finger protein Y-linked | P08048 |
| ZFYVE1 | Zinc finger FYVE-type containing 1 | Q9HBF4 |
| ZFYVE16 | Zinc finger FYVE-type containing 16 | Q7Z3T8 |
| ZFYVE19 | Zinc finger FYVE-type containing 19 | Q96K21 |
| ZFYVE21 | Zinc finger FYVE-type containing 21 | Q9BQ24 |
| ZFYVE27 | Zinc finger FYVE-type containing 27 | Q5T4F4 |
| ZFYVE28 | Zinc finger FYVE-type containing 28 | Q9HCC9 |
| ZGPAT | Zinc finger CCCH-type and G-patch domain containing | Q8N5A5 |
| ZHX1 | Zinc fingers and homeoboxes 1 | Q9UKY1 |
| ZHX1-C8orf76 | ZHX1-C8orf76 readthrough | Q96EF9 |
| ZHX2 | Zinc fingers and homeoboxes 2 | Q9Y6X8 |
| ZIK1 | Zinc finger protein interacting with K protein 1 | Q3SY52 |
| ZKSCAN1 | Zinc finger with KRAB and SCAN domains 1 | P17029 |
| ZKSCAN3 | Zinc finger with KRAB and SCAN domains 3 | Q9BRR0 |
| ZKSCAN4 | Zinc finger with KRAB and SCAN domains 4 | Q969J2 |
| ZKSCAN5 | Zinc finger with KRAB and SCAN domains 5 | Q9Y2L8 |
| ZMAT2 | Zinc finger matrin-type 2 | Q96NC0 |
| ZMAT3 | Zinc finger matrin-type 3 | Q9HA38 |
| ZMAT5 | Zinc finger matrin-type 5 | Q9UDW3 |
| ZMPSTE24 | Zinc metallopeptidase STE24 | O75844 |
| ZMYM2 | Zinc finger MYM-type containing 2 | Q9UBW7 |
| ZMYM3 | Zinc finger MYM-type containing 3 | Q14202 |
| ZMYM4 | Zinc finger MYM-type containing 4 | Q5VZL5 |
| ZMYM5 | Zinc finger MYM-type containing 5 | Q9UJ78 |
| ZMYM6 | Zinc finger MYM-type containing 6 | O95789 |
| ZMYND11 | Zinc finger MYND-type containing 11 | Q15326 |
| ZMYND8 | Zinc finger MYND-type containing 8 | Q9ULU4 |
| ZNF10 | Zinc finger protein 10 | P21506 |
| ZNF101 | Zinc finger protein 101 | Q8IZC7 |
| ZNF106 | Zinc finger protein 106 | Q9H2Y7 |
| ZNF121 | Zinc finger protein 121 | P58317 |
| ZNF124 | Zinc finger protein 124 | Q15973 |
| ZNF131 | Zinc finger protein 131 | P52739 |
| ZNF132 | Zinc finger protein 132 | P52740 |
| ZNF133 | Zinc finger protein 133 | P52736 |
| ZNF134 | Zinc finger protein 134 | P52741 |
| ZNF135 | Zinc finger protein 135 | P52742 |
| ZNF136 | Zinc finger protein 136 | P52737 |
| ZNF138 | Zinc finger protein 138 | P52744 |
| ZNF14 | Zinc finger protein 14 | P17017 |
| ZNF140 | Zinc finger protein 140 | P52738 |
| ZNF143 | Zinc finger protein 143 | P52747 |
| ZNF146 | Zinc finger protein 146 | Q15072 |
| ZNF148 | Zinc finger protein 148 | Q9UQR1 |
| ZNF155 | Zinc finger protein 155 | Q12901 |
| ZNF16 | Zinc finger protein 16 | P17020 |
| ZNF160 | Zinc finger protein 160 | Q9HCG1 |
| ZNF165 | Zinc finger protein 165 | P49910 |
| ZNF169 | Zinc finger protein 169 | Q14929 |
| ZNF17 | Zinc finger protein 17 | P17021 |
| ZNF174 | Zinc finger protein 174 | Q15697 |
| ZNF18 | Zinc finger protein 18 | P17022 |
| ZNF180 | Zinc finger protein 180 | Q9UJW8 |
| ZNF181 | Zinc finger protein 181 | Q2M3W8 |
| ZNF182 | Zinc finger protein 182 | P17025 |
| ZNF184 | Zinc finger protein 184 | Q99676 |
| ZNF185 | Zinc finger protein 185 with LIM domain | O15231 |
| ZNF189 | Zinc finger protein 189 | O75820 |
| ZNF19 | Zinc finger protein 19 | P17023 |
| ZNF195 | Zinc finger protein 195 | O14628 |
| ZNF2 | Zinc finger protein 2 | Q9BSG1 |
| ZNF20 | Zinc finger protein 20 | P17024 |
| ZNF200 | Zinc finger protein 200 | P98182 |
| ZNF202 | Zinc finger protein 202 | O95125 |
| ZNF207 | Zinc finger protein 207 | O43670 |
| ZNF212 | Zinc finger protein 212 | Q9UDV6 |
| ZNF213 | Zinc finger protein 213 | O14771 |
| ZNF217 | Zinc finger protein 217 | O75362 |
| ZNF22 | Zinc finger protein 22 | P17026 |
| ZNF222 | Zinc finger protein 222 | Q9UK12 |
| ZNF223 | Zinc finger protein 223 | Q9UK11 |
| ZNF224 | Zinc finger protein 224 | Q9NZL3 |
| ZNF225 | Zinc finger protein 225 | Q9UK10 |
| ZNF226 | Zinc finger protein 226 | Q9NYT6 |
| ZNF23 | Zinc finger protein 23 | P17027 |
| ZNF230 | Zinc finger protein 230 | Q9UIE0 |
| ZNF232 | Zinc finger protein 232 | Q9UNY5 |
| ZNF234 | Zinc finger protein 234 | Q14588 |
| ZNF235 | Zinc finger protein 235 | Q14590 |
| ZNF239 | Zinc finger protein 239 | Q16600 |
| ZNF24 | Zinc finger protein 24 | P17028 |
| ZNF248 | Zinc finger protein 248 | Q8NDW4 |
| ZNF25 | Zinc finger protein 25 | P17030 |
| ZNF250 | Zinc finger protein 250 | P15622 |
| ZNF253 | Zinc finger protein 253 | O75346 |
| ZNF254 | Zinc finger protein 254 | O75437 |
| ZNF256 | Zinc finger protein 256 | Q9Y2P7 |
| ZNF257 | Zinc finger protein 257 | Q9Y2Q1 |
| ZNF26 | Zinc finger protein 26 | P17031 |
| ZNF260 | Zinc finger protein 260 | Q3ZCT1 |
| ZNF263 | Zinc finger protein 263 | O14978 |
| ZNF264 | Zinc finger protein 264 | O43296 |
| ZNF266 | Zinc finger protein 266 | Q14584 |
| ZNF267 | Zinc finger protein 267 | Q14586 |
| ZNF268 | Zinc finger protein 268 | Q14587 |
| ZNF274 | Zinc finger protein 274 | Q96GC6 |
| ZNF276 | Zinc finger protein 276 | Q8N554 |
| ZNF277 | Zinc finger protein 277 | Q9NRM2 |
| ZNF28 | Zinc finger protein 28 | P17035 |
| ZNF280B | Zinc finger protein 280B | Q86YH2 |
| ZNF280C | Zinc finger protein 280C | Q8ND82 |
| ZNF280D | Zinc finger protein 280D | Q6N043 |
| ZNF281 | Zinc finger protein 281 | Q9Y2X9 |
| ZNF283 | Zinc finger protein 283 | Q8N7M2 |
| ZNF284 | Zinc finger protein 284 | Q2VY69 |
| ZNF285 | Zinc finger protein 285 | Q96NJ3 |
| ZNF287 | Zinc finger protein 287 | Q9HBT7 |
| ZNF292 | Zinc finger protein 292 | O60281 |
| ZNF296 | Zinc finger protein 296 | Q8WUU4 |
| ZNF3 | Zinc finger protein 3 | P17036 |
| ZNF30 | Zinc finger protein 30 | P17039 |
| ZNF302 | Zinc finger protein 302 | Q9NR11 |
| ZNF304 | Zinc finger protein 304 | Q9HCX3 |
| ZNF317 | Zinc finger protein 317 | Q96PQ6 |
| ZNF319 | Zinc finger protein 319 | Q9P2F9 |
| ZNF32 | Zinc finger protein 32 | P17041 |
| ZNF320 | Zinc finger protein 320 | A2RRD8 |
| ZNF322 | Zinc finger protein 322 | Q6U7Q0 |
| ZNF324 | Zinc finger protein 324 | O75467 |
| ZNF326 | Zinc finger protein 326 | Q5BKZ1 |
| ZNF329 | Zinc finger protein 329 | Q86UD4 |
| ZNF330 | Zinc finger protein 330 | Q9Y3S2 |
| ZNF331 | Zinc finger protein 331 | Q9NQX6 |
| ZNF333 | Zinc finger protein 333 | Q96JL9 |
| ZNF335 | Zinc finger protein 335 | Q9H4Z2 |
| ZNF337 | Zinc finger protein 337 | Q9Y3M9 |
| ZNF33A | Zinc finger protein 33A | Q06730 |
| ZNF34 | Zinc finger protein 34 | Q8IZ26 |
| ZNF341 | Zinc finger protein 341 | Q9BYN7 |
| ZNF343 | Zinc finger protein 343 | Q6P1L6 |
| ZNF345 | Zinc finger protein 345 | Q14585 |
| ZNF346 | Zinc finger protein 346 | Q9UL40 |
| ZNF35 | Zinc finger protein 35 | P13682 |
| ZNF350 | Zinc finger protein 350 | Q9GZX5 |
| ZNF354A | Zinc finger protein 354A | O60765 |
| ZNF354B | Zinc finger protein 354B | Q96LW1 |
| ZNF362 | Zinc finger protein 362 | Q5T0B9 |
| ZNF365 | Zinc finger protein 365 | Q70YC5 |
| ZNF37A | Zinc finger protein 37A | P17032 |
| ZNF382 | Zinc finger protein 382 | Q96SR6 |
| ZNF383 | Zinc finger protein 383 | Q8NA42 |
| ZNF384 | Zinc finger protein 384 | Q8TF68 |
| ZNF394 | Zinc finger protein 394 | Q53GI3 |
| ZNF395 | Zinc finger protein 395 | Q9H8N7 |
| ZNF396 | Zinc finger protein 396 | Q96N95 |
| ZNF397 | Zinc finger protein 397 | Q8NF99 |
| ZNF398 | Zinc finger protein 398 | Q8TD17 |
| ZNF408 | Zinc finger protein 408 | Q9H9D4 |
| ZNF41 | Zinc finger protein 41 | P51814 |
| ZNF410 | Zinc finger protein 410 | Q86VK4 |
| ZNF415 | Zinc finger protein 415 | Q09FC8 |
| ZNF416 | Zinc finger protein 416 | Q9BWM5 |
| ZNF417 | Zinc finger protein 417 | Q8TAU3 |
| ZNF420 | Zinc finger protein 420 | Q8TAQ5 |
| ZNF425 | Zinc finger protein 425 | Q6IV72 |
| ZNF426 | Zinc finger protein 426 | Q9BUY5 |
| ZNF428 | Zinc finger protein 428 | Q96B54 |
| ZNF43 | Zinc finger protein 43 | P17038 |
| ZNF430 | Zinc finger protein 430 | Q9H8G1 |
| ZNF431 | Zinc finger protein 431 | Q8TF32 |
| ZNF432 | Zinc finger protein 432 | O94892 |
| ZNF433 | Zinc finger protein 433 | Q8N7K0 |
| ZNF438 | Zinc finger protein 438 | Q7Z4V0 |
| ZNF44 | Zinc finger protein 44 | P15621 |
| ZNF440 | Zinc finger protein 440 | Q8IYI8 |
| ZNF441 | Zinc finger protein 441 | Q8N8Z8 |
| ZNF443 | Zinc finger protein 443 | Q9Y2A4 |
| ZNF444 | Zinc finger protein 444 | Q8N0Y2 |
| ZNF446 | Zinc finger protein 446 | Q9NWS9 |
| ZNF449 | Zinc finger protein 449 | Q6P9G9 |
| ZNF45 | Zinc finger protein 45 | Q02386 |
| ZNF451 | Zinc finger protein 451 | Q9Y4E5 |
| ZNF461 | Zinc finger protein 461 | Q8TAF7 |
| ZNF468 | Zinc finger protein 468 | Q5VIY5 |
| ZNF473 | Zinc finger protein 473 | Q8WTR7 |
| ZNF480 | Zinc finger protein 480 | Q8WV37 |
| ZNF483 | Zinc finger protein 483 | Q8TF39 |
| ZNF485 | Zinc finger protein 485 | Q8NCK3 |
| ZNF486 | Zinc finger protein 486 | Q96H40 |
| ZNF490 | Zinc finger protein 490 | Q9ULM2 |
| ZNF493 | Zinc finger protein 493 | Q6ZR52 |
| ZNF496 | Zinc finger protein 496 | Q96IT1 |
| ZNF497 | Zinc finger protein 497 | Q6ZNH5 |
| ZNF502 | Zinc finger protein 502 | Q8TBZ5 |
| ZNF506 | Zinc finger protein 506 | Q5JVG8 |
| ZNF511 | Zinc finger protein 511 | Q8NB15 |
| ZNF512 | Zinc finger protein 512 | Q96ME7 |
| ZNF514 | Zinc finger protein 514 | Q96K75 |
| ZNF524 | Zinc finger protein 524 | Q96C55 |
| ZNF526 | Zinc finger protein 526 | Q8TF50 |
| ZNF527 | Zinc finger protein 527 | Q8NB42 |
| ZNF528 | Zinc finger protein 528 | Q3MIS6 |
| ZNF529 | Zinc finger protein 529 | Q6P280 |
| ZNF530 | Zinc finger protein 530 | Q6P9A1 |
| ZNF540 | Zinc finger protein 540 | Q8NDQ6 |
| ZNF543 | Zinc finger protein 543 | Q08ER8 |
| ZNF544 | Zinc finger protein 544 | Q6NX49 |
| ZNF546 | Zinc finger protein 546 | Q86UE3 |
| ZNF547 | Zinc finger protein 547 | Q8IVP9 |
| ZNF548 | Zinc finger protein 548 | Q8NEK5 |
| ZNF549 | Zinc finger protein 549 | Q6P9A3 |
| ZNF550 | Zinc finger protein 550 |  |
| ZNF551 | Zinc finger protein 551 | Q7Z340 |
| ZNF552 | Zinc finger protein 552 | Q9H707 |
| ZNF555 | Zinc finger protein 555 | Q8NEP9 |
| ZNF557 | Zinc finger protein 557 | Q8N988 |
| ZNF558 | Zinc finger protein 558 | Q96NG5 |
| ZNF559 | Zinc finger protein 559 | Q9BR84 |
| ZNF561 | Zinc finger protein 561 | Q8N587 |
| ZNF562 | Zinc finger protein 562 | Q6V9R5 |
| ZNF563 | Zinc finger protein 563 | Q8TA94 |
| ZNF564 | Zinc finger protein 564 | Q8TBZ8 |
| ZNF565 | Zinc finger protein 565 | Q8N9K5 |
| ZNF566 | Zinc finger protein 566 | Q969W8 |
| ZNF567 | Zinc finger protein 567 | Q8N184 |
| ZNF57 | Zinc finger protein 57 | Q68EA5 |
| ZNF571 | Zinc finger protein 571 | Q7Z3V5 |
| ZNF573 | Zinc finger protein 573 | Q86YE8 |
| ZNF574 | Zinc finger protein 574 | Q6ZN55 |
| ZNF575 | Zinc finger protein 575 | Q86XF7 |
| ZNF576 | Zinc finger protein 576 | Q9H609 |
| ZNF577 | Zinc finger protein 577 | Q9BSK1 |
| ZNF580 | Zinc finger protein 580 | Q9UK33 |
| ZNF581 | Zinc finger protein 581 | Q9P0T4 |
| ZNF582 | Zinc finger protein 582 | Q96NG8 |
| ZNF583 | Zinc finger protein 583 | Q96ND8 |
| ZNF584 | Zinc finger protein 584 | Q8IVC4 |
| ZNF585A | Zinc finger protein 585A | Q6P3V2 |
| ZNF585B | Zinc finger protein 585B | Q52M93 |
| ZNF586 | Zinc finger protein 586 | Q9NXT0 |
| ZNF589 | Zinc finger protein 589 | Q86UQ0 |
| ZNF592 | Zinc finger protein 592 | Q92610 |
| ZNF593 | Zinc finger protein 593 | O00488 |
| ZNF595 | Zinc finger protein 595 | Q8IYB9 |
| ZNF596 | Zinc finger protein 596 | Q8TC21 |
| ZNF599 | Zinc finger protein 599 | Q96NL3 |
| ZNF606 | Zinc finger protein 606 | Q8WXB4 |
| ZNF607 | Zinc finger protein 607 | Q96SK3 |
| ZNF613 | Zinc finger protein 613 | Q6PF04 |
| ZNF614 | Zinc finger protein 614 | Q8N883 |
| ZNF615 | Zinc finger protein 615 | Q8N8J6 |
| ZNF620 | Zinc finger protein 620 | Q6ZNG0 |
| ZNF621 | Zinc finger protein 621 | Q6ZSS3 |
| ZNF622 | Zinc finger protein 622 | Q969S3 |
| ZNF624 | Zinc finger protein 624 | Q9P2J8 |
| ZNF626 | Zinc finger protein 626 | Q68DY1 |
| ZNF627 | Zinc finger protein 627 | Q7L945 |
| ZNF630 | Zinc finger protein 630 | Q2M218 |
| ZNF638 | Zinc finger protein 638 | Q14966 |
| ZNF639 | Zinc finger protein 639 | Q9UID6 |
| ZNF641 | Zinc finger protein 641 | Q96N77 |
| ZNF644 | Zinc finger protein 644 | Q9H582 |
| ZNF646 | Zinc finger protein 646 | O15015 |
| ZNF649 | Zinc finger protein 649 | Q9BS31 |
| ZNF652 | Zinc finger protein 652 | Q9Y2D9 |
| ZNF654 | Zinc finger protein 654 | Q8IZM8 |
| ZNF655 | Zinc finger protein 655 | Q8N720 |
| ZNF658 | Zinc finger protein 658 | Q5TYW1 |
| ZNF662 | Zinc finger protein 662 | Q6ZS27 |
| ZNF667 | Zinc finger protein 667 | Q5HYK9 |
| ZNF668 | Zinc finger protein 668 | Q96K58 |
| ZNF669 | Zinc finger protein 669 | Q96BR6 |
| ZNF670 | Zinc finger protein 670 | Q9BS34 |
| ZNF671 | Zinc finger protein 671 | Q8TAW3 |
| ZNF672 | Zinc finger protein 672 | Q499Z4 |
| ZNF675 | Zinc finger protein 675 | Q8TD23 |
| ZNF677 | Zinc finger protein 677 | Q86XU0 |
| ZNF680 | Zinc finger protein 680 | Q8NEM1 |
| ZNF681 | Zinc finger protein 681 | Q96N22 |
| ZNF682 | Zinc finger protein 682 | O95780 |
| ZNF683 | Zinc finger protein 683 | Q8IZ20 |
| ZNF684 | Zinc finger protein 684 | Q5T5D7 |
| ZNF688 | Zinc finger protein 688 | P0C7X2 |
| ZNF689 | Zinc finger protein 689 | Q96CS4 |
| ZNF69 | Zinc finger protein 69 | Q9UC07 |
| ZNF691 | Zinc finger protein 691 | Q5VV52 |
| ZNF692 | Zinc finger protein 692 | Q9BU19 |
| ZNF696 | Zinc finger protein 696 | Q9H7X3 |
| ZNF7 | Zinc finger protein 7 | P17097 |
| ZNF701 | Zinc finger protein 701 | Q9NV72 |
| ZNF706 | Zinc finger protein 706 | Q9Y5V0 |
| ZNF708 | Zinc finger protein 708 | P17019 |
| ZNF709 | Zinc finger protein 709 | Q8N972 |
| ZNF71 | Zinc finger protein 71 | Q9NQZ8 |
| ZNF714 | Zinc finger protein 714 |  |
| ZNF718 | Zinc finger protein 718 | Q3SXZ3 |
| ZNF726 | Zinc finger protein 726 | A6NNF4 |
| ZNF738 | Zinc finger protein 738 | Q8NE65 |
| ZNF74 | Zinc finger protein 74 | Q16587 |
| ZNF746 | Zinc finger protein 746 | Q6NUN9 |
| ZNF747 | Zinc finger protein 747 | Q9BV97 |
| ZNF75A | Zinc finger protein 75a | Q96N20 |
| ZNF75D | Zinc finger protein 75D | P51815 |
| ZNF76 | Zinc finger protein 76 | P36508 |
| ZNF761 | Zinc finger protein 761 | Q86XN6 |
| ZNF763 | Zinc finger protein 763 | Q0D2J5 |
| ZNF764 | Zinc finger protein 764 | Q96H86 |
| ZNF765 | Zinc finger protein 765 | Q7L2R6 |
| ZNF766 | Zinc finger protein 766 | Q5HY98 |
| ZNF77 | Zinc finger protein 77 | Q15935 |
| ZNF770 | Zinc finger protein 770 | Q6IQ21 |
| ZNF771 | Zinc finger protein 771 | Q7L3S4 |
| ZNF772 | Zinc finger protein 772 | Q68DY9 |
| ZNF773 | Zinc finger protein 773 | Q6PK81 |
| ZNF776 | Zinc finger protein 776 | Q68DI1 |
| ZNF778 | Zinc finger protein 778 | Q96MU6 |
| ZNF780A | Zinc finger protein 780A | O75290 |
| ZNF785 | Zinc finger protein 785 | A8K8V0 |
| ZNF786 | Zinc finger protein 786 | Q8N393 |
| ZNF789 | Zinc finger protein 789 | Q5FWF6 |
| ZNF79 | Zinc finger protein 79 | Q15937 |
| ZNF790 | Zinc finger protein 790 | Q6PG37 |
| ZNF791 | Zinc finger protein 791 | Q3KP31 |
| ZNF792 | Zinc finger protein 792 | Q3KQV3 |
| ZNF793 | Zinc finger protein 793 | Q6ZN11 |
| ZNF800 | Zinc finger protein 800 | Q2TB10 |
| ZNF808 | Zinc finger protein 808 | Q8N4W9 |
| ZNF814 | Zinc finger protein 814 | B7Z6K7 |
| ZNF816 | Zinc finger protein 816 | Q0VGE8 |
| ZNF821 | Zinc finger protein 821 | O75541 |
| ZNF823 | Zinc finger protein 823 | P16415 |
| ZNF827 | Zinc finger protein 827 | Q17R98 |
| ZNF829 | Zinc finger protein 829 | Q3KNS6 |
| ZNF83 | Zinc finger protein 83 | P51522 |
| ZNF830 | Zinc finger protein 830 | Q96NB3 |
| ZNF835 | Zinc finger protein 835 | Q9Y2P0 |
| ZNF84 | Zinc finger protein 84 | P51523 |
| ZNF846 | Zinc finger protein 846 | Q147U1 |
| ZNF85 | Zinc finger protein 85 | Q03923 |
| ZNF880 | Zinc finger protein 880 | Q6PDB4 |
| ZNF92 | Zinc finger protein 92 | Q03936 |
| ZNF93 | Zinc finger protein 93 | P35789 |
| ZNFX1 | Zinc finger NFX1-type containing 1 | Q9P2E3 |
| ZNHIT1 | Zinc finger HIT-type containing 1 | O43257 |
| ZNHIT2 | Zinc finger HIT-type containing 2 | Q9UHR6 |
| ZNHIT3 | Zinc finger HIT-type containing 3 | Q15649 |
| ZNHIT6 | Zinc finger HIT-type containing 6 | Q9NWK9 |
| ZP3 | Zona pellucida glycoprotein 3 | P21754 |
| ZPR1 | ZPR1 zinc finger | O75312 |
| ZRANB1 | Zinc finger RANBP2-type containing 1 | Q9UGI0 |
| ZRANB2 | Zinc finger RANBP2-type containing 2 | O95218 |
| ZRSR2 | Zinc finger CCCH-type, RNA binding motif and serine/arginine rich 2 | Q15696 |
| ZSCAN12 | Zinc finger and SCAN domain containing 12 | O43309 |
| ZSCAN16 | Zinc finger and SCAN domain containing 16 | Q9H4T2 |
| ZSCAN2 | Zinc finger and SCAN domain containing 2 | Q7Z7L9 |
| ZSCAN21 | Zinc finger and SCAN domain containing 21 | Q9Y5A6 |
| ZSCAN22 | Zinc finger and SCAN domain containing 22 | P10073 |
| ZSCAN25 | Zinc finger and SCAN domain containing 25 | Q6NSZ9 |
| ZSCAN26 | Zinc finger and SCAN domain containing 26 | Q16670 |
| ZSCAN30 | Zinc finger and SCAN domain containing 30 | Q86W11 |
| ZSCAN31 | Zinc finger and SCAN domain containing 31 | Q96LW9 |
| ZSCAN32 | Zinc finger and SCAN domain containing 32 | Q9NX65 |
| ZSCAN5A | Zinc finger and SCAN domain containing 5A | Q9BUG6 |
| ZSCAN9 | Zinc finger and SCAN domain containing 9 | O15535 |
| ZSWIM1 | Zinc finger SWIM-type containing 1 | Q9BR11 |
| ZSWIM7 | Zinc finger SWIM-type containing 7 | Q19AV6 |
| ZW10 | Zw10 kinetochore protein | O43264 |
| ZWILCH | Zwilch kinetochore protein | Q9H900 |
| ZWINT | ZW10 interacting kinetochore protein | O95229 |
| ZYX | Zyxin | Q15942 |
| ZZZ3 | Zinc finger ZZ-type containing 3 | Q8IYH5 |

| **CD8+ T cells evaluated proteins** | | | | |  |
| --- | --- | --- | --- | --- | --- |
| **Acronym** | **Protein name** | | | **UniprotID** |  |
| AAAS | Aladin WD repeat nucleoporin | | | Q9NRG9 |  |
| AACS | Acetoacetyl-CoA synthetase | | | Q86V21 |  |
| AAGAB | Alpha and gamma adaptin binding protein | | | Q6PD74 |  |
| AAK1 | AP2 associated kinase 1 | | | Q2M2I8 |  |
| AAMDC | Adipogenesis associated Mth938 domain containing | | | Q9H7C9 |  |
| AAMP | Angio associated migratory cell protein | | | Q13685 |  |
| AAR2 | AAR2 splicing factor | | | Q9Y312 |  |
| AARSD1 | Alanyl-tRNA synthetase domain containing 1 | | | Q9BTE6 |  |
| AASDHPPT | Aminoadipate-semialdehyde dehydrogenase-phosphopantetheinyl transferase | | | Q9NRN7 |  |
| AATF | Apoptosis antagonizing transcription factor | | | Q9NY61 |  |
| ABAT | 4-aminobutyrate aminotransferase | | | P80404 |  |
| ABCB6 | ATP binding cassette subfamily B member 6 (Langereis blood group) | | | Q9NP58 |  |
| ABCB7 | ATP binding cassette subfamily B member 7 | | | O75027 |  |
| ABCC10 | ATP binding cassette subfamily C member 10 | | | Q5T3U5 |  |
| ABCC4 | ATP binding cassette subfamily C member 4 | | | O15439 |  |
| ABCC5 | ATP binding cassette subfamily C member 5 | | | O15440 |  |
| ABCD1 | ATP binding cassette subfamily D member 1 | | | P33897 |  |
| ABCD3 | ATP binding cassette subfamily D member 3 | | | P28288 |  |
| ABCD4 | ATP binding cassette subfamily D member 4 | | | O14678 |  |
| ABCE1 | ATP binding cassette subfamily E member 1 | | | P61221 |  |
| ABCF1 | ATP binding cassette subfamily F member 1 | | | Q8NE71 |  |
| ABCF2 | ATP binding cassette subfamily F member 2 | | | Q9UG63 |  |
| ABCF3 | ATP binding cassette subfamily F member 3 | | | Q9NUQ8 |  |
| ABCG1 | ATP binding cassette subfamily G member 1 | | | P45844 |  |
| ABHD10 | Abhydrolase domain containing 10, depalmitoylase | | | Q9NUJ1 |  |
| ABHD11 | Abhydrolase domain containing 11 | | | Q8NFV4 |  |
| ABHD12 | Abhydrolase domain containing 12, lysophospholipase | | | Q8N2K0 |  |
| ABHD13 | Abhydrolase domain containing 13 | | | Q7L211 |  |
| ABHD14A | Abhydrolase domain containing 14A | | | Q9BUJ0 |  |
| ABHD14B | Abhydrolase domain containing 14B | | | Q96IU4 |  |
| ABHD15 | Abhydrolase domain containing 15 | | | Q6UXT9 |  |
| ABHD16A | Abhydrolase domain containing 16A, phospholipase | | | O95870 |  |
| ABHD17A | Abhydrolase domain containing 17A, depalmitoylase | | | Q96GS6 |  |
| ABHD17B | Abhydrolase domain containing 17B, depalmitoylase | | | Q5VST6 |  |
| ABHD18 | Abhydrolase domain containing 18 | | | Q0P651 |  |
| ABHD2 | Abhydrolase domain containing 2, acylglycerol lipase | | | P08910 |  |
| ABHD3 | Abhydrolase domain containing 3, phospholipase | | | Q8WU67 |  |
| ABHD4 | Abhydrolase domain containing 4, N-acyl phospholipase B | | | Q8TB40 |  |
| ABHD5 | Abhydrolase domain containing 5, lysophosphatidic acid acyltransferase | | | Q8WTS1 |  |
| ABHD8 | Abhydrolase domain containing 8 | | | Q96I13 |  |
| ABI1 | Abl interactor 1 | | | Q8IZP0 |  |
| ABI2 | Abl interactor 2 | | | Q9NYB9 |  |
| ABI3 | ABI family member 3 | | | Q9P2A4 |  |
| ABLIM1 | Actin binding LIM protein 1 | | | O14639 |  |
| ABLIM1 | Actin binding LIM protein 1 | | | O14639 |  |
| ABRACL | ABRA C-terminal like | | | Q9P1F3 |  |
| ABT1 | Activator of basal transcription 1 | | | Q9ULW3 |  |
| ABTB1 | Ankyrin repeat and BTB domain containing 1 | | | Q969K4 |  |
| ACAA1 | Acetyl-CoA acyltransferase 1 | | | P09110 |  |
| ACAA2 | Acetyl-CoA acyltransferase 2 | | | P42765 |  |
| ACACA | Acetyl-CoA carboxylase alpha | | | Q13085 |  |
| ACACB | Acetyl-CoA carboxylase beta | | | O00763 |  |
| ACAD10 | Acyl-CoA dehydrogenase family member 10 | | | Q6JQN1 |  |
| ACAD11 | Acyl-CoA dehydrogenase family member 11 | | | Q709F0 |  |
| ACAD8 | Acyl-CoA dehydrogenase family member 8 | | | Q9UKU7 |  |
| ACAD9 | Acyl-CoA dehydrogenase family member 9 | | | Q9H845 |  |
| ACADM | Acyl-CoA dehydrogenase medium chain | | | P11310 |  |
| ACADS | Acyl-CoA dehydrogenase short chain | | | P16219 |  |
| ACADSB | Acyl-CoA dehydrogenase short/branched chain | | | P45954 |  |
| ACADVL | Acyl-CoA dehydrogenase very long chain | | | P49748 |  |
| ACAP1 | ArfGAP with coiled-coil, ankyrin repeat and PH domains 1 | | | Q15027 |  |
| ACAP2 | ArfGAP with coiled-coil, ankyrin repeat and PH domains 2 | | | Q15057 |  |
| ACAT1 | Acetyl-CoA acetyltransferase 1 | | | P24752 |  |
| ACAT2 | Acetyl-CoA acetyltransferase 2 | | | Q9BWD1 |  |
| ACBD3 | Acyl-CoA binding domain containing 3 | | | Q9H3P7 |  |
| ACBD4 | Acyl-CoA binding domain containing 4 | | | Q8NC06 |  |
| ACBD5 | Acyl-CoA binding domain containing 5 | | | Q5T8D3 |  |
| ACBD6 | Acyl-CoA binding domain containing 6 | | | Q9BR61 |  |
| ACCS | 1-aminocyclopropane-1-carboxylate synthase homolog (inactive) | | | Q96QU6 |  |
| ACD | ACD shelterin complex subunit and telomerase recruitment factor | | | Q96AP0 |  |
| ACER1 | Alkaline ceramidase 1 | | | Q8TDN7 |  |
| ACKR3 | Atypical chemokine receptor 3 | | | P25106 |  |
| ACO1 | Aconitase 1 | | | P21399 |  |
| ACO2 | Aconitase 2 | | | Q99798 |  |
| ACOT1 | Acyl-CoA thioesterase 1 | | | Q86TX2 |  |
| ACOT13 | Acyl-CoA thioesterase 13 | | | Q9NPJ3 |  |
| ACOT2 | Acyl-CoA thioesterase 2 | | | P49753 |  |
| ACOT4 | Acyl-CoA thioesterase 4 | | | Q8N9L9 |  |
| ACOT7 | Acyl-CoA thioesterase 7 | | | O00154 |  |
| ACOT8 | Acyl-CoA thioesterase 8 | | | O14734 |  |
| ACOT9 | Acyl-CoA thioesterase 9 | | | Q9Y305 |  |
| ACOX1 | Acyl-CoA oxidase 1 | | | Q15067 |  |
| ACOX3 | Acyl-CoA oxidase 3, pristanoyl | | | O15254 |  |
| ACP1 | Acid phosphatase 1 | | | P24666 |  |
| ACP2 | Acid phosphatase 2, lysosomal | | | P11117 |  |
| ACP5 | Acid phosphatase 5, tartrate resistant | | | P13686 |  |
| ACP6 | Acid phosphatase 6, lysophosphatidic | | | Q9NPH0 |  |
| ACSF2 | Acyl-CoA synthetase family member 2 | | | Q96CM8 |  |
| ACSF3 | Acyl-CoA synthetase family member 3 | | | Q4G176 |  |
| ACSL3 | Acyl-CoA synthetase long chain family member 3 | | | O95573 |  |
| ACSL4 | Acyl-CoA synthetase long chain family member 4 | | | O60488 |  |
| ACSL5 | Acyl-CoA synthetase long chain family member 5 | | | Q9ULC5 |  |
| ACSL6 | Acyl-CoA synthetase long chain family member 6 | | | Q9UKU0 |  |
| ACSM3 | Acyl-CoA synthetase medium chain family member 3 | | | Q53FZ2 |  |
| ACSS1 | Acyl-CoA synthetase short chain family member 1 | | | Q9NUB1 |  |
| ACSS2 | Acyl-CoA synthetase short chain family member 2 | | | Q9NR19 |  |
| ACTA1 | Actin alpha 1, skeletal muscle | | | P68133 |  |
| ACTA2 | Actin alpha 2, smooth muscle | | | P62736 |  |
| ACTB | Actin beta | | | P60709 |  |
| ACTG1 | Actin gamma 1 | | | P63261 |  |
| ACTL6A | Actin like 6A | | | O96019 |  |
| ACTN1 | Actinin alpha 1 | | | P12814 |  |
| ACTN4 | Actinin alpha 4 | | | O43707 |  |
| ACTR10 | Actin related protein 10 | | | Q9NZ32 |  |
| ACTR1A | Actin related protein 1A | | | P61163 |  |
| ACTR1B | Actin related protein 1B | | | P42025 |  |
| ACTR2 | Actin related protein 2 | | | P61160 |  |
| ACTR3 | Actin related protein 3 | | | P61158 |  |
| ACTR3B | Actin related protein 3B | | | Q9P1U1 |  |
| ACTR6 | Actin related protein 6 | | | Q9GZN1 |  |
| ACTR8 | Actin related protein 8 | | | Q9H981 |  |
| ACVR1 | Activin A receptor type 1 | | | Q04771 |  |
| ACVR2A | Activin A receptor type 2A | | | P27037 |  |
| ACY1 | Aminoacylase 1 | | | Q03154 |  |
| ACY3 | Aminoacylase 3 | | | Q96HD9 |  |
| ACYP1 | Acylphosphatase 1 | | | P07311 |  |
| ACYP2 | Acylphosphatase 2 | | | P14621 |  |
| ADA | Adenosine deaminase | | | P00813 |  |
| ADAL | Adenosine deaminase like | | | Q6DHV7 |  |
| ADAM15 | ADAM metallopeptidase domain 15 | | | Q13444 |  |
| ADAM19 | ADAM metallopeptidase domain 19 | | | Q9H013 |  |
| ADAM23 | ADAM metallopeptidase domain 23 | | | O75077 |  |
| ADAP1 | ArfGAP with dual PH domains 1 | | | O75689 |  |
| ADAR | Adenosine deaminase RNA specific | | | P55265 |  |
| ADAT1 | Adenosine deaminase tRNA specific 1 | | | Q9BUB4 |  |
| ADAT3 | Adenosine deaminase tRNA specific 3 | | |  |  |
| ADCK1 | AarF domain containing kinase 1 | | | Q86TW2 |  |
| ADCK2 | AarF domain containing kinase 2 | | | Q7Z695 |  |
| ADCK5 | AarF domain containing kinase 5 | | | Q3MIX3 |  |
| ADD1 | Adducin 1 | | | P35611 |  |
| ADGRE1 | Adhesion G protein-coupled receptor E1 | | | Q14246 |  |
| ADGRG5 | Adhesion G protein-coupled receptor G5 | | | Q8IZF4 |  |
| ADH5 | Alcohol dehydrogenase 5 (class III), chi polypeptide | | | P11766 |  |
| ADI1 | Acireductone dioxygenase 1 | | | Q9BV57 |  |
| ADIPOR1 | Adiponectin receptor 1 | | | Q96A54 |  |
| ADIRF | Adipogenesis regulatory factor | | | Q15847 |  |
| ADIRF | Adipogenesis regulatory factor | | | Q15847 |  |
| ADK | Adenosine kinase | | | P55263 |  |
| ADNP | Activity dependent neuroprotector homeobox | | | Q9H2P0 |  |
| ADNP2 | ADNP homeobox 2 | | | Q6IQ32 |  |
| ADORA2A | Adenosine A2a receptor | | | P29274 |  |
| ADPGK | ADP dependent glucokinase | | | Q9BRR6 |  |
| ADPRH | ADP-ribosylarginine hydrolase | | | P54922 |  |
| ADPRM | ADP-ribose/CDP-alcohol diphosphatase, manganese dependent | | | Q3LIE5 |  |
| ADRB2 | Adrenoceptor beta 2 | | | P07550 |  |
| ADRM1 | ADRM1 26S proteasome ubiquitin receptor | | | Q16186 |  |
| ADSL | Adenylosuccinate lyase | | | P30566 |  |
| ADTRP | Androgen dependent TFPI regulating protein | | | Q96IZ2 |  |
| ADTRP | Androgen dependent TFPI regulating protein | | | Q96IZ2 |  |
| AEBP1 | AE binding protein 1 | | | Q8IUX7 |  |
| AEBP2 | AE binding protein 2 | | | Q6ZN18 |  |
| AEN | Apoptosis enhancing nuclease | | | Q8WTP8 |  |
| AFAP1 | Actin filament associated protein 1 | | | Q8N556 |  |
| AFAP1L2 | Actin filament associated protein 1 like 2 | | | Q8N4X5 |  |
| AFF1 | ALF transcription elongation factor 1 | | | P51825 |  |
| AFF3 | ALF transcription elongation factor 3 | | | P51826 |  |
| AFF4 | ALF transcription elongation factor 4 | | | Q9UHB7 |  |
| AFG3L2 | AFG3 like matrix AAA peptidase subunit 2 | | | Q9Y4W6 |  |
| AFMID | Arylformamidase | | | Q63HM1 |  |
| AFTPH | Aftiphilin | | | Q6ULP2 |  |
| AGA | Aspartylglucosaminidase | | | P20933 |  |
| AGAP2 | ArfGAP with GTPase domain, ankyrin repeat and PH domain 2 | | | Q99490 |  |
| AGAP3 | ArfGAP with GTPase domain, ankyrin repeat and PH domain 3 | | | Q96P47 |  |
| AGBL5 | AGBL carboxypeptidase 5 | | | Q8NDL9 |  |
| AGFG1 | ArfGAP with FG repeats 1 | | | P52594 |  |
| AGFG2 | ArfGAP with FG repeats 2 | | | O95081 |  |
| AGGF1 | Angiogenic factor with G-patch and FHA domains 1 | | | Q8N302 |  |
| AGK | Acylglycerol kinase | | | Q53H12 |  |
| AGL | Amylo-alpha-1, 6-glucosidase, 4-alpha-glucanotransferase | | | P35573 |  |
| AGMAT | Agmatinase | | | Q9BSE5 |  |
| AGPAT1 | 1-acylglycerol-3-phosphate O-acyltransferase 1 | | | Q99943 |  |
| AGPAT2 | 1-acylglycerol-3-phosphate O-acyltransferase 2 | | | O15120 |  |
| AGPAT3 | 1-acylglycerol-3-phosphate O-acyltransferase 3 | | | Q9NRZ7 |  |
| AGPAT4 | 1-acylglycerol-3-phosphate O-acyltransferase 4 | | | Q9NRZ5 |  |
| AGPAT5 | 1-acylglycerol-3-phosphate O-acyltransferase 5 | | | Q9NUQ2 |  |
| AGTPBP1 | ATP/GTP binding carboxypeptidase 1 | | | Q9UPW5 |  |
| AGTRAP | Angiotensin II receptor associated protein | | | Q6RW13 |  |
| AHCY | Adenosylhomocysteinase | | | P23526 |  |
| AHCYL1 | Adenosylhomocysteinase like 1 | | | O43865 |  |
| AHI1 | Abelson helper integration site 1 | | | Q8N157 |  |
| AHNAK | AHNAK nucleoprotein | | | Q09666 |  |
| AHR | Aryl hydrocarbon receptor | | | P35869 |  |
| AHSA1 | Activator of HSP90 ATPase activity 1 | | | O95433 |  |
| AIDA | Axin interactor, dorsalization associated | | | Q96BJ3 |  |
| AIF1 | Allograft inflammatory factor 1 | | | P55008 |  |
| AIFM1 | Apoptosis inducing factor mitochondria associated 1 | | | O95831 |  |
| AIG1 | Androgen induced 1 | | | Q9NVV5 |  |
| AIM2 | Absent in melanoma 2 | | | O14862 |  |
| AIMP1 | Aminoacyl tRNA synthetase complex interacting multifunctional protein 1 | | | Q12904 |  |
| AIMP2 | Aminoacyl tRNA synthetase complex interacting multifunctional protein 2 | | | Q13155 |  |
| AK2 | Adenylate kinase 2 | | | P54819 |  |
| AK3 | Adenylate kinase 3 | | | Q9UIJ7 |  |
| AK5 | Adenylate kinase 5 | | | Q9Y6K8 |  |
| AK5 | Adenylate kinase 5 | | | Q9Y6K8 |  |
| AK6 | Adenylate kinase 6 | | | Q9Y3D8 |  |
| AK9 | Adenylate kinase 9 | | | Q5TCS8 |  |
| AKAP1 | A-kinase anchoring protein 1 | | | Q92667 |  |
| AKAP10 | A-kinase anchoring protein 10 | | | O43572 |  |
| AKAP13 | A-kinase anchoring protein 13 | | | Q12802 |  |
| AKAP7 | A-kinase anchoring protein 7 | | | O43687, Q9P0M2 |  |
| AKAP8 | A-kinase anchoring protein 8 | | | O43823 |  |
| AKAP8L | A-kinase anchoring protein 8 like | | | Q9ULX6 |  |
| AKIP1 | A-kinase interacting protein 1 | | | Q9NQ31 |  |
| AKIRIN2 | Akirin 2 | | | Q53H80 |  |
| AKNA | AT-hook transcription factor | | | Q7Z591 |  |
| AKR1A1 | Aldo-keto reductase family 1 member A1 | | | P14550 |  |
| AKR1B1 | Aldo-keto reductase family 1 member B | | | P15121 |  |
| AKR1C3 | Aldo-keto reductase family 1 member C3 | | | P42330 |  |
| AKR7A2 | Aldo-keto reductase family 7 member A2 | | | O43488 |  |
| AKT1 | AKT serine/threonine kinase 1 | | | P31749 |  |
| AKT2 | AKT serine/threonine kinase 2 | | | P31751 |  |
| AKTIP | AKT interacting protein | | | Q9H8T0 |  |
| ALAD | Aminolevulinate dehydratase | | | P13716 |  |
| ALAS1 | 5'-aminolevulinate synthase 1 | | | P13196 |  |
| ALCAM | Activated leukocyte cell adhesion molecule | | | Q13740 |  |
| ALDH16A1 | Aldehyde dehydrogenase 16 family member A1 | | | Q8IZ83 |  |
| ALDH18A1 | Aldehyde dehydrogenase 18 family member A1 | | | P54886 |  |
| ALDH1B1 | Aldehyde dehydrogenase 1 family member B1 | | | P30837 |  |
| ALDH3A2 | Aldehyde dehydrogenase 3 family member A2 | | | P51648 |  |
| ALDH5A1 | Aldehyde dehydrogenase 5 family member A1 | | | P51649 |  |
| ALDH6A1 | Aldehyde dehydrogenase 6 family member A1 | | | Q02252 |  |
| ALDH7A1 | Aldehyde dehydrogenase 7 family member A1 | | | P49419 |  |
| ALDH9A1 | Aldehyde dehydrogenase 9 family member A1 | | | P49189 |  |
| ALDOA | Aldolase, fructose-bisphosphate A | | | P04075 |  |
| ALDOC | Aldolase, fructose-bisphosphate C | | | P09972 |  |
| ALG1 | ALG1 chitobiosyldiphosphodolichol beta-mannosyltransferase | | | Q9BT22 |  |
| ALG10 | ALG10 alpha-1,2-glucosyltransferase | | | Q5BKT4 |  |
| ALG12 | ALG12 alpha-1,6-mannosyltransferase | | | Q9BV10 |  |
| ALG13 | ALG13 UDP-N-acetylglucosaminyltransferase subunit | | | Q9NP73 |  |
| ALG2 | ALG2 alpha-1,3/1,6-mannosyltransferase | | | Q9H553 |  |
| ALG3 | ALG3 alpha-1,3- mannosyltransferase | | | Q92685 |  |
| ALG5 | ALG5 dolichyl-phosphate beta-glucosyltransferase | | | Q9Y673 |  |
| ALG8 | ALG8 alpha-1,3-glucosyltransferase | | | Q9BVK2 |  |
| ALG9 | ALG9 alpha-1,2-mannosyltransferase | | | Q9H6U8 |  |
| ALKBH1 | AlkB homolog 1, histone H2A dioxygenase | | | Q13686 |  |
| ALKBH2 | AlkB homolog 2, alpha-ketoglutarate dependent dioxygenase | | | Q6NS38 |  |
| ALKBH3 | AlkB homolog 3, alpha-ketoglutarate dependent dioxygenase | | | Q96Q83 |  |
| ALKBH4 | AlkB homolog 4, lysine demethylase | | | Q9NXW9 |  |
| ALKBH5 | AlkB homolog 5, RNA demethylase | | | Q6P6C2 |  |
| ALKBH7 | AlkB homolog 7 | | | Q9BT30 |  |
| ALKBH8 | AlkB homolog 8, tRNA methyltransferase | | | Q96BT7 |  |
| ALOX5 | Arachidonate 5-lipoxygenase | | | P09917 |  |
| ALOX5AP | Arachidonate 5-lipoxygenase activating protein | | | P20292 |  |
| ALS2 | Alsin Rho guanine nucleotide exchange factor ALS2 | | | Q96Q42 |  |
| ALYREF | Aly/REF export factor | | |  |  |
| AMACR | Alpha-methylacyl-CoA racemase | | | Q9UHK6 |  |
| AMBRA1 | Autophagy and beclin 1 regulator 1 | | | Q9C0C7 |  |
| AMD1 | Adenosylmethionine decarboxylase 1 | | | P17707 |  |
| AMDHD2 | Amidohydrolase domain containing 2 | | | Q9Y303 |  |
| AMIGO1 | Adhesion molecule with Ig like domain 1 | | | Q86WK6 |  |
| AMIGO2 | Adhesion molecule with Ig like domain 2 | | | Q86SJ2 |  |
| AMMECR1 | AMMECR nuclear protein 1 | | | Q9Y4X0 |  |
| AMMECR1L | AMMECR1 like | | | Q6DCA0 |  |
| AMN1 | Antagonist of mitotic exit network 1 homolog | | | Q8IY45 |  |
| AMPD2 | Adenosine monophosphate deaminase 2 | | | Q01433 |  |
| AMPD3 | Adenosine monophosphate deaminase 3 | | | Q01432 |  |
| AMZ2 | Archaelysin family metallopeptidase 2 | | | Q86W34 |  |
| ANAPC10 | Anaphase promoting complex subunit 10 | | | Q9UM13 |  |
| ANAPC11 | Anaphase promoting complex subunit 11 | | | Q9NYG5 |  |
| ANAPC13 | Anaphase promoting complex subunit 13 | | | Q9BS18 |  |
| ANAPC15 | Anaphase promoting complex subunit 15 | | | P60006 |  |
| ANAPC16 | Anaphase promoting complex subunit 16 | | | Q96DE5 |  |
| ANAPC5 | Anaphase promoting complex subunit 5 | | | Q9UJX4 |  |
| ANG | Angiogenin | | | P03950 |  |
| ANGEL1 | Angel homolog 1 | | | Q9UNK9 |  |
| ANGEL2 | Angel homolog 2 | | | Q5VTE6 |  |
| ANK3 | Ankyrin 3 | | | Q12955 |  |
| ANKEF1 | Ankyrin repeat and EF-hand domain containing 1 | | | Q9NU02 |  |
| ANKFY1 | Ankyrin repeat and FYVE domain containing 1 | | | Q9P2R3 |  |
| ANKH | ANKH inorganic pyrophosphate transport regulator | | | Q9HCJ1 |  |
| ANKHD1 | Ankyrin repeat and KH domain containing 1 | | | Q8IWZ3 |  |
| ANKLE2 | Ankyrin repeat and LEM domain containing 2 | | | Q86XL3 |  |
| ANKMY1 | Ankyrin repeat and MYND domain containing 1 | | | Q9P2S6 |  |
| ANKMY2 | Ankyrin repeat and MYND domain containing 2 | | | Q8IV38 |  |
| ANKRA2 | Ankyrin repeat family A member 2 | | | Q9H9E1 |  |
| ANKRD10 | Ankyrin repeat domain 10 | | | Q9NXR5 |  |
| ANKRD12 | Ankyrin repeat domain 12 | | | Q6UB98 |  |
| ANKRD13A | Ankyrin repeat domain 13A | | | Q8IZ07 |  |
| ANKRD13C | Ankyrin repeat domain 13C | | | Q8N6S4 |  |
| ANKRD13D | Ankyrin repeat domain 13D | | | Q6ZTN6 |  |
| ANKRD17 | Ankyrin repeat domain 17 | | | O75179 |  |
| ANKRD27 | Ankyrin repeat domain 27 | | | Q96NW4 |  |
| ANKRD36 | Ankyrin repeat domain 36 | | | A6QL64 |  |
| ANKRD36B | Ankyrin repeat domain 36B | | | Q8N2N9 |  |
| ANKRD37 | Ankyrin repeat domain 37 | | | Q7Z713 |  |
| ANKRD39 | Ankyrin repeat domain 39 | | | Q53RE8 |  |
| ANKRD40 | Ankyrin repeat domain 40 | | | Q6AI12 |  |
| ANKRD42 | Ankyrin repeat domain 42 | | | Q8N9B4 |  |
| ANKRD44 | Ankyrin repeat domain 44 | | | Q8N8A2 |  |
| ANKRD46 | Ankyrin repeat domain 46 | | | Q86W74 |  |
| ANKRD49 | Ankyrin repeat domain 49 | | | Q8WVL7 |  |
| ANKRD54 | Ankyrin repeat domain 54 | | | Q6NXT1 |  |
| ANKRD55 | Ankyrin repeat domain 55 | | | Q3KP44 |  |
| ANKRD55 | Ankyrin repeat domain 55 | | | Q3KP44 |  |
| ANKS1B | Ankyrin repeat and sterile alpha motif domain containing 1B | | | Q7Z6G8 |  |
| ANKS3 | Ankyrin repeat and sterile alpha motif domain containing 3 | | | Q6ZW76 |  |
| ANKZF1 | Ankyrin repeat and zinc finger peptidyl tRNA hydrolase 1 | | | Q9H8Y5 |  |
| ANO10 | Anoctamin 10 | | | Q9NW15 |  |
| ANO6 | Anoctamin 6 | | | Q4KMQ2 |  |
| ANP32A | Acidic nuclear phosphoprotein 32 family member A | | | P39687 |  |
| ANP32B | Acidic nuclear phosphoprotein 32 family member B | | | Q92688 |  |
| ANP32E | Acidic nuclear phosphoprotein 32 family member E | | | Q9BTT0 |  |
| ANTXR2 | ANTXR cell adhesion molecule 2 | | | P58335 |  |
| ANXA1 | Annexin A1 | | | P04083 |  |
| ANXA11 | Annexin A11 | | | P50995 |  |
| ANXA2 | Annexin A2 | | | P07355 |  |
| ANXA2R | Annexin A2 receptor | | | Q3ZCQ2 |  |
| ANXA4 | Annexin A4 | | | P09525 |  |
| ANXA5 | Annexin A5 | | | P08758 |  |
| ANXA6 | Annexin A6 | | | P08133 |  |
| ANXA7 | Annexin A7 | | | P20073 |  |
| AOAH | Acyloxyacyl hydrolase | | | P28039 |  |
| AOPEP | Aminopeptidase O (putative) | | | Q8N6M6 |  |
| AP1AR | Adaptor related protein complex 1 associated regulatory protein | | | Q63HQ0 |  |
| AP1B1 | Adaptor related protein complex 1 subunit beta 1 | | | Q10567 |  |
| AP1G1 | Adaptor related protein complex 1 subunit gamma 1 | | | O43747 |  |
| AP1S1 | Adaptor related protein complex 1 subunit sigma 1 | | | P61966 |  |
| AP1S2 | Adaptor related protein complex 1 subunit sigma 2 | | | P56377 |  |
| AP2A1 | Adaptor related protein complex 2 subunit alpha 1 | | | O95782 |  |
| AP2B1 | Adaptor related protein complex 2 subunit beta 1 | | | P63010 |  |
| AP2M1 | Adaptor related protein complex 2 subunit mu 1 | | | Q96CW1 |  |
| AP2S1 | Adaptor related protein complex 2 subunit sigma 1 | | | P53680 |  |
| AP3B1 | Adaptor related protein complex 3 subunit beta 1 | | | O00203 |  |
| AP3D1 | Adaptor related protein complex 3 subunit delta 1 | | | O14617 |  |
| AP3M1 | Adaptor related protein complex 3 subunit mu 1 | | | Q9Y2T2 |  |
| AP3M2 | Adaptor related protein complex 3 subunit mu 2 | | | P53677 |  |
| AP3S1 | Adaptor related protein complex 3 subunit sigma 1 | | | Q92572 |  |
| AP3S2 | Adaptor related protein complex 3 subunit sigma 2 | | | P59780 |  |
| AP4B1 | Adaptor related protein complex 4 subunit beta 1 | | | Q9Y6B7 |  |
| AP4M1 | Adaptor related protein complex 4 subunit mu 1 | | | O00189 |  |
| AP4S1 | Adaptor related protein complex 4 subunit sigma 1 | | | Q9Y587 |  |
| AP5M1 | Adaptor related protein complex 5 subunit mu 1 | | | Q9H0R1 |  |
| AP5S1 | Adaptor related protein complex 5 subunit sigma 1 | | | Q9NUS5 |  |
| AP5Z1 | Adaptor related protein complex 5 subunit zeta 1 | | | O43299 |  |
| APAF1 | Apoptotic peptidase activating factor 1 | | | O14727 |  |
| APBA2 | Amyloid beta precursor protein binding family A member 2 | | | Q99767 |  |
| APBA2 | Amyloid beta precursor protein binding family A member 2 | | | Q99767 |  |
| APBA3 | Amyloid beta precursor protein binding family A member 3 | | | O96018 |  |
| APBB1 | Amyloid beta precursor protein binding family B member 1 | | | O00213 |  |
| APBB1 | Amyloid beta precursor protein binding family B member 1 | | | O00213 |  |
| APBB1IP | Amyloid beta precursor protein binding family B member 1 interacting protein | | | Q7Z5R6 |  |
| APC | APC regulator of WNT signaling pathway | | | P25054 |  |
| APEH | Acylaminoacyl-peptide hydrolase | | | P13798 |  |
| APEX1 | Apurinic/apyrimidinic endodeoxyribonuclease 1 | | | P27695 |  |
| APEX2 | Apurinic/apyrimidinic endodeoxyribonuclease 2 | | | Q9UBZ4 |  |
| APH1A | Aph-1 homolog A, gamma-secretase subunit | | | Q96BI3 |  |
| APH1B | Aph-1 homolog B, gamma-secretase subunit | | | Q8WW43 |  |
| API5 | Apoptosis inhibitor 5 | | | Q9BZZ5 |  |
| APIP | APAF1 interacting protein | | | Q96GX9 |  |
| APLP2 | Amyloid beta precursor like protein 2 | | | Q06481 |  |
| APMAP | Adipocyte plasma membrane associated protein | | | Q9HDC9 |  |
| APOBEC2 | Apolipoprotein B mRNA editing enzyme catalytic subunit 2 | | | Q9Y235 |  |
| APOBEC3C | Apolipoprotein B mRNA editing enzyme catalytic subunit 3C | | | Q9NRW3 |  |
| APOBEC3D | Apolipoprotein B mRNA editing enzyme catalytic subunit 3D | | | Q96AK3 |  |
| APOBEC3F | Apolipoprotein B mRNA editing enzyme catalytic subunit 3F | | | Q8IUX4 |  |
| APOBEC3G | Apolipoprotein B mRNA editing enzyme catalytic subunit 3G | | | Q9HC16 |  |
| APOBEC3H | Apolipoprotein B mRNA editing enzyme catalytic subunit 3H | | | Q6NTF7 |  |
| APOL1 | Apolipoprotein L1 | | | O14791 |  |
| APOL2 | Apolipoprotein L2 | | | Q9BQE5 |  |
| APOL3 | Apolipoprotein L3 | | | O95236 |  |
| APOL6 | Apolipoprotein L6 | | | Q9BWW8 |  |
| APOO | Apolipoprotein O | | | Q9BUR5 |  |
| APOOL | Apolipoprotein O like | | | Q6UXV4 |  |
| APP | Amyloid beta precursor protein | | | P05067 |  |
| APPBP2 | Amyloid beta precursor protein binding protein 2 | | | Q92624 |  |
| APPL1 | Adaptor protein, phosphotyrosine interacting with PH domain and leucine zipper 1 | | | Q9UKG1 |  |
| APRT | Adenine phosphoribosyltransferase | | | P07741 |  |
| APTX | Aprataxin | | | Q7Z2E3 |  |
| ARAF | A-Raf proto-oncogene, serine/threonine kinase | | | P10398 |  |
| ARAP1 | ArfGAP with RhoGAP domain, ankyrin repeat and PH domain 1 | | | Q96P48 |  |
| ARAP2 | ArfGAP with RhoGAP domain, ankyrin repeat and PH domain 2 | | | Q8WZ64 |  |
| ARCN1 | Archain 1 | | | P48444 |  |
| AREL1 | Apoptosis resistant E3 ubiquitin protein ligase 1 | | | O15033 |  |
| ARF1 | ADP ribosylation factor 1 | | | P84077 |  |
| ARF3 | ADP ribosylation factor 3 | | | P61204 |  |
| ARF4 | ADP ribosylation factor 4 | | | P18085 |  |
| ARF5 | ADP ribosylation factor 5 | | | P84085 |  |
| ARF6 | ADP ribosylation factor 6 | | | P62330 |  |
| ARFGAP1 | ADP ribosylation factor GTPase activating protein 1 | | | Q8N6T3 |  |
| ARFGAP2 | ADP ribosylation factor GTPase activating protein 2 | | | Q8N6H7 |  |
| ARFGAP3 | ADP ribosylation factor GTPase activating protein 3 | | | Q9NP61 |  |
| ARFGEF1 | ADP ribosylation factor guanine nucleotide exchange factor 1 | | | Q9Y6D6 |  |
| ARFIP1 | ADP ribosylation factor interacting protein 1 | | | P53367 |  |
| ARFIP2 | ADP ribosylation factor interacting protein 2 | | | P53365 |  |
| ARFRP1 | ADP ribosylation factor related protein 1 | | | Q13795 |  |
| ARG1 | Arginase 1 | | | P05089 |  |
| ARGLU1 | Arginine and glutamate rich 1 | | | Q9NWB6 |  |
| ARHGAP1 | Rho GTPase activating protein 1 | | | Q07960 |  |
| ARHGAP12 | Rho GTPase activating protein 12 | | | Q8IWW6 |  |
| ARHGAP15 | Rho GTPase activating protein 15 | | | Q53QZ3 |  |
| ARHGAP17 | Rho GTPase activating protein 17 | | | Q68EM7 |  |
| ARHGAP18 | Rho GTPase activating protein 18 | | | Q8N392 |  |
| ARHGAP19 | Rho GTPase activating protein 19 | | | Q14CB8 |  |
| ARHGAP25 | Rho GTPase activating protein 25 | | | P42331 |  |
| ARHGAP4 | Rho GTPase activating protein 4 | | | P98171 |  |
| ARHGAP9 | Rho GTPase activating protein 9 | | | Q9BRR9 |  |
| ARHGDIA | Rho GDP dissociation inhibitor alpha | | | P52565 |  |
| ARHGDIB | Rho GDP dissociation inhibitor beta | | | P52566 |  |
| ARHGEF1 | Rho guanine nucleotide exchange factor 1 | | | Q92888 |  |
| ARHGEF2 | Rho/Rac guanine nucleotide exchange factor 2 | | | Q92974 |  |
| ARHGEF3 | Rho guanine nucleotide exchange factor 3 | | | Q9NR81 |  |
| ARHGEF7 | Rho guanine nucleotide exchange factor 7 | | | Q14155 |  |
| ARHGEF9 | Cdc42 guanine nucleotide exchange factor 9 | | | O43307 |  |
| ARID2 | AT-rich interaction domain 2 | | | Q68CP9 |  |
| ARID3B | AT-rich interaction domain 3B | | | Q8IVW6 |  |
| ARID5A | AT-rich interaction domain 5A | | | Q03989 |  |
| ARID5B | AT-rich interaction domain 5B | | | Q14865 |  |
| ARIH2 | Ariadne RBR E3 ubiquitin protein ligase 2 | | | O95376 |  |
| ARL1 | ADP ribosylation factor like GTPase 1 | | | P40616 |  |
| ARL10 | ADP ribosylation factor like GTPase 10 | | | Q8N8L6 |  |
| ARL13B | ADP ribosylation factor like GTPase 13B | | | Q3SXY8 |  |
| ARL14EP | ADP ribosylation factor like GTPase 14 effector protein | | | Q8N8R7 |  |
| ARL15 | ADP ribosylation factor like GTPase 15 | | | Q9NXU5 |  |
| ARL16 | ADP ribosylation factor like GTPase 16 | | |  |  |
| ARL17A | ADP ribosylation factor like GTPase 17A | | | Q8IVW1 |  |
| ARL17B | ADP ribosylation factor like GTPase 17B | | | Q8IVW1 |  |
| ARL2 | ADP ribosylation factor like GTPase 2 | | | P36404 |  |
| ARL2BP | ADP ribosylation factor like GTPase 2 binding protein | | | Q9Y2Y0 |  |
| ARL3 | ADP ribosylation factor like GTPase 3 | | | P36405 |  |
| ARL4A | ADP ribosylation factor like GTPase 4A | | | P40617 |  |
| ARL4C | ADP ribosylation factor like GTPase 4C | | | P56559 |  |
| ARL4D | ADP ribosylation factor like GTPase 4D | | | P49703 |  |
| ARL5A | ADP ribosylation factor like GTPase 5A | | | Q9Y689 |  |
| ARL6 | ADP ribosylation factor like GTPase 6 | | | Q9H0F7 |  |
| ARL6IP1 | ADP ribosylation factor like GTPase 6 interacting protein 1 | | | Q15041 |  |
| ARL6IP4 | ADP ribosylation factor like GTPase 6 interacting protein 4 | | | Q66PJ3 |  |
| ARL6IP5 | ADP ribosylation factor like GTPase 6 interacting protein 5 | | | O75915 |  |
| ARL6IP6 | ADP ribosylation factor like GTPase 6 interacting protein 6 | | | Q8N6S5 |  |
| ARL8A | ADP ribosylation factor like GTPase 8A | | | Q96BM9 |  |
| ARL8B | ADP ribosylation factor like GTPase 8B | | | Q9NVJ2 |  |
| ARMC1 | Armadillo repeat containing 1 | | | Q9NVT9 |  |
| ARMC10 | Armadillo repeat containing 10 | | | Q8N2F6 |  |
| ARMC2 | Armadillo repeat containing 2 | | | Q8NEN0 |  |
| ARMC5 | Armadillo repeat containing 5 | | | Q96C12 |  |
| ARMC6 | Armadillo repeat containing 6 | | | Q6NXE6 |  |
| ARMC7 | Armadillo repeat containing 7 | | | Q9H6L4 |  |
| ARMC8 | Armadillo repeat containing 8 | | | Q8IUR7 |  |
| ARMCX1 | Armadillo repeat containing X-linked 1 | | | Q9P291 |  |
| ARMCX2 | Armadillo repeat containing X-linked 2 | | | Q7L311 |  |
| ARMCX3 | Armadillo repeat containing X-linked 3 | | | Q9UH62 |  |
| ARMCX5 | Armadillo repeat containing X-linked 5 | | | Q6P1M9 |  |
| ARMCX6 | Armadillo repeat containing X-linked 6 | | | Q7L4S7 |  |
| ARNT | Aryl hydrocarbon receptor nuclear translocator | | | P27540 |  |
| ARPC1A | Actin related protein 2/3 complex subunit 1A | | | Q92747 |  |
| ARPC1B | Actin related protein 2/3 complex subunit 1B | | | O15143 |  |
| ARPC3 | Actin related protein 2/3 complex subunit 3 | | | O15145 |  |
| ARPC4 | Actin related protein 2/3 complex subunit 4 | | | P59998 |  |
| ARPC5 | Actin related protein 2/3 complex subunit 5 | | | O15511 |  |
| ARPC5L | Actin related protein 2/3 complex subunit 5 like | | | Q9BPX5 |  |
| ARPP19 | CAMP regulated phosphoprotein 19 | | | P56211 |  |
| ARRB1 | Arrestin beta 1 | | | P49407 |  |
| ARRB2 | Arrestin beta 2 | | | P32121 |  |
| ARRDC1 | Arrestin domain containing 1 | | | Q8N5I2 |  |
| ARRDC2 | Arrestin domain containing 2 | | | Q8TBH0 |  |
| ARRDC3 | Arrestin domain containing 3 | | | Q96B67 |  |
| ARSA | Arylsulfatase A | | | P15289 |  |
| ARSG | Arylsulfatase G | | | Q96EG1 |  |
| ARSK | Arylsulfatase family member K | | | Q6UWY0 |  |
| ARV1 | ARV1 homolog, fatty acid homeostasis modulator | | | Q9H2C2 |  |
| ASAH1 | N-acylsphingosine amidohydrolase 1 | | | Q13510 |  |
| ASAH2B | N-acylsphingosine amidohydrolase 2B | | | P0C7U1 |  |
| ASB1 | Ankyrin repeat and SOCS box containing 1 | | | Q9Y576 |  |
| ASB13 | Ankyrin repeat and SOCS box containing 13 | | | Q8WXK3 |  |
| ASB16 | Ankyrin repeat and SOCS box containing 16 | | | Q96NS5 |  |
| ASB2 | Ankyrin repeat and SOCS box containing 2 | | | Q96Q27 |  |
| ASB3 | Ankyrin repeat and SOCS box containing 3 | | | Q9Y575 |  |
| ASB6 | Ankyrin repeat and SOCS box containing 6 | | | Q9NWX5 |  |
| ASB7 | Ankyrin repeat and SOCS box containing 7 | | | Q9H672 |  |
| ASB8 | Ankyrin repeat and SOCS box containing 8 | | | Q9H765 |  |
| ASB9 | Ankyrin repeat and SOCS box containing 9 | | | Q96DX5 |  |
| ASCC1 | Activating signal cointegrator 1 complex subunit 1 | | | Q8N9N2 |  |
| ASCC2 | Activating signal cointegrator 1 complex subunit 2 | | | Q9H1I8 |  |
| ASCC3 | Activating signal cointegrator 1 complex subunit 3 | | | Q8N3C0 |  |
| ASF1A | Anti-silencing function 1A histone chaperone | | | Q9Y294 |  |
| ASF1B | Anti-silencing function 1B histone chaperone | | | Q9NVP2 |  |
| ASH2L | ASH2 like, histone lysine methyltransferase complex subunit | | | Q9UBL3 |  |
| ASL | Argininosuccinate lyase | | | P04424 |  |
| ASMTL | Acetylserotonin O-methyltransferase like | | | O95671 |  |
| ASNS | Asparagine synthetase (glutamine-hydrolyzing) | | | P08243 |  |
| ASNSD1 | Asparagine synthetase domain containing 1 | | | Q9NWL6 |  |
| ASPH | Aspartate beta-hydroxylase | | | Q12797 |  |
| ASPSCR1 | ASPSCR1 tether for SLC2A4, UBX domain containing | | | Q9BZE9 |  |
| ASRGL1 | Asparaginase and isoaspartyl peptidase 1 | | | Q7L266 |  |
| ASTE1 | Asteroid homolog 1 | | | Q2TB18 |  |
| ASXL1 | ASXL transcriptional regulator 1 | | | Q8IXJ9 |  |
| ASXL2 | ASXL transcriptional regulator 2 | | | Q76L83 |  |
| ATAD2 | ATPase family AAA domain containing 2 | | | Q6PL18 |  |
| ATAD3A | ATPase family AAA domain containing 3A | | | Q9NVI7 |  |
| ATE1 | Arginyltransferase 1 | | | O95260 |  |
| ATF1 | Activating transcription factor 1 | | | P18846 |  |
| ATF2 | Activating transcription factor 2 | | | P15336 |  |
| ATF4 | Activating transcription factor 4 | | | P18848 |  |
| ATF5 | Activating transcription factor 5 | | | Q9Y2D1 |  |
| ATF6 | Activating transcription factor 6 | | | P18850 |  |
| ATF6B | Activating transcription factor 6 beta | | | Q99941 |  |
| ATF7 | Activating transcription factor 7 | | | P17544 |  |
| ATF7IP2 | Activating transcription factor 7 interacting protein 2 | | | Q5U623 |  |
| ATG10 | Autophagy related 10 | | | Q9H0Y0 |  |
| ATG101 | Autophagy related 101 | | | Q9BSB4 |  |
| ATG12 | Autophagy related 12 | | | O94817 |  |
| ATG13 | Autophagy related 13 | | | O75143 |  |
| ATG14 | Autophagy related 14 | | | Q6ZNE5 |  |
| ATG16L1 | Autophagy related 16 like 1 | | | Q676U5 |  |
| ATG3 | Autophagy related 3 | | | Q9NT62 |  |
| ATG4A | Autophagy related 4A cysteine peptidase | | | Q8WYN0 |  |
| ATG4B | Autophagy related 4B cysteine peptidase | | | Q9Y4P1 |  |
| ATG4C | Autophagy related 4C cysteine peptidase | | | Q96DT6 |  |
| ATG4D | Autophagy related 4D cysteine peptidase | | | Q86TL0 |  |
| ATG5 | Autophagy related 5 | | | Q9H1Y0 |  |
| ATG7 | Autophagy related 7 | | | O95352 |  |
| ATG9A | Autophagy related 9A | | | Q7Z3C6 |  |
| ATIC | 5-aminoimidazole-4-carboxamide ribonucleotide formyltransferase/IMP cyclohydrolase | | | P31939 |  |
| ATL2 | Atlastin GTPase 2 | | | Q8NHH9 |  |
| ATL3 | Atlastin GTPase 3 | | | Q6DD88 |  |
| ATM | ATM serine/threonine kinase | | | Q13315 |  |
| ATMIN | ATM interactor | | | O43313 |  |
| ATOX1 | Antioxidant 1 copper chaperone | | | O00244 |  |
| ATP11B | ATPase phospholipid transporting 11B (putative) | | | Q9Y2G3 |  |
| ATP13A1 | ATPase 13A1 | | | Q9HD20 |  |
| ATP13A2 | ATPase cation transporting 13A2 | | | Q9NQ11 |  |
| ATP1A1 | ATPase Na+/K+ transporting subunit alpha 1 | | | P05023 |  |
| ATP1B1 | ATPase Na+/K+ transporting subunit beta 1 | | | P05026 |  |
| ATP1B3 | ATPase Na+/K+ transporting subunit beta 3 | | | P54709 |  |
| ATP2A3 | ATPase sarcoplasmic/endoplasmic reticulum Ca2+ transporting 3 | | | Q93084 |  |
| ATP2B4 | ATPase plasma membrane Ca2+ transporting 4 | | | P23634 |  |
| ATP2C1 | ATPase secretory pathway Ca2+ transporting 1 | | | P98194 |  |
| ATP6AP1 | ATPase H+ transporting accessory protein 1 | | | Q15904 |  |
| ATP6AP2 | ATPase H+ transporting accessory protein 2 | | | O75787 |  |
| ATP6V0A1 | ATPase H+ transporting V0 subunit a1 | | | Q93050 |  |
| ATP6V0A2 | ATPase H+ transporting V0 subunit a2 | | | Q9Y487 |  |
| ATP6V0B | ATPase H+ transporting V0 subunit b | | | Q99437 |  |
| ATP6V0C | ATPase H+ transporting V0 subunit c | | | P27449 |  |
| ATP6V0D1 | ATPase H+ transporting V0 subunit d1 | | | P61421 |  |
| ATP6V0E1 | ATPase H+ transporting V0 subunit e1 | | | O15342 |  |
| ATP6V0E2 | ATPase H+ transporting V0 subunit e2 | | | Q8NHE4 |  |
| ATP6V1B2 | ATPase H+ transporting V1 subunit B2 | | | P21281 |  |
| ATP6V1C1 | ATPase H+ transporting V1 subunit C1 | | | P21283 |  |
| ATP6V1D | ATPase H+ transporting V1 subunit D | | | Q9Y5K8 |  |
| ATP6V1E1 | ATPase H+ transporting V1 subunit E1 | | | P36543 |  |
| ATP6V1E2 | ATPase H+ transporting V1 subunit E2 | | | Q96A05 |  |
| ATP6V1F | ATPase H+ transporting V1 subunit F | | | Q16864 |  |
| ATP6V1G1 | ATPase H+ transporting V1 subunit G1 | | | O75348 |  |
| ATP6V1H | ATPase H+ transporting V1 subunit H | | | Q9UI12 |  |
| ATP9B | ATPase phospholipid transporting 9B (putative) | | | O43861 |  |
| ATPAF1 | ATP synthase mitochondrial F1 complex assembly factor 1 | | | Q5TC12 |  |
| ATPAF2 | ATP synthase mitochondrial F1 complex assembly factor 2 | | | Q8N5M1 |  |
| ATRAID | All-trans retinoic acid induced differentiation factor | | | Q6UW56 |  |
| ATRX | ATRX chromatin remodeler | | | P46100 |  |
| ATXN10 | Ataxin 10 | | | Q9UBB4 |  |
| ATXN3 | Ataxin 3 | | | P54252 |  |
| ATXN7 | Ataxin 7 | | | O15265 |  |
| ATXN7L1 | Ataxin 7 like 1 | | | Q9ULK2 |  |
| ATXN7L3 | Ataxin 7 like 3 | | | Q14CW9 |  |
| AUH | AU RNA binding methylglutaconyl-CoA hydratase | | | Q13825 |  |
| AUP1 | AUP1 lipid droplet regulating VLDL assembly factor | | | Q9Y679 |  |
| AURKA | Aurora kinase A | | | O14965 |  |
| AURKAIP1 | Aurora kinase A interacting protein 1 | | | Q9NWT8 |  |
| AURKB | Aurora kinase B | | | Q96GD4 |  |
| AVL9 | AVL9 cell migration associated | | | Q8NBF6 |  |
| AVPI1 | Arginine vasopressin induced 1 | | | Q5T686 |  |
| AXIN2 | Axin 2 | | | Q9Y2T1 |  |
| AZI2 | 5-azacytidine induced 2 | | | Q9H6S1 |  |
| AZIN1 | Antizyme inhibitor 1 | | | O14977 |  |
| AZIN2 | Antizyme inhibitor 2 | | | Q96A70 |  |
| B2M | Beta-2-microglobulin | | | P61769 |  |
| B3GALT2 | Beta-1,3-galactosyltransferase 2 | | | O43825 |  |
| B3GALT4 | Beta-1,3-galactosyltransferase 4 | | | O96024 |  |
| B3GALT6 | Beta-1,3-galactosyltransferase 6 | | | Q96L58 |  |
| B3GAT3 | Beta-1,3-glucuronyltransferase 3 | | | O94766 |  |
| B3GLCT | Beta 3-glucosyltransferase | | | Q6Y288 |  |
| B3GNT2 | UDP-GlcNAc:betaGal beta-1,3-N-acetylglucosaminyltransferase 2 | | | Q9NY97 |  |
| B3GNT8 | UDP-GlcNAc:betaGal beta-1,3-N-acetylglucosaminyltransferase 8 | | | Q7Z7M8 |  |
| B4GALT1 | Beta-1,4-galactosyltransferase 1 | | | P15291 |  |
| B4GALT3 | Beta-1,4-galactosyltransferase 3 | | | O60512 |  |
| B4GALT4 | Beta-1,4-galactosyltransferase 4 | | | O60513 |  |
| B4GALT7 | Beta-1,4-galactosyltransferase 7 | | | Q9UBV7 |  |
| B4GAT1 | Beta-1,4-glucuronyltransferase 1 | | | O43505 |  |
| B9D2 | B9 domain containing 2 | | | Q9BPU9 |  |
| BABAM1 | BRISC and BRCA1 A complex member 1 | | | Q9NWV8 |  |
| BACH1 | BTB domain and CNC homolog 1 | | | O14867 |  |
| BACH2 | BTB domain and CNC homolog 2 | | | Q9BYV9 |  |
| BAD | BCL2 associated agonist of cell death | | | Q92934 |  |
| BAG1 | BAG cochaperone 1 | | | Q99933 |  |
| BAG2 | BAG cochaperone 2 | | | O95816 |  |
| BAG3 | BAG cochaperone 3 | | | O95817 |  |
| BAG3 | BAG cochaperone 3 | | | O95817 |  |
| BAG4 | BAG cochaperone 4 | | | O95429 |  |
| BAG5 | BAG cochaperone 5 | | | Q9UL15 |  |
| BAG6 | BAG cochaperone 6 | | | P46379 |  |
| BAHD1 | Bromo adjacent homology domain containing 1 | | | Q8TBE0 |  |
| BAIAP2 | BAR/IMD domain containing adaptor protein 2 | | | Q9UQB8 |  |
| BAK1 | BCL2 antagonist/killer 1 | | | Q16611 |  |
| BANF1 | BAF nuclear assembly factor 1 | | | O75531 |  |
| BANP | BTG3 associated nuclear protein | | | Q8N9N5 |  |
| BAP1 | BRCA1 associated protein 1 | | | Q92560 |  |
| BATF | Basic leucine zipper ATF-like transcription factor | | | Q16520 |  |
| BAX | BCL2 associated X, apoptosis regulator | | | Q07812 |  |
| BAZ2A | Bromodomain adjacent to zinc finger domain 2A | | | Q9UIF9 |  |
| BAZ2B | Bromodomain adjacent to zinc finger domain 2B | | | Q9UIF8 |  |
| BBS1 | Bardet-Biedl syndrome 1 | | | Q8NFJ9 |  |
| BBS10 | Bardet-Biedl syndrome 10 | | | Q8TAM1 |  |
| BBS12 | Bardet-Biedl syndrome 12 | | | Q6ZW61 |  |
| BBS2 | Bardet-Biedl syndrome 2 | | | Q9BXC9 |  |
| BBS4 | Bardet-Biedl syndrome 4 | | | Q96RK4 |  |
| BBS7 | Bardet-Biedl syndrome 7 | | | Q8IWZ6 |  |
| BBX | BBX high mobility group box domain containing | | | Q8WY36 |  |
| BCAP29 | B cell receptor associated protein 29 | | | Q9UHQ4 |  |
| BCAP31 | B cell receptor associated protein 31 | | | P51572 |  |
| BCAS2 | BCAS2 pre-mRNA processing factor | | | O75934 |  |
| BCAS3 | BCAS3 microtubule associated cell migration factor | | | Q9H6U6 |  |
| BCAS4 | Breast carcinoma amplified sequence 4 | | | Q8TDM0 |  |
| BCAT2 | Branched chain amino acid transaminase 2 | | | O15382 |  |
| BCCIP | BRCA2 and CDKN1A interacting protein | | | Q9P287 |  |
| BCDIN3D | BCDIN3 domain containing RNA methyltransferase | | | Q7Z5W3 |  |
| BCKDHA | Branched chain keto acid dehydrogenase E1 subunit alpha | | | P12694 |  |
| BCKDHB | Branched chain keto acid dehydrogenase E1 subunit beta | | | P21953 |  |
| BCKDK | Branched chain keto acid dehydrogenase kinase | | | O14874 |  |
| BCL10 | BCL10 immune signaling adaptor | | | O95999 |  |
| BCL11A | BAF chromatin remodeling complex subunit BCL11A | | | Q9H165 |  |
| BCL11B | BAF chromatin remodeling complex subunit BCL11B | | | Q9C0K0 |  |
| BCL2 | BCL2 apoptosis regulator | | | P10415 |  |
| BCL2A1 | BCL2 related protein A1 | | | Q16548 |  |
| BCL2L1 | BCL2 like 1 | | | Q07817 |  |
| BCL2L11 | BCL2 like 11 | | | O43521 |  |
| BCL2L12 | BCL2 like 12 | | | Q9HB09 |  |
| BCL2L13 | BCL2 like 13 | | | Q9BXK5 |  |
| BCL3 | BCL3 transcription coactivator | | | P20749 |  |
| BCL6 | BCL6 transcription repressor | | | P41182 |  |
| BCL7B | BAF chromatin remodeling complex subunit BCL7B | | | Q9BQE9 |  |
| BCL7C | BAF chromatin remodeling complex subunit BCL7C | | | Q8WUZ0 |  |
| BCLAF1 | BCL2 associated transcription factor 1 | | | Q9NYF8 |  |
| BCOR | BCL6 corepressor | | | Q6W2J9 |  |
| BCS1L | BCS1 homolog, ubiquinol-cytochrome c reductase complex chaperone | | | Q9Y276 |  |
| BDH1 | 3-hydroxybutyrate dehydrogenase 1 | | | Q02338 |  |
| BDH2 | 3-hydroxybutyrate dehydrogenase 2 | | | Q9BUT1 |  |
| BECN1 | Beclin 1 | | | Q14457 |  |
| BEND5 | BEN domain containing 5 | | | Q7L4P6 |  |
| BET1 | Bet1 golgi vesicular membrane trafficking protein | | | O15155 |  |
| BET1L | Bet1 golgi vesicular membrane trafficking protein like | | | Q9NYM9 |  |
| BEX1 | Brain expressed X-linked 1 | | | Q9HBH7 |  |
| BEX2 | Brain expressed X-linked 2 | | | Q9BXY8 |  |
| BEX4 | Brain expressed X-linked 4 | | | Q9NWD9 |  |
| BEX5 | Brain expressed X-linked 5 | | | Q5H9J7 |  |
| BFAR | Bifunctional apoptosis regulator | | | Q9NZS9 |  |
| BFSP2 | Beaded filament structural protein 2 | | | Q13515 |  |
| BHLHE40 | Basic helix-loop-helix family member e40 | | | O14503 |  |
| BICD2 | BICD cargo adaptor 2 | | | Q8TD16 |  |
| BID | BH3 interacting domain death agonist | | | P55957 |  |
| BIK | BCL2 interacting killer | | | Q13323 |  |
| BIN1 | Bridging integrator 1 | | | O00499 |  |
| BIN2 | Bridging integrator 2 | | | Q9UBW5 |  |
| BIN3 | Bridging integrator 3 | | | Q9NQY0 |  |
| BIRC2 | Baculoviral IAP repeat containing 2 | | | Q13490 |  |
| BIRC3 | Baculoviral IAP repeat containing 3 | | | Q13489 |  |
| BIRC5 | Baculoviral IAP repeat containing 5 | | | O15392 |  |
| BIVM | Basic, immunoglobulin-like variable motif containing | | | Q86UB2 |  |
| BLCAP | BLCAP apoptosis inducing factor | | | P62952 |  |
| BLK | BLK proto-oncogene, Src family tyrosine kinase | | | P51451 |  |
| BLMH | Bleomycin hydrolase | | | Q13867 |  |
| BLOC1S1 | Biogenesis of lysosomal organelles complex 1 subunit 1 | | | P78537 |  |
| BLOC1S2 | Biogenesis of lysosomal organelles complex 1 subunit 2 | | | Q6QNY1 |  |
| BLOC1S4 | Biogenesis of lysosomal organelles complex 1 subunit 4 | | | Q9NUP1 |  |
| BLOC1S5 | Biogenesis of lysosomal organelles complex 1 subunit 5 | | | Q8TDH9 |  |
| BLOC1S6 | Biogenesis of lysosomal organelles complex 1 subunit 6 | | | Q9UL45 |  |
| BLVRA | Biliverdin reductase A | | | P53004 |  |
| BLVRB | Biliverdin reductase B | | | P30043 |  |
| BLZF1 | Basic leucine zipper nuclear factor 1 | | | Q9H2G9 |  |
| BMI1 | BMI1 proto-oncogene, polycomb ring finger | | | P35226 |  |
| BNIP1 | BCL2 interacting protein 1 | | | Q12981 |  |
| BNIP2 | BCL2 interacting protein 2 | | | Q12982 |  |
| BNIP3 | BCL2 interacting protein 3 | | | Q12983 |  |
| BNIP3L | BCL2 interacting protein 3 like | | | O60238 |  |
| BOD1 | Biorientation of chromosomes in cell division 1 | | | Q96IK1 |  |
| BOLA1 | BolA family member 1 | | | Q9Y3E2 |  |
| BOLA2 | BolA family member 2 | | | Q9H3K6 |  |
| BOLA3 | BolA family member 3 | | | Q53S33 |  |
| BOP1 | BOP1 ribosomal biogenesis factor | | | Q14137 |  |
| BORA | BORA aurora kinase A activator | | | Q6PGQ7 |  |
| BPGM | Bisphosphoglycerate mutase | | | P07738 |  |
| BPHL | Biphenyl hydrolase like | | | Q86WA6 |  |
| BPNT1 | 3'(2'), 5'-bisphosphate nucleotidase 1 | | | O95861 |  |
| BRAF | B-Raf proto-oncogene, serine/threonine kinase | | | P15056 |  |
| BRAT1 | BRCA1 associated ATM activator 1 | | | Q6PJG6 |  |
| BRCA1 | BRCA1 DNA repair associated | | | P38398 |  |
| BRCC3 | BRCA1/BRCA2-containing complex subunit 3 | | | P46736 |  |
| BRD2 | Bromodomain containing 2 | | | P25440 |  |
| BRD3 | Bromodomain containing 3 | | | Q15059 |  |
| BRD4 | Bromodomain containing 4 | | | O60885 |  |
| BRD7 | Bromodomain containing 7 | | | Q9NPI1 |  |
| BRD9 | Bromodomain containing 9 | | | Q9H8M2 |  |
| BRF1 | BRF1 RNA polymerase III transcription initiation factor subunit | | | Q92994 |  |
| BRF2 | BRF2 RNA polymerase III transcription initiation factor subunit | | | Q9HAW0 |  |
| BRI3 | Brain protein I3 | | | O95415 |  |
| BRIX1 | Biogenesis of ribosomes BRX1 | | | Q8TDN6 |  |
| BRK1 | BRICK1 subunit of SCAR/WAVE actin nucleating complex | | | Q8WUW1 |  |
| BRMS1 | BRMS1 transcriptional repressor and anoikis regulator | | | Q9HCU9 |  |
| BRMS1L | BRMS1 like transcriptional repressor | | | Q5PSV4 |  |
| BROX | BRO1 domain and CAAX motif containing | | | Q5VW32 |  |
| BRPF1 | Bromodomain and PHD finger containing 1 | | | P55201 |  |
| BRWD1 | Bromodomain and WD repeat domain containing 1 | | | Q9NSI6 |  |
| BSCL2 | BSCL2 lipid droplet biogenesis associated, seipin | | | Q96G97 |  |
| BSDC1 | BSD domain containing 1 | | | Q9NW68 |  |
| BSG | Basigin (Ok blood group) | | | P35613 |  |
| BST1 | Bone marrow stromal cell antigen 1 | | | Q10588 |  |
| BST2 | Bone marrow stromal cell antigen 2 | | | Q10589 |  |
| BTBD1 | BTB domain containing 1 | | | Q9H0C5 |  |
| BTBD10 | BTB domain containing 10 | | | Q9BSF8 |  |
| BTBD2 | BTB domain containing 2 | | | Q9BX70 |  |
| BTBD9 | BTB domain containing 9 | | | Q96Q07 |  |
| BTD | Biotinidase | | | P43251 |  |
| BTF3 | Basic transcription factor 3 | | | P20290 |  |
| BTF3L4 | Basic transcription factor 3 like 4 | | | Q96K17 |  |
| BTG1 | BTG anti-proliferation factor 1 | | | P62324 |  |
| BTG2 | BTG anti-proliferation factor 2 | | | P78543 |  |
| BTG3 | BTG anti-proliferation factor 3 | | | Q14201 |  |
| BTLA | B and T lymphocyte associated | | | Q7Z6A9 |  |
| BTN2A1 | Butyrophilin subfamily 2 member A1 | | | Q7KYR7 |  |
| BTN2A2 | Butyrophilin subfamily 2 member A2 | | | Q8WVV5 |  |
| BTN3A1 | Butyrophilin subfamily 3 member A1 | | | O00481 |  |
| BTN3A2 | Butyrophilin subfamily 3 member A2 | | | P78410 |  |
| BTN3A3 | Butyrophilin subfamily 3 member A3 | | | O00478 |  |
| BTRC | Beta-transducin repeat containing E3 ubiquitin protein ligase | | | Q9Y297 |  |
| BUB3 | BUB3 mitotic checkpoint protein | | | O43684 |  |
| BUD13 | BUD13 homolog | | | Q9BRD0 |  |
| BUD31 | BUD31 homolog | | | P41223 |  |
| BYSL | Bystin like | | | Q13895 |  |
| BZW1 | Basic leucine zipper and W2 domains 1 | | | Q7L1Q6 |  |
| BZW2 | Basic leucine zipper and W2 domains 2 | | | Q9Y6E2 |  |
| C10orf88 | Chromosome 10 open reading frame 88 | | | Q9H8K7 |  |
| C11orf1 | Chromosome 11 open reading frame 1 | | | Q9H5F2 |  |
| C11orf54 | Chromosome 11 open reading frame 54 | | | Q9H0W9 |  |
| C11orf68 | Chromosome 11 open reading frame 68 | | | Q9H3H3 |  |
| C11orf71 | Chromosome 11 open reading frame 71 | | | Q6IPW1 |  |
| C11orf98 | Chromosome 11 open reading frame 98 | | | E9PRG8 |  |
| C12orf4 | Chromosome 12 open reading frame 4 | | | Q9NQ89 |  |
| C12orf42 | Chromosome 12 open reading frame 42 | | | Q96LP6 |  |
| C12orf43 | Chromosome 12 open reading frame 43 | | | Q96C57 |  |
| C12orf57 | Chromosome 12 open reading frame 57 | | | Q99622 |  |
| C12orf60 | Chromosome 12 open reading frame 60 | | | Q5U649 |  |
| C14orf119 | Chromosome 14 open reading frame 119 | | | Q9NWQ9 |  |
| C14orf28 | Chromosome 14 open reading frame 28 | | | Q4W4Y0 |  |
| C14orf93 | Chromosome 14 open reading frame 93 | | | Q9H972 |  |
| C16orf54 | Chromosome 16 open reading frame 54 | | | Q6UWD8 |  |
| C16orf74 | Chromosome 16 open reading frame 74 | | | Q96GX8 |  |
| C16orf87 | Chromosome 16 open reading frame 87 | | | Q6PH81 |  |
| C17orf50 | Chromosome 17 open reading frame 50 | | | Q8WW18 |  |
| C17orf75 | Chromosome 17 open reading frame 75 | | | Q9HAS0 |  |
| C17orf80 | Chromosome 17 open reading frame 80 | | | Q9BSJ5 |  |
| C18orf21 | Chromosome 18 open reading frame 21 | | | Q32NC0 |  |
| C18orf25 | Chromosome 18 open reading frame 25 | | | Q96B23 |  |
| C18orf32 | Chromosome 18 open reading frame 32 | | | Q8TCD1 |  |
| C19orf12 | Chromosome 19 open reading frame 12 | | | Q9NSK7 |  |
| C19orf25 | Chromosome 19 open reading frame 25 | | | Q9UFG5 |  |
| C19orf47 | Chromosome 19 open reading frame 47 | | | Q8N9M1 |  |
| C19orf53 | Chromosome 19 open reading frame 53 | | | Q9UNZ5 |  |
| C19orf54 | Chromosome 19 open reading frame 54 | | | Q5BKX5 |  |
| C19orf73 | Chromosome 19 open reading frame 73 | | | Q9NVV2 |  |
| C1D | C1D nuclear receptor corepressor | | | Q13901 |  |
| C1GALT1C1 | C1GALT1 specific chaperone 1 | | | Q96EU7 |  |
| C1orf109 | Chromosome 1 open reading frame 109 | | | Q9NX04 |  |
| C1orf131 | Chromosome 1 open reading frame 131 | | | Q8NDD1 |  |
| C1orf159 | Chromosome 1 open reading frame 159 | | | Q96HA4 |  |
| C1orf162 | Chromosome 1 open reading frame 162 | | | Q8NEQ5 |  |
| C1orf174 | Chromosome 1 open reading frame 174 | | | Q8IYL3 |  |
| C1orf198 | Chromosome 1 open reading frame 198 | | | Q9H425 |  |
| C1orf43 | Chromosome 1 open reading frame 43 | | | Q9BWL3 |  |
| C1orf54 | Chromosome 1 open reading frame 54 | | | Q8WWF1 |  |
| C1orf74 | Chromosome 1 open reading frame 74 | | | Q96LT6 |  |
| C1QBP | Complement C1q binding protein | | | Q07021 |  |
| C20orf27 | Chromosome 20 open reading frame 27 | | | Q9GZN8 |  |
| C22orf39 | Chromosome 22 open reading frame 39 | | | Q6P5X5 |  |
| C2orf15 | Chromosome 2 open reading frame 15 | | | Q8WU43 |  |
| C2orf42 | Chromosome 2 open reading frame 42 | | | Q9NWW7 |  |
| C2orf49 | Chromosome 2 open reading frame 49 | | | Q9BVC5 |  |
| C2orf68 | Chromosome 2 open reading frame 68 | | | Q2NKX9 |  |
| C2orf74 | Chromosome 2 open reading frame 74 | | | A8MZ97 |  |
| C2orf76 | Chromosome 2 open reading frame 76 | | | Q3KRA6 |  |
| C3AR1 | Complement C3a receptor 1 | | | Q16581 |  |
| C3orf14 | Chromosome 3 open reading frame 14 | | | Q9HBI5 |  |
| C3orf18 | Chromosome 3 open reading frame 18 | | | Q9UK00 |  |
| C3orf33 | Chromosome 3 open reading frame 33 | | | Q6P1S2 |  |
| C3orf38 | Chromosome 3 open reading frame 38 | | | Q5JPI3 |  |
| C3orf62 | Chromosome 3 open reading frame 62 | | | Q6ZUJ4 |  |
| C4orf33 | Chromosome 4 open reading frame 33 | | | Q8N1A6 |  |
| C4orf46 | Chromosome 4 open reading frame 46 | | | Q504U0 |  |
| C5orf22 | Chromosome 5 open reading frame 22 | | | Q49AR2 |  |
| C5orf24 | Chromosome 5 open reading frame 24 | | | Q7Z6I8 |  |
| C6orf136 | Chromosome 6 open reading frame 136 | | | Q5SQH8 |  |
| C6orf226 | Chromosome 6 open reading frame 226 | | | Q5I0X4 |  |
| C6orf62 | Chromosome 6 open reading frame 62 | | | Q9GZU0 |  |
| C6orf89 | Chromosome 6 open reading frame 89 | | | Q6UWU4 |  |
| C7orf25 | Chromosome 7 open reading frame 25 | | | Q9BPX7 |  |
| C7orf50 | Chromosome 7 open reading frame 50 | | | Q9BRJ6 |  |
| C8orf33 | Chromosome 8 open reading frame 33 | | | Q9H7E9 |  |
| C8orf76 | Chromosome 8 open reading frame 76 | | | Q96K31 |  |
| C9orf72 | C9orf72-SMCR8 complex subunit | | | Q96LT7 |  |
| C9orf78 | Chromosome 9 open reading frame 78 | | | Q9NZ63 |  |
| C9orf85 | Chromosome 9 open reading frame 85 | | | Q96MD7 |  |
| CA11 | Carbonic anhydrase 11 | | | O75493 |  |
| CA5B | Carbonic anhydrase 5B | | | Q9Y2D0 |  |
| CA6 | Carbonic anhydrase 6 | | | P23280 |  |
| CAAP1 | Caspase activity and apoptosis inhibitor 1 | | | Q9H8G2 |  |
| CAB39 | Calcium binding protein 39 | | | Q9Y376 |  |
| CAB39L | Calcium binding protein 39 like | | | Q9H9S4 |  |
| CACNA2D4 | Calcium voltage-gated channel auxiliary subunit alpha2delta 4 | | | Q7Z3S7 |  |
| CACUL1 | CDK2 associated cullin domain 1 | | | Q86Y37 |  |
| CACYBP | Calcyclin binding protein | | | Q9HB71 |  |
| CADM1 | Cell adhesion molecule 1 | | | Q9BY67 |  |
| CALCOCO1 | Calcium binding and coiled-coil domain 1 | | | Q9P1Z2 |  |
| CALCOCO2 | Calcium binding and coiled-coil domain 2 | | | Q13137 |  |
| CALM1 | Calmodulin 1 | | | P0DP23 |  |
| CALR | Calreticulin | | | P27797 |  |
| CALU | Calumenin | | | O43852 |  |
| CAMK1D | Calcium/calmodulin dependent protein kinase ID | | | Q8IU85 |  |
| CAMK2D | Calcium/calmodulin dependent protein kinase II delta | | | Q13557 |  |
| CAMK2G | Calcium/calmodulin dependent protein kinase II gamma | | | Q13555 |  |
| CAMK4 | Calcium/calmodulin dependent protein kinase IV | | | Q16566 |  |
| CAMKK2 | Calcium/calmodulin dependent protein kinase kinase 2 | | | Q96RR4 |  |
| CAMKMT | Calmodulin-lysine N-methyltransferase | | | Q7Z624 |  |
| CAMLG | Calcium modulating ligand | | | P49069 |  |
| CAMTA1 | Calmodulin binding transcription activator 1 | | | Q9Y6Y1 |  |
| CAND1 | Cullin associated and neddylation dissociated 1 | | | Q86VP6 |  |
| CANT1 | Calcium activated nucleotidase 1 | | | Q8WVQ1 |  |
| CANX | Calnexin | | | P27824 |  |
| CAP1 | Cyclase associated actin cytoskeleton regulatory protein 1 | | | Q01518 |  |
| CAPG | Capping actin protein, gelsolin like | | | P40121 |  |
| CAPN1 | Calpain 1 | | | P07384 |  |
| CAPN12 | Calpain 12 | | | Q6ZSI9 |  |
| CAPN2 | Calpain 2 | | | P17655 |  |
| CAPN7 | Calpain 7 | | | Q9Y6W3 |  |
| CAPNS1 | Calpain small subunit 1 | | | P04632 |  |
| CAPRIN1 | Cell cycle associated protein 1 | | | Q14444 |  |
| CAPS | Calcyphosine | | | Q13938 |  |
| CAPZA1 | Capping actin protein of muscle Z-line subunit alpha 1 | | | P52907 |  |
| CAPZA2 | Capping actin protein of muscle Z-line subunit alpha 2 | | | P47755 |  |
| CARD16 | Caspase recruitment domain family member 16 | | | Q5EG05 |  |
| CARD19 | Caspase recruitment domain family member 19 | | | Q96LW7 |  |
| CARD8 | Caspase recruitment domain family member 8 | | | Q9Y2G2 |  |
| CARF | Calcium responsive transcription factor | | | Q8N187 |  |
| CARHSP1 | Calcium regulated heat stable protein 1 | | | Q9Y2V2 |  |
| CARNMT1 | Carnosine N-methyltransferase 1 | | | Q8N4J0 |  |
| CASK | Calcium/calmodulin dependent serine protein kinase | | | O14936 |  |
| CASP1 | Caspase 1 | | | P29466 |  |
| CASP10 | Caspase 10 | | | Q92851 |  |
| CASP2 | Caspase 2 | | | P42575 |  |
| CASP3 | Caspase 3 | | | P42574 |  |
| CASP4 | Caspase 4 | | | P49662 |  |
| CASP6 | Caspase 6 | | | P55212 |  |
| CASP7 | Caspase 7 | | | P55210 |  |
| CASP8 | Caspase 8 | | | Q14790 |  |
| CASP8AP2 | Caspase 8 associated protein 2 | | |  |  |
| CASP9 | Caspase 9 | | | P55211 |  |
| CASS4 | Cas scaffold protein family member 4 | | | Q9NQ75 |  |
| CAST | Calpastatin | | | P20810 |  |
| CAT | Catalase | | | P04040 |  |
| CBFA2T2 | CBFA2/RUNX1 partner transcriptional co-repressor 2 | | | O43439 |  |
| CBFB | Core-binding factor subunit beta | | | Q13951 |  |
| CBL | Cbl proto-oncogene | | | P22681 |  |
| CBLB | Cbl proto-oncogene B | | | Q13191 |  |
| CBLL1 | Cbl proto-oncogene like 1 | | | Q75N03 |  |
| CBR1 | Carbonyl reductase 1 | | | P16152 |  |
| CBR3 | Carbonyl reductase 3 | | | O75828 |  |
| CBR4 | Carbonyl reductase 4 | | | Q8N4T8 |  |
| CBX1 | Chromobox 1 | | | P83916 |  |
| CBX3 | Chromobox 3 | | | Q13185 |  |
| CBX4 | Chromobox 4 | | | O00257 |  |
| CBX5 | Chromobox 5 | | | P45973 |  |
| CBX6 | Chromobox 6 | | | O95503 |  |
| CBX7 | Chromobox 7 | | | O95931 |  |
| CBX8 | Chromobox 8 | | | Q9HC52 |  |
| CBY1 | Chibby family member 1, beta catenin antagonist | | | Q9Y3M2 |  |
| CCAR2 | Cell cycle and apoptosis regulator 2 | | | Q8N163 |  |
| CCDC106 | Coiled-coil domain containing 106 | | | Q9BWC9 |  |
| CCDC107 | Coiled-coil domain containing 107 | | | Q8WV48 |  |
| CCDC112 | Coiled-coil domain containing 112 | | | Q8NEF3 |  |
| CCDC115 | Coiled-coil domain containing 115 | | | Q96NT0 |  |
| CCDC117 | Coiled-coil domain containing 117 | | | Q8IWD4 |  |
| CCDC12 | Coiled-coil domain containing 12 | | | Q8WUD4 |  |
| CCDC121 | Coiled-coil domain containing 121 | | | Q6ZUS5 |  |
| CCDC124 | Coiled-coil domain containing 124 | | | Q96CT7 |  |
| CCDC126 | Coiled-coil domain containing 126 | | | Q96EE4 |  |
| CCDC127 | Coiled-coil domain containing 127 | | | Q96BQ5 |  |
| CCDC134 | Coiled-coil domain containing 134 | | | Q9H6E4 |  |
| CCDC137 | Coiled-coil domain containing 137 | | | Q6PK04 |  |
| CCDC138 | Coiled-coil domain containing 138 | | | Q96M89 |  |
| CCDC14 | Coiled-coil domain containing 14 | | | Q49A88 |  |
| CCDC141 | Coiled-coil domain containing 141 | | | Q6ZP82 |  |
| CCDC142 | Coiled-coil domain containing 142 | | | Q17RM4 |  |
| CCDC146 | Coiled-coil domain containing 146 | | | Q8IYE0 |  |
| CCDC167 | Coiled-coil domain containing 167 | | | Q9P0B6 |  |
| CCDC18 | Coiled-coil domain containing 18 | | | Q5T9S5 |  |
| CCDC186 | Coiled-coil domain containing 186 | | | Q7Z3E2 |  |
| CCDC191 | Coiled-coil domain containing 191 | | | Q8NCU4 |  |
| CCDC22 | Coiled-coil domain containing 22 | | | O60826 |  |
| CCDC25 | Coiled-coil domain containing 25 | | | Q86WR0 |  |
| CCDC28A | Coiled-coil domain containing 28A | | |  |  |
| CCDC28B | Coiled-coil domain containing 28B | | | Q9BUN5 |  |
| CCDC34 | Coiled-coil domain containing 34 | | | Q96HJ3 |  |
| CCDC43 | Coiled-coil domain containing 43 | | | Q96MW1 |  |
| CCDC47 | Coiled-coil domain containing 47 | | | Q96A33 |  |
| CCDC50 | Coiled-coil domain containing 50 | | | Q8IVM0 |  |
| CCDC51 | Coiled-coil domain containing 51 | | | Q96ER9 |  |
| CCDC57 | Coiled-coil domain containing 57 | | | Q2TAC2 |  |
| CCDC59 | Coiled-coil domain containing 59 | | | Q9P031 |  |
| CCDC6 | Coiled-coil domain containing 6 | | | Q16204 |  |
| CCDC65 | Coiled-coil domain containing 65 | | | Q8IXS2 |  |
| CCDC66 | Coiled-coil domain containing 66 | | | A2RUB6 |  |
| CCDC69 | Coiled-coil domain containing 69 | | | A6NI79 |  |
| CCDC7 | Coiled-coil domain containing 7 | | | Q96M83 |  |
| CCDC71 | Coiled-coil domain containing 71 | | | Q8IV32 |  |
| CCDC77 | Coiled-coil domain containing 77 | | | Q9BR77 |  |
| CCDC82 | Coiled-coil domain containing 82 | | | Q8N4S0 |  |
| CCDC85B | Coiled-coil domain containing 85B | | | Q15834 |  |
| CCDC86 | Coiled-coil domain containing 86 | | | Q9H6F5 |  |
| CCDC88C | Coiled-coil domain containing 88C | | | Q9P219 |  |
| CCDC9 | Coiled-coil domain containing 9 | | | Q9Y3X0 |  |
| CCDC90B | Coiled-coil domain containing 90B | | | Q9GZT6 |  |
| CCDC91 | Coiled-coil domain containing 91 | | | Q7Z6B0 |  |
| CCDC92 | Coiled-coil domain containing 92 | | | Q53HC0 |  |
| CCDC93 | Coiled-coil domain containing 93 | | | Q567U6 |  |
| CCDC97 | Coiled-coil domain containing 97 | | | Q96F63 |  |
| CCL20 | C-C motif chemokine ligand 20 | | | P78556 |  |
| CCL28 | C-C motif chemokine ligand 28 | | | Q9NRJ3 |  |
| CCL4 | C-C motif chemokine ligand 4 | | | P13236 |  |
| CCL5 | C-C motif chemokine ligand 5 | | | P13501 |  |
| CCM2 | CCM2 scaffold protein | | | Q9BSQ5 |  |
| CCNB1 | Cyclin B1 | | | P14635 |  |
| CCNB1IP1 | Cyclin B1 interacting protein 1 | | | Q9NPC3 |  |
| CCNB2 | Cyclin B2 | | | O95067 |  |
| CCNC | Cyclin C | | | P24863 |  |
| CCND2 | Cyclin D2 | | | P30279 |  |
| CCND3 | Cyclin D3 | | | P30281 |  |
| CCNDBP1 | Cyclin D1 binding protein 1 | | | O95273 |  |
| CCNE2 | Cyclin E2 | | | O96020 |  |
| CCNG1 | Cyclin G1 | | | P51959 |  |
| CCNG2 | Cyclin G2 | | | Q16589 |  |
| CCNH | Cyclin H | | | P51946 |  |
| CCNI | Cyclin I | | | Q14094 |  |
| CCNJ | Cyclin J | | | Q5T5M9 |  |
| CCNK | Cyclin K | | | O75909 |  |
| CCNL1 | Cyclin L1 | | | Q9UK58 |  |
| CCNL2 | Cyclin L2 | | | Q96S94 |  |
| CCNT1 | Cyclin T1 | | | O60563 |  |
| CCNY | Cyclin Y | | | Q8ND76 |  |
| CCP110 | Centriolar coiled-coil protein 110 | | | O43303 |  |
| CCR10 | C-C motif chemokine receptor 10 | | | P46092 |  |
| CCR2 | C-C motif chemokine receptor 2 | | | P41597 |  |
| CCR3 | C-C motif chemokine receptor 3 | | | P51677 |  |
| CCR4 | C-C motif chemokine receptor 4 | | | P51679 |  |
| CCR5 | C-C motif chemokine receptor 5 | | | P51681 |  |
| CCR6 | C-C motif chemokine receptor 6 | | | P51684 |  |
| CCR7 | C-C motif chemokine receptor 7 | | | P32248 |  |
| CCR7 | C-C motif chemokine receptor 7 | | | P32248 |  |
| CCR8 | C-C motif chemokine receptor 8 | | | P51685 |  |
| CCR9 | C-C motif chemokine receptor 9 | | | P51686 |  |
| CCRL2 | C-C motif chemokine receptor like 2 | | | O00421 |  |
| CCS | Copper chaperone for superoxide dismutase | | | O14618 |  |
| CCSER2 | Coiled-coil serine rich protein 2 | | | Q9H7U1 |  |
| CCT2 | Chaperonin containing TCP1 subunit 2 | | | P78371 |  |
| CCT3 | Chaperonin containing TCP1 subunit 3 | | | P49368 |  |
| CCT4 | Chaperonin containing TCP1 subunit 4 | | | P50991 |  |
| CCT5 | Chaperonin containing TCP1 subunit 5 | | | P48643 |  |
| CCT6A | Chaperonin containing TCP1 subunit 6A | | | P40227 |  |
| CCT6B | Chaperonin containing TCP1 subunit 6B | | | Q92526 |  |
| CCT7 | Chaperonin containing TCP1 subunit 7 | | | Q99832 |  |
| CCT8 | Chaperonin containing TCP1 subunit 8 | | | P50990 |  |
| CCZ1B | CCZ1 homolog B, vacuolar protein trafficking and biogenesis associated | | | P86790 |  |
| CD14 | CD14 molecule | | | P08571 |  |
| CD151 | CD151 molecule (Raph blood group) | | | P48509 |  |
| CD160 | CD160 molecule | | | O95971 |  |
| CD164 | CD164 molecule | | | Q04900 |  |
| CD2 | CD2 molecule | | | P06729 |  |
| CD200 | CD200 molecule | | | P41217 |  |
| CD200R1 | CD200 receptor 1 | | | Q8TD46 |  |
| CD226 | CD226 molecule | | | Q15762 |  |
| CD244 | CD244 molecule | | | Q9BZW8 |  |
| CD247 | CD247 molecule | | | P20963 |  |
| CD248 | CD248 molecule | | | Q9HCU0 |  |
| CD27 | CD27 molecule | | | P26842 |  |
| CD274 | CD274 molecule | | | Q9NZQ7 |  |
| CD28 | CD28 molecule | | | P10747 |  |
| CD2BP2 | CD2 cytoplasmic tail binding protein 2 | | | O95400 |  |
| CD300A | CD300a molecule | | | Q9UGN4 |  |
| CD320 | CD320 molecule | | | Q9NPF0 |  |
| CD37 | CD37 molecule | | | P11049 |  |
| CD38 | CD38 molecule | | | P28907 |  |
| CD3D | CD3 delta subunit of T-cell receptor complex | | | P04234 |  |
| CD3E | CD3 epsilon subunit of T-cell receptor complex | | | P07766 |  |
| CD4 | CD4 molecule | | | P01730 |  |
| CD40 | CD40 molecule | | | P25942 |  |
| CD40LG | CD40 ligand | | | P29965 |  |
| CD44 | CD44 molecule (Indian blood group) | | | P16070 |  |
| CD46 | CD46 molecule | | | P15529 |  |
| CD47 | CD47 molecule | | | Q08722 |  |
| CD48 | CD48 molecule | | | P09326 |  |
| CD5 | CD5 molecule | | | P06127 |  |
| CD52 | CD52 molecule | | | P31358 |  |
| CD53 | CD53 molecule | | | P19397 |  |
| CD55 | CD55 molecule (Cromer blood group) | | | P08174 |  |
| CD58 | CD58 molecule | | | P19256 |  |
| CD59 | CD59 molecule (CD59 blood group) | | | P13987 |  |
| CD6 | CD6 molecule | | | P30203 |  |
| CD63 | CD63 molecule | | | P08962 |  |
| CD68 | CD68 molecule | | | P34810 |  |
| CD69 | CD69 molecule | | | Q07108 |  |
| CD7 | CD7 molecule | | | P09564 |  |
| CD70 | CD70 molecule | | | P32970 |  |
| CD72 | CD72 molecule | | | P21854 |  |
| CD74 | CD74 molecule | | | P04233 |  |
| CD79A | CD79a molecule | | | P11912 |  |
| CD79B | CD79b molecule | | | P40259 |  |
| CD81 | CD81 molecule | | | P60033 |  |
| CD82 | CD82 molecule | | | P27701 |  |
| CD83 | CD83 molecule | | | Q01151 |  |
| CD84 | CD84 molecule | | | Q9UIB8 |  |
| CD8A | CD8a molecule | | | P01732 |  |
| CD8B | CD8b molecule | | | P10966 |  |
| CD9 | CD9 molecule | | | P21926 |  |
| CD96 | CD96 molecule | | | P40200 |  |
| CD99 | CD99 molecule (Xg blood group) | | | P14209 |  |
| CD99L2 | CD99 molecule like 2 | | | Q8TCZ2 |  |
| CDADC1 | Cytidine and dCMP deaminase domain containing 1 | | | Q9BWV3 |  |
| CDC123 | Cell division cycle 123 | | | O75794 |  |
| CDC14A | Cell division cycle 14A | | | Q9UNH5 |  |
| CDC14B | Cell division cycle 14B | | | O60729 |  |
| CDC16 | Cell division cycle 16 | | | Q13042 |  |
| CDC20 | Cell division cycle 20 | | | Q12834 |  |
| CDC23 | Cell division cycle 23 | | | Q9UJX2 |  |
| CDC25B | Cell division cycle 25B | | | P30305 |  |
| CDC26 | Cell division cycle 26 | | | Q8NHZ8 |  |
| CDC27 | Cell division cycle 27 | | | P30260 |  |
| CDC34 | Cell division cycle 34, ubiqiutin conjugating enzyme | | | P49427 |  |
| CDC37 | Cell division cycle 37, HSP90 cochaperone | | | Q16543 |  |
| CDC37L1 | Cell division cycle 37 like 1, HSP90 cochaperone | | | Q7L3B6 |  |
| CDC40 | Cell division cycle 40 | | | O60508 |  |
| CDC42 | Cell division cycle 42 | | | P60953 |  |
| CDC42EP3 | CDC42 effector protein 3 | | | Q9UKI2 |  |
| CDC42SE1 | CDC42 small effector 1 | | | Q9NRR8 |  |
| CDC42SE2 | CDC42 small effector 2 | | | Q9NRR3 |  |
| CDC5L | Cell division cycle 5 like | | | Q99459 |  |
| CDC7 | Cell division cycle 7 | | | O00311 |  |
| CDC73 | Cell division cycle 73 | | | Q6P1J9 |  |
| CDCA4 | Cell division cycle associated 4 | | | Q9BXL8 |  |
| CDCA7 | Cell division cycle associated 7 | | | Q9BWT1 |  |
| CDCA7L | Cell division cycle associated 7 like | | | Q96GN5 |  |
| CDCA8 | Cell division cycle associated 8 | | | Q53HL2 |  |
| CDIP1 | Cell death inducing p53 target 1 | | | Q9H305 |  |
| CDIPT | CDP-diacylglycerol--inositol 3-phosphatidyltransferase | | | O14735 |  |
| CDK10 | Cyclin dependent kinase 10 | | | Q15131 |  |
| CDK11A | Cyclin dependent kinase 11A | | | Q9UQ88 |  |
| CDK11B | Cyclin dependent kinase 11B | | | P21127 |  |
| CDK12 | Cyclin dependent kinase 12 | | | Q9NYV4 |  |
| CDK13 | Cyclin dependent kinase 13 | | | Q14004 |  |
| CDK16 | Cyclin dependent kinase 16 | | | Q00536 |  |
| CDK17 | Cyclin dependent kinase 17 | | | Q00537 |  |
| CDK20 | Cyclin dependent kinase 20 | | | Q8IZL9 |  |
| CDK2AP2 | Cyclin dependent kinase 2 associated protein 2 | | | O75956 |  |
| CDK4 | Cyclin dependent kinase 4 | | | P11802 |  |
| CDK5 | Cyclin dependent kinase 5 | | | Q00535 |  |
| CDK5R1 | Cyclin dependent kinase 5 regulatory subunit 1 | | | Q15078 |  |
| CDK5RAP1 | CDK5 regulatory subunit associated protein 1 | | | Q96SZ6 |  |
| CDK5RAP3 | CDK5 regulatory subunit associated protein 3 | | | Q96JB5 |  |
| CDK7 | Cyclin dependent kinase 7 | | | P50613 |  |
| CDK9 | Cyclin dependent kinase 9 | | | P50750 |  |
| CDKAL1 | CDK5 regulatory subunit associated protein 1 like 1 | | | Q5VV42 |  |
| CDKL1 | Cyclin dependent kinase like 1 | | | Q00532 |  |
| CDKL3 | Cyclin dependent kinase like 3 | | | Q8IVW4 |  |
| CDKN1A | Cyclin dependent kinase inhibitor 1A | | | P38936 |  |
| CDKN1B | Cyclin dependent kinase inhibitor 1B | | | P46527 |  |
| CDKN2A | Cyclin dependent kinase inhibitor 2A | | | P42771, Q8N726 |  |
| CDKN2AIP | CDKN2A interacting protein | | | Q9NXV6 |  |
| CDKN2AIPNL | CDKN2A interacting protein N-terminal like | | | Q96HQ2 |  |
| CDKN2C | Cyclin dependent kinase inhibitor 2C | | | P42773 |  |
| CDKN2D | Cyclin dependent kinase inhibitor 2D | | | P55273 |  |
| CDKN3 | Cyclin dependent kinase inhibitor 3 | | | Q16667 |  |
| CDNF | Cerebral dopamine neurotrophic factor | | | Q49AH0 |  |
| CDO1 | Cysteine dioxygenase type 1 | | | Q16878 |  |
| CDPF1 | Cysteine rich DPF motif domain containing 1 | | | Q6NVV7 |  |
| CDR2 | Cerebellar degeneration related protein 2 | | | Q01850 |  |
| CDS2 | CDP-diacylglycerol synthase 2 | | | O95674 |  |
| CDV3 | CDV3 homolog | | | Q9UKY7 |  |
| CDYL | Chromodomain Y like | | | Q9Y232 |  |
| CEACAM1 | CEA cell adhesion molecule 1 | | | P13688 |  |
| CEACAM21 | CEA cell adhesion molecule 21 | | | Q3KPI0 |  |
| CEACAM4 | CEA cell adhesion molecule 4 | | | O75871 |  |
| CEBPG | CCAAT enhancer binding protein gamma | | | P53567 |  |
| CEBPZ | CCAAT enhancer binding protein zeta | | | Q03701 |  |
| CELA1 | Chymotrypsin like elastase 1 | | | Q9UNI1 |  |
| CELA1 | Chymotrypsin like elastase 1 | | | Q9UNI1 |  |
| CELF1 | CUGBP Elav-like family member 1 | | | Q92879 |  |
| CELF2 | CUGBP Elav-like family member 2 | | | O95319 |  |
| CENPB | Centromere protein B | | | P07199 |  |
| CENPC | Centromere protein C | | | Q03188 |  |
| CENPH | Centromere protein H | | | Q9H3R5 |  |
| CENPK | Centromere protein K | | | Q9BS16 |  |
| CENPL | Centromere protein L | | | Q8N0S6 |  |
| CENPM | Centromere protein M | | | Q9NSP4 |  |
| CENPN | Centromere protein N | | | Q96H22 |  |
| CENPP | Centromere protein P | | | Q6IPU0 |  |
| CENPQ | Centromere protein Q | | | Q7L2Z9 |  |
| CENPT | Centromere protein T | | | Q96BT3 |  |
| CENPW | Centromere protein W | | | Q5EE01 |  |
| CEP120 | Centrosomal protein 120 | | | Q8N960 |  |
| CEP135 | Centrosomal protein 135 | | | Q66GS9 |  |
| CEP164 | Centrosomal protein 164 | | | Q9UPV0 |  |
| CEP19 | Centrosomal protein 19 | | | Q96LK0 |  |
| CEP250 | Centrosomal protein 250 | | | Q9BV73 |  |
| CEP41 | Centrosomal protein 41 | | | Q9BYV8 |  |
| CEP57 | Centrosomal protein 57 | | | Q86XR8 |  |
| CEP57L1 | Centrosomal protein 57 like 1 | | | Q8IYX8 |  |
| CEP63 | Centrosomal protein 63 | | | Q96MT8 |  |
| CEP70 | Centrosomal protein 70 | | | Q8NHQ1 |  |
| CEP72 | Centrosomal protein 72 | | | Q9P209 |  |
| CEP76 | Centrosomal protein 76 | | | Q8TAP6 |  |
| CEP83 | Centrosomal protein 83 | | | Q9Y592 |  |
| CEP85 | Centrosomal protein 85 | | | Q6P2H3 |  |
| CEP85L | Centrosomal protein 85 like | | | Q5SZL2 |  |
| CEP89 | Centrosomal protein 89 | | | Q96ST8 |  |
| CEP95 | Centrosomal protein 95 | | | Q96GE4 |  |
| CEPT1 | Choline/ethanolamine phosphotransferase 1 | | | Q9Y6K0 |  |
| CERK | Ceramide kinase | | | Q8TCT0 |  |
| CERS2 | Ceramide synthase 2 | | | Q96G23 |  |
| CERS4 | Ceramide synthase 4 | | | Q9HA82 |  |
| CES2 | Carboxylesterase 2 | | | O00748 |  |
| CES4A | Carboxylesterase 4A | | | Q5XG92 |  |
| CETN2 | Centrin 2 | | | P41208 |  |
| CETN3 | Centrin 3 | | | O15182 |  |
| CFAP20 | Cilia and flagella associated protein 20 | | | Q9Y6A4 |  |
| CFAP36 | Cilia and flagella associated protein 36 | | | Q96G28 |  |
| CFAP97 | Cilia and flagella associated protein 97 | | | Q9P2B7 |  |
| CFD | Complement factor D | | | P00746 |  |
| CFDP1 | Craniofacial development protein 1 | | | Q9UEE9 |  |
| CFH | Complement factor H | | | P08603 |  |
| CFL1 | Cofilin 1 | | | P23528 |  |
| CFL2 | Cofilin 2 | | | Q9Y281 |  |
| CFLAR | CASP8 and FADD like apoptosis regulator | | | O15519 |  |
| CFP | Complement factor properdin | | | P27918 |  |
| CGGBP1 | CGG triplet repeat binding protein 1 | | | Q9UFW8 |  |
| CGRRF1 | Cell growth regulator with ring finger domain 1 | | | Q99675 |  |
| CHAC2 | ChaC glutathione specific gamma-glutamylcyclotransferase 2 | | | Q8WUX2 |  |
| CHAF1B | Chromatin assembly factor 1 subunit B | | | Q13112 |  |
| CHAMP1 | Chromosome alignment maintaining phosphoprotein 1 | | | Q96JM3 |  |
| CHCHD1 | Coiled-coil-helix-coiled-coil-helix domain containing 1 | | | Q96BP2 |  |
| CHCHD10 | Coiled-coil-helix-coiled-coil-helix domain containing 10 | | | Q8WYQ3 |  |
| CHCHD2 | Coiled-coil-helix-coiled-coil-helix domain containing 2 | | | Q9Y6H1 |  |
| CHCHD3 | Coiled-coil-helix-coiled-coil-helix domain containing 3 | | | Q9NX63 |  |
| CHCHD4 | Coiled-coil-helix-coiled-coil-helix domain containing 4 | | | Q8N4Q1 |  |
| CHCHD5 | Coiled-coil-helix-coiled-coil-helix domain containing 5 | | | Q9BSY4 |  |
| CHCHD6 | Coiled-coil-helix-coiled-coil-helix domain containing 6 | | | Q9BRQ6 |  |
| CHCHD7 | Coiled-coil-helix-coiled-coil-helix domain containing 7 | | | Q9BUK0 |  |
| CHD1 | Chromodomain helicase DNA binding protein 1 | | | O14646 |  |
| CHD2 | Chromodomain helicase DNA binding protein 2 | | | O14647 |  |
| CHD4 | Chromodomain helicase DNA binding protein 4 | | | Q14839 |  |
| CHEK1 | Checkpoint kinase 1 | | | O14757 |  |
| CHEK2 | Checkpoint kinase 2 | | | O96017 |  |
| CHERP | Calcium homeostasis endoplasmic reticulum protein | | | Q8IWX8 |  |
| CHFR | Checkpoint with forkhead and ring finger domains | | | Q96EP1 |  |
| CHI3L2 | Chitinase 3 like 2 | | | Q15782 |  |
| CHI3L2 | Chitinase 3 like 2 | | | Q15782 |  |
| CHIC2 | Cysteine rich hydrophobic domain 2 | | | Q9UKJ5 |  |
| CHID1 | Chitinase domain containing 1 | | | Q9BWS9 |  |
| CHKB | Choline kinase beta | | | Q9Y259 |  |
| CHMP1A | Charged multivesicular body protein 1A | | | Q9HD42 |  |
| CHMP1B | Charged multivesicular body protein 1B | | | Q7LBR1 |  |
| CHMP2A | Charged multivesicular body protein 2A | | | O43633 |  |
| CHMP2B | Charged multivesicular body protein 2B | | | Q9UQN3 |  |
| CHMP3 | Charged multivesicular body protein 3 | | | Q9Y3E7 |  |
| CHMP4A | Charged multivesicular body protein 4A | | | Q9BY43 |  |
| CHMP4B | Charged multivesicular body protein 4B | | | Q9H444 |  |
| CHMP5 | Charged multivesicular body protein 5 | | | Q9NZZ3 |  |
| CHMP6 | Charged multivesicular body protein 6 | | | Q96FZ7 |  |
| CHMP7 | Charged multivesicular body protein 7 | | | Q8WUX9 |  |
| CHN1 | Chimerin 1 | | | P15882 |  |
| CHN1 | Chimerin 1 | | | P15882 |  |
| CHORDC1 | Cysteine and histidine rich domain containing 1 | | | Q9UHD1 |  |
| CHP1 | Calcineurin like EF-hand protein 1 | | | Q99653 |  |
| CHPF | Chondroitin polymerizing factor | | | Q8IZ52 |  |
| CHPF2 | Chondroitin polymerizing factor 2 | | | Q9P2E5 |  |
| CHPT1 | Choline phosphotransferase 1 | | | Q8WUD6 |  |
| CHRAC1 | Chromatin accessibility complex subunit 1 | | | Q9NRG0 |  |
| CHRNB1 | Cholinergic receptor nicotinic beta 1 subunit | | | P11230 |  |
| CHST11 | Carbohydrate sulfotransferase 11 | | | Q9NPF2 |  |
| CHST12 | Carbohydrate sulfotransferase 12 | | | Q9NRB3 |  |
| CHST14 | Carbohydrate sulfotransferase 14 | | | Q8NCH0 |  |
| CHST7 | Carbohydrate sulfotransferase 7 | | | Q9NS84 |  |
| CHTF8 | Chromosome transmission fidelity factor 8 | | | P0CG13 |  |
| CHTOP | Chromatin target of PRMT1 | | | Q9Y3Y2 |  |
| CHUK | Component of inhibitor of nuclear factor kappa B kinase complex | | | O15111 |  |
| CHURC1 | Churchill domain containing 1 | | | Q8WUH1 |  |
| CHURC1-FNTB | CHURC1-FNTB readthrough | | |  |  |
| CIAO1 | Cytosolic iron-sulfur assembly component 1 | | | O76071 |  |
| CIAPIN1 | Cytokine induced apoptosis inhibitor 1 | | | Q6FI81 |  |
| CIART | Circadian associated repressor of transcription | | | Q8N365 |  |
| CIB1 | Calcium and integrin binding 1 | | | Q99828 |  |
| CIDEB | Cell death inducing DFFA like effector b | | | Q9UHD4 |  |
| CINP | Cyclin dependent kinase 2 interacting protein | | | Q9BW66 |  |
| CIPC | CLOCK interacting pacemaker | | | Q9C0C6 |  |
| CIR1 | Corepressor interacting with RBPJ, CIR1 | | | Q86X95 |  |
| CIRBP | Cold inducible RNA binding protein | | | Q14011 |  |
| CISD1 | CDGSH iron sulfur domain 1 | | | Q9NZ45 |  |
| CISD2 | CDGSH iron sulfur domain 2 | | | Q8N5K1 |  |
| CISH | Cytokine inducible SH2 containing protein | | | Q9NSE2 |  |
| CITED2 | Cbp/p300 interacting transactivator with Glu/Asp rich carboxy-terminal domain 2 | | | Q99967 |  |
| CIZ1 | CDKN1A interacting zinc finger protein 1 | | | Q9ULV3 |  |
| CKAP2 | Cytoskeleton associated protein 2 | | | Q8WWK9 |  |
| CKAP4 | Cytoskeleton associated protein 4 | | | Q07065 |  |
| CKLF | Chemokine like factor | | | Q9UBR5 |  |
| CKS1B | CDC28 protein kinase regulatory subunit 1B | | | P61024 |  |
| CKS2 | CDC28 protein kinase regulatory subunit 2 | | | P33552 |  |
| CLASP2 | Cytoplasmic linker associated protein 2 | | | O75122 |  |
| CLASRP | CLK4 associating serine/arginine rich protein | | | Q8N2M8 |  |
| CLC | Charcot-Leyden crystal galectin | | | Q05315 |  |
| CLCC1 | Chloride channel CLIC like 1 | | | Q96S66 |  |
| CLCF1 | Cardiotrophin like cytokine factor 1 | | | Q9UBD9 |  |
| CLCN3 | Chloride voltage-gated channel 3 | | | P51790 |  |
| CLDN5 | Claudin 5 | | | O00501 |  |
| CLDND1 | Claudin domain containing 1 | | | Q9NY35 |  |
| CLEC11A | C-type lectin domain containing 11A | | | Q9Y240 |  |
| CLEC2B | C-type lectin domain family 2 member B | | | Q92478 |  |
| CLEC2D | C-type lectin domain family 2 member D | | | Q9UHP7 |  |
| CLIC1 | Chloride intracellular channel 1 | | | O00299 |  |
| CLIC3 | Chloride intracellular channel 3 | | | O95833 |  |
| CLIC5 | Chloride intracellular channel 5 | | | Q9NZA1 |  |
| CLINT1 | Clathrin interactor 1 | | | Q14677 |  |
| CLIP4 | CAP-Gly domain containing linker protein family member 4 | | | Q8N3C7 |  |
| CLK1 | CDC like kinase 1 | | | P49759 |  |
| CLK2 | CDC like kinase 2 | | | P49760 |  |
| CLK3 | CDC like kinase 3 | | | P49761 |  |
| CLK4 | CDC like kinase 4 | | | Q9HAZ1 |  |
| CLN3 | CLN3 lysosomal/endosomal transmembrane protein, battenin | | | Q13286 |  |
| CLN5 | CLN5 intracellular trafficking protein | | | O75503 |  |
| CLN6 | CLN6 transmembrane ER protein | | | Q9NWW5 |  |
| CLN8 | CLN8 transmembrane ER and ERGIC protein | | | Q9UBY8 |  |
| CLP1 | Cleavage factor polyribonucleotide kinase subunit 1 | | | Q92989 |  |
| CLPB | Caseinolytic mitochondrial matrix peptidase chaperone subunit B | | | Q9H078 |  |
| CLPP | Caseinolytic mitochondrial matrix peptidase proteolytic subunit | | | Q16740 |  |
| CLPTM1 | CLPTM1 regulator of GABA type A receptor forward trafficking | | | O96005 |  |
| CLPTM1L | CLPTM1 like | | | Q96KA5 |  |
| CLSTN1 | Calsyntenin 1 | | | O94985 |  |
| CLSTN3 | Calsyntenin 3 | | | Q9BQT9 |  |
| CLTA | Clathrin light chain A | | | P09496 |  |
| CLTB | Clathrin light chain B | | | P09497 |  |
| CLU | Clusterin | | | P10909 |  |
| CLUAP1 | Clusterin associated protein 1 | | | Q96AJ1 |  |
| CLUH | Clustered mitochondria homolog | | | O75153 |  |
| CLYBL | Citramalyl-CoA lyase | | | Q8N0X4 |  |
| CMAS | Cytidine monophosphate N-acetylneuraminic acid synthetase | | | Q8NFW8 |  |
| CMC1 | C-X9-C motif containing 1 | | | Q7Z7K0 |  |
| CMC2 | C-X9-C motif containing 2 | | | Q9NRP2 |  |
| CMC4 | C-X9-C motif containing 4 | | | P56277 |  |
| CMKLR1 | Chemerin chemokine-like receptor 1 | | | Q99788 |  |
| CMPK1 | Cytidine/uridine monophosphate kinase 1 | | | P30085 |  |
| CMSS1 | Cms1 ribosomal small subunit homolog | | | Q9BQ75 |  |
| CMTM3 | CKLF like MARVEL transmembrane domain containing 3 | | | Q96MX0 |  |
| CMTM6 | CKLF like MARVEL transmembrane domain containing 6 | | | Q9NX76 |  |
| CMTM7 | CKLF like MARVEL transmembrane domain containing 7 | | | Q96FZ5 |  |
| CMTR1 | Cap methyltransferase 1 | | | Q8N1G2 |  |
| CMTR2 | Cap methyltransferase 2 | | | Q8IYT2 |  |
| CNBP | CCHC-type zinc finger nucleic acid binding protein | | | P62633 |  |
| CNDP2 | Carnosine dipeptidase 2 | | | Q96KP4 |  |
| CNEP1R1 | CTD nuclear envelope phosphatase 1 regulatory subunit 1 | | | Q8N9A8 |  |
| CNFN | Cornifelin | | | Q9BYD5 |  |
| CNIH1 | Cornichon family AMPA receptor auxiliary protein 1 | | | O95406 |  |
| CNIH4 | Cornichon family AMPA receptor auxiliary protein 4 | | | Q9P003 |  |
| CNN2 | Calponin 2 | | | Q99439 |  |
| CNN3 | Calponin 3 | | | Q15417 |  |
| CNN3 | Calponin 3 | | | Q15417 |  |
| CNNM3 | Cyclin and CBS domain divalent metal cation transport mediator 3 | | | Q8NE01 |  |
| CNOT10 | CCR4-NOT transcription complex subunit 10 | | | Q9H9A5 |  |
| CNOT11 | CCR4-NOT transcription complex subunit 11 | | | Q9UKZ1 |  |
| CNOT2 | CCR4-NOT transcription complex subunit 2 | | | Q9NZN8 |  |
| CNOT3 | CCR4-NOT transcription complex subunit 3 | | | O75175 |  |
| CNOT4 | CCR4-NOT transcription complex subunit 4 | | | O95628 |  |
| CNOT6 | CCR4-NOT transcription complex subunit 6 | | | Q9ULM6 |  |
| CNOT6L | CCR4-NOT transcription complex subunit 6 like | | | Q96LI5 |  |
| CNOT7 | CCR4-NOT transcription complex subunit 7 | | | Q9UIV1 |  |
| CNOT8 | CCR4-NOT transcription complex subunit 8 | | | Q9UFF9 |  |
| CNP | 2',3'-cyclic nucleotide 3' phosphodiesterase | | | P09543 |  |
| CNPPD1 | Cyclin Pas1/PHO80 domain containing 1 | | | Q9BV87 |  |
| CNPY2 | Canopy FGF signaling regulator 2 | | | Q9Y2B0 |  |
| CNPY3 | Canopy FGF signaling regulator 3 | | | Q9BT09 |  |
| CNPY4 | Canopy FGF signaling regulator 4 | | | Q8N129 |  |
| CNST | Consortin, connexin sorting protein | | | Q6PJW8 |  |
| CNTROB | Centrobin, centriole duplication and spindle assembly protein | | | Q8N137 |  |
| COA1 | Cytochrome c oxidase assembly factor 1 | | | Q9GZY4 |  |
| COA3 | Cytochrome c oxidase assembly factor 3 | | | Q9Y2R0 |  |
| COA4 | Cytochrome c oxidase assembly factor 4 homolog | | | Q9NYJ1 |  |
| COA5 | Cytochrome c oxidase assembly factor 5 | | | Q86WW8 |  |
| COA6 | Cytochrome c oxidase assembly factor 6 | | | Q5JTJ3 |  |
| COA7 | Cytochrome c oxidase assembly factor 7 | | | Q96BR5 |  |
| COASY | Coenzyme A synthase | | | Q13057 |  |
| COG1 | Component of oligomeric golgi complex 1 | | | Q8WTW3 |  |
| COG2 | Component of oligomeric golgi complex 2 | | | Q14746 |  |
| COG3 | Component of oligomeric golgi complex 3 | | | Q96JB2 |  |
| COG4 | Component of oligomeric golgi complex 4 | | |  |  |
| COG5 | Component of oligomeric golgi complex 5 | | | Q9UP83 |  |
| COG6 | Component of oligomeric golgi complex 6 | | | Q9Y2V7 |  |
| COG7 | Component of oligomeric golgi complex 7 | | | P83436 |  |
| COIL | Coilin | | | P38432 |  |
| COLGALT1 | Collagen beta(1-O)galactosyltransferase 1 | | | Q8NBJ5 |  |
| COMMD1 | Copper metabolism domain containing 1 | | | Q8N668 |  |
| COMMD10 | COMM domain containing 10 | | | Q9Y6G5 |  |
| COMMD2 | COMM domain containing 2 | | | Q86X83 |  |
| COMMD3 | COMM domain containing 3 | | | Q9UBI1 |  |
| COMMD4 | COMM domain containing 4 | | | Q9H0A8 |  |
| COMMD5 | COMM domain containing 5 | | | Q9GZQ3 |  |
| COMMD6 | COMM domain containing 6 | | | Q7Z4G1 |  |
| COMMD7 | COMM domain containing 7 | | | Q86VX2 |  |
| COMMD8 | COMM domain containing 8 | | | Q9NX08 |  |
| COMMD9 | COMM domain containing 9 | | | Q9P000 |  |
| COMT | Catechol-O-methyltransferase | | | P21964 |  |
| COMTD1 | Catechol-O-methyltransferase domain containing 1 | | | Q86VU5 |  |
| COPB1 | COPI coat complex subunit beta 1 | | | P53618 |  |
| COPB2 | COPI coat complex subunit beta 2 | | | P35606 |  |
| COPE | COPI coat complex subunit epsilon | | | O14579 |  |
| COPG1 | COPI coat complex subunit gamma 1 | | | Q9Y678 |  |
| COPS2 | COP9 signalosome subunit 2 | | | P61201 |  |
| COPS3 | COP9 signalosome subunit 3 | | | Q9UNS2 |  |
| COPS5 | COP9 signalosome subunit 5 | | | Q92905 |  |
| COPS6 | COP9 signalosome subunit 6 | | | Q7L5N1 |  |
| COPS7A | COP9 signalosome subunit 7A | | | Q9UBW8 |  |
| COPS7B | COP9 signalosome subunit 7B | | | Q9H9Q2 |  |
| COPS8 | COP9 signalosome subunit 8 | | | Q99627 |  |
| COPZ1 | COPI coat complex subunit zeta 1 | | | P61923 |  |
| COQ10A | Coenzyme Q10A | | | Q96MF6 |  |
| COQ10B | Coenzyme Q10B | | | Q9H8M1 |  |
| COQ2 | Coenzyme Q2, polyprenyltransferase | | | Q96H96 |  |
| COQ3 | Coenzyme Q3, methyltransferase | | | Q9NZJ6 |  |
| COQ4 | Coenzyme Q4 | | | Q9Y3A0 |  |
| COQ5 | Coenzyme Q5, methyltransferase | | | Q5HYK3 |  |
| COQ6 | Coenzyme Q6, monooxygenase | | | Q9Y2Z9 |  |
| COQ7 | Coenzyme Q7, hydroxylase | | | Q99807 |  |
| COQ9 | Coenzyme Q9 | | | O75208 |  |
| CORO1A | Coronin 1A | | | P31146 |  |
| CORO1B | Coronin 1B | | | Q9BR76 |  |
| CORO1C | Coronin 1C | | | Q9ULV4 |  |
| CORO2A | Coronin 2A | | | Q92828 |  |
| CORO7 | Coronin 7 | | | P57737 |  |
| COTL1 | Coactosin like F-actin binding protein 1 | | | Q14019 |  |
| COX10 | Cytochrome c oxidase assembly factor heme A:farnesyltransferase COX10 | | | Q12887 |  |
| COX11 | Cytochrome c oxidase copper chaperone COX11 | | | Q9Y6N1 |  |
| COX14 | Cytochrome c oxidase assembly factor COX14 | | | Q96I36 |  |
| COX15 | Cytochrome c oxidase assembly homolog COX15 | | | Q7KZN9 |  |
| COX16 | Cytochrome c oxidase assembly factor COX16 | | | Q9P0S2 |  |
| COX17 | Cytochrome c oxidase copper chaperone COX17 | | | Q14061 |  |
| COX19 | Cytochrome c oxidase assembly factor COX19 | | | Q49B96 |  |
| COX20 | Cytochrome c oxidase assembly factor COX20 | | | Q5RI15 |  |
| COX4I1 | Cytochrome c oxidase subunit 4I1 | | | P13073 |  |
| COX5A | Cytochrome c oxidase subunit 5A | | | P20674 |  |
| COX5B | Cytochrome c oxidase subunit 5B | | | P10606 |  |
| COX6A1 | Cytochrome c oxidase subunit 6A1 | | | P12074 |  |
| COX6B1 | Cytochrome c oxidase subunit 6B1 | | | P14854 |  |
| COX6C | Cytochrome c oxidase subunit 6C | | | P09669 |  |
| COX7A2 | Cytochrome c oxidase subunit 7A2 | | | P14406 |  |
| COX7A2L | Cytochrome c oxidase subunit 7A2 like | | | O14548 |  |
| COX7B | Cytochrome c oxidase subunit 7B | | | P24311 |  |
| COX7C | Cytochrome c oxidase subunit 7C | | | P15954 |  |
| COX8A | Cytochrome c oxidase subunit 8A | | | P10176 |  |
| CPA5 | Carboxypeptidase A5 | | | Q8WXQ8 |  |
| CPA5 | Carboxypeptidase A5 | | | Q8WXQ8 |  |
| CPD | Carboxypeptidase D | | | O75976 |  |
| CPM | Carboxypeptidase M | | | P14384 |  |
| CPNE1 | Copine 1 | | | Q99829 |  |
| CPNE3 | Copine 3 | | | O75131 |  |
| CPNE7 | Copine 7 | | | Q9UBL6 |  |
| CPNE8 | Copine 8 | | | Q86YQ8 |  |
| CPPED1 | Calcineurin like phosphoesterase domain containing 1 | | | Q9BRF8 |  |
| CPQ | Carboxypeptidase Q | | | Q9Y646 |  |
| CPSF1 | Cleavage and polyadenylation specific factor 1 | | | Q10570 |  |
| CPSF2 | Cleavage and polyadenylation specific factor 2 | | | Q9P2I0 |  |
| CPSF3 | Cleavage and polyadenylation specific factor 3 | | | Q9UKF6 |  |
| CPSF4 | Cleavage and polyadenylation specific factor 4 | | | O95639 |  |
| CPSF6 | Cleavage and polyadenylation specific factor 6 | | | Q16630 |  |
| CPSF7 | Cleavage and polyadenylation specific factor 7 | | | Q8N684 |  |
| CPT1A | Carnitine palmitoyltransferase 1A | | | P50416 |  |
| CPT2 | Carnitine palmitoyltransferase 2 | | | P23786 |  |
| CPVL | Carboxypeptidase vitellogenic like | | | Q9H3G5 |  |
| CRACR2A | Calcium release activated channel regulator 2A | | | Q9BSW2 |  |
| CRADD | CASP2 and RIPK1 domain containing adaptor with death domain | | | P78560 |  |
| CRAT | Carnitine O-acetyltransferase | | | P43155 |  |
| CRB3 | Crumbs cell polarity complex component 3 | | | Q9BUF7 |  |
| CRB3 | Crumbs cell polarity complex component 3 | | | Q9BUF7 |  |
| CRBN | Cereblon | | | Q96SW2 |  |
| CRCP | CGRP receptor component | | | O75575 |  |
| CREB1 | CAMP responsive element binding protein 1 | | | P16220 |  |
| CREB3 | CAMP responsive element binding protein 3 | | | O43889 |  |
| CREB3L2 | CAMP responsive element binding protein 3 like 2 | | | Q70SY1 |  |
| CREB3L4 | CAMP responsive element binding protein 3 like 4 | | | Q8TEY5 |  |
| CREBL2 | CAMP responsive element binding protein like 2 | | | O60519 |  |
| CREBRF | CREB3 regulatory factor | | | Q8IUR6 |  |
| CREG1 | Cellular repressor of E1A stimulated genes 1 | | | O75629 |  |
| CRELD1 | Cysteine rich with EGF like domains 1 | | | Q96HD1 |  |
| CRELD2 | Cysteine rich with EGF like domains 2 | | | Q6UXH1 |  |
| CREM | CAMP responsive element modulator | | | Q03060 |  |
| CRIP1 | Cysteine rich protein 1 | | | P50238 |  |
| CRIP2 | Cysteine rich protein 2 | | | P52943 |  |
| CRIP2 | Cysteine rich protein 2 | | | P52943 |  |
| CRIPT | CXXC repeat containing interactor of PDZ3 domain | | | Q9P021 |  |
| CRK | CRK proto-oncogene, adaptor protein | | | P46108 |  |
| CRKL | CRK like proto-oncogene, adaptor protein | | | P46109 |  |
| CRLF3 | Cytokine receptor like factor 3 | | | Q8IUI8 |  |
| CRNKL1 | Crooked neck pre-mRNA splicing factor 1 | | | Q9BZJ0 |  |
| CROT | Carnitine O-octanoyltransferase | | | Q9UKG9 |  |
| CRTAM | Cytotoxic and regulatory T cell molecule | | | O95727 |  |
| CRTAP | Cartilage associated protein | | | O75718 |  |
| CRTC2 | CREB regulated transcription coactivator 2 | | | Q53ET0 |  |
| CRTC3 | CREB regulated transcription coactivator 3 | | | Q6UUV7 |  |
| CRY1 | Cryptochrome circadian regulator 1 | | | Q16526 |  |
| CRY2 | Cryptochrome circadian regulator 2 | | | Q49AN0 |  |
| CRYGN | Crystallin gamma N | | | Q8WXF5 |  |
| CRYL1 | Crystallin lambda 1 | | | Q9Y2S2 |  |
| CRYZ | Crystallin zeta | | | Q08257 |  |
| CRYZL1 | Crystallin zeta like 1 | | | O95825 |  |
| CS | Citrate synthase | | | O75390 |  |
| CSAD | Cysteine sulfinic acid decarboxylase | | | Q9Y600 |  |
| CSDE1 | Cold shock domain containing E1 | | | O75534 |  |
| CSE1L | Chromosome segregation 1 like | | | P55060 |  |
| CSF2RA | Colony stimulating factor 2 receptor subunit alpha | | | P15509 |  |
| CSGALNACT1 | Chondroitin sulfate N-acetylgalactosaminyltransferase 1 | | | Q8TDX6 |  |
| CSGALNACT2 | Chondroitin sulfate N-acetylgalactosaminyltransferase 2 | | | Q8N6G5 |  |
| CSK | C-terminal Src kinase | | | P41240 |  |
| CSNK1A1 | Casein kinase 1 alpha 1 | | | P48729 |  |
| CSNK1D | Casein kinase 1 delta | | | P48730 |  |
| CSNK1E | Casein kinase 1 epsilon | | | P49674 |  |
| CSNK1G1 | Casein kinase 1 gamma 1 | | | Q9HCP0 |  |
| CSNK1G2 | Casein kinase 1 gamma 2 | | | P78368 |  |
| CSNK1G3 | Casein kinase 1 gamma 3 | | | Q9Y6M4 |  |
| CSNK2A1 | Casein kinase 2 alpha 1 | | | P68400 |  |
| CSNK2A2 | Casein kinase 2 alpha 2 | | | P19784 |  |
| CSNK2B | Casein kinase 2 beta | | | P67870 |  |
| CSRP1 | Cysteine and glycine rich protein 1 | | | P21291 |  |
| CST3 | Cystatin C | | | P01034 |  |
| CST7 | Cystatin F | | | O76096 |  |
| CSTB | Cystatin B | | | P04080 |  |
| CSTF1 | Cleavage stimulation factor subunit 1 | | | Q05048 |  |
| CSTF2 | Cleavage stimulation factor subunit 2 | | | P33240 |  |
| CSTF2T | Cleavage stimulation factor subunit 2 tau variant | | | Q9H0L4 |  |
| CSTF3 | Cleavage stimulation factor subunit 3 | | | Q12996 |  |
| CTBP1 | C-terminal binding protein 1 | | | Q13363 |  |
| CTC1 | CST telomere replication complex component 1 | | | Q2NKJ3 |  |
| CTCF | CCCTC-binding factor | | | P49711 |  |
| CTDNEP1 | CTD nuclear envelope phosphatase 1 | | | O95476 |  |
| CTDP1 | CTD phosphatase subunit 1 | | | Q9Y5B0 |  |
| CTDSP1 | CTD small phosphatase 1 | | | Q9GZU7 |  |
| CTDSP2 | CTD small phosphatase 2 | | | O14595 |  |
| CTDSPL2 | CTD small phosphatase like 2 | | | Q05D32 |  |
| CTH | Cystathionine gamma-lyase | | | P32929 |  |
| CTLA4 | Cytotoxic T-lymphocyte associated protein 4 | | | P16410 |  |
| CTNNA1 | Catenin alpha 1 | | | P35221 |  |
| CTNNAL1 | Catenin alpha like 1 | | | Q9UBT7 |  |
| CTNNB1 | Catenin beta 1 | | | P35222 |  |
| CTNNBIP1 | Catenin beta interacting protein 1 | | | Q9NSA3 |  |
| CTNS | Cystinosin, lysosomal cystine transporter | | | O60931 |  |
| CTR9 | CTR9 homolog, Paf1/RNA polymerase II complex component | | | Q6PD62 |  |
| CTSA | Cathepsin A | | | P10619 |  |
| CTSB | Cathepsin B | | | P07858 |  |
| CTSC | Cathepsin C | | | P53634 |  |
| CTSD | Cathepsin D | | | P07339 |  |
| CTSF | Cathepsin F | | | Q9UBX1 |  |
| CTSH | Cathepsin H | | | P09668 |  |
| CTSL | Cathepsin L | | | P07711 |  |
| CTSO | Cathepsin O | | | P43234 |  |
| CTSS | Cathepsin S | | | P25774 |  |
| CTSW | Cathepsin W | | | P56202 |  |
| CUL1 | Cullin 1 | | | Q13616 |  |
| CUL2 | Cullin 2 | | | Q13617 |  |
| CUL3 | Cullin 3 | | | Q13618 |  |
| CUL4A | Cullin 4A | | | Q13619 |  |
| CUL4B | Cullin 4B | | | Q13620 |  |
| CUL5 | Cullin 5 | | | Q93034 |  |
| CUTA | CutA divalent cation tolerance homolog | | | O60888 |  |
| CUTC | CutC copper transporter | | | Q9NTM9 |  |
| CUX1 | Cut like homeobox 1 | | | P39880, Q13948 |  |
| CWC15 | CWC15 spliceosome associated protein homolog | | | Q9P013 |  |
| CWC22 | CWC22 spliceosome associated protein homolog | | | Q9HCG8 |  |
| CWC25 | CWC25 spliceosome associated protein homolog | | | Q9NXE8 |  |
| CWF19L1 | CWF19 like cell cycle control factor 1 | | | Q69YN2 |  |
| CWF19L2 | CWF19 like cell cycle control factor 2 | | | Q2TBE0 |  |
| CX3CR1 | C-X3-C motif chemokine receptor 1 | | | P49238 |  |
| CXCL16 | C-X-C motif chemokine ligand 16 | | | Q9H2A7 |  |
| CXCR1 | C-X-C motif chemokine receptor 1 | | | P25024 |  |
| CXCR2 | C-X-C motif chemokine receptor 2 | | | P25025 |  |
| CXCR3 | C-X-C motif chemokine receptor 3 | | | P49682 |  |
| CXCR4 | C-X-C motif chemokine receptor 4 | | | P61073 |  |
| CXCR5 | C-X-C motif chemokine receptor 5 | | | P32302 |  |
| CXCR6 | C-X-C motif chemokine receptor 6 | | | O00574 |  |
| CXorf38 | Chromosome X open reading frame 38 | | | Q8TB03 |  |
| CXXC1 | CXXC finger protein 1 | | | Q9P0U4 |  |
| CYB561 | Cytochrome b561 | | | P49447 |  |
| CYB561A3 | Cytochrome b561 family member A3 | | | Q8NBI2 |  |
| CYB561D1 | Cytochrome b561 family member D1 | | | Q8N8Q1 |  |
| CYB561D2 | Cytochrome b561 family member D2 | | | O14569 |  |
| CYB5A | Cytochrome b5 type A | | | P00167 |  |
| CYB5B | Cytochrome b5 type B | | | O43169 |  |
| CYB5D1 | Cytochrome b5 domain containing 1 | | | Q6P9G0 |  |
| CYB5D2 | Cytochrome b5 domain containing 2 | | | Q8WUJ1 |  |
| CYB5R1 | Cytochrome b5 reductase 1 | | | Q9UHQ9 |  |
| CYB5R3 | Cytochrome b5 reductase 3 | | | P00387 |  |
| CYB5R4 | Cytochrome b5 reductase 4 | | | Q7L1T6 |  |
| CYBA | Cytochrome b-245 alpha chain | | | P13498 |  |
| CYC1 | Cytochrome c1 | | | P08574 |  |
| CYCS | Cytochrome c, somatic | | | P99999 |  |
| CYFIP1 | Cytoplasmic FMR1 interacting protein 1 | | | Q7L576 |  |
| CYFIP2 | Cytoplasmic FMR1 interacting protein 2 | | | Q96F07 |  |
| CYP20A1 | Cytochrome P450 family 20 subfamily A member 1 | | | Q6UW02 |  |
| CYP2E1 | Cytochrome P450 family 2 subfamily E member 1 | | | P05181 |  |
| CYP2J2 | Cytochrome P450 family 2 subfamily J member 2 | | | P51589 |  |
| CYP2R1 | Cytochrome P450 family 2 subfamily R member 1 | | | Q6VVX0 |  |
| CYP4V2 | Cytochrome P450 family 4 subfamily V member 2 | | | Q6ZWL3 |  |
| CYP51A1 | Cytochrome P450 family 51 subfamily A member 1 | | | Q16850 |  |
| CYSLTR1 | Cysteinyl leukotriene receptor 1 | | | Q9Y271 |  |
| CYSTM1 | Cysteine rich transmembrane module containing 1 | | | Q9H1C7 |  |
| CYTH1 | Cytohesin 1 | | | Q15438 |  |
| CYTH2 | Cytohesin 2 | | | Q99418 |  |
| CYTH4 | Cytohesin 4 | | | Q9UIA0 |  |
| CYTIP | Cytohesin 1 interacting protein | | | O60759 |  |
| DAAM1 | Dishevelled associated activator of morphogenesis 1 | | | Q9Y4D1 |  |
| DAD1 | Defender against cell death 1 | | | P61803 |  |
| DAGLB | Diacylglycerol lipase beta | | | Q8NCG7 |  |
| DALRD3 | DALR anticodon binding domain containing 3 | | | Q5D0E6 |  |
| DAP | Death associated protein | | | P51397 |  |
| DAP3 | Death associated protein 3 | | | P51398 |  |
| DAPK3 | Death associated protein kinase 3 | | | O43293 |  |
| DAPP1 | Dual adaptor of phosphotyrosine and 3-phosphoinositides 1 | | | Q9UN19 |  |
| DARS2 | Aspartyl-tRNA synthetase 2, mitochondrial | | | Q6PI48 |  |
| DAXX | Death domain associated protein | | | Q9UER7 |  |
| DAZAP1 | DAZ associated protein 1 | | | Q96EP5 |  |
| DAZAP2 | DAZ associated protein 2 | | | Q15038 |  |
| DBI | Diazepam binding inhibitor, acyl-CoA binding protein | | | P07108 |  |
| DBNDD1 | Dysbindin domain containing 1 | | | Q9H9R9 |  |
| DBNDD2 | Dysbindin domain containing 2 | | | Q9BQY9 |  |
| DBNL | Drebrin like | | | Q9UJU6 |  |
| DBP | D-box binding PAR bZIP transcription factor | | | Q10586 |  |
| DBR1 | Debranching RNA lariats 1 | | | Q9UK59 |  |
| DBT | Dihydrolipoamide branched chain transacylase E2 | | | P11182 |  |
| DCAF11 | DDB1 and CUL4 associated factor 11 | | | Q8TEB1 |  |
| DCAF12 | DDB1 and CUL4 associated factor 12 | | | Q5T6F0 |  |
| DCAF4 | DDB1 and CUL4 associated factor 4 | | | Q8WV16 |  |
| DCAF7 | DDB1 and CUL4 associated factor 7 | | | P61962 |  |
| DCAF8 | DDB1 and CUL4 associated factor 8 | | | Q5TAQ9 |  |
| DCAKD | Dephospho-CoA kinase domain containing | | | Q8WVC6 |  |
| DCK | Deoxycytidine kinase | | | P27707 |  |
| DCLRE1A | DNA cross-link repair 1A | | | Q6PJP8 |  |
| DCLRE1B | DNA cross-link repair 1B | | | Q9H816 |  |
| DCLRE1C | DNA cross-link repair 1C | | | Q96SD1 |  |
| DCP1A | Decapping mRNA 1A | | | Q9NPI6 |  |
| DCP1B | Decapping mRNA 1B | | | Q8IZD4 |  |
| DCP2 | Decapping mRNA 2 | | | Q8IU60 |  |
| DCPS | Decapping enzyme, scavenger | | | Q96C86 |  |
| DCTD | DCMP deaminase | | | P32321 |  |
| DCTN1 | Dynactin subunit 1 | | | Q14203 |  |
| DCTN2 | Dynactin subunit 2 | | | Q13561 |  |
| DCTN3 | Dynactin subunit 3 | | | O75935 |  |
| DCTN4 | Dynactin subunit 4 | | | Q9UJW0 |  |
| DCTN6 | Dynactin subunit 6 | | | O00399 |  |
| DCTPP1 | DCTP pyrophosphatase 1 | | | Q9H773 |  |
| DCUN1D1 | Defective in cullin neddylation 1 domain containing 1 | | | Q96GG9 |  |
| DCUN1D2 | Defective in cullin neddylation 1 domain containing 2 | | | Q6PH85 |  |
| DCUN1D4 | Defective in cullin neddylation 1 domain containing 4 | | | Q92564 |  |
| DCUN1D5 | Defective in cullin neddylation 1 domain containing 5 | | | Q9BTE7 |  |
| DCXR | Dicarbonyl and L-xylulose reductase | | | Q7Z4W1 |  |
| DDA1 | DET1 and DDB1 associated 1 | | | Q9BW61 |  |
| DDAH2 | Dimethylarginine dimethylaminohydrolase 2 | | | O95865 |  |
| DDB1 | Damage specific DNA binding protein 1 | | | Q16531 |  |
| DDB2 | Damage specific DNA binding protein 2 | | | Q92466 |  |
| DDHD1 | DDHD domain containing 1 | | | Q8NEL9 |  |
| DDHD2 | DDHD domain containing 2 | | | O94830 |  |
| DDIT3 | DNA damage inducible transcript 3 | | | P35638 |  |
| DDIT4 | DNA damage inducible transcript 4 | | | Q9NX09 |  |
| DDOST | Dolichyl-diphosphooligosaccharide--protein glycosyltransferase non-catalytic subunit | | | P39656 |  |
| DDRGK1 | DDRGK domain containing 1 | | | Q96HY6 |  |
| DDT | D-dopachrome tautomerase | | | P30046 |  |
| DDX1 | DEAD-box helicase 1 | | | Q92499 |  |
| DDX11 | DEAD/H-box helicase 11 | | | Q96FC9 |  |
| DDX17 | DEAD-box helicase 17 | | | Q92841 |  |
| DDX18 | DEAD-box helicase 18 | | | Q9NVP1 |  |
| DDX19B | DEAD-box helicase 19B | | | Q9UMR2 |  |
| DDX20 | DEAD-box helicase 20 | | | Q9UHI6 |  |
| DDX21 | DExD-box helicase 21 | | | Q9NR30 |  |
| DDX23 | DEAD-box helicase 23 | | | Q9BUQ8 |  |
| DDX24 | DEAD-box helicase 24 | | | Q9GZR7 |  |
| DDX27 | DEAD-box helicase 27 | | |  |  |
| DDX28 | DEAD-box helicase 28 | | | Q9NUL7 |  |
| DDX31 | DEAD-box helicase 31 | | | Q9H8H2 |  |
| DDX39A | DExD-box helicase 39A | | | O00148 |  |
| DDX39B | DExD-box helicase 39B | | | Q13838 |  |
| DDX3X | DEAD-box helicase 3 X-linked | | | O00571 |  |
| DDX3Y | DEAD-box helicase 3 Y-linked | | | O15523 |  |
| DDX41 | DEAD-box helicase 41 | | | Q9UJV9 |  |
| DDX42 | DEAD-box helicase 42 | | | Q86XP3 |  |
| DDX46 | DEAD-box helicase 46 | | | Q7L014 |  |
| DDX47 | DEAD-box helicase 47 | | | Q9H0S4 |  |
| DDX49 | DEAD-box helicase 49 | | | Q9Y6V7 |  |
| DDX5 | DEAD-box helicase 5 | | | P17844 |  |
| DDX50 | DExD-box helicase 50 | | | Q9BQ39 |  |
| DDX54 | DEAD-box helicase 54 | | | Q8TDD1 |  |
| DDX55 | DEAD-box helicase 55 | | | Q8NHQ9 |  |
| DDX56 | DEAD-box helicase 56 | | | Q9NY93 |  |
| DDX59 | DEAD-box helicase 59 | | | Q5T1V6 |  |
| DDX6 | DEAD-box helicase 6 | | | P26196 |  |
| DDX60 | DExD/H-box helicase 60 | | | Q8IY21 |  |
| DEAF1 | DEAF1 transcription factor | | | O75398 |  |
| DECR1 | 2,4-dienoyl-CoA reductase 1 | | | Q16698 |  |
| DECR2 | 2,4-dienoyl-CoA reductase 2 | | | Q9NUI1 |  |
| DEDD | Death effector domain containing | | | O75618 |  |
| DEDD2 | Death effector domain containing 2 | | | Q8WXF8 |  |
| DEF6 | DEF6 guanine nucleotide exchange factor | | | Q9H4E7 |  |
| DEFA1 | Defensin alpha 1 | | | P59665 |  |
| DEFA3 | Defensin alpha 3 | | | P59666 |  |
| DEGS1 | Delta 4-desaturase, sphingolipid 1 | | | O15121 |  |
| DEK | DEK proto-oncogene | | | P35659 |  |
| DENND1A | DENN domain containing 1A | | | Q8TEH3 |  |
| DENND1C | DENN domain containing 1C | | | Q8IV53 |  |
| DENND2D | DENN domain containing 2D | | | Q9H6A0 |  |
| DENND5A | DENN domain containing 5A | | | Q6IQ26 |  |
| DENND6A | DENN domain containing 6A | | | Q8IWF6 |  |
| DENR | Density regulated re-initiation and release factor | | | O43583 |  |
| DEPDC5 | DEP domain containing 5, GATOR1 subcomplex subunit | | | O75140 |  |
| DEPDC7 | DEP domain containing 7 | | | Q96QD5 |  |
| DERA | Deoxyribose-phosphate aldolase | | | Q9Y315 |  |
| DERL1 | Derlin 1 | | | Q9BUN8 |  |
| DERL2 | Derlin 2 | | | Q9GZP9 |  |
| DESI1 | Desumoylating isopeptidase 1 | | | Q6ICB0 |  |
| DESI2 | Desumoylating isopeptidase 2 | | | Q9BSY9 |  |
| DEXI | Dexi homolog | | | O95424 |  |
| DFFA | DNA fragmentation factor subunit alpha | | | O00273 |  |
| DFFB | DNA fragmentation factor subunit beta | | | O76075 |  |
| DGCR2 | DiGeorge syndrome critical region gene 2 | | | P98153 |  |
| DGCR6 | DiGeorge syndrome critical region gene 6 | | | Q14129 |  |
| DGCR6L | DiGeorge syndrome critical region gene 6 like | | | Q9BY27 |  |
| DGCR8 | DGCR8 microprocessor complex subunit | | | Q8WYQ5 |  |
| DGKA | Diacylglycerol kinase alpha | | | P23743 |  |
| DGKD | Diacylglycerol kinase delta | | | Q16760 |  |
| DGKZ | Diacylglycerol kinase zeta | | | Q13574 |  |
| DGUOK | Deoxyguanosine kinase | | | Q16854 |  |
| DHCR24 | 24-dehydrocholesterol reductase | | | Q15392 |  |
| DHCR7 | 7-dehydrocholesterol reductase | | | Q9UBM7 |  |
| DHDDS | Dehydrodolichyl diphosphate synthase subunit | | | Q86SQ9 |  |
| DHDH | Dihydrodiol dehydrogenase | | | Q9UQ10 |  |
| DHODH | Dihydroorotate dehydrogenase (quinone) | | | Q02127 |  |
| DHPS | Deoxyhypusine synthase | | | P49366 |  |
| DHRS1 | Dehydrogenase/reductase 1 | | | Q96LJ7 |  |
| DHRS12 | Dehydrogenase/reductase 12 | | | A0PJE2 |  |
| DHRS13 | Dehydrogenase/reductase 13 | | | Q6UX07 |  |
| DHRS3 | Dehydrogenase/reductase 3 | | | O75911 |  |
| DHRS4 | Dehydrogenase/reductase 4 | | | Q9BTZ2 |  |
| DHRS4L2 | Dehydrogenase/reductase 4 like 2 | | | Q6PKH6 |  |
| DHRS7 | Dehydrogenase/reductase 7 | | | Q9Y394 |  |
| DHRS7B | Dehydrogenase/reductase 7B | | | Q6IAN0 |  |
| DHRSX | Dehydrogenase/reductase X-linked | | | Q8N5I4 |  |
| DHTKD1 | Dehydrogenase E1 and transketolase domain containing 1 | | | Q96HY7 |  |
| DHX16 | DEAH-box helicase 16 | | | O60231 |  |
| DHX29 | DExH-box helicase 29 | | | Q7Z478 |  |
| DHX30 | DExH-box helicase 30 | | | Q7L2E3 |  |
| DHX32 | DEAH-box helicase 32 (putative) | | | Q7L7V1 |  |
| DHX34 | DExH-box helicase 34 | | | Q14147 |  |
| DHX35 | DEAH-box helicase 35 | | | Q9H5Z1 |  |
| DHX36 | DEAH-box helicase 36 | | | Q9H2U1 |  |
| DHX38 | DEAH-box helicase 38 | | | Q92620 |  |
| DHX40 | DEAH-box helicase 40 | | | Q8IX18 |  |
| DHX58 | DExH-box helicase 58 | | | Q96C10 |  |
| DHX8 | DEAH-box helicase 8 | | | Q14562 |  |
| DIABLO | Diablo IAP-binding mitochondrial protein | | | Q9NR28 |  |
| DIDO1 | Death inducer-obliterator 1 | | | Q9BTC0 |  |
| DIMT1 | DIM1 rRNA methyltransferase and ribosome maturation factor | | | Q9UNQ2 |  |
| DIP2A | Disco interacting protein 2 homolog A | | | Q14689 |  |
| DIS3 | DIS3 homolog, exosome endoribonuclease and 3'-5' exoribonuclease | | | Q9Y2L1 |  |
| DIS3L | DIS3 like exosome 3'-5' exoribonuclease | | | Q8TF46 |  |
| DIS3L2 | DIS3 like 3'-5' exoribonuclease 2 | | | Q8IYB7 |  |
| DKC1 | Dyskerin pseudouridine synthase 1 | | | O60832 |  |
| DLAT | Dihydrolipoamide S-acetyltransferase | | | P10515 |  |
| DLD | Dihydrolipoamide dehydrogenase | | | P09622 |  |
| DLGAP4 | DLG associated protein 4 | | | Q9Y2H0 |  |
| DLGAP5 | DLG associated protein 5 | | | Q15398 |  |
| DLST | Dihydrolipoamide S-succinyltransferase | | | P36957 |  |
| DMAP1 | DNA methyltransferase 1 associated protein 1 | | | Q9NPF5 |  |
| DMTF1 | Cyclin D binding myb like transcription factor 1 | | | Q9Y222 |  |
| DMTN | Dematin actin binding protein | | | Q08495 |  |
| DMWD | DM1 locus, WD repeat containing | | | Q09019 |  |
| DNAJA1 | DnaJ heat shock protein family (Hsp40) member A1 | | | P31689 |  |
| DNAJA3 | DnaJ heat shock protein family (Hsp40) member A3 | | | Q96EY1 |  |
| DNAJA4 | DnaJ heat shock protein family (Hsp40) member A4 | | | Q8WW22 |  |
| DNAJB1 | DnaJ heat shock protein family (Hsp40) member B1 | | | P25685 |  |
| DNAJB11 | DnaJ heat shock protein family (Hsp40) member B11 | | | Q9UBS4 |  |
| DNAJB12 | DnaJ heat shock protein family (Hsp40) member B12 | | | Q9NXW2 |  |
| DNAJB14 | DnaJ heat shock protein family (Hsp40) member B14 | | | Q8TBM8 |  |
| DNAJB2 | DnaJ heat shock protein family (Hsp40) member B2 | | | P25686 |  |
| DNAJB4 | DnaJ heat shock protein family (Hsp40) member B4 | | | Q9UDY4 |  |
| DNAJB6 | DnaJ heat shock protein family (Hsp40) member B6 | | | O75190 |  |
| DNAJB9 | DnaJ heat shock protein family (Hsp40) member B9 | | | Q9UBS3 |  |
| DNAJC10 | DnaJ heat shock protein family (Hsp40) member C10 | | | Q8IXB1 |  |
| DNAJC11 | DnaJ heat shock protein family (Hsp40) member C11 | | | Q9NVH1 |  |
| DNAJC14 | DnaJ heat shock protein family (Hsp40) member C14 | | | Q6Y2X3 |  |
| DNAJC15 | DnaJ heat shock protein family (Hsp40) member C15 | | | Q9Y5T4 |  |
| DNAJC16 | DnaJ heat shock protein family (Hsp40) member C16 | | | Q9Y2G8 |  |
| DNAJC17 | DnaJ heat shock protein family (Hsp40) member C17 | | | Q9NVM6 |  |
| DNAJC19 | DnaJ heat shock protein family (Hsp40) member C19 | | | Q96DA6 |  |
| DNAJC2 | DnaJ heat shock protein family (Hsp40) member C2 | | | Q99543 |  |
| DNAJC3 | DnaJ heat shock protein family (Hsp40) member C3 | | | Q13217 |  |
| DNAJC30 | DnaJ heat shock protein family (Hsp40) member C30 | | | Q96LL9 |  |
| DNAJC4 | DnaJ heat shock protein family (Hsp40) member C4 | | | Q9NNZ3 |  |
| DNAJC5 | DnaJ heat shock protein family (Hsp40) member C5 | | | Q9H3Z4 |  |
| DNAJC7 | DnaJ heat shock protein family (Hsp40) member C7 | | | Q99615 |  |
| DNAJC8 | DnaJ heat shock protein family (Hsp40) member C8 | | | O75937 |  |
| DNAL1 | Dynein axonemal light chain 1 | | | Q4LDG9 |  |
| DNAL4 | Dynein axonemal light chain 4 | | | O96015 |  |
| DNASE1L1 | Deoxyribonuclease 1 like 1 | | | P49184 |  |
| DNASE2 | Deoxyribonuclease 2, lysosomal | | | O00115 |  |
| DNM1L | Dynamin 1 like | | | O00429 |  |
| DNM2 | Dynamin 2 | | | P50570 |  |
| DNMT3A | DNA methyltransferase 3 alpha | | | Q9Y6K1 |  |
| DNPEP | Aspartyl aminopeptidase | | | Q9ULA0 |  |
| DNPH1 | 2'-deoxynucleoside 5'-phosphate N-hydrolase 1 | | | O43598 |  |
| DNTT | DNA nucleotidylexotransferase | | | P04053 |  |
| DNTTIP1 | Deoxynucleotidyltransferase terminal interacting protein 1 | | | Q9H147 |  |
| DNTTIP2 | Deoxynucleotidyltransferase terminal interacting protein 2 | | | Q5QJE6 |  |
| DOCK2 | Dedicator of cytokinesis 2 | | | Q92608 |  |
| DOHH | Deoxyhypusine hydroxylase | | | Q9BU89 |  |
| DOK1 | Docking protein 1 | | | Q99704 |  |
| DOK2 | Docking protein 2 | | | O60496 |  |
| DOLK | Dolichol kinase | | | Q9UPQ8 |  |
| DOLPP1 | Dolichyldiphosphatase 1 | | | Q86YN1 |  |
| DPAGT1 | Dolichyl-phosphate N-acetylglucosaminephosphotransferase 1 | | | Q9H3H5 |  |
| DPCD | Deleted in primary ciliary dyskinesia homolog (mouse) | | | Q9BVM2 |  |
| DPEP2 | Dipeptidase 2 | | | Q9H4A9 |  |
| DPF2 | Double PHD fingers 2 | | | Q92785 |  |
| DPH1 | Diphthamide biosynthesis 1 | | | Q9BZG8 |  |
| DPH2 | Diphthamide biosynthesis 2 | | | Q9BQC3 |  |
| DPH3 | Diphthamide biosynthesis 3 | | | Q96FX2 |  |
| DPH5 | Diphthamide biosynthesis 5 | | | Q9H2P9 |  |
| DPH6 | Diphthamine biosynthesis 6 | | | Q7L8W6 |  |
| DPH7 | Diphthamide biosynthesis 7 | | | Q9BTV6 |  |
| DPM1 | Dolichyl-phosphate mannosyltransferase subunit 1, catalytic | | | O60762 |  |
| DPM2 | Dolichyl-phosphate mannosyltransferase subunit 2, regulatory | | | O94777 |  |
| DPM3 | Dolichyl-phosphate mannosyltransferase subunit 3, regulatory | | | Q9P2X0 |  |
| DPP3 | Dipeptidyl peptidase 3 | | | Q9NY33 |  |
| DPP4 | Dipeptidyl peptidase 4 | | | P27487 |  |
| DPP7 | Dipeptidyl peptidase 7 | | | Q9UHL4 |  |
| DPP8 | Dipeptidyl peptidase 8 | | | Q6V1X1 |  |
| DPP9 | Dipeptidyl peptidase 9 | | | Q86TI2 |  |
| DPY19L3 | Dpy-19 like C-mannosyltransferase 3 | | | Q6ZPD9 |  |
| DPY19L4 | Dpy-19 like 4 | | | Q7Z388 |  |
| DPY30 | Dpy-30 histone methyltransferase complex regulatory subunit | | | Q9C005 |  |
| DPYD | Dihydropyrimidine dehydrogenase | | | Q12882 |  |
| DPYSL2 | Dihydropyrimidinase like 2 | | | Q16555 |  |
| DR1 | Down-regulator of transcription 1 | | | Q01658 |  |
| DRAM1 | DNA damage regulated autophagy modulator 1 | | | Q8N682 |  |
| DRAP1 | DR1 associated protein 1 | | | Q14919 |  |
| DRG1 | Developmentally regulated GTP binding protein 1 | | | Q9Y295 |  |
| DRG2 | Developmentally regulated GTP binding protein 2 | | | P55039 |  |
| DSC1 | Desmocollin 1 | | | Q08554 |  |
| DSN1 | DSN1 component of MIS12 kinetochore complex | | | Q9H410 |  |
| DSTN | Destrin, actin depolymerizing factor | | | P60981 |  |
| DSTYK | Dual serine/threonine and tyrosine protein kinase | | | Q6XUX3 |  |
| DTD2 | D-aminoacyl-tRNA deacylase 2 | | | Q96FN9 |  |
| DTNA | Dystrobrevin alpha | | | Q9Y4J8 |  |
| DTNB | Dystrobrevin beta | | | O60941 |  |
| DTNBP1 | Dystrobrevin binding protein 1 | | | Q96EV8 |  |
| DTWD1 | DTW domain containing 1 | | | Q8N5C7 |  |
| DTX2 | Deltex E3 ubiquitin ligase 2 | | | Q86UW9 |  |
| DTX3L | Deltex E3 ubiquitin ligase 3L | | | Q8TDB6 |  |
| DTYMK | Deoxythymidylate kinase | | | P23919 |  |
| DUS1L | Dihydrouridine synthase 1 like | | | Q6P1R4 |  |
| DUS2 | Dihydrouridine synthase 2 | | | Q9NX74 |  |
| DUS3L | Dihydrouridine synthase 3 like | | | Q96G46 |  |
| DUSP10 | Dual specificity phosphatase 10 | | | Q9Y6W6 |  |
| DUSP11 | Dual specificity phosphatase 11 | | | O75319 |  |
| DUSP12 | Dual specificity phosphatase 12 | | | Q9UNI6 |  |
| DUSP16 | Dual specificity phosphatase 16 | | | Q9BY84 |  |
| DUSP18 | Dual specificity phosphatase 18 | | | Q8NEJ0 |  |
| DUSP2 | Dual specificity phosphatase 2 | | | Q05923 |  |
| DUSP22 | Dual specificity phosphatase 22 | | | Q9NRW4 |  |
| DUSP23 | Dual specificity phosphatase 23 | | | Q9BVJ7 |  |
| DUSP28 | Dual specificity phosphatase 28 | | | Q4G0W2 |  |
| DUSP3 | Dual specificity phosphatase 3 | | | P51452 |  |
| DUSP5 | Dual specificity phosphatase 5 | | | Q16690 |  |
| DUSP6 | Dual specificity phosphatase 6 | | | Q16828 |  |
| DUT | Deoxyuridine triphosphatase | | | P33316 |  |
| DVL2 | Dishevelled segment polarity protein 2 | | | O14641 |  |
| DXO | Decapping exoribonuclease | | | O77932 |  |
| DYM | Dymeclin | | | Q7RTS9 |  |
| DYNC1LI1 | Dynein cytoplasmic 1 light intermediate chain 1 | | | Q9Y6G9 |  |
| DYNC1LI2 | Dynein cytoplasmic 1 light intermediate chain 2 | | | O43237 |  |
| DYNC2LI1 | Dynein cytoplasmic 2 light intermediate chain 1 | | | Q8TCX1 |  |
| DYNLL1 | Dynein light chain LC8-type 1 | | | P63167 |  |
| DYNLRB1 | Dynein light chain roadblock-type 1 | | | Q9NP97 |  |
| DYNLT1 | Dynein light chain Tctex-type 1 | | | P63172 |  |
| DYNLT3 | Dynein light chain Tctex-type 3 | | | P51808 |  |
| DYRK1A | Dual specificity tyrosine phosphorylation regulated kinase 1A | | | Q13627 |  |
| DYRK1B | Dual specificity tyrosine phosphorylation regulated kinase 1B | | | Q9Y463 |  |
| DYRK2 | Dual specificity tyrosine phosphorylation regulated kinase 2 | | | Q92630 |  |
| DZIP3 | DAZ interacting zinc finger protein 3 | | | Q86Y13 |  |
| E2F3 | E2F transcription factor 3 | | | O00716 |  |
| E2F4 | E2F transcription factor 4 | | | Q16254 |  |
| E4F1 | E4F transcription factor 1 | | | Q66K89 |  |
| EAF1 | ELL associated factor 1 | | | Q96JC9 |  |
| EAF2 | ELL associated factor 2 | | | Q96CJ1 |  |
| EARS2 | Glutamyl-tRNA synthetase 2, mitochondrial | | | Q5JPH6 |  |
| EBAG9 | Estrogen receptor binding site associated antigen 9 | | | O00559 |  |
| EBLN2 | Endogenous Bornavirus like nucleoprotein 2 | | | Q6P2I7 |  |
| EBNA1BP2 | EBNA1 binding protein 2 | | | Q99848 |  |
| EBP | EBP cholestenol delta-isomerase | | | Q15125 |  |
| EBPL | EBP like | | | Q9BY08 |  |
| ECD | Ecdysoneless cell cycle regulator | | | O95905 |  |
| ECE1 | Endothelin converting enzyme 1 | | | P42892 |  |
| ECH1 | Enoyl-CoA hydratase 1 | | | Q13011 |  |
| ECHDC1 | Ethylmalonyl-CoA decarboxylase 1 | | | Q9NTX5 |  |
| ECHDC2 | Enoyl-CoA hydratase domain containing 2 | | | Q86YB7 |  |
| ECHS1 | Enoyl-CoA hydratase, short chain 1 | | | P30084 |  |
| ECI1 | Enoyl-CoA delta isomerase 1 | | | P42126 |  |
| ECI2 | Enoyl-CoA delta isomerase 2 | | | O75521 |  |
| ECSIT | ECSIT signaling integrator | | | Q9BQ95 |  |
| ECT2 | Epithelial cell transforming 2 | | | Q9H8V3 |  |
| EDAR | Ectodysplasin A receptor | | | Q9UNE0 |  |
| EDARADD | EDAR associated death domain | | | Q8WWZ3 |  |
| EDC3 | Enhancer of mRNA decapping 3 | | | Q96F86 |  |
| EDC4 | Enhancer of mRNA decapping 4 | | | Q6P2E9 |  |
| EDEM1 | ER degradation enhancing alpha-mannosidase like protein 1 | | | Q92611 |  |
| EDEM2 | ER degradation enhancing alpha-mannosidase like protein 2 | | | Q9BV94 |  |
| EDF1 | Endothelial differentiation related factor 1 | | | O60869 |  |
| EED | Embryonic ectoderm development | | | O75530 |  |
| EEF1A1 | Eukaryotic translation elongation factor 1 alpha 1 | | | P68104 |  |
| EEF1B2 | Eukaryotic translation elongation factor 1 beta 2 | | | P24534 |  |
| EEF1D | Eukaryotic translation elongation factor 1 delta | | | P29692 |  |
| EEF1E1 | Eukaryotic translation elongation factor 1 epsilon 1 | | | O43324 |  |
| EEF1G | Eukaryotic translation elongation factor 1 gamma | | | P26641 |  |
| EEF2 | Eukaryotic translation elongation factor 2 | | | P13639 |  |
| EEF2KMT | Eukaryotic elongation factor 2 lysine methyltransferase | | | Q96G04 |  |
| EEFSEC | Eukaryotic elongation factor, selenocysteine-tRNA specific | | | P57772 |  |
| EEPD1 | Endonuclease/exonuclease/phosphatase family domain containing 1 | | | Q7L9B9 |  |
| EFCAB11 | EF-hand calcium binding domain 11 | | | Q9BUY7 |  |
| EFCAB13 | EF-hand calcium binding domain 13 | | | Q8IY85 |  |
| EFCAB14 | EF-hand calcium binding domain 14 | | | O75071 |  |
| EFCAB7 | EF-hand calcium binding domain 7 | | | A8K855 |  |
| EFHD2 | EF-hand domain family member D2 | | | Q96C19 |  |
| EFNA1 | Ephrin A1 | | | P20827 |  |
| EFNA1 | Ephrin A1 | | | P20827 |  |
| EFNA4 | Ephrin A4 | | | P52798 |  |
| EFR3A | EFR3 homolog A | | | Q14156 |  |
| EFTUD2 | Elongation factor Tu GTP binding domain containing 2 | | | Q15029 |  |
| EGLN2 | Egl-9 family hypoxia inducible factor 2 | | | Q96KS0 |  |
| EGLN3 | Egl-9 family hypoxia inducible factor 3 | | | Q9H6Z9 |  |
| EHD1 | EH domain containing 1 | | | Q9H4M9 |  |
| EHD3 | EH domain containing 3 | | | Q9NZN3 |  |
| EHMT1 | Euchromatic histone lysine methyltransferase 1 | | | Q9H9B1 |  |
| EI24 | EI24 autophagy associated transmembrane protein | | | O14681 |  |
| EID1 | EP300 interacting inhibitor of differentiation 1 | | | Q9Y6B2 |  |
| EID2B | EP300 interacting inhibitor of differentiation 2B | | | Q96D98 |  |
| EID3 | EP300 interacting inhibitor of differentiation 3 | | | Q8N140 |  |
| EIF1 | Eukaryotic translation initiation factor 1 | | | P41567 |  |
| EIF1AD | Eukaryotic translation initiation factor 1A domain containing | | | Q8N9N8 |  |
| EIF1AX | Eukaryotic translation initiation factor 1A X-linked | | | P47813 |  |
| EIF1AY | Eukaryotic translation initiation factor 1A Y-linked | | | O14602 |  |
| EIF1B | Eukaryotic translation initiation factor 1B | | | O60739 |  |
| EIF2A | Eukaryotic translation initiation factor 2A | | | Q9BY44 |  |
| EIF2AK1 | Eukaryotic translation initiation factor 2 alpha kinase 1 | | | Q9BQI3 |  |
| EIF2AK2 | Eukaryotic translation initiation factor 2 alpha kinase 2 | | | P19525 |  |
| EIF2AK4 | Eukaryotic translation initiation factor 2 alpha kinase 4 | | | Q9P2K8 |  |
| EIF2B1 | Eukaryotic translation initiation factor 2B subunit alpha | | | Q14232 |  |
| EIF2B2 | Eukaryotic translation initiation factor 2B subunit beta | | | P49770 |  |
| EIF2B3 | Eukaryotic translation initiation factor 2B subunit gamma | | | Q9NR50 |  |
| EIF2B4 | Eukaryotic translation initiation factor 2B subunit delta | | | Q9UI10 |  |
| EIF2B5 | Eukaryotic translation initiation factor 2B subunit epsilon | | | Q13144 |  |
| EIF2D | Eukaryotic translation initiation factor 2D | | | P41214 |  |
| EIF2S1 | Eukaryotic translation initiation factor 2 subunit alpha | | | P05198 |  |
| EIF2S2 | Eukaryotic translation initiation factor 2 subunit beta | | | P20042 |  |
| EIF2S3 | Eukaryotic translation initiation factor 2 subunit gamma | | | P41091 |  |
| EIF3C | Eukaryotic translation initiation factor 3 subunit C | | | Q99613 |  |
| EIF3D | Eukaryotic translation initiation factor 3 subunit D | | | O15371 |  |
| EIF3E | Eukaryotic translation initiation factor 3 subunit E | | | P60228 |  |
| EIF3F | Eukaryotic translation initiation factor 3 subunit F | | | O00303 |  |
| EIF3G | Eukaryotic translation initiation factor 3 subunit G | | | O75821 |  |
| EIF3H | Eukaryotic translation initiation factor 3 subunit H | | | O15372 |  |
| EIF3I | Eukaryotic translation initiation factor 3 subunit I | | | Q13347 |  |
| EIF3J | Eukaryotic translation initiation factor 3 subunit J | | | O75822 |  |
| EIF3K | Eukaryotic translation initiation factor 3 subunit K | | | Q9UBQ5 |  |
| EIF3L | Eukaryotic translation initiation factor 3 subunit L | | | Q9Y262 |  |
| EIF3M | Eukaryotic translation initiation factor 3 subunit M | | | Q7L2H7 |  |
| EIF4A1 | Eukaryotic translation initiation factor 4A1 | | | P60842 |  |
| EIF4A2 | Eukaryotic translation initiation factor 4A2 | | | Q14240 |  |
| EIF4A3 | Eukaryotic translation initiation factor 4A3 | | | P38919 |  |
| EIF4B | Eukaryotic translation initiation factor 4B | | | P23588 |  |
| EIF4E | Eukaryotic translation initiation factor 4E | | | P06730 |  |
| EIF4E2 | Eukaryotic translation initiation factor 4E family member 2 | | | O60573 |  |
| EIF4E3 | Eukaryotic translation initiation factor 4E family member 3 | | | Q8N5X7 |  |
| EIF4EBP1 | Eukaryotic translation initiation factor 4E binding protein 1 | | | Q13541 |  |
| EIF4EBP2 | Eukaryotic translation initiation factor 4E binding protein 2 | | | Q13542 |  |
| EIF4EBP3 | Eukaryotic translation initiation factor 4E binding protein 3 | | | O60516 |  |
| EIF4ENIF1 | Eukaryotic translation initiation factor 4E nuclear import factor 1 | | | Q9NRA8 |  |
| EIF4H | Eukaryotic translation initiation factor 4H | | | Q15056 |  |
| EIF5 | Eukaryotic translation initiation factor 5 | | | P55010 |  |
| EIF5A | Eukaryotic translation initiation factor 5A | | | P63241 |  |
| EIF5A2 | Eukaryotic translation initiation factor 5A2 | | | Q9GZV4 |  |
| EIF5B | Eukaryotic translation initiation factor 5B | | | O60841 |  |
| EIF6 | Eukaryotic translation initiation factor 6 | | | P56537 |  |
| ELAC1 | ElaC ribonuclease Z 1 | | | Q9H777 |  |
| ELAC2 | ElaC ribonuclease Z 2 | | | Q9BQ52 |  |
| ELAVL1 | ELAV like RNA binding protein 1 | | | Q15717 |  |
| ELF1 | E74 like ETS transcription factor 1 | | | P32519 |  |
| ELF2 | E74 like ETS transcription factor 2 | | | Q15723 |  |
| ELF4 | E74 like ETS transcription factor 4 | | | Q99607 |  |
| ELK1 | ETS transcription factor ELK1 | | | P19419 |  |
| ELK3 | ETS transcription factor ELK3 | | | P41970 |  |
| ELK4 | ETS transcription factor ELK4 | | | P28324 |  |
| ELL | Elongation factor for RNA polymerase II | | | P55199 |  |
| ELL3 | Elongation factor for RNA polymerase II 3 | | | Q9HB65 |  |
| ELMO1 | Engulfment and cell motility 1 | | | Q92556 |  |
| ELMO2 | Engulfment and cell motility 2 | | | Q96JJ3 |  |
| ELMOD2 | ELMO domain containing 2 | | | Q8IZ81 |  |
| ELMOD3 | ELMO domain containing 3 | | | Q96FG2 |  |
| ELOF1 | Elongation factor 1 | | | P60002 |  |
| ELOVL1 | ELOVL fatty acid elongase 1 | | | Q9BW60 |  |
| ELOVL4 | ELOVL fatty acid elongase 4 | | | Q9GZR5 |  |
| ELOVL5 | ELOVL fatty acid elongase 5 | | | Q9NYP7 |  |
| ELOVL6 | ELOVL fatty acid elongase 6 | | | Q9H5J4 |  |
| ELP2 | Elongator acetyltransferase complex subunit 2 | | | Q6IA86 |  |
| ELP3 | Elongator acetyltransferase complex subunit 3 | | | Q9H9T3 |  |
| ELP4 | Elongator acetyltransferase complex subunit 4 | | | Q96EB1 |  |
| ELP5 | Elongator acetyltransferase complex subunit 5 | | | Q8TE02 |  |
| ELP6 | Elongator acetyltransferase complex subunit 6 | | | Q0PNE2 |  |
| EMB | Embigin | | | Q6PCB8 |  |
| EMC1 | ER membrane protein complex subunit 1 | | | Q8N766 |  |
| EMC10 | ER membrane protein complex subunit 10 | | | Q5UCC4 |  |
| EMC2 | ER membrane protein complex subunit 2 | | | Q15006 |  |
| EMC3 | ER membrane protein complex subunit 3 | | | Q9P0I2 |  |
| EMC4 | ER membrane protein complex subunit 4 | | | Q5J8M3 |  |
| EMC6 | ER membrane protein complex subunit 6 | | | Q9BV81 |  |
| EMC7 | ER membrane protein complex subunit 7 | | | Q9NPA0 |  |
| EMC8 | ER membrane protein complex subunit 8 | | | O43402 |  |
| EMC9 | ER membrane protein complex subunit 9 | | | Q9Y3B6 |  |
| EMD | Emerin | | | P50402 |  |
| EMG1 | EMG1 N1-specific pseudouridine methyltransferase | | | Q92979 |  |
| EMP3 | Epithelial membrane protein 3 | | | P54852 |  |
| ENDOD1 | Endonuclease domain containing 1 | | | O94919 |  |
| ENDOV | Endonuclease V | | | Q8N8Q3 |  |
| ENG | Endoglin | | | P17813 |  |
| ENO1 | Enolase 1 | | | P06733 |  |
| ENO2 | Enolase 2 | | | P09104 |  |
| ENO3 | Enolase 3 | | | P13929 |  |
| ENOPH1 | Enolase-phosphatase 1 | | | Q9UHY7 |  |
| ENOSF1 | Enolase superfamily member 1 | | | Q7L5Y1 |  |
| ENOX2 | Ecto-NOX disulfide-thiol exchanger 2 | | | Q16206 |  |
| ENPP2 | Ectonucleotide pyrophosphatase/phosphodiesterase 2 | | | Q13822 |  |
| ENPP4 | Ectonucleotide pyrophosphatase/phosphodiesterase 4 | | | Q9Y6X5 |  |
| ENPP5 | Ectonucleotide pyrophosphatase/phosphodiesterase family member 5 | | | Q9UJA9 |  |
| ENSA | Endosulfine alpha | | | O43768 |  |
| ENTPD1 | Ectonucleoside triphosphate diphosphohydrolase 1 | | | P49961 |  |
| ENTPD4 | Ectonucleoside triphosphate diphosphohydrolase 4 | | | Q9Y227 |  |
| ENTPD5 | Ectonucleoside triphosphate diphosphohydrolase 5 (inactive) | | | O75356 |  |
| ENTPD6 | Ectonucleoside triphosphate diphosphohydrolase 6 | | | O75354 |  |
| ENY2 | ENY2 transcription and export complex 2 subunit | | | Q9NPA8 |  |
| EOGT | EGF domain specific O-linked N-acetylglucosamine transferase | | | Q5NDL2 |  |
| EOMES | Eomesodermin | | | O95936 |  |
| EP400 | E1A binding protein p400 | | | Q96L91 |  |
| EPB41 | Erythrocyte membrane protein band 4.1 | | | P11171 |  |
| EPB41L2 | Erythrocyte membrane protein band 4.1 like 2 | | | O43491 |  |
| EPB41L5 | Erythrocyte membrane protein band 4.1 like 5 | | | Q9HCM4 |  |
| EPC1 | Enhancer of polycomb homolog 1 | | | Q9H2F5 |  |
| EPHA1 | EPH receptor A1 | | | P21709 |  |
| EPHA4 | EPH receptor A4 | | | P54764 |  |
| EPHB6 | EPH receptor B6 | | |  |  |
| EPHX2 | Epoxide hydrolase 2 | | | P34913 |  |
| EPHX4 | Epoxide hydrolase 4 | | | Q8IUS5 |  |
| EPM2AIP1 | EPM2A interacting protein 1 | | | Q7L775 |  |
| EPOR | Erythropoietin receptor | | | P19235 |  |
| EPS15L1 | Epidermal growth factor receptor pathway substrate 15 like 1 | | | Q9UBC2 |  |
| EPS8 | Epidermal growth factor receptor pathway substrate 8 | | | Q12929 |  |
| EPSTI1 | Epithelial stromal interaction 1 | | | Q96J88 |  |
| ERAL1 | Era like 12S mitochondrial rRNA chaperone 1 | | | O75616 |  |
| ERAP1 | Endoplasmic reticulum aminopeptidase 1 | | | Q9NZ08 |  |
| ERAP2 | Endoplasmic reticulum aminopeptidase 2 | | | Q6P179 |  |
| ERCC1 | ERCC excision repair 1, endonuclease non-catalytic subunit | | | P07992 |  |
| ERCC2 | ERCC excision repair 2, TFIIH core complex helicase subunit | | | P18074 |  |
| ERCC3 | ERCC excision repair 3, TFIIH core complex helicase subunit | | | P19447 |  |
| ERCC4 | ERCC excision repair 4, endonuclease catalytic subunit | | | Q92889 |  |
| ERCC5 | ERCC excision repair 5, endonuclease | | | P28715 |  |
| ERCC6L2 | ERCC excision repair 6 like 2 | | | Q5T890 |  |
| ERCC8 | ERCC excision repair 8, CSA ubiquitin ligase complex subunit | | | Q13216 |  |
| ERGIC1 | Endoplasmic reticulum-golgi intermediate compartment 1 | | | Q969X5 |  |
| ERGIC2 | ERGIC and golgi 2 | | | Q96RQ1 |  |
| ERGIC3 | ERGIC and golgi 3 | | | Q9Y282 |  |
| ERH | ERH mRNA splicing and mitosis factor | | | P84090 |  |
| ERI1 | Exoribonuclease 1 | | | Q8IV48 |  |
| ERI3 | ERI1 exoribonuclease family member 3 | | | O43414 |  |
| ERICH1 | Glutamate rich 1 | | | Q86X53 |  |
| ERICH6 | Glutamate rich 6 | | | Q7L0X2 |  |
| ERLEC1 | Endoplasmic reticulum lectin 1 | | | Q96DZ1 |  |
| ERLIN1 | ER lipid raft associated 1 | | | O75477 |  |
| ERLIN2 | ER lipid raft associated 2 | | | O94905 |  |
| ERMAP | Erythroblast membrane associated protein (Scianna blood group) | | | Q96PL5 |  |
| ERN1 | Endoplasmic reticulum to nucleus signaling 1 | | | O75460 |  |
| ERO1A | Endoplasmic reticulum oxidoreductase 1 alpha | | | Q96HE7 |  |
| ERP27 | Endoplasmic reticulum protein 27 | | | Q96DN0 |  |
| ERP29 | Endoplasmic reticulum protein 29 | | | P30040 |  |
| ERP44 | Endoplasmic reticulum protein 44 | | | Q9BS26 |  |
| ESAM | Endothelial cell adhesion molecule | | | Q96AP7 |  |
| ESCO1 | Establishment of sister chromatid cohesion N-acetyltransferase 1 | | | Q5FWF5 |  |
| ESD | Esterase D | | | P10768 |  |
| ESF1 | ESF1 nucleolar pre-rRNA processing protein homolog | | | Q9H501 |  |
| ESYT1 | Extended synaptotagmin 1 | | | Q9BSJ8 |  |
| ESYT2 | Extended synaptotagmin 2 | | | A0FGR8 |  |
| ETF1 | Eukaryotic translation termination factor 1 | | | P62495 |  |
| ETFA | Electron transfer flavoprotein subunit alpha | | | P13804 |  |
| ETFB | Electron transfer flavoprotein subunit beta | | | P38117 |  |
| ETFDH | Electron transfer flavoprotein dehydrogenase | | | Q16134 |  |
| ETHE1 | ETHE1 persulfide dioxygenase | | | O95571 |  |
| ETNK1 | Ethanolamine kinase 1 | | | Q9HBU6 |  |
| ETNK2 | Ethanolamine kinase 2 | | | Q9NVF9 |  |
| ETS1 | ETS proto-oncogene 1, transcription factor | | | P14921 |  |
| ETS2 | ETS proto-oncogene 2, transcription factor | | | P15036 |  |
| ETV2 | ETS variant transcription factor 2 | | | O00321 |  |
| ETV3 | ETS variant transcription factor 3 | | | P41162 |  |
| ETV6 | ETS variant transcription factor 6 | | | P41212 |  |
| ETV7 | ETS variant transcription factor 7 | | | Q9Y603 |  |
| EVA1C | Eva-1 homolog C | | | P58658 |  |
| EVI2A | Ecotropic viral integration site 2A | | | P22794 |  |
| EVI2B | Ecotropic viral integration site 2B | | | P34910 |  |
| EVL | Enah/Vasp-like | | | Q9UI08 |  |
| EWSR1 | EWS RNA binding protein 1 | | | Q01844 |  |
| EXD2 | Exonuclease 3'-5' domain containing 2 | | | Q9NVH0 |  |
| EXD3 | Exonuclease 3'-5' domain containing 3 | | | Q8N9H8 |  |
| EXO5 | Exonuclease 5 | | | Q9H790 |  |
| EXOC1 | Exocyst complex component 1 | | | Q9NV70 |  |
| EXOC3 | Exocyst complex component 3 | | | O60645 |  |
| EXOC5 | Exocyst complex component 5 | | | O00471 |  |
| EXOC6 | Exocyst complex component 6 | | | Q8TAG9 |  |
| EXOC7 | Exocyst complex component 7 | | | Q9UPT5 |  |
| EXOG | Exo/endonuclease G | | | Q9Y2C4 |  |
| EXOSC1 | Exosome component 1 | | | Q9Y3B2 |  |
| EXOSC10 | Exosome component 10 | | | Q01780 |  |
| EXOSC2 | Exosome component 2 | | | Q13868 |  |
| EXOSC3 | Exosome component 3 | | | Q9NQT5 |  |
| EXOSC4 | Exosome component 4 | | | Q9NPD3 |  |
| EXOSC5 | Exosome component 5 | | | Q9NQT4 |  |
| EXOSC7 | Exosome component 7 | | | Q15024 |  |
| EXOSC8 | Exosome component 8 | | | Q96B26 |  |
| EXT2 | Exostosin glycosyltransferase 2 | | | Q93063 |  |
| EXTL2 | Exostosin like glycosyltransferase 2 | | | Q9UBQ6 |  |
| EYA3 | EYA transcriptional coactivator and phosphatase 3 | | | Q99504 |  |
| EZH1 | Enhancer of zeste 1 polycomb repressive complex 2 subunit | | | Q92800 |  |
| EZH2 | Enhancer of zeste 2 polycomb repressive complex 2 subunit | | | Q15910 |  |
| EZR | Ezrin | | | P15311 |  |
| F11R | F11 receptor | | | Q9Y624 |  |
| F2R | Coagulation factor II thrombin receptor | | | P25116 |  |
| F2RL1 | F2R like trypsin receptor 1 | | | P55085 |  |
| F5 | Coagulation factor V | | | P12259 |  |
| F8 | Coagulation factor VIII | | | P00451 |  |
| F8A1 | Coagulation factor VIII associated 1 | | | P23610 |  |
| FAAH2 | Fatty acid amide hydrolase 2 | | | Q6GMR7 |  |
| FAAP24 | FA core complex associated protein 24 | | | Q9BTP7 |  |
| FABP5 | Fatty acid binding protein 5 | | | Q01469 |  |
| FADD | Fas associated via death domain | | | Q13158 |  |
| FADS1 | Fatty acid desaturase 1 | | | O60427 |  |
| FADS2 | Fatty acid desaturase 2 | | | O95864 |  |
| FADS3 | Fatty acid desaturase 3 | | | Q9Y5Q0 |  |
| FAF1 | Fas associated factor 1 | | | Q9UNN5 |  |
| FAF2 | Fas associated factor family member 2 | | | Q96CS3 |  |
| FAH | Fumarylacetoacetate hydrolase | | | P16930 |  |
| FAHD1 | Fumarylacetoacetate hydrolase domain containing 1 | | | Q6P587 |  |
| FAHD2A | Fumarylacetoacetate hydrolase domain containing 2A | | | Q96GK7 |  |
| FAHD2B | Fumarylacetoacetate hydrolase domain containing 2B | | | Q6P2I3 |  |
| FAIM | Fas apoptotic inhibitory molecule | | | Q9NVQ4 |  |
| FAM102A | Family with sequence similarity 102 member A | | | Q5T9C2 |  |
| FAM104B | Family with sequence similarity 104 member B | | | Q5XKR9 |  |
| FAM107B | Family with sequence similarity 107 member B | | | Q9H098 |  |
| FAM110A | Family with sequence similarity 110 member A | | | Q9BQ89 |  |
| FAM111A | FAM111 trypsin like peptidase A | | | Q96PZ2 |  |
| FAM114A2 | Family with sequence similarity 114 member A2 | | | Q9NRY5 |  |
| FAM117A | Family with sequence similarity 117 member A | | | Q9C073 |  |
| FAM118A | Family with sequence similarity 118 member A | | | Q9NWS6 |  |
| FAM118B | Family with sequence similarity 118 member B | | | Q9BPY3 |  |
| FAM120A | Family with sequence similarity 120A | | | Q9NZB2 |  |
| FAM120AOS | Family with sequence similarity 120A opposite strand | | | Q5T036 |  |
| FAM120B | Family with sequence similarity 120B | | | Q96EK7 |  |
| FAM133B | Family with sequence similarity 133 member B | | | Q5BKY9 |  |
| FAM135A | Family with sequence similarity 135 member A | | | Q9P2D6 |  |
| FAM136A | Family with sequence similarity 136 member A | | | Q96C01 |  |
| FAM13A | Family with sequence similarity 13 member A | | | O94988 |  |
| FAM13B | Family with sequence similarity 13 member B | | | Q9NYF5 |  |
| FAM153A | Family with sequence similarity 153 member A | | | Q9UHL3 |  |
| FAM156A | Family with sequence similarity 156 member A | | | Q8NDB6 |  |
| FAM161B | FAM161 centrosomal protein B | | | Q96MY7 |  |
| FAM162A | Family with sequence similarity 162 member A | | | Q96A26 |  |
| FAM168B | Family with sequence similarity 168 member B | | | A1KXE4 |  |
| FAM172A | Family with sequence similarity 172 member A | | | Q8WUF8 |  |
| FAM174A | Family with sequence similarity 174 member A | | | Q8TBP5 |  |
| FAM177A1 | Family with sequence similarity 177 member A1 | | | Q8N128 |  |
| FAM200A | Family with sequence similarity 200 member A | | | Q8TCP9 |  |
| FAM204A | Family with sequence similarity 204 member A | | | Q9H8W3 |  |
| FAM20B | FAM20B glycosaminoglycan xylosylkinase | | | O75063 |  |
| FAM210A | Family with sequence similarity 210 member A | | | Q96ND0 |  |
| FAM216A | Family with sequence similarity 216 member A | | | Q8WUB2 |  |
| FAM217B | Family with sequence similarity 217 member B | | | Q9NTX9 |  |
| FAM219B | Family with sequence similarity 219 member B | | | Q5XKK7 |  |
| FAM220A | Family with sequence similarity 220 member A | | | Q7Z4H9 |  |
| FAM221A | Family with sequence similarity 221 member A | | | A4D161 |  |
| FAM222B | Family with sequence similarity 222 member B | | | Q8WU58 |  |
| FAM227B | Family with sequence similarity 227 member B | | | Q96M60 |  |
| FAM234A | Family with sequence similarity 234 member A | | | Q9H0X4 |  |
| FAM234B | Family with sequence similarity 234 member B | | | A2RU67 |  |
| FAM24B | Family with sequence similarity 24 member B | | | Q8N5W8 |  |
| FAM3A | FAM3 metabolism regulating signaling molecule A | | | P98173 |  |
| FAM3C | FAM3 metabolism regulating signaling molecule C | | | Q92520 |  |
| FAM50A | Family with sequence similarity 50 member A | | | Q14320 |  |
| FAM50B | Family with sequence similarity 50 member B | | | Q9Y247 |  |
| FAM53A | Family with sequence similarity 53 member A | | | Q6NSI3 |  |
| FAM53B | Family with sequence similarity 53 member B | | | Q14153 |  |
| FAM53C | Family with sequence similarity 53 member C | | | Q9NYF3 |  |
| FAM76A | Family with sequence similarity 76 member A | | | Q8TAV0 |  |
| FAM76B | Family with sequence similarity 76 member B | | | Q5HYJ3 |  |
| FAM78A | Family with sequence similarity 78 member A | | | Q5JUQ0 |  |
| FAM89B | Family with sequence similarity 89 member B | | | Q8N5H3 |  |
| FAM98A | Family with sequence similarity 98 member A | | | Q8NCA5 |  |
| FAM98B | Family with sequence similarity 98 member B | | | Q52LJ0 |  |
| FAM98C | Family with sequence similarity 98 member C | | | Q17RN3 |  |
| FAN1 | FANCD2 and FANCI associated nuclease 1 | | | Q9Y2M0 |  |
| FANCA | FA complementation group A | | | O15360 |  |
| FANCF | FA complementation group F | | | Q9NPI8 |  |
| FANCG | FA complementation group G | | | O15287 |  |
| FANCI | FA complementation group I | | | Q9NVI1 |  |
| FANCL | FA complementation group L | | | Q9NW38 |  |
| FANCM | FA complementation group M | | | Q8IYD8 |  |
| FANK1 | Fibronectin type III and ankyrin repeat domains 1 | | | Q8TC84 |  |
| FAR1 | Fatty acyl-CoA reductase 1 | | | Q8WVX9 |  |
| FARP2 | FERM, ARH/RhoGEF and pleckstrin domain protein 2 | | | O94887 |  |
| FARS2 | Phenylalanyl-tRNA synthetase 2, mitochondrial | | | O95363 |  |
| FARSA | Phenylalanyl-tRNA synthetase subunit alpha | | | Q9Y285 |  |
| FARSB | Phenylalanyl-tRNA synthetase subunit beta | | | Q9NSD9 |  |
| FAS | Fas cell surface death receptor | | | P25445 |  |
| FASLG | Fas ligand | | | P48023 |  |
| FASTK | Fas activated serine/threonine kinase | | | Q14296 |  |
| FASTKD2 | FAST kinase domains 2 | | | Q9NYY8 |  |
| FASTKD3 | FAST kinase domains 3 | | | Q14CZ7 |  |
| FASTKD5 | FAST kinase domains 5 | | | Q7L8L6 |  |
| FAU | FAU ubiquitin like and ribosomal protein S30 fusion | | | P62861 |  |
| FBL | Fibrillarin | | | P22087 |  |
| FBLN7 | Fibulin 7 | | | Q53RD9 |  |
| FBP1 | Fructose-bisphosphatase 1 | | | P09467 |  |
| FBXL12 | F-box and leucine rich repeat protein 12 | | | Q9NXK8 |  |
| FBXL15 | F-box and leucine rich repeat protein 15 | | | Q9H469 |  |
| FBXL20 | F-box and leucine rich repeat protein 20 | | | Q96IG2 |  |
| FBXL22 | F-box and leucine rich repeat protein 22 | | | Q6P050 |  |
| FBXL3 | F-box and leucine rich repeat protein 3 | | | Q9UKT7 |  |
| FBXL4 | F-box and leucine rich repeat protein 4 | | | Q9UKA2 |  |
| FBXL5 | F-box and leucine rich repeat protein 5 | | | Q9UKA1 |  |
| FBXL8 | F-box and leucine rich repeat protein 8 | | | Q96CD0 |  |
| FBXO11 | F-box protein 11 | | | Q86XK2 |  |
| FBXO15 | F-box protein 15 | | | Q8NCQ5 |  |
| FBXO21 | F-box protein 21 | | | O94952 |  |
| FBXO22 | F-box protein 22 | | | Q8NEZ5 |  |
| FBXO25 | F-box protein 25 | | | Q8TCJ0 |  |
| FBXO28 | F-box protein 28 | | | Q9NVF7 |  |
| FBXO3 | F-box protein 3 | | | Q9UK99 |  |
| FBXO32 | F-box protein 32 | | | Q969P5 |  |
| FBXO33 | F-box protein 33 | | | Q7Z6M2 |  |
| FBXO34 | F-box protein 34 | | | Q9NWN3 |  |
| FBXO36 | F-box protein 36 | | | Q8NEA4 |  |
| FBXO38 | F-box protein 38 | | | Q6PIJ6 |  |
| FBXO4 | F-box protein 4 | | | Q9UKT5 |  |
| FBXO42 | F-box protein 42 | | | Q6P3S6 |  |
| FBXO44 | F-box protein 44 | | | Q9H4M3 |  |
| FBXO5 | F-box protein 5 | | | Q9UKT4 |  |
| FBXO6 | F-box protein 6 | | | Q9NRD1 |  |
| FBXO7 | F-box protein 7 | | | Q9Y3I1 |  |
| FBXO8 | F-box protein 8 | | | Q9NRD0 |  |
| FBXO9 | F-box protein 9 | | | Q9UK97 |  |
| FBXW11 | F-box and WD repeat domain containing 11 | | | Q9UKB1 |  |
| FBXW2 | F-box and WD repeat domain containing 2 | | | Q9UKT8 |  |
| FBXW4 | F-box and WD repeat domain containing 4 | | | P57775 |  |
| FBXW5 | F-box and WD repeat domain containing 5 | | | Q969U6 |  |
| FBXW7 | F-box and WD repeat domain containing 7 | | | Q969H0 |  |
| FBXW9 | F-box and WD repeat domain containing 9 | | | Q5XUX1 |  |
| FCER1A | Fc epsilon receptor Ia | | | P12319 |  |
| FCER1G | Fc epsilon receptor Ig | | | P30273 |  |
| FCF1 | FCF1 rRNA-processing protein | | | Q9Y324 |  |
| FCGR3A | Fc gamma receptor IIIa | | | P08637 |  |
| FCGRT | Fc gamma receptor and transporter | | | P55899 |  |
| FCHO1 | FCH and mu domain containing endocytic adaptor 1 | | | O14526 |  |
| FCHSD1 | FCH and double SH3 domains 1 | | | Q86WN1 |  |
| FCHSD2 | FCH and double SH3 domains 2 | | | O94868 |  |
| FCMR | Fc mu receptor | | | O60667 |  |
| FCN1 | Ficolin 1 | | | O00602 |  |
| FCRL3 | Fc receptor like 3 | | | Q96P31 |  |
| FDFT1 | Farnesyl-diphosphate farnesyltransferase 1 | | | P37268 |  |
| FDPS | Farnesyl diphosphate synthase | | | P14324 |  |
| FDX1 | Ferredoxin 1 | | | P10109 |  |
| FDXACB1 | Ferredoxin-fold anticodon binding domain containing 1 | | | Q9BRP7 |  |
| FDXR | Ferredoxin reductase | | | P22570 |  |
| FECH | Ferrochelatase | | | P22830 |  |
| FEM1A | Fem-1 homolog A | | | Q9BSK4 |  |
| FEM1B | Fem-1 homolog B | | | Q9UK73 |  |
| FEM1C | Fem-1 homolog C | | | Q96JP0 |  |
| FEN1 | Flap structure-specific endonuclease 1 | | | P39748 |  |
| FERMT3 | FERM domain containing kindlin 3 | | | Q86UX7 |  |
| FGD3 | FYVE, RhoGEF and PH domain containing 3 | | | Q5JSP0 |  |
| FGF23 | Fibroblast growth factor 23 | | | Q9GZV9 |  |
| FGFBP2 | Fibroblast growth factor binding protein 2 | | | Q9BYJ0 |  |
| FGFR1OP2 | FGFR1 oncogene partner 2 | | | Q9NVK5 |  |
| FGGY | FGGY carbohydrate kinase domain containing | | | Q96C11 |  |
| FGL2 | Fibrinogen like 2 | | | Q14314 |  |
| FGR | FGR proto-oncogene, Src family tyrosine kinase | | | P09769 |  |
| FHIT | Fragile histidine triad diadenosine triphosphatase | | | P49789 |  |
| FHIT | Fragile histidine triad diadenosine triphosphatase | | | P49789 |  |
| FHL1 | Four and a half LIM domains 1 | | | Q13642 |  |
| FHL3 | Four and a half LIM domains 3 | | | Q13643 |  |
| FIBP | FGF1 intracellular binding protein | | | O43427 |  |
| FICD | FIC domain protein adenylyltransferase | | | Q9BVA6 |  |
| FIG4 | FIG4 phosphoinositide 5-phosphatase | | | Q92562 |  |
| FIGNL1 | Fidgetin like 1 | | | Q6PIW4 |  |
| FIP1L1 | Factor interacting with PAPOLA and CPSF1 | | | Q6UN15 |  |
| FIS1 | Fission, mitochondrial 1 | | | Q9Y3D6 |  |
| FITM2 | Fat storage inducing transmembrane protein 2 | | | Q8N6M3 |  |
| FKBP11 | FKBP prolyl isomerase 11 | | | Q9NYL4 |  |
| FKBP1A | FKBP prolyl isomerase 1A | | | P62942 |  |
| FKBP2 | FKBP prolyl isomerase 2 | | | P26885 |  |
| FKBP3 | FKBP prolyl isomerase 3 | | | Q00688 |  |
| FKBP4 | FKBP prolyl isomerase 4 | | | Q02790 |  |
| FKBP5 | FKBP prolyl isomerase 5 | | | Q13451 |  |
| FKBP7 | FKBP prolyl isomerase 7 | | | Q9Y680 |  |
| FKBP8 | FKBP prolyl isomerase 8 | | | Q14318 |  |
| FKBPL | FKBP prolyl isomerase like | | | Q9UIM3 |  |
| FKRP | Fukutin related protein | | | Q9H9S5 |  |
| FLAD1 | Flavin adenine dinucleotide synthetase 1 | | | Q8NFF5 |  |
| FLCN | Folliculin | | | Q8NFG4 |  |
| FLI1 | Fli-1 proto-oncogene, ETS transcription factor | | | Q01543 |  |
| FLII | FLII actin remodeling protein | | | Q13045 |  |
| FLOT1 | Flotillin 1 | | | O75955 |  |
| FLOT2 | Flotillin 2 | | | Q14254 |  |
| FLT3LG | Fms related receptor tyrosine kinase 3 ligand | | | P49771 |  |
| FLVCR2 | FLVCR heme transporter 2 | | | Q9UPI3 |  |
| FLYWCH1 | FLYWCH-type zinc finger 1 | | | Q4VC44 |  |
| FLYWCH2 | FLYWCH family member 2 | | | Q96CP2 |  |
| FMO4 | Flavin containing dimethylaniline monoxygenase 4 | | | P31512 |  |
| FMR1 | Fragile X messenger ribonucleoprotein 1 | | | Q06787 |  |
| FN3KRP | Fructosamine 3 kinase related protein | | | Q9HA64 |  |
| FNBP4 | Formin binding protein 4 | | | Q8N3X1 |  |
| FNDC3A | Fibronectin type III domain containing 3A | | | Q9Y2H6 |  |
| FNDC3B | Fibronectin type III domain containing 3B | | | Q53EP0 |  |
| FNIP1 | Folliculin interacting protein 1 | | | Q8TF40 |  |
| FNTA | Farnesyltransferase, CAAX box, alpha | | | P49354 |  |
| FNTB | Farnesyltransferase, CAAX box, beta | | | P49356 |  |
| FOSL2 | FOS like 2, AP-1 transcription factor subunit | | | P15408 |  |
| FOXJ2 | Forkhead box J2 | | | Q9P0K8 |  |
| FOXJ3 | Forkhead box J3 | | | Q9UPW0 |  |
| FOXK1 | Forkhead box K1 | | | P85037 |  |
| FOXK2 | Forkhead box K2 | | | Q01167 |  |
| FOXN2 | Forkhead box N2 | | | P32314 |  |
| FOXN3 | Forkhead box N3 | | | O00409 |  |
| FOXO1 | Forkhead box O1 | | | Q12778 |  |
| FOXO3 | Forkhead box O3 | | | O43524 |  |
| FOXP1 | Forkhead box P1 | | | Q9H334 |  |
| FOXP3 | Forkhead box P3 | | | Q9BZS1 |  |
| FOXRED1 | FAD dependent oxidoreductase domain containing 1 | | | Q96CU9 |  |
| FPGS | Folylpolyglutamate synthase | | | Q05932 |  |
| FPGT | Fucose-1-phosphate guanylyltransferase | | | O14772 |  |
| FRA10AC1 | FRA10A associated CGG repeat 1 | | | Q70Z53 |  |
| FRAT2 | FRAT regulator of WNT signaling pathway 2 | | | O75474 |  |
| FRG1 | FSHD region gene 1 | | | Q14331 |  |
| FRMD4B | FERM domain containing 4B | | | Q9Y2L6 |  |
| FRMD8 | FERM domain containing 8 | | | Q9BZ67 |  |
| FRS2 | Fibroblast growth factor receptor substrate 2 | | | Q8WU20 |  |
| FRYL | FRY like transcription coactivator | | | O94915 |  |
| FSTL3 | Follistatin like 3 | | | O95633 |  |
| FTCDNL1 | Formiminotransferase cyclodeaminase N-terminal like | | | E5RQL4 |  |
| FTH1 | Ferritin heavy chain 1 | | | P02794 |  |
| FTL | Ferritin light chain | | | P02792 |  |
| FTSJ1 | FtsJ RNA 2'-O-methyltransferase 1 | | | Q9UET6 |  |
| FTSJ3 | FtsJ RNA 2'-O-methyltransferase 3 | | | Q8IY81 |  |
| FUBP1 | Far upstream element binding protein 1 | | | Q96AE4 |  |
| FUBP3 | Far upstream element binding protein 3 | | | Q96I24 |  |
| FUCA1 | Alpha-L-fucosidase 1 | | | P04066 |  |
| FUCA2 | Alpha-L-fucosidase 2 | | | Q9BTY2 |  |
| FUNDC1 | FUN14 domain containing 1 | | | Q8IVP5 |  |
| FUNDC2 | FUN14 domain containing 2 | | | Q9BWH2 |  |
| FUOM | Fucose mutarotase | | | A2VDF0 |  |
| FUS | FUS RNA binding protein | | | P35637 |  |
| FUT11 | Fucosyltransferase 11 | | | Q495W5 |  |
| FUT7 | Fucosyltransferase 7 | | | Q11130 |  |
| FUT8 | Fucosyltransferase 8 | | | Q9BYC5 |  |
| FUZ | Fuzzy planar cell polarity protein | | | Q9BT04 |  |
| FXN | Frataxin | | | Q16595 |  |
| FXR1 | FMR1 autosomal homolog 1 | | | P51114 |  |
| FXR2 | FMR1 autosomal homolog 2 | | | P51116 |  |
| FXYD1 | FXYD domain containing ion transport regulator 1 | | | O00168 |  |
| FXYD2 | FXYD domain containing ion transport regulator 2 | | | P54710 |  |
| FXYD2 | FXYD domain containing ion transport regulator 2 | | | P54710 |  |
| FXYD5 | FXYD domain containing ion transport regulator 5 | | | Q96DB9 |  |
| FXYD7 | FXYD domain containing ion transport regulator 7 | | | P58549 |  |
| FYCO1 | FYVE and coiled-coil domain autophagy adaptor 1 | | | Q9BQS8 |  |
| FYN | FYN proto-oncogene, Src family tyrosine kinase | | | P06241 |  |
| FYTTD1 | Forty-two-three domain containing 1 | | | Q96QD9 |  |
| FZD3 | Frizzled class receptor 3 | | | Q9NPG1 |  |
| FZR1 | Fizzy and cell division cycle 20 related 1 | | | Q9UM11 |  |
| G0S2 | G0/G1 switch 2 | | | P27469 |  |
| G2E3 | G2/M-phase specific E3 ubiquitin protein ligase | | | Q7L622 |  |
| G3BP1 | G3BP stress granule assembly factor 1 | | | Q13283 |  |
| G3BP2 | G3BP stress granule assembly factor 2 | | | Q9UN86 |  |
| G6PC3 | Glucose-6-phosphatase catalytic subunit 3 | | | Q9BUM1 |  |
| G6PD | Glucose-6-phosphate dehydrogenase | | | P11413 |  |
| GAA | Alpha glucosidase | | | P10253 |  |
| GAB3 | GRB2 associated binding protein 3 | | | Q8WWW8 |  |
| GABARAP | GABA type A receptor-associated protein | | | O95166 |  |
| GABARAPL1 | GABA type A receptor associated protein like 1 | | | Q9H0R8 |  |
| GABARAPL2 | GABA type A receptor associated protein like 2 | | | P60520 |  |
| GABPA | GA binding protein transcription factor subunit alpha | | | Q06546 |  |
| GABPB1 | GA binding protein transcription factor subunit beta 1 | | | Q06547 |  |
| GABPB2 | GA binding protein transcription factor subunit beta 2 | | | Q8TAK5 |  |
| GABRG2 | Gamma-aminobutyric acid type A receptor subunit gamma2 | | | P18507 |  |
| GADD45A | Growth arrest and DNA damage inducible alpha | | | P24522 |  |
| GADD45B | Growth arrest and DNA damage inducible beta | | | O75293 |  |
| GADD45GIP1 | GADD45G interacting protein 1 | | | Q8TAE8 |  |
| GAK | Cyclin G associated kinase | | | O14976 |  |
| GAL3ST4 | Galactose-3-O-sulfotransferase 4 | | | Q96RP7 |  |
| GALC | Galactosylceramidase | | | P54803 |  |
| GALE | UDP-galactose-4-epimerase | | | Q14376 |  |
| GALK1 | Galactokinase 1 | | | P51570 |  |
| GALK2 | Galactokinase 2 | | | Q01415 |  |
| GALM | Galactose mutarotase | | | Q96C23 |  |
| GALNS | Galactosamine (N-acetyl)-6-sulfatase | | | P34059 |  |
| GALNT1 | Polypeptide N-acetylgalactosaminyltransferase 1 | | | Q10472 |  |
| GALNT10 | Polypeptide N-acetylgalactosaminyltransferase 10 | | | Q86SR1 |  |
| GALNT2 | Polypeptide N-acetylgalactosaminyltransferase 2 | | | Q10471 |  |
| GALNT3 | Polypeptide N-acetylgalactosaminyltransferase 3 | | | Q14435 |  |
| GALNT4 | Polypeptide N-acetylgalactosaminyltransferase 4 | | | Q8N4A0 |  |
| GALNT6 | Polypeptide N-acetylgalactosaminyltransferase 6 | | | Q8NCL4 |  |
| GALNT7 | Polypeptide N-acetylgalactosaminyltransferase 7 | | | Q86SF2 |  |
| GALT | Galactose-1-phosphate uridylyltransferase | | | P07902 |  |
| GAMT | Guanidinoacetate N-methyltransferase | | | Q14353 |  |
| GANAB | Glucosidase II alpha subunit | | | Q14697 |  |
| GANC | Glucosidase alpha, neutral C | | | Q8TET4 |  |
| GAPDH | Glyceraldehyde-3-phosphate dehydrogenase | | | P04406 |  |
| GAPVD1 | GTPase activating protein and VPS9 domains 1 | | | Q14C86 |  |
| GAR1 | GAR1 ribonucleoprotein | | | Q9NY12 |  |
| GART | Phosphoribosylglycinamide formyltransferase, phosphoribosylglycinamide synthetase, phosphoribosylaminoimidazole synthetase | | | P22102 |  |
| GATA3 | GATA binding protein 3 | | | P23771 |  |
| GATC | Glutamyl-tRNA amidotransferase subunit C | | | O43716 |  |
| GBA2 | Glucosylceramidase beta 2 | | | Q9HCG7 |  |
| GBE1 | 1,4-alpha-glucan branching enzyme 1 | | | Q04446 |  |
| GBP1 | Guanylate binding protein 1 | | | P32455 |  |
| GBP2 | Guanylate binding protein 2 | | | P32456 |  |
| GBP3 | Guanylate binding protein 3 | | | Q9H0R5 |  |
| GBP4 | Guanylate binding protein 4 | | | Q96PP9 |  |
| GBP5 | Guanylate binding protein 5 | | | Q96PP8 |  |
| GCA | Grancalcin | | | P28676 |  |
| GCAT | Glycine C-acetyltransferase | | | O75600 |  |
| GCC1 | GRIP and coiled-coil domain containing 1 | | | Q96CN9 |  |
| GCDH | Glutaryl-CoA dehydrogenase | | | Q92947 |  |
| GCFC2 | GC-rich sequence DNA-binding factor 2 | | | P16383 |  |
| GCH1 | GTP cyclohydrolase 1 | | | P30793 |  |
| GCHFR | GTP cyclohydrolase I feedback regulator | | | P30047 |  |
| GCK | Glucokinase | | | P35557 |  |
| GCLM | Glutamate-cysteine ligase modifier subunit | | | P48507 |  |
| GCNT1 | Glucosaminyl (N-acetyl) transferase 1 | | | Q02742 |  |
| GCNT4 | Glucosaminyl (N-acetyl) transferase 4 | | | Q9P109 |  |
| GCSAM | Germinal center associated signaling and motility | | | Q8N6F7 |  |
| GCSH | Glycine cleavage system protein H | | | P23434 |  |
| GDAP2 | Ganglioside induced differentiation associated protein 2 | | | Q9NXN4 |  |
| GDE1 | Glycerophosphodiester phosphodiesterase 1 | | | Q9NZC3 |  |
| GDI1 | GDP dissociation inhibitor 1 | | | P31150 |  |
| GDI2 | GDP dissociation inhibitor 2 | | | P50395 |  |
| GDPD5 | Glycerophosphodiester phosphodiesterase domain containing 5 | | | Q8WTR4 |  |
| GDPGP1 | GDP-D-glucose phosphorylase 1 | | | Q6ZNW5 |  |
| GEMIN2 | Gem nuclear organelle associated protein 2 | | | O14893 |  |
| GEMIN4 | Gem nuclear organelle associated protein 4 | | | P57678 |  |
| GEMIN6 | Gem nuclear organelle associated protein 6 | | | Q8WXD5 |  |
| GEMIN7 | Gem nuclear organelle associated protein 7 | | | Q9H840 |  |
| GEMIN8 | Gem nuclear organelle associated protein 8 | | | Q9NWZ8 |  |
| GET4 | Guided entry of tail-anchored proteins factor 4 | | | Q7L5D6 |  |
| GFER | Growth factor, augmenter of liver regeneration | | | P55789 |  |
| GFI1 | Growth factor independent 1 transcriptional repressor | | | Q99684 |  |
| GFM1 | G elongation factor mitochondrial 1 | | | Q96RP9 |  |
| GFM2 | GTP dependent ribosome recycling factor mitochondrial 2 | | | Q969S9 |  |
| GFOD2 | Glucose-fructose oxidoreductase domain containing 2 | | | Q3B7J2 |  |
| GFPT1 | Glutamine--fructose-6-phosphate transaminase 1 | | | Q06210 |  |
| GGA1 | Golgi associated, gamma adaptin ear containing, ARF binding protein 1 | | | Q9UJY5 |  |
| GGA2 | Golgi associated, gamma adaptin ear containing, ARF binding protein 2 | | | Q9UJY4 |  |
| GGA3 | Golgi associated, gamma adaptin ear containing, ARF binding protein 3 | | | Q9NZ52 |  |
| GGACT | Gamma-glutamylamine cyclotransferase | | | Q9BVM4 |  |
| GGCT | Gamma-glutamylcyclotransferase | | | O75223 |  |
| GGCX | Gamma-glutamyl carboxylase | | | P38435 |  |
| GGH | Gamma-glutamyl hydrolase | | | Q92820 |  |
| GGPS1 | Geranylgeranyl diphosphate synthase 1 | | | O95749 |  |
| GGT1 | Gamma-glutamyltransferase 1 | | | P19440 |  |
| GGT7 | Gamma-glutamyltransferase 7 | | | Q9UJ14 |  |
| GGTLC2 | Gamma-glutamyltransferase light chain 2 | | | Q14390 |  |
| GHDC | GH3 domain containing | | | Q8N2G8 |  |
| GHITM | Growth hormone inducible transmembrane protein | | | Q9H3K2 |  |
| GID8 | GID complex subunit 8 homolog | | | Q9NWU2 |  |
| GIGYF1 | GRB10 interacting GYF protein 1 | | | O75420 |  |
| GIGYF2 | GRB10 interacting GYF protein 2 | | | Q6Y7W6 |  |
| GIMAP1 | GTPase, IMAP family member 1 | | | Q8WWP7 |  |
| GIMAP2 | GTPase, IMAP family member 2 | | | Q9UG22 |  |
| GIMAP4 | GTPase, IMAP family member 4 | | | Q9NUV9 |  |
| GIMAP5 | GTPase, IMAP family member 5 | | | Q96F15 |  |
| GIMAP6 | GTPase, IMAP family member 6 | | | Q6P9H5 |  |
| GIMAP7 | GTPase, IMAP family member 7 | | | Q8NHV1 |  |
| GIMAP8 | GTPase, IMAP family member 8 | | | Q8ND71 |  |
| GIN1 | Gypsy retrotransposon integrase 1 | | | Q9NXP7 |  |
| GINM1 | Glycosylated integral membrane protein 1 | | | Q9NU53 |  |
| GINS2 | GINS complex subunit 2 | | | Q9Y248 |  |
| GINS3 | GINS complex subunit 3 | | | Q9BRX5 |  |
| GINS4 | GINS complex subunit 4 | | | Q9BRT9 |  |
| GIPC1 | GIPC PDZ domain containing family member 1 | | | O14908 |  |
| GIT1 | GIT ArfGAP 1 | | | Q9Y2X7 |  |
| GIT2 | GIT ArfGAP 2 | | | Q14161 |  |
| GJB6 | Gap junction protein beta 6 | | | O95452 |  |
| GK | Glycerol kinase | | | P32189 |  |
| GLA | Galactosidase alpha | | | P06280 |  |
| GLB1 | Galactosidase beta 1 | | | P16278 |  |
| GLB1L3 | Galactosidase beta 1 like 3 | | | Q8NCI6 |  |
| GLE1 | GLE1 RNA export mediator | | | Q53GS7 |  |
| GLIPR1 | GLI pathogenesis related 1 | | | P48060 |  |
| GLIPR2 | GLI pathogenesis related 2 | | | Q9H4G4 |  |
| GLMN | Glomulin, FKBP associated protein | | | Q92990 |  |
| GLMP | Glycosylated lysosomal membrane protein | | | Q8WWB7 |  |
| GLO1 | Glyoxalase I | | | Q04760 |  |
| GLOD4 | Glyoxalase domain containing 4 | | | Q9HC38 |  |
| GLRX | Glutaredoxin | | | P35754 |  |
| GLRX2 | Glutaredoxin 2 | | | Q9NS18 |  |
| GLRX3 | Glutaredoxin 3 | | | O76003 |  |
| GLRX5 | Glutaredoxin 5 | | | Q86SX6 |  |
| GLS2 | Glutaminase 2 | | | Q9UI32 |  |
| GLT8D1 | Glycosyltransferase 8 domain containing 1 | | | Q68CQ7 |  |
| GLTP | Glycolipid transfer protein | | | Q9NZD2 |  |
| GLUD1 | Glutamate dehydrogenase 1 | | | P00367 |  |
| GLUL | Glutamate-ammonia ligase | | | P15104 |  |
| GLYCTK | Glycerate kinase | | | Q8IVS8 |  |
| GLYR1 | Glyoxylate reductase 1 homolog | | | Q49A26 |  |
| GM2A | Ganglioside GM2 activator | | | P17900 |  |
| GMCL1 | Germ cell-less 1, spermatogenesis associated | | | Q96IK5 |  |
| GMDS | GDP-mannose 4,6-dehydratase | | | O60547 |  |
| GMEB1 | Glucocorticoid modulatory element binding protein 1 | | | Q9Y692 |  |
| GMEB2 | Glucocorticoid modulatory element binding protein 2 | | | Q9UKD1 |  |
| GMFB | Glia maturation factor beta | | | P60983 |  |
| GMFG | Glia maturation factor gamma | | | O60234 |  |
| GMIP | GEM interacting protein | | | Q9P107 |  |
| GMNN | Geminin DNA replication inhibitor | | | O75496 |  |
| GMPPA | GDP-mannose pyrophosphorylase A | | | Q96IJ6 |  |
| GMPPB | GDP-mannose pyrophosphorylase B | | | Q9Y5P6 |  |
| GMPR2 | Guanosine monophosphate reductase 2 | | | Q9P2T1 |  |
| GMPS | Guanine monophosphate synthase | | | P49915 |  |
| GNA11 | G protein subunit alpha 11 | | | P29992 |  |
| GNA13 | G protein subunit alpha 13 | | | Q14344 |  |
| GNA15 | G protein subunit alpha 15 | | | P30679 |  |
| GNAI1 | G protein subunit alpha i1 | | | P63096 |  |
| GNAI2 | G protein subunit alpha i2 | | | P04899 |  |
| GNAI3 | G protein subunit alpha i3 | | | P08754 |  |
| GNAQ | G protein subunit alpha q | | | P50148 |  |
| GNAS | GNAS complex locus | | | O95467, P63092, Q5JWF2 |  |
| GNB1 | G protein subunit beta 1 | | | P62873 |  |
| GNB1L | G protein subunit beta 1 like | | | Q9BYB4 |  |
| GNB2 | G protein subunit beta 2 | | | P62879 |  |
| GNB5 | G protein subunit beta 5 | | | O14775 |  |
| GNE | Glucosamine (UDP-N-acetyl)-2-epimerase/N-acetylmannosamine kinase | | | Q9Y223 |  |
| GNG10 | G protein subunit gamma 10 | | | P50151 |  |
| GNG2 | G protein subunit gamma 2 | | | P59768 |  |
| GNG5 | G protein subunit gamma 5 | | | P63218 |  |
| GNG7 | G protein subunit gamma 7 | | | O60262 |  |
| GNG8 | G protein subunit gamma 8 | | | Q9UK08 |  |
| GNGT2 | G protein subunit gamma transducin 2 | | | O14610 |  |
| GNL1 | G protein nucleolar 1 (putative) | | | P36915 |  |
| GNL2 | G protein nucleolar 2 | | | Q13823 |  |
| GNL3 | G protein nucleolar 3 | | | Q9BVP2 |  |
| GNL3L | G protein nucleolar 3 like | | | Q9NVN8 |  |
| GNLY | Granulysin | | | P22749 |  |
| GNPAT | Glyceronephosphate O-acyltransferase | | | O15228 |  |
| GNPDA1 | Glucosamine-6-phosphate deaminase 1 | | | P46926 |  |
| GNPDA2 | Glucosamine-6-phosphate deaminase 2 | | | Q8TDQ7 |  |
| GNPTAB | N-acetylglucosamine-1-phosphate transferase subunits alpha and beta | | | Q3T906 |  |
| GNPTG | N-acetylglucosamine-1-phosphate transferase subunit gamma | | | Q9UJJ9 |  |
| GNS | Glucosamine (N-acetyl)-6-sulfatase | | | P15586 |  |
| GOLGA1 | Golgin A1 | | | Q92805 |  |
| GOLGA2 | Golgin A2 | | | Q08379 |  |
| GOLGA4 | Golgin A4 | | | Q13439 |  |
| GOLGA5 | Golgin A5 | | | Q8TBA6 |  |
| GOLGA7 | Golgin A7 | | | Q7Z5G4 |  |
| GOLPH3 | Golgi phosphoprotein 3 | | | Q9H4A6 |  |
| GOLPH3L | Golgi phosphoprotein 3 like | | | Q9H4A5 |  |
| GOLT1B | Golgi transport 1B | | | Q9Y3E0 |  |
| GON4L | Gon-4 like | | | Q3T8J9 |  |
| GOPC | Golgi associated PDZ and coiled-coil motif containing | | | Q9HD26 |  |
| GORAB | Golgin, RAB6 interacting | | | Q5T7V8 |  |
| GORASP1 | Golgi reassembly stacking protein 1 | | | Q9BQQ3 |  |
| GORASP2 | Golgi reassembly stacking protein 2 | | | Q9H8Y8 |  |
| GOSR1 | Golgi SNAP receptor complex member 1 | | | O95249 |  |
| GOSR2 | Golgi SNAP receptor complex member 2 | | | O14653 |  |
| GOT1 | Glutamic-oxaloacetic transaminase 1 | | | P17174 |  |
| GOT2 | Glutamic-oxaloacetic transaminase 2 | | | P00505 |  |
| GP1BB | Glycoprotein Ib platelet subunit beta | | | P13224 |  |
| GPA33 | Glycoprotein A33 | | | Q99795 |  |
| GPA33 | Glycoprotein A33 | | | Q99795 |  |
| GPAA1 | Glycosylphosphatidylinositol anchor attachment 1 | | | O43292 |  |
| GPANK1 | G-patch domain and ankyrin repeats 1 | | | O95872 |  |
| GPAT4 | Glycerol-3-phosphate acyltransferase 4 | | | Q86UL3 |  |
| GPATCH1 | G-patch domain containing 1 | | | Q9BRR8 |  |
| GPATCH11 | G-patch domain containing 11 | | |  |  |
| GPATCH2 | G-patch domain containing 2 | | | Q9NW75 |  |
| GPATCH2L | G-patch domain containing 2 like | | | Q9NWQ4 |  |
| GPATCH3 | G-patch domain containing 3 | | | Q96I76 |  |
| GPATCH4 | G-patch domain containing 4 (gene/pseudogene) | | |  |  |
| GPBP1 | GC-rich promoter binding protein 1 | | | Q86WP2 |  |
| GPBP1L1 | GC-rich promoter binding protein 1 like 1 | | | Q9HC44 |  |
| GPC3 | Glypican 3 | | | P51654 |  |
| GPCPD1 | Glycerophosphocholine phosphodiesterase 1 | | | Q9NPB8 |  |
| GPD1L | Glycerol-3-phosphate dehydrogenase 1 like | | | Q8N335 |  |
| GPHN | Gephyrin | | | Q9NQX3 |  |
| GPI | Glucose-6-phosphate isomerase | | | P06744 |  |
| GPKOW | G-patch domain and KOW motifs | | | Q92917 |  |
| GPN1 | GPN-loop GTPase 1 | | | Q9HCN4 |  |
| GPN2 | GPN-loop GTPase 2 | | | Q9H9Y4 |  |
| GPN3 | GPN-loop GTPase 3 | | | Q9UHW5 |  |
| GPR108 | G protein-coupled receptor 108 | | | Q9NPR9 |  |
| GPR132 | G protein-coupled receptor 132 | | | Q9UNW8 |  |
| GPR137 | G protein-coupled receptor 137 | | | Q96N19 |  |
| GPR141 | G protein-coupled receptor 141 | | | Q7Z602 |  |
| GPR146 | G protein-coupled receptor 146 | | | Q96CH1 |  |
| GPR146 | G protein-coupled receptor 146 | | | Q96CH1 |  |
| GPR15 | G protein-coupled receptor 15 | | | P49685 |  |
| GPR155 | G protein-coupled receptor 155 | | | Q7Z3F1 |  |
| GPR160 | G protein-coupled receptor 160 | | | Q9UJ42 |  |
| GPR171 | G protein-coupled receptor 171 | | | O14626 |  |
| GPR18 | G protein-coupled receptor 18 | | | Q14330 |  |
| GPR183 | G protein-coupled receptor 183 | | | P32249 |  |
| GPR65 | G protein-coupled receptor 65 | | | Q8IYL9 |  |
| GPR68 | G protein-coupled receptor 68 | | | Q15743 |  |
| GPR89A | G protein-coupled receptor 89A | | | B7ZAQ6 |  |
| GPRASP2 | G protein-coupled receptor associated sorting protein 2 | | | Q96D09 |  |
| GPRC5B | G protein-coupled receptor class C group 5 member B | | | Q9NZH0 |  |
| GPRIN3 | GPRIN family member 3 | | | Q6ZVF9 |  |
| GPS1 | G protein pathway suppressor 1 | | | Q13098 |  |
| GPS2 | G protein pathway suppressor 2 | | | Q13227 |  |
| GPSM3 | G protein signaling modulator 3 | | | Q9Y4H4 |  |
| GPX7 | Glutathione peroxidase 7 | | | Q96SL4 |  |
| GRAMD1A | GRAM domain containing 1A | | | Q96CP6 |  |
| GRAMD1B | GRAM domain containing 1B | | | Q3KR37 |  |
| GRAP2 | GRB2 related adaptor protein 2 | | | O75791 |  |
| GRAPL | GRB2 related adaptor protein like | | | Q8TC17 |  |
| GRB2 | Growth factor receptor bound protein 2 | | | P62993 |  |
| GRHPR | Glyoxylate and hydroxypyruvate reductase | | | Q9UBQ7 |  |
| GRINA | Glutamate ionotropic receptor NMDA type subunit associated protein 1 | | | Q7Z429 |  |
| GRIPAP1 | GRIP1 associated protein 1 | | | Q4V328 |  |
| GRK5 | G protein-coupled receptor kinase 5 | | | P34947 |  |
| GRK6 | G protein-coupled receptor kinase 6 | | | P43250 |  |
| GRN | Granulin precursor | | | P28799 |  |
| GRPEL1 | GrpE like 1, mitochondrial | | | Q9HAV7 |  |
| GRPEL2 | GrpE like 2, mitochondrial | | | Q8TAA5 |  |
| GRWD1 | Glutamate rich WD repeat containing 1 | | | Q9BQ67 |  |
| GSDMA | Gasdermin A | | | Q96QA5 |  |
| GSDMD | Gasdermin D | | | P57764 |  |
| GSK3B | Glycogen synthase kinase 3 beta | | | P49841 |  |
| GSKIP | GSK3B interacting protein | | | Q9P0R6 |  |
| GSPT1 | G1 to S phase transition 1 | | | P15170 |  |
| GSPT2 | G1 to S phase transition 2 | | | Q8IYD1 |  |
| GSR | Glutathione-disulfide reductase | | | P00390 |  |
| GSS | Glutathione synthetase | | | P48637 |  |
| GSTK1 | Glutathione S-transferase kappa 1 | | | Q9Y2Q3 |  |
| GSTM1 | Glutathione S-transferase mu 1 | | | P09488 |  |
| GSTM2 | Glutathione S-transferase mu 2 | | | P28161 |  |
| GSTM3 | Glutathione S-transferase mu 3 | | | P21266 |  |
| GSTM4 | Glutathione S-transferase mu 4 | | | Q03013 |  |
| GSTO1 | Glutathione S-transferase omega 1 | | | P78417 |  |
| GSTP1 | Glutathione S-transferase pi 1 | | | P09211 |  |
| GSTZ1 | Glutathione S-transferase zeta 1 | | | O43708 |  |
| GTDC1 | Glycosyltransferase like domain containing 1 | | | Q4AE62 |  |
| GTF2A1 | General transcription factor IIA subunit 1 | | | P52655 |  |
| GTF2A2 | General transcription factor IIA subunit 2 | | | P52657 |  |
| GTF2B | General transcription factor IIB | | | Q00403 |  |
| GTF2E1 | General transcription factor IIE subunit 1 | | | P29083 |  |
| GTF2E2 | General transcription factor IIE subunit 2 | | | P29084 |  |
| GTF2F1 | General transcription factor IIF subunit 1 | | | P35269 |  |
| GTF2F2 | General transcription factor IIF subunit 2 | | | P13984 |  |
| GTF2H1 | General transcription factor IIH subunit 1 | | | P32780 |  |
| GTF2H2 | General transcription factor IIH subunit 2 | | | Q13888 |  |
| GTF2H2C | GTF2H2 family member C | | | Q6P1K8 |  |
| GTF2H3 | General transcription factor IIH subunit 3 | | | Q13889 |  |
| GTF2H4 | General transcription factor IIH subunit 4 | | | Q92759 |  |
| GTF2H5 | General transcription factor IIH subunit 5 | | | Q6ZYL4 |  |
| GTF2I | General transcription factor IIi | | | P78347 |  |
| GTF2IRD2 | GTF2I repeat domain containing 2 | | | Q86UP8 |  |
| GTF3C2 | General transcription factor IIIC subunit 2 | | | Q8WUA4 |  |
| GTF3C3 | General transcription factor IIIC subunit 3 | | | Q9Y5Q9 |  |
| GTF3C5 | General transcription factor IIIC subunit 5 | | | Q9Y5Q8 |  |
| GTF3C6 | General transcription factor IIIC subunit 6 | | | Q969F1 |  |
| GTPBP1 | GTP binding protein 1 | | | O00178 |  |
| GTPBP3 | GTP binding protein 3, mitochondrial | | | Q969Y2 |  |
| GTPBP4 | GTP binding protein 4 | | | Q9BZE4 |  |
| GTPBP6 | GTP binding protein 6 (putative) | | | O43824 |  |
| GTPBP8 | GTP binding protein 8 (putative) | | | Q8N3Z3 |  |
| GTSF1 | Gametocyte specific factor 1 | | | Q8WW33 |  |
| GUCD1 | Guanylyl cyclase domain containing 1 | | | Q96NT3 |  |
| GUF1 | GTP binding elongation factor GUF1 | | | Q8N442 |  |
| GUK1 | Guanylate kinase 1 | | | Q16774 |  |
| GUSB | Glucuronidase beta | | | P08236 |  |
| GYG1 | Glycogenin 1 | | | P46976 |  |
| GYPC | Glycophorin C (Gerbich blood group) | | | P04921 |  |
| GYS1 | Glycogen synthase 1 | | | P13807 |  |
| GZMA | Granzyme A | | | P12544 |  |
| GZMA | Granzyme A | | | P12544 |  |
| GZMB | Granzyme B | | | P10144 |  |
| GZMH | Granzyme H | | | P20718 |  |
| GZMH | Granzyme H | | | P20718 |  |
| GZMK | Granzyme K | | | P49863 |  |
| GZMM | Granzyme M | | | P51124 |  |
| H6PD | Hexose-6-phosphate dehydrogenase/glucose 1-dehydrogenase | | | O95479 |  |
| HABP4 | Hyaluronan binding protein 4 | | | Q5JVS0 |  |
| HACD1 | 3-hydroxyacyl-CoA dehydratase 1 | | | B0YJ81 |  |
| HACD2 | 3-hydroxyacyl-CoA dehydratase 2 | | | Q6Y1H2 |  |
| HACD3 | 3-hydroxyacyl-CoA dehydratase 3 | | | Q9P035 |  |
| HACD4 | 3-hydroxyacyl-CoA dehydratase 4 | | | Q5VWC8 |  |
| HACE1 | HECT domain and ankyrin repeat containing E3 ubiquitin protein ligase 1 | | | Q8IYU2 |  |
| HACL1 | 2-hydroxyacyl-CoA lyase 1 | | | Q9UJ83 |  |
| HADH | Hydroxyacyl-CoA dehydrogenase | | | Q16836 |  |
| HADHA | Hydroxyacyl-CoA dehydrogenase trifunctional multienzyme complex subunit alpha | | | P40939 |  |
| HADHB | Hydroxyacyl-CoA dehydrogenase trifunctional multienzyme complex subunit beta | | | P55084 |  |
| HAGH | Hydroxyacylglutathione hydrolase | | | Q16775 |  |
| HAGHL | Hydroxyacylglutathione hydrolase like | | | Q6PII5 |  |
| HAPLN3 | Hyaluronan and proteoglycan link protein 3 | | | Q96S86 |  |
| HARBI1 | Harbinger transposase derived 1 | | | Q96MB7 |  |
| HARS2 | Histidyl-tRNA synthetase 2, mitochondrial | | | P49590 |  |
| HAT1 | Histone acetyltransferase 1 | | | O14929 |  |
| HAUS1 | HAUS augmin like complex subunit 1 | | | Q96CS2 |  |
| HAUS2 | HAUS augmin like complex subunit 2 | | | Q9NVX0 |  |
| HAUS3 | HAUS augmin like complex subunit 3 | | | Q68CZ6 |  |
| HAUS4 | HAUS augmin like complex subunit 4 | | | Q9H6D7 |  |
| HAUS5 | HAUS augmin like complex subunit 5 | | | O94927 |  |
| HAUS6 | HAUS augmin like complex subunit 6 | | | Q7Z4H7 |  |
| HAUS7 | HAUS augmin like complex subunit 7 | | | Q99871 |  |
| HAUS8 | HAUS augmin like complex subunit 8 | | | Q9BT25 |  |
| HAVCR1 | Hepatitis A virus cellular receptor 1 | | | Q96D42 |  |
| HAVCR1 | Hepatitis A virus cellular receptor 1 | | | Q96D42 |  |
| HAVCR2 | Hepatitis A virus cellular receptor 2 | | | Q8TDQ0 |  |
| HAX1 | HCLS1 associated protein X-1 | | | O00165 |  |
| HBA1 | Hemoglobin subunit alpha 1 | | | P69905 |  |
| HBB | Hemoglobin subunit beta | | | P68871 |  |
| HBP1 | HMG-box transcription factor 1 | | | O60381 |  |
| HBS1L | HBS1 like translational GTPase | | | Q9Y450 |  |
| HCCS | Holocytochrome c synthase | | | P53701 |  |
| HCFC1 | Host cell factor C1 | | | P51610 |  |
| HCFC1R1 | Host cell factor C1 regulator 1 | | | Q9NWW0 |  |
| HCK | HCK proto-oncogene, Src family tyrosine kinase | | | P08631 |  |
| HCLS1 | Hematopoietic cell-specific Lyn substrate 1 | | | P14317 |  |
| HCST | Hematopoietic cell signal transducer | | | Q9UBK5 |  |
| HDAC1 | Histone deacetylase 1 | | | Q13547 |  |
| HDAC11 | Histone deacetylase 11 | | | Q96DB2 |  |
| HDAC3 | Histone deacetylase 3 | | | O15379 |  |
| HDAC6 | Histone deacetylase 6 | | | Q9UBN7 |  |
| HDAC8 | Histone deacetylase 8 | | | Q9BY41 |  |
| HDAC9 | Histone deacetylase 9 | | | Q9UKV0 |  |
| HDDC2 | HD domain containing 2 | | | Q7Z4H3 |  |
| HDDC3 | HD domain containing 3 | | | Q8N4P3 |  |
| HDGF | Heparin binding growth factor | | | P51858 |  |
| HDHD2 | Haloacid dehalogenase like hydrolase domain containing 2 | | | Q9H0R4 |  |
| HDHD3 | Haloacid dehalogenase like hydrolase domain containing 3 | | | Q9BSH5 |  |
| HDLBP | High density lipoprotein binding protein | | | Q00341 |  |
| HEATR1 | HEAT repeat containing 1 | | | Q9H583 |  |
| HEATR5A | HEAT repeat containing 5A | | | Q86XA9 |  |
| HEBP1 | Heme binding protein 1 | | | Q9NRV9 |  |
| HEBP2 | Heme binding protein 2 | | | Q9Y5Z4 |  |
| HECTD3 | HECT domain E3 ubiquitin protein ligase 3 | | | Q5T447 |  |
| HELB | DNA helicase B | | | Q8NG08 |  |
| HELLS | Helicase, lymphoid specific | | | Q9NRZ9 |  |
| HEMGN | Hemogen | | | Q9BXL5 |  |
| HEMGN | Hemogen | | | Q9BXL5 |  |
| HEMK1 | HemK methyltransferase family member 1 | | | Q9Y5R4 |  |
| HENMT1 | HEN methyltransferase 1 | | | Q5T8I9 |  |
| HERC3 | HECT and RLD domain containing E3 ubiquitin protein ligase 3 | | | Q15034 |  |
| HERPUD1 | Homocysteine inducible ER protein with ubiquitin like domain 1 | | | Q15011 |  |
| HERPUD2 | HERPUD family member 2 | | | Q9BSE4 |  |
| HEXA | Hexosaminidase subunit alpha | | | P06865 |  |
| HEXB | Hexosaminidase subunit beta | | | P07686 |  |
| HEXIM1 | HEXIM P-TEFb complex subunit 1 | | | O94992 |  |
| HEXIM2 | HEXIM P-TEFb complex subunit 2 | | | Q96MH2 |  |
| HFE | Homeostatic iron regulator | | | Q30201 |  |
| HGS | Hepatocyte growth factor-regulated tyrosine kinase substrate | | | O14964 |  |
| HGSNAT | Heparan-alpha-glucosaminide N-acetyltransferase | | | Q68CP4 |  |
| HIBADH | 3-hydroxyisobutyrate dehydrogenase | | | P31937 |  |
| HIBCH | 3-hydroxyisobutyryl-CoA hydrolase | | | Q6NVY1 |  |
| HID1 | HID1 domain containing | | | Q8IV36 |  |
| HIF1A | Hypoxia inducible factor 1 subunit alpha | | | Q16665 |  |
| HIF1AN | Hypoxia inducible factor 1 subunit alpha inhibitor | | | Q9NWT6 |  |
| HIGD1A | HIG1 hypoxia inducible domain family member 1A | | | Q9Y241 |  |
| HIGD2A | HIG1 hypoxia inducible domain family member 2A | | | Q9BW72 |  |
| HILPDA | Hypoxia inducible lipid droplet associated | | | Q9Y5L2 |  |
| HINFP | Histone H4 transcription factor | | | Q9BQA5 |  |
| HINT1 | Histidine triad nucleotide binding protein 1 | | | P49773 |  |
| HINT2 | Histidine triad nucleotide binding protein 2 | | | Q9BX68 |  |
| HINT3 | Histidine triad nucleotide binding protein 3 | | | Q9NQE9 |  |
| HIP1R | Huntingtin interacting protein 1 related | | | O75146 |  |
| HIPK1 | Homeodomain interacting protein kinase 1 | | | Q86Z02 |  |
| HIPK3 | Homeodomain interacting protein kinase 3 | | | Q9H422 |  |
| HIRIP3 | HIRA interacting protein 3 | | | Q9BW71 |  |
| HIVEP2 | HIVEP zinc finger 2 | | | P31629 |  |
| HK1 | Hexokinase 1 | | | P19367 |  |
| HK2 | Hexokinase 2 | | | P52789 |  |
| HLA-A | Major histocompatibility complex, class I, A | | | P04439 |  |
| HLA-B | Major histocompatibility complex, class I, B | | | P01889 |  |
| HLA-DMB | Major histocompatibility complex, class II, DM beta | | | P28068 |  |
| HLA-DPA1 | Major histocompatibility complex, class II, DP alpha 1 | | | P20036 |  |
| HLA-DPB1 | Major histocompatibility complex, class II, DP beta 1 | | | P04440 |  |
| HLA-DQA1 | Major histocompatibility complex, class II, DQ alpha 1 | | | P01909 |  |
| HLA-DQB1 | Major histocompatibility complex, class II, DQ beta 1 | | |  |  |
| HLA-DRA | Major histocompatibility complex, class II, DR alpha | | | P01903 |  |
| HLA-DRB1 | Major histocompatibility complex, class II, DR beta 1 | | | P01911 |  |
| HLA-E | Major histocompatibility complex, class I, E | | | P13747 |  |
| HLA-F | Major histocompatibility complex, class I, F | | | P30511 |  |
| HLCS | Holocarboxylase synthetase | | | P50747 |  |
| HLTF | Helicase like transcription factor | | | Q14527 |  |
| HM13 | Histocompatibility minor 13 | | | Q8TCT9 |  |
| HMBOX1 | Homeobox containing 1 | | | Q6NT76 |  |
| HMBS | Hydroxymethylbilane synthase | | | P08397 |  |
| HMCES | 5-hydroxymethylcytosine binding, ES cell specific | | | Q96FZ2 |  |
| HMG20A | High mobility group 20A | | | Q9NP66 |  |
| HMG20B | High mobility group 20B | | | Q9P0W2 |  |
| HMGA1 | High mobility group AT-hook 1 | | | P17096 |  |
| HMGB1 | High mobility group box 1 | | | P09429 |  |
| HMGB2 | High mobility group box 2 | | | P26583 |  |
| HMGCL | 3-hydroxy-3-methylglutaryl-CoA lyase | | | P35914 |  |
| HMGCR | 3-hydroxy-3-methylglutaryl-CoA reductase | | | P04035 |  |
| HMGN1 | High mobility group nucleosome binding domain 1 | | | P05114 |  |
| HMGN2 | High mobility group nucleosomal binding domain 2 | | | P05204 |  |
| HMGN3 | High mobility group nucleosomal binding domain 3 | | | Q15651 |  |
| HMGN4 | High mobility group nucleosomal binding domain 4 | | | O00479 |  |
| HMGN5 | High mobility group nucleosome binding domain 5 | | | P82970 |  |
| HMGXB4 | HMG-box containing 4 | | | Q9UGU5 |  |
| HMOX2 | Heme oxygenase 2 | | | P30519 |  |
| HNRNPA0 | Heterogeneous nuclear ribonucleoprotein A0 | | | Q13151 |  |
| HNRNPA1 | Heterogeneous nuclear ribonucleoprotein A1 | | | P09651 |  |
| HNRNPAB | Heterogeneous nuclear ribonucleoprotein A/B | | | Q99729 |  |
| HNRNPC | Heterogeneous nuclear ribonucleoprotein C | | | P07910 |  |
| HNRNPD | Heterogeneous nuclear ribonucleoprotein D | | | Q14103 |  |
| HNRNPDL | Heterogeneous nuclear ribonucleoprotein D like | | | O14979 |  |
| HNRNPF | Heterogeneous nuclear ribonucleoprotein F | | | P52597 |  |
| HNRNPH1 | Heterogeneous nuclear ribonucleoprotein H1 | | | P31943 |  |
| HNRNPH2 | Heterogeneous nuclear ribonucleoprotein H2 | | | P55795 |  |
| HNRNPH3 | Heterogeneous nuclear ribonucleoprotein H3 | | | P31942 |  |
| HNRNPK | Heterogeneous nuclear ribonucleoprotein K | | | P61978 |  |
| HNRNPLL | Heterogeneous nuclear ribonucleoprotein L like | | | Q8WVV9 |  |
| HNRNPR | Heterogeneous nuclear ribonucleoprotein R | | | O43390 |  |
| HNRNPU | Heterogeneous nuclear ribonucleoprotein U | | | Q00839 |  |
| HNRNPUL1 | Heterogeneous nuclear ribonucleoprotein U like 1 | | | Q9BUJ2 |  |
| HNRNPUL2 | Heterogeneous nuclear ribonucleoprotein U like 2 | | | Q1KMD3 |  |
| HOMEZ | Homeobox and leucine zipper encoding | | | Q8IX15 |  |
| HOOK1 | Hook microtubule tethering protein 1 | | | Q9UJC3 |  |
| HOOK1 | Hook microtubule tethering protein 1 | | | Q9UJC3 |  |
| HOOK2 | Hook microtubule tethering protein 2 | | | Q96ED9 |  |
| HOOK3 | Hook microtubule tethering protein 3 | | | Q86VS8 |  |
| HOPX | HOP homeobox | | | Q9BPY8 |  |
| HOXB2 | Homeobox B2 | | | P14652 |  |
| HP1BP3 | Heterochromatin protein 1 binding protein 3 | | | Q5SSJ5 |  |
| HPCAL1 | Hippocalcin like 1 | | | P37235 |  |
| HPCAL4 | Hippocalcin like 4 | | | Q9UM19 |  |
| HPGD | 15-hydroxyprostaglandin dehydrogenase | | | P15428 |  |
| HPRT1 | Hypoxanthine phosphoribosyltransferase 1 | | | P00492 |  |
| HPS1 | HPS1 biogenesis of lysosomal organelles complex 3 subunit 1 | | | Q92902 |  |
| HPS3 | HPS3 biogenesis of lysosomal organelles complex 2 subunit 1 | | | Q969F9 |  |
| HPS4 | HPS4 biogenesis of lysosomal organelles complex 3 subunit 2 | | | Q9NQG7 |  |
| HPS5 | HPS5 biogenesis of lysosomal organelles complex 2 subunit 2 | | | Q9UPZ3 |  |
| HPS6 | HPS6 biogenesis of lysosomal organelles complex 2 subunit 3 | | | Q86YV9 |  |
| HPSE | Heparanase | | | Q9Y251 |  |
| HRH2 | Histamine receptor H2 | | | P25021 |  |
| HRH4 | Histamine receptor H4 | | | Q9H3N8 |  |
| HS1BP3 | HCLS1 binding protein 3 | | | Q53T59 |  |
| HS2ST1 | Heparan sulfate 2-O-sulfotransferase 1 | | | Q7LGA3 |  |
| HS3ST3B1 | Heparan sulfate-glucosamine 3-sulfotransferase 3B1 | | | Q9Y662 |  |
| HSBP1 | Heat shock factor binding protein 1 | | | O75506 |  |
| HSCB | HscB mitochondrial iron-sulfur cluster cochaperone | | | Q8IWL3 |  |
| HSD11B1 | Hydroxysteroid 11-beta dehydrogenase 1 | | | P28845 |  |
| HSD11B1L | Hydroxysteroid 11-beta dehydrogenase 1 like | | | Q7Z5J1 |  |
| HSD17B1 | Hydroxysteroid 17-beta dehydrogenase 1 | | | P14061 |  |
| HSD17B10 | Hydroxysteroid 17-beta dehydrogenase 10 | | | Q99714 |  |
| HSD17B11 | Hydroxysteroid 17-beta dehydrogenase 11 | | | Q8NBQ5 |  |
| HSD17B12 | Hydroxysteroid 17-beta dehydrogenase 12 | | | Q53GQ0 |  |
| HSD17B14 | Hydroxysteroid 17-beta dehydrogenase 14 | | | Q9BPX1 |  |
| HSD17B4 | Hydroxysteroid 17-beta dehydrogenase 4 | | | P51659 |  |
| HSD17B6 | Hydroxysteroid 17-beta dehydrogenase 6 | | | O14756 |  |
| HSD17B7 | Hydroxysteroid 17-beta dehydrogenase 7 | | | P56937 |  |
| HSD17B8 | Hydroxysteroid 17-beta dehydrogenase 8 | | | Q92506 |  |
| HSDL1 | Hydroxysteroid dehydrogenase like 1 | | | Q3SXM5 |  |
| HSDL2 | Hydroxysteroid dehydrogenase like 2 | | | Q6YN16 |  |
| HSF1 | Heat shock transcription factor 1 | | | Q00613 |  |
| HSF2 | Heat shock transcription factor 2 | | | Q03933 |  |
| HSF5 | Heat shock transcription factor 5 | | | Q4G112 |  |
| HSH2D | Hematopoietic SH2 domain containing | | | Q96JZ2 |  |
| HSP90AA1 | Heat shock protein 90 alpha family class A member 1 | | | P07900 |  |
| HSP90AB1 | Heat shock protein 90 alpha family class B member 1 | | | P08238 |  |
| HSP90B1 | Heat shock protein 90 beta family member 1 | | | P14625 |  |
| HSPA13 | Heat shock protein family A (Hsp70) member 13 | | | P48723 |  |
| HSPA14 | Heat shock protein family A (Hsp70) member 14 | | | Q0VDF9 |  |
| HSPA1A | Heat shock protein family A (Hsp70) member 1A | | | P0DMV8 |  |
| HSPA4 | Heat shock protein family A (Hsp70) member 4 | | | P34932 |  |
| HSPA5 | Heat shock protein family A (Hsp70) member 5 | | | P11021 |  |
| HSPA8 | Heat shock protein family A (Hsp70) member 8 | | | P11142 |  |
| HSPA9 | Heat shock protein family A (Hsp70) member 9 | | | P38646 |  |
| HSPB1 | Heat shock protein family B (small) member 1 | | | P04792 |  |
| HSPB11 | Heat shock protein family B (small) member 11 | | | Q9Y547 |  |
| HSPBAP1 | HSPB1 associated protein 1 | | | Q96EW2 |  |
| HSPBP1 | HSPA (Hsp70) binding protein 1 | | | Q9NZL4 |  |
| HSPD1 | Heat shock protein family D (Hsp60) member 1 | | | P10809 |  |
| HSPE1 | Heat shock protein family E (Hsp10) member 1 | | | P61604 |  |
| HSPH1 | Heat shock protein family H (Hsp110) member 1 | | | Q92598 |  |
| HTATIP2 | HIV-1 Tat interactive protein 2 | | | Q9BUP3 |  |
| HTRA2 | HtrA serine peptidase 2 | | | O43464 |  |
| HUS1 | HUS1 checkpoint clamp component | | | O60921 |  |
| HVCN1 | Hydrogen voltage gated channel 1 | | | Q96D96 |  |
| HYAL2 | Hyaluronidase 2 | | | Q12891 |  |
| HYAL3 | Hyaluronidase 3 | | | O43820 |  |
| HYI | Hydroxypyruvate isomerase (putative) | | | Q5T013 |  |
| HYKK | Hydroxylysine kinase | | | A2RU49 |  |
| HYKK | Hydroxylysine kinase | | | A2RU49 |  |
| HYLS1 | HYLS1 centriolar and ciliogenesis associated | | | Q96M11 |  |
| HYOU1 | Hypoxia up-regulated 1 | | | Q9Y4L1 |  |
| HYPK | Huntingtin interacting protein K | | | Q9NX55 |  |
| IAH1 | Isoamyl acetate hydrolyzing esterase 1 (putative) | | | Q2TAA2 |  |
| IARS2 | Isoleucyl-tRNA synthetase 2, mitochondrial | | | Q9NSE4 |  |
| IBTK | Inhibitor of Bruton tyrosine kinase | | | Q9P2D0 |  |
| ICA1 | Islet cell autoantigen 1 | | | Q05084 |  |
| ICA1L | Islet cell autoantigen 1 like | | | Q8NDH6 |  |
| ICAM1 | Intercellular adhesion molecule 1 | | | P05362 |  |
| ICAM2 | Intercellular adhesion molecule 2 | | | P13598 |  |
| ICAM3 | Intercellular adhesion molecule 3 | | | P32942 |  |
| ICAM4 | Intercellular adhesion molecule 4 (Landsteiner-Wiener blood group) | | | Q14773 |  |
| ICMT | Isoprenylcysteine carboxyl methyltransferase | | | O60725 |  |
| ICOS | Inducible T cell costimulator | | | Q9Y6W8 |  |
| ID2 | Inhibitor of DNA binding 2 | | | Q02363 |  |
| ID3 | Inhibitor of DNA binding 3 | | | Q02535 |  |
| IDE | Insulin degrading enzyme | | | P14735 |  |
| IDH1 | Isocitrate dehydrogenase (NADP(+)) 1 | | | O75874 |  |
| IDH2 | Isocitrate dehydrogenase (NADP(+)) 2 | | | P48735 |  |
| IDH3A | Isocitrate dehydrogenase (NAD(+)) 3 catalytic subunit alpha | | | P50213 |  |
| IDH3B | Isocitrate dehydrogenase (NAD(+)) 3 non-catalytic subunit beta | | | O43837 |  |
| IDH3G | Isocitrate dehydrogenase (NAD(+)) 3 non-catalytic subunit gamma | | | P51553 |  |
| IDI1 | Isopentenyl-diphosphate delta isomerase 1 | | | Q13907 |  |
| IDNK | IDNK gluconokinase | | | Q5T6J7 |  |
| IDS | Iduronate 2-sulfatase | | | P22304 |  |
| IDUA | Alpha-L-iduronidase | | | P35475 |  |
| IER2 | Immediate early response 2 | | | Q9BTL4 |  |
| IER3IP1 | Immediate early response 3 interacting protein 1 | | | Q9Y5U9 |  |
| IFFO1 | Intermediate filament family orphan 1 | | | Q0D2I5 |  |
| IFI16 | Interferon gamma inducible protein 16 | | | Q16666 |  |
| IFI27 | Interferon alpha inducible protein 27 | | | P40305 |  |
| IFI27L1 | Interferon alpha inducible protein 27 like 1 | | | Q96BM0 |  |
| IFI27L2 | Interferon alpha inducible protein 27 like 2 | | | Q9H2X8 |  |
| IFI30 | IFI30 lysosomal thiol reductase | | | P13284 |  |
| IFI35 | Interferon induced protein 35 | | | P80217 |  |
| IFI44 | Interferon induced protein 44 | | | Q8TCB0 |  |
| IFI44L | Interferon induced protein 44 like | | | Q53G44 |  |
| IFI6 | Interferon alpha inducible protein 6 | | | P09912 |  |
| IFIH1 | Interferon induced with helicase C domain 1 | | | Q9BYX4 |  |
| IFIT1 | Interferon induced protein with tetratricopeptide repeats 1 | | | P09914 |  |
| IFIT2 | Interferon induced protein with tetratricopeptide repeats 2 | | | P09913 |  |
| IFIT3 | Interferon induced protein with tetratricopeptide repeats 3 | | | O14879 |  |
| IFIT5 | Interferon induced protein with tetratricopeptide repeats 5 | | | Q13325 |  |
| IFITM1 | Interferon induced transmembrane protein 1 | | | P13164 |  |
| IFITM2 | Interferon induced transmembrane protein 2 | | | Q01629 |  |
| IFITM3 | Interferon induced transmembrane protein 3 | | | Q01628 |  |
| IFNAR1 | Interferon alpha and beta receptor subunit 1 | | | P17181 |  |
| IFNAR2 | Interferon alpha and beta receptor subunit 2 | | | P48551 |  |
| IFNG | Interferon gamma | | | P01579 |  |
| IFNGR1 | Interferon gamma receptor 1 | | | P15260 |  |
| IFNGR2 | Interferon gamma receptor 2 | | | P38484 |  |
| IFNL1 | Interferon lambda 1 | | | Q8IU54 |  |
| IFRD1 | Interferon related developmental regulator 1 | | | O00458 |  |
| IFRD2 | Interferon related developmental regulator 2 | | | Q12894 |  |
| IFT140 | Intraflagellar transport 140 | | | Q96RY7 |  |
| IFT20 | Intraflagellar transport 20 | | | Q8IY31 |  |
| IFT22 | Intraflagellar transport 22 | | | Q9H7X7 |  |
| IFT27 | Intraflagellar transport 27 | | | Q9BW83 |  |
| IFT43 | Intraflagellar transport 43 | | | Q96FT9 |  |
| IFT46 | Intraflagellar transport 46 | | | Q9NQC8 |  |
| IFT52 | Intraflagellar transport 52 | | | Q9Y366 |  |
| IFT57 | Intraflagellar transport 57 | | | Q9NWB7 |  |
| IFT74 | Intraflagellar transport 74 | | | Q96LB3 |  |
| IFT80 | Intraflagellar transport 80 | | | Q9P2H3 |  |
| IFT88 | Intraflagellar transport 88 | | | Q13099 |  |
| IGBP1 | Immunoglobulin binding protein 1 | | | P78318 |  |
| IGF1R | Insulin like growth factor 1 receptor | | | P08069 |  |
| IGFBP3 | Insulin like growth factor binding protein 3 | | | P17936 |  |
| IGFBP4 | Insulin like growth factor binding protein 4 | | | P22692 |  |
| IGFBP6 | Insulin like growth factor binding protein 6 | | | P24592 |  |
| IGFLR1 | IGF like family receptor 1 | | | Q9H665 |  |
| IGKC | Immunoglobulin kappa constant | | |  |  |
| IGLC2 | Immunoglobulin lambda constant 2 | | | P0DOY2 |  |
| IGSF8 | Immunoglobulin superfamily member 8 | | | Q969P0 |  |
| IK | IK cytokine | | | Q13123 |  |
| IKBIP | IKBKB interacting protein | | | Q70UQ0 |  |
| IKBKB | Inhibitor of nuclear factor kappa B kinase subunit beta | | | O14920 |  |
| IKBKE | Inhibitor of nuclear factor kappa B kinase subunit epsilon | | | Q14164 |  |
| IKZF1 | IKAROS family zinc finger 1 | | | Q13422 |  |
| IKZF3 | IKAROS family zinc finger 3 | | | Q9UKT9 |  |
| IKZF5 | IKAROS family zinc finger 5 | | | Q9H5V7 |  |
| IL10RA | Interleukin 10 receptor subunit alpha | | | Q13651 |  |
| IL10RB | Interleukin 10 receptor subunit beta | | | Q08334 |  |
| IL11RA | Interleukin 11 receptor subunit alpha | | | Q14626 |  |
| IL12RB1 | Interleukin 12 receptor subunit beta 1 | | | P42701 |  |
| IL12RB2 | Interleukin 12 receptor subunit beta 2 | | | Q99665 |  |
| IL15 | Interleukin 15 | | | P40933 |  |
| IL15RA | Interleukin 15 receptor subunit alpha | | | Q13261 |  |
| IL16 | Interleukin 16 | | | Q14005 |  |
| IL17RA | Interleukin 17 receptor A | | | Q96F46 |  |
| IL18BP | Interleukin 18 binding protein | | | O95998 |  |
| IL18R1 | Interleukin 18 receptor 1 | | | Q13478 |  |
| IL18RAP | Interleukin 18 receptor accessory protein | | | O95256 |  |
| IL1RAP | Interleukin 1 receptor accessory protein | | | Q9NPH3 |  |
| IL21R | Interleukin 21 receptor | | | Q9HBE5 |  |
| IL23A | Interleukin 23 subunit alpha | | | Q9NPF7 |  |
| IL24 | Interleukin 24 | | | Q13007 |  |
| IL27RA | Interleukin 27 receptor subunit alpha | | | Q6UWB1 |  |
| IL2RA | Interleukin 2 receptor subunit alpha | | | P01589 |  |
| IL2RB | Interleukin 2 receptor subunit beta | | | P14784 |  |
| IL2RG | Interleukin 2 receptor subunit gamma | | | P31785 |  |
| IL32 | Interleukin 32 | | | P24001 |  |
| IL4I1 | Interleukin 4 induced 1 | | | Q96RQ9 |  |
| IL4R | Interleukin 4 receptor | | | P24394 |  |
| IL5RA | Interleukin 5 receptor subunit alpha | | | Q01344 |  |
| IL6R | Interleukin 6 receptor | | | P08887 |  |
| IL6ST | Interleukin 6 cytokine family signal transducer | | | P40189 |  |
| IL6ST | Interleukin 6 cytokine family signal transducer | | | P40189 |  |
| IL7 | Interleukin 7 | | | P13232 |  |
| IL7R | Interleukin 7 receptor | | | P16871 |  |
| ILF2 | Interleukin enhancer binding factor 2 | | | Q12905 |  |
| ILF3 | Interleukin enhancer binding factor 3 | | | Q12906 |  |
| ILK | Integrin linked kinase | | | Q13418 |  |
| ILKAP | ILK associated serine/threonine phosphatase | | | Q9H0C8 |  |
| ILVBL | IlvB acetolactate synthase like | | | A1L0T0 |  |
| IMMP1L | Inner mitochondrial membrane peptidase subunit 1 | | | Q96LU5 |  |
| IMMP2L | Inner mitochondrial membrane peptidase subunit 2 | | | Q96T52 |  |
| IMMT | Inner membrane mitochondrial protein | | | Q16891 |  |
| IMP3 | IMP U3 small nucleolar ribonucleoprotein 3 | | | Q9NV31 |  |
| IMP4 | IMP U3 small nucleolar ribonucleoprotein 4 | | | Q96G21 |  |
| IMPA1 | Inositol monophosphatase 1 | | | P29218 |  |
| IMPACT | Impact RWD domain protein | | | Q9P2X3 |  |
| IMPDH1 | Inosine monophosphate dehydrogenase 1 | | | P20839 |  |
| IMPDH2 | Inosine monophosphate dehydrogenase 2 | | | P12268 |  |
| INF2 | Inverted formin 2 | | | Q27J81 |  |
| ING1 | Inhibitor of growth family member 1 | | | Q9UK53 |  |
| ING2 | Inhibitor of growth family member 2 | | | Q9H160 |  |
| ING3 | Inhibitor of growth family member 3 | | | Q9NXR8 |  |
| ING4 | Inhibitor of growth family member 4 | | | Q9UNL4 |  |
| ING5 | Inhibitor of growth family member 5 | | | Q8WYH8 |  |
| INIP | INTS3 and NABP interacting protein | | | Q9NRY2 |  |
| INO80B | INO80 complex subunit B | | | Q9C086 |  |
| INO80C | INO80 complex subunit C | | | Q6PI98 |  |
| INO80E | INO80 complex subunit E | | | Q8NBZ0 |  |
| INPP1 | Inositol polyphosphate-1-phosphatase | | | P49441 |  |
| INPP4A | Inositol polyphosphate-4-phosphatase type I A | | | Q96PE3 |  |
| INPP4B | Inositol polyphosphate-4-phosphatase type II B | | | O15327 |  |
| INPP4B | Inositol polyphosphate-4-phosphatase type II B | | | O15327 |  |
| INPP5B | Inositol polyphosphate-5-phosphatase B | | | P32019 |  |
| INPP5D | Inositol polyphosphate-5-phosphatase D | | | Q92835 |  |
| INPP5F | Inositol polyphosphate-5-phosphatase F | | | Q9Y2H2 |  |
| INPP5K | Inositol polyphosphate-5-phosphatase K | | | Q9BT40 |  |
| INSIG1 | Insulin induced gene 1 | | | O15503 |  |
| INSIG2 | Insulin induced gene 2 | | | Q9Y5U4 |  |
| INTS10 | Integrator complex subunit 10 | | | Q9NVR2 |  |
| INTS12 | Integrator complex subunit 12 | | | Q96CB8 |  |
| INTS4 | Integrator complex subunit 4 | | | Q96HW7 |  |
| INTS5 | Integrator complex subunit 5 | | | Q6P9B9 |  |
| INTS6 | Integrator complex subunit 6 | | | Q9UL03 |  |
| INTS7 | Integrator complex subunit 7 | | | Q9NVH2 |  |
| INTS9 | Integrator complex subunit 9 | | | Q9NV88 |  |
| INVS | Inversin | | | Q9Y283 |  |
| IP6K1 | Inositol hexakisphosphate kinase 1 | | | Q92551 |  |
| IP6K2 | Inositol hexakisphosphate kinase 2 | | | Q9UHH9 |  |
| IPCEF1 | Interaction protein for cytohesin exchange factors 1 | | | Q8WWN9 |  |
| IPO11 | Importin 11 | | | Q9UI26 |  |
| IPO13 | Importin 13 | | | O94829 |  |
| IPO5 | Importin 5 | | | O00410 |  |
| IPO8 | Importin 8 | | | O15397 |  |
| IPPK | Inositol-pentakisphosphate 2-kinase | | | Q9H8X2 |  |
| IQCB1 | IQ motif containing B1 | | | Q15051 |  |
| IQCC | IQ motif containing C | | | Q4KMZ1 |  |
| IQCG | IQ motif containing G | | | Q9H095 |  |
| IQCK | IQ motif containing K | | | Q8N0W5 |  |
| IQSEC1 | IQ motif and Sec7 domain ArfGEF 1 | | | Q6DN90 |  |
| IRAK2 | Interleukin 1 receptor associated kinase 2 | | | O43187 |  |
| IRAK4 | Interleukin 1 receptor associated kinase 4 | | | Q9NWZ3 |  |
| IREB2 | Iron responsive element binding protein 2 | | | P48200 |  |
| IRF1 | Interferon regulatory factor 1 | | | P10914 |  |
| IRF2 | Interferon regulatory factor 2 | | | P14316 |  |
| IRF2BP2 | Interferon regulatory factor 2 binding protein 2 | | | Q7Z5L9 |  |
| IRF3 | Interferon regulatory factor 3 | | | Q14653 |  |
| IRF4 | Interferon regulatory factor 4 | | | Q15306 |  |
| IRF5 | Interferon regulatory factor 5 | | | Q13568 |  |
| IRF7 | Interferon regulatory factor 7 | | | Q92985 |  |
| IRF8 | Interferon regulatory factor 8 | | | Q02556 |  |
| IRF9 | Interferon regulatory factor 9 | | | Q00978 |  |
| ISCA1 | Iron-sulfur cluster assembly 1 | | | Q9BUE6 |  |
| ISCA2 | Iron-sulfur cluster assembly 2 | | | Q86U28 |  |
| ISCU | Iron-sulfur cluster assembly enzyme | | | Q9H1K1 |  |
| ISG15 | ISG15 ubiquitin like modifier | | | P05161 |  |
| ISG20 | Interferon stimulated exonuclease gene 20 | | | Q96AZ6 |  |
| ISG20L2 | Interferon stimulated exonuclease gene 20 like 2 | | | Q9H9L3 |  |
| ISM1 | Isthmin 1 | | | B1AKI9 |  |
| ISOC1 | Isochorismatase domain containing 1 | | | Q96CN7 |  |
| ISOC2 | Isochorismatase domain containing 2 | | | Q96AB3 |  |
| IST1 | IST1 factor associated with ESCRT-III | | | P53990 |  |
| ISY1 | ISY1 splicing factor homolog | | | Q9ULR0 |  |
| ISYNA1 | Inositol-3-phosphate synthase 1 | | | Q9NPH2 |  |
| ITCH | Itchy E3 ubiquitin protein ligase | | | Q96J02 |  |
| ITFG1 | Integrin alpha FG-GAP repeat containing 1 | | | Q8TB96 |  |
| ITFG2 | Integrin alpha FG-GAP repeat containing 2 | | | Q969R8 |  |
| ITGA5 | Integrin subunit alpha 5 | | | P08648 |  |
| ITGA6 | Integrin subunit alpha 6 | | | P23229 |  |
| ITGB1 | Integrin subunit beta 1 | | | P05556 |  |
| ITGB1BP1 | Integrin subunit beta 1 binding protein 1 | | | O14713 |  |
| ITGB2 | Integrin subunit beta 2 | | | P05107 |  |
| ITGB3BP | Integrin subunit beta 3 binding protein | | | Q13352 |  |
| ITGB7 | Integrin subunit beta 7 | | | P26010 |  |
| ITK | IL2 inducible T cell kinase | | | Q08881 |  |
| ITM2A | Integral membrane protein 2A | | | O43736 |  |
| ITM2B | Integral membrane protein 2B | | | Q9Y287 |  |
| ITM2C | Integral membrane protein 2C | | | Q9NQX7 |  |
| ITPA | Inosine triphosphatase | | | Q9BY32 |  |
| ITPK1 | Inositol-tetrakisphosphate 1-kinase | | | Q13572 |  |
| ITPKB | Inositol-trisphosphate 3-kinase B | | | P27987 |  |
| ITPRIP | Inositol 1,4,5-trisphosphate receptor interacting protein | | | Q8IWB1 |  |
| IVD | Isovaleryl-CoA dehydrogenase | | | P26440 |  |
| IVNS1ABP | Influenza virus NS1A binding protein | | | Q9Y6Y0 |  |
| IZUMO4 | IZUMO family member 4 | | | Q1ZYL8 |  |
| JADE1 | Jade family PHD finger 1 | | | Q6IE81 |  |
| JADE2 | Jade family PHD finger 2 | | | Q9NQC1 |  |
| JAGN1 | Jagunal homolog 1 | | | Q8N5M9 |  |
| JAK1 | Janus kinase 1 | | | P23458 |  |
| JAK2 | Janus kinase 2 | | | O60674 |  |
| JAK3 | Janus kinase 3 | | | P52333 |  |
| JAKMIP1 | Janus kinase and microtubule interacting protein 1 | | | Q96N16 |  |
| JAKMIP2 | Janus kinase and microtubule interacting protein 2 | | | Q96AA8 |  |
| JAM3 | Junctional adhesion molecule 3 | | | Q9BX67 |  |
| JARID2 | Jumonji and AT-rich interaction domain containing 2 | | | Q92833 |  |
| JAZF1 | JAZF zinc finger 1 | | | Q86VZ6 |  |
| JCHAIN | Joining chain of multimeric IgA and IgM | | | P01591 |  |
| JKAMP | JNK1/MAPK8 associated membrane protein | | | Q9P055 |  |
| JMJD6 | Jumonji domain containing 6, arginine demethylase and lysine hydroxylase | | | Q6NYC1 |  |
| JMJD7 | Jumonji domain containing 7 | | | P0C870 |  |
| JMJD8 | Jumonji domain containing 8 | | | Q96S16 |  |
| JOSD1 | Josephin domain containing 1 | | | Q15040 |  |
| JOSD2 | Josephin domain containing 2 | | | Q8TAC2 |  |
| JRK | Jrk helix-turn-helix protein | | | O75564 |  |
| JTB | Jumping translocation breakpoint | | | O76095 |  |
| JUNB | JunB proto-oncogene, AP-1 transcription factor subunit | | | P17275 |  |
| JUND | JunD proto-oncogene, AP-1 transcription factor subunit | | | P17535 |  |
| KANK1 | KN motif and ankyrin repeat domains 1 | | | Q14678 |  |
| KANSL3 | KAT8 regulatory NSL complex subunit 3 | | | Q9P2N6 |  |
| KAT2B | Lysine acetyltransferase 2B | | | Q92831 |  |
| KAT5 | Lysine acetyltransferase 5 | | | Q92993 |  |
| KAT7 | Lysine acetyltransferase 7 | | | O95251 |  |
| KAT8 | Lysine acetyltransferase 8 | | | Q9H7Z6 |  |
| KATNA1 | Katanin catalytic subunit A1 | | | O75449 |  |
| KATNAL1 | Katanin catalytic subunit A1 like 1 | | | Q9BW62 |  |
| KATNB1 | Katanin regulatory subunit B1 | | | Q9BVA0 |  |
| KATNBL1 | Katanin regulatory subunit B1 like 1 | | | Q9H079 |  |
| KBTBD3 | Kelch repeat and BTB domain containing 3 | | | Q8NAB2 |  |
| KBTBD4 | Kelch repeat and BTB domain containing 4 | | | Q9NVX7 |  |
| KBTBD6 | Kelch repeat and BTB domain containing 6 | | | Q86V97 |  |
| KBTBD7 | Kelch repeat and BTB domain containing 7 | | | Q8WVZ9 |  |
| KCMF1 | Potassium channel modulatory factor 1 | | | Q9P0J7 |  |
| KCNA3 | Potassium voltage-gated channel subfamily A member 3 | | | P22001 |  |
| KCNAB2 | Potassium voltage-gated channel subfamily A regulatory beta subunit 2 | | | Q13303 |  |
| KCNK6 | Potassium two pore domain channel subfamily K member 6 | | | Q9Y257 |  |
| KCNN4 | Potassium calcium-activated channel subfamily N member 4 | | | O15554 |  |
| KCNQ1 | Potassium voltage-gated channel subfamily Q member 1 | | | P51787 |  |
| KCTD10 | Potassium channel tetramerization domain containing 10 | | | Q9H3F6 |  |
| KCTD13 | Potassium channel tetramerization domain containing 13 | | | Q8WZ19 |  |
| KCTD18 | Potassium channel tetramerization domain containing 18 | | | Q6PI47 |  |
| KCTD21 | Potassium channel tetramerization domain containing 21 | | | Q4G0X4 |  |
| KCTD5 | Potassium channel tetramerization domain containing 5 | | | Q9NXV2 |  |
| KCTD6 | Potassium channel tetramerization domain containing 6 | | | Q8NC69 |  |
| KCTD7 | Potassium channel tetramerization domain containing 7 | | | Q96MP8 |  |
| KCTD9 | Potassium channel tetramerization domain containing 9 | | | Q7L273 |  |
| KDELR1 | KDEL endoplasmic reticulum protein retention receptor 1 | | | P24390 |  |
| KDELR2 | KDEL endoplasmic reticulum protein retention receptor 2 | | | P33947 |  |
| KDM1A | Lysine demethylase 1A | | | O60341 |  |
| KDM2A | Lysine demethylase 2A | | | Q9Y2K7 |  |
| KDM3A | Lysine demethylase 3A | | | Q9Y4C1 |  |
| KDM3B | Lysine demethylase 3B | | | Q7LBC6 |  |
| KDM4A | Lysine demethylase 4A | | | O75164 |  |
| KDM4C | Lysine demethylase 4C | | | Q9H3R0 |  |
| KDM5A | Lysine demethylase 5A | | | P29375 |  |
| KDM5C | Lysine demethylase 5C | | | P41229 |  |
| KDM5D | Lysine demethylase 5D | | | Q9BY66 |  |
| KDM6A | Lysine demethylase 6A | | | O15550 |  |
| KDM8 | Lysine demethylase 8 | | | Q8N371 |  |
| KDSR | 3-ketodihydrosphingosine reductase | | | Q06136 |  |
| KEAP1 | Kelch like ECH associated protein 1 | | | Q14145 |  |
| KHDC1 | KH domain containing 1 | | | Q4VXA5 |  |
| KHDRBS1 | KH RNA binding domain containing, signal transduction associated 1 | | | Q07666 |  |
| KHK | Ketohexokinase | | | P50053 |  |
| KHNYN | KH and NYN domain containing | | | O15037 |  |
| KHSRP | KH-type splicing regulatory protein | | | Q92945 |  |
| KIAA0040 | KIAA0040 | | | Q15053 |  |
| KIAA0319L | KIAA0319 like | | | Q8IZA0 |  |
| KIAA0586 | KIAA0586 | | | Q9BVV6 |  |
| KIAA1143 | KIAA1143 | | | Q96AT1 |  |
| KIAA1191 | KIAA1191 | | | Q96A73 |  |
| KIAA1328 | KIAA1328 | | | Q86T90 |  |
| KIAA1586 | KIAA1586 | | | Q9HCI6 |  |
| KIAA2013 | KIAA2013 | | | Q8IYS2 |  |
| KIF16B | Kinesin family member 16B | | | Q96L93 |  |
| KIF20B | Kinesin family member 20B | | | Q96Q89 |  |
| KIF22 | Kinesin family member 22 | | | Q14807 |  |
| KIF2A | Kinesin family member 2A | | | O00139 |  |
| KIF2C | Kinesin family member 2C | | | Q99661 |  |
| KIF3A | Kinesin family member 3A | | | Q9Y496 |  |
| KIF3B | Kinesin family member 3B | | | O15066 |  |
| KIF9 | Kinesin family member 9 | | | Q9HAQ2 |  |
| KIFAP3 | Kinesin associated protein 3 | | | Q92845 |  |
| KIN | Kin17 DNA and RNA binding protein | | | O60870 |  |
| KIZ | Kizuna centrosomal protein | | | Q2M2Z5 |  |
| KLC1 | Kinesin light chain 1 | | | Q07866 |  |
| KLC2 | Kinesin light chain 2 | | | Q9H0B6 |  |
| KLC4 | Kinesin light chain 4 | | | Q9NSK0 |  |
| KLF10 | KLF transcription factor 10 | | | Q13118 |  |
| KLF11 | KLF transcription factor 11 | | | O14901 |  |
| KLF12 | KLF transcription factor 12 | | | Q9Y4X4 |  |
| KLF13 | KLF transcription factor 13 | | | Q9Y2Y9 |  |
| KLF15 | KLF transcription factor 15 | | | Q9UIH9 |  |
| KLF2 | KLF transcription factor 2 | | | Q9Y5W3 |  |
| KLF3 | KLF transcription factor 3 | | | P57682 |  |
| KLF6 | KLF transcription factor 6 | | | Q99612 |  |
| KLF7 | KLF transcription factor 7 | | | O75840 |  |
| KLHDC1 | Kelch domain containing 1 | | | Q8N7A1 |  |
| KLHDC2 | Kelch domain containing 2 | | | Q9Y2U9 |  |
| KLHDC3 | Kelch domain containing 3 | | | Q9BQ90 |  |
| KLHDC4 | Kelch domain containing 4 | | | Q8TBB5 |  |
| KLHDC7B | Kelch domain containing 7B | | | Q96G42 |  |
| KLHL12 | Kelch like family member 12 | | | Q53G59 |  |
| KLHL18 | Kelch like family member 18 | | | O94889 |  |
| KLHL20 | Kelch like family member 20 | | | Q9Y2M5 |  |
| KLHL22 | Kelch like family member 22 | | | Q53GT1 |  |
| KLHL23 | Kelch like family member 23 | | | Q8NBE8 |  |
| KLHL25 | Kelch like family member 25 | | | Q9H0H3 |  |
| KLHL26 | Kelch like family member 26 | | | Q53HC5 |  |
| KLHL32 | Kelch like family member 32 | | | Q96NJ5 |  |
| KLHL36 | Kelch like family member 36 | | | Q8N4N3 |  |
| KLHL42 | Kelch like family member 42 | | | Q9P2K6 |  |
| KLHL6 | Kelch like family member 6 | | | Q8WZ60 |  |
| KLHL7 | Kelch like family member 7 | | | Q8IXQ5 |  |
| KLHL8 | Kelch like family member 8 | | | Q9P2G9 |  |
| KLHL9 | Kelch like family member 9 | | | Q9P2J3 |  |
| KLRB1 | Killer cell lectin like receptor B1 | | | Q12918 |  |
| KLRD1 | Killer cell lectin like receptor D1 | | | Q13241 |  |
| KLRF1 | Killer cell lectin like receptor F1 | | | Q9NZS2 |  |
| KLRG1 | Killer cell lectin like receptor G1 | | | Q96E93 |  |
| KLRK1 | Killer cell lectin like receptor K1 | | | P26718 |  |
| KMT2E | Lysine methyltransferase 2E (inactive) | | | Q8IZD2 |  |
| KNOP1 | Lysine rich nucleolar protein 1 | | | Q1ED39 |  |
| KNSTRN | Kinetochore localized astrin (SPAG5) binding protein | | | Q9Y448 |  |
| KPNA1 | Karyopherin subunit alpha 1 | | | P52294 |  |
| KPNA2 | Karyopherin subunit alpha 2 | | | P52292 |  |
| KPNA3 | Karyopherin subunit alpha 3 | | | O00505 |  |
| KPNA4 | Karyopherin subunit alpha 4 | | | O00629 |  |
| KPNA5 | Karyopherin subunit alpha 5 | | | O15131 |  |
| KPNA6 | Karyopherin subunit alpha 6 | | | O60684 |  |
| KPNB1 | Karyopherin subunit beta 1 | | | Q14974 |  |
| KPTN | Kaptin, actin binding protein | | | Q9Y664 |  |
| KRAS | KRAS proto-oncogene, GTPase | | | P01116 |  |
| KRBA2 | KRAB-A domain containing 2 | | | Q6ZNG9 |  |
| KRBOX4 | KRAB box domain containing 4 | | | Q5JUW0 |  |
| KRCC1 | Lysine rich coiled-coil 1 | | | Q9NPI7 |  |
| KRI1 | KRI1 homolog | | | Q8N9T8 |  |
| KRIT1 | KRIT1 ankyrin repeat containing | | | O00522 |  |
| KRR1 | KRR1 small subunit processome component homolog | | | Q13601 |  |
| KRT1 | Keratin 1 | | | P04264 |  |
| KRT10 | Keratin 10 | | | P13645 |  |
| KRT18 | Keratin 18 | | | P05783 |  |
| KRT2 | Keratin 2 | | | P35908 |  |
| KRTCAP2 | Keratinocyte associated protein 2 | | | Q8N6L1 |  |
| KRTCAP3 | Keratinocyte associated protein 3 | | | Q53RY4 |  |
| KTI12 | KTI12 chromatin associated homolog | | | Q96EK9 |  |
| KTN1 | Kinectin 1 | | | Q86UP2 |  |
| KXD1 | KxDL motif containing 1 | | | Q9BQD3 |  |
| L2HGDH | L-2-hydroxyglutarate dehydrogenase | | | Q9H9P8 |  |
| L3HYPDH | Trans-L-3-hydroxyproline dehydratase | | | Q96EM0 |  |
| L3MBTL2 | L3MBTL histone methyl-lysine binding protein 2 | | | Q969R5 |  |
| L3MBTL3 | L3MBTL histone methyl-lysine binding protein 3 | | | Q96JM7 |  |
| LACTB2 | Lactamase beta 2 | | | Q53H82 |  |
| LAG3 | Lymphocyte activating 3 | | | P18627 |  |
| LAGE3 | L antigen family member 3 | | | Q14657 |  |
| LAIR1 | Leukocyte associated immunoglobulin like receptor 1 | | | Q6GTX8 |  |
| LAIR2 | Leukocyte associated immunoglobulin like receptor 2 | | | Q6ISS4 |  |
| LAIR2 | Leukocyte associated immunoglobulin like receptor 2 | | | Q6ISS4 |  |
| LAMP1 | Lysosomal associated membrane protein 1 | | | P11279 |  |
| LAMP2 | Lysosomal associated membrane protein 2 | | | P13473 |  |
| LAMP3 | Lysosomal associated membrane protein 3 | | | Q9UQV4 |  |
| LAMTOR1 | Late endosomal/lysosomal adaptor, MAPK and MTOR activator 1 | | | Q6IAA8 |  |
| LAMTOR2 | Late endosomal/lysosomal adaptor, MAPK and MTOR activator 2 | | | Q9Y2Q5 |  |
| LAMTOR3 | Late endosomal/lysosomal adaptor, MAPK and MTOR activator 3 | | | Q9UHA4 |  |
| LAMTOR4 | Late endosomal/lysosomal adaptor, MAPK and MTOR activator 4 | | | Q0VGL1 |  |
| LAMTOR5 | Late endosomal/lysosomal adaptor, MAPK and MTOR activator 5 | | | O43504 |  |
| LANCL1 | LanC like glutathione S-transferase 1 | | | O43813 |  |
| LANCL2 | LanC like glutathione S-transferase 2 | | | Q9NS86 |  |
| LAP3 | Leucine aminopeptidase 3 | | | P28838 |  |
| LAPTM4A | Lysosomal protein transmembrane 4 alpha | | | Q15012 |  |
| LAPTM5 | Lysosomal protein transmembrane 5 | | | Q13571 |  |
| LARP1 | La ribonucleoprotein 1, translational regulator | | | Q6PKG0 |  |
| LARP1B | La ribonucleoprotein 1B | | | Q659C4 |  |
| LARP4 | La ribonucleoprotein 4 | | | Q71RC2 |  |
| LARP4B | La ribonucleoprotein 4B | | | Q92615 |  |
| LARP7 | La ribonucleoprotein 7, transcriptional regulator | | | Q4G0J3 |  |
| LAS1L | LAS1 like ribosome biogenesis factor | | | Q9Y4W2 |  |
| LASP1 | LIM and SH3 protein 1 | | | Q14847 |  |
| LAT | Linker for activation of T cells | | | O43561 |  |
| LAT2 | Linker for activation of T cells family member 2 | | | Q9GZY6 |  |
| LATS1 | Large tumor suppressor kinase 1 | | | O95835 |  |
| LAX1 | Lymphocyte transmembrane adaptor 1 | | | Q8IWV1 |  |
| LBH | LBH regulator of WNT signaling pathway | | | Q53QV2 |  |
| LBHD1 | LBH domain containing 1 | | | Q9BQE6 |  |
| LBR | Lamin B receptor | | | Q14739 |  |
| LCK | LCK proto-oncogene, Src family tyrosine kinase | | | P06239 |  |
| LCMT1 | Leucine carboxyl methyltransferase 1 | | | Q9UIC8 |  |
| LCMT2 | Leucine carboxyl methyltransferase 2 | | | O60294 |  |
| LCOR | Ligand dependent nuclear receptor corepressor | | | Q96JN0 |  |
| LCORL | Ligand dependent nuclear receptor corepressor like | | | Q8N3X6 |  |
| LCP1 | Lymphocyte cytosolic protein 1 | | | P13796 |  |
| LCP2 | Lymphocyte cytosolic protein 2 | | | Q13094 |  |
| LDAH | Lipid droplet associated hydrolase | | | Q9H6V9 |  |
| LDB1 | LIM domain binding 1 | | | Q86U70 |  |
| LDHA | Lactate dehydrogenase A | | | P00338 |  |
| LDHB | Lactate dehydrogenase B | | | P07195 |  |
| LDHC | Lactate dehydrogenase C | | | P07864 |  |
| LDLR | Low density lipoprotein receptor | | | P01130 |  |
| LDLRAD4 | Low density lipoprotein receptor class A domain containing 4 | | | O15165 |  |
| LDOC1 | LDOC1 regulator of NFKB signaling | | | O95751 |  |
| LEAP2 | Liver enriched antimicrobial peptide 2 | | | Q969E1 |  |
| LEF1 | Lymphoid enhancer binding factor 1 | | | Q9UJU2 |  |
| LEF1 | Lymphoid enhancer binding factor 1 | | | Q9UJU2 |  |
| LEMD3 | LEM domain containing 3 | | | Q9Y2U8 |  |
| LEPROT | Leptin receptor overlapping transcript | | | O15243 |  |
| LEPROTL1 | Leptin receptor overlapping transcript like 1 | | | O95214 |  |
| LETM1 | Leucine zipper and EF-hand containing transmembrane protein 1 | | | O95202 |  |
| LETM2 | Leucine zipper and EF-hand containing transmembrane protein 2 | | | Q2VYF4 |  |
| LETMD1 | LETM1 domain containing 1 | | | Q6P1Q0 |  |
| LFNG | LFNG O-fucosylpeptide 3-beta-N-acetylglucosaminyltransferase | | | Q8NES3 |  |
| LGALS1 | Galectin 1 | | | P09382 |  |
| LGALS2 | Galectin 2 | | | P05162 |  |
| LGALS3 | Galectin 3 | | | P17931 |  |
| LGALS3BP | Galectin 3 binding protein | | | Q08380 |  |
| LGALS8 | Galectin 8 | | | O00214 |  |
| LGALS9 | Galectin 9 | | | O00182 |  |
| LGMN | Legumain | | | Q99538 |  |
| LGR6 | Leucine rich repeat containing G protein-coupled receptor 6 | | | Q9HBX8 |  |
| LHPP | Phospholysine phosphohistidine inorganic pyrophosphate phosphatase | | | Q9H008 |  |
| LIAS | Lipoic acid synthetase | | | O43766 |  |
| LIG1 | DNA ligase 1 | | | P18858 |  |
| LIG3 | DNA ligase 3 | | | P49916 |  |
| LIG4 | DNA ligase 4 | | | P49917 |  |
| LIMA1 | LIM domain and actin binding 1 | | | Q9UHB6 |  |
| LIMD2 | LIM domain containing 2 | | | Q9BT23 |  |
| LIME1 | Lck interacting transmembrane adaptor 1 | | | Q9H400 |  |
| LIMK2 | LIM domain kinase 2 | | | P53671 |  |
| LIMS1 | LIM zinc finger domain containing 1 | | | P48059 |  |
| LIN37 | Lin-37 DREAM MuvB core complex component | | | Q96GY3 |  |
| LIN52 | Lin-52 DREAM MuvB core complex component | | |  |  |
| LIN54 | Lin-54 DREAM MuvB core complex component | | | Q6MZP7 |  |
| LIN7B | Lin-7 homolog B, crumbs cell polarity complex component | | | Q9HAP6 |  |
| LIN7C | Lin-7 homolog C, crumbs cell polarity complex component | | | Q9NUP9 |  |
| LIN9 | Lin-9 DREAM MuvB core complex component | | | Q5TKA1 |  |
| LINS1 | Lines homolog 1 | | | Q8NG48 |  |
| LIPA | Lipase A, lysosomal acid type | | | P38571 |  |
| LIPT1 | Lipoyltransferase 1 | | | Q9Y234 |  |
| LITAF | Lipopolysaccharide induced TNF factor | | | Q99732 |  |
| LIX1L | Limb and CNS expressed 1 like | | | Q8IVB5 |  |
| LLPH | LLP homolog, long-term synaptic facilitation factor | | | Q9BRT6 |  |
| LMAN1 | Lectin, mannose binding 1 | | | P49257 |  |
| LMAN2 | Lectin, mannose binding 2 | | | Q12907 |  |
| LMAN2L | Lectin, mannose binding 2 like | | | Q9H0V9 |  |
| LMBR1L | Limb development membrane protein 1 like | | | Q6UX01 |  |
| LMBRD1 | LMBR1 domain containing 1 | | | Q9NUN5 |  |
| LMBRD2 | LMBR1 domain containing 2 | | | Q68DH5 |  |
| LMCD1 | LIM and cysteine rich domains 1 | | | Q9NZU5 |  |
| LMF1 | Lipase maturation factor 1 | | | Q96S06 |  |
| LMF2 | Lipase maturation factor 2 | | | Q9BU23 |  |
| LMNA | Lamin A/C | | | P02545 |  |
| LMNB1 | Lamin B1 | | | P20700 |  |
| LMNB2 | Lamin B2 | | | Q03252 |  |
| LMO4 | LIM domain only 4 | | | P61968 |  |
| LNPEP | Leucyl and cystinyl aminopeptidase | | | Q9UIQ6 |  |
| LONP1 | Lon peptidase 1, mitochondrial | | | P36776 |  |
| LOXL1 | Lysyl oxidase like 1 | | | Q08397 |  |
| LPAR2 | Lysophosphatidic acid receptor 2 | | | Q9HBW0 |  |
| LPAR5 | Lysophosphatidic acid receptor 5 | | | Q9H1C0 |  |
| LPAR6 | Lysophosphatidic acid receptor 6 | | | P43657 |  |
| LPCAT3 | Lysophosphatidylcholine acyltransferase 3 | | | Q6P1A2 |  |
| LPCAT4 | Lysophosphatidylcholine acyltransferase 4 | | | Q643R3 |  |
| LPIN1 | Lipin 1 | | | Q14693 |  |
| LPIN2 | Lipin 2 | | | Q92539 |  |
| LPXN | Leupaxin | | | O60711 |  |
| LRCH1 | Leucine rich repeats and calponin homology domain containing 1 | | | Q9Y2L9 |  |
| LRCH3 | Leucine rich repeats and calponin homology domain containing 3 | | | Q96II8 |  |
| LRCH4 | Leucine rich repeats and calponin homology domain containing 4 | | | O75427 |  |
| LRP10 | LDL receptor related protein 10 | | | Q7Z4F1 |  |
| LRP12 | LDL receptor related protein 12 | | | Q9Y561 |  |
| LRPAP1 | LDL receptor related protein associated protein 1 | | | P30533 |  |
| LRPPRC | Leucine rich pentatricopeptide repeat containing | | | P42704 |  |
| LRR1 | Leucine rich repeat protein 1 | | | Q96L50 |  |
| LRRC14 | Leucine rich repeat containing 14 | | | Q15048 |  |
| LRRC20 | Leucine rich repeat containing 20 | | | Q8TCA0 |  |
| LRRC23 | Leucine rich repeat containing 23 | | | Q53EV4 |  |
| LRRC27 | Leucine rich repeat containing 27 | | | Q9C0I9 |  |
| LRRC28 | Leucine rich repeat containing 28 | | | Q86X40 |  |
| LRRC37B | Leucine rich repeat containing 37B | | | Q96QE4 |  |
| LRRC40 | Leucine rich repeat containing 40 | | | Q9H9A6 |  |
| LRRC42 | Leucine rich repeat containing 42 | | | Q9Y546 |  |
| LRRC45 | Leucine rich repeat containing 45 | | | Q96CN5 |  |
| LRRC47 | Leucine rich repeat containing 47 | | | Q8N1G4 |  |
| LRRC57 | Leucine rich repeat containing 57 | | | Q8N9N7 |  |
| LRRC58 | Leucine rich repeat containing 58 | | | Q96CX6 |  |
| LRRC59 | Leucine rich repeat containing 59 | | | Q96AG4 |  |
| LRRC61 | Leucine rich repeat containing 61 | | | Q9BV99 |  |
| LRRFIP1 | LRR binding FLII interacting protein 1 | | | Q32MZ4 |  |
| LRRFIP2 | LRR binding FLII interacting protein 2 | | | Q9Y608 |  |
| LRRIQ3 | Leucine rich repeats and IQ motif containing 3 | | | A6PVS8 |  |
| LRRN3 | Leucine rich repeat neuronal 3 | | | Q9H3W5 |  |
| LRRN3 | Leucine rich repeat neuronal 3 | | | Q9H3W5 |  |
| LRSAM1 | Leucine rich repeat and sterile alpha motif containing 1 | | | Q6UWE0 |  |
| LRWD1 | Leucine rich repeats and WD repeat domain containing 1 | | | Q9UFC0 |  |
| LSG1 | Large 60S subunit nuclear export GTPase 1 | | | Q9H089 |  |
| LSM1 | LSM1 homolog, mRNA degradation associated | | | O15116 |  |
| LSM10 | LSM10, U7 small nuclear RNA associated | | | Q969L4 |  |
| LSM12 | LSM12 homolog | | | Q3MHD2 |  |
| LSM14A | LSM14A mRNA processing body assembly factor | | | Q8ND56 |  |
| LSM2 | LSM2 homolog, U6 small nuclear RNA and mRNA degradation associated | | | Q9Y333 |  |
| LSM3 | LSM3 homolog, U6 small nuclear RNA and mRNA degradation associated | | | P62310 |  |
| LSM4 | LSM4 homolog, U6 small nuclear RNA and mRNA degradation associated | | | Q9Y4Z0 |  |
| LSM5 | LSM5 homolog, U6 small nuclear RNA and mRNA degradation associated | | | Q9Y4Y9 |  |
| LSM6 | LSM6 homolog, U6 small nuclear RNA and mRNA degradation associated | | | P62312 |  |
| LSM7 | LSM7 homolog, U6 small nuclear RNA and mRNA degradation associated | | | Q9UK45 |  |
| LSM8 | LSM8 homolog, U6 small nuclear RNA associated | | | O95777 |  |
| LSP1 | Lymphocyte specific protein 1 | | | P33241 |  |
| LSR | Lipolysis stimulated lipoprotein receptor | | | Q86X29 |  |
| LSS | Lanosterol synthase | | | P48449 |  |
| LST1 | Leukocyte specific transcript 1 | | | O00453 |  |
| LTA4H | Leukotriene A4 hydrolase | | | P09960 |  |
| LTB | Lymphotoxin beta | | | Q06643 |  |
| LTB4R | Leukotriene B4 receptor | | | Q15722 |  |
| LTBP3 | Latent transforming growth factor beta binding protein 3 | | | Q9NS15 |  |
| LTBP4 | Latent transforming growth factor beta binding protein 4 | | | Q8N2S1 |  |
| LTC4S | Leukotriene C4 synthase | | | Q16873 |  |
| LTV1 | LTV1 ribosome biogenesis factor | | | Q96GA3 |  |
| LUC7L | LUC7 like | | | Q9NQ29 |  |
| LUC7L2 | LUC7 like 2, pre-mRNA splicing factor | | | Q9Y383 |  |
| LUC7L3 | LUC7 like 3 pre-mRNA splicing factor | | | O95232 |  |
| LUZP1 | Leucine zipper protein 1 | | | Q86V48 |  |
| LXN | Latexin | | | Q9BS40 |  |
| LY6E | Lymphocyte antigen 6 family member E | | | Q16553 |  |
| LY75 | Lymphocyte antigen 75 | | | O60449 |  |
| LY86 | Lymphocyte antigen 86 | | | O95711 |  |
| LY9 | Lymphocyte antigen 9 | | | Q9HBG7 |  |
| LY96 | Lymphocyte antigen 96 | | | Q9Y6Y9 |  |
| LYAR | Ly1 antibody reactive | | | Q9NX58 |  |
| LYPD3 | LY6/PLAUR domain containing 3 | | | O95274 |  |
| LYPD3 | LY6/PLAUR domain containing 3 | | | O95274 |  |
| LYPLA1 | Lysophospholipase 1 | | | O75608 |  |
| LYPLA2 | Lysophospholipase 2 | | | O95372 |  |
| LYPLAL1 | Lysophospholipase like 1 | | | Q5VWZ2 |  |
| LYRM1 | LYR motif containing 1 | | | O43325 |  |
| LYRM2 | LYR motif containing 2 | | | Q9NU23 |  |
| LYRM4 | LYR motif containing 4 | | | Q9HD34 |  |
| LYRM7 | LYR motif containing 7 | | | Q5U5X0 |  |
| LYSMD1 | LysM domain containing 1 | | | Q96S90 |  |
| LYSMD2 | LysM domain containing 2 | | | Q8IV50 |  |
| LYSMD3 | LysM domain containing 3 | | | Q7Z3D4 |  |
| LYSMD4 | LysM domain containing 4 | | | Q5XG99 |  |
| LYST | Lysosomal trafficking regulator | | | Q99698 |  |
| LYZ | Lysozyme | | | P61626 |  |
| LZIC | Leucine zipper and CTNNBIP1 domain containing | | | Q8WZA0 |  |
| LZTS2 | Leucine zipper tumor suppressor 2 | | | Q9BRK4 |  |
| M6PR | Mannose-6-phosphate receptor, cation dependent | | | P20645 |  |
| MAD1L1 | Mitotic arrest deficient 1 like 1 | | | Q9Y6D9 |  |
| MAD2L1 | Mitotic arrest deficient 2 like 1 | | | Q13257 |  |
| MAD2L1BP | MAD2L1 binding protein | | | Q15013 |  |
| MAD2L2 | Mitotic arrest deficient 2 like 2 | | | Q9UI95 |  |
| MAEA | Macrophage erythroblast attacher, E3 ubiquitin ligase | | | Q7L5Y9 |  |
| MAF | MAF bZIP transcription factor | | | O75444 |  |
| MAF1 | MAF1 homolog, negative regulator of RNA polymerase III | | | Q9H063 |  |
| MAFF | MAF bZIP transcription factor F | | | Q9ULX9 |  |
| MAGED1 | MAGE family member D1 | | | Q9Y5V3 |  |
| MAGED2 | MAGE family member D2 | | | Q9UNF1 |  |
| MAGED4B | MAGE family member D4B | | | Q96JG8 |  |
| MAGEF1 | MAGE family member F1 | | | Q9HAY2 |  |
| MAGEH1 | MAGE family member H1 | | | Q9H213 |  |
| MAGOH | Mago homolog, exon junction complex subunit | | | P61326 |  |
| MAGOHB | Mago homolog B, exon junction complex subunit | | | Q96A72 |  |
| MAGT1 | Magnesium transporter 1 | | | Q9H0U3 |  |
| MAK16 | MAK16 homolog | | | Q9BXY0 |  |
| MAL | Mal, T cell differentiation protein | | | P21145 |  |
| MALSU1 | Mitochondrial assembly of ribosomal large subunit 1 | | | Q96EH3 |  |
| MALT1 | MALT1 paracaspase | | | Q9UDY8 |  |
| MAML2 | Mastermind like transcriptional coactivator 2 | | | Q8IZL2 |  |
| MAN1A2 | Mannosidase alpha class 1A member 2 | | | O60476 |  |
| MAN1B1 | Mannosidase alpha class 1B member 1 | | | Q9UKM7 |  |
| MAN1C1 | Mannosidase alpha class 1C member 1 | | | Q9NR34 |  |
| MAN1C1 | Mannosidase alpha class 1C member 1 | | | Q9NR34 |  |
| MAN2B1 | Mannosidase alpha class 2B member 1 | | | O00754 |  |
| MAN2B2 | Mannosidase alpha class 2B member 2 | | | Q9Y2E5 |  |
| MAN2C1 | Mannosidase alpha class 2C member 1 | | | Q9NTJ4 |  |
| MANBAL | Mannosidase beta like | | | Q9NQG1 |  |
| MANEA | Mannosidase endo-alpha | | | Q5SRI9 |  |
| MANF | Mesencephalic astrocyte derived neurotrophic factor | | | P55145 |  |
| MANSC1 | MANSC domain containing 1 | | | Q9H8J5 |  |
| MAP10 | Microtubule associated protein 10 | | | Q9P2G4 |  |
| MAP1LC3A | Microtubule associated protein 1 light chain 3 alpha | | | Q9H492 |  |
| MAP1LC3B | Microtubule associated protein 1 light chain 3 beta | | | Q9GZQ8 |  |
| MAP1LC3B2 | Microtubule associated protein 1 light chain 3 beta 2 | | | A6NCE7 |  |
| MAP1S | Microtubule associated protein 1S | | | Q66K74 |  |
| MAP2K2 | Mitogen-activated protein kinase kinase 2 | | | P36507 |  |
| MAP2K3 | Mitogen-activated protein kinase kinase 3 | | | P46734 |  |
| MAP2K4 | Mitogen-activated protein kinase kinase 4 | | | P45985 |  |
| MAP2K5 | Mitogen-activated protein kinase kinase 5 | | | Q13163 |  |
| MAP2K6 | Mitogen-activated protein kinase kinase 6 | | | P52564 |  |
| MAP3K11 | Mitogen-activated protein kinase kinase kinase 11 | | | Q16584 |  |
| MAP3K13 | Mitogen-activated protein kinase kinase kinase 13 | | | O43283 |  |
| MAP3K14 | Mitogen-activated protein kinase kinase kinase 14 | | | Q99558 |  |
| MAP3K2 | Mitogen-activated protein kinase kinase kinase 2 | | | Q9Y2U5 |  |
| MAP3K5 | Mitogen-activated protein kinase kinase kinase 5 | | | Q99683 |  |
| MAP3K6 | Mitogen-activated protein kinase kinase kinase 6 | | | O95382 |  |
| MAP3K7 | Mitogen-activated protein kinase kinase kinase 7 | | | O43318 |  |
| MAP3K7CL | MAP3K7 C-terminal like | | | P57077 |  |
| MAP3K8 | Mitogen-activated protein kinase kinase kinase 8 | | | P41279 |  |
| MAP4 | Microtubule associated protein 4 | | | P27816 |  |
| MAP4K1 | Mitogen-activated protein kinase kinase kinase kinase 1 | | | Q92918 |  |
| MAP4K2 | Mitogen-activated protein kinase kinase kinase kinase 2 | | | Q12851 |  |
| MAP4K4 | Mitogen-activated protein kinase kinase kinase kinase 4 | | | O95819 |  |
| MAP9 | Microtubule associated protein 9 | | | Q49MG5 |  |
| MAPK1 | Mitogen-activated protein kinase 1 | | | P28482 |  |
| MAPK10 | Mitogen-activated protein kinase 10 | | | P53779 |  |
| MAPK13 | Mitogen-activated protein kinase 13 | | | O15264 |  |
| MAPK14 | Mitogen-activated protein kinase 14 | | | Q16539 |  |
| MAPK1IP1L | Mitogen-activated protein kinase 1 interacting protein 1 like | | | Q8NDC0 |  |
| MAPK3 | Mitogen-activated protein kinase 3 | | | P27361 |  |
| MAPK6 | Mitogen-activated protein kinase 6 | | | Q16659 |  |
| MAPK7 | Mitogen-activated protein kinase 7 | | | Q13164 |  |
| MAPK8 | Mitogen-activated protein kinase 8 | | | P45983 |  |
| MAPK9 | Mitogen-activated protein kinase 9 | | | P45984 |  |
| MAPKAP1 | MAPK associated protein 1 | | | Q9BPZ7 |  |
| MAPKAPK2 | MAPK activated protein kinase 2 | | | P49137 |  |
| MAPKAPK3 | MAPK activated protein kinase 3 | | | Q16644 |  |
| MAPKAPK5 | MAPK activated protein kinase 5 | | | Q8IW41 |  |
| MAPRE1 | Microtubule associated protein RP/EB family member 1 | | | Q15691 |  |
| MAPRE2 | Microtubule associated protein RP/EB family member 2 | | | Q15555 |  |
| MARCHF2 | Membrane associated ring-CH-type finger 2 | | | Q9P0N8 |  |
| MARCHF3 | Membrane associated ring-CH-type finger 3 | | | Q86UD3 |  |
| MARCHF5 | Membrane associated ring-CH-type finger 5 | | | Q9NX47 |  |
| MARCHF6 | Membrane associated ring-CH-type finger 6 | | | O60337 |  |
| MARCHF8 | Membrane associated ring-CH-type finger 8 | | | Q5T0T0 |  |
| MARCHF9 | Membrane associated ring-CH-type finger 9 | | | Q86YJ5 |  |
| MARCKSL1 | MARCKS like 1 | | | P49006 |  |
| MARK2 | Microtubule affinity regulating kinase 2 | | | Q7KZI7 |  |
| MARK3 | Microtubule affinity regulating kinase 3 | | | P27448 |  |
| MARS2 | Methionyl-tRNA synthetase 2, mitochondrial | | | Q96GW9 |  |
| MAST3 | Microtubule associated serine/threonine kinase 3 | | | O60307 |  |
| MAST4 | Microtubule associated serine/threonine kinase family member 4 | | | O15021 |  |
| MAT2A | Methionine adenosyltransferase 2A | | | P31153 |  |
| MAT2B | Methionine adenosyltransferase 2B | | | Q9NZL9 |  |
| MATK | Megakaryocyte-associated tyrosine kinase | | | P42679 |  |
| MATR3 | Matrin 3 | | | P43243 |  |
| MATR3 | Matrin 3 | | |  |  |
| MAU2 | MAU2 sister chromatid cohesion factor | | | Q9Y6X3 |  |
| MAVS | Mitochondrial antiviral signaling protein | | | Q7Z434 |  |
| MAX | MYC associated factor X | | | P61244 |  |
| MB21D2 | Mab-21 domain containing 2 | | | Q8IYB1 |  |
| MB21D2 | Mab-21 domain containing 2 | | | Q8IYB1 |  |
| MBD1 | Methyl-CpG binding domain protein 1 | | | Q9UIS9 |  |
| MBD4 | Methyl-CpG binding domain 4, DNA glycosylase | | | O95243 |  |
| MBIP | MAP3K12 binding inhibitory protein 1 | | | Q9NS73 |  |
| MBNL1 | Muscleblind like splicing regulator 1 | | | Q9NR56 |  |
| MBNL2 | Muscleblind like splicing regulator 2 | | | Q5VZF2 |  |
| MBNL3 | Muscleblind like splicing regulator 3 | | | Q9NUK0 |  |
| MBOAT7 | Membrane bound O-acyltransferase domain containing 7 | | | Q96N66 |  |
| MBP | Myelin basic protein | | | P02686 |  |
| MBTPS1 | Membrane bound transcription factor peptidase, site 1 | | | Q14703 |  |
| MBTPS2 | Membrane bound transcription factor peptidase, site 2 | | | O43462 |  |
| MCAT | Malonyl-CoA-acyl carrier protein transacylase | | | Q8IVS2 |  |
| MCCC1 | Methylcrotonyl-CoA carboxylase subunit 1 | | | Q96RQ3 |  |
| MCCC2 | Methylcrotonyl-CoA carboxylase subunit 2 | | | Q9HCC0 |  |
| MCEE | Methylmalonyl-CoA epimerase | | | Q96PE7 |  |
| MCFD2 | Multiple coagulation factor deficiency 2, ER cargo receptor complex subunit | | | Q8NI22 |  |
| MCL1 | MCL1 apoptosis regulator, BCL2 family member | | | Q07820 |  |
| MCM2 | Minichromosome maintenance complex component 2 | | | P49736 |  |
| MCM3 | Minichromosome maintenance complex component 3 | | | P25205 |  |
| MCM4 | Minichromosome maintenance complex component 4 | | | P33991 |  |
| MCM5 | Minichromosome maintenance complex component 5 | | | P33992 |  |
| MCM6 | Minichromosome maintenance complex component 6 | | | Q14566 |  |
| MCM7 | Minichromosome maintenance complex component 7 | | | P33993 |  |
| MCM9 | Minichromosome maintenance 9 homologous recombination repair factor | | | Q9NXL9 |  |
| MCOLN1 | Mucolipin TRP cation channel 1 | | | Q9GZU1 |  |
| MCOLN2 | Mucolipin TRP cation channel 2 | | | Q8IZK6 |  |
| MCPH1 | Microcephalin 1 | | | Q8NEM0 |  |
| MCRS1 | Microspherule protein 1 | | | Q96EZ8 |  |
| MCTP2 | Multiple C2 and transmembrane domain containing 2 | | | Q6DN12 |  |
| MCTS1 | MCTS1 re-initiation and release factor | | | Q9ULC4 |  |
| MCU | Mitochondrial calcium uniporter | | | Q8NE86 |  |
| MCUR1 | Mitochondrial calcium uniporter regulator 1 | | | Q96AQ8 |  |
| MDFIC | MyoD family inhibitor domain containing | | | Q9P1T7 |  |
| MDH1 | Malate dehydrogenase 1 | | | P40925 |  |
| MDH2 | Malate dehydrogenase 2 | | | P40926 |  |
| MDM1 | Mdm1 nuclear protein | | | Q8TC05 |  |
| MDM2 | MDM2 proto-oncogene | | | Q00987 |  |
| MDM4 | MDM4 regulator of p53 | | | O15151 |  |
| MDP1 | Magnesium dependent phosphatase 1 | | | Q86V88 |  |
| MEA1 | Male-enhanced antigen 1 | | | Q16626 |  |
| MEAF6 | MYST/Esa1 associated factor 6 | | | Q9HAF1 |  |
| MECP2 | Methyl-CpG binding protein 2 | | | P51608 |  |
| MECR | Mitochondrial trans-2-enoyl-CoA reductase | | | Q9BV79 |  |
| MED1 | Mediator complex subunit 1 | | | Q15648 |  |
| MED10 | Mediator complex subunit 10 | | | Q9BTT4 |  |
| MED11 | Mediator complex subunit 11 | | | Q9P086 |  |
| MED12 | Mediator complex subunit 12 | | | Q93074 |  |
| MED15 | Mediator complex subunit 15 | | | Q96RN5 |  |
| MED16 | Mediator complex subunit 16 | | | Q9Y2X0 |  |
| MED17 | Mediator complex subunit 17 | | | Q9NVC6 |  |
| MED18 | Mediator complex subunit 18 | | | Q9BUE0 |  |
| MED19 | Mediator complex subunit 19 | | | A0JLT2 |  |
| MED20 | Mediator complex subunit 20 | | | Q9H944 |  |
| MED21 | Mediator complex subunit 21 | | | Q13503 |  |
| MED23 | Mediator complex subunit 23 | | | Q9ULK4 |  |
| MED24 | Mediator complex subunit 24 | | | O75448 |  |
| MED25 | Mediator complex subunit 25 | | | Q71SY5 |  |
| MED27 | Mediator complex subunit 27 | | | Q6P2C8 |  |
| MED28 | Mediator complex subunit 28 | | | Q9H204 |  |
| MED29 | Mediator complex subunit 29 | | | Q9NX70 |  |
| MED30 | Mediator complex subunit 30 | | | Q96HR3 |  |
| MED31 | Mediator complex subunit 31 | | | Q9Y3C7 |  |
| MED4 | Mediator complex subunit 4 | | | Q9NPJ6 |  |
| MED6 | Mediator complex subunit 6 | | | O75586 |  |
| MED7 | Mediator complex subunit 7 | | | O43513 |  |
| MED8 | Mediator complex subunit 8 | | | Q96G25 |  |
| MED9 | Mediator complex subunit 9 | | | Q9NWA0 |  |
| MEF2A | Myocyte enhancer factor 2A | | | Q02078 |  |
| MEI1 | Meiotic double-stranded break formation protein 1 | | | Q5TIA1 |  |
| MEMO1 | Mediator of cell motility 1 | | | Q9Y316 |  |
| MEN1 | Menin 1 | | | O00255 |  |
| MEOX1 | Mesenchyme homeobox 1 | | | P50221 |  |
| MEPCE | Methylphosphate capping enzyme | | | Q7L2J0 |  |
| MEST | Mesoderm specific transcript | | | Q5EB52 |  |
| METAP2 | Methionyl aminopeptidase 2 | | | P50579 |  |
| METTL1 | Methyltransferase 1, tRNA methylguanosine | | | Q9UBP6 |  |
| METTL13 | Methyltransferase 13, eEF1A lysine and N-terminal methyltransferase | | | Q8N6R0 |  |
| METTL14 | Methyltransferase 14, N6-adenosine-methyltransferase subunit | | | Q9HCE5 |  |
| METTL15 | Methyltransferase like 15 | | | A6NJ78 |  |
| METTL16 | Methyltransferase 16, N6-methyladenosine | | | Q86W50 |  |
| METTL17 | Methyltransferase like 17 | | | Q9H7H0 |  |
| METTL18 | Methyltransferase like 18 | | | O95568 |  |
| METTL21A | Methyltransferase 21A, HSPA lysine | | | Q8WXB1 |  |
| METTL22 | Methyltransferase 22, Kin17 lysine | | | Q9BUU2 |  |
| METTL25 | Methyltransferase like 25 | | | Q8N6Q8 |  |
| METTL2A | Methyltransferase 2A, methylcytidine | | | Q96IZ6 |  |
| METTL2B | Methyltransferase 2B, methylcytidine | | | Q6P1Q9 |  |
| METTL3 | Methyltransferase 3, N6-adenosine-methyltransferase complex catalytic subunit | | | Q86U44 |  |
| METTL4 | Methyltransferase 4, N6-adenosine | | | Q8N3J2 |  |
| METTL5 | Methyltransferase 5, N6-adenosine | | | Q9NRN9 |  |
| METTL6 | Methyltransferase 6, methylcytidine | | | Q8TCB7 |  |
| METTL7A | Methyltransferase like 7A | | | Q9H8H3 |  |
| METTL8 | Methyltransferase 8, methylcytidine | | |  |  |
| METTL9 | Methyltransferase like 9 | | | Q9H1A3 |  |
| MEX3C | Mex-3 RNA binding family member C | | | Q5U5Q3 |  |
| MFAP1 | Microfibril associated protein 1 | | | P55081 |  |
| MFAP3 | Microfibril associated protein 3 | | | P55082 |  |
| MFF | Mitochondrial fission factor | | | Q9GZY8 |  |
| MFGE8 | Milk fat globule EGF and factor V/VIII domain containing | | | Q08431 |  |
| MFHAS1 | Multifunctional ROCO family signaling regulator 1 | | | Q9Y4C4 |  |
| MFN1 | Mitofusin 1 | | | Q8IWA4 |  |
| MFN2 | Mitofusin 2 | | | O95140 |  |
| MFNG | MFNG O-fucosylpeptide 3-beta-N-acetylglucosaminyltransferase | | | O00587 |  |
| MFSD10 | Major facilitator superfamily domain containing 10 | | | Q14728 |  |
| MFSD11 | Major facilitator superfamily domain containing 11 | | | O43934 |  |
| MFSD12 | Major facilitator superfamily domain containing 12 | | | Q6NUT3 |  |
| MFSD13A | Major facilitator superfamily domain containing 13A | | | Q14CX5 |  |
| MFSD3 | Major facilitator superfamily domain containing 3 | | | Q96ES6 |  |
| MFSD5 | Major facilitator superfamily domain containing 5 | | | Q6N075 |  |
| MFSD8 | Major facilitator superfamily domain containing 8 | | | Q8NHS3 |  |
| MGAT1 | Alpha-1,3-mannosyl-glycoprotein 2-beta-N-acetylglucosaminyltransferase | | | P26572 |  |
| MGAT2 | Alpha-1,6-mannosyl-glycoprotein 2-beta-N-acetylglucosaminyltransferase | | | Q10469 |  |
| MGAT4A | Alpha-1,3-mannosyl-glycoprotein 4-beta-N-acetylglucosaminyltransferase A | | | Q9UM21 |  |
| MGAT5 | Alpha-1,6-mannosylglycoprotein 6-beta-N-acetylglucosaminyltransferase | | | Q09328 |  |
| MGME1 | Mitochondrial genome maintenance exonuclease 1 | | | Q9BQP7 |  |
| MGMT | O-6-methylguanine-DNA methyltransferase | | | P16455 |  |
| MGRN1 | Mahogunin ring finger 1 | | | O60291 |  |
| MGST2 | Microsomal glutathione S-transferase 2 | | | Q99735 |  |
| MGST3 | Microsomal glutathione S-transferase 3 | | | O14880 |  |
| MIA2 | MIA SH3 domain ER export factor 2 | | | Q96PC5 |  |
| MIB2 | MIB E3 ubiquitin protein ligase 2 | | | Q96AX9 |  |
| MICA | MHC class I polypeptide-related sequence A | | |  |  |
| MICU1 | Mitochondrial calcium uptake 1 | | | Q9BPX6 |  |
| MICU2 | Mitochondrial calcium uptake 2 | | | Q8IYU8 |  |
| MID1IP1 | MID1 interacting protein 1 | | | Q9NPA3 |  |
| MID2 | Midline 2 | | | Q9UJV3 |  |
| MIEF1 | Mitochondrial elongation factor 1 | | | Q9NQG6 |  |
| MIEF2 | Mitochondrial elongation factor 2 | | | Q96C03 |  |
| MIEN1 | Migration and invasion enhancer 1 | | | Q9BRT3 |  |
| MIER1 | MIER1 transcriptional regulator | | | Q8N108 |  |
| MIER2 | MIER family member 2 | | | Q8N344 |  |
| MIER3 | MIER family member 3 | | | Q7Z3K6 |  |
| MIF | Macrophage migration inhibitory factor | | | P14174 |  |
| MIF4GD | MIF4G domain containing | | | A9UHW6 |  |
| MIIP | Migration and invasion inhibitory protein | | | Q5JXC2 |  |
| MINK1 | Misshapen like kinase 1 | | | Q8N4C8 |  |
| MINPP1 | Multiple inositol-polyphosphate phosphatase 1 | | | Q9UNW1 |  |
| MIOS | Meiosis regulator for oocyte development | | | Q9NXC5 |  |
| MIPEP | Mitochondrial intermediate peptidase | | | Q99797 |  |
| MIS12 | MIS12 kinetochore complex component | | | Q9H081 |  |
| MIS18A | MIS18 kinetochore protein A | | | Q9NYP9 |  |
| MIS18BP1 | MIS18 binding protein 1 | | | Q6P0N0 |  |
| MITD1 | Microtubule interacting and trafficking domain containing 1 | | | Q8WV92 |  |
| MIXL1 | Mix paired-like homeobox | | | Q9H2W2 |  |
| MKKS | MKKS centrosomal shuttling protein | | | Q9NPJ1 |  |
| MKKS | MKKS centrosomal shuttling protein | | |  |  |
| MKKS | MKKS centrosomal shuttling protein | | |  |  |
| MKNK1 | MAPK interacting serine/threonine kinase 1 | | | Q9BUB5 |  |
| MKNK2 | MAPK interacting serine/threonine kinase 2 | | | Q9HBH9 |  |
| MKRN1 | Makorin ring finger protein 1 | | | Q9UHC7 |  |
| MKRN2 | Makorin ring finger protein 2 | | | Q9H000 |  |
| MLEC | Malectin | | | Q14165 |  |
| MLF1 | Myeloid leukemia factor 1 | | | P58340 |  |
| MLF2 | Myeloid leukemia factor 2 | | | Q15773 |  |
| MLH1 | MutL homolog 1 | | | P40692 |  |
| MLH3 | MutL homolog 3 | | | Q9UHC1 |  |
| MLKL | Mixed lineage kinase domain like pseudokinase | | | Q8NB16 |  |
| MLLT10 | MLLT10 histone lysine methyltransferase DOT1L cofactor | | | P55197 |  |
| MLLT11 | MLLT11 transcription factor 7 cofactor | | | Q13015 |  |
| MLLT3 | MLLT3 super elongation complex subunit | | | P42568 |  |
| MLST8 | MTOR associated protein, LST8 homolog | | | Q9BVC4 |  |
| MLX | MAX dimerization protein MLX | | | Q9UH92 |  |
| MLXIP | MLX interacting protein | | | Q9HAP2 |  |
| MMAA | Metabolism of cobalamin associated A | | | Q8IVH4 |  |
| MMAB | Metabolism of cobalamin associated B | | | Q96EY8 |  |
| MMACHC | Metabolism of cobalamin associated C | | | Q9Y4U1 |  |
| MMADHC | Metabolism of cobalamin associated D | | | Q9H3L0 |  |
| MMD | Monocyte to macrophage differentiation associated | | | Q15546 |  |
| MMEL1 | Membrane metalloendopeptidase like 1 | | | Q495T6 |  |
| MMGT1 | Membrane magnesium transporter 1 | | | Q8N4V1 |  |
| MMP28 | Matrix metallopeptidase 28 | | | Q9H239 |  |
| MMS19 | MMS19 homolog, cytosolic iron-sulfur assembly component | | | Q96T76 |  |
| MNAT1 | MNAT1 component of CDK activating kinase | | | P51948 |  |
| MNDA | Myeloid cell nuclear differentiation antigen | | | P41218 |  |
| MOAP1 | Modulator of apoptosis 1 | | | Q96BY2 |  |
| MOB1A | MOB kinase activator 1A | | | Q9H8S9 |  |
| MOB2 | MOB kinase activator 2 | | | Q70IA6 |  |
| MOB3A | MOB kinase activator 3A | | | Q96BX8 |  |
| MOB3C | MOB kinase activator 3C | | | Q70IA8 |  |
| MOB4 | MOB family member 4, phocein | | | Q9Y3A3 |  |
| MOCS2 | Molybdenum cofactor synthesis 2 | | | O96007, O96033 |  |
| MOCS3 | Molybdenum cofactor synthesis 3 | | | O95396 |  |
| MOGS | Mannosyl-oligosaccharide glucosidase | | | Q13724 |  |
| MON1A | MON1 homolog A, secretory trafficking associated | | | Q86VX9 |  |
| MON1B | MON1 homolog B, secretory trafficking associated | | | Q7L1V2 |  |
| MORC2 | MORC family CW-type zinc finger 2 | | | Q9Y6X9 |  |
| MORF4L1 | Mortality factor 4 like 1 | | | Q9UBU8 |  |
| MORF4L2 | Mortality factor 4 like 2 | | | Q15014 |  |
| MORN2 | MORN repeat containing 2 | | | Q502X0 |  |
| MORN4 | MORN repeat containing 4 | | | Q8NDC4 |  |
| MOSPD1 | Motile sperm domain containing 1 | | | Q9UJG1 |  |
| MOSPD2 | Motile sperm domain containing 2 | | | Q8NHP6 |  |
| MOSPD3 | Motile sperm domain containing 3 | | | O75425 |  |
| MOV10 | Mov10 RISC complex RNA helicase | | | Q9HCE1 |  |
| MPC1 | Mitochondrial pyruvate carrier 1 | | | Q9Y5U8 |  |
| MPC2 | Mitochondrial pyruvate carrier 2 | | | O95563 |  |
| MPDU1 | Mannose-P-dolichol utilization defect 1 | | | O75352 |  |
| MPG | N-methylpurine DNA glycosylase | | | P29372 |  |
| MPHOSPH6 | M-phase phosphoprotein 6 | | | Q99547 |  |
| MPHOSPH9 | M-phase phosphoprotein 9 | | | Q99550 |  |
| MPI | Mannose phosphate isomerase | | | P34949 |  |
| MPLKIP | M-phase specific PLK1 interacting protein | | | Q8TAP9 |  |
| MPND | MPN domain containing | | | Q8N594 |  |
| MPP1 | MAGUK p55 scaffold protein 1 | | | Q00013 |  |
| MPP7 | MAGUK p55 scaffold protein 7 | | | Q5T2T1 |  |
| MPPE1 | Metallophosphoesterase 1 | | | Q53F39 |  |
| MPRIP | Myosin phosphatase Rho interacting protein | | | Q6WCQ1 |  |
| MPST | Mercaptopyruvate sulfurtransferase | | | P25325 |  |
| MPV17 | Mitochondrial inner membrane protein MPV17 | | | P39210 |  |
| MPV17L2 | MPV17 mitochondrial inner membrane protein like 2 | | | Q567V2 |  |
| MPZL1 | Myelin protein zero like 1 | | | O95297 |  |
| MPZL3 | Myelin protein zero like 3 | | | Q6UWV2 |  |
| MR1 | Major histocompatibility complex, class I-related | | | Q95460 |  |
| MRFAP1 | Morf4 family associated protein 1 | | | Q9Y605 |  |
| MRFAP1L1 | Morf4 family associated protein 1 like 1 | | | Q96HT8 |  |
| MRI1 | Methylthioribose-1-phosphate isomerase 1 | | | Q9BV20 |  |
| MRM1 | Mitochondrial rRNA methyltransferase 1 | | | Q6IN84 |  |
| MRPL1 | Mitochondrial ribosomal protein L1 | | | Q9BYD6 |  |
| MRPL10 | Mitochondrial ribosomal protein L10 | | | Q7Z7H8 |  |
| MRPL11 | Mitochondrial ribosomal protein L11 | | | Q9Y3B7 |  |
| MRPL12 | Mitochondrial ribosomal protein L12 | | | P52815 |  |
| MRPL13 | Mitochondrial ribosomal protein L13 | | | Q9BYD1 |  |
| MRPL14 | Mitochondrial ribosomal protein L14 | | | Q6P1L8 |  |
| MRPL15 | Mitochondrial ribosomal protein L15 | | | Q9P015 |  |
| MRPL16 | Mitochondrial ribosomal protein L16 | | | Q9NX20 |  |
| MRPL17 | Mitochondrial ribosomal protein L17 | | | Q9NRX2 |  |
| MRPL18 | Mitochondrial ribosomal protein L18 | | | Q9H0U6 |  |
| MRPL19 | Mitochondrial ribosomal protein L19 | | | P49406 |  |
| MRPL2 | Mitochondrial ribosomal protein L2 | | | Q5T653 |  |
| MRPL20 | Mitochondrial ribosomal protein L20 | | | Q9BYC9 |  |
| MRPL21 | Mitochondrial ribosomal protein L21 | | | Q7Z2W9 |  |
| MRPL22 | Mitochondrial ribosomal protein L22 | | | Q9NWU5 |  |
| MRPL23 | Mitochondrial ribosomal protein L23 | | | Q16540 |  |
| MRPL24 | Mitochondrial ribosomal protein L24 | | | Q96A35 |  |
| MRPL27 | Mitochondrial ribosomal protein L27 | | | Q9P0M9 |  |
| MRPL28 | Mitochondrial ribosomal protein L28 | | | Q13084 |  |
| MRPL3 | Mitochondrial ribosomal protein L3 | | | P09001 |  |
| MRPL30 | Mitochondrial ribosomal protein L30 | | | Q8TCC3 |  |
| MRPL32 | Mitochondrial ribosomal protein L32 | | | Q9BYC8 |  |
| MRPL33 | Mitochondrial ribosomal protein L33 | | | O75394 |  |
| MRPL34 | Mitochondrial ribosomal protein L34 | | | Q9BQ48 |  |
| MRPL35 | Mitochondrial ribosomal protein L35 | | | Q9NZE8 |  |
| MRPL36 | Mitochondrial ribosomal protein L36 | | | Q9P0J6 |  |
| MRPL37 | Mitochondrial ribosomal protein L37 | | | Q9BZE1 |  |
| MRPL38 | Mitochondrial ribosomal protein L38 | | | Q96DV4 |  |
| MRPL39 | Mitochondrial ribosomal protein L39 | | | Q9NYK5 |  |
| MRPL4 | Mitochondrial ribosomal protein L4 | | | Q9BYD3 |  |
| MRPL40 | Mitochondrial ribosomal protein L40 | | | Q9NQ50 |  |
| MRPL41 | Mitochondrial ribosomal protein L41 | | | Q8IXM3 |  |
| MRPL42 | Mitochondrial ribosomal protein L42 | | | Q9Y6G3 |  |
| MRPL43 | Mitochondrial ribosomal protein L43 | | | Q8N983 |  |
| MRPL44 | Mitochondrial ribosomal protein L44 | | | Q9H9J2 |  |
| MRPL46 | Mitochondrial ribosomal protein L46 | | | Q9H2W6 |  |
| MRPL47 | Mitochondrial ribosomal protein L47 | | | Q9HD33 |  |
| MRPL48 | Mitochondrial ribosomal protein L48 | | | Q96GC5 |  |
| MRPL49 | Mitochondrial ribosomal protein L49 | | | Q13405 |  |
| MRPL50 | Mitochondrial ribosomal protein L50 | | | Q8N5N7 |  |
| MRPL51 | Mitochondrial ribosomal protein L51 | | | Q4U2R6 |  |
| MRPL52 | Mitochondrial ribosomal protein L52 | | | Q86TS9 |  |
| MRPL53 | Mitochondrial ribosomal protein L53 | | | Q96EL3 |  |
| MRPL54 | Mitochondrial ribosomal protein L54 | | | Q6P161 |  |
| MRPL55 | Mitochondrial ribosomal protein L55 | | | Q7Z7F7 |  |
| MRPL57 | Mitochondrial ribosomal protein L57 | | | Q9BQC6 |  |
| MRPL9 | Mitochondrial ribosomal protein L9 | | | Q9BYD2 |  |
| MRPS10 | Mitochondrial ribosomal protein S10 | | | P82664 |  |
| MRPS11 | Mitochondrial ribosomal protein S11 | | | P82912 |  |
| MRPS12 | Mitochondrial ribosomal protein S12 | | | O15235 |  |
| MRPS14 | Mitochondrial ribosomal protein S14 | | | O60783 |  |
| MRPS15 | Mitochondrial ribosomal protein S15 | | | P82914 |  |
| MRPS16 | Mitochondrial ribosomal protein S16 | | | Q9Y3D3 |  |
| MRPS17 | Mitochondrial ribosomal protein S17 | | | Q9Y2R5 |  |
| MRPS18A | Mitochondrial ribosomal protein S18A | | | Q9NVS2 |  |
| MRPS18B | Mitochondrial ribosomal protein S18B | | | Q9Y676 |  |
| MRPS18C | Mitochondrial ribosomal protein S18C | | | Q9Y3D5 |  |
| MRPS2 | Mitochondrial ribosomal protein S2 | | | Q9Y399 |  |
| MRPS21 | Mitochondrial ribosomal protein S21 | | | P82921 |  |
| MRPS22 | Mitochondrial ribosomal protein S22 | | | P82650 |  |
| MRPS23 | Mitochondrial ribosomal protein S23 | | | Q9Y3D9 |  |
| MRPS24 | Mitochondrial ribosomal protein S24 | | | Q96EL2 |  |
| MRPS25 | Mitochondrial ribosomal protein S25 | | | P82663 |  |
| MRPS26 | Mitochondrial ribosomal protein S26 | | | Q9BYN8 |  |
| MRPS27 | Mitochondrial ribosomal protein S27 | | | Q92552 |  |
| MRPS28 | Mitochondrial ribosomal protein S28 | | | Q9Y2Q9 |  |
| MRPS30 | Mitochondrial ribosomal protein S30 | | | Q9NP92 |  |
| MRPS31 | Mitochondrial ribosomal protein S31 | | | Q92665 |  |
| MRPS33 | Mitochondrial ribosomal protein S33 | | | Q9Y291 |  |
| MRPS34 | Mitochondrial ribosomal protein S34 | | | P82930 |  |
| MRPS35 | Mitochondrial ribosomal protein S35 | | | P82673 |  |
| MRPS36 | Mitochondrial ribosomal protein S36 | | | P82909 |  |
| MRPS5 | Mitochondrial ribosomal protein S5 | | | P82675 |  |
| MRPS6 | Mitochondrial ribosomal protein S6 | | | P82932 |  |
| MRPS7 | Mitochondrial ribosomal protein S7 | | | Q9Y2R9 |  |
| MRPS9 | Mitochondrial ribosomal protein S9 | | | P82933 |  |
| MRRF | Mitochondrial ribosome recycling factor | | | Q96E11 |  |
| MRS2 | Magnesium transporter MRS2 | | | Q9HD23 |  |
| MRTO4 | MRT4 homolog, ribosome maturation factor | | | Q9UKD2 |  |
| MS4A1 | Membrane spanning 4-domains A1 | | | P11836 |  |
| MS4A6A | Membrane spanning 4-domains A6A | | | Q9H2W1 |  |
| MSANTD3 | Myb/SANT DNA binding domain containing 3 | | | Q96H12 |  |
| MSANTD4 | Myb/SANT DNA binding domain containing 4 with coiled-coils | | | Q8NCY6 |  |
| MSC | Musculin | | | O60682 |  |
| MSH2 | MutS homolog 2 | | | P43246 |  |
| MSH6 | MutS homolog 6 | | | P52701 |  |
| MSL2 | MSL complex subunit 2 | | | Q9HCI7 |  |
| MSL3 | MSL complex subunit 3 | | | Q8N5Y2 |  |
| MSMO1 | Methylsterol monooxygenase 1 | | | Q15800 |  |
| MSN | Moesin | | | P26038 |  |
| MSR1 | Macrophage scavenger receptor 1 | | | P21757 |  |
| MSRB1 | Methionine sulfoxide reductase B1 | | | Q9NZV6 |  |
| MSTO1 | Misato mitochondrial distribution and morphology regulator 1 | | | Q9BUK6 |  |
| MT1E | Metallothionein 1E | | | P04732 |  |
| MT1F | Metallothionein 1F | | | P04733 |  |
| MT1X | Metallothionein 1X | | | P80297 |  |
| MT2A | Metallothionein 2A | | | P02795 |  |
| MT3 | Metallothionein 3 | | | P25713 |  |
| MTA2 | Metastasis associated 1 family member 2 | | | O94776 |  |
| MTA3 | Metastasis associated 1 family member 3 | | | Q9BTC8 |  |
| MTAP | Methylthioadenosine phosphorylase | | | Q13126 |  |
| MTCH2 | Mitochondrial carrier 2 | | | Q9Y6C9 |  |
| MTDH | Metadherin | | | Q86UE4 |  |
| MTERF1 | Mitochondrial transcription termination factor 1 | | | Q99551 |  |
| MTERF2 | Mitochondrial transcription termination factor 2 | | | Q49AM1 |  |
| MTERF3 | Mitochondrial transcription termination factor 3 | | | Q96E29 |  |
| MTERF4 | Mitochondrial transcription termination factor 4 | | | Q7Z6M4 |  |
| MTF1 | Metal regulatory transcription factor 1 | | | Q14872 |  |
| MTF2 | Metal response element binding transcription factor 2 | | | Q9Y483 |  |
| MTFMT | Mitochondrial methionyl-tRNA formyltransferase | | | Q96DP5 |  |
| MTFP1 | Mitochondrial fission process 1 | | | Q9UDX5 |  |
| MTFR1L | Mitochondrial fission regulator 1 like | | | Q9H019 |  |
| MTG1 | Mitochondrial ribosome associated GTPase 1 | | | Q9BT17 |  |
| MTG2 | Mitochondrial ribosome associated GTPase 2 | | | Q9H4K7 |  |
| MTHFD1 | Methylenetetrahydrofolate dehydrogenase, cyclohydrolase and formyltetrahydrofolate synthetase 1 | | | P11586 |  |
| MTHFD2 | Methylenetetrahydrofolate dehydrogenase (NADP+ dependent) 2, methenyltetrahydrofolate cyclohydrolase | | | P13995 |  |
| MTHFR | Methylenetetrahydrofolate reductase | | | P42898 |  |
| MTHFS | Methenyltetrahydrofolate synthetase | | | P49914 |  |
| MTHFSD | Methenyltetrahydrofolate synthetase domain containing | | | Q2M296 |  |
| MTIF3 | Mitochondrial translational initiation factor 3 | | | Q9H2K0 |  |
| MTM1 | Myotubularin 1 | | | Q13496 |  |
| MTMR1 | Myotubularin related protein 1 | | | Q13613 |  |
| MTMR10 | Myotubularin related protein 10 | | | Q9NXD2 |  |
| MTMR12 | Myotubularin related protein 12 | | | Q9C0I1 |  |
| MTMR14 | Myotubularin related protein 14 | | | Q8NCE2 |  |
| MTMR2 | Myotubularin related protein 2 | | | Q13614 |  |
| MTMR3 | Myotubularin related protein 3 | | | Q13615 |  |
| MTMR4 | Myotubularin related protein 4 | | | Q9NYA4 |  |
| MTMR6 | Myotubularin related protein 6 | | | Q9Y217 |  |
| MTMR8 | Myotubularin related protein 8 | | | Q96EF0 |  |
| MTPAP | Mitochondrial poly(A) polymerase | | | Q9NVV4 |  |
| MTPN | Myotrophin | | | P58546 |  |
| MTRF1 | Mitochondrial translation release factor 1 | | | O75570 |  |
| MTRF1L | Mitochondrial translation release factor 1 like | | | Q9UGC7 |  |
| MTUS1 | Microtubule associated scaffold protein 1 | | | Q9ULD2 |  |
| MTX2 | Metaxin 2 | | | O75431 |  |
| MUL1 | Mitochondrial E3 ubiquitin protein ligase 1 | | | Q969V5 |  |
| MUS81 | MUS81 structure-specific endonuclease subunit | | | Q96NY9 |  |
| MUTYH | MutY DNA glycosylase | | | Q9UIF7 |  |
| MVB12A | Multivesicular body subunit 12A | | | Q96EY5 |  |
| MVD | Mevalonate diphosphate decarboxylase | | | P53602 |  |
| MVK | Mevalonate kinase | | | Q03426 |  |
| MVP | Major vault protein | | | Q14764 |  |
| MX1 | MX dynamin like GTPase 1 | | | P20591 |  |
| MX2 | MX dynamin like GTPase 2 | | | P20592 |  |
| MXD1 | MAX dimerization protein 1 | | | Q05195 |  |
| MXD4 | MAX dimerization protein 4 | | | Q14582 |  |
| MXI1 | MAX interactor 1, dimerization protein | | | P50539 |  |
| MYADM | Myeloid associated differentiation marker | | | Q96S97 |  |
| MYBBP1A | MYB binding protein 1a | | | Q9BQG0 |  |
| MYC | MYC proto-oncogene, bHLH transcription factor | | | P01106 |  |
| MYCBP | MYC binding protein | | | Q99417 |  |
| MYD88 | MYD88 innate immune signal transduction adaptor | | | Q99836 |  |
| MYDGF | Myeloid derived growth factor | | | Q969H8 |  |
| MYH9 | Myosin heavy chain 9 | | | P35579 |  |
| MYL12A | Myosin light chain 12A | | | P19105 |  |
| MYL12B | Myosin light chain 12B | | | O14950 |  |
| MYL5 | Myosin light chain 5 | | | Q02045 |  |
| MYL6 | Myosin light chain 6 | | | P60660 |  |
| MYL6B | Myosin light chain 6B | | | P14649 |  |
| MYLIP | Myosin regulatory light chain interacting protein | | | Q8WY64 |  |
| MYNN | Myoneurin | | | Q9NPC7 |  |
| MYO1C | Myosin IC | | | O00159 |  |
| MYO1F | Myosin IF | | | O00160 |  |
| MYO6 | Myosin VI | | | Q9UM54 |  |
| MYZAP | Myocardial zonula adherens protein | | | P0CAP1 |  |
| MZF1 | Myeloid zinc finger 1 | | | P28698 |  |
| MZT1 | Mitotic spindle organizing protein 1 | | | Q08AG7 |  |
| MZT2B | Mitotic spindle organizing protein 2B | | | Q6NZ67 |  |
| N4BP1 | NEDD4 binding protein 1 | | | O75113 |  |
| N4BP2L1 | NEDD4 binding protein 2 like 1 | | | Q5TBK1 |  |
| N4BP2L2 | NEDD4 binding protein 2 like 2 | | | Q92802 |  |
| N6AMT1 | N-6 adenine-specific DNA methyltransferase 1 | | | Q9Y5N5 |  |
| NAA10 | N-alpha-acetyltransferase 10, NatA catalytic subunit | | | P41227 |  |
| NAA15 | N-alpha-acetyltransferase 15, NatA auxiliary subunit | | | Q9BXJ9 |  |
| NAA20 | N-alpha-acetyltransferase 20, NatB catalytic subunit | | | P61599 |  |
| NAA25 | N-alpha-acetyltransferase 25, NatB auxiliary subunit | | | Q14CX7 |  |
| NAA30 | N-alpha-acetyltransferase 30, NatC catalytic subunit | | | Q147X3 |  |
| NAA38 | N-alpha-acetyltransferase 38, NatC auxiliary subunit | | | Q9BRA0 |  |
| NAA40 | N-alpha-acetyltransferase 40, NatD catalytic subunit | | | Q86UY6 |  |
| NAA50 | N-alpha-acetyltransferase 50, NatE catalytic subunit | | | Q9GZZ1 |  |
| NAA60 | N-alpha-acetyltransferase 60, NatF catalytic subunit | | | Q9H7X0 |  |
| NAAA | N-acylethanolamine acid amidase | | | Q02083 |  |
| NAALADL1 | N-acetylated alpha-linked acidic dipeptidase like 1 | | | Q9UQQ1 |  |
| NAB1 | NGFI-A binding protein 1 | | | Q13506 |  |
| NAB2 | NGFI-A binding protein 2 | | | Q15742 |  |
| NABP1 | Nucleic acid binding protein 1 | | | Q96AH0 |  |
| NABP2 | Nucleic acid binding protein 2 | | | Q9BQ15 |  |
| NACA | Nascent polypeptide associated complex subunit alpha | | | E9PAV3, Q13765 |  |
| NADK | NAD kinase | | | O95544 |  |
| NADK2 | NAD kinase 2, mitochondrial | | | Q4G0N4 |  |
| NADSYN1 | NAD synthetase 1 | | | Q6IA69 |  |
| NAE1 | NEDD8 activating enzyme E1 subunit 1 | | | Q13564 |  |
| NAF1 | Nuclear assembly factor 1 ribonucleoprotein | | | Q96HR8 |  |
| NAGA | Alpha-N-acetylgalactosaminidase | | | P17050 |  |
| NAGK | N-acetylglucosamine kinase | | | Q9UJ70 |  |
| NAGLU | N-acetyl-alpha-glucosaminidase | | | P54802 |  |
| NAIF1 | Nuclear apoptosis inducing factor 1 | | | Q69YI7 |  |
| NAMPT | Nicotinamide phosphoribosyltransferase | | | P43490 |  |
| NANS | N-acetylneuraminate synthase | | | Q9NR45 |  |
| NAP1L1 | Nucleosome assembly protein 1 like 1 | | | P55209 |  |
| NAP1L2 | Nucleosome assembly protein 1 like 2 | | | Q9ULW6 |  |
| NAP1L3 | Nucleosome assembly protein 1 like 3 | | | Q99457 |  |
| NAP1L3 | Nucleosome assembly protein 1 like 3 | | | Q99457 |  |
| NAP1L4 | Nucleosome assembly protein 1 like 4 | | | Q99733 |  |
| NAP1L5 | Nucleosome assembly protein 1 like 5 | | | Q96NT1 |  |
| NAPA | NSF attachment protein alpha | | | P54920 |  |
| NAPEPLD | N-acyl phosphatidylethanolamine phospholipase D | | | Q6IQ20 |  |
| NAPG | NSF attachment protein gamma | | | Q99747 |  |
| NAPRT | Nicotinate phosphoribosyltransferase | | | Q6XQN6 |  |
| NARF | Nuclear prelamin A recognition factor | | | Q9UHQ1 |  |
| NARS2 | Asparaginyl-tRNA synthetase 2, mitochondrial | | | Q96I59 |  |
| NASP | Nuclear autoantigenic sperm protein | | | P49321 |  |
| NAT1 | N-acetyltransferase 1 | | | P18440 |  |
| NAT9 | N-acetyltransferase 9 (putative) | | | Q9BTE0 |  |
| NBPF15 | NBPF member 15 | | | Q8N660 |  |
| NBPF3 | NBPF member 3 | | | Q9H094 |  |
| NBPF9 | NBPF member 9 | | | P0DPF3 |  |
| NBR1 | NBR1 autophagy cargo receptor | | | Q14596 |  |
| NCALD | Neurocalcin delta | | | P61601 |  |
| NCAPG2 | Non-SMC condensin II complex subunit G2 | | | Q86XI2 |  |
| NCAPH | Non-SMC condensin I complex subunit H | | | Q15003 |  |
| NCAPH2 | Non-SMC condensin II complex subunit H2 | | | Q6IBW4 |  |
| NCBP1 | Nuclear cap binding protein subunit 1 | | | Q09161 |  |
| NCBP2 | Nuclear cap binding protein subunit 2 | | | P52298 |  |
| NCBP3 | Nuclear cap binding subunit 3 | | | Q53F19 |  |
| NCDN | Neurochondrin | | | Q9UBB6 |  |
| NCEH1 | Neutral cholesterol ester hydrolase 1 | | | Q6PIU2 |  |
| NCF1 | Neutrophil cytosolic factor 1 | | | P14598 |  |
| NCF2 | Neutrophil cytosolic factor 2 | | | P19878 |  |
| NCF4 | Neutrophil cytosolic factor 4 | | | Q15080 |  |
| NCK1 | NCK adaptor protein 1 | | | P16333 |  |
| NCK2 | NCK adaptor protein 2 | | | O43639 |  |
| NCKIPSD | NCK interacting protein with SH3 domain | | | Q9NZQ3 |  |
| NCL | Nucleolin | | | P19338 |  |
| NCLN | Nicalin | | | Q969V3 |  |
| NCOA1 | Nuclear receptor coactivator 1 | | | Q15788 |  |
| NCOA3 | Nuclear receptor coactivator 3 | | | Q9Y6Q9 |  |
| NCOA4 | Nuclear receptor coactivator 4 | | | Q13772 |  |
| NCOA5 | Nuclear receptor coactivator 5 | | | Q9HCD5 |  |
| NCOR1 | Nuclear receptor corepressor 1 | | | O75376 |  |
| NCR3 | Natural cytotoxicity triggering receptor 3 | | | O14931 |  |
| NCSTN | Nicastrin | | | Q92542 |  |
| NDC1 | NDC1 transmembrane nucleoporin | | | Q9BTX1 |  |
| NDC80 | NDC80 kinetochore complex component | | | O14777 |  |
| NDE1 | NudE neurodevelopment protein 1 | | | Q9NXR1 |  |
| NDEL1 | NudE neurodevelopment protein 1 like 1 | | | Q9GZM8 |  |
| NDFIP1 | Nedd4 family interacting protein 1 | | | Q9BT67 |  |
| NDFIP2 | Nedd4 family interacting protein 2 | | | Q9NV92 |  |
| NDOR1 | NADPH dependent diflavin oxidoreductase 1 | | | Q9UHB4 |  |
| NDRG1 | N-myc downstream regulated 1 | | | Q92597 |  |
| NDRG2 | NDRG family member 2 | | | Q9UN36 |  |
| NDRG3 | NDRG family member 3 | | | Q9UGV2 |  |
| NDST2 | N-deacetylase and N-sulfotransferase 2 | | | P52849 |  |
| NDUFA1 | NADH:ubiquinone oxidoreductase subunit A1 | | | O15239 |  |
| NDUFA10 | NADH:ubiquinone oxidoreductase subunit A10 | | | O95299 |  |
| NDUFA11 | NADH:ubiquinone oxidoreductase subunit A11 | | | Q86Y39 |  |
| NDUFA12 | NADH:ubiquinone oxidoreductase subunit A12 | | | Q9UI09 |  |
| NDUFA13 | NADH:ubiquinone oxidoreductase subunit A13 | | | Q9P0J0 |  |
| NDUFA2 | NADH:ubiquinone oxidoreductase subunit A2 | | | O43678 |  |
| NDUFA3 | NADH:ubiquinone oxidoreductase subunit A3 | | | O95167 |  |
| NDUFA4 | NDUFA4 mitochondrial complex associated | | | O00483 |  |
| NDUFA5 | NADH:ubiquinone oxidoreductase subunit A5 | | | Q16718 |  |
| NDUFA6 | NADH:ubiquinone oxidoreductase subunit A6 | | | P56556 |  |
| NDUFA7 | NADH:ubiquinone oxidoreductase subunit A7 | | | O95182 |  |
| NDUFA8 | NADH:ubiquinone oxidoreductase subunit A8 | | | P51970 |  |
| NDUFA9 | NADH:ubiquinone oxidoreductase subunit A9 | | | Q16795 |  |
| NDUFAB1 | NADH:ubiquinone oxidoreductase subunit AB1 | | | O14561 |  |
| NDUFAF1 | NADH:ubiquinone oxidoreductase complex assembly factor 1 | | | Q9Y375 |  |
| NDUFAF2 | NADH:ubiquinone oxidoreductase complex assembly factor 2 | | | Q8N183 |  |
| NDUFAF4 | NADH:ubiquinone oxidoreductase complex assembly factor 4 | | | Q9P032 |  |
| NDUFAF5 | NADH:ubiquinone oxidoreductase complex assembly factor 5 | | | Q5TEU4 |  |
| NDUFAF6 | NADH:ubiquinone oxidoreductase complex assembly factor 6 | | | Q330K2 |  |
| NDUFAF7 | NADH:ubiquinone oxidoreductase complex assembly factor 7 | | | Q7L592 |  |
| NDUFB1 | NADH:ubiquinone oxidoreductase subunit B1 | | | O75438 |  |
| NDUFB10 | NADH:ubiquinone oxidoreductase subunit B10 | | | O96000 |  |
| NDUFB11 | NADH:ubiquinone oxidoreductase subunit B11 | | | Q9NX14 |  |
| NDUFB2 | NADH:ubiquinone oxidoreductase subunit B2 | | | O95178 |  |
| NDUFB3 | NADH:ubiquinone oxidoreductase subunit B3 | | | O43676 |  |
| NDUFB4 | NADH:ubiquinone oxidoreductase subunit B4 | | | O95168 |  |
| NDUFB5 | NADH:ubiquinone oxidoreductase subunit B5 | | | O43674 |  |
| NDUFB6 | NADH:ubiquinone oxidoreductase subunit B6 | | | O95139 |  |
| NDUFB7 | NADH:ubiquinone oxidoreductase subunit B7 | | | P17568 |  |
| NDUFB8 | NADH:ubiquinone oxidoreductase subunit B8 | | | O95169 |  |
| NDUFC2 | NADH:ubiquinone oxidoreductase subunit C2 | | | O95298 |  |
| NDUFS1 | NADH:ubiquinone oxidoreductase core subunit S1 | | | P28331 |  |
| NDUFS2 | NADH:ubiquinone oxidoreductase core subunit S2 | | | O75306 |  |
| NDUFS3 | NADH:ubiquinone oxidoreductase core subunit S3 | | | O75489 |  |
| NDUFS4 | NADH:ubiquinone oxidoreductase subunit S4 | | | O43181 |  |
| NDUFS5 | NADH:ubiquinone oxidoreductase subunit S5 | | | O43920 |  |
| NDUFS6 | NADH:ubiquinone oxidoreductase subunit S6 | | | O75380 |  |
| NDUFS7 | NADH:ubiquinone oxidoreductase core subunit S7 | | | O75251 |  |
| NDUFS8 | NADH:ubiquinone oxidoreductase core subunit S8 | | | O00217 |  |
| NDUFV1 | NADH:ubiquinone oxidoreductase core subunit V1 | | | P49821 |  |
| NDUFV2 | NADH:ubiquinone oxidoreductase core subunit V2 | | | P19404 |  |
| NDUFV3 | NADH:ubiquinone oxidoreductase subunit V3 | | | P56181 |  |
| NECAP1 | NECAP endocytosis associated 1 | | | Q8NC96 |  |
| NECAP2 | NECAP endocytosis associated 2 | | | Q9NVZ3 |  |
| NEDD1 | NEDD1 gamma-tubulin ring complex targeting factor | | | Q8NHV4 |  |
| NEDD8 | NEDD8 ubiquitin like modifier | | | Q15843 |  |
| NEDD9 | Neural precursor cell expressed, developmentally down-regulated 9 | | | Q14511 |  |
| NEFL | Neurofilament light chain | | | P07196 |  |
| NEFL | Neurofilament light chain | | | P07196 |  |
| NEIL1 | Nei like DNA glycosylase 1 | | | Q96FI4 |  |
| NEIL2 | Nei like DNA glycosylase 2 | | | Q969S2 |  |
| NEK1 | NIMA related kinase 1 | | | Q96PY6 |  |
| NEK3 | NIMA related kinase 3 | | | P51956 |  |
| NEK6 | NIMA related kinase 6 | | | Q9HC98 |  |
| NEK7 | NIMA related kinase 7 | | | Q8TDX7 |  |
| NEK9 | NIMA related kinase 9 | | | Q8TD19 |  |
| NELFA | Negative elongation factor complex member A | | | Q9H3P2 |  |
| NELFB | Negative elongation factor complex member B | | | Q8WX92 |  |
| NELFCD | Negative elongation factor complex member C/D | | | Q8IXH7 |  |
| NELFE | Negative elongation factor complex member E | | | P18615 |  |
| NELL2 | Neural EGFL like 2 | | | Q99435 |  |
| NELL2 | Neural EGFL like 2 | | | Q99435 |  |
| NEMF | Nuclear export mediator factor | | | O60524 |  |
| NEMP1 | Nuclear envelope integral membrane protein 1 | | | O14524 |  |
| NENF | Neudesin neurotrophic factor | | | Q9UMX5 |  |
| NEPRO | Nucleolus and neural progenitor protein | | | Q6NW34 |  |
| NET1 | Neuroepithelial cell transforming 1 | | | Q7Z628 |  |
| NET1 | Neuroepithelial cell transforming 1 | | | Q7Z628 |  |
| NF2 | NF2, moesin-ezrin-radixin like (MERLIN) tumor suppressor | | | P35240 |  |
| NFATC1 | Nuclear factor of activated T cells 1 | | | O95644 |  |
| NFATC2 | Nuclear factor of activated T cells 2 | | | Q13469 |  |
| NFATC2IP | Nuclear factor of activated T cells 2 interacting protein | | | Q8NCF5 |  |
| NFATC3 | Nuclear factor of activated T cells 3 | | | Q12968 |  |
| NFE2 | Nuclear factor, erythroid 2 | | | Q16621 |  |
| NFE2L1 | NFE2 like bZIP transcription factor 1 | | | Q14494 |  |
| NFE2L2 | NFE2 like bZIP transcription factor 2 | | | Q16236 |  |
| NFIA | Nuclear factor I A | | | Q12857 |  |
| NFIC | Nuclear factor I C | | | P08651 |  |
| NFKB1 | Nuclear factor kappa B subunit 1 | | | P19838 |  |
| NFKBIA | NFKB inhibitor alpha | | | P25963 |  |
| NFKBIB | NFKB inhibitor beta | | | Q15653 |  |
| NFKBID | NFKB inhibitor delta | | | Q8NI38 |  |
| NFKBIE | NFKB inhibitor epsilon | | | O00221 |  |
| NFKBIZ | NFKB inhibitor zeta | | | Q9BYH8 |  |
| NFS1 | NFS1 cysteine desulfurase | | | Q9Y697 |  |
| NFU1 | NFU1 iron-sulfur cluster scaffold | | | Q9UMS0 |  |
| NFX1 | Nuclear transcription factor, X-box binding 1 | | | Q12986 |  |
| NFYA | Nuclear transcription factor Y subunit alpha | | | P23511 |  |
| NFYB | Nuclear transcription factor Y subunit beta | | | P25208 |  |
| NFYC | Nuclear transcription factor Y subunit gamma | | | Q13952 |  |
| NGDN | Neuroguidin | | | Q8NEJ9 |  |
| NGLY1 | N-glycanase 1 | | | Q96IV0 |  |
| NGRN | Neugrin, neurite outgrowth associated | | | Q9NPE2 |  |
| NHEJ1 | Non-homologous end joining factor 1 | | | Q9H9Q4 |  |
| NHLRC1 | NHL repeat containing E3 ubiquitin protein ligase 1 | | | Q6VVB1 |  |
| NHLRC3 | NHL repeat containing 3 | | | Q5JS37 |  |
| NHP2 | NHP2 ribonucleoprotein | | | Q9NX24 |  |
| NHSL2 | NHS like 2 | | | Q5HYW2 |  |
| NICN1 | Nicolin 1, tubulin polyglutamylase complex subunit | | | Q9BSH3 |  |
| NIF3L1 | NGG1 interacting factor 3 like 1 | | | Q9GZT8 |  |
| NIFK | Nucleolar protein interacting with the FHA domain of MKI67 | | | Q9BYG3 |  |
| NIN | Ninein | | | Q8N4C6 |  |
| NINJ1 | Ninjurin 1 | | | Q92982 |  |
| NINJ2 | Ninjurin 2 | | | Q9NZG7 |  |
| NIP7 | Nucleolar pre-rRNA processing protein NIP7 | | | Q9Y221 |  |
| NIPA2 | NIPA magnesium transporter 2 | | | Q8N8Q9 |  |
| NIPAL3 | NIPA like domain containing 3 | | | Q6P499 |  |
| NIPSNAP1 | Nipsnap homolog 1 | | | Q9BPW8 |  |
| NIPSNAP3A | Nipsnap homolog 3A | | | Q9UFN0 |  |
| NISCH | Nischarin | | | Q9Y2I1 |  |
| NIT1 | Nitrilase 1 | | | Q86X76 |  |
| NIT2 | Nitrilase family member 2 | | | Q9NQR4 |  |
| NKAP | NFKB activating protein | | | Q8N5F7 |  |
| NKG7 | Natural killer cell granule protein 7 | | | Q16617 |  |
| NKIRAS1 | NFKB inhibitor interacting Ras like 1 | | | Q9NYS0 |  |
| NKIRAS2 | NFKB inhibitor interacting Ras like 2 | | | Q9NYR9 |  |
| NKRF | NFKB repressing factor | | | O15226 |  |
| NKTR | Natural killer cell triggering receptor | | | P30414 |  |
| NKX3-1 | NK3 homeobox 1 | | | Q99801 |  |
| NLRC5 | NLR family CARD domain containing 5 | | | Q86WI3 |  |
| NLRP1 | NLR family pyrin domain containing 1 | | | Q9C000 |  |
| NLRP3 | NLR family pyrin domain containing 3 | | |  |  |
| NMB | Neuromedin B | | | P08949 |  |
| NMD3 | NMD3 ribosome export adaptor | | | Q96D46 |  |
| NME1 | NME/NM23 nucleoside diphosphate kinase 1 | | | P15531 |  |
| NME2 | NME/NM23 nucleoside diphosphate kinase 2 | | | P22392 |  |
| NME3 | NME/NM23 nucleoside diphosphate kinase 3 | | | Q13232 |  |
| NME4 | NME/NM23 nucleoside diphosphate kinase 4 | | | O00746 |  |
| NME6 | NME/NM23 nucleoside diphosphate kinase 6 | | | O75414 |  |
| NME7 | NME/NM23 family member 7 | | | Q9Y5B8 |  |
| NMI | N-myc and STAT interactor | | | Q13287 |  |
| NMNAT1 | Nicotinamide nucleotide adenylyltransferase 1 | | | Q9HAN9 |  |
| NMNAT3 | Nicotinamide nucleotide adenylyltransferase 3 | | | Q96T66 |  |
| NMRAL1 | NmrA like redox sensor 1 | | | Q9HBL8 |  |
| NMRK1 | Nicotinamide riboside kinase 1 | | | Q9NWW6 |  |
| NMT1 | N-myristoyltransferase 1 | | | P30419 |  |
| NMT2 | N-myristoyltransferase 2 | | | O60551 |  |
| NMU | Neuromedin U | | | P48645 |  |
| NMUR1 | Neuromedin U receptor 1 | | | Q9HB89 |  |
| NOA1 | Nitric oxide associated 1 | | | Q8NC60 |  |
| NOB1 | NIN1 (RPN12) binding protein 1 homolog | | | Q9ULX3 |  |
| NOC3L | NOC3 like DNA replication regulator | | | Q8WTT2 |  |
| NOC4L | Nucleolar complex associated 4 homolog | | | Q9BVI4 |  |
| NOD1 | Nucleotide binding oligomerization domain containing 1 | | | Q9Y239 |  |
| NOD2 | Nucleotide binding oligomerization domain containing 2 | | | Q9HC29 |  |
| NOG | Noggin | | | Q13253 |  |
| NOG | Noggin | | | Q13253 |  |
| NOL12 | Nucleolar protein 12 | | | Q9UGY1 |  |
| NOL3 | Nucleolar protein 3 | | | O60936 |  |
| NOL4L | Nucleolar protein 4 like | | | Q96MY1 |  |
| NOL6 | Nucleolar protein 6 | | | Q9H6R4 |  |
| NOL7 | Nucleolar protein 7 | | | Q9UMY1 |  |
| NOL9 | Nucleolar protein 9 | | | Q5SY16 |  |
| NOLC1 | Nucleolar and coiled-body phosphoprotein 1 | | | Q14978 |  |
| NOMO2 | NODAL modulator 2 | | | Q5JPE7 |  |
| NONO | Non-POU domain containing octamer binding | | | Q15233 |  |
| NOP10 | NOP10 ribonucleoprotein | | | Q9NPE3 |  |
| NOP14 | NOP14 nucleolar protein | | | P78316 |  |
| NOP16 | NOP16 nucleolar protein | | | Q9Y3C1 |  |
| NOP2 | NOP2 nucleolar protein | | | P46087 |  |
| NOP58 | NOP58 ribonucleoprotein | | | Q9Y2X3 |  |
| NOS3 | Nitric oxide synthase 3 | | | P29474 |  |
| NOSIP | Nitric oxide synthase interacting protein | | | Q9Y314 |  |
| NPC2 | NPC intracellular cholesterol transporter 2 | | | P61916 |  |
| NPDC1 | Neural proliferation, differentiation and control 1 | | | Q9NQX5 |  |
| NPEPL1 | Aminopeptidase like 1 | | | Q8NDH3 |  |
| NPL | N-acetylneuraminate pyruvate lyase | | | Q9BXD5 |  |
| NPLOC4 | NPL4 homolog, ubiquitin recognition factor | | | Q8TAT6 |  |
| NPM1 | Nucleophosmin 1 | | | P06748 |  |
| NPM3 | Nucleophosmin/nucleoplasmin 3 | | | O75607 |  |
| NPRL2 | NPR2 like, GATOR1 complex subunit | | | Q8WTW4 |  |
| NPTN | Neuroplastin | | | Q9Y639 |  |
| NQO1 | NAD(P)H quinone dehydrogenase 1 | | | P15559 |  |
| NQO2 | N-ribosyldihydronicotinamide:quinone reductase 2 | | | P16083 |  |
| NR1D1 | Nuclear receptor subfamily 1 group D member 1 | | | P20393 |  |
| NR1H2 | Nuclear receptor subfamily 1 group H member 2 | | | P55055 |  |
| NR1H3 | Nuclear receptor subfamily 1 group H member 3 | | | Q13133 |  |
| NR2C1 | Nuclear receptor subfamily 2 group C member 1 | | | P13056 |  |
| NR2C2AP | Nuclear receptor 2C2 associated protein | | | Q86WQ0 |  |
| NR2F6 | Nuclear receptor subfamily 2 group F member 6 | | | P10588 |  |
| NR3C1 | Nuclear receptor subfamily 3 group C member 1 | | | P04150 |  |
| NR3C2 | Nuclear receptor subfamily 3 group C member 2 | | | P08235 |  |
| NRAS | NRAS proto-oncogene, GTPase | | | P01111 |  |
| NRBF2 | Nuclear receptor binding factor 2 | | | Q96F24 |  |
| NRBP1 | Nuclear receptor binding protein 1 | | | Q9UHY1 |  |
| NREP | Neuronal regeneration related protein | | | Q16612 |  |
| NRF1 | Nuclear respiratory factor 1 | | | Q16656 |  |
| NRM | Nurim | | | Q8IXM6 |  |
| NRSN2 | Neurensin 2 | | | Q9GZP1 |  |
| NSA2 | NSA2 ribosome biogenesis factor | | | O95478 |  |
| NSDHL | NAD(P) dependent steroid dehydrogenase-like | | | Q15738 |  |
| NSF | N-ethylmaleimide sensitive factor, vesicle fusing ATPase | | | P46459 |  |
| NSFL1C | NSFL1 cofactor | | | Q9UNZ2 |  |
| NSG1 | Neuronal vesicle trafficking associated 1 | | | P42857 |  |
| NSG1 | Neuronal vesicle trafficking associated 1 | | | P42857 |  |
| NSL1 | NSL1 component of MIS12 kinetochore complex | | | Q96IY1 |  |
| NSMCE1 | NSE1 homolog, SMC5-SMC6 complex component | | | Q8WV22 |  |
| NSMCE2 | NSE2 (MMS21) homolog, SMC5-SMC6 complex SUMO ligase | | | Q96MF7 |  |
| NSMCE4A | NSE4 homolog A, SMC5-SMC6 complex component | | | Q9NXX6 |  |
| NSRP1 | Nuclear speckle splicing regulatory protein 1 | | | Q9H0G5 |  |
| NSUN3 | NOP2/Sun RNA methyltransferase 3 | | | Q9H649 |  |
| NSUN4 | NOP2/Sun RNA methyltransferase 4 | | | Q96CB9 |  |
| NSUN5 | NOP2/Sun RNA methyltransferase 5 | | | Q96P11 |  |
| NSUN6 | NOP2/Sun RNA methyltransferase 6 | | | Q8TEA1 |  |
| NT5C | 5', 3'-nucleotidase, cytosolic | | | Q8TCD5 |  |
| NT5C2 | 5'-nucleotidase, cytosolic II | | | P49902 |  |
| NT5C3A | 5'-nucleotidase, cytosolic IIIA | | | Q9H0P0 |  |
| NT5C3B | 5'-nucleotidase, cytosolic IIIB | | | Q969T7 |  |
| NT5DC1 | 5'-nucleotidase domain containing 1 | | | Q5TFE4 |  |
| NT5E | 5'-nucleotidase ecto | | | P21589 |  |
| NTAN1 | N-terminal asparagine amidase | | | Q96AB6 |  |
| NTHL1 | Nth like DNA glycosylase 1 | | | P78549 |  |
| NTMT1 | N-terminal Xaa-Pro-Lys N-methyltransferase 1 | | | Q9BV86 |  |
| NTPCR | Nucleoside-triphosphatase, cancer-related | | | Q9BSD7 |  |
| NUAK2 | NUAK family kinase 2 | | | Q9H093 |  |
| NUB1 | Negative regulator of ubiquitin like proteins 1 | | | Q9Y5A7 |  |
| NUBP1 | NUBP iron-sulfur cluster assembly factor 1, cytosolic | | | P53384 |  |
| NUBP2 | NUBP iron-sulfur cluster assembly factor 2, cytosolic | | | Q9Y5Y2 |  |
| NUBPL | NUBP iron-sulfur cluster assembly factor, mitochondrial | | | Q8TB37 |  |
| NUCB1 | Nucleobindin 1 | | | Q02818 |  |
| NUCKS1 | Nuclear casein kinase and cyclin dependent kinase substrate 1 | | | Q9H1E3 |  |
| NUDC | Nuclear distribution C, dynein complex regulator | | | Q9Y266 |  |
| NUDCD1 | NudC domain containing 1 | | | Q96RS6 |  |
| NUDCD2 | NudC domain containing 2 | | | Q8WVJ2 |  |
| NUDT1 | Nudix hydrolase 1 | | | P36639 |  |
| NUDT14 | Nudix hydrolase 14 | | | O95848 |  |
| NUDT15 | Nudix hydrolase 15 | | | Q9NV35 |  |
| NUDT16 | Nudix hydrolase 16 | | | Q96DE0 |  |
| NUDT16L1 | Nudix hydrolase 16 like 1 | | | Q9BRJ7 |  |
| NUDT18 | Nudix hydrolase 18 | | | Q6ZVK8 |  |
| NUDT2 | Nudix hydrolase 2 | | | P50583 |  |
| NUDT21 | Nudix hydrolase 21 | | | O43809 |  |
| NUDT22 | Nudix hydrolase 22 | | | Q9BRQ3 |  |
| NUDT3 | Nudix hydrolase 3 | | | O95989 |  |
| NUDT4 | Nudix hydrolase 4 | | | Q9NZJ9 |  |
| NUDT5 | Nudix hydrolase 5 | | | Q9UKK9 |  |
| NUDT6 | Nudix hydrolase 6 | | | P53370 |  |
| NUDT7 | Nudix hydrolase 7 | | | P0C024 |  |
| NUDT8 | Nudix hydrolase 8 | | | Q8WV74 |  |
| NUDT9 | Nudix hydrolase 9 | | | Q9BW91 |  |
| NUF2 | NUF2 component of NDC80 kinetochore complex | | | Q9BZD4 |  |
| NUFIP2 | Nuclear FMR1 interacting protein 2 | | | Q7Z417 |  |
| NUGGC | Nuclear GTPase, germinal center associated | | | Q68CJ6 |  |
| NUMA1 | Nuclear mitotic apparatus protein 1 | | | Q14980 |  |
| NUMB | NUMB endocytic adaptor protein | | | P49757 |  |
| NUP107 | Nucleoporin 107 | | | P57740 |  |
| NUP133 | Nucleoporin 133 | | | Q8WUM0 |  |
| NUP160 | Nucleoporin 160 | | | Q12769 |  |
| NUP188 | Nucleoporin 188 | | | Q5SRE5 |  |
| NUP210 | Nucleoporin 210 | | | Q8TEM1 |  |
| NUP35 | Nucleoporin 35 | | | Q8NFH5 |  |
| NUP37 | Nucleoporin 37 | | | Q8NFH4 |  |
| NUP43 | Nucleoporin 43 | | | Q8NFH3 |  |
| NUP50 | Nucleoporin 50 | | | Q9UKX7 |  |
| NUP54 | Nucleoporin 54 | | | Q7Z3B4 |  |
| NUP58 | Nucleoporin 58 | | | Q9BVL2 |  |
| NUP62 | Nucleoporin 62 | | | P37198 |  |
| NUP85 | Nucleoporin 85 | | | Q9BW27 |  |
| NUP88 | Nucleoporin 88 | | | Q99567 |  |
| NUP98 | Nucleoporin 98 and 96 precursor | | | P52948 |  |
| NUS1 | NUS1 dehydrodolichyl diphosphate synthase subunit | | | Q96E22 |  |
| NUTF2 | Nuclear transport factor 2 | | | P61970 |  |
| NVL | Nuclear VCP like | | | O15381 |  |
| NXF1 | Nuclear RNA export factor 1 | | | Q9UBU9 |  |
| NXPE3 | Neurexophilin and PC-esterase domain family member 3 | | | Q969Y0 |  |
| NXT1 | Nuclear transport factor 2 like export factor 1 | | | Q9UKK6 |  |
| NXT2 | Nuclear transport factor 2 like export factor 2 | | | Q9NPJ8 |  |
| OARD1 | O-acyl-ADP-ribose deacylase 1 | | | Q9Y530 |  |
| OAS1 | 2'-5'-oligoadenylate synthetase 1 | | | P00973 |  |
| OAS2 | 2'-5'-oligoadenylate synthetase 2 | | | P29728 |  |
| OASL | 2'-5'-oligoadenylate synthetase like | | | Q15646 |  |
| OAT | Ornithine aminotransferase | | | P04181 |  |
| OCEL1 | Occludin/ELL domain containing 1 | | | Q9H607 |  |
| OCIAD1 | OCIA domain containing 1 | | | Q9NX40 |  |
| OCIAD2 | OCIA domain containing 2 | | | Q56VL3 |  |
| OCM | Oncomodulin | | | P0CE72 |  |
| ODC1 | Ornithine decarboxylase 1 | | | P11926 |  |
| ODF2 | Outer dense fiber of sperm tails 2 | | | Q5BJF6 |  |
| ODF2L | Outer dense fiber of sperm tails 2 like | | | Q9ULJ1 |  |
| ODF3B | Outer dense fiber of sperm tails 3B | | | A8MYP8 |  |
| ODR4 | Odr-4 GPCR localization factor homolog | | | Q5SWX8 |  |
| OGDH | Oxoglutarate dehydrogenase | | | Q02218 |  |
| OGFOD1 | 2-oxoglutarate and iron dependent oxygenase domain containing 1 | | | Q8N543 |  |
| OGFOD2 | 2-oxoglutarate and iron dependent oxygenase domain containing 2 | | | Q6N063 |  |
| OGFOD3 | 2-oxoglutarate and iron dependent oxygenase domain containing 3 | | | Q6PK18 |  |
| OGG1 | 8-oxoguanine DNA glycosylase | | | O15527 |  |
| OGT | O-linked N-acetylglucosamine (GlcNAc) transferase | | | O15294 |  |
| OIP5 | Opa interacting protein 5 | | | O43482 |  |
| OLA1 | Obg like ATPase 1 | | | Q9NTK5 |  |
| OLFM2 | Olfactomedin 2 | | | O95897 |  |
| OMA1 | OMA1 zinc metallopeptidase | | | Q96E52 |  |
| OPA1 | OPA1 mitochondrial dynamin like GTPase | | | O60313 |  |
| OPA3 | Outer mitochondrial membrane lipid metabolism regulator OPA3 | | | Q9H6K4 |  |
| OPTN | Optineurin | | | Q96CV9 |  |
| OR4D1 | Olfactory receptor family 4 subfamily D member 1 | | | Q15615 |  |
| ORAI1 | ORAI calcium release-activated calcium modulator 1 | | | Q96D31 |  |
| ORAI2 | ORAI calcium release-activated calcium modulator 2 | | | Q96SN7 |  |
| ORAI3 | ORAI calcium release-activated calcium modulator 3 | | | Q9BRQ5 |  |
| ORC2 | Origin recognition complex subunit 2 | | | Q13416 |  |
| ORC3 | Origin recognition complex subunit 3 | | | Q9UBD5 |  |
| ORC4 | Origin recognition complex subunit 4 | | | O43929 |  |
| ORC5 | Origin recognition complex subunit 5 | | | O43913 |  |
| ORMDL1 | ORMDL sphingolipid biosynthesis regulator 1 | | | Q9P0S3 |  |
| ORMDL2 | ORMDL sphingolipid biosynthesis regulator 2 | | | Q53FV1 |  |
| ORMDL3 | ORMDL sphingolipid biosynthesis regulator 3 | | | Q8N138 |  |
| OS9 | OS9 endoplasmic reticulum lectin | | | Q13438 |  |
| OSBPL11 | Oxysterol binding protein like 11 | | | Q9BXB4 |  |
| OSBPL3 | Oxysterol binding protein like 3 | | | Q9H4L5 |  |
| OSBPL8 | Oxysterol binding protein like 8 | | | Q9BZF1 |  |
| OSER1 | Oxidative stress responsive serine rich 1 | | | Q9NX31 |  |
| OSGEP | O-sialoglycoprotein endopeptidase | | | Q9NPF4 |  |
| OSGEPL1 | O-sialoglycoprotein endopeptidase like 1 | | | Q9H4B0 |  |
| OSGIN2 | Oxidative stress induced growth inhibitor family member 2 | | | Q9Y236 |  |
| OSM | Oncostatin M | | | P13725 |  |
| OSTC | Oligosaccharyltransferase complex non-catalytic subunit | | | Q9NRP0 |  |
| OSTF1 | Osteoclast stimulating factor 1 | | | Q92882 |  |
| OSTM1 | Osteoclastogenesis associated transmembrane protein 1 | | | Q86WC4 |  |
| OTUB1 | OTU deubiquitinase, ubiquitin aldehyde binding 1 | | | Q96FW1 |  |
| OTUD5 | OTU deubiquitinase 5 | | | Q96G74 |  |
| OTUD6B | OTU deubiquitinase 6B | | | Q8N6M0 |  |
| OVCA2 | OVCA2 serine hydrolase domain containing | | | Q8WZ82 |  |
| OXA1L | OXA1L mitochondrial inner membrane protein | | | Q15070 |  |
| OXCT1 | 3-oxoacid CoA-transferase 1 | | | P55809 |  |
| OXLD1 | Oxidoreductase like domain containing 1 | | | Q5BKU9 |  |
| OXNAD1 | Oxidoreductase NAD binding domain containing 1 | | | Q96HP4 |  |
| OXNAD1 | Oxidoreductase NAD binding domain containing 1 | | | Q96HP4 |  |
| OXR1 | Oxidation resistance 1 | | | Q8N573 |  |
| OXSM | 3-oxoacyl-ACP synthase, mitochondrial | | | Q9NWU1 |  |
| OXSR1 | Oxidative stress responsive kinase 1 | | | O95747 |  |
| OXT | Oxytocin/neurophysin I prepropeptide | | | P01178 |  |
| P2RX4 | Purinergic receptor P2X 4 | | | Q99571 |  |
| P2RX5 | Purinergic receptor P2X 5 | | | Q93086 |  |
| P2RY10 | P2Y receptor family member 10 | | | O00398 |  |
| P2RY8 | P2Y receptor family member 8 | | | Q86VZ1 |  |
| P3H1 | Prolyl 3-hydroxylase 1 | | | Q32P28 |  |
| P3H4 | Prolyl 3-hydroxylase family member 4 (inactive) | | | Q92791 |  |
| P4HA1 | Prolyl 4-hydroxylase subunit alpha 1 | | | P13674 |  |
| P4HB | Prolyl 4-hydroxylase subunit beta | | | P07237 |  |
| P4HTM | Prolyl 4-hydroxylase, transmembrane | | | Q9NXG6 |  |
| PA2G4 | Proliferation-associated 2G4 | | | Q9UQ80 |  |
| PAAF1 | Proteasomal ATPase associated factor 1 | | | Q9BRP4 |  |
| PABPC1 | Poly(A) binding protein cytoplasmic 1 | | | P11940 |  |
| PABPC4 | Poly(A) binding protein cytoplasmic 4 | | | Q13310 |  |
| PABPN1 | Poly(A) binding protein nuclear 1 | | | Q86U42 |  |
| PACRG | Parkin coregulated | | | Q96M98 |  |
| PACRGL | Parkin coregulated like | | | Q8N7B6 |  |
| PACS1 | Phosphofurin acidic cluster sorting protein 1 | | | Q6VY07 |  |
| PACSIN2 | Protein kinase C and casein kinase substrate in neurons 2 | | | Q9UNF0 |  |
| PADI4 | Peptidyl arginine deiminase 4 | | | Q9UM07 |  |
| PAF1 | PAF1 homolog, Paf1/RNA polymerase II complex component | | | Q8N7H5 |  |
| PAFAH1B1 | Platelet activating factor acetylhydrolase 1b regulatory subunit 1 | | | P43034 |  |
| PAFAH1B2 | Platelet activating factor acetylhydrolase 1b catalytic subunit 2 | | | P68402 |  |
| PAFAH1B3 | Platelet activating factor acetylhydrolase 1b catalytic subunit 3 | | | Q15102 |  |
| PAFAH2 | Platelet activating factor acetylhydrolase 2 | | | Q99487 |  |
| PAG1 | Phosphoprotein membrane anchor with glycosphingolipid microdomains 1 | | | Q9NWQ8 |  |
| PAGE5 | PAGE family member 5 | | | Q96GU1 |  |
| PAGR1 | PAXIP1 associated glutamate rich protein 1 | | | Q9BTK6 |  |
| PAICS | Phosphoribosylaminoimidazole carboxylase and phosphoribosylaminoimidazolesuccinocarboxamide synthase | | | P22234 |  |
| PAIP1 | Poly(A) binding protein interacting protein 1 | | | Q9H074 |  |
| PAIP2 | Poly(A) binding protein interacting protein 2 | | | Q9BPZ3 |  |
| PAIP2B | Poly(A) binding protein interacting protein 2B | | | Q9ULR5 |  |
| PAK1 | P21 (RAC1) activated kinase 1 | | | Q13153 |  |
| PAK1IP1 | PAK1 interacting protein 1 | | | Q9NWT1 |  |
| PAK2 | P21 (RAC1) activated kinase 2 | | | Q13177 |  |
| PALB2 | Partner and localizer of BRCA2 | | | Q86YC2 |  |
| PAM | Peptidylglycine alpha-amidating monooxygenase | | | P19021 |  |
| PAM16 | Presequence translocase associated motor 16 | | | Q9Y3D7 |  |
| PANK2 | Pantothenate kinase 2 | | | Q9BZ23 |  |
| PANX1 | Pannexin 1 | | | Q96RD7 |  |
| PAPOLA | Poly(A) polymerase alpha | | | P51003 |  |
| PAPOLG | Poly(A) polymerase gamma | | | Q9BWT3 |  |
| PAPSS1 | 3'-phosphoadenosine 5'-phosphosulfate synthase 1 | | | O43252 |  |
| PAQR7 | Progestin and adipoQ receptor family member 7 | | | Q86WK9 |  |
| PAQR8 | Progestin and adipoQ receptor family member 8 | | | Q8TEZ7 |  |
| PARD6A | Par-6 family cell polarity regulator alpha | | | Q9NPB6 |  |
| PARD6B | Par-6 family cell polarity regulator beta | | | Q9BYG5 |  |
| PARG | Poly(ADP-ribose) glycohydrolase | | | Q86W56 |  |
| PARK7 | Parkinsonism associated deglycase | | | Q99497 |  |
| PARL | Presenilin associated rhomboid like | | | Q9H300 |  |
| PARN | Poly(A)-specific ribonuclease | | | O95453 |  |
| PARP1 | Poly(ADP-ribose) polymerase 1 | | | P09874 |  |
| PARP11 | Poly(ADP-ribose) polymerase family member 11 | | | Q9NR21 |  |
| PARP15 | Poly(ADP-ribose) polymerase family member 15 | | | Q460N3 |  |
| PARP16 | Poly(ADP-ribose) polymerase family member 16 | | | Q8N5Y8 |  |
| PARP3 | Poly(ADP-ribose) polymerase family member 3 | | | Q9Y6F1 |  |
| PARP8 | Poly(ADP-ribose) polymerase family member 8 | | | Q8N3A8 |  |
| PARS2 | Prolyl-tRNA synthetase 2, mitochondrial | | | Q7L3T8 |  |
| PARVG | Parvin gamma | | | Q9HBI0 |  |
| PASK | PAS domain containing serine/threonine kinase | | | Q96RG2 |  |
| PATL1 | PAT1 homolog 1, processing body mRNA decay factor | | | Q86TB9 |  |
| PATL2 | PAT1 homolog 2 | | | C9JE40 |  |
| PATZ1 | POZ/BTB and AT hook containing zinc finger 1 | | | Q9HBE1 |  |
| PBDC1 | Polysaccharide biosynthesis domain containing 1 | | | Q9BVG4 |  |
| PBLD | Phenazine biosynthesis like protein domain containing | | | P30039 |  |
| PBX3 | PBX homeobox 3 | | | P40426 |  |
| PBX4 | PBX homeobox 4 | | | Q9BYU1 |  |
| PBXIP1 | PBX homeobox interacting protein 1 | | | Q96AQ6 |  |
| PCBD1 | Pterin-4 alpha-carbinolamine dehydratase 1 | | | P61457 |  |
| PCBD2 | Pterin-4 alpha-carbinolamine dehydratase 2 | | | Q9H0N5 |  |
| PCBP1 | Poly(rC) binding protein 1 | | | Q15365 |  |
| PCBP2 | Poly(rC) binding protein 2 | | | Q15366 |  |
| PCBP4 | Poly(rC) binding protein 4 | | | P57723 |  |
| PCCA | Propionyl-CoA carboxylase subunit alpha | | | P05165 |  |
| PCCB | Propionyl-CoA carboxylase subunit beta | | | P05166 |  |
| PCED1A | PC-esterase domain containing 1A | | | Q9H1Q7 |  |
| PCED1B | PC-esterase domain containing 1B | | | Q96HM7 |  |
| PCGF1 | Polycomb group ring finger 1 | | | Q9BSM1 |  |
| PCGF3 | Polycomb group ring finger 3 | | | Q3KNV8 |  |
| PCGF5 | Polycomb group ring finger 5 | | | Q86SE9 |  |
| PCGF6 | Polycomb group ring finger 6 | | | Q9BYE7 |  |
| PCID2 | PCI domain containing 2 | | | Q5JVF3 |  |
| PCIF1 | Phosphorylated CTD interacting factor 1 | | | Q9H4Z3 |  |
| PCK2 | Phosphoenolpyruvate carboxykinase 2, mitochondrial | | | Q16822 |  |
| PCM1 | Pericentriolar material 1 | | | Q15154 |  |
| PCMT1 | Protein-L-isoaspartate (D-aspartate) O-methyltransferase | | | P22061 |  |
| PCMTD1 | Protein-L-isoaspartate (D-aspartate) O-methyltransferase domain containing 1 | | | Q96MG8 |  |
| PCMTD2 | Protein-L-isoaspartate (D-aspartate) O-methyltransferase domain containing 2 | | | Q9NV79 |  |
| PCNA | Proliferating cell nuclear antigen | | | P12004 |  |
| PCNP | PEST proteolytic signal containing nuclear protein | | | Q8WW12 |  |
| PCSK1N | Proprotein convertase subtilisin/kexin type 1 inhibitor | | | Q9UHG2 |  |
| PCSK5 | Proprotein convertase subtilisin/kexin type 5 | | | Q92824 |  |
| PCSK7 | Proprotein convertase subtilisin/kexin type 7 | | | Q16549 |  |
| PCTP | Phosphatidylcholine transfer protein | | | Q9UKL6 |  |
| PCYOX1 | Prenylcysteine oxidase 1 | | | Q9UHG3 |  |
| PCYOX1L | Prenylcysteine oxidase 1 like | | | Q8NBM8 |  |
| PCYT1A | Phosphate cytidylyltransferase 1A, choline | | | P49585 |  |
| PCYT2 | Phosphate cytidylyltransferase 2, ethanolamine | | | Q99447 |  |
| PDAP1 | PDGFA associated protein 1 | | | Q13442 |  |
| PDCD1 | Programmed cell death 1 | | | Q15116 |  |
| PDCD10 | Programmed cell death 10 | | | Q9BUL8 |  |
| PDCD11 | Programmed cell death 11 | | | Q14690 |  |
| PDCD2 | Programmed cell death 2 | | | Q16342 |  |
| PDCD2L | Programmed cell death 2 like | | | Q9BRP1 |  |
| PDCD4 | Programmed cell death 4 | | | Q53EL6 |  |
| PDCD5 | Programmed cell death 5 | | | O14737 |  |
| PDCD6 | Programmed cell death 6 | | | O75340 |  |
| PDCD6IP | Programmed cell death 6 interacting protein | | | Q8WUM4 |  |
| PDCD7 | Programmed cell death 7 | | | Q8N8D1 |  |
| PDCL | Phosducin like | | | Q13371 |  |
| PDCL3 | Phosducin like 3 | | | Q9H2J4 |  |
| PDE12 | Phosphodiesterase 12 | | | Q6L8Q7 |  |
| PDE4D | Phosphodiesterase 4D | | | Q08499 |  |
| PDE4DIP | Phosphodiesterase 4D interacting protein | | | Q5VU43 |  |
| PDE6B | Phosphodiesterase 6B | | | P35913 |  |
| PDE6D | Phosphodiesterase 6D | | | O43924 |  |
| PDE6G | Phosphodiesterase 6G | | | P18545 |  |
| PDE7A | Phosphodiesterase 7A | | | Q13946 |  |
| PDE9A | Phosphodiesterase 9A | | | O76083 |  |
| PDF | Peptide deformylase, mitochondrial | | | Q9HBH1 |  |
| PDGFA | Platelet derived growth factor subunit A | | | P04085 |  |
| PDGFB | Platelet derived growth factor subunit B | | | P01127 |  |
| PDGFD | Platelet derived growth factor D | | | Q9GZP0 |  |
| PDHA1 | Pyruvate dehydrogenase E1 subunit alpha 1 | | | P08559 |  |
| PDHB | Pyruvate dehydrogenase E1 subunit beta | | | P11177 |  |
| PDHX | Pyruvate dehydrogenase complex component X | | | O00330 |  |
| PDIA3 | Protein disulfide isomerase family A member 3 | | | P30101 |  |
| PDIA4 | Protein disulfide isomerase family A member 4 | | | P13667 |  |
| PDIA5 | Protein disulfide isomerase family A member 5 | | | Q14554 |  |
| PDIA6 | Protein disulfide isomerase family A member 6 | | | Q15084 |  |
| PDIK1L | PDLIM1 interacting kinase 1 like | | | Q8N165 |  |
| PDK1 | Pyruvate dehydrogenase kinase 1 | | | Q15118 |  |
| PDK2 | Pyruvate dehydrogenase kinase 2 | | | Q15119 |  |
| PDK3 | Pyruvate dehydrogenase kinase 3 | | | Q15120 |  |
| PDLIM1 | PDZ and LIM domain 1 | | | O00151 |  |
| PDLIM2 | PDZ and LIM domain 2 | | | Q96JY6 |  |
| PDLIM5 | PDZ and LIM domain 5 | | | Q96HC4 |  |
| PDLIM7 | PDZ and LIM domain 7 | | | Q9NR12 |  |
| PDP1 | Pyruvate dehydrogenase phosphatase catalytic subunit 1 | | | Q9P0J1 |  |
| PDP2 | Pyruvate dehydrogenase phosphatase catalytic subunit 2 | | | Q9P2J9 |  |
| PDPK1 | 3-phosphoinositide dependent protein kinase 1 | | | O15530 |  |
| PDPR | Pyruvate dehydrogenase phosphatase regulatory subunit | | | Q8NCN5 |  |
| PDRG1 | P53 and DNA damage regulated 1 | | | Q9NUG6 |  |
| PDS5A | PDS5 cohesin associated factor A | | | Q29RF7 |  |
| PDS5B | PDS5 cohesin associated factor B | | | Q9NTI5 |  |
| PDSS1 | Decaprenyl diphosphate synthase subunit 1 | | | Q5T2R2 |  |
| PDSS2 | Decaprenyl diphosphate synthase subunit 2 | | | Q86YH6 |  |
| PDXDC1 | Pyridoxal dependent decarboxylase domain containing 1 | | | Q6P996 |  |
| PDZD11 | PDZ domain containing 11 | | | Q5EBL8 |  |
| PEA15 | Proliferation and apoptosis adaptor protein 15 | | | Q15121 |  |
| PECAM1 | Platelet and endothelial cell adhesion molecule 1 | | | P16284 |  |
| PECR | Peroxisomal trans-2-enoyl-CoA reductase | | | Q9BY49 |  |
| PEF1 | Penta-EF-hand domain containing 1 | | | Q9UBV8 |  |
| PELI1 | Pellino E3 ubiquitin protein ligase 1 | | | Q96FA3 |  |
| PELO | Pelota mRNA surveillance and ribosome rescue factor | | | Q9BRX2 |  |
| PEMT | Phosphatidylethanolamine N-methyltransferase | | | Q9UBM1 |  |
| PEPD | Peptidase D | | | P12955 |  |
| PER2 | Period circadian regulator 2 | | | O15055 |  |
| PERP | P53 apoptosis effector related to PMP22 | | | Q96FX8 |  |
| PES1 | Pescadillo ribosomal biogenesis factor 1 | | | O00541 |  |
| PEX1 | Peroxisomal biogenesis factor 1 | | | O43933 |  |
| PEX10 | Peroxisomal biogenesis factor 10 | | | O60683 |  |
| PEX11A | Peroxisomal biogenesis factor 11 alpha | | | O75192 |  |
| PEX11B | Peroxisomal biogenesis factor 11 beta | | | O96011 |  |
| PEX11G | Peroxisomal biogenesis factor 11 gamma | | | Q96HA9 |  |
| PEX12 | Peroxisomal biogenesis factor 12 | | | O00623 |  |
| PEX13 | Peroxisomal biogenesis factor 13 | | | Q92968 |  |
| PEX14 | Peroxisomal biogenesis factor 14 | | | O75381 |  |
| PEX16 | Peroxisomal biogenesis factor 16 | | | Q9Y5Y5 |  |
| PEX19 | Peroxisomal biogenesis factor 19 | | | P40855 |  |
| PEX2 | Peroxisomal biogenesis factor 2 | | | P28328 |  |
| PEX26 | Peroxisomal biogenesis factor 26 | | | Q7Z412 |  |
| PEX3 | Peroxisomal biogenesis factor 3 | | | P56589 |  |
| PEX5 | Peroxisomal biogenesis factor 5 | | | P50542 |  |
| PEX6 | Peroxisomal biogenesis factor 6 | | | Q13608 |  |
| PEX7 | Peroxisomal biogenesis factor 7 | | | O00628 |  |
| PFAS | Phosphoribosylformylglycinamidine synthase | | | O15067 |  |
| PFDN1 | Prefoldin subunit 1 | | | O60925 |  |
| PFDN2 | Prefoldin subunit 2 | | | Q9UHV9 |  |
| PFDN4 | Prefoldin subunit 4 | | | Q9NQP4 |  |
| PFDN5 | Prefoldin subunit 5 | | | Q99471 |  |
| PFDN6 | Prefoldin subunit 6 | | | O15212 |  |
| PFKFB3 | 6-phosphofructo-2-kinase/fructose-2,6-biphosphatase 3 | | | Q16875 |  |
| PFKFB4 | 6-phosphofructo-2-kinase/fructose-2,6-biphosphatase 4 | | | Q16877 |  |
| PFKL | Phosphofructokinase, liver type | | | P17858 |  |
| PFKM | Phosphofructokinase, muscle | | | P08237 |  |
| PFKP | Phosphofructokinase, platelet | | | Q01813 |  |
| PFN1 | Profilin 1 | | | P07737 |  |
| PFN2 | Profilin 2 | | | P35080 |  |
| PGAM1 | Phosphoglycerate mutase 1 | | | P18669 |  |
| PGAM5 | PGAM family member 5, mitochondrial serine/threonine protein phosphatase | | | Q96HS1 |  |
| PGAP2 | Post-GPI attachment to proteins 2 | | | Q9UHJ9 |  |
| PGAP3 | Post-GPI attachment to proteins phospholipase 3 | | | Q96FM1 |  |
| PGBD2 | PiggyBac transposable element derived 2 | | | Q6P3X8 |  |
| PGD | Phosphogluconate dehydrogenase | | | P52209 |  |
| PGGT1B | Protein geranylgeranyltransferase type I subunit beta | | | P53609 |  |
| PGK1 | Phosphoglycerate kinase 1 | | | P00558 |  |
| PGLS | 6-phosphogluconolactonase | | | O95336 |  |
| PGLYRP2 | Peptidoglycan recognition protein 2 | | | Q96PD5 |  |
| PGM1 | Phosphoglucomutase 1 | | | P36871 |  |
| PGM2 | Phosphoglucomutase 2 | | | Q96G03 |  |
| PGM2L1 | Phosphoglucomutase 2 like 1 | | | Q6PCE3 |  |
| PGM3 | Phosphoglucomutase 3 | | | O95394 |  |
| PGPEP1 | Pyroglutamyl-peptidase I | | | Q9NXJ5 |  |
| PGRMC1 | Progesterone receptor membrane component 1 | | | O00264 |  |
| PGRMC2 | Progesterone receptor membrane component 2 | | | O15173 |  |
| PGS1 | Phosphatidylglycerophosphate synthase 1 | | | Q32NB8 |  |
| PHACTR2 | Phosphatase and actin regulator 2 | | | O75167 |  |
| PHACTR4 | Phosphatase and actin regulator 4 | | | Q8IZ21 |  |
| PHAX | Phosphorylated adaptor for RNA export | | | Q9H814 |  |
| PHB2 | Prohibitin 2 | | | Q99623 |  |
| PHC1 | Polyhomeotic homolog 1 | | | P78364 |  |
| PHC2 | Polyhomeotic homolog 2 | | | Q8IXK0 |  |
| PHC3 | Polyhomeotic homolog 3 | | | Q8NDX5 |  |
| PHF1 | PHD finger protein 1 | | | O43189 |  |
| PHF10 | PHD finger protein 10 | | | Q8WUB8 |  |
| PHF11 | PHD finger protein 11 | | | Q9UIL8 |  |
| PHF13 | PHD finger protein 13 | | | Q86YI8 |  |
| PHF14 | PHD finger protein 14 | | | O94880 |  |
| PHF19 | PHD finger protein 19 | | | Q5T6S3 |  |
| PHF2 | PHD finger protein 2 | | | O75151 |  |
| PHF20 | PHD finger protein 20 | | | Q9BVI0 |  |
| PHF20L1 | PHD finger protein 20 like 1 | | | A8MW92 |  |
| PHF21A | PHD finger protein 21A | | | Q96BD5 |  |
| PHF23 | PHD finger protein 23 | | | Q9BUL5 |  |
| PHF3 | PHD finger protein 3 | | | Q92576 |  |
| PHF5A | PHD finger protein 5A | | | Q7RTV0 |  |
| PHF6 | PHD finger protein 6 | | | Q8IWS0 |  |
| PHF7 | PHD finger protein 7 | | | Q9BWX1 |  |
| PHF8 | PHD finger protein 8 | | | Q9UPP1 |  |
| PHGDH | Phosphoglycerate dehydrogenase | | | O43175 |  |
| PHKA2 | Phosphorylase kinase regulatory subunit alpha 2 | | | P46019 |  |
| PHKB | Phosphorylase kinase regulatory subunit beta | | | Q93100 |  |
| PHKG2 | Phosphorylase kinase catalytic subunit gamma 2 | | | P15735 |  |
| PHLDA1 | Pleckstrin homology like domain family A member 1 | | | Q8WV24 |  |
| PHLDA2 | Pleckstrin homology like domain family A member 2 | | | Q53GA4 |  |
| PHOSPHO2 | Phosphatase, orphan 2 | | | Q8TCD6 |  |
| PHPT1 | Phosphohistidine phosphatase 1 | | | Q9NRX4 |  |
| PHTF1 | Putative homeodomain transcription factor 1 | | | Q9UMS5 |  |
| PHTF2 | Putative homeodomain transcription factor 2 | | | Q8N3S3 |  |
| PHYH | Phytanoyl-CoA 2-hydroxylase | | | O14832 |  |
| PHYHD1 | Phytanoyl-CoA dioxygenase domain containing 1 | | | Q5SRE7 |  |
| PHYKPL | 5-phosphohydroxy-L-lysine phospho-lyase | | | Q8IUZ5 |  |
| PI16 | Peptidase inhibitor 16 | | | Q6UXB8 |  |
| PI4K2B | Phosphatidylinositol 4-kinase type 2 beta | | | Q8TCG2 |  |
| PI4KA | Phosphatidylinositol 4-kinase alpha | | | P42356 |  |
| PI4KB | Phosphatidylinositol 4-kinase beta | | | Q9UBF8 |  |
| PIAS1 | Protein inhibitor of activated STAT 1 | | | O75925 |  |
| PIAS2 | Protein inhibitor of activated STAT 2 | | | O75928 |  |
| PIAS3 | Protein inhibitor of activated STAT 3 | | | Q9Y6X2 |  |
| PIAS4 | Protein inhibitor of activated STAT 4 | | | Q8N2W9 |  |
| PIBF1 | Progesterone immunomodulatory binding factor 1 | | | Q8WXW3 |  |
| PICALM | Phosphatidylinositol binding clathrin assembly protein | | | Q13492 |  |
| PICK1 | Protein interacting with PRKCA 1 | | | Q9NRD5 |  |
| PIEZO1 | Piezo type mechanosensitive ion channel component 1 | | | Q92508 |  |
| PIGA | Phosphatidylinositol glycan anchor biosynthesis class A | | | P37287 |  |
| PIGB | Phosphatidylinositol glycan anchor biosynthesis class B | | | Q92521 |  |
| PIGC | Phosphatidylinositol glycan anchor biosynthesis class C | | | Q92535 |  |
| PIGF | Phosphatidylinositol glycan anchor biosynthesis class F | | | Q07326 |  |
| PIGG | Phosphatidylinositol glycan anchor biosynthesis class G | | | Q5H8A4 |  |
| PIGH | Phosphatidylinositol glycan anchor biosynthesis class H | | | Q14442 |  |
| PIGK | Phosphatidylinositol glycan anchor biosynthesis class K | | | Q92643 |  |
| PIGL | Phosphatidylinositol glycan anchor biosynthesis class L | | | Q9Y2B2 |  |
| PIGM | Phosphatidylinositol glycan anchor biosynthesis class M | | | Q9H3S5 |  |
| PIGN | Phosphatidylinositol glycan anchor biosynthesis class N | | | O95427 |  |
| PIGO | Phosphatidylinositol glycan anchor biosynthesis class O | | | Q8TEQ8 |  |
| PIGP | Phosphatidylinositol glycan anchor biosynthesis class P | | | P57054 |  |
| PIGQ | Phosphatidylinositol glycan anchor biosynthesis class Q | | | Q9BRB3 |  |
| PIGS | Phosphatidylinositol glycan anchor biosynthesis class S | | | Q96S52 |  |
| PIGT | Phosphatidylinositol glycan anchor biosynthesis class T | | | Q969N2 |  |
| PIGU | Phosphatidylinositol glycan anchor biosynthesis class U | | | Q9H490 |  |
| PIGV | Phosphatidylinositol glycan anchor biosynthesis class V | | | Q9NUD9 |  |
| PIGX | Phosphatidylinositol glycan anchor biosynthesis class X | | | Q8TBF5 |  |
| PIH1D1 | PIH1 domain containing 1 | | | Q9NWS0 |  |
| PIK3C2A | Phosphatidylinositol-4-phosphate 3-kinase catalytic subunit type 2 alpha | | | O00443 |  |
| PIK3C3 | Phosphatidylinositol 3-kinase catalytic subunit type 3 | | | Q8NEB9 |  |
| PIK3CA | Phosphatidylinositol-4,5-bisphosphate 3-kinase catalytic subunit alpha | | | P42336 |  |
| PIK3CB | Phosphatidylinositol-4,5-bisphosphate 3-kinase catalytic subunit beta | | | P42338 |  |
| PIK3CD | Phosphatidylinositol-4,5-bisphosphate 3-kinase catalytic subunit delta | | | O00329 |  |
| PIK3CG | Phosphatidylinositol-4,5-bisphosphate 3-kinase catalytic subunit gamma | | | P48736 |  |
| PIK3IP1 | Phosphoinositide-3-kinase interacting protein 1 | | | Q96FE7 |  |
| PIK3IP1 | Phosphoinositide-3-kinase interacting protein 1 | | | Q96FE7 |  |
| PIK3R1 | Phosphoinositide-3-kinase regulatory subunit 1 | | | P27986 |  |
| PIK3R4 | Phosphoinositide-3-kinase regulatory subunit 4 | | | Q99570 |  |
| PIK3R5 | Phosphoinositide-3-kinase regulatory subunit 5 | | | Q8WYR1 |  |
| PIKFYVE | Phosphoinositide kinase, FYVE-type zinc finger containing | | | Q9Y2I7 |  |
| PILRA | Paired immunoglobin like type 2 receptor alpha | | | Q9UKJ1 |  |
| PILRB | Paired immunoglobin like type 2 receptor beta | | | Q9UKJ0 |  |
| PIM1 | Pim-1 proto-oncogene, serine/threonine kinase | | | P11309 |  |
| PIM2 | Pim-2 proto-oncogene, serine/threonine kinase | | | Q9P1W9 |  |
| PIM3 | Pim-3 proto-oncogene, serine/threonine kinase | | | Q86V86 |  |
| PIN1 | Peptidylprolyl cis/trans isomerase, NIMA-interacting 1 | | | Q13526 |  |
| PIN4 | Peptidylprolyl cis/trans isomerase, NIMA-interacting 4 | | | Q9Y237 |  |
| PINX1 | PIN2 (TERF1) interacting telomerase inhibitor 1 | | | Q96BK5 |  |
| PIP4K2A | Phosphatidylinositol-5-phosphate 4-kinase type 2 alpha | | | P48426 |  |
| PIP4K2B | Phosphatidylinositol-5-phosphate 4-kinase type 2 beta | | | P78356 |  |
| PIP4K2C | Phosphatidylinositol-5-phosphate 4-kinase type 2 gamma | | | Q8TBX8 |  |
| PIP5K1A | Phosphatidylinositol-4-phosphate 5-kinase type 1 alpha | | | Q99755 |  |
| PISD | Phosphatidylserine decarboxylase | | | Q9UG56 |  |
| PITHD1 | PITH domain containing 1 | | | Q9GZP4 |  |
| PITPNA | Phosphatidylinositol transfer protein alpha | | | Q00169 |  |
| PITPNB | Phosphatidylinositol transfer protein beta | | | P48739 |  |
| PITPNC1 | Phosphatidylinositol transfer protein cytoplasmic 1 | | | Q9UKF7 |  |
| PITRM1 | Pitrilysin metallopeptidase 1 | | | Q5JRX3 |  |
| PJA2 | Praja ring finger ubiquitin ligase 2 | | | O43164 |  |
| PKD2L2 | Polycystin 2 like 2, transient receptor potential cation channel | | | Q9NZM6 |  |
| PKIA | CAMP-dependent protein kinase inhibitor alpha | | | P61925 |  |
| PKIG | CAMP-dependent protein kinase inhibitor gamma | | | Q9Y2B9 |  |
| PKM | Pyruvate kinase M1/2 | | | P14618 |  |
| PKN1 | Protein kinase N1 | | | Q16512 |  |
| PKN2 | Protein kinase N2 | | | Q16513 |  |
| PKNOX1 | PBX/knotted 1 homeobox 1 | | | P55347 |  |
| PLA2G12A | Phospholipase A2 group XIIA | | | Q9BZM1 |  |
| PLA2G15 | Phospholipase A2 group XV | | | Q8NCC3 |  |
| PLA2G6 | Phospholipase A2 group VI | | | O60733 |  |
| PLAA | Phospholipase A2 activating protein | | | Q9Y263 |  |
| PLAC8 | Placenta associated 8 | | | Q9NZF1 |  |
| PLAG1 | PLAG1 zinc finger | | | Q6DJT9 |  |
| PLAGL1 | PLAG1 like zinc finger 1 | | | Q9UM63 |  |
| PLAGL2 | PLAG1 like zinc finger 2 | | | Q9UPG8 |  |
| PLAUR | Plasminogen activator, urokinase receptor | | | Q03405 |  |
| PLCD1 | Phospholipase C delta 1 | | | P51178 |  |
| PLCL2 | Phospholipase C like 2 | | | Q9UPR0 |  |
| PLCXD1 | Phosphatidylinositol specific phospholipase C X domain containing 1 | | | Q9NUJ7 |  |
| PLD2 | Phospholipase D2 | | | O14939 |  |
| PLD3 | Phospholipase D family member 3 | | | Q8IV08 |  |
| PLD4 | Phospholipase D family member 4 | | | Q96BZ4 |  |
| PLD6 | Phospholipase D family member 6 | | | Q8N2A8 |  |
| PLEK | Pleckstrin | | | P08567 |  |
| PLEK2 | Pleckstrin 2 | | | Q9NYT0 |  |
| PLEKHA1 | Pleckstrin homology domain containing A1 | | | Q9HB21 |  |
| PLEKHA2 | Pleckstrin homology domain containing A2 | | |  |  |
| PLEKHA5 | Pleckstrin homology domain containing A5 | | | Q9HAU0 |  |
| PLEKHB1 | Pleckstrin homology domain containing B1 | | | Q9UF11 |  |
| PLEKHB1 | Pleckstrin homology domain containing B1 | | | Q9UF11 |  |
| PLEKHB2 | Pleckstrin homology domain containing B2 | | | Q96CS7 |  |
| PLEKHF1 | Pleckstrin homology and FYVE domain containing 1 | | | Q96S99 |  |
| PLEKHF2 | Pleckstrin homology and FYVE domain containing 2 | | | Q9H8W4 |  |
| PLEKHG3 | Pleckstrin homology and RhoGEF domain containing G3 | | | A1L390 |  |
| PLEKHJ1 | Pleckstrin homology domain containing J1 | | | Q9NW61 |  |
| PLEKHM3 | Pleckstrin homology domain containing M3 | | | Q6ZWE6 |  |
| PLEKHO1 | Pleckstrin homology domain containing O1 | | | Q53GL0 |  |
| PLEKHO2 | Pleckstrin homology domain containing O2 | | | Q8TD55 |  |
| PLGRKT | Plasminogen receptor with a C-terminal lysine | | | Q9HBL7 |  |
| PLIN2 | Perilipin 2 | | | Q99541 |  |
| PLIN3 | Perilipin 3 | | | O60664 |  |
| PLK3 | Polo like kinase 3 | | | Q9H4B4 |  |
| PLLP | Plasmolipin | | | Q9Y342 |  |
| PLLP | Plasmolipin | | | Q9Y342 |  |
| PLOD1 | Procollagen-lysine,2-oxoglutarate 5-dioxygenase 1 | | | Q02809 |  |
| PLOD3 | Procollagen-lysine,2-oxoglutarate 5-dioxygenase 3 | | | O60568 |  |
| PLP2 | Proteolipid protein 2 | | | Q04941 |  |
| PLPP1 | Phospholipid phosphatase 1 | | | O14494 |  |
| PLPP2 | Phospholipid phosphatase 2 | | | O43688 |  |
| PLPP5 | Phospholipid phosphatase 5 | | | Q8NEB5 |  |
| PLPP6 | Phospholipid phosphatase 6 | | | Q8IY26 |  |
| PLRG1 | Pleiotropic regulator 1 | | | O43660 |  |
| PLSCR1 | Phospholipid scramblase 1 | | | O15162 |  |
| PLSCR3 | Phospholipid scramblase 3 | | | Q9NRY6 |  |
| PLXDC1 | Plexin domain containing 1 | | | Q8IUK5 |  |
| PLXNC1 | Plexin C1 | | | O60486 |  |
| PM20D2 | Peptidase M20 domain containing 2 | | | Q8IYS1 |  |
| PMAIP1 | Phorbol-12-myristate-13-acetate-induced protein 1 | | | Q13794 |  |
| PMCH | Pro-melanin concentrating hormone | | | P20382 |  |
| PMEPA1 | Prostate transmembrane protein, androgen induced 1 | | | Q969W9 |  |
| PMF1 | Polyamine modulated factor 1 | | | Q6P1K2 |  |
| PML | PML nuclear body scaffold | | | P29590 |  |
| PMM1 | Phosphomannomutase 1 | | | Q92871 |  |
| PMM2 | Phosphomannomutase 2 | | | O15305 |  |
| PMPCA | Peptidase, mitochondrial processing subunit alpha | | | Q10713 |  |
| PMPCB | Peptidase, mitochondrial processing subunit beta | | | O75439 |  |
| PMS1 | PMS1 homolog 1, mismatch repair system component | | | P54277 |  |
| PMS2 | PMS1 homolog 2, mismatch repair system component | | | P54278 |  |
| PMVK | Phosphomevalonate kinase | | | Q15126 |  |
| PNKD | PNKD metallo-beta-lactamase domain containing | | | Q8N490 |  |
| PNKP | Polynucleotide kinase 3'-phosphatase | | | Q96T60 |  |
| PNMA1 | PNMA family member 1 | | | Q8ND90 |  |
| PNN | Pinin, desmosome associated protein | | | Q9H307 |  |
| PNO1 | Partner of NOB1 homolog | | | Q9NRX1 |  |
| PNP | Purine nucleoside phosphorylase | | | P00491 |  |
| PNPLA2 | Patatin like phospholipase domain containing 2 | | | Q96AD5 |  |
| PNPLA4 | Patatin like phospholipase domain containing 4 | | | P41247 |  |
| PNPLA6 | Patatin like phospholipase domain containing 6 | | | Q8IY17 |  |
| PNPLA8 | Patatin like phospholipase domain containing 8 | | | Q9NP80 |  |
| PNPO | Pyridoxamine 5'-phosphate oxidase | | | Q9NVS9 |  |
| PNPT1 | Polyribonucleotide nucleotidyltransferase 1 | | | Q8TCS8 |  |
| PNRC2 | Proline rich nuclear receptor coactivator 2 | | | Q9NPJ4 |  |
| POC1B | POC1 centriolar protein B | | | Q8TC44 |  |
| POC5 | POC5 centriolar protein | | | Q8NA72 |  |
| POFUT1 | Protein O-fucosyltransferase 1 | | | Q9H488 |  |
| POGK | Pogo transposable element derived with KRAB domain | | | Q9P215 |  |
| POGLUT1 | Protein O-glucosyltransferase 1 | | | Q8NBL1 |  |
| POGZ | Pogo transposable element derived with ZNF domain | | | Q7Z3K3 |  |
| POLA2 | DNA polymerase alpha 2, accessory subunit | | | Q14181 |  |
| POLB | DNA polymerase beta | | | P06746 |  |
| POLD1 | DNA polymerase delta 1, catalytic subunit | | | P28340 |  |
| POLD2 | DNA polymerase delta 2, accessory subunit | | | P49005 |  |
| POLD3 | DNA polymerase delta 3, accessory subunit | | | Q15054 |  |
| POLD4 | DNA polymerase delta 4, accessory subunit | | | Q9HCU8 |  |
| POLDIP2 | DNA polymerase delta interacting protein 2 | | | Q9Y2S7 |  |
| POLDIP3 | DNA polymerase delta interacting protein 3 | | | Q9BY77 |  |
| POLE3 | DNA polymerase epsilon 3, accessory subunit | | | Q9NRF9 |  |
| POLE4 | DNA polymerase epsilon 4, accessory subunit | | | Q9NR33 |  |
| POLG2 | DNA polymerase gamma 2, accessory subunit | | | Q9UHN1 |  |
| POLH | DNA polymerase eta | | | Q9Y253 |  |
| POLI | DNA polymerase iota | | | Q9UNA4 |  |
| POLK | DNA polymerase kappa | | | Q9UBT6 |  |
| POLL | DNA polymerase lambda | | | Q9UGP5 |  |
| POLR1C | RNA polymerase I and III subunit C | | | O15160 |  |
| POLR1D | RNA polymerase I and III subunit D | | | P0DPB5, P0DPB6 |  |
| POLR1E | RNA polymerase I subunit E | | | Q9GZS1 |  |
| POLR2C | RNA polymerase II subunit C | | | P19387 |  |
| POLR2D | RNA polymerase II subunit D | | | O15514 |  |
| POLR2E | RNA polymerase II, I and III subunit E | | | P19388 |  |
| POLR2F | RNA polymerase II, I and III subunit F | | | P61218 |  |
| POLR2G | RNA polymerase II subunit G | | | P62487 |  |
| POLR2H | RNA polymerase II, I and III subunit H | | | P52434 |  |
| POLR2I | RNA polymerase II subunit I | | | P36954 |  |
| POLR2J | RNA polymerase II subunit J | | | P52435 |  |
| POLR2J2 | RNA polymerase II subunit J2 | | | Q9GZM3 |  |
| POLR2J3 | RNA polymerase II subunit J3 | | |  |  |
| POLR2J3 | RNA polymerase II subunit J3 | | |  |  |
| POLR2K | RNA polymerase II, I and III subunit K | | | P53803 |  |
| POLR2L | RNA polymerase II, I and III subunit L | | | P62875 |  |
| POLR2M | RNA polymerase II subunit M | | | P0CAP2, Q6EEV4 |  |
| POLR3B | RNA polymerase III subunit B | | | Q9NW08 |  |
| POLR3C | RNA polymerase III subunit C | | | Q9BUI4 |  |
| POLR3D | RNA polymerase III subunit D | | | P05423 |  |
| POLR3E | RNA polymerase III subunit E | | | Q9NVU0 |  |
| POLR3F | RNA polymerase III subunit F | | | Q9H1D9 |  |
| POLR3G | RNA polymerase III subunit G | | | O15318 |  |
| POLR3GL | RNA polymerase III subunit GL | | | Q9BT43 |  |
| POLR3H | RNA polymerase III subunit H | | | Q9Y535 |  |
| POLR3K | RNA polymerase III subunit K | | | Q9Y2Y1 |  |
| POLRMT | RNA polymerase mitochondrial | | | O00411 |  |
| POM121 | POM121 transmembrane nucleoporin | | | Q96HA1 |  |
| POMC | Proopiomelanocortin | | | P01189 |  |
| POMGNT2 | Protein O-linked mannose N-acetylglucosaminyltransferase 2 (beta 1,4-) | | | Q8NAT1 |  |
| POMK | Protein O-mannose kinase | | | Q9H5K3 |  |
| POMP | Proteasome maturation protein | | | Q9Y244 |  |
| POMT1 | Protein O-mannosyltransferase 1 | | | Q9Y6A1 |  |
| POMZP3 | POM121 and ZP3 fusion | | | Q6PJE2 |  |
| PON2 | Paraoxonase 2 | | | Q15165 |  |
| POP1 | POP1 homolog, ribonuclease P/MRP subunit | | | Q99575 |  |
| POP4 | POP4 homolog, ribonuclease P/MRP subunit | | | O95707 |  |
| POP5 | POP5 homolog, ribonuclease P/MRP subunit | | | Q969H6 |  |
| POP7 | POP7 homolog, ribonuclease P/MRP subunit | | | O75817 |  |
| POR | Cytochrome p450 oxidoreductase | | | P16435 |  |
| PORCN | Porcupine O-acyltransferase | | | Q9H237 |  |
| POT1 | Protection of telomeres 1 | | | Q9NUX5 |  |
| POTEG | POTE ankyrin domain family member G | | | Q6S5H5 |  |
| POU2F2 | POU class 2 homeobox 2 | | | P09086 |  |
| PPA1 | Inorganic pyrophosphatase 1 | | | Q15181 |  |
| PPA2 | Inorganic pyrophosphatase 2 | | | Q9H2U2 |  |
| PPAN | Peter pan homolog | | | Q9NQ55 |  |
| PPARD | Peroxisome proliferator activated receptor delta | | | Q03181 |  |
| PPAT | Phosphoribosyl pyrophosphate amidotransferase | | | Q06203 |  |
| PPBP | Pro-platelet basic protein | | | P02775 |  |
| PPCDC | Phosphopantothenoylcysteine decarboxylase | | | Q96CD2 |  |
| PPCS | Phosphopantothenoylcysteine synthetase | | | Q9HAB8 |  |
| PPDPF | Pancreatic progenitor cell differentiation and proliferation factor | | | Q9H3Y8 |  |
| PPFIA1 | PTPRF interacting protein alpha 1 | | | Q13136 |  |
| PPFIBP2 | PPFIA binding protein 2 | | | Q8ND30 |  |
| PPHLN1 | Periphilin 1 | | | Q8NEY8 |  |
| PPIA | Peptidylprolyl isomerase A | | | P62937 |  |
| PPIAL4C | Peptidylprolyl isomerase A like 4C | | | A0A0B4J2A2 |  |
| PPIB | Peptidylprolyl isomerase B | | | P23284 |  |
| PPID | Peptidylprolyl isomerase D | | | Q08752 |  |
| PPIE | Peptidylprolyl isomerase E | | | Q9UNP9 |  |
| PPIF | Peptidylprolyl isomerase F | | | P30405 |  |
| PPIG | Peptidylprolyl isomerase G | | | Q13427 |  |
| PPIH | Peptidylprolyl isomerase H | | | O43447 |  |
| PPIL1 | Peptidylprolyl isomerase like 1 | | | Q9Y3C6 |  |
| PPIL2 | Peptidylprolyl isomerase like 2 | | | Q13356 |  |
| PPIL3 | Peptidylprolyl isomerase like 3 | | | Q9H2H8 |  |
| PPIL4 | Peptidylprolyl isomerase like 4 | | | Q8WUA2 |  |
| PPIP5K1 | Diphosphoinositol pentakisphosphate kinase 1 | | | Q6PFW1 |  |
| PPIP5K2 | Diphosphoinositol pentakisphosphate kinase 2 | | | O43314 |  |
| PPM1A | Protein phosphatase, Mg2+/Mn2+ dependent 1A | | | P35813 |  |
| PPM1B | Protein phosphatase, Mg2+/Mn2+ dependent 1B | | | O75688 |  |
| PPM1D | Protein phosphatase, Mg2+/Mn2+ dependent 1D | | | O15297 |  |
| PPM1F | Protein phosphatase, Mg2+/Mn2+ dependent 1F | | | P49593 |  |
| PPM1G | Protein phosphatase, Mg2+/Mn2+ dependent 1G | | | O15355 |  |
| PPM1K | Protein phosphatase, Mg2+/Mn2+ dependent 1K | | | Q8N3J5 |  |
| PPM1M | Protein phosphatase, Mg2+/Mn2+ dependent 1M | | | Q96MI6 |  |
| PPM1N | Protein phosphatase, Mg2+/Mn2+ dependent 1N (putative) | | | Q8N819 |  |
| PPME1 | Protein phosphatase methylesterase 1 | | | Q9Y570 |  |
| PPOX | Protoporphyrinogen oxidase | | | P50336 |  |
| PPP1CA | Protein phosphatase 1 catalytic subunit alpha | | | P62136 |  |
| PPP1CB | Protein phosphatase 1 catalytic subunit beta | | | P62140 |  |
| PPP1CC | Protein phosphatase 1 catalytic subunit gamma | | | P36873 |  |
| PPP1R11 | Protein phosphatase 1 regulatory inhibitor subunit 11 | | | O60927 |  |
| PPP1R12A | Protein phosphatase 1 regulatory subunit 12A | | | O14974 |  |
| PPP1R12B | Protein phosphatase 1 regulatory subunit 12B | | | O60237 |  |
| PPP1R12C | Protein phosphatase 1 regulatory subunit 12C | | | Q9BZL4 |  |
| PPP1R14B | Protein phosphatase 1 regulatory inhibitor subunit 14B | | | Q96C90 |  |
| PPP1R15A | Protein phosphatase 1 regulatory subunit 15A | | | O75807 |  |
| PPP1R16B | Protein phosphatase 1 regulatory subunit 16B | | | Q96T49 |  |
| PPP1R18 | Protein phosphatase 1 regulatory subunit 18 | | | Q6NYC8 |  |
| PPP1R2 | Protein phosphatase 1 regulatory inhibitor subunit 2 | | | P41236 |  |
| PPP1R21 | Protein phosphatase 1 regulatory subunit 21 | | | Q6ZMI0 |  |
| PPP1R35 | Protein phosphatase 1 regulatory subunit 35 | | | Q8TAP8 |  |
| PPP1R3B | Protein phosphatase 1 regulatory subunit 3B | | | Q86XI6 |  |
| PPP1R3F | Protein phosphatase 1 regulatory subunit 3F | | | Q6ZSY5 |  |
| PPP1R7 | Protein phosphatase 1 regulatory subunit 7 | | | Q15435 |  |
| PPP1R8 | Protein phosphatase 1 regulatory subunit 8 | | | Q12972 |  |
| PPP1R9B | Protein phosphatase 1 regulatory subunit 9B | | | Q96SB3 |  |
| PPP2CA | Protein phosphatase 2 catalytic subunit alpha | | | P67775 |  |
| PPP2CB | Protein phosphatase 2 catalytic subunit beta | | | P62714 |  |
| PPP2R1A | Protein phosphatase 2 scaffold subunit Aalpha | | | P30153 |  |
| PPP2R1B | Protein phosphatase 2 scaffold subunit Abeta | | | P30154 |  |
| PPP2R2A | Protein phosphatase 2 regulatory subunit Balpha | | | P63151 |  |
| PPP2R2B | Protein phosphatase 2 regulatory subunit Bbeta | | | Q00005 |  |
| PPP2R3C | Protein phosphatase 2 regulatory subunit B''gamma | | | Q969Q6 |  |
| PPP2R5A | Protein phosphatase 2 regulatory subunit B'alpha | | | Q15172 |  |
| PPP2R5C | Protein phosphatase 2 regulatory subunit B'gamma | | | Q13362 |  |
| PPP2R5D | Protein phosphatase 2 regulatory subunit B'delta | | | Q14738 |  |
| PPP2R5E | Protein phosphatase 2 regulatory subunit B'epsilon | | | Q16537 |  |
| PPP3CA | Protein phosphatase 3 catalytic subunit alpha | | | Q08209 |  |
| PPP3CB | Protein phosphatase 3 catalytic subunit beta | | | P16298 |  |
| PPP3CC | Protein phosphatase 3 catalytic subunit gamma | | | P48454 |  |
| PPP4C | Protein phosphatase 4 catalytic subunit | | | P60510 |  |
| PPP4R1 | Protein phosphatase 4 regulatory subunit 1 | | | Q8TF05 |  |
| PPP4R2 | Protein phosphatase 4 regulatory subunit 2 | | | Q9NY27 |  |
| PPP4R3A | Protein phosphatase 4 regulatory subunit 3A | | | Q6IN85 |  |
| PPP5C | Protein phosphatase 5 catalytic subunit | | | P53041 |  |
| PPP6C | Protein phosphatase 6 catalytic subunit | | | O00743 |  |
| PPP6R2 | Protein phosphatase 6 regulatory subunit 2 | | | O75170 |  |
| PPRC1 | PPARG related coactivator 1 | | | Q5VV67 |  |
| PPT1 | Palmitoyl-protein thioesterase 1 | | | P50897 |  |
| PPWD1 | Peptidylprolyl isomerase domain and WD repeat containing 1 | | | Q96BP3 |  |
| PQBP1 | Polyglutamine binding protein 1 | | | O60828 |  |
| PRADC1 | Protease associated domain containing 1 | | | Q9BSG0 |  |
| PRAF2 | PRA1 domain family member 2 | | | O60831 |  |
| PRC1 | Protein regulator of cytokinesis 1 | | | O43663 |  |
| PRCC | Proline rich mitotic checkpoint control factor | | | Q92733 |  |
| PRCP | Prolylcarboxypeptidase | | | P42785 |  |
| PRDM1 | PR/SET domain 1 | | | O75626 |  |
| PRDM1 | PR/SET domain 1 | | | O75626 |  |
| PRDM2 | PR/SET domain 2 | | | Q13029 |  |
| PRDM4 | PR/SET domain 4 | | | Q9UKN5 |  |
| PRDX1 | Peroxiredoxin 1 | | | Q06830 |  |
| PRDX2 | Peroxiredoxin 2 | | | P32119 |  |
| PRDX3 | Peroxiredoxin 3 | | | P30048 |  |
| PRDX4 | Peroxiredoxin 4 | | | Q13162 |  |
| PRDX5 | Peroxiredoxin 5 | | | P30044 |  |
| PRDX6 | Peroxiredoxin 6 | | | P30041 |  |
| PREB | Prolactin regulatory element binding | | | Q9HCU5 |  |
| PRELID1 | PRELI domain containing 1 | | | Q9Y255 |  |
| PRELID2 | PRELI domain containing 2 | | | Q8N945 |  |
| PRELID3B | PRELI domain containing 3B | | | Q9Y3B1 |  |
| PREP | Prolyl endopeptidase | | | P48147 |  |
| PREPL | Prolyl endopeptidase like | | | Q4J6C6 |  |
| PREX1 | Phosphatidylinositol-3,4,5-trisphosphate dependent Rac exchange factor 1 | | | Q8TCU6 |  |
| PRF1 | Perforin 1 | | | P14222 |  |
| PRICKLE3 | Prickle planar cell polarity protein 3 | | | O43900 |  |
| PRIM1 | DNA primase subunit 1 | | | P49642 |  |
| PRIM2 | DNA primase subunit 2 | | | P49643 |  |
| PRIMPOL | Primase and DNA directed polymerase | | | Q96LW4 |  |
| PRKAA1 | Protein kinase AMP-activated catalytic subunit alpha 1 | | | Q13131 |  |
| PRKAB1 | Protein kinase AMP-activated non-catalytic subunit beta 1 | | | Q9Y478 |  |
| PRKACB | Protein kinase cAMP-activated catalytic subunit beta | | | P22694 |  |
| PRKAG1 | Protein kinase AMP-activated non-catalytic subunit gamma 1 | | | P54619 |  |
| PRKAG2 | Protein kinase AMP-activated non-catalytic subunit gamma 2 | | | Q9UGJ0 |  |
| PRKAR1A | Protein kinase cAMP-dependent type I regulatory subunit alpha | | | P10644 |  |
| PRKAR1B | Protein kinase cAMP-dependent type I regulatory subunit beta | | | P31321 |  |
| PRKAR2A | Protein kinase cAMP-dependent type II regulatory subunit alpha | | | P13861 |  |
| PRKCA | Protein kinase C alpha | | | P17252 |  |
| PRKCB | Protein kinase C beta | | | P05771 |  |
| PRKCD | Protein kinase C delta | | | Q05655 |  |
| PRKCH | Protein kinase C eta | | | P24723 |  |
| PRKCI | Protein kinase C iota | | | P41743 |  |
| PRKCQ | Protein kinase C theta | | | Q04759 |  |
| PRKD2 | Protein kinase D2 | | | Q9BZL6 |  |
| PRKD3 | Protein kinase D3 | | | O94806 |  |
| PRKRA | Protein activator of interferon induced protein kinase EIF2AK2 | | | O75569 |  |
| PRKRIP1 | PRKR interacting protein 1 | | | Q9H875 |  |
| PRKX | Protein kinase cAMP-dependent X-linked catalytic subunit | | | P51817 |  |
| PRMT1 | Protein arginine methyltransferase 1 | | | Q99873 |  |
| PRMT2 | Protein arginine methyltransferase 2 | | | P55345 |  |
| PRMT3 | Protein arginine methyltransferase 3 | | | O60678 |  |
| PRMT5 | Protein arginine methyltransferase 5 | | | O14744 |  |
| PRMT6 | Protein arginine methyltransferase 6 | | | Q96LA8 |  |
| PRMT7 | Protein arginine methyltransferase 7 | | | Q9NVM4 |  |
| PRNP | Prion protein | | | P04156 |  |
| PROCA1 | Protein interacting with cyclin A1 | | | Q8NCQ7 |  |
| PROCR | Protein C receptor | | | Q9UNN8 |  |
| PROK2 | Prokineticin 2 | | | Q9HC23 |  |
| PROSER3 | Proline and serine rich 3 | | | Q2NL68 |  |
| PRPF18 | Pre-mRNA processing factor 18 | | | Q99633 |  |
| PRPF19 | Pre-mRNA processing factor 19 | | | Q9UMS4 |  |
| PRPF3 | Pre-mRNA processing factor 3 | | | O43395 |  |
| PRPF31 | Pre-mRNA processing factor 31 | | | Q8WWY3 |  |
| PRPF38A | Pre-mRNA processing factor 38A | | | Q8NAV1 |  |
| PRPF4 | Pre-mRNA processing factor 4 | | | O43172 |  |
| PRPF6 | Pre-mRNA processing factor 6 | | | O94906 |  |
| PRPS1 | Phosphoribosyl pyrophosphate synthetase 1 | | | P60891 |  |
| PRPS2 | Phosphoribosyl pyrophosphate synthetase 2 | | | P11908 |  |
| PRPSAP1 | Phosphoribosyl pyrophosphate synthetase associated protein 1 | | | Q14558 |  |
| PRPSAP2 | Phosphoribosyl pyrophosphate synthetase associated protein 2 | | | O60256 |  |
| PRR13 | Proline rich 13 | | | Q9NZ81 |  |
| PRR14 | Proline rich 14 | | | Q9BWN1 |  |
| PRR5 | Proline rich 5 | | | P85299 |  |
| PRR7 | Proline rich 7, synaptic | | | Q8TB68 |  |
| PRRC1 | Proline rich coiled-coil 1 | | | Q96M27 |  |
| PRRC2B | Proline rich coiled-coil 2B | | | Q5JSZ5 |  |
| PRRT3 | Proline rich transmembrane protein 3 | | | Q5FWE3 |  |
| PRSS21 | Serine protease 21 | | | Q9Y6M0 |  |
| PSAP | Prosaposin | | | P07602 |  |
| PSAT1 | Phosphoserine aminotransferase 1 | | | Q9Y617 |  |
| PSD4 | Pleckstrin and Sec7 domain containing 4 | | | Q8NDX1 |  |
| PSEN1 | Presenilin 1 | | | P49768 |  |
| PSEN2 | Presenilin 2 | | | P49810 |  |
| PSENEN | Presenilin enhancer, gamma-secretase subunit | | | Q9NZ42 |  |
| PSIP1 | PC4 and SFRS1 interacting protein 1 | | | O75475 |  |
| PSKH1 | Protein serine kinase H1 | | | P11801 |  |
| PSMA1 | Proteasome 20S subunit alpha 1 | | | P25786 |  |
| PSMA2 | Proteasome 20S subunit alpha 2 | | | P25787 |  |
| PSMA3 | Proteasome 20S subunit alpha 3 | | | P25788 |  |
| PSMA4 | Proteasome 20S subunit alpha 4 | | | P25789 |  |
| PSMA5 | Proteasome 20S subunit alpha 5 | | | P28066 |  |
| PSMA6 | Proteasome 20S subunit alpha 6 | | | P60900 |  |
| PSMA7 | Proteasome 20S subunit alpha 7 | | | O14818 |  |
| PSMB1 | Proteasome 20S subunit beta 1 | | | P20618 |  |
| PSMB10 | Proteasome 20S subunit beta 10 | | | P40306 |  |
| PSMB2 | Proteasome 20S subunit beta 2 | | | P49721 |  |
| PSMB3 | Proteasome 20S subunit beta 3 | | | P49720 |  |
| PSMB4 | Proteasome 20S subunit beta 4 | | | P28070 |  |
| PSMB5 | Proteasome 20S subunit beta 5 | | | P28074 |  |
| PSMB6 | Proteasome 20S subunit beta 6 | | | P28072 |  |
| PSMB7 | Proteasome 20S subunit beta 7 | | | Q99436 |  |
| PSMB8 | Proteasome 20S subunit beta 8 | | | P28062 |  |
| PSMB9 | Proteasome 20S subunit beta 9 | | | P28065 |  |
| PSMC1 | Proteasome 26S subunit, ATPase 1 | | | P62191 |  |
| PSMC2 | Proteasome 26S subunit, ATPase 2 | | | P35998 |  |
| PSMC3 | Proteasome 26S subunit, ATPase 3 | | | P17980 |  |
| PSMC4 | Proteasome 26S subunit, ATPase 4 | | | P43686 |  |
| PSMC5 | Proteasome 26S subunit, ATPase 5 | | | P62195 |  |
| PSMC6 | Proteasome 26S subunit, ATPase 6 | | | P62333 |  |
| PSMD10 | Proteasome 26S subunit, non-ATPase 10 | | | O75832 |  |
| PSMD11 | Proteasome 26S subunit, non-ATPase 11 | | | O00231 |  |
| PSMD12 | Proteasome 26S subunit, non-ATPase 12 | | | O00232 |  |
| PSMD13 | Proteasome 26S subunit, non-ATPase 13 | | | Q9UNM6 |  |
| PSMD14 | Proteasome 26S subunit, non-ATPase 14 | | | O00487 |  |
| PSMD2 | Proteasome 26S subunit ubiquitin receptor, non-ATPase 2 | | | Q13200 |  |
| PSMD3 | Proteasome 26S subunit, non-ATPase 3 | | | O43242 |  |
| PSMD4 | Proteasome 26S subunit ubiquitin receptor, non-ATPase 4 | | | P55036 |  |
| PSMD5 | Proteasome 26S subunit, non-ATPase 5 | | | Q16401 |  |
| PSMD7 | Proteasome 26S subunit, non-ATPase 7 | | | P51665 |  |
| PSMD8 | Proteasome 26S subunit, non-ATPase 8 | | | P48556 |  |
| PSMD9 | Proteasome 26S subunit, non-ATPase 9 | | | O00233 |  |
| PSME1 | Proteasome activator subunit 1 | | | Q06323 |  |
| PSME2 | Proteasome activator subunit 2 | | | Q9UL46 |  |
| PSME3 | Proteasome activator subunit 3 | | | P61289 |  |
| PSMF1 | Proteasome inhibitor subunit 1 | | | Q92530 |  |
| PSMG1 | Proteasome assembly chaperone 1 | | | O95456 |  |
| PSMG2 | Proteasome assembly chaperone 2 | | | Q969U7 |  |
| PSMG3 | Proteasome assembly chaperone 3 | | | Q9BT73 |  |
| PSPC1 | Paraspeckle component 1 | | | Q8WXF1 |  |
| PSPH | Phosphoserine phosphatase | | | P78330 |  |
| PSTK | Phosphoseryl-tRNA kinase | | | Q8IV42 |  |
| PSTPIP1 | Proline-serine-threonine phosphatase interacting protein 1 | | | O43586 |  |
| PSTPIP2 | Proline-serine-threonine phosphatase interacting protein 2 | | | Q9H939 |  |
| PTBP1 | Polypyrimidine tract binding protein 1 | | | P26599 |  |
| PTBP2 | Polypyrimidine tract binding protein 2 | | | Q9UKA9 |  |
| PTBP3 | Polypyrimidine tract binding protein 3 | | | O95758 |  |
| PTCD1 | Pentatricopeptide repeat domain 1 | | | O75127 |  |
| PTCD2 | Pentatricopeptide repeat domain 2 | | | Q8WV60 |  |
| PTCD3 | Pentatricopeptide repeat domain 3 | | | Q96EY7 |  |
| PTCH1 | Patched 1 | | | Q13635 |  |
| PTDSS1 | Phosphatidylserine synthase 1 | | | P48651 |  |
| PTEN | Phosphatase and tensin homolog | | | P60484 |  |
| PTER | Phosphotriesterase related | | | Q96BW5 |  |
| PTGDR | Prostaglandin D2 receptor | | | Q13258 |  |
| PTGDS | Prostaglandin D2 synthase | | | P41222 |  |
| PTGER2 | Prostaglandin E receptor 2 | | | P43116 |  |
| PTGER4 | Prostaglandin E receptor 4 | | | P35408 |  |
| PTGES2 | Prostaglandin E synthase 2 | | | Q9H7Z7 |  |
| PTGES3 | Prostaglandin E synthase 3 | | | Q15185 |  |
| PTGIR | Prostaglandin I2 receptor | | | P43119 |  |
| PTGR2 | Prostaglandin reductase 2 | | | Q8N8N7 |  |
| PTK2 | Protein tyrosine kinase 2 | | | Q05397 |  |
| PTK2B | Protein tyrosine kinase 2 beta | | | Q14289 |  |
| PTMA | Prothymosin alpha | | | P06454 |  |
| PTOV1 | PTOV1 extended AT-hook containing adaptor protein | | | Q86YD1 |  |
| PTP4A1 | Protein tyrosine phosphatase 4A1 | | | Q93096 |  |
| PTP4A2 | Protein tyrosine phosphatase 4A2 | | | Q12974 |  |
| PTP4A3 | Protein tyrosine phosphatase 4A3 | | | O75365 |  |
| PTPMT1 | Protein tyrosine phosphatase mitochondrial 1 | | | Q8WUK0 |  |
| PTPN1 | Protein tyrosine phosphatase non-receptor type 1 | | | P18031 |  |
| PTPN11 | Protein tyrosine phosphatase non-receptor type 11 | | | Q06124 |  |
| PTPN12 | Protein tyrosine phosphatase non-receptor type 12 | | | Q05209 |  |
| PTPN18 | Protein tyrosine phosphatase non-receptor type 18 | | | Q99952 |  |
| PTPN2 | Protein tyrosine phosphatase non-receptor type 2 | | | P17706 |  |
| PTPN22 | Protein tyrosine phosphatase non-receptor type 22 | | | Q9Y2R2 |  |
| PTPN4 | Protein tyrosine phosphatase non-receptor type 4 | | | P29074 |  |
| PTPN4 | Protein tyrosine phosphatase non-receptor type 4 | | | P29074 |  |
| PTPN6 | Protein tyrosine phosphatase non-receptor type 6 | | | P29350 |  |
| PTPN7 | Protein tyrosine phosphatase non-receptor type 7 | | | P35236 |  |
| PTPN9 | Protein tyrosine phosphatase non-receptor type 9 | | | P43378 |  |
| PTPRA | Protein tyrosine phosphatase receptor type A | | | P18433 |  |
| PTPRC | Protein tyrosine phosphatase receptor type C | | | P08575 |  |
| PTPRCAP | Protein tyrosine phosphatase receptor type C associated protein | | | Q14761 |  |
| PTPRE | Protein tyrosine phosphatase receptor type E | | | P23469 |  |
| PTPRN2 | Protein tyrosine phosphatase receptor type N2 | | | Q92932 |  |
| PTPRO | Protein tyrosine phosphatase receptor type O | | | Q16827 |  |
| PTRH1 | Peptidyl-tRNA hydrolase 1 homolog | | | Q86Y79 |  |
| PTRH2 | Peptidyl-tRNA hydrolase 2 | | | Q9Y3E5 |  |
| PTRHD1 | Peptidyl-tRNA hydrolase domain containing 1 | | | Q6GMV3 |  |
| PTS | 6-pyruvoyltetrahydropterin synthase | | | Q03393 |  |
| PTTG1 | PTTG1 regulator of sister chromatid separation, securin | | | O95997 |  |
| PTTG1IP | PTTG1 interacting protein | | | P53801 |  |
| PUDP | Pseudouridine 5'-phosphatase | | | Q08623 |  |
| PUF60 | Poly(U) binding splicing factor 60 | | | Q9UHX1 |  |
| PUM1 | Pumilio RNA binding family member 1 | | | Q14671 |  |
| PUM2 | Pumilio RNA binding family member 2 | | | Q8TB72 |  |
| PUM3 | Pumilio RNA binding family member 3 | | | Q15397 |  |
| PURA | Purine rich element binding protein A | | | Q00577 |  |
| PUS1 | Pseudouridine synthase 1 | | | Q9Y606 |  |
| PUS3 | Pseudouridine synthase 3 | | | Q9BZE2 |  |
| PUS7L | Pseudouridine synthase 7 like | | | Q9H0K6 |  |
| PUSL1 | Pseudouridine synthase like 1 | | | Q8N0Z8 |  |
| PVRIG | PVR related immunoglobulin domain containing | | | Q6DKI7 |  |
| PWP1 | PWP1 homolog, endonuclein | | | Q13610 |  |
| PWP2 | PWP2 small subunit processome component | | | Q15269 |  |
| PXK | PX domain containing serine/threonine kinase like | | | Q7Z7A4 |  |
| PXMP4 | Peroxisomal membrane protein 4 | | | Q9Y6I8 |  |
| PXN | Paxillin | | | P49023 |  |
| PXYLP1 | 2-phosphoxylose phosphatase 1 | | | Q8TE99 |  |
| PYCARD | PYD and CARD domain containing | | | Q9ULZ3 |  |
| PYCR1 | Pyrroline-5-carboxylate reductase 1 | | | P32322 |  |
| PYCR2 | Pyrroline-5-carboxylate reductase 2 | | | Q96C36 |  |
| PYGB | Glycogen phosphorylase B | | | P11216 |  |
| PYGO2 | Pygopus family PHD finger 2 | | | Q9BRQ0 |  |
| PYHIN1 | Pyrin and HIN domain family member 1 | | | Q6K0P9 |  |
| PYHIN1 | Pyrin and HIN domain family member 1 | | | Q6K0P9 |  |
| PYM1 | PYM homolog 1, exon junction complex associated factor | | | Q9BRP8 |  |
| PYROXD1 | Pyridine nucleotide-disulphide oxidoreductase domain 1 | | | Q8WU10 |  |
| PYROXD2 | Pyridine nucleotide-disulphide oxidoreductase domain 2 | | | Q8N2H3 |  |
| PYURF | PIGY upstream open reading frame | | | Q96I23 |  |
| QDPR | Quinoid dihydropteridine reductase | | | P09417 |  |
| QKI | QKI, KH domain containing RNA binding | | | Q96PU8 |  |
| QRICH1 | Glutamine rich 1 | | | Q2TAL8 |  |
| QRSL1 | Glutaminyl-tRNA amidotransferase subunit QRSL1 | | | Q9H0R6 |  |
| QSOX1 | Quiescin sulfhydryl oxidase 1 | | | O00391 |  |
| QTRT1 | Queuine tRNA-ribosyltransferase catalytic subunit 1 | | | Q9BXR0 |  |
| R3HCC1L | R3H domain and coiled-coil containing 1 like | | | Q7Z5L2 |  |
| R3HDM1 | R3H domain containing 1 | | | Q15032 |  |
| R3HDM4 | R3H domain containing 4 | | | Q96D70 |  |
| RAB10 | RAB10, member RAS oncogene family | | | P61026 |  |
| RAB11A | RAB11A, member RAS oncogene family | | | P62491 |  |
| RAB11B | RAB11B, member RAS oncogene family | | | Q15907 |  |
| RAB11FIP1 | RAB11 family interacting protein 1 | | | Q6WKZ4 |  |
| RAB11FIP4 | RAB11 family interacting protein 4 | | | Q86YS3 |  |
| RAB12 | RAB12, member RAS oncogene family | | |  |  |
| RAB14 | RAB14, member RAS oncogene family | | | P61106 |  |
| RAB18 | RAB18, member RAS oncogene family | | | Q9NP72 |  |
| RAB1A | RAB1A, member RAS oncogene family | | | P62820 |  |
| RAB1B | RAB1B, member RAS oncogene family | | | Q9H0U4 |  |
| RAB20 | RAB20, member RAS oncogene family | | | Q9NX57 |  |
| RAB22A | RAB22A, member RAS oncogene family | | | Q9UL26 |  |
| RAB24 | RAB24, member RAS oncogene family | | | Q969Q5 |  |
| RAB25 | RAB25, member RAS oncogene family | | | P57735 |  |
| RAB25 | RAB25, member RAS oncogene family | | | P57735 |  |
| RAB27A | RAB27A, member RAS oncogene family | | | P51159 |  |
| RAB28 | RAB28, member RAS oncogene family | | | P51157 |  |
| RAB29 | RAB29, member RAS oncogene family | | | O14966 |  |
| RAB2A | RAB2A, member RAS oncogene family | | | P61019 |  |
| RAB2B | RAB2B, member RAS oncogene family | | | Q8WUD1 |  |
| RAB30 | RAB30, member RAS oncogene family | | | Q15771 |  |
| RAB33A | RAB33A, member RAS oncogene family | | | Q14088 |  |
| RAB33B | RAB33B, member RAS oncogene family | | | Q9H082 |  |
| RAB34 | RAB34, member RAS oncogene family | | | P0DI83, Q9BZG1 |  |
| RAB35 | RAB35, member RAS oncogene family | | | Q15286 |  |
| RAB37 | RAB37, member RAS oncogene family | | | Q96AX2 |  |
| RAB39B | RAB39B, member RAS oncogene family | | | Q96DA2 |  |
| RAB3A | RAB3A, member RAS oncogene family | | | P20336 |  |
| RAB3D | RAB3D, member RAS oncogene family | | | O95716 |  |
| RAB3IP | RAB3A interacting protein | | | Q96QF0 |  |
| RAB40B | RAB40B, member RAS oncogene family | | | Q12829 |  |
| RAB40C | RAB40C, member RAS oncogene family | | | Q96S21 |  |
| RAB43 | RAB43, member RAS oncogene family | | | Q86YS6 |  |
| RAB4A | RAB4A, member RAS oncogene family | | | P20338 |  |
| RAB4B | RAB4B, member RAS oncogene family | | | P61018 |  |
| RAB5A | RAB5A, member RAS oncogene family | | | P20339 |  |
| RAB5B | RAB5B, member RAS oncogene family | | | P61020 |  |
| RAB5C | RAB5C, member RAS oncogene family | | | P51148 |  |
| RAB7A | RAB7A, member RAS oncogene family | | | P51149 |  |
| RAB8A | RAB8A, member RAS oncogene family | | | P61006 |  |
| RAB8B | RAB8B, member RAS oncogene family | | | Q92930 |  |
| RAB9A | RAB9A, member RAS oncogene family | | | P51151 |  |
| RABAC1 | Rab acceptor 1 | | | Q9UI14 |  |
| RABEP1 | Rabaptin, RAB GTPase binding effector protein 1 | | | Q15276 |  |
| RABEP2 | Rabaptin, RAB GTPase binding effector protein 2 | | | Q9H5N1 |  |
| RABEPK | Rab9 effector protein with kelch motifs | | | Q7Z6M1 |  |
| RABGAP1 | RAB GTPase activating protein 1 | | | Q9Y3P9 |  |
| RABGAP1L | RAB GTPase activating protein 1 like | | | B7ZAP0, Q5R372 |  |
| RABGEF1 | RAB guanine nucleotide exchange factor 1 | | | Q9UJ41 |  |
| RABGGTA | Rab geranylgeranyltransferase subunit alpha | | | Q92696 |  |
| RABGGTB | Rab geranylgeranyltransferase subunit beta | | | P53611 |  |
| RABIF | RAB interacting factor | | | P47224 |  |
| RABL2A | RAB, member of RAS oncogene family like 2A | | | Q9UBK7 |  |
| RABL2B | RAB, member of RAS oncogene family like 2B | | | Q9UNT1 |  |
| RABL3 | RAB, member of RAS oncogene family like 3 | | | Q5HYI8 |  |
| RABL6 | RAB, member RAS oncogene family like 6 | | | Q3YEC7 |  |
| RAC1 | Rac family small GTPase 1 | | | P63000 |  |
| RAC2 | Rac family small GTPase 2 | | | P15153 |  |
| RACGAP1 | Rac GTPase activating protein 1 | | | Q9H0H5 |  |
| RACK1 | Receptor for activated C kinase 1 | | | P63244 |  |
| RAD1 | RAD1 checkpoint DNA exonuclease | | | O60671 |  |
| RAD17 | RAD17 checkpoint clamp loader component | | | O75943 |  |
| RAD18 | RAD18 E3 ubiquitin protein ligase | | | Q9NS91 |  |
| RAD21 | RAD21 cohesin complex component | | | O60216 |  |
| RAD23A | RAD23 homolog A, nucleotide excision repair protein | | | P54725 |  |
| RAD23B | RAD23 homolog B, nucleotide excision repair protein | | | P54727 |  |
| RAD51 | RAD51 recombinase | | | Q06609 |  |
| RAD51B | RAD51 paralog B | | | O15315 |  |
| RAD51C | RAD51 paralog C | | | O43502 |  |
| RAD51D | RAD51 paralog D | | | O75771 |  |
| RAD52 | RAD52 homolog, DNA repair protein | | | P43351 |  |
| RAD54L2 | RAD54 like 2 | | | Q9Y4B4 |  |
| RAE1 | Ribonucleic acid export 1 | | | P78406 |  |
| RAF1 | Raf-1 proto-oncogene, serine/threonine kinase | | | P04049 |  |
| RALA | RAS like proto-oncogene A | | | P11233 |  |
| RALB | RAS like proto-oncogene B | | | P11234 |  |
| RALBP1 | RalA binding protein 1 | | | Q15311 |  |
| RALGAPB | Ral GTPase activating protein non-catalytic subunit beta | | | Q86X10 |  |
| RALGDS | Ral guanine nucleotide dissociation stimulator | | | Q12967 |  |
| RALGDS | Ral guanine nucleotide dissociation stimulator | | | Q12967 |  |
| RALY | RALY heterogeneous nuclear ribonucleoprotein | | | Q9UKM9 |  |
| RAN | RAN, member RAS oncogene family | | | P62826 |  |
| RANBP1 | RAN binding protein 1 | | | P43487 |  |
| RANBP3 | RAN binding protein 3 | | | Q9H6Z4 |  |
| RANBP6 | RAN binding protein 6 | | | O60518 |  |
| RANGAP1 | Ran GTPase activating protein 1 | | | P46060 |  |
| RANGRF | RAN guanine nucleotide release factor | | | Q9HD47 |  |
| RAP1A | RAP1A, member of RAS oncogene family | | | P62834 |  |
| RAP1B | RAP1B, member of RAS oncogene family | | | P61224 |  |
| RAP1GDS1 | Rap1 GTPase-GDP dissociation stimulator 1 | | | P52306 |  |
| RAP2B | RAP2B, member of RAS oncogene family | | | P61225 |  |
| RAP2C | RAP2C, member of RAS oncogene family | | | Q9Y3L5 |  |
| RAPGEF1 | Rap guanine nucleotide exchange factor 1 | | | Q13905 |  |
| RAPGEF6 | Rap guanine nucleotide exchange factor 6 | | | Q8TEU7 |  |
| RARA | Retinoic acid receptor alpha | | | P10276 |  |
| RARG | Retinoic acid receptor gamma | | | P13631 |  |
| RARS2 | Arginyl-tRNA synthetase 2, mitochondrial | | | Q5T160 |  |
| RASA1 | RAS p21 protein activator 1 | | | P20936 |  |
| RASA2 | RAS p21 protein activator 2 | | | Q15283 |  |
| RASA3 | RAS p21 protein activator 3 | | | Q14644 |  |
| RASA4 | RAS p21 protein activator 4 | | | O43374 |  |
| RASAL3 | RAS protein activator like 3 | | | Q86YV0 |  |
| RASEF | RAS and EF-hand domain containing | | | Q8IZ41 |  |
| RASGEF1B | RasGEF domain family member 1B | | | Q0VAM2 |  |
| RASGRP1 | RAS guanyl releasing protein 1 | | | O95267 |  |
| RASGRP2 | RAS guanyl releasing protein 2 | | | Q7LDG7 |  |
| RASSF1 | Ras association domain family member 1 | | | Q9NS23 |  |
| RASSF2 | Ras association domain family member 2 | | | P50749 |  |
| RASSF3 | Ras association domain family member 3 | | | Q86WH2 |  |
| RASSF5 | Ras association domain family member 5 | | | Q8WWW0 |  |
| RASSF7 | Ras association domain family member 7 | | | Q02833 |  |
| RB1 | RB transcriptional corepressor 1 | | | P06400 |  |
| RB1CC1 | RB1 inducible coiled-coil 1 | | | Q8TDY2 |  |
| RBBP4 | RB binding protein 4, chromatin remodeling factor | | | Q09028 |  |
| RBBP5 | RB binding protein 5, histone lysine methyltransferase complex subunit | | | Q15291 |  |
| RBBP6 | RB binding protein 6, ubiquitin ligase | | | Q7Z6E9 |  |
| RBBP7 | RB binding protein 7, chromatin remodeling factor | | | Q16576 |  |
| RBBP8 | RB binding protein 8, endonuclease | | | Q99708 |  |
| RBBP9 | RB binding protein 9, serine hydrolase | | | O75884 |  |
| RBCK1 | RANBP2-type and C3HC4-type zinc finger containing 1 | | | Q9BYM8 |  |
| RBFA | Ribosome binding factor A | | | Q8N0V3 |  |
| RBKS | Ribokinase | | | Q9H477 |  |
| RBL1 | RB transcriptional corepressor like 1 | | | P28749 |  |
| RBL2 | RB transcriptional corepressor like 2 | | | Q08999 |  |
| RBM10 | RNA binding motif protein 10 | | | P98175 |  |
| RBM11 | RNA binding motif protein 11 | | | P57052 |  |
| RBM12 | RNA binding motif protein 12 | | | Q9NTZ6 |  |
| RBM14 | RNA binding motif protein 14 | | | Q96PK6 |  |
| RBM15 | RNA binding motif protein 15 | | | Q96T37 |  |
| RBM15B | RNA binding motif protein 15B | | | Q8NDT2 |  |
| RBM17 | RNA binding motif protein 17 | | | Q96I25 |  |
| RBM18 | RNA binding motif protein 18 | | | Q96H35 |  |
| RBM19 | RNA binding motif protein 19 | | | Q9Y4C8 |  |
| RBM22 | RNA binding motif protein 22 | | | Q9NW64 |  |
| RBM23 | RNA binding motif protein 23 | | | Q86U06 |  |
| RBM25 | RNA binding motif protein 25 | | | P49756 |  |
| RBM28 | RNA binding motif protein 28 | | | Q9NW13 |  |
| RBM3 | RNA binding motif protein 3 | | | P98179 |  |
| RBM33 | RNA binding motif protein 33 | | | Q96EV2 |  |
| RBM34 | RNA binding motif protein 34 | | | P42696 |  |
| RBM38 | RNA binding motif protein 38 | | | Q9H0Z9 |  |
| RBM39 | RNA binding motif protein 39 | | | Q14498 |  |
| RBM4 | RNA binding motif protein 4 | | | Q9BWF3 |  |
| RBM41 | RNA binding motif protein 41 | | | Q96IZ5 |  |
| RBM42 | RNA binding motif protein 42 | | | Q9BTD8 |  |
| RBM43 | RNA binding motif protein 43 | | | Q6ZSC3 |  |
| RBM45 | RNA binding motif protein 45 | | | Q8IUH3 |  |
| RBM48 | RNA binding motif protein 48 | | | Q5RL73 |  |
| RBM4B | RNA binding motif protein 4B | | | Q9BQ04 |  |
| RBM5 | RNA binding motif protein 5 | | | P52756 |  |
| RBM6 | RNA binding motif protein 6 | | | P78332 |  |
| RBM7 | RNA binding motif protein 7 | | | Q9Y580 |  |
| RBM8A | RNA binding motif protein 8A | | | Q9Y5S9 |  |
| RBMS1 | RNA binding motif single stranded interacting protein 1 | | | P29558 |  |
| RBMX | RNA binding motif protein X-linked | | | P38159 |  |
| RBMX2 | RNA binding motif protein X-linked 2 | | | Q9Y388 |  |
| RBPJ | Recombination signal binding protein for immunoglobulin kappa J region | | | Q06330 |  |
| RBSN | Rabenosyn, RAB effector | | | Q9H1K0 |  |
| RBX1 | Ring-box 1 | | | P62877 |  |
| RC3H2 | Ring finger and CCCH-type domains 2 | | | Q9HBD1 |  |
| RCAN2 | Regulator of calcineurin 2 | | | Q14206 |  |
| RCAN3 | RCAN family member 3 | | | Q9UKA8 |  |
| RCBTB1 | RCC1 and BTB domain containing protein 1 | | | Q8NDN9 |  |
| RCBTB2 | RCC1 and BTB domain containing protein 2 | | | O95199 |  |
| RCC1 | Regulator of chromosome condensation 1 | | | P18754 |  |
| RCE1 | Ras converting CAAX endopeptidase 1 | | | Q9Y256 |  |
| RCHY1 | Ring finger and CHY zinc finger domain containing 1 | | | Q96PM5 |  |
| RCL1 | RNA terminal phosphate cyclase like 1 | | | Q9Y2P8 |  |
| RCN1 | Reticulocalbin 1 | | | Q15293 |  |
| RCN2 | Reticulocalbin 2 | | | Q14257 |  |
| RCN3 | Reticulocalbin 3 | | | Q96D15 |  |
| RCN3 | Reticulocalbin 3 | | | Q96D15 |  |
| RCOR3 | REST corepressor 3 | | | Q9P2K3 |  |
| RCSD1 | RCSD domain containing 1 | | | Q6JBY9 |  |
| RDH11 | Retinol dehydrogenase 11 | | | Q8TC12 |  |
| RDH13 | Retinol dehydrogenase 13 | | | Q8NBN7 |  |
| RDH14 | Retinol dehydrogenase 14 | | | Q9HBH5 |  |
| RDX | Radixin | | | P35241 |  |
| REC8 | REC8 meiotic recombination protein | | | O95072 |  |
| RECK | Reversion inducing cysteine rich protein with kazal motifs | | | O95980 |  |
| RECQL | RecQ like helicase | | | P46063 |  |
| RECQL5 | RecQ like helicase 5 | | | O94762 |  |
| REEP3 | Receptor accessory protein 3 | | | Q6NUK4 |  |
| REEP4 | Receptor accessory protein 4 | | | Q9H6H4 |  |
| REEP5 | Receptor accessory protein 5 | | | Q00765 |  |
| REEP6 | Receptor accessory protein 6 | | | Q96HR9 |  |
| REL | REL proto-oncogene, NF-kB subunit | | | Q04864 |  |
| RELA | RELA proto-oncogene, NF-kB subunit | | | Q04206 |  |
| RELB | RELB proto-oncogene, NF-kB subunit | | | Q01201 |  |
| RELL1 | RELT like 1 | | | Q8IUW5 |  |
| RELL2 | RELT like 2 | | | Q8NC24 |  |
| RELT | RELT TNF receptor | | | Q969Z4 |  |
| REPIN1 | Replication initiator 1 | | | Q9BWE0 |  |
| REPS1 | RALBP1 associated Eps domain containing 1 | | | Q96D71 |  |
| RER1 | Retention in endoplasmic reticulum sorting receptor 1 | | | O15258 |  |
| RERE | Arginine-glutamic acid dipeptide repeats | | | Q9P2R6 |  |
| REST | RE1 silencing transcription factor | | | Q13127 |  |
| RETN | Resistin | | | Q9HD89 |  |
| RETSAT | Retinol saturase | | | Q6NUM9 |  |
| REXO2 | RNA exonuclease 2 | | | Q9Y3B8 |  |
| REXO4 | REX4 homolog, 3'-5' exonuclease | | | Q9GZR2 |  |
| RFC1 | Replication factor C subunit 1 | | | P35251 |  |
| RFC2 | Replication factor C subunit 2 | | | P35250 |  |
| RFC3 | Replication factor C subunit 3 | | | P40938 |  |
| RFC4 | Replication factor C subunit 4 | | | P35249 |  |
| RFC5 | Replication factor C subunit 5 | | | P40937 |  |
| RFESD | Rieske Fe-S domain containing | | | Q8TAC1 |  |
| RFFL | Ring finger and FYVE like domain containing E3 ubiquitin protein ligase | | | Q8WZ73 |  |
| RFK | Riboflavin kinase | | | Q969G6 |  |
| RFT1 | RFT1 homolog | | | Q96AA3 |  |
| RFTN1 | Raftlin, lipid raft linker 1 | | | Q14699 |  |
| RFWD3 | Ring finger and WD repeat domain 3 | | | Q6PCD5 |  |
| RFX3 | Regulatory factor X3 | | | P48380 |  |
| RFX5 | Regulatory factor X5 | | | P48382 |  |
| RFXANK | Regulatory factor X associated ankyrin containing protein | | | O14593 |  |
| RGCC | Regulator of cell cycle | | | Q9H4X1 |  |
| RGCC | Regulator of cell cycle | | | Q9H4X1 |  |
| RGL4 | Ral guanine nucleotide dissociation stimulator like 4 | | | Q8IZJ4 |  |
| RGMB | Repulsive guidance molecule BMP co-receptor b | | | Q6NW40 |  |
| RGMB | Repulsive guidance molecule BMP co-receptor b | | | Q6NW40 |  |
| RGPD2 | RANBP2 like and GRIP domain containing 2 | | | P0DJD1 |  |
| RGPD5 | RANBP2 like and GRIP domain containing 5 | | | Q99666 |  |
| RGS1 | Regulator of G protein signaling 1 | | | Q08116 |  |
| RGS10 | Regulator of G protein signaling 10 | | | O43665 |  |
| RGS14 | Regulator of G protein signaling 14 | | | O43566 |  |
| RGS16 | Regulator of G protein signaling 16 | | | O15492 |  |
| RGS19 | Regulator of G protein signaling 19 | | | P49795 |  |
| RGS2 | Regulator of G protein signaling 2 | | | P41220 |  |
| RGS3 | Regulator of G protein signaling 3 | | | P49796 |  |
| RHBDD1 | Rhomboid domain containing 1 | | | Q8TEB9 |  |
| RHBDD2 | Rhomboid domain containing 2 | | | Q6NTF9 |  |
| RHBDD3 | Rhomboid domain containing 3 | | | Q9Y3P4 |  |
| RHBDF2 | Rhomboid 5 homolog 2 | | | Q6PJF5 |  |
| RHEB | Ras homolog, mTORC1 binding | | | Q15382 |  |
| RHEBL1 | RHEB like 1 | | | Q8TAI7 |  |
| RHNO1 | RAD9-HUS1-RAD1 interacting nuclear orphan 1 | | | Q9BSD3 |  |
| RHOA | Ras homolog family member A | | | P61586 |  |
| RHOBTB3 | Rho related BTB domain containing 3 | | | O94955 |  |
| RHOC | Ras homolog family member C | | | P08134 |  |
| RHOG | Ras homolog family member G | | | P84095 |  |
| RHOH | Ras homolog family member H | | | Q15669 |  |
| RHOT1 | Ras homolog family member T1 | | | Q8IXI2 |  |
| RHOT2 | Ras homolog family member T2 | | | Q8IXI1 |  |
| RIC8A | RIC8 guanine nucleotide exchange factor A | | | Q9NPQ8 |  |
| RIC8B | RIC8 guanine nucleotide exchange factor B | | | Q9NVN3 |  |
| RICTOR | RPTOR independent companion of MTOR complex 2 | | | Q6R327 |  |
| RILPL2 | Rab interacting lysosomal protein like 2 | | | Q969X0 |  |
| RIMKLB | Ribosomal modification protein rimK like family member B | | | Q9ULI2 |  |
| RINL | Ras and Rab interactor like | | | Q6ZS11 |  |
| RINT1 | RAD50 interactor 1 | | | Q6NUQ1 |  |
| RIOK1 | RIO kinase 1 | | | Q9BRS2 |  |
| RIOK2 | RIO kinase 2 | | | Q9BVS4 |  |
| RIOK3 | RIO kinase 3 | | | O14730 |  |
| RIPK2 | Receptor interacting serine/threonine kinase 2 | | | O43353 |  |
| RIPK3 | Receptor interacting serine/threonine kinase 3 | | | Q9Y572 |  |
| RIT1 | Ras like without CAAX 1 | | | Q92963 |  |
| RITA1 | RBPJ interacting and tubulin associated 1 | | | Q96K30 |  |
| RLF | RLF zinc finger | | | Q13129 |  |
| RLIM | Ring finger protein, LIM domain interacting | | | Q9NVW2 |  |
| RLN2 | Relaxin 2 | | | P04090 |  |
| RMDN3 | Regulator of microtubule dynamics 3 | | | Q96TC7 |  |
| RMI1 | RecQ mediated genome instability 1 | | | Q9H9A7 |  |
| RMND1 | Required for meiotic nuclear division 1 homolog | | | Q9NWS8 |  |
| RMND5A | Required for meiotic nuclear division 5 homolog A | | | Q9H871 |  |
| RMND5B | Required for meiotic nuclear division 5 homolog B | | | Q96G75 |  |
| RNASE4 | Ribonuclease A family member 4 | | | P34096 |  |
| RNASE6 | Ribonuclease A family member k6 | | | Q93091 |  |
| RNASEH1 | Ribonuclease H1 | | | O60930 |  |
| RNASEH2A | Ribonuclease H2 subunit A | | | O75792 |  |
| RNASEH2B | Ribonuclease H2 subunit B | | | Q5TBB1 |  |
| RNASEH2C | Ribonuclease H2 subunit C | | | Q8TDP1 |  |
| RNASEK | Ribonuclease K | | | Q6P5S7 |  |
| RNASEL | Ribonuclease L | | | Q05823 |  |
| RNASET2 | Ribonuclease T2 | | | O00584 |  |
| RNF10 | Ring finger protein 10 | | | Q8N5U6 |  |
| RNF103 | Ring finger protein 103 | | | O00237 |  |
| RNF11 | Ring finger protein 11 | | | Q9Y3C5 |  |
| RNF111 | Ring finger protein 111 | | | Q6ZNA4 |  |
| RNF113A | Ring finger protein 113A | | | O15541 |  |
| RNF114 | Ring finger protein 114 | | | Q9Y508 |  |
| RNF115 | Ring finger protein 115 | | | Q9Y4L5 |  |
| RNF121 | Ring finger protein 121 | | | Q9H920 |  |
| RNF122 | Ring finger protein 122 | | | Q9H9V4 |  |
| RNF123 | Ring finger protein 123 | | | Q5XPI4 |  |
| RNF125 | Ring finger protein 125 | | | Q96EQ8 |  |
| RNF126 | Ring finger protein 126 | | | Q9BV68 |  |
| RNF13 | Ring finger protein 13 | | | O43567 |  |
| RNF135 | Ring finger protein 135 | | | Q8IUD6 |  |
| RNF138 | Ring finger protein 138 | | | Q8WVD3 |  |
| RNF139 | Ring finger protein 139 | | | Q8WU17 |  |
| RNF14 | Ring finger protein 14 | | | Q9UBS8 |  |
| RNF141 | Ring finger protein 141 | | | Q8WVD5 |  |
| RNF144A | Ring finger protein 144A | | | P50876 |  |
| RNF144B | Ring finger protein 144B | | | Q7Z419 |  |
| RNF149 | Ring finger protein 149 | | | Q8NC42 |  |
| RNF157 | Ring finger protein 157 | | | Q96PX1 |  |
| RNF166 | Ring finger protein 166 | | | Q96A37 |  |
| RNF167 | Ring finger protein 167 | | | Q9H6Y7 |  |
| RNF168 | Ring finger protein 168 | | | Q8IYW5 |  |
| RNF170 | Ring finger protein 170 | | | Q96K19 |  |
| RNF175 | Ring finger protein 175 | | | Q8N4F7 |  |
| RNF180 | Ring finger protein 180 | | | Q86T96 |  |
| RNF181 | Ring finger protein 181 | | | Q9P0P0 |  |
| RNF185 | Ring finger protein 185 | | | Q96GF1 |  |
| RNF19A | Ring finger protein 19A, RBR E3 ubiquitin protein ligase | | | Q9NV58 |  |
| RNF2 | Ring finger protein 2 | | | Q99496 |  |
| RNF20 | Ring finger protein 20 | | | Q5VTR2 |  |
| RNF212 | Ring finger protein 212 | | | Q495C1 |  |
| RNF213 | Ring finger protein 213 | | | Q63HN8 |  |
| RNF215 | Ring finger protein 215 | | | Q9Y6U7 |  |
| RNF216 | Ring finger protein 216 | | | Q9NWF9 |  |
| RNF24 | Ring finger protein 24 | | | Q9Y225 |  |
| RNF25 | Ring finger protein 25 | | | Q96BH1 |  |
| RNF26 | Ring finger protein 26 | | | Q9BY78 |  |
| RNF32 | Ring finger protein 32 | | | Q9H0A6 |  |
| RNF34 | Ring finger protein 34 | | | Q969K3 |  |
| RNF38 | Ring finger protein 38 | | | Q9H0F5 |  |
| RNF4 | Ring finger protein 4 | | | P78317 |  |
| RNF40 | Ring finger protein 40 | | | O75150 |  |
| RNF41 | Ring finger protein 41 | | | Q9H4P4 |  |
| RNF5 | Ring finger protein 5 | | | Q99942 |  |
| RNF6 | Ring finger protein 6 | | | Q9Y252 |  |
| RNF7 | Ring finger protein 7 | | | Q9UBF6 |  |
| RNF8 | Ring finger protein 8 | | | O76064 |  |
| RNFT1 | Ring finger protein, transmembrane 1 | | | Q5M7Z0 |  |
| RNGTT | RNA guanylyltransferase and 5'-phosphatase | | | O60942 |  |
| RNH1 | Ribonuclease/angiogenin inhibitor 1 | | | P13489 |  |
| RNLS | Renalase, FAD dependent amine oxidase | | | Q5VYX0 |  |
| RNMT | RNA guanine-7 methyltransferase | | | O43148 |  |
| RNPC3 | RNA binding region (RNP1, RRM) containing 3 | | | Q96LT9 |  |
| RNPEP | Arginyl aminopeptidase | | | Q9H4A4 |  |
| RNPEPL1 | Arginyl aminopeptidase like 1 | | | Q9HAU8 |  |
| RNPS1 | RNA binding protein with serine rich domain 1 | | | Q15287 |  |
| ROM1 | Retinal outer segment membrane protein 1 | | | Q03395 |  |
| ROMO1 | Reactive oxygen species modulator 1 | | | P60602 |  |
| ROPN1L | Rhophilin associated tail protein 1 like | | | Q96C74 |  |
| RORA | RAR related orphan receptor A | | | P35398 |  |
| RP2 | RP2 activator of ARL3 GTPase | | | O75695 |  |
| RP9 | RP9 pre-mRNA splicing factor | | | Q8TA86 |  |
| RPA1 | Replication protein A1 | | | P27694 |  |
| RPA2 | Replication protein A2 | | | P15927 |  |
| RPA3 | Replication protein A3 | | | P35244 |  |
| RPAIN | RPA interacting protein | | | Q86UA6 |  |
| RPAP1 | RNA polymerase II associated protein 1 | | | Q9BWH6 |  |
| RPAP3 | RNA polymerase II associated protein 3 | | | Q9H6T3 |  |
| RPE | Ribulose-5-phosphate-3-epimerase | | | Q96AT9 |  |
| RPF1 | Ribosome production factor 1 homolog | | | Q9H9Y2 |  |
| RPF2 | Ribosome production factor 2 homolog | | | Q9H7B2 |  |
| RPL10 | Ribosomal protein L10 | | | P27635 |  |
| RPL10A | Ribosomal protein L10a | | | P62906 |  |
| RPL11 | Ribosomal protein L11 | | | P62913 |  |
| RPL12 | Ribosomal protein L12 | | | P30050 |  |
| RPL13 | Ribosomal protein L13 | | | P26373 |  |
| RPL13A | Ribosomal protein L13a | | | P40429 |  |
| RPL14 | Ribosomal protein L14 | | | P50914 |  |
| RPL15 | Ribosomal protein L15 | | | P61313 |  |
| RPL17 | Ribosomal protein L17 | | | P18621 |  |
| RPL18 | Ribosomal protein L18 | | | Q07020 |  |
| RPL18A | Ribosomal protein L18a | | | Q02543 |  |
| RPL19 | Ribosomal protein L19 | | | P84098 |  |
| RPL21 | Ribosomal protein L21 | | | P46778 |  |
| RPL22 | Ribosomal protein L22 | | | P35268 |  |
| RPL22L1 | Ribosomal protein L22 like 1 | | | Q6P5R6 |  |
| RPL23 | Ribosomal protein L23 | | | P62829 |  |
| RPL23A | Ribosomal protein L23a | | | P62750 |  |
| RPL24 | Ribosomal protein L24 | | | P83731 |  |
| RPL26 | Ribosomal protein L26 | | | P61254 |  |
| RPL26L1 | Ribosomal protein L26 like 1 | | | Q9UNX3 |  |
| RPL27 | Ribosomal protein L27 | | | P61353 |  |
| RPL27A | Ribosomal protein L27a | | | P46776 |  |
| RPL28 | Ribosomal protein L28 | | | P46779 |  |
| RPL29 | Ribosomal protein L29 | | | P47914 |  |
| RPL3 | Ribosomal protein L3 | | | P39023 |  |
| RPL30 | Ribosomal protein L30 | | | P62888 |  |
| RPL31 | Ribosomal protein L31 | | | P62899 |  |
| RPL32 | Ribosomal protein L32 | | | P62910 |  |
| RPL34 | Ribosomal protein L34 | | | P49207 |  |
| RPL35 | Ribosomal protein L35 | | | P42766 |  |
| RPL35A | Ribosomal protein L35a | | | P18077 |  |
| RPL36 | Ribosomal protein L36 | | | Q9Y3U8 |  |
| RPL36A | Ribosomal protein L36a | | | P83881 |  |
| RPL36AL | Ribosomal protein L36a like | | | Q969Q0 |  |
| RPL37 | Ribosomal protein L37 | | | P61927 |  |
| RPL37A | Ribosomal protein L37a | | | P61513 |  |
| RPL38 | Ribosomal protein L38 | | | P63173 |  |
| RPL39 | Ribosomal protein L39 | | | P62891 |  |
| RPL39L | Ribosomal protein L39 like | | | Q96EH5 |  |
| RPL4 | Ribosomal protein L4 | | | P36578 |  |
| RPL6 | Ribosomal protein L6 | | | Q02878 |  |
| RPL7 | Ribosomal protein L7 | | | P18124 |  |
| RPL7A | Ribosomal protein L7a | | | P62424 |  |
| RPL7L1 | Ribosomal protein L7 like 1 | | | Q6DKI1 |  |
| RPL8 | Ribosomal protein L8 | | | P62917 |  |
| RPL9 | Ribosomal protein L9 | | | P32969 |  |
| RPLP0 | Ribosomal protein lateral stalk subunit P0 | | | P05388 |  |
| RPLP1 | Ribosomal protein lateral stalk subunit P1 | | | P05386 |  |
| RPLP2 | Ribosomal protein lateral stalk subunit P2 | | | P05387 |  |
| RPN2 | Ribophorin II | | | P04844 |  |
| RPP14 | Ribonuclease P/MRP subunit p14 | | | O95059 |  |
| RPP21 | Ribonuclease P/MRP subunit p21 | | | Q9H633 |  |
| RPP25 | Ribonuclease P and MRP subunit p25 | | | Q9BUL9 |  |
| RPP25L | Ribonuclease P/MRP subunit p25 like | | | Q8N5L8 |  |
| RPP30 | Ribonuclease P/MRP subunit p30 | | | P78346 |  |
| RPP38 | Ribonuclease P/MRP subunit p38 | | | P78345 |  |
| RPP40 | Ribonuclease P/MRP subunit p40 | | | O75818 |  |
| RPRD1A | Regulation of nuclear pre-mRNA domain containing 1A | | | Q96P16 |  |
| RPRD1B | Regulation of nuclear pre-mRNA domain containing 1B | | | Q9NQG5 |  |
| RPRD2 | Regulation of nuclear pre-mRNA domain containing 2 | | | Q5VT52 |  |
| RPS10 | Ribosomal protein S10 | | | P46783 |  |
| RPS11 | Ribosomal protein S11 | | | P62280 |  |
| RPS12 | Ribosomal protein S12 | | | P25398 |  |
| RPS13 | Ribosomal protein S13 | | | P62277 |  |
| RPS14 | Ribosomal protein S14 | | | P62263 |  |
| RPS15 | Ribosomal protein S15 | | | P62841 |  |
| RPS15A | Ribosomal protein S15a | | | P62244 |  |
| RPS16 | Ribosomal protein S16 | | | P62249 |  |
| RPS17 | Ribosomal protein S17 | | | P08708 |  |
| RPS18 | Ribosomal protein S18 | | | P62269 |  |
| RPS19 | Ribosomal protein S19 | | | P39019 |  |
| RPS19BP1 | Ribosomal protein S19 binding protein 1 | | | Q86WX3 |  |
| RPS2 | Ribosomal protein S2 | | | P15880 |  |
| RPS20 | Ribosomal protein S20 | | | P60866 |  |
| RPS21 | Ribosomal protein S21 | | | P63220 |  |
| RPS24 | Ribosomal protein S24 | | | P62847 |  |
| RPS25 | Ribosomal protein S25 | | | P62851 |  |
| RPS26 | Ribosomal protein S26 | | | P62854 |  |
| RPS27 | Ribosomal protein S27 | | | P42677 |  |
| RPS27A | Ribosomal protein S27a | | | P62979 |  |
| RPS27L | Ribosomal protein S27 like | | | Q71UM5 |  |
| RPS28 | Ribosomal protein S28 | | | P62857 |  |
| RPS29 | Ribosomal protein S29 | | | P62273 |  |
| RPS3 | Ribosomal protein S3 | | | P23396 |  |
| RPS3A | Ribosomal protein S3A | | | P61247 |  |
| RPS4X | Ribosomal protein S4 X-linked | | | P62701 |  |
| RPS4Y1 | Ribosomal protein S4 Y-linked 1 | | | P22090 |  |
| RPS5 | Ribosomal protein S5 | | | P46782 |  |
| RPS6 | Ribosomal protein S6 | | | P62753 |  |
| RPS6KA1 | Ribosomal protein S6 kinase A1 | | | Q15418 |  |
| RPS6KA3 | Ribosomal protein S6 kinase A3 | | | P51812 |  |
| RPS6KA5 | Ribosomal protein S6 kinase A5 | | | O75582 |  |
| RPS6KB1 | Ribosomal protein S6 kinase B1 | | | P23443 |  |
| RPS6KB2 | Ribosomal protein S6 kinase B2 | | | Q9UBS0 |  |
| RPS7 | Ribosomal protein S7 | | | P62081 |  |
| RPS8 | Ribosomal protein S8 | | | P62241 |  |
| RPS9 | Ribosomal protein S9 | | | P46781 |  |
| RPSA | Ribosomal protein SA | | | P08865 |  |
| RPUSD1 | RNA pseudouridine synthase domain containing 1 | | | Q9UJJ7 |  |
| RPUSD2 | RNA pseudouridine synthase domain containing 2 | | | Q8IZ73 |  |
| RPUSD3 | RNA pseudouridine synthase D3 | | | Q6P087 |  |
| RPUSD4 | RNA pseudouridine synthase D4 | | | Q96CM3 |  |
| RRAGA | Ras related GTP binding A | | | Q7L523 |  |
| RRAGB | Ras related GTP binding B | | | Q5VZM2 |  |
| RRAGC | Ras related GTP binding C | | | Q9HB90 |  |
| RRAS | RAS related | | | P10301 |  |
| RRAS2 | RAS related 2 | | | P62070 |  |
| RREB1 | Ras responsive element binding protein 1 | | | Q92766 |  |
| RRM1 | Ribonucleotide reductase catalytic subunit M1 | | | P23921 |  |
| RRM2B | Ribonucleotide reductase regulatory TP53 inducible subunit M2B | | | Q7LG56 |  |
| RRP1 | Ribosomal RNA processing 1 | | | P56182 |  |
| RRP12 | Ribosomal RNA processing 12 homolog | | | Q5JTH9 |  |
| RRP15 | Ribosomal RNA processing 15 homolog | | | Q9Y3B9 |  |
| RRP36 | Ribosomal RNA processing 36 | | | Q96EU6 |  |
| RRP7A | Ribosomal RNA processing 7 homolog A | | | Q9Y3A4 |  |
| RRP8 | Ribosomal RNA processing 8 | | | O43159 |  |
| RRP9 | Ribosomal RNA processing 9, U3 small nucleolar RNA binding protein | | | O43818 |  |
| RRS1 | Ribosome biogenesis regulator 1 homolog | | | Q15050 |  |
| RSAD1 | Radical S-adenosyl methionine domain containing 1 | | | Q9HA92 |  |
| RSAD2 | Radical S-adenosyl methionine domain containing 2 | | | Q8WXG1 |  |
| RSBN1 | Round spermatid basic protein 1 | | | Q5VWQ0 |  |
| RSBN1L | Round spermatid basic protein 1 like | | | Q6PCB5 |  |
| RSF1 | Remodeling and spacing factor 1 | | | Q96T23 |  |
| RSL1D1 | Ribosomal L1 domain containing 1 | | | O76021 |  |
| RSL24D1 | Ribosomal L24 domain containing 1 | | | Q9UHA3 |  |
| RSPRY1 | Ring finger and SPRY domain containing 1 | | | Q96DX4 |  |
| RSRC1 | Arginine and serine rich coiled-coil 1 | | | Q96IZ7 |  |
| RSRC2 | Arginine and serine rich coiled-coil 2 | | | Q7L4I2 |  |
| RSRP1 | Arginine and serine rich protein 1 | | | Q9BUV0 |  |
| RSU1 | Ras suppressor protein 1 | | | Q15404 |  |
| RTCA | RNA 3'-terminal phosphate cyclase | | | O00442 |  |
| RTCB | RNA 2',3'-cyclic phosphate and 5'-OH ligase | | | Q9Y3I0 |  |
| RTF1 | RTF1 homolog, Paf1/RNA polymerase II complex component | | | Q92541 |  |
| RTKN2 | Rhotekin 2 | | | Q8IZC4 |  |
| RTN3 | Reticulon 3 | | | O95197 |  |
| RTN4 | Reticulon 4 | | | Q9NQC3 |  |
| RTN4IP1 | Reticulon 4 interacting protein 1 | | | Q8WWV3 |  |
| RTN4R | Reticulon 4 receptor | | | Q9BZR6 |  |
| RTP4 | Receptor transporter protein 4 | | | Q96DX8 |  |
| RUBCN | Rubicon autophagy regulator | | | Q92622 |  |
| RUFY1 | RUN and FYVE domain containing 1 | | | Q96T51 |  |
| RUFY2 | RUN and FYVE domain containing 2 | | | Q8WXA3 |  |
| RUFY3 | RUN and FYVE domain containing 3 | | | Q7L099 |  |
| RUNDC1 | RUN domain containing 1 | | | Q96C34 |  |
| RUNX1 | RUNX family transcription factor 1 | | | Q01196 |  |
| RUNX2 | RUNX family transcription factor 2 | | | Q13950 |  |
| RUNX3 | RUNX family transcription factor 3 | | | Q13761 |  |
| RUSC1 | RUN and SH3 domain containing 1 | | | Q9BVN2 |  |
| RUVBL1 | RuvB like AAA ATPase 1 | | | Q9Y265 |  |
| RUVBL2 | RuvB like AAA ATPase 2 | | | Q9Y230 |  |
| RWDD1 | RWD domain containing 1 | | | Q9H446 |  |
| RWDD2A | RWD domain containing 2A | | | Q9UIY3 |  |
| RWDD3 | RWD domain containing 3 | | | Q9Y3V2 |  |
| RWDD4 | RWD domain containing 4 | | | Q6NW29 |  |
| RXRB | Retinoid X receptor beta | | | P28702 |  |
| RYK | Receptor like tyrosine kinase | | | P34925 |  |
| S100A10 | S100 calcium binding protein A10 | | | P60903 |  |
| S100A11 | S100 calcium binding protein A11 | | | P31949 |  |
| S100A12 | S100 calcium binding protein A12 | | | P80511 |  |
| S100A2 | S100 calcium binding protein A2 | | | P29034 |  |
| S100A4 | S100 calcium binding protein A4 | | | P26447 |  |
| S100A6 | S100 calcium binding protein A6 | | | P06703 |  |
| S100A8 | S100 calcium binding protein A8 | | | P05109 |  |
| S100A9 | S100 calcium binding protein A9 | | | P06702 |  |
| S100B | S100 calcium binding protein B | | | P04271 |  |
| S100P | S100 calcium binding protein P | | | P25815 |  |
| S1PR1 | Sphingosine-1-phosphate receptor 1 | | | P21453 |  |
| S1PR2 | Sphingosine-1-phosphate receptor 2 | | | O95136 |  |
| S1PR4 | Sphingosine-1-phosphate receptor 4 | | | O95977 |  |
| S1PR5 | Sphingosine-1-phosphate receptor 5 | | | Q9H228 |  |
| SAAL1 | Serum amyloid A like 1 | | | Q96ER3 |  |
| SAC3D1 | SAC3 domain containing 1 | | |  |  |
| SACM1L | SAC1 like phosphatidylinositide phosphatase | | | Q9NTJ5 |  |
| SAE1 | SUMO1 activating enzyme subunit 1 | | | Q9UBE0 |  |
| SAFB | Scaffold attachment factor B | | | Q15424 |  |
| SAFB2 | Scaffold attachment factor B2 | | | Q14151 |  |
| SAMD1 | Sterile alpha motif domain containing 1 | | |  |  |
| SAMD12 | Sterile alpha motif domain containing 12 | | | Q8N8I0 |  |
| SAMD3 | Sterile alpha motif domain containing 3 | | | Q8N6K7 |  |
| SAMD4B | Sterile alpha motif domain containing 4B | | | Q5PRF9 |  |
| SAMD8 | Sterile alpha motif domain containing 8 | | | Q96LT4 |  |
| SAMHD1 | SAM and HD domain containing deoxynucleoside triphosphate triphosphohydrolase 1 | | | Q9Y3Z3 |  |
| SAMM50 | SAMM50 sorting and assembly machinery component | | | Q9Y512 |  |
| SAMSN1 | SAM domain, SH3 domain and nuclear localization signals 1 | | | Q9NSI8 |  |
| SAP130 | Sin3A associated protein 130 | | | Q9H0E3 |  |
| SAP18 | Sin3A associated protein 18 | | | O00422 |  |
| SAP30BP | SAP30 binding protein | | | Q9UHR5 |  |
| SAR1A | Secretion associated Ras related GTPase 1A | | | Q9NR31 |  |
| SAR1B | Secretion associated Ras related GTPase 1B | | | Q9Y6B6 |  |
| SARAF | Store-operated calcium entry associated regulatory factor | | | Q96BY9 |  |
| SARNP | SAP domain containing ribonucleoprotein | | | P82979 |  |
| SARS2 | Seryl-tRNA synthetase 2, mitochondrial | | | Q9NP81 |  |
| SART1 | Spliceosome associated factor 1, recruiter of U4/U6.U5 tri-snRNP | | | O43290 |  |
| SART3 | Spliceosome associated factor 3, U4/U6 recycling protein | | | Q15020 |  |
| SASH3 | SAM and SH3 domain containing 3 | | | O75995 |  |
| SAT1 | Spermidine/spermine N1-acetyltransferase 1 | | | P21673 |  |
| SAT2 | Spermidine/spermine N1-acetyltransferase family member 2 | | | Q96F10 |  |
| SATB1 | SATB homeobox 1 | | | Q01826 |  |
| SATB1 | SATB homeobox 1 | | | Q01826 |  |
| SAYSD1 | SAYSVFN motif domain containing 1 | | | Q9NPB0 |  |
| SBDS | SBDS ribosome maturation factor | | | Q9Y3A5 |  |
| SBF1 | SET binding factor 1 | | | O95248 |  |
| SC5D | Sterol-C5-desaturase | | | O75845 |  |
| SCAF11 | SR-related CTD associated factor 11 | | | Q99590 |  |
| SCAF4 | SR-related CTD associated factor 4 | | | O95104 |  |
| SCAF8 | SR-related CTD associated factor 8 | | | Q9UPN6 |  |
| SCAMP1 | Secretory carrier membrane protein 1 | | | O15126 |  |
| SCAMP2 | Secretory carrier membrane protein 2 | | | O15127 |  |
| SCAMP3 | Secretory carrier membrane protein 3 | | | O14828 |  |
| SCAMP4 | Secretory carrier membrane protein 4 | | | Q969E2 |  |
| SCAND1 | SCAN domain containing 1 | | | P57086 |  |
| SCAP | SREBF chaperone | | | Q12770 |  |
| SCAPER | S-phase cyclin A associated protein in the ER | | | Q9BY12 |  |
| SCARB2 | Scavenger receptor class B member 2 | | | Q14108 |  |
| SCCPDH | Saccharopine dehydrogenase (putative) | | | Q8NBX0 |  |
| SCFD1 | Sec1 family domain containing 1 | | | Q8WVM8 |  |
| SCFD2 | Sec1 family domain containing 2 | | | Q8WU76 |  |
| SCGB3A1 | Secretoglobin family 3A member 1 | | | Q96QR1 |  |
| SCIMP | SLP adaptor and CSK interacting membrane protein | | | Q6UWF3 |  |
| SCLT1 | Sodium channel and clathrin linker 1 | | | Q96NL6 |  |
| SCLY | Selenocysteine lyase | | | Q96I15 |  |
| SCMH1 | Scm polycomb group protein homolog 1 | | | Q96GD3 |  |
| SCML1 | Scm polycomb group protein like 1 | | | Q9UN30 |  |
| SCML1 | Scm polycomb group protein like 1 | | | Q9UN30 |  |
| SCML4 | Scm polycomb group protein like 4 | | | Q8N228 |  |
| SCNM1 | Sodium channel modifier 1 | | | Q9BWG6 |  |
| SCO1 | Synthesis of cytochrome C oxidase 1 | | | O75880 |  |
| SCO2 | Synthesis of cytochrome C oxidase 2 | | | O43819 |  |
| SCOC | Short coiled-coil protein | | | Q9UIL1 |  |
| SCP2 | Sterol carrier protein 2 | | | P22307 |  |
| SCPEP1 | Serine carboxypeptidase 1 | | | Q9HB40 |  |
| SCRN2 | Secernin 2 | | | Q96FV2 |  |
| SCRN3 | Secernin 3 | | | Q0VDG4 |  |
| SCYL1 | SCY1 like pseudokinase 1 | | | Q96KG9 |  |
| SCYL2 | SCY1 like pseudokinase 2 | | | Q6P3W7 |  |
| SCYL3 | SCY1 like pseudokinase 3 | | | Q8IZE3 |  |
| SDC4 | Syndecan 4 | | | P31431 |  |
| SDCBP | Syndecan binding protein | | | O00560 |  |
| SDCBP2 | Syndecan binding protein 2 | | | Q9H190 |  |
| SDCCAG8 | SHH signaling and ciliogenesis regulator SDCCAG8 | | | Q86SQ7 |  |
| SDE2 | SDE2 telomere maintenance homolog | | | Q6IQ49 |  |
| SDF2 | Stromal cell derived factor 2 | | | Q99470 |  |
| SDF2L1 | Stromal cell derived factor 2 like 1 | | | Q9HCN8 |  |
| SDF4 | Stromal cell derived factor 4 | | |  |  |
| SDHA | Succinate dehydrogenase complex flavoprotein subunit A | | | P31040 |  |
| SDHAF1 | Succinate dehydrogenase complex assembly factor 1 | | | A6NFY7 |  |
| SDHAF2 | Succinate dehydrogenase complex assembly factor 2 | | | Q9NX18 |  |
| SDHAF3 | Succinate dehydrogenase complex assembly factor 3 | | | Q9NRP4 |  |
| SDHAF4 | Succinate dehydrogenase complex assembly factor 4 | | | Q5VUM1 |  |
| SDHB | Succinate dehydrogenase complex iron sulfur subunit B | | | P21912 |  |
| SDHC | Succinate dehydrogenase complex subunit C | | | Q99643 |  |
| SDHD | Succinate dehydrogenase complex subunit D | | | O14521 |  |
| SDR39U1 | Short chain dehydrogenase/reductase family 39U member 1 | | | Q9NRG7 |  |
| SDR42E1 | Short chain dehydrogenase/reductase family 42E, member 1 | | | Q8WUS8 |  |
| SEC11A | SEC11 homolog A, signal peptidase complex subunit | | | P67812 |  |
| SEC13 | SEC13 homolog, nuclear pore and COPII coat complex component | | | P55735 |  |
| SEC14L2 | SEC14 like lipid binding 2 | | | O76054 |  |
| SEC14L2 | SEC14 like lipid binding 2 | | | O76054 |  |
| SEC22B | SEC22 homolog B, vesicle trafficking protein | | | O75396 |  |
| SEC22C | SEC22 homolog C, vesicle trafficking protein | | | Q9BRL7 |  |
| SEC23A | SEC23 homolog A, COPII coat complex component | | | Q15436 |  |
| SEC23B | SEC23 homolog B, COPII coat complex component | | | Q15437 |  |
| SEC23IP | SEC23 interacting protein | | | Q9Y6Y8 |  |
| SEC24C | SEC24 homolog C, COPII coat complex component | | | P53992 |  |
| SEC24D | SEC24 homolog D, COPII coat complex component | | | O94855 |  |
| SEC31A | SEC31 homolog A, COPII coat complex component | | | O94979 |  |
| SEC61A1 | SEC61 translocon subunit alpha 1 | | | P61619 |  |
| SEC61A2 | SEC61 translocon subunit alpha 2 | | | Q9H9S3 |  |
| SEC61B | SEC61 translocon subunit beta | | | P60468 |  |
| SEC61G | SEC61 translocon subunit gamma | | | P60059 |  |
| SEC62 | SEC62 homolog, preprotein translocation factor | | | Q99442 |  |
| SEC63 | SEC63 homolog, protein translocation regulator | | | Q9UGP8 |  |
| SECISBP2 | SECIS binding protein 2 | | | Q96T21 |  |
| SECISBP2L | SECIS binding protein 2 like | | | Q93073 |  |
| SEH1L | SEH1 like nucleoporin | | | Q96EE3 |  |
| SEL1L3 | SEL1L family member 3 | | | Q68CR1 |  |
| SELL | Selectin L | | | P14151 |  |
| SELPLG | Selectin P ligand | | | Q14242 |  |
| SEMA4A | Semaphorin 4A | | | Q9H3S1 |  |
| SEMA4D | Semaphorin 4D | | | Q92854 |  |
| SEMA4F | Ssemaphorin 4F | | | O95754 |  |
| SENP1 | SUMO specific peptidase 1 | | | Q9P0U3 |  |
| SENP2 | SUMO specific peptidase 2 | | | Q9HC62 |  |
| SENP5 | SUMO specific peptidase 5 | | | Q96HI0 |  |
| SENP6 | SUMO specific peptidase 6 | | | Q9GZR1 |  |
| SENP7 | SUMO specific peptidase 7 | | | Q9BQF6 |  |
| SENP8 | SUMO peptidase family member, NEDD8 specific | | | Q96LD8 |  |
| SEPHS1 | Selenophosphate synthetase 1 | | | P49903 |  |
| SEPTIN1 | Septin 1 | | | Q8WYJ6 |  |
| SEPTIN10 | Septin 10 | | | Q9P0V9 |  |
| SEPTIN11 | Septin 11 | | | Q9NVA2 |  |
| SEPTIN2 | Septin 2 | | | Q15019 |  |
| SEPTIN4 | Septin 4 | | | O43236 |  |
| SEPTIN6 | Septin 6 | | | Q14141 |  |
| SEPTIN7 | Septin 7 | | | Q16181 |  |
| SEPTIN8 | Septin 8 | | | Q92599 |  |
| SEPTIN9 | Septin 9 | | | Q9UHD8 |  |
| SERBP1 | SERPINE1 mRNA binding protein 1 | | | Q8NC51 |  |
| SERF1A | Small EDRK-rich factor 1A | | | O75920 |  |
| SERF2 | Small EDRK-rich factor 2 | | | P84101 |  |
| SERGEF | Secretion regulating guanine nucleotide exchange factor | | | Q9UGK8 |  |
| SERINC1 | Serine incorporator 1 | | | Q9NRX5 |  |
| SERINC3 | Serine incorporator 3 | | | Q13530 |  |
| SERINC5 | Serine incorporator 5 | | | Q86VE9 |  |
| SERP1 | Stress associated endoplasmic reticulum protein 1 | | | Q9Y6X1 |  |
| SERPINA1 | Serpin family A member 1 | | | P01009 |  |
| SERPINB1 | Serpin family B member 1 | | | P30740 |  |
| SERPINB6 | Serpin family B member 6 | | | P35237 |  |
| SERPINB8 | Serpin family B member 8 | | | P50452 |  |
| SERPINB9 | Serpin family B member 9 | | | P50453 |  |
| SERPINE2 | Serpin family E member 2 | | | P07093 |  |
| SERPINF1 | Serpin family F member 1 | | | P36955 |  |
| SERPINH1 | Serpin family H member 1 | | | P50454 |  |
| SERTAD1 | SERTA domain containing 1 | | | Q9UHV2 |  |
| SERTAD2 | SERTA domain containing 2 | | | Q14140 |  |
| SERTAD3 | SERTA domain containing 3 | | | Q9UJW9 |  |
| SESN2 | Sestrin 2 | | | P58004 |  |
| SESN3 | Sestrin 3 | | | P58005 |  |
| SET | SET nuclear proto-oncogene | | | Q01105 |  |
| SETD3 | SET domain containing 3, actin histidine methyltransferase | | | Q86TU7 |  |
| SETD4 | SET domain containing 4 | | | Q9NVD3 |  |
| SETD6 | SET domain containing 6, protein lysine methyltransferase | | | Q8TBK2 |  |
| SETD7 | SET domain containing 7, histone lysine methyltransferase | | | Q8WTS6 |  |
| SETDB1 | SET domain bifurcated histone lysine methyltransferase 1 | | | Q15047 |  |
| SETDB2 | SET domain bifurcated histone lysine methyltransferase 2 | | | Q96T68 |  |
| SETMAR | SET domain and mariner transposase fusion gene | | | Q53H47 |  |
| SF1 | Splicing factor 1 | | | Q15637 |  |
| SF3A1 | Splicing factor 3a subunit 1 | | | Q15459 |  |
| SF3A2 | Splicing factor 3a subunit 2 | | | Q15428 |  |
| SF3A3 | Splicing factor 3a subunit 3 | | | Q12874 |  |
| SF3B2 | Splicing factor 3b subunit 2 | | | Q13435 |  |
| SF3B3 | Splicing factor 3b subunit 3 | | | Q15393 |  |
| SF3B4 | Splicing factor 3b subunit 4 | | | Q15427 |  |
| SF3B5 | Splicing factor 3b subunit 5 | | | Q9BWJ5 |  |
| SF3B6 | Splicing factor 3b subunit 6 | | | Q9Y3B4 |  |
| SFMBT1 | Scm like with four mbt domains 1 | | | Q9UHJ3 |  |
| SFPQ | Splicing factor proline and glutamine rich | | | P23246 |  |
| SFR1 | SWI5 dependent homologous recombination repair protein 1 | | | Q86XK3 |  |
| SFSWAP | Splicing factor SWAP | | | Q12872 |  |
| SFT2D1 | SFT2 domain containing 1 | | | Q8WV19 |  |
| SFT2D2 | SFT2 domain containing 2 | | | O95562 |  |
| SFXN1 | Sideroflexin 1 | | | Q9H9B4 |  |
| SFXN2 | Sideroflexin 2 | | | Q96NB2 |  |
| SFXN3 | Sideroflexin 3 | | | Q9BWM7 |  |
| SFXN4 | Sideroflexin 4 | | | Q6P4A7 |  |
| SGCB | Sarcoglycan beta | | | Q16585 |  |
| SGF29 | SAGA complex associated factor 29 | | | Q96ES7 |  |
| SGK1 | Serum/glucocorticoid regulated kinase 1 | | | O00141 |  |
| SGK3 | Serum/glucocorticoid regulated kinase family member 3 | | | Q96BR1 |  |
| SGPL1 | Sphingosine-1-phosphate lyase 1 | | | O95470 |  |
| SGSH | N-sulfoglucosamine sulfohydrolase | | | P51688 |  |
| SGSM3 | Small G protein signaling modulator 3 | | | Q96HU1 |  |
| SGTA | Small glutamine rich tetratricopeptide repeat co-chaperone alpha | | | O43765 |  |
| SGTB | Small glutamine rich tetratricopeptide repeat co-chaperone beta | | | Q96EQ0 |  |
| SH2B1 | SH2B adaptor protein 1 | | | Q9NRF2 |  |
| SH2D1A | SH2 domain containing 1A | | | O60880 |  |
| SH2D2A | SH2 domain containing 2A | | | Q9NP31 |  |
| SH2D3A | SH2 domain containing 3A | | | Q9BRG2 |  |
| SH2D3C | SH2 domain containing 3C | | | Q8N5H7 |  |
| SH3BGR | SH3 domain binding glutamate rich protein | | | P55822 |  |
| SH3BGRL | SH3 domain binding glutamate rich protein like | | | O75368 |  |
| SH3BGRL3 | SH3 domain binding glutamate rich protein like 3 | | | Q9H299 |  |
| SH3BP1 | SH3 domain binding protein 1 | | | Q9Y3L3 |  |
| SH3BP2 | SH3 domain binding protein 2 | | | P78314 |  |
| SH3BP5 | SH3 domain binding protein 5 | | | O60239 |  |
| SH3BP5L | SH3 binding domain protein 5 like | | | Q7L8J4 |  |
| SH3GL1 | SH3 domain containing GRB2 like 1, endophilin A2 | | | Q99961 |  |
| SH3GLB1 | SH3 domain containing GRB2 like, endophilin B1 | | | Q9Y371 |  |
| SH3GLB2 | SH3 domain containing GRB2 like, endophilin B2 | | | Q9NR46 |  |
| SH3TC1 | SH3 domain and tetratricopeptide repeats 1 | | | Q8TE82 |  |
| SH3YL1 | SH3 and SYLF domain containing 1 | | | Q96HL8 |  |
| SHARPIN | SHANK associated RH domain interactor | | | Q9H0F6 |  |
| SHC1 | SHC adaptor protein 1 | | | P29353 |  |
| SHISA2 | Shisa family member 2 | | | Q6UWI4 |  |
| SHISA5 | Shisa family member 5 | | | Q8N114 |  |
| SHKBP1 | SH3KBP1 binding protein 1 | | | Q8TBC3 |  |
| SHMT1 | Serine hydroxymethyltransferase 1 | | | P34896 |  |
| SHMT2 | Serine hydroxymethyltransferase 2 | | | P34897 |  |
| SHOC2 | SHOC2 leucine rich repeat scaffold protein | | | Q9UQ13 |  |
| SHPK | Sedoheptulokinase | | | Q9UHJ6 |  |
| SHQ1 | SHQ1, H/ACA ribonucleoprotein assembly factor | | | Q6PI26 |  |
| SIAE | Sialic acid acetylesterase | | | Q9HAT2 |  |
| SIAH1 | Siah E3 ubiquitin protein ligase 1 | | | Q8IUQ4 |  |
| SIAH2 | Siah E3 ubiquitin protein ligase 2 | | | O43255 |  |
| SIGIRR | Single Ig and TIR domain containing | | | Q6IA17 |  |
| SIGLEC9 | Sialic acid binding Ig like lectin 9 | | | Q9Y336 |  |
| SIGMAR1 | Sigma non-opioid intracellular receptor 1 | | | Q99720 |  |
| SIKE1 | Suppressor of IKBKE 1 | | | Q9BRV8 |  |
| SIL1 | SIL1 nucleotide exchange factor | | | Q9H173 |  |
| SIMC1 | SUMO interacting motifs containing 1 | | | Q8NDZ2 |  |
| SIN3A | SIN3 transcription regulator family member A | | | Q96ST3 |  |
| SIN3B | SIN3 transcription regulator family member B | | | O75182 |  |
| SIRPB1 | Signal regulatory protein beta 1 | | | O00241, Q5TFQ8 |  |
| SIRPG | Signal regulatory protein gamma | | | Q9P1W8 |  |
| SIRT1 | Sirtuin 1 | | | Q96EB6 |  |
| SIRT2 | Sirtuin 2 | | | Q8IXJ6 |  |
| SIRT3 | Sirtuin 3 | | | Q9NTG7 |  |
| SIRT4 | Sirtuin 4 | | | Q9Y6E7 |  |
| SIRT5 | Sirtuin 5 | | | Q9NXA8 |  |
| SIRT6 | Sirtuin 6 | | | Q8N6T7 |  |
| SIRT7 | Sirtuin 7 | | | Q9NRC8 |  |
| SIVA1 | SIVA1 apoptosis inducing factor | | | O15304 |  |
| SKA2 | Spindle and kinetochore associated complex subunit 2 | | | Q8WVK7 |  |
| SKAP1 | Src kinase associated phosphoprotein 1 | | | Q86WV1 |  |
| SKAP2 | Src kinase associated phosphoprotein 2 | | | O75563 |  |
| SKIL | SKI like proto-oncogene | | | P12757 |  |
| SKP1 | S-phase kinase associated protein 1 | | | P63208 |  |
| SLA | Src like adaptor | | | Q13239 |  |
| SLA2 | Src like adaptor 2 | | | Q9H6Q3 |  |
| SLAIN1 | SLAIN motif family member 1 | | | Q8ND83 |  |
| SLAIN2 | SLAIN motif family member 2 | | | Q9P270 |  |
| SLAMF1 | Signaling lymphocytic activation molecule family member 1 | | | Q13291 |  |
| SLAMF6 | SLAM family member 6 | | | Q96DU3 |  |
| SLAMF7 | SLAM family member 7 | | | Q9NQ25 |  |
| SLAMF8 | SLAM family member 8 | | | Q9P0V8 |  |
| SLBP | Stem-loop binding protein | | | Q14493 |  |
| SLC10A3 | Solute carrier family 10 member 3 | | | P09131 |  |
| SLC10A7 | Solute carrier family 10 member 7 | | | Q0GE19 |  |
| SLC11A2 | Solute carrier family 11 member 2 | | | P49281 |  |
| SLC12A6 | Solute carrier family 12 member 6 | | | Q9UHW9 |  |
| SLC12A7 | Solute carrier family 12 member 7 | | | Q9Y666 |  |
| SLC16A1 | Solute carrier family 16 member 1 | | | P53985 |  |
| SLC16A10 | Solute carrier family 16 member 10 | | | Q8TF71 |  |
| SLC16A3 | Solute carrier family 16 member 3 | | | O15427 |  |
| SLC16A5 | Solute carrier family 16 member 5 | | | O15375 |  |
| SLC16A7 | Solute carrier family 16 member 7 | | | O60669 |  |
| SLC17A5 | Solute carrier family 17 member 5 | | | Q9NRA2 |  |
| SLC19A1 | Solute carrier family 19 member 1 | | | P41440 |  |
| SLC19A2 | Solute carrier family 19 member 2 | | | O60779 |  |
| SLC1A5 | Solute carrier family 1 member 5 | | | Q15758 |  |
| SLC20A1 | Solute carrier family 20 member 1 | | | Q8WUM9 |  |
| SLC20A2 | Solute carrier family 20 member 2 | | | Q08357 |  |
| SLC22A18 | Solute carrier family 22 member 18 | | | Q96BI1 |  |
| SLC25A1 | Solute carrier family 25 member 1 | | | P53007 |  |
| SLC25A11 | Solute carrier family 25 member 11 | | | Q02978 |  |
| SLC25A13 | Solute carrier family 25 member 13 | | | Q9UJS0 |  |
| SLC25A14 | Solute carrier family 25 member 14 | | | O95258 |  |
| SLC25A15 | Solute carrier family 25 member 15 | | | Q9Y619 |  |
| SLC25A17 | Solute carrier family 25 member 17 | | | O43808 |  |
| SLC25A19 | Solute carrier family 25 member 19 | | | Q9HC21 |  |
| SLC25A20 | Solute carrier family 25 member 20 | | | O43772 |  |
| SLC25A22 | Solute carrier family 25 member 22 | | | Q9H936 |  |
| SLC25A23 | Solute carrier family 25 member 23 | | | Q9BV35 |  |
| SLC25A25 | Solute carrier family 25 member 25 | | | Q6KCM7 |  |
| SLC25A26 | Solute carrier family 25 member 26 | | | Q70HW3 |  |
| SLC25A28 | Solute carrier family 25 member 28 | | | Q96A46 |  |
| SLC25A3 | Solute carrier family 25 member 3 | | | Q00325 |  |
| SLC25A30 | Solute carrier family 25 member 30 | | | Q5SVS4 |  |
| SLC25A32 | Solute carrier family 25 member 32 | | | Q9H2D1 |  |
| SLC25A35 | Solute carrier family 25 member 35 | | | Q3KQZ1 |  |
| SLC25A36 | Solute carrier family 25 member 36 | | | Q96CQ1 |  |
| SLC25A37 | Solute carrier family 25 member 37 | | | Q9NYZ2 |  |
| SLC25A38 | Solute carrier family 25 member 38 | | | Q96DW6 |  |
| SLC25A39 | Solute carrier family 25 member 39 | | | Q9BZJ4 |  |
| SLC25A40 | Solute carrier family 25 member 40 | | | Q8TBP6 |  |
| SLC25A42 | Solute carrier family 25 member 42 | | | Q86VD7 |  |
| SLC25A43 | Solute carrier family 25 member 43 | | | Q8WUT9 |  |
| SLC25A44 | Solute carrier family 25 member 44 | | | Q96H78 |  |
| SLC25A45 | Solute carrier family 25 member 45 | | | Q8N413 |  |
| SLC25A46 | Solute carrier family 25 member 46 | | | Q96AG3 |  |
| SLC25A5 | Solute carrier family 25 member 5 | | | P05141 |  |
| SLC25A51 | Solute carrier family 25 member 51 | | | Q9H1U9 |  |
| SLC25A6 | Solute carrier family 25 member 6 | | | P12236 |  |
| SLC26A6 | Solute carrier family 26 member 6 | | | Q9BXS9 |  |
| SLC27A1 | Solute carrier family 27 member 1 | | | Q6PCB7 |  |
| SLC27A5 | Solute carrier family 27 member 5 | | | Q9Y2P5 |  |
| SLC29A1 | Solute carrier family 29 member 1 (Augustine blood group) | | | Q99808 |  |
| SLC29A2 | Solute carrier family 29 member 2 | | | Q14542 |  |
| SLC29A3 | Solute carrier family 29 member 3 | | | Q9BZD2 |  |
| SLC2A1 | Solute carrier family 2 member 1 | | | P11166 |  |
| SLC2A11 | Solute carrier family 2 member 11 | | | Q9BYW1 |  |
| SLC2A3 | Solute carrier family 2 member 3 | | | P11169 |  |
| SLC2A8 | Solute carrier family 2 member 8 | | | Q9NY64 |  |
| SLC30A4 | Solute carrier family 30 member 4 | | | O14863 |  |
| SLC30A5 | Solute carrier family 30 member 5 | | | Q8TAD4 |  |
| SLC30A6 | Solute carrier family 30 member 6 | | | Q6NXT4 |  |
| SLC30A7 | Solute carrier family 30 member 7 | | | Q8NEW0 |  |
| SLC30A9 | Solute carrier family 30 member 9 | | | Q6PML9 |  |
| SLC31A1 | Solute carrier family 31 member 1 | | | O15431 |  |
| SLC31A2 | Solute carrier family 31 member 2 | | | O15432 |  |
| SLC33A1 | Solute carrier family 33 member 1 | | | O00400 |  |
| SLC35A1 | Solute carrier family 35 member A1 | | | P78382 |  |
| SLC35A2 | Solute carrier family 35 member A2 | | | P78381 |  |
| SLC35A3 | Solute carrier family 35 member A3 | | | Q9Y2D2 |  |
| SLC35A4 | Solute carrier family 35 member A4 | | | L0R6Q1, Q96G79 |  |
| SLC35A5 | Solute carrier family 35 member A5 | | | Q9BS91 |  |
| SLC35B1 | Solute carrier family 35 member B1 | | | P78383 |  |
| SLC35B2 | Solute carrier family 35 member B2 | | | Q8TB61 |  |
| SLC35B3 | Solute carrier family 35 member B3 | | | Q9H1N7 |  |
| SLC35B4 | Solute carrier family 35 member B4 | | | Q969S0 |  |
| SLC35C1 | Solute carrier family 35 member C1 | | | Q96A29 |  |
| SLC35C2 | Solute carrier family 35 member C2 | | | Q9NQQ7 |  |
| SLC35D1 | Solute carrier family 35 member D1 | | | Q9NTN3 |  |
| SLC35D2 | Solute carrier family 35 member D2 | | | Q76EJ3 |  |
| SLC35E2B | Solute carrier family 35 member E2B | | | P0CK96 |  |
| SLC35F2 | Solute carrier family 35 member F2 | | | Q8IXU6 |  |
| SLC35F6 | Solute carrier family 35 member F6 | | | Q8N357 |  |
| SLC36A1 | Solute carrier family 36 member 1 | | | Q7Z2H8 |  |
| SLC36A4 | Solute carrier family 36 member 4 | | | Q6YBV0 |  |
| SLC37A1 | Solute carrier family 37 member 1 | | | P57057 |  |
| SLC37A3 | Solute carrier family 37 member 3 | | | Q8NCC5 |  |
| SLC37A4 | Solute carrier family 37 member 4 | | |  |  |
| SLC38A1 | Solute carrier family 38 member 1 | | | Q9H2H9 |  |
| SLC38A10 | Solute carrier family 38 member 10 | | | Q9HBR0 |  |
| SLC38A2 | Solute carrier family 38 member 2 | | | Q96QD8 |  |
| SLC38A5 | Solute carrier family 38 member 5 | | | Q8WUX1 |  |
| SLC38A6 | Solute carrier family 38 member 6 | | | Q8IZM9 |  |
| SLC38A7 | Solute carrier family 38 member 7 | | | Q9NVC3 |  |
| SLC39A1 | Solute carrier family 39 member 1 | | | Q9NY26 |  |
| SLC39A10 | Solute carrier family 39 member 10 | | | Q9ULF5 |  |
| SLC39A11 | Solute carrier family 39 member 11 | | | Q8N1S5 |  |
| SLC39A13 | Solute carrier family 39 member 13 | | | Q96H72 |  |
| SLC39A14 | Solute carrier family 39 member 14 | | | Q15043 |  |
| SLC39A3 | Solute carrier family 39 member 3 | | | Q9BRY0 |  |
| SLC39A6 | Solute carrier family 39 member 6 | | | Q13433 |  |
| SLC39A7 | Solute carrier family 39 member 7 | | | Q92504 |  |
| SLC39A8 | Solute carrier family 39 member 8 | | | Q9C0K1 |  |
| SLC39A9 | Solute carrier family 39 member 9 | | | Q9NUM3 |  |
| SLC3A2 | Solute carrier family 3 member 2 | | | P08195 |  |
| SLC40A1 | Solute carrier family 40 member 1 | | | Q9NP59 |  |
| SLC41A1 | Solute carrier family 41 member 1 | | | Q8IVJ1 |  |
| SLC41A3 | Solute carrier family 41 member 3 | | | Q96GZ6 |  |
| SLC43A1 | Solute carrier family 43 member 1 | | | O75387 |  |
| SLC43A3 | Solute carrier family 43 member 3 | | | Q8NBI5 |  |
| SLC44A1 | Solute carrier family 44 member 1 | | | Q8WWI5 |  |
| SLC44A2 | Solute carrier family 44 member 2 | | | Q8IWA5 |  |
| SLC46A3 | Solute carrier family 46 member 3 | | | Q7Z3Q1 |  |
| SLC48A1 | Solute carrier family 48 member 1 | | | Q6P1K1 |  |
| SLC4A1AP | Solute carrier family 4 member 1 adaptor protein | | | Q9BWU0 |  |
| SLC4A7 | Solute carrier family 4 member 7 | | | Q9Y6M7 |  |
| SLC50A1 | Solute carrier family 50 member 1 | | | Q9BRV3 |  |
| SLC5A5 | Solute carrier family 5 member 5 | | | Q92911 |  |
| SLC5A6 | Solute carrier family 5 member 6 | | | Q9Y289 |  |
| SLC6A16 | Solute carrier family 6 member 16 | | | Q9GZN6 |  |
| SLC6A6 | Solute carrier family 6 member 6 | | | P31641 |  |
| SLC7A6 | Solute carrier family 7 member 6 | | | Q92536 |  |
| SLC7A6OS | Solute carrier family 7 member 6 opposite strand | | | Q96CW6 |  |
| SLC8B1 | Solute carrier family 8 member B1 | | | Q6J4K2 |  |
| SLC9A3R1 | SLC9A3 regulator 1 | | | O14745 |  |
| SLC9A6 | Solute carrier family 9 member A6 | | | Q92581 |  |
| SLC9B1 | Solute carrier family 9 member B1 | | | Q4ZJI4 |  |
| SLC9B2 | Solute carrier family 9 member B2 | | | Q86UD5 |  |
| SLCO3A1 | Solute carrier organic anion transporter family member 3A1 | | | Q9UIG8 |  |
| SLF1 | SMC5-SMC6 complex localization factor 1 | | | Q9BQI6 |  |
| SLF2 | SMC5-SMC6 complex localization factor 2 | | | Q8IX21 |  |
| SLFN11 | Schlafen family member 11 | | | Q7Z7L1 |  |
| SLFN5 | Schlafen family member 5 | | | Q08AF3 |  |
| SLIRP | SRA stem-loop interacting RNA binding protein | | | Q9GZT3 |  |
| SLTM | SAFB like transcription modulator | | | Q9NWH9 |  |
| SLU7 | SLU7 homolog, splicing factor | | | O95391 |  |
| SLX1A | SLX1 homolog A, structure-specific endonuclease subunit | | | Q9BQ83 |  |
| SMAD2 | SMAD family member 2 | | | Q15796 |  |
| SMAD3 | SMAD family member 3 | | | P84022 |  |
| SMAD4 | SMAD family member 4 | | | Q13485 |  |
| SMAD5 | SMAD family member 5 | | | Q99717 |  |
| SMAGP | Small cell adhesion glycoprotein | | | Q0VAQ4 |  |
| SMAP1 | Small ArfGAP 1 | | | Q8IYB5 |  |
| SMAP2 | Small ArfGAP2 | | | Q8WU79 |  |
| SMARCA2 | SWI/SNF related, matrix associated, actin dependent regulator of chromatin, subfamily a, member 2 | | | P51531 |  |
| SMARCA4 | SWI/SNF related, matrix associated, actin dependent regulator of chromatin, subfamily a, member 4 | | | P51532 |  |
| SMARCA5 | SWI/SNF related, matrix associated, actin dependent regulator of chromatin, subfamily a, member 5 | | | O60264 |  |
| SMARCAD1 | SWI/SNF-related, matrix-associated actin-dependent regulator of chromatin, subfamily a, containing DEAD/H box 1 | | | Q9H4L7 |  |
| SMARCAL1 | SWI/SNF related, matrix associated, actin dependent regulator of chromatin, subfamily a like 1 | | | Q9NZC9 |  |
| SMARCB1 | SWI/SNF related, matrix associated, actin dependent regulator of chromatin, subfamily b, member 1 | | | Q12824 |  |
| SMARCC1 | SWI/SNF related, matrix associated, actin dependent regulator of chromatin subfamily c member 1 | | | Q92922 |  |
| SMARCC2 | SWI/SNF related, matrix associated, actin dependent regulator of chromatin subfamily c member 2 | | | Q8TAQ2 |  |
| SMARCD1 | SWI/SNF related, matrix associated, actin dependent regulator of chromatin, subfamily d, member 1 | | | Q96GM5 |  |
| SMARCE1 | SWI/SNF related, matrix associated, actin dependent regulator of chromatin, subfamily e, member 1 | | | Q969G3 |  |
| SMC1A | Structural maintenance of chromosomes 1A | | | Q14683 |  |
| SMC4 | Structural maintenance of chromosomes 4 | | | Q9NTJ3 |  |
| SMC5 | Structural maintenance of chromosomes 5 | | | Q8IY18 |  |
| SMC6 | Structural maintenance of chromosomes 6 | | | Q96SB8 |  |
| SMCO4 | Single-pass membrane protein with coiled-coil domains 4 | | | Q9NRQ5 |  |
| SMDT1 | Single-pass membrane protein with aspartate rich tail 1 | | | Q9H4I9 |  |
| SMG5 | SMG5 nonsense mediated mRNA decay factor | | | Q9UPR3 |  |
| SMG6 | SMG6 nonsense mediated mRNA decay factor | | | Q86US8 |  |
| SMG9 | SMG9 nonsense mediated mRNA decay factor | | | Q9H0W8 |  |
| SMIM12 | Small integral membrane protein 12 | | | Q96EX1 |  |
| SMIM14 | Small integral membrane protein 14 | | | Q96QK8 |  |
| SMIM19 | Small integral membrane protein 19 | | | Q96E16 |  |
| SMIM3 | Small integral membrane protein 3 | | | Q9BZL3 |  |
| SMIM8 | Small integral membrane protein 8 | | | Q96KF7 |  |
| SMN1 | Survival of motor neuron 1, telomeric | | | Q16637 |  |
| SMNDC1 | Survival motor neuron domain containing 1 | | | O75940 |  |
| SMPD1 | Sphingomyelin phosphodiesterase 1 | | | P17405 |  |
| SMPD2 | Sphingomyelin phosphodiesterase 2 | | | O60906 |  |
| SMS | Spermine synthase | | | P52788 |  |
| SMU1 | SMU1 DNA replication regulator and spliceosomal factor | | | Q2TAY7 |  |
| SMUG1 | Single-strand-selective monofunctional uracil-DNA glycosylase 1 | | | Q53HV7 |  |
| SMYD2 | SET and MYND domain containing 2 | | | Q9NRG4 |  |
| SMYD3 | SET and MYND domain containing 3 | | | Q9H7B4 |  |
| SMYD4 | SET and MYND domain containing 4 | | | Q8IYR2 |  |
| SNAI3 | Snail family transcriptional repressor 3 | | | Q3KNW1 |  |
| SNAP23 | Synaptosome associated protein 23 | | | O00161 |  |
| SNAP29 | Synaptosome associated protein 29 | | | O95721 |  |
| SNAP47 | Synaptosome associated protein 47 | | | Q5SQN1 |  |
| SNAPC1 | Small nuclear RNA activating complex polypeptide 1 | | | Q16533 |  |
| SNAPC2 | Small nuclear RNA activating complex polypeptide 2 | | | Q13487 |  |
| SNAPC5 | Small nuclear RNA activating complex polypeptide 5 | | | O75971 |  |
| SNAPIN | SNAP associated protein | | | O95295 |  |
| SNCA | Synuclein alpha | | | P37840 |  |
| SND1 | Staphylococcal nuclease and tudor domain containing 1 | | | Q7KZF4 |  |
| SNF8 | SNF8 subunit of ESCRT-II | | | Q96H20 |  |
| SNIP1 | Smad nuclear interacting protein 1 | | | Q8TAD8 |  |
| SNN | Stannin | | | O75324 |  |
| SNPH | Syntaphilin | | | O15079 |  |
| SNRK | SNF related kinase | | | Q9NRH2 |  |
| SNRNP200 | Small nuclear ribonucleoprotein U5 subunit 200 | | | O75643 |  |
| SNRNP25 | Small nuclear ribonucleoprotein U11/U12 subunit 25 | | | Q9BV90 |  |
| SNRNP27 | Small nuclear ribonucleoprotein U4/U6.U5 subunit 27 | | | Q8WVK2 |  |
| SNRNP35 | Small nuclear ribonucleoprotein U11/U12 subunit 35 | | | Q16560 |  |
| SNRNP40 | Small nuclear ribonucleoprotein U5 subunit 40 | | | Q96DI7 |  |
| SNRNP70 | Small nuclear ribonucleoprotein U1 subunit 70 | | | P08621 |  |
| SNRPA | Small nuclear ribonucleoprotein polypeptide A | | | P09012 |  |
| SNRPA1 | Small nuclear ribonucleoprotein polypeptide A' | | | P09661 |  |
| SNRPB | Small nuclear ribonucleoprotein polypeptides B and B1 | | | P14678 |  |
| SNRPB2 | Small nuclear ribonucleoprotein polypeptide B2 | | | P08579 |  |
| SNRPC | Small nuclear ribonucleoprotein polypeptide C | | | P09234 |  |
| SNRPD1 | Small nuclear ribonucleoprotein D1 polypeptide | | | P62314 |  |
| SNRPD2 | Small nuclear ribonucleoprotein D2 polypeptide | | | P62316 |  |
| SNRPD3 | Small nuclear ribonucleoprotein D3 polypeptide | | | P62318 |  |
| SNRPE | Small nuclear ribonucleoprotein polypeptide E | | | P62304 |  |
| SNRPF | Small nuclear ribonucleoprotein polypeptide F | | | P62306 |  |
| SNRPG | Small nuclear ribonucleoprotein polypeptide G | | | P62308 |  |
| SNRPN | Small nuclear ribonucleoprotein polypeptide N | | | P63162 |  |
| SNU13 | Small nuclear ribonucleoprotein 13 | | | P55769 |  |
| SNUPN | Snurportin 1 | | | O95149 |  |
| SNURF | SNRPN upstream open reading frame | | | Q9Y675 |  |
| SNW1 | SNW domain containing 1 | | | Q13573 |  |
| SNX1 | Sorting nexin 1 | | | Q13596 |  |
| SNX10 | Sorting nexin 10 | | | Q9Y5X0 |  |
| SNX11 | Sorting nexin 11 | | | Q9Y5W9 |  |
| SNX12 | Sorting nexin 12 | | | Q9UMY4 |  |
| SNX15 | Sorting nexin 15 | | | Q9NRS6 |  |
| SNX16 | Sorting nexin 16 | | | P57768 |  |
| SNX17 | Sorting nexin 17 | | | Q15036 |  |
| SNX19 | Sorting nexin 19 | | | Q92543 |  |
| SNX2 | Sorting nexin 2 | | | O60749 |  |
| SNX20 | Sorting nexin 20 | | | Q7Z614 |  |
| SNX29 | Sorting nexin 29 | | | Q8TEQ0 |  |
| SNX3 | Sorting nexin 3 | | | O60493 |  |
| SNX4 | Sorting nexin 4 | | | O95219 |  |
| SNX5 | Sorting nexin 5 | | | Q9Y5X3 |  |
| SNX6 | Sorting nexin 6 | | | Q9UNH7 |  |
| SNX8 | Sorting nexin 8 | | | Q9Y5X2 |  |
| SNX9 | Sorting nexin 9 | | | Q9Y5X1 |  |
| SOAT1 | Sterol O-acyltransferase 1 | | | P35610 |  |
| SOCS1 | Suppressor of cytokine signaling 1 | | | O15524 |  |
| SOCS2 | Suppressor of cytokine signaling 2 | | | O14508 |  |
| SOCS3 | Suppressor of cytokine signaling 3 | | | O14543 |  |
| SOCS4 | Suppressor of cytokine signaling 4 | | | Q8WXH5 |  |
| SOCS5 | Suppressor of cytokine signaling 5 | | | O75159 |  |
| SOD1 | Superoxide dismutase 1 | | | P00441 |  |
| SOD2 | Superoxide dismutase 2 | | | P04179 |  |
| SORBS3 | Sorbin and SH3 domain containing 3 | | | O60504 |  |
| SORD | Sorbitol dehydrogenase | | | Q00796 |  |
| SOS1 | SOS Ras/Rac guanine nucleotide exchange factor 1 | | | Q07889 |  |
| SOX4 | SRY-box transcription factor 4 | | | Q06945 |  |
| SP1 | Sp1 transcription factor | | | P08047 |  |
| SP100 | SP100 nuclear antigen | | | P23497 |  |
| SP110 | SP110 nuclear body protein | | | Q9HB58 |  |
| SP140 | SP140 nuclear body protein | | | Q13342 |  |
| SP140L | SP140 nuclear body protein like | | | Q9H930 |  |
| SP2 | Sp2 transcription factor | | | Q02086 |  |
| SP3 | Sp3 transcription factor | | | Q02447 |  |
| SP4 | Sp4 transcription factor | | | Q02446 |  |
| SPAG16 | Sperm associated antigen 16 | | | Q8N0X2 |  |
| SPAG7 | Sperm associated antigen 7 | | | O75391 |  |
| SPAST | Spastin | | | Q9UBP0 |  |
| SPATA2 | Spermatogenesis associated 2 | | | Q9UM82 |  |
| SPATA20 | Spermatogenesis associated 20 | | | Q8TB22 |  |
| SPATA24 | Spermatogenesis associated 24 | | | Q86W54 |  |
| SPATA2L | Spermatogenesis associated 2 like | | | Q8IUW3 |  |
| SPATA33 | Spermatogenesis associated 33 | | | Q96N06 |  |
| SPATA7 | Spermatogenesis associated 7 | | | Q9P0W8 |  |
| SPATC1L | Spermatogenesis and centriole associated 1 like | | | Q9H0A9 |  |
| SPATS2 | Spermatogenesis associated serine rich 2 | | | Q86XZ4 |  |
| SPATS2L | Spermatogenesis associated serine rich 2 like | | | Q9NUQ6 |  |
| SPCS1 | Signal peptidase complex subunit 1 | | | Q9Y6A9 |  |
| SPCS2 | Signal peptidase complex subunit 2 | | | Q15005 |  |
| SPCS3 | Signal peptidase complex subunit 3 | | | P61009 |  |
| SPDL1 | Spindle apparatus coiled-coil protein 1 | | | Q96EA4 |  |
| SPECC1 | Sperm antigen with calponin homology and coiled-coil domains 1 | | | Q5M775 |  |
| SPECC1L | Sperm antigen with calponin homology and coiled-coil domains 1 like | | | Q69YQ0 |  |
| SPEF2 | Sperm flagellar 2 | | | Q9C093 |  |
| SPG21 | SPG21 abhydrolase domain containing, maspardin | | | Q9NZD8 |  |
| SPG7 | SPG7 matrix AAA peptidase subunit, paraplegin | | | Q9UQ90 |  |
| SPHK2 | Sphingosine kinase 2 | | | Q9NRA0 |  |
| SPICE1 | Spindle and centriole associated protein 1 | | | Q8N0Z3 |  |
| SPIDR | Scaffold protein involved in DNA repair | | | Q14159 |  |
| SPIN1 | Spindlin 1 | | | Q9Y657 |  |
| SPIN2B | Spindlin family member 2B | | | Q9BPZ2 |  |
| SPIN3 | Spindlin family member 3 | | | Q5JUX0 |  |
| SPINK2 | Serine peptidase inhibitor Kazal type 2 | | | P20155 |  |
| SPINT1 | Serine peptidase inhibitor, Kunitz type 1 | | | O43278 |  |
| SPINT2 | Serine peptidase inhibitor, Kunitz type 2 | | | O43291 |  |
| SPN | Sialophorin | | | P16150 |  |
| SPNS1 | Sphingolipid transporter 1 (putative) | | | Q9H2V7 |  |
| SPOCK2 | SPARC (osteonectin), cwcv and kazal like domains proteoglycan 2 | | | Q92563 |  |
| SPON2 | Spondin 2 | | | Q9BUD6 |  |
| SPOP | Speckle type BTB/POZ protein | | | O43791 |  |
| SPPL2B | Signal peptide peptidase like 2B | | | Q8TCT7 |  |
| SPPL3 | Signal peptide peptidase like 3 | | | Q8TCT6 |  |
| SPRTN | SprT-like N-terminal domain | | | Q9H040 |  |
| SPRY1 | Sprouty RTK signaling antagonist 1 | | | O43609 |  |
| SPRYD7 | SPRY domain containing 7 | | | Q5W111 |  |
| SPSB2 | SplA/ryanodine receptor domain and SOCS box containing 2 | | | Q99619 |  |
| SPSB3 | SplA/ryanodine receptor domain and SOCS box containing 3 | | | Q6PJ21 |  |
| SPTLC1 | Serine palmitoyltransferase long chain base subunit 1 | | | O15269 |  |
| SPTLC2 | Serine palmitoyltransferase long chain base subunit 2 | | | O15270 |  |
| SPTSSA | Serine palmitoyltransferase small subunit A | | | Q969W0 |  |
| SPTY2D1 | SPT2 chromatin protein domain containing 1 | | | Q68D10 |  |
| SQLE | Squalene epoxidase | | | Q14534 |  |
| SQSTM1 | Sequestosome 1 | | | Q13501 |  |
| SRA1 | Steroid receptor RNA activator 1 | | | Q9HD15 |  |
| SRBD1 | S1 RNA binding domain 1 | | | Q8N5C6 |  |
| SRD5A3 | Steroid 5 alpha-reductase 3 | | | Q9H8P0 |  |
| SREBF2 | Sterol regulatory element binding transcription factor 2 | | | Q12772 |  |
| SREK1 | Splicing regulatory glutamic acid and lysine rich protein 1 | | | Q8WXA9 |  |
| SREK1IP1 | SREK1 interacting protein 1 | | | Q8N9Q2 |  |
| SRFBP1 | Serum response factor binding protein 1 | | | Q8NEF9 |  |
| SRGN | Serglycin | | | P10124 |  |
| SRI | Sorcin | | | P30626 |  |
| SRM | Spermidine synthase | | | P19623 |  |
| SRP14 | Signal recognition particle 14 | | | P37108 |  |
| SRP19 | Signal recognition particle 19 | | | P09132 |  |
| SRP54 | Signal recognition particle 54 | | | P61011 |  |
| SRP68 | Signal recognition particle 68 | | | Q9UHB9 |  |
| SRP72 | Signal recognition particle 72 | | | O76094 |  |
| SRP9 | Signal recognition particle 9 | | | P49458 |  |
| SRPK1 | SRSF protein kinase 1 | | | Q96SB4 |  |
| SRPK2 | SRSF protein kinase 2 | | | P78362 |  |
| SRPRA | SRP receptor subunit alpha | | | P08240 |  |
| SRPRB | SRP receptor subunit beta | | | Q9Y5M8 |  |
| SRR | Serine racemase | | | Q9GZT4 |  |
| SRRD | SRR1 domain containing | | | Q9UH36 |  |
| SRRM1 | Serine and arginine repetitive matrix 1 | | | Q8IYB3 |  |
| SRRT | Serrate, RNA effector molecule | | | Q9BXP5 |  |
| SRSF1 | Serine and arginine rich splicing factor 1 | | | Q07955 |  |
| SRSF10 | Serine and arginine rich splicing factor 10 | | | O75494 |  |
| SRSF11 | Serine and arginine rich splicing factor 11 | | | Q05519 |  |
| SRSF2 | Serine and arginine rich splicing factor 2 | | | Q01130 |  |
| SRSF3 | Serine and arginine rich splicing factor 3 | | | P84103 |  |
| SRSF4 | Serine and arginine rich splicing factor 4 | | | Q08170 |  |
| SRSF5 | Serine and arginine rich splicing factor 5 | | | Q13243 |  |
| SRSF6 | Serine and arginine rich splicing factor 6 | | | Q13247 |  |
| SRSF7 | Serine and arginine rich splicing factor 7 | | | Q16629 |  |
| SRSF8 | Serine and arginine rich splicing factor 8 | | | Q9BRL6 |  |
| SRSF9 | Serine and arginine rich splicing factor 9 | | | Q13242 |  |
| SRXN1 | Sulfiredoxin 1 | | | Q9BYN0 |  |
| SS18 | SS18 subunit of BAF chromatin remodeling complex | | | Q15532 |  |
| SS18L2 | SS18 like 2 | | | Q9UHA2 |  |
| SSB | Small RNA binding exonuclease protection factor La | | | P05455 |  |
| SSBP1 | Single stranded DNA binding protein 1 | | | Q04837 |  |
| SSBP2 | Single stranded DNA binding protein 2 | | | P81877 |  |
| SSBP3 | Single stranded DNA binding protein 3 | | | Q9BWW4 |  |
| SSBP4 | Single stranded DNA binding protein 4 | | | Q9BWG4 |  |
| SSH2 | Slingshot protein phosphatase 2 | | | Q76I76 |  |
| SSH3 | Slingshot protein phosphatase 3 | | | Q8TE77 |  |
| SSNA1 | SS nuclear autoantigen 1 | | | O43805 |  |
| SSR1 | Signal sequence receptor subunit 1 | | | P43307 |  |
| SSR2 | Signal sequence receptor subunit 2 | | | P43308 |  |
| SSR3 | Signal sequence receptor subunit 3 | | | Q9UNL2 |  |
| SSR4 | Signal sequence receptor subunit 4 | | | P51571 |  |
| SSRP1 | Structure specific recognition protein 1 | | | Q08945 |  |
| SSTR3 | Somatostatin receptor 3 | | | P32745 |  |
| SSU72 | SSU72 homolog, RNA polymerase II CTD phosphatase | | | Q9NP77 |  |
| SSX2IP | SSX family member 2 interacting protein | | | Q9Y2D8 |  |
| ST3GAL1 | ST3 beta-galactoside alpha-2,3-sialyltransferase 1 | | | Q11201 |  |
| ST3GAL2 | ST3 beta-galactoside alpha-2,3-sialyltransferase 2 | | | Q16842 |  |
| ST3GAL3 | ST3 beta-galactoside alpha-2,3-sialyltransferase 3 | | | Q11203 |  |
| ST3GAL4 | ST3 beta-galactoside alpha-2,3-sialyltransferase 4 | | | Q11206 |  |
| ST6GAL1 | ST6 beta-galactoside alpha-2,6-sialyltransferase 1 | | | P15907 |  |
| ST6GALNAC1 | ST6 N-acetylgalactosaminide alpha-2,6-sialyltransferase 1 | | | Q9NSC7 |  |
| ST6GALNAC3 | ST6 N-acetylgalactosaminide alpha-2,6-sialyltransferase 3 | | | Q8NDV1 |  |
| ST6GALNAC4 | ST6 N-acetylgalactosaminide alpha-2,6-sialyltransferase 4 | | | Q9H4F1 |  |
| ST6GALNAC6 | ST6 N-acetylgalactosaminide alpha-2,6-sialyltransferase 6 | | | Q969X2 |  |
| ST7 | Suppression of tumorigenicity 7 | | | Q9NRC1 |  |
| ST7L | Suppression of tumorigenicity 7 like | | | Q8TDW4 |  |
| ST8SIA4 | ST8 alpha-N-acetyl-neuraminide alpha-2,8-sialyltransferase 4 | | | Q92187 |  |
| STAG2 | Stromal antigen 2 | | | Q8N3U4 |  |
| STAM | Signal transducing adaptor molecule | | | Q92783 |  |
| STAM2 | Signal transducing adaptor molecule 2 | | | O75886 |  |
| STAMBP | STAM binding protein | | | O95630 |  |
| STAP1 | Signal transducing adaptor family member 1 | | | Q9ULZ2 |  |
| STAP2 | Signal transducing adaptor family member 2 | | | Q9UGK3 |  |
| STARD10 | StAR related lipid transfer domain containing 10 | | | Q9Y365 |  |
| STARD3 | StAR related lipid transfer domain containing 3 | | | Q14849 |  |
| STARD3NL | STARD3 N-terminal like | | | O95772 |  |
| STARD4 | StAR related lipid transfer domain containing 4 | | | Q96DR4 |  |
| STARD5 | StAR related lipid transfer domain containing 5 | | | Q9NSY2 |  |
| STARD7 | StAR related lipid transfer domain containing 7 | | | Q9NQZ5 |  |
| STAT1 | Signal transducer and activator of transcription 1 | | | P42224 |  |
| STAT2 | Signal transducer and activator of transcription 2 | | | P52630 |  |
| STAT3 | Signal transducer and activator of transcription 3 | | | P40763 |  |
| STAT4 | Signal transducer and activator of transcription 4 | | | Q14765 |  |
| STAT5A | Signal transducer and activator of transcription 5A | | | P42229 |  |
| STAT5B | Signal transducer and activator of transcription 5B | | | P51692 |  |
| STAT6 | Signal transducer and activator of transcription 6 | | | P42226 |  |
| STAU1 | Staufen double-stranded RNA binding protein 1 | | | O95793 |  |
| STAU2 | Staufen double-stranded RNA binding protein 2 | | | Q9NUL3 |  |
| STBD1 | Starch binding domain 1 | | | O95210 |  |
| STIM1 | Stromal interaction molecule 1 | | | Q13586 |  |
| STIP1 | Stress induced phosphoprotein 1 | | | P31948 |  |
| STK11IP | Serine/threonine kinase 11 interacting protein | | | Q8N1F8 |  |
| STK16 | Serine/threonine kinase 16 | | | O75716 |  |
| STK17A | Serine/threonine kinase 17a | | | Q9UEE5 |  |
| STK17B | Serine/threonine kinase 17b | | | O94768 |  |
| STK19 | Serine/threonine kinase 19 | | | P49842 |  |
| STK24 | Serine/threonine kinase 24 | | | Q9Y6E0 |  |
| STK25 | Serine/threonine kinase 25 | | | O00506 |  |
| STK26 | Serine/threonine kinase 26 | | | Q9P289 |  |
| STK3 | Serine/threonine kinase 3 | | | Q13188 |  |
| STK32C | Serine/threonine kinase 32C | | | Q86UX6 |  |
| STK38 | Serine/threonine kinase 38 | | | Q15208 |  |
| STK38L | Serine/threonine kinase 38 like | | | Q9Y2H1 |  |
| STK40 | Serine/threonine kinase 40 | | | Q8N2I9 |  |
| STMN1 | Stathmin 1 | | | P16949 |  |
| STMN3 | Stathmin 3 | | | Q9NZ72 |  |
| STOM | Stomatin | | | P27105 |  |
| STOML1 | Stomatin like 1 | | | Q9UBI4 |  |
| STOML2 | Stomatin like 2 | | | Q9UJZ1 |  |
| STRADA | STE20 related adaptor alpha | | | Q7RTN6 |  |
| STRADB | STE20 related adaptor beta | | | Q9C0K7 |  |
| STRAP | Serine/threonine kinase receptor associated protein | | | Q9Y3F4 |  |
| STRBP | Spermatid perinuclear RNA binding protein | | | Q96SI9 |  |
| STRIP1 | Striatin interacting protein 1 | | | Q5VSL9 |  |
| STT3A | STT3 oligosaccharyltransferase complex catalytic subunit A | | | P46977 |  |
| STT3B | STT3 oligosaccharyltransferase complex catalytic subunit B | | | Q8TCJ2 |  |
| STUB1 | STIP1 homology and U-box containing protein 1 | | | Q9UNE7 |  |
| STX10 | Syntaxin 10 | | | O60499 |  |
| STX11 | Syntaxin 11 | | | O75558 |  |
| STX12 | Syntaxin 12 | | | Q86Y82 |  |
| STX16 | Syntaxin 16 | | | O14662 |  |
| STX17 | Syntaxin 17 | | | P56962 |  |
| STX18 | Syntaxin 18 | | | Q9P2W9 |  |
| STX3 | Syntaxin 3 | | | Q13277 |  |
| STX4 | Syntaxin 4 | | | Q12846 |  |
| STX5 | Syntaxin 5 | | | Q13190 |  |
| STX6 | Syntaxin 6 | | | O43752 |  |
| STX7 | Syntaxin 7 | | | O15400 |  |
| STX8 | Syntaxin 8 | | | Q9UNK0 |  |
| STXBP2 | Syntaxin binding protein 2 | | | Q15833 |  |
| STXBP3 | Syntaxin binding protein 3 | | | O00186 |  |
| STYX | Serine/threonine/tyrosine interacting protein | | | Q8WUJ0 |  |
| STYXL1 | Serine/threonine/tyrosine interacting like 1 | | | Q9Y6J8 |  |
| SUB1 | SUB1 regulator of transcription | | | P53999 |  |
| SUCLG1 | Succinate-CoA ligase GDP/ADP-forming subunit alpha | | | P53597 |  |
| SUCLG2 | Succinate-CoA ligase GDP-forming subunit beta | | | Q96I99 |  |
| SUDS3 | SDS3 homolog, SIN3A corepressor complex component | | | Q9H7L9 |  |
| SUFU | SUFU negative regulator of hedgehog signaling | | | Q9UMX1 |  |
| SUGCT | Succinyl-CoA:glutarate-CoA transferase | | | Q9HAC7 |  |
| SUGP1 | SURP and G-patch domain containing 1 | | | Q8IWZ8 |  |
| SUGP2 | SURP and G-patch domain containing 2 | | | Q8IX01 |  |
| SUGT1 | SGT1 homolog, MIS12 kinetochore complex assembly cochaperone | | | Q9Y2Z0 |  |
| SULT1A1 | Sulfotransferase family 1A member 1 | | | P50225 |  |
| SULT1A3 | Sulfotransferase family 1A member 3 | | | P0DMM9 |  |
| SULT1B1 | Sulfotransferase family 1B member 1 | | | O43704 |  |
| SUMF1 | Sulfatase modifying factor 1 | | | Q8NBK3 |  |
| SUMF2 | Sulfatase modifying factor 2 | | | Q8NBJ7 |  |
| SUMO1 | Small ubiquitin like modifier 1 | | | P63165 |  |
| SUMO2 | Small ubiquitin like modifier 2 | | | P61956 |  |
| SUMO3 | Small ubiquitin like modifier 3 | | | P55854 |  |
| SUMO4 | Small ubiquitin like modifier 4 | | | Q6EEV6 |  |
| SUN1 | Sad1 and UNC84 domain containing 1 | | | O94901 |  |
| SUN2 | Sad1 and UNC84 domain containing 2 | | | Q9UH99 |  |
| SUOX | Sulfite oxidase | | | P51687 |  |
| SUPT20H | SPT20 homolog, SAGA complex component | | | Q8NEM7 |  |
| SUPT3H | SPT3 homolog, SAGA and STAGA complex component | | | O75486 |  |
| SUPT4H1 | SPT4 homolog, DSIF elongation factor subunit | | | P63272 |  |
| SUPT5H | SPT5 homolog, DSIF elongation factor subunit | | | O00267 |  |
| SUPT6H | SPT6 homolog, histone chaperone and transcription elongation factor | | | Q7KZ85 |  |
| SUPV3L1 | Suv3 like RNA helicase | | | Q8IYB8 |  |
| SURF1 | SURF1 cytochrome c oxidase assembly factor | | | Q15526 |  |
| SURF2 | Surfeit 2 | | | Q15527 |  |
| SURF4 | Surfeit 4 | | | O15260 |  |
| SURF6 | Surfeit 6 | | | O75683 |  |
| SUSD3 | Sushi domain containing 3 | | | Q96L08 |  |
| SUSD4 | Sushi domain containing 4 | | | Q5VX71 |  |
| SUSD6 | Sushi domain containing 6 | | | Q92537 |  |
| SUV39H1 | SUV39H1 histone lysine methyltransferase | | | O43463 |  |
| SUV39H2 | SUV39H2 histone lysine methyltransferase | | | Q9H5I1 |  |
| SVBP | Small vasohibin binding protein | | | Q8N300 |  |
| SVIL | Supervillin | | | O95425 |  |
| SWAP70 | Switching B cell complex subunit SWAP70 | | | Q9UH65 |  |
| SWSAP1 | SWIM-type zinc finger 7 associated protein 1 | | | Q6NVH7 |  |
| SWT1 | SWT1 RNA endoribonuclease homolog | | | Q5T5J6 |  |
| SYAP1 | Synapse associated protein 1 | | | Q96A49 |  |
| SYF2 | SYF2 pre-mRNA splicing factor | | | O95926 |  |
| SYMPK | Symplekin scaffold protein | | | Q92797 |  |
| SYNCRIP | Synaptotagmin binding cytoplasmic RNA interacting protein | | | O60506 |  |
| SYNE2 | Spectrin repeat containing nuclear envelope protein 2 | | | Q8WXH0 |  |
| SYNGR1 | Synaptogyrin 1 | | | O43759 |  |
| SYNGR2 | Synaptogyrin 2 | | | O43760 |  |
| SYNJ2 | Synaptojanin 2 | | | O15056 |  |
| SYNJ2BP | Synaptojanin 2 binding protein | | | P57105 |  |
| SYPL1 | Synaptophysin like 1 | | | Q16563 |  |
| SYS1 | SYS1 golgi trafficking protein | | | Q8N2H4 |  |
| SYT11 | Synaptotagmin 11 | | | Q9BT88 |  |
| SYTL1 | Synaptotagmin like 1 | | | Q8IYJ3 |  |
| SYTL2 | Synaptotagmin like 2 | | | Q9HCH5 |  |
| SYTL3 | Synaptotagmin like 3 | | | Q4VX76 |  |
| SYVN1 | Synoviolin 1 | | | Q86TM6 |  |
| SZRD1 | SUZ RNA binding domain containing 1 | | | Q7Z422 |  |
| TAB1 | TGF-beta activated kinase 1 (MAP3K7) binding protein 1 | | | Q15750 |  |
| TAB2 | TGF-beta activated kinase 1 (MAP3K7) binding protein 2 | | | Q9NYJ8 |  |
| TACC1 | Transforming acidic coiled-coil containing protein 1 | | | O75410 |  |
| TACC3 | Transforming acidic coiled-coil containing protein 3 | | | Q9Y6A5 |  |
| TACO1 | Translational activator of cytochrome c oxidase I | | | Q9BSH4 |  |
| TADA1 | Transcriptional adaptor 1 | | | Q96BN2 |  |
| TADA2A | Transcriptional adaptor 2A | | |  |  |
| TADA2B | Transcriptional adaptor 2B | | | Q86TJ2 |  |
| TADA3 | Transcriptional adaptor 3 | | | O75528 |  |
| TAF1 | TATA-box binding protein associated factor 1 | | | P21675 |  |
| TAF10 | TATA-box binding protein associated factor 10 | | | Q12962 |  |
| TAF11 | TATA-box binding protein associated factor 11 | | | Q15544 |  |
| TAF12 | TATA-box binding protein associated factor 12 | | | Q16514 |  |
| TAF13 | TATA-box binding protein associated factor 13 | | | Q15543 |  |
| TAF1A | TATA-box binding protein associated factor, RNA polymerase I subunit A | | | Q15573 |  |
| TAF1B | TATA-box binding protein associated factor, RNA polymerase I subunit B | | | Q53T94 |  |
| TAF1D | TATA-box binding protein associated factor, RNA polymerase I subunit D | | | Q9H5J8 |  |
| TAF2 | TATA-box binding protein associated factor 2 | | | Q6P1X5 |  |
| TAF3 | TATA-box binding protein associated factor 3 | | | Q5VWG9 |  |
| TAF6 | TATA-box binding protein associated factor 6 | | | P49848 |  |
| TAF7 | TATA-box binding protein associated factor 7 | | | Q15545 |  |
| TAF8 | TATA-box binding protein associated factor 8 | | | Q7Z7C8 |  |
| TAF9 | TATA-box binding protein associated factor 9 | | | Q16594 |  |
| TAF9B | TATA-box binding protein associated factor 9b | | | Q9HBM6 |  |
| TAGAP | T cell activation RhoGTPase activating protein | | | Q8N103 |  |
| TAGLN2 | Transgelin 2 | | | P37802 |  |
| TALDO1 | Transaldolase 1 | | | P37837 |  |
| TAMM41 | TAM41 mitochondrial translocator assembly and maintenance homolog | | | Q96BW9 |  |
| TANGO2 | Transport and golgi organization 2 homolog | | | Q6ICL3 |  |
| TANK | TRAF family member associated NFKB activator | | | Q92844 |  |
| TAOK3 | TAO kinase 3 | | | Q9H2K8 |  |
| TAP1 | Transporter 1, ATP binding cassette subfamily B member | | | Q03518 |  |
| TAP2 | Transporter 2, ATP binding cassette subfamily B member | | | Q03519 |  |
| TAPBP | TAP binding protein | | | O15533 |  |
| TAPBPL | TAP binding protein like | | | Q9BX59 |  |
| TARBP2 | TARBP2 subunit of RISC loading complex | | | Q15633 |  |
| TARDBP | TAR DNA binding protein | | | Q13148 |  |
| TARP | TCR gamma alternate reading frame protein | | |  |  |
| TASP1 | Taspase 1 | | | Q9H6P5 |  |
| TATDN1 | TatD DNase domain containing 1 | | | Q6P1N9 |  |
| TATDN3 | TatD DNase domain containing 3 | | | Q17R31 |  |
| TAX1BP1 | Tax1 binding protein 1 | | | Q86VP1 |  |
| TAX1BP3 | Tax1 binding protein 3 | | | O14907 |  |
| TBC1D10A | TBC1 domain family member 10A | | | Q9BXI6 |  |
| TBC1D10C | TBC1 domain family member 10C | | | Q8IV04 |  |
| TBC1D13 | TBC1 domain family member 13 | | | Q9NVG8 |  |
| TBC1D15 | TBC1 domain family member 15 | | | Q8TC07 |  |
| TBC1D19 | TBC1 domain family member 19 | | | Q8N5T2 |  |
| TBC1D2 | TBC1 domain family member 2 | | | Q9BYX2 |  |
| TBC1D20 | TBC1 domain family member 20 | | | Q96BZ9 |  |
| TBC1D22A | TBC1 domain family member 22A | | | Q8WUA7 |  |
| TBC1D22B | TBC1 domain family member 22B | | | Q9NU19 |  |
| TBC1D23 | TBC1 domain family member 23 | | | Q9NUY8 |  |
| TBC1D25 | TBC1 domain family member 25 | | | Q3MII6 |  |
| TBC1D4 | TBC1 domain family member 4 | | | O60343 |  |
| TBC1D5 | TBC1 domain family member 5 | | | Q92609 |  |
| TBC1D7 | TBC1 domain family member 7 | | | Q9P0N9 |  |
| TBC1D9B | TBC1 domain family member 9B | | | Q66K14 |  |
| TBCA | Tubulin folding cofactor A | | | O75347 |  |
| TBCB | Tubulin folding cofactor B | | | Q99426 |  |
| TBCC | Tubulin folding cofactor C | | | Q15814 |  |
| TBCCD1 | TBCC domain containing 1 | | | Q9NVR7 |  |
| TBCD | Tubulin folding cofactor D | | | Q9BTW9 |  |
| TBCE | Tubulin folding cofactor E | | | Q15813 |  |
| TBCK | TBC1 domain containing kinase | | | Q8TEA7 |  |
| TBK1 | TANK binding kinase 1 | | | Q9UHD2 |  |
| TBL1X | Transducin beta like 1 X-linked | | | O60907 |  |
| TBL1XR1 | TBL1X/Y related 1 | | | Q9BZK7 |  |
| TBL2 | Transducin beta like 2 | | | Q9Y4P3 |  |
| TBL3 | Transducin beta like 3 | | | Q12788 |  |
| TBP | TATA-box binding protein | | | P20226 |  |
| TBPL1 | TATA-box binding protein like 1 | | | P62380 |  |
| TBRG1 | Transforming growth factor beta regulator 1 | | | Q3YBR2 |  |
| TBRG4 | Transforming growth factor beta regulator 4 | | | Q969Z0 |  |
| TBX21 | T-box transcription factor 21 | | | Q9UL17 |  |
| TBXAS1 | Thromboxane A synthase 1 | | | P24557 |  |
| TC2N | Tandem C2 domains, nuclear | | | Q8N9U0 |  |
| TCAF1 | TRPM8 channel associated factor 1 | | | Q9Y4C2 |  |
| TCAF2 | TRPM8 channel associated factor 2 | | | A6NFQ2 |  |
| TCAIM | T cell activation inhibitor, mitochondrial | | | Q8N3R3 |  |
| TCEA1 | Transcription elongation factor A1 | | | P23193 |  |
| TCEA2 | Transcription elongation factor A2 | | | Q15560 |  |
| TCEA3 | Transcription elongation factor A3 | | | O75764 |  |
| TCEAL1 | Transcription elongation factor A like 1 | | | Q15170 |  |
| TCEAL3 | Transcription elongation factor A like 3 | | | Q969E4 |  |
| TCEAL4 | Transcription elongation factor A like 4 | | | Q96EI5 |  |
| TCEAL8 | Transcription elongation factor A like 8 | | | Q8IYN2 |  |
| TCEANC | Transcription elongation factor A N-terminal and central domain containing | | | Q8N8B7 |  |
| TCEANC2 | Transcription elongation factor A N-terminal and central domain containing 2 | | | Q96MN5 |  |
| TCF12 | Transcription factor 12 | | | Q99081 |  |
| TCF19 | Transcription factor 19 | | | Q9Y242 |  |
| TCF25 | Transcription factor 25 | | | Q9BQ70 |  |
| TCF3 | Transcription factor 3 | | | P15923 |  |
| TCF7 | Transcription factor 7 | | | P36402 |  |
| TCHP | Trichoplein keratin filament binding | | | Q9BT92 |  |
| TCIRG1 | T cell immune regulator 1, ATPase H+ transporting V0 subunit a3 | | | Q13488 |  |
| TCL1A | TCL1 family AKT coactivator A | | | P56279 |  |
| TCOF1 | Treacle ribosome biogenesis factor 1 | | | Q13428 |  |
| TCP1 | T-complex 1 | | | P17987 |  |
| TCP11L1 | T-complex 11 like 1 | | | Q9NUJ3 |  |
| TCP11L2 | T-complex 11 like 2 | | | Q8N4U5 |  |
| TCTA | T cell leukemia translocation altered | | | P57738 |  |
| TCTN1 | Tectonic family member 1 | | | Q2MV58 |  |
| TCTN2 | Tectonic family member 2 | | | Q96GX1 |  |
| TCTN3 | Tectonic family member 3 | | | Q6NUS6 |  |
| TDG | Thymine DNA glycosylase | | | Q13569 |  |
| TDP1 | Tyrosyl-DNA phosphodiesterase 1 | | | Q9NUW8 |  |
| TDP2 | Tyrosyl-DNA phosphodiesterase 2 | | | O95551 |  |
| TDRD3 | Tudor domain containing 3 | | | Q9H7E2 |  |
| TDRD7 | Tudor domain containing 7 | | | Q8NHU6 |  |
| TDRKH | Tudor and KH domain containing | | | Q9Y2W6 |  |
| TECPR1 | Tectonin beta-propeller repeat containing 1 | | | Q7Z6L1 |  |
| TECR | Trans-2,3-enoyl-CoA reductase | | | Q9NZ01 |  |
| TEFM | Transcription elongation factor, mitochondrial | | | Q96QE5 |  |
| TELO2 | Telomere maintenance 2 | | | Q9Y4R8 |  |
| TEN1 | TEN1 subunit of CST complex | | | Q86WV5 |  |
| TERF1 | Telomeric repeat binding factor 1 | | | P54274 |  |
| TERF2 | Telomeric repeat binding factor 2 | | | Q15554 |  |
| TERF2IP | TERF2 interacting protein | | | Q9NYB0 |  |
| TES | Testin LIM domain protein | | | Q9UGI8 |  |
| TESC | Tescalcin | | | Q96BS2 |  |
| TESK2 | Testis associated actin remodelling kinase 2 | | | Q96S53 |  |
| TESPA1 | Thymocyte expressed, positive selection associated 1 | | | A2RU30 |  |
| TEX10 | Testis expressed 10 | | | Q9NXF1 |  |
| TEX2 | Testis expressed 2 | | | Q8IWB9 |  |
| TEX261 | Testis expressed 261 | | | Q6UWH6 |  |
| TEX264 | Testis expressed 264, ER-phagy receptor | | | Q9Y6I9 |  |
| TEX30 | Testis expressed 30 | | | Q5JUR7 |  |
| TFAM | Transcription factor A, mitochondrial | | | Q00059 |  |
| TFB1M | Transcription factor B1, mitochondrial | | | Q8WVM0 |  |
| TFB2M | Transcription factor B2, mitochondrial | | | Q9H5Q4 |  |
| TFCP2 | Transcription factor CP2 | | | Q12800 |  |
| TFDP1 | Transcription factor Dp-1 | | | Q14186 |  |
| TFDP2 | Transcription factor Dp-2 | | | Q14188 |  |
| TFEB | Transcription factor EB | | | P19484 |  |
| TFG | Trafficking from ER to golgi regulator | | | Q92734 |  |
| TFIP11 | Tuftelin interacting protein 11 | | | Q9UBB9 |  |
| TFPT | TCF3 fusion partner | | | P0C1Z6 |  |
| TFRC | Transferrin receptor | | | P02786 |  |
| TGDS | TDP-glucose 4,6-dehydratase | | | O95455 |  |
| TGFA | Transforming growth factor alpha | | | P01135 |  |
| TGFBI | Transforming growth factor beta induced | | | Q15582 |  |
| TGFBR1 | Transforming growth factor beta receptor 1 | | | P36897 |  |
| TGFBR2 | Transforming growth factor beta receptor 2 | | | P37173 |  |
| TGFBR3 | Transforming growth factor beta receptor 3 | | | Q03167 |  |
| TGFBR3 | Transforming growth factor beta receptor 3 | | | Q03167 |  |
| TGFBRAP1 | Transforming growth factor beta receptor associated protein 1 | | | Q8WUH2 |  |
| TGIF1 | TGFB induced factor homeobox 1 | | | Q15583 |  |
| TGIF2 | TGFB induced factor homeobox 2 | | | Q9GZN2 |  |
| TGOLN2 | Trans-golgi network protein 2 | | | O43493 |  |
| TGS1 | Trimethylguanosine synthase 1 | | | Q96RS0 |  |
| THADA | THADA armadillo repeat containing | | | Q6YHU6 |  |
| THAP1 | THAP domain containing 1 | | | Q9NVV9 |  |
| THAP11 | THAP domain containing 11 | | | Q96EK4 |  |
| THAP2 | THAP domain containing 2 | | | Q9H0W7 |  |
| THAP3 | THAP domain containing 3 | | | Q8WTV1 |  |
| THAP4 | THAP domain containing 4 | | | Q8WY91 |  |
| THAP5 | THAP domain containing 5 | | | Q7Z6K1 |  |
| THAP6 | THAP domain containing 6 | | | Q8TBB0 |  |
| THAP7 | THAP domain containing 7 | | | Q9BT49 |  |
| THAP8 | THAP domain containing 8 | | | Q8NA92 |  |
| THAP9 | THAP domain containing 9 | | | Q9H5L6 |  |
| THBS3 | Thrombospondin 3 | | | P49746 |  |
| THEM4 | Thioesterase superfamily member 4 | | | Q5T1C6 |  |
| THEM6 | Thioesterase superfamily member 6 | | | Q8WUY1 |  |
| THEMIS | Thymocyte selection associated | | | Q8N1K5 |  |
| THEMIS2 | Thymocyte selection associated family member 2 | | | Q5TEJ8 |  |
| THG1L | TRNA-histidine guanylyltransferase 1 like | | | Q9NWX6 |  |
| THNSL1 | Threonine synthase like 1 | | | Q8IYQ7 |  |
| THOC1 | THO complex subunit 1 | | | Q96FV9 |  |
| THOC3 | THO complex subunit 3 | | | Q96J01 |  |
| THOC5 | THO complex subunit 5 | | | Q13769 |  |
| THOC6 | THO complex subunit 6 | | | Q86W42 |  |
| THOC7 | THO complex subunit 7 | | | Q6I9Y2 |  |
| THOP1 | Thimet oligopeptidase 1 | | | P52888 |  |
| THRA | Thyroid hormone receptor alpha | | | P10827 |  |
| THRAP3 | Thyroid hormone receptor associated protein 3 | | | Q9Y2W1 |  |
| THTPA | Thiamine triphosphatase | | | Q9BU02 |  |
| THUMPD1 | THUMP domain containing 1 | | | Q9NXG2 |  |
| THUMPD3 | THUMP domain containing 3 | | | Q9BV44 |  |
| THYN1 | Thymocyte nuclear protein 1 | | | Q9P016 |  |
| TIA1 | TIA1 cytotoxic granule associated RNA binding protein | | | P31483 |  |
| TIAL1 | TIA1 cytotoxic granule associated RNA binding protein like 1 | | | Q01085 |  |
| TIFA | TRAF interacting protein with forkhead associated domain | | | Q96CG3 |  |
| TIGD1 | Tigger transposable element derived 1 | | | Q96MW7 |  |
| TIGD6 | Tigger transposable element derived 6 | | | Q17RP2 |  |
| TIGD7 | Tigger transposable element derived 7 | | | Q6NT04 |  |
| TIGIT | T cell immunoreceptor with Ig and ITIM domains | | | Q495A1 |  |
| TIMD4 | T cell immunoglobulin and mucin domain containing 4 | | | Q96H15 |  |
| TIMELESS | Timeless circadian regulator | | | Q9UNS1 |  |
| TIMM10 | Translocase of inner mitochondrial membrane 10 | | | P62072 |  |
| TIMM10B | Translocase of inner mitochondrial membrane 10B | | | Q9Y5J6 |  |
| TIMM13 | Translocase of inner mitochondrial membrane 13 | | | Q9Y5L4 |  |
| TIMM17A | Translocase of inner mitochondrial membrane 17A | | | Q99595 |  |
| TIMM17B | Translocase of inner mitochondrial membrane 17B | | | O60830 |  |
| TIMM21 | Translocase of inner mitochondrial membrane 21 | | | Q9BVV7 |  |
| TIMM23 | Translocase of inner mitochondrial membrane 23 | | | O14925 |  |
| TIMM44 | Translocase of inner mitochondrial membrane 44 | | | O43615 |  |
| TIMM50 | Translocase of inner mitochondrial membrane 50 | | | Q3ZCQ8 |  |
| TIMM8A | Translocase of inner mitochondrial membrane 8A | | | O60220 |  |
| TIMM8B | Translocase of inner mitochondrial membrane 8 homolog B | | | Q9Y5J9 |  |
| TIMM9 | Translocase of inner mitochondrial membrane 9 | | | Q9Y5J7 |  |
| TIMMDC1 | Translocase of inner mitochondrial membrane domain containing 1 | | | Q9NPL8 |  |
| TIMP1 | TIMP metallopeptidase inhibitor 1 | | | P01033 |  |
| TINF2 | TERF1 interacting nuclear factor 2 | | | Q9BSI4 |  |
| TIPARP | TCDD inducible poly(ADP-ribose) polymerase | | | Q7Z3E1 |  |
| TIPIN | TIMELESS interacting protein | | | Q9BVW5 |  |
| TIPRL | TOR signaling pathway regulator | | | O75663 |  |
| TIRAP | TIR domain containing adaptor protein | | | P58753 |  |
| TK1 | Thymidine kinase 1 | | | P04183 |  |
| TK2 | Thymidine kinase 2 | | | O00142 |  |
| TKFC | Triokinase and FMN cyclase | | | Q3LXA3 |  |
| TKT | Transketolase | | | P29401 |  |
| TKTL1 | Transketolase like 1 | | | P51854 |  |
| TLE2 | TLE family member 2, transcriptional corepressor | | | Q04725 |  |
| TLE3 | TLE family member 3, transcriptional corepressor | | | Q04726 |  |
| TLE4 | TLE family member 4, transcriptional corepressor | | | Q04727 |  |
| TLK1 | Tousled like kinase 1 | | | Q9UKI8 |  |
| TLK2 | Tousled like kinase 2 | | | Q86UE8 |  |
| TLR1 | Toll like receptor 1 | | | Q15399 |  |
| TLR2 | Toll like receptor 2 | | | O60603 |  |
| TLR5 | Toll like receptor 5 | | | O60602 |  |
| TM2D1 | TM2 domain containing 1 | | | Q9BX74 |  |
| TM2D2 | TM2 domain containing 2 | | | Q9BX73 |  |
| TM2D3 | TM2 domain containing 3 | | | Q9BRN9 |  |
| TM4SF19 | Transmembrane 4 L six family member 19 | | | Q96DZ7 |  |
| TM7SF2 | Transmembrane 7 superfamily member 2 | | | O76062 |  |
| TM9SF1 | Transmembrane 9 superfamily member 1 | | | O15321 |  |
| TM9SF2 | Transmembrane 9 superfamily member 2 | | | Q99805 |  |
| TM9SF4 | Transmembrane 9 superfamily member 4 | | | Q92544 |  |
| TMA16 | Translation machinery associated 16 homolog | | | Q96EY4 |  |
| TMA7 | Translation machinery associated 7 homolog | | | Q9Y2S6 |  |
| TMBIM1 | Transmembrane BAX inhibitor motif containing 1 | | | Q969X1 |  |
| TMBIM4 | Transmembrane BAX inhibitor motif containing 4 | | | Q9HC24 |  |
| TMBIM6 | Transmembrane BAX inhibitor motif containing 6 | | | P55061 |  |
| TMC6 | Transmembrane channel like 6 | | | Q7Z403 |  |
| TMCC1 | Transmembrane and coiled-coil domain family 1 | | | O94876 |  |
| TMCC3 | Transmembrane and coiled-coil domain family 3 | | | Q9ULS5 |  |
| TMCO1 | Transmembrane and coiled-coil domains 1 | | | Q9UM00 |  |
| TMCO3 | Transmembrane and coiled-coil domains 3 | | | Q6UWJ1 |  |
| TMCO4 | Transmembrane and coiled-coil domains 4 | | | Q5TGY1 |  |
| TMCO6 | Transmembrane and coiled-coil domains 6 | | | Q96DC7 |  |
| TMED1 | Transmembrane p24 trafficking protein 1 | | | Q13445 |  |
| TMED10 | Transmembrane p24 trafficking protein 10 | | | P49755 |  |
| TMED2 | Transmembrane p24 trafficking protein 2 | | | Q15363 |  |
| TMED3 | Transmembrane p24 trafficking protein 3 | | | Q9Y3Q3 |  |
| TMED4 | Transmembrane p24 trafficking protein 4 | | | Q7Z7H5 |  |
| TMED5 | Transmembrane p24 trafficking protein 5 | | | Q9Y3A6 |  |
| TMED7 | Transmembrane p24 trafficking protein 7 | | | Q9Y3B3 |  |
| TMED9 | Transmembrane p24 trafficking protein 9 | | | Q9BVK6 |  |
| TMEM101 | Transmembrane protein 101 | | | Q96IK0 |  |
| TMEM104 | Transmembrane protein 104 | | | Q8NE00 |  |
| TMEM106A | Transmembrane protein 106A | | | Q96A25 |  |
| TMEM106B | Transmembrane protein 106B | | | Q9NUM4 |  |
| TMEM106C | Transmembrane protein 106C | | | Q9BVX2 |  |
| TMEM107 | Transmembrane protein 107 | | | Q6UX40 |  |
| TMEM109 | Transmembrane protein 109 | | | Q9BVC6 |  |
| TMEM11 | Transmembrane protein 11 | | | P17152 |  |
| TMEM115 | Transmembrane protein 115 | | | Q12893 |  |
| TMEM116 | Transmembrane protein 116 | | | Q8NCL8 |  |
| TMEM120A | Transmembrane protein 120A | | | Q9BXJ8 |  |
| TMEM121 | Transmembrane protein 121 | | | Q9BTD3 |  |
| TMEM123 | Transmembrane protein 123 | | | Q8N131 |  |
| TMEM126A | Transmembrane protein 126A | | | Q9H061 |  |
| TMEM126B | Transmembrane protein 126B | | | Q8IUX1 |  |
| TMEM128 | Transmembrane protein 128 | | | Q5BJH2 |  |
| TMEM129 | Transmembrane protein 129, E3 ubiquitin ligase | | | A0AVI4 |  |
| TMEM134 | Transmembrane protein 134 | | | Q9H6X4 |  |
| TMEM135 | Transmembrane protein 135 | | | Q86UB9 |  |
| TMEM138 | Transmembrane protein 138 | | | Q9NPI0 |  |
| TMEM140 | Transmembrane protein 140 | | | Q9NV12 |  |
| TMEM141 | Transmembrane protein 141 | | | Q96I45 |  |
| TMEM143 | Transmembrane protein 143 | | | Q96AN5 |  |
| TMEM147 | Transmembrane protein 147 | | | Q9BVK8 |  |
| TMEM14A | Transmembrane protein 14A | | | Q9Y6G1 |  |
| TMEM14B | Transmembrane protein 14B | | | Q9NUH8 |  |
| TMEM14C | Transmembrane protein 14C | | | Q9P0S9 |  |
| TMEM150A | Transmembrane protein 150A | | | Q86TG1 |  |
| TMEM154 | Transmembrane protein 154 | | | Q6P9G4 |  |
| TMEM156 | Transmembrane protein 156 | | | Q8N614 |  |
| TMEM160 | Transmembrane protein 160 | | | Q9NX00 |  |
| TMEM161A | Transmembrane protein 161A | | | Q9NX61 |  |
| TMEM161B | Transmembrane protein 161B | | | Q8NDZ6 |  |
| TMEM167A | Transmembrane protein 167A | | | Q8TBQ9 |  |
| TMEM167B | Transmembrane protein 167B | | | Q9NRX6 |  |
| TMEM170A | Transmembrane protein 170A | | | Q8WVE7 |  |
| TMEM175 | Transmembrane protein 175 | | | Q9BSA9 |  |
| TMEM177 | Transmembrane protein 177 | | | Q53S58 |  |
| TMEM179B | Transmembrane protein 179B | | | Q7Z7N9 |  |
| TMEM18 | Transmembrane protein 18 | | | Q96B42 |  |
| TMEM182 | Transmembrane protein 182 | | | Q6ZP80 |  |
| TMEM183A | Transmembrane protein 183A | | | Q8IXX5 |  |
| TMEM184B | Transmembrane protein 184B | | | Q9Y519 |  |
| TMEM184C | Transmembrane protein 184C | | | Q9NVA4 |  |
| TMEM185A | Transmembrane protein 185A | | | Q8NFB2 |  |
| TMEM186 | Transmembrane protein 186 | | | Q96B77 |  |
| TMEM187 | Transmembrane protein 187 | | | Q14656 |  |
| TMEM19 | Transmembrane protein 19 | | | Q96HH6 |  |
| TMEM192 | Transmembrane protein 192 | | | Q8IY95 |  |
| TMEM199 | Transmembrane protein 199 | | | Q8N511 |  |
| TMEM203 | Transmembrane protein 203 | | | Q969S6 |  |
| TMEM204 | Transmembrane protein 204 | | | Q9BSN7 |  |
| TMEM204 | Transmembrane protein 204 | | | Q9BSN7 |  |
| TMEM205 | Transmembrane protein 205 | | | Q6UW68 |  |
| TMEM208 | Transmembrane protein 208 | | | Q9BTX3 |  |
| TMEM209 | Transmembrane protein 209 | | | Q96SK2 |  |
| TMEM214 | Transmembrane protein 214 | | | Q6NUQ4 |  |
| TMEM216 | Transmembrane protein 216 | | | Q9P0N5 |  |
| TMEM218 | Transmembrane protein 218 | | | A2RU14 |  |
| TMEM219 | Transmembrane protein 219 | | | Q86XT9 |  |
| TMEM220 | Transmembrane protein 220 | | | Q6QAJ8 |  |
| TMEM222 | Transmembrane protein 222 | | | Q9H0R3 |  |
| TMEM223 | Transmembrane protein 223 | | | A0PJW6 |  |
| TMEM229B | Transmembrane protein 229B | | | Q8NBD8 |  |
| TMEM230 | Transmembrane protein 230 | | | Q96A57 |  |
| TMEM234 | Transmembrane protein 234 | | | Q8WY98 |  |
| TMEM242 | Transmembrane protein 242 | | | Q9NWH2 |  |
| TMEM243 | Transmembrane protein 243 | | | Q9BU79 |  |
| TMEM248 | Transmembrane protein 248 | | | Q9NWD8 |  |
| TMEM25 | Transmembrane protein 25 | | | Q86YD3 |  |
| TMEM254 | Transmembrane protein 254 | | | Q8TBM7 |  |
| TMEM256 | Transmembrane protein 256 | | | Q8N2U0 |  |
| TMEM258 | Transmembrane protein 258 | | | P61165 |  |
| TMEM259 | Transmembrane protein 259 | | | Q4ZIN3 |  |
| TMEM260 | Transmembrane protein 260 | | | Q9NX78 |  |
| TMEM263 | Transmembrane protein 263 | | | Q8WUH6 |  |
| TMEM30A | Transmembrane protein 30A | | | Q9NV96 |  |
| TMEM33 | Transmembrane protein 33 | | | P57088 |  |
| TMEM38B | Transmembrane protein 38B | | | Q9NVV0 |  |
| TMEM39A | Transmembrane protein 39A | | | Q9NV64 |  |
| TMEM39B | Transmembrane protein 39B | | | Q9GZU3 |  |
| TMEM41A | Transmembrane protein 41A | | | Q96HV5 |  |
| TMEM41B | Transmembrane protein 41B | | | Q5BJD5 |  |
| TMEM42 | Transmembrane protein 42 | | | Q69YG0 |  |
| TMEM43 | Transmembrane protein 43 | | | Q9BTV4 |  |
| TMEM45B | Transmembrane protein 45B | | | Q96B21 |  |
| TMEM50A | Transmembrane protein 50A | | | O95807 |  |
| TMEM50B | Transmembrane protein 50B | | | P56557 |  |
| TMEM53 | Transmembrane protein 53 | | | Q6P2H8 |  |
| TMEM59 | Transmembrane protein 59 | | | Q9BXS4 |  |
| TMEM60 | Transmembrane protein 60 | | | Q9H2L4 |  |
| TMEM62 | Transmembrane protein 62 | | | Q0P6H9 |  |
| TMEM63A | Transmembrane protein 63A | | | O94886 |  |
| TMEM68 | Transmembrane protein 68 | | | Q96MH6 |  |
| TMEM70 | Transmembrane protein 70 | | | Q9BUB7 |  |
| TMEM71 | Transmembrane protein 71 | | | Q6P5X7 |  |
| TMEM79 | Transmembrane protein 79 | | | Q9BSE2 |  |
| TMEM80 | Transmembrane protein 80 | | | Q96HE8 |  |
| TMEM87A | Transmembrane protein 87A | | | Q8NBN3 |  |
| TMEM9 | Transmembrane protein 9 | | | Q9P0T7 |  |
| TMEM91 | Transmembrane protein 91 | | | Q6ZNR0 |  |
| TMEM97 | Transmembrane protein 97 | | | Q5BJF2 |  |
| TMEM9B | TMEM9 domain family member B | | | Q9NQ34 |  |
| TMF1 | TATA element modulatory factor 1 | | | P82094 |  |
| TMIE | Transmembrane inner ear | | | Q8NEW7 |  |
| TMIGD2 | Transmembrane and immunoglobulin domain containing 2 | | | Q96BF3 |  |
| TMLHE | Trimethyllysine hydroxylase, epsilon | | | Q9NVH6 |  |
| TMOD1 | Tropomodulin 1 | | | P28289 |  |
| TMOD2 | Tropomodulin 2 | | | Q9NZR1 |  |
| TMOD3 | Tropomodulin 3 | | | Q9NYL9 |  |
| TMPO | Thymopoietin | | | P42166, P42167 |  |
| TMPRSS3 | Transmembrane serine protease 3 | | | P57727 |  |
| TMSB10 | Thymosin beta 10 | | | P63313 |  |
| TMSB4X | Thymosin beta 4 X-linked | | | P62328 |  |
| TMSB4Y | Thymosin beta 4 Y-linked | | | O14604 |  |
| TMTC3 | Transmembrane O-mannosyltransferase targeting cadherins 3 | | | Q6ZXV5 |  |
| TMTC4 | Transmembrane O-mannosyltransferase targeting cadherins 4 | | | Q5T4D3 |  |
| TMUB1 | Transmembrane and ubiquitin like domain containing 1 | | | Q9BVT8 |  |
| TMUB2 | Transmembrane and ubiquitin like domain containing 2 | | | Q71RG4 |  |
| TMX1 | Thioredoxin related transmembrane protein 1 | | | Q9H3N1 |  |
| TMX2 | Thioredoxin related transmembrane protein 2 | | | Q9Y320 |  |
| TMX3 | Thioredoxin related transmembrane protein 3 | | | Q96JJ7 |  |
| TMX4 | Thioredoxin related transmembrane protein 4 | | | Q9H1E5 |  |
| TNF | Tumor necrosis factor | | | P01375 |  |
| TNFAIP3 | TNF alpha induced protein 3 | | | P21580 |  |
| TNFAIP8 | TNF alpha induced protein 8 | | | O95379 |  |
| TNFAIP8L1 | TNF alpha induced protein 8 like 1 | | | Q8WVP5 |  |
| TNFAIP8L2 | TNF alpha induced protein 8 like 2 | | | Q6P589 |  |
| TNFRSF10A | TNF receptor superfamily member 10a | | | O00220 |  |
| TNFRSF10B | TNF receptor superfamily member 10b | | | O14763 |  |
| TNFRSF10D | TNF receptor superfamily member 10d | | | Q9UBN6 |  |
| TNFRSF12A | TNF receptor superfamily member 12A | | | Q9NP84 |  |
| TNFRSF14 | TNF receptor superfamily member 14 | | | Q92956 |  |
| TNFRSF18 | TNF receptor superfamily member 18 | | | Q9Y5U5 |  |
| TNFRSF1A | TNF receptor superfamily member 1A | | | P19438 |  |
| TNFRSF1B | TNF receptor superfamily member 1B | | | P20333 |  |
| TNFRSF25 | TNF receptor superfamily member 25 | | | Q93038 |  |
| TNFRSF9 | TNF receptor superfamily member 9 | | | Q07011 |  |
| TNFSF10 | TNF superfamily member 10 | | | P50591 |  |
| TNFSF13 | TNF superfamily member 13 | | | O75888 |  |
| TNFSF13B | TNF superfamily member 13b | | | Q9Y275 |  |
| TNFSF14 | TNF superfamily member 14 | | | O43557 |  |
| TNFSF8 | TNF superfamily member 8 | | | P32971 |  |
| TNIP1 | TNFAIP3 interacting protein 1 | | | Q15025 |  |
| TNIP2 | TNFAIP3 interacting protein 2 | | | Q8NFZ5 |  |
| TNIP3 | TNFAIP3 interacting protein 3 | | | Q96KP6 |  |
| TNK1 | Tyrosine kinase non receptor 1 | | | Q13470 |  |
| TNNC2 | Troponin C2, fast skeletal type | | | P02585 |  |
| TNPO1 | Transportin 1 | | | Q92973 |  |
| TNPO3 | Transportin 3 | | | Q9Y5L0 |  |
| TNRC6C | Trinucleotide repeat containing adaptor 6C | | | Q9HCJ0 |  |
| TOB1 | Transducer of ERBB2, 1 | | | P50616 |  |
| TOB2 | Transducer of ERBB2, 2 | | | Q14106 |  |
| TOE1 | Target of EGR1, exonuclease | | | Q96GM8 |  |
| TOLLIP | Toll interacting protein | | | Q9H0E2 |  |
| TOM1 | Target of myb1 membrane trafficking protein | | | O60784 |  |
| TOM1L2 | Target of myb1 like 2 membrane trafficking protein | | | Q6ZVM7 |  |
| TOMM20 | Translocase of outer mitochondrial membrane 20 | | | Q15388 |  |
| TOMM22 | Translocase of outer mitochondrial membrane 22 | | | Q9NS69 |  |
| TOMM34 | Translocase of outer mitochondrial membrane 34 | | | Q15785 |  |
| TOMM40 | Translocase of outer mitochondrial membrane 40 | | | O96008 |  |
| TOMM5 | Translocase of outer mitochondrial membrane 5 | | | Q8N4H5 |  |
| TOMM6 | Translocase of outer mitochondrial membrane 6 | | | Q96B49 |  |
| TOMM7 | Translocase of outer mitochondrial membrane 7 | | | Q9P0U1 |  |
| TOP1MT | DNA topoisomerase I mitochondrial | | | Q969P6 |  |
| TOP3B | DNA topoisomerase III beta | | | O95985 |  |
| TOR1A | Torsin family 1 member A | | | O14656 |  |
| TOR1AIP1 | Torsin 1A interacting protein 1 | | | Q5JTV8 |  |
| TOR1AIP2 | Torsin 1A interacting protein 2 | | | Q8NFQ8, Q9H496 |  |
| TOR2A | Torsin family 2 member A | | | Q5JU69, Q8N2E6 |  |
| TOR3A | Torsin family 3 member A | | | Q9H497 |  |
| TOX | Thymocyte selection associated high mobility group box | | | O94900 |  |
| TOX4 | TOX high mobility group box family member 4 | | | O94842 |  |
| TP53 | Tumor protein p53 | | | P04637 |  |
| TP53I13 | Tumor protein p53 inducible protein 13 | | | Q8NBR0 |  |
| TP53INP1 | Tumor protein p53 inducible nuclear protein 1 | | | Q96A56 |  |
| TP53RK | TP53 regulating kinase | | | Q96S44 |  |
| TPCN1 | Two pore segment channel 1 | | | Q9ULQ1 |  |
| TPCN2 | Two pore segment channel 2 | | | Q8NHX9 |  |
| TPD52 | Tumor protein D52 | | | P55327 |  |
| TPD52L2 | TPD52 like 2 | | | O43399 |  |
| TPGS1 | Tubulin polyglutamylase complex subunit 1 | | | Q6ZTW0 |  |
| TPGS2 | Tubulin polyglutamylase complex subunit 2 | | | Q68CL5 |  |
| TPI1 | Triosephosphate isomerase 1 | | | P60174 |  |
| TPK1 | Thiamin pyrophosphokinase 1 | | | Q9H3S4 |  |
| TPM1 | Tropomyosin 1 | | | P09493 |  |
| TPM3 | Tropomyosin 3 | | | P06753 |  |
| TPM4 | Tropomyosin 4 | | | P67936 |  |
| TPMT | Thiopurine S-methyltransferase | | | P51580 |  |
| TPO | Thyroid peroxidase | | | P07202 |  |
| TPP1 | Tripeptidyl peptidase 1 | | | O14773 |  |
| TPP2 | Tripeptidyl peptidase 2 | | | P29144 |  |
| TPR | Translocated promoter region, nuclear basket protein | | | P12270 |  |
| TPRA1 | Transmembrane protein adipocyte associated 1 | | | Q86W33 |  |
| TPRG1 | Tumor protein p63 regulated 1 | | | Q6ZUI0 |  |
| TPRG1L | Tumor protein p63 regulated 1 like | | | Q5T0D9 |  |
| TPRKB | TP53RK binding protein | | | Q9Y3C4 |  |
| TPST1 | Tyrosylprotein sulfotransferase 1 | | | O60507 |  |
| TPST2 | Tyrosylprotein sulfotransferase 2 | | | O60704 |  |
| TPT1 | Tumor protein, translationally-controlled 1 | | | P13693 |  |
| TRA2A | Transformer 2 alpha homolog | | | Q13595 |  |
| TRA2B | Transformer 2 beta homolog | | | P62995 |  |
| TRABD | TraB domain containing | | | Q9H4I3 |  |
| TRAC | T cell receptor alpha constant | | | P01848 |  |
| TRADD | TNFRSF1A associated via death domain | | | Q15628 |  |
| TRAF1 | TNF receptor associated factor 1 | | | Q13077 |  |
| TRAF2 | TNF receptor associated factor 2 | | | Q12933 |  |
| TRAF3 | TNF receptor associated factor 3 | | | Q13114 |  |
| TRAF3IP2 | TRAF3 interacting protein 2 | | | O43734 |  |
| TRAF3IP3 | TRAF3 interacting protein 3 | | | Q9Y228 |  |
| TRAF4 | TNF receptor associated factor 4 | | | Q9BUZ4 |  |
| TRAF5 | TNF receptor associated factor 5 | | | O00463 |  |
| TRAF6 | TNF receptor associated factor 6 | | | Q9Y4K3 |  |
| TRAF7 | TNF receptor associated factor 7 | | | Q6Q0C0 |  |
| TRAFD1 | TRAF-type zinc finger domain containing 1 | | | O14545 |  |
| TRAK2 | Trafficking kinesin protein 2 | | | O60296 |  |
| TRAM1 | Translocation associated membrane protein 1 | | | Q15629 |  |
| TRAP1 | TNF receptor associated protein 1 | | | Q12931 |  |
| TRAPPC1 | Trafficking protein particle complex subunit 1 | | | Q9Y5R8 |  |
| TRAPPC10 | Trafficking protein particle complex subunit 10 | | | P48553 |  |
| TRAPPC11 | Trafficking protein particle complex subunit 11 | | | Q7Z392 |  |
| TRAPPC12 | Trafficking protein particle complex subunit 12 | | | Q8WVT3 |  |
| TRAPPC2 | Trafficking protein particle complex subunit 2 | | | P0DI81 |  |
| TRAPPC2L | Trafficking protein particle complex subunit 2L | | | Q9UL33 |  |
| TRAPPC3 | Trafficking protein particle complex subunit 3 | | | O43617 |  |
| TRAPPC4 | Trafficking protein particle complex subunit 4 | | | Q9Y296 |  |
| TRAPPC5 | Trafficking protein particle complex subunit 5 | | | Q8IUR0 |  |
| TRAPPC6A | Trafficking protein particle complex subunit 6A | | | O75865 |  |
| TRAPPC6B | Trafficking protein particle complex subunit 6B | | | Q86SZ2 |  |
| TRAPPC8 | Trafficking protein particle complex subunit 8 | | | Q9Y2L5 |  |
| TRAPPC9 | Trafficking protein particle complex subunit 9 | | | Q96Q05 |  |
| TRAT1 | T cell receptor associated transmembrane adaptor 1 | | | Q6PIZ9 |  |
| TRBC1 | T cell receptor beta constant 1 | | | P01850 |  |
| TRBC2 | T cell receptor beta constant 2 | | | A0A5B9 |  |
| TRDC | T cell receptor delta constant | | | B7Z8K6 |  |
| TRDMT1 | TRNA aspartic acid methyltransferase 1 | | | O14717 |  |
| TREML2 | Triggering receptor expressed on myeloid cells like 2 | | | Q5T2D2 |  |
| TRERF1 | Transcriptional regulating factor 1 | | | Q96PN7 |  |
| TREX1 | Three prime repair exonuclease 1 | | | Q9NSU2 |  |
| TRGC1 | T cell receptor gamma constant 1 | | |  |  |
| TRGC2 | T cell receptor gamma constant 2 | | |  |  |
| TRGV3 | T cell receptor gamma variable 3 | | | P03979 |  |
| TRIAP1 | TP53 regulated inhibitor of apoptosis 1 | | | O43715 |  |
| TRIB2 | Tribbles pseudokinase 2 | | | Q92519 |  |
| TRIB3 | Tribbles pseudokinase 3 | | | Q96RU7 |  |
| TRIM11 | Tripartite motif containing 11 | | | Q96F44 |  |
| TRIM13 | Tripartite motif containing 13 | | | O60858 |  |
| TRIM14 | Tripartite motif containing 14 | | | Q14142 |  |
| TRIM16 | Tripartite motif containing 16 | | | O95361 |  |
| TRIM2 | Tripartite motif containing 2 | | | Q9C040 |  |
| TRIM21 | Tripartite motif containing 21 | | | P19474 |  |
| TRIM22 | Tripartite motif containing 22 | | | Q8IYM9 |  |
| TRIM23 | Tripartite motif containing 23 | | | P36406 |  |
| TRIM26 | Tripartite motif containing 26 | | | Q12899 |  |
| TRIM27 | Tripartite motif containing 27 | | | P14373 |  |
| TRIM28 | Tripartite motif containing 28 | | | Q13263 |  |
| TRIM32 | Tripartite motif containing 32 | | | Q13049 |  |
| TRIM33 | Tripartite motif containing 33 | | | Q9UPN9 |  |
| TRIM34 | Tripartite motif containing 34 | | | Q9BYJ4 |  |
| TRIM35 | Tripartite motif containing 35 | | | Q9UPQ4 |  |
| TRIM37 | Tripartite motif containing 37 | | | O94972 |  |
| TRIM38 | Tripartite motif containing 38 | | | O00635 |  |
| TRIM39 | Tripartite motif containing 39 | | | Q9HCM9 |  |
| TRIM4 | Tripartite motif containing 4 | | | Q9C037 |  |
| TRIM44 | Tripartite motif containing 44 | | | Q96DX7 |  |
| TRIM47 | Tripartite motif containing 47 | | | Q96LD4 |  |
| TRIM5 | Tripartite motif containing 5 | | | Q9C035 |  |
| TRIM52 | Tripartite motif containing 52 | | | Q96A61 |  |
| TRIM56 | Tripartite motif containing 56 | | | Q9BRZ2 |  |
| TRIM59 | Tripartite motif containing 59 | | | Q8IWR1 |  |
| TRIM61 | Tripartite motif containing 61 | | | Q5EBN2 |  |
| TRIM62 | Tripartite motif containing 62 | | | Q9BVG3 |  |
| TRIM65 | Tripartite motif containing 65 | | | Q6PJ69 |  |
| TRIM68 | Tripartite motif containing 68 | | | Q6AZZ1 |  |
| TRIM8 | Tripartite motif containing 8 | | | Q9BZR9 |  |
| TRIOBP | TRIO and F-actin binding protein | | | Q9H2D6 |  |
| TRIP4 | Thyroid hormone receptor interactor 4 | | | Q15650 |  |
| TRIP6 | Thyroid hormone receptor interactor 6 | | | Q15654 |  |
| TRIT1 | TRNA isopentenyltransferase 1 | | | Q9H3H1 |  |
| TRMO | TRNA methyltransferase O | | | Q9BU70 |  |
| TRMT1 | TRNA methyltransferase 1 | | | Q9NXH9 |  |
| TRMT10B | TRNA methyltransferase 10B | | | Q6PF06 |  |
| TRMT10C | TRNA methyltransferase 10C, mitochondrial RNase P subunit | | | Q7L0Y3 |  |
| TRMT11 | TRNA methyltransferase 11 homolog | | | Q7Z4G4 |  |
| TRMT112 | TRNA methyltransferase activator subunit 11-2 | | | Q9UI30 |  |
| TRMT12 | TRNA methyltransferase 12 homolog | | | Q53H54 |  |
| TRMT13 | TRNA methyltransferase 13 homolog | | | Q9NUP7 |  |
| TRMT1L | TRNA methyltransferase 1 like | | | Q7Z2T5 |  |
| TRMT2A | TRNA methyltransferase 2 homolog A | | | Q8IZ69 |  |
| TRMT2B | TRNA methyltransferase 2 homolog B | | | Q96GJ1 |  |
| TRMT5 | TRNA methyltransferase 5 | | | Q32P41 |  |
| TRMT6 | TRNA methyltransferase 6 non-catalytic subunit | | | Q9UJA5 |  |
| TRMT61A | TRNA methyltransferase 61A | | | Q96FX7 |  |
| TRMT61B | TRNA methyltransferase 61B | | | Q9BVS5 |  |
| TRMU | TRNA mitochondrial 2-thiouridylase | | | O75648 |  |
| TRNAU1AP | TRNA selenocysteine 1 associated protein 1 | | | Q9NX07 |  |
| TRNT1 | TRNA nucleotidyl transferase 1 | | | Q96Q11 |  |
| TROAP | Trophinin associated protein | | | Q12815 |  |
| TRPC4AP | Transient receptor potential cation channel subfamily C member 4 associated protein | | | Q8TEL6 |  |
| TRPT1 | TRNA phosphotransferase 1 | | | Q86TN4 |  |
| TRPV2 | Transient receptor potential cation channel subfamily V member 2 | | | Q9Y5S1 |  |
| TRUB1 | TruB pseudouridine synthase family member 1 | | | Q8WWH5 |  |
| TRUB2 | TruB pseudouridine synthase family member 2 | | | O95900 |  |
| TSACC | TSSK6 activating cochaperone | | | Q96A04 |  |
| TSC1 | TSC complex subunit 1 | | | Q92574 |  |
| TSC2 | TSC complex subunit 2 | | | P49815 |  |
| TSC22D1 | TSC22 domain family member 1 | | | Q15714 |  |
| TSC22D2 | TSC22 domain family member 2 | | | O75157 |  |
| TSC22D3 | TSC22 domain family member 3 | | | Q99576 |  |
| TSC22D4 | TSC22 domain family member 4 | | | Q9Y3Q8 |  |
| TSEN15 | TRNA splicing endonuclease subunit 15 | | | Q8WW01 |  |
| TSEN2 | TRNA splicing endonuclease subunit 2 | | | Q8NCE0 |  |
| TSFM | Ts translation elongation factor, mitochondrial | | | P43897 |  |
| TSG101 | Tumor susceptibility 101 | | | Q99816 |  |
| TSGA10 | Testis specific 10 | | | Q9BZW7 |  |
| TSHZ1 | Teashirt zinc finger homeobox 1 | | | Q6ZSZ6 |  |
| TSHZ2 | Teashirt zinc finger homeobox 2 | | | Q9NRE2 |  |
| TSHZ2 | Teashirt zinc finger homeobox 2 | | | Q9NRE2 |  |
| TSN | Translin | | | Q15631 |  |
| TSNAX | Translin associated factor X | | | Q99598 |  |
| TSPAN14 | Tetraspanin 14 | | | Q8NG11 |  |
| TSPAN15 | Tetraspanin 15 | | | O95858 |  |
| TSPAN17 | Tetraspanin 17 | | | Q96FV3 |  |
| TSPAN18 | Tetraspanin 18 | | | Q96SJ8 |  |
| TSPAN2 | Tetraspanin 2 | | | O60636 |  |
| TSPAN3 | Tetraspanin 3 | | | O60637 |  |
| TSPAN31 | Tetraspanin 31 | | | Q12999 |  |
| TSPAN32 | Tetraspanin 32 | | | Q96QS1 |  |
| TSPAN4 | Tetraspanin 4 | | | O14817 |  |
| TSPAN5 | Tetraspanin 5 | | | P62079 |  |
| TSPAN6 | Tetraspanin 6 | | | O43657 |  |
| TSPO | Translocator protein | | | P30536 |  |
| TSPYL1 | TSPY like 1 | | | Q9H0U9 |  |
| TSPYL2 | TSPY like 2 | | | Q9H2G4 |  |
| TSR1 | TSR1 ribosome maturation factor | | | Q2NL82 |  |
| TSR2 | TSR2 ribosome maturation factor | | | Q969E8 |  |
| TSSC4 | Tumor suppressing subtransferable candidate 4 | | | Q9Y5U2 |  |
| TSTD2 | Thiosulfate sulfurtransferase like domain containing 2 | | | Q5T7W7 |  |
| TTC1 | Tetratricopeptide repeat domain 1 | | | Q99614 |  |
| TTC12 | Tetratricopeptide repeat domain 12 | | | Q9H892 |  |
| TTC13 | Tetratricopeptide repeat domain 13 | | | Q8NBP0 |  |
| TTC14 | Tetratricopeptide repeat domain 14 | | | Q96N46 |  |
| TTC17 | Tetratricopeptide repeat domain 17 | | | Q96AE7 |  |
| TTC27 | Tetratricopeptide repeat domain 27 | | | Q6P3X3 |  |
| TTC31 | Tetratricopeptide repeat domain 31 | | | Q49AM3 |  |
| TTC32 | Tetratricopeptide repeat domain 32 | | | Q5I0X7 |  |
| TTC33 | Tetratricopeptide repeat domain 33 | | | Q6PID6 |  |
| TTC38 | Tetratricopeptide repeat domain 38 | | | Q5R3I4 |  |
| TTC39B | Tetratricopeptide repeat domain 39B | | | Q5VTQ0 |  |
| TTC4 | Tetratricopeptide repeat domain 4 | | | O95801 |  |
| TTC5 | Tetratricopeptide repeat domain 5 | | | Q8N0Z6 |  |
| TTC9 | Tetratricopeptide repeat domain 9 | | | Q92623 |  |
| TTC9C | Tetratricopeptide repeat domain 9C | | | Q8N5M4 |  |
| TTF1 | Transcription termination factor 1 | | | Q15361 |  |
| TTF2 | Transcription termination factor 2 | | | Q9UNY4 |  |
| TTI2 | TELO2 interacting protein 2 | | | Q6NXR4 |  |
| TTLL1 | TTL family tubulin polyglutamylase complex subunit L1 | | | O95922 |  |
| TTLL12 | Tubulin tyrosine ligase like 12 | | | Q14166 |  |
| TTLL3 | Tubulin tyrosine ligase like 3 | | | Q9Y4R7 |  |
| TTLL4 | Tubulin tyrosine ligase like 4 | | | Q14679 |  |
| TTN | Titin | | | Q8WZ42 |  |
| TTPAL | Alpha tocopherol transfer protein like | | | Q9BTX7 |  |
| TTYH2 | Tweety family member 2 | | | Q9BSA4 |  |
| TUBA1A | Tubulin alpha 1a | | | Q71U36 |  |
| TUBA1B | Tubulin alpha 1b | | | P68363 |  |
| TUBA1C | Tubulin alpha 1c | | | Q9BQE3 |  |
| TUBA4A | Tubulin alpha 4a | | | P68366 |  |
| TUBB | Tubulin beta class I | | | P07437 |  |
| TUBB2A | Tubulin beta 2A class IIa | | | Q13885 |  |
| TUBB4A | Tubulin beta 4A class IVa | | | P04350 |  |
| TUBB4B | Tubulin beta 4B class IVb | | | P68371 |  |
| TUBD1 | Tubulin delta 1 | | | Q9UJT1 |  |
| TUBE1 | Tubulin epsilon 1 | | | Q9UJT0 |  |
| TUBG1 | Tubulin gamma 1 | | | P23258 |  |
| TUBGCP2 | Tubulin gamma complex associated protein 2 | | | Q9BSJ2 |  |
| TUBGCP3 | Tubulin gamma complex associated protein 3 | | | Q96CW5 |  |
| TUBGCP4 | Tubulin gamma complex associated protein 4 | | | Q9UGJ1 |  |
| TUBGCP5 | Tubulin gamma complex associated protein 5 | | | Q96RT8 |  |
| TUFM | Tu translation elongation factor, mitochondrial | | | P49411 |  |
| TULP3 | TUB like protein 3 | | | O75386 |  |
| TULP4 | TUB like protein 4 | | | Q9NRJ4 |  |
| TUSC2 | Tumor suppressor 2, mitochondrial calcium regulator | | | O75896 |  |
| TUT1 | Terminal uridylyl transferase 1, U6 snRNA-specific | | | Q9H6E5 |  |
| TVP23B | Trans-golgi network vesicle protein 23 homolog B | | | Q9NYZ1 |  |
| TVP23C | Trans-golgi network vesicle protein 23 homolog C | | | Q96ET8 |  |
| TWF1 | Twinfilin actin binding protein 1 | | | Q12792 |  |
| TWF2 | Twinfilin actin binding protein 2 | | | Q6IBS0 |  |
| TWSG1 | Twisted gastrulation BMP signaling modulator 1 | | | Q9GZX9 |  |
| TXK | TXK tyrosine kinase | | | P42681 |  |
| TXLNA | Taxilin alpha | | | P40222 |  |
| TXN | Thioredoxin | | | P10599 |  |
| TXN2 | Thioredoxin 2 | | | Q99757 |  |
| TXNDC11 | Thioredoxin domain containing 11 | | | Q6PKC3 |  |
| TXNDC12 | Thioredoxin domain containing 12 | | | O95881 |  |
| TXNDC15 | Thioredoxin domain containing 15 | | | Q96J42 |  |
| TXNDC16 | Thioredoxin domain containing 16 | | | Q9P2K2 |  |
| TXNDC17 | Thioredoxin domain containing 17 | | | Q9BRA2 |  |
| TXNDC9 | Thioredoxin domain containing 9 | | | O14530 |  |
| TXNIP | Thioredoxin interacting protein | | | Q9H3M7 |  |
| TXNL1 | Thioredoxin like 1 | | | O43396 |  |
| TXNL4A | Thioredoxin like 4A | | | P83876 |  |
| TXNL4B | Thioredoxin like 4B | | | Q9NX01 |  |
| TXNRD1 | Thioredoxin reductase 1 | | | Q16881 |  |
| TXNRD2 | Thioredoxin reductase 2 | | | Q9NNW7 |  |
| TYK2 | Tyrosine kinase 2 | | | P29597 |  |
| TYMP | Thymidine phosphorylase | | | P19971 |  |
| TYMS | Thymidylate synthetase | | | P04818 |  |
| TYROBP | Transmembrane immune signaling adaptor TYROBP | | | O43914 |  |
| TYSND1 | Trypsin like peroxisomal matrix peptidase 1 | | | Q2T9J0 |  |
| TYW1 | TRNA-yW synthesizing protein 1 homolog | | | Q9NV66 |  |
| TYW1B | TRNA-yW synthesizing protein 1 homolog B | | | Q6NUM6 |  |
| TYW3 | TRNA-yW synthesizing protein 3 homolog | | | Q6IPR3 |  |
| U2AF1 | U2 small nuclear RNA auxiliary factor 1 | | | Q01081 |  |
| U2AF1L4 | U2 small nuclear RNA auxiliary factor 1 like 4 | | | Q8WU68 |  |
| U2AF2 | U2 small nuclear RNA auxiliary factor 2 | | | P26368 |  |
| U2SURP | U2 snRNP associated SURP domain containing | | | O15042 |  |
| UAP1 | UDP-N-acetylglucosamine pyrophosphorylase 1 | | | Q16222 |  |
| UBA1 | Ubiquitin like modifier activating enzyme 1 | | | P22314 |  |
| UBA2 | Ubiquitin like modifier activating enzyme 2 | | | Q9UBT2 |  |
| UBA3 | Ubiquitin like modifier activating enzyme 3 | | | Q8TBC4 |  |
| UBA5 | Ubiquitin like modifier activating enzyme 5 | | | Q9GZZ9 |  |
| UBA52 | Ubiquitin A-52 residue ribosomal protein fusion product 1 | | | P62987 |  |
| UBA6 | Ubiquitin like modifier activating enzyme 6 | | | A0AVT1 |  |
| UBA7 | Ubiquitin like modifier activating enzyme 7 | | | P41226 |  |
| UBAC1 | UBA domain containing 1 | | | Q9BSL1 |  |
| UBAC2 | UBA domain containing 2 | | | Q8NBM4 |  |
| UBALD1 | UBA like domain containing 1 | | | Q8TB05 |  |
| UBALD2 | UBA like domain containing 2 | | | Q8IYN6 |  |
| UBAP1 | Ubiquitin associated protein 1 | | | Q9NZ09 |  |
| UBAP2 | Ubiquitin associated protein 2 | | | Q5T6F2 |  |
| UBASH3A | Ubiquitin associated and SH3 domain containing A | | | P57075 |  |
| UBASH3B | Ubiquitin associated and SH3 domain containing B | | | Q8TF42 |  |
| UBB | Ubiquitin B | | | P0CG47 |  |
| UBE2A | Ubiquitin conjugating enzyme E2 A | | | P49459 |  |
| UBE2B | Ubiquitin conjugating enzyme E2 B | | | P63146 |  |
| UBE2D1 | Ubiquitin conjugating enzyme E2 D1 | | | P51668 |  |
| UBE2D2 | Ubiquitin conjugating enzyme E2 D2 | | | P62837 |  |
| UBE2D3 | Ubiquitin conjugating enzyme E2 D3 | | | P61077 |  |
| UBE2D4 | Ubiquitin conjugating enzyme E2 D4 (putative) | | | Q9Y2X8 |  |
| UBE2E1 | Ubiquitin conjugating enzyme E2 E1 | | | P51965 |  |
| UBE2E2 | Ubiquitin conjugating enzyme E2 E2 | | | Q96LR5 |  |
| UBE2E3 | Ubiquitin conjugating enzyme E2 E3 | | | Q969T4 |  |
| UBE2F | Ubiquitin conjugating enzyme E2 F (putative) | | | Q969M7 |  |
| UBE2G1 | Ubiquitin conjugating enzyme E2 G1 | | | P62253 |  |
| UBE2G2 | Ubiquitin conjugating enzyme E2 G2 | | | P60604 |  |
| UBE2H | Ubiquitin conjugating enzyme E2 H | | | P62256 |  |
| UBE2I | Ubiquitin conjugating enzyme E2 I | | | P63279 |  |
| UBE2J1 | Ubiquitin conjugating enzyme E2 J1 | | | Q9Y385 |  |
| UBE2J2 | Ubiquitin conjugating enzyme E2 J2 | | | Q8N2K1 |  |
| UBE2K | Ubiquitin conjugating enzyme E2 K | | | P61086 |  |
| UBE2L3 | Ubiquitin conjugating enzyme E2 L3 | | | P68036 |  |
| UBE2L6 | Ubiquitin conjugating enzyme E2 L6 | | | O14933 |  |
| UBE2M | Ubiquitin conjugating enzyme E2 M | | | P61081 |  |
| UBE2N | Ubiquitin conjugating enzyme E2 N | | | P61088 |  |
| UBE2Q1 | Ubiquitin conjugating enzyme E2 Q1 | | | Q7Z7E8 |  |
| UBE2Q2 | Ubiquitin conjugating enzyme E2 Q2 | | | Q8WVN8 |  |
| UBE2R2 | Ubiquitin conjugating enzyme E2 R2 | | | Q712K3 |  |
| UBE2S | Ubiquitin conjugating enzyme E2 S | | | Q16763 |  |
| UBE2T | Ubiquitin conjugating enzyme E2 T | | | Q9NPD8 |  |
| UBE2V1 | Ubiquitin conjugating enzyme E2 V1 | | | Q13404 |  |
| UBE2V2 | Ubiquitin conjugating enzyme E2 V2 | | | Q15819 |  |
| UBE2W | Ubiquitin conjugating enzyme E2 W | | | Q96B02 |  |
| UBE2Z | Ubiquitin conjugating enzyme E2 Z | | | Q9H832 |  |
| UBE3A | Ubiquitin protein ligase E3A | | | Q05086 |  |
| UBE3B | Ubiquitin protein ligase E3B | | | Q7Z3V4 |  |
| UBE3C | Ubiquitin protein ligase E3C | | | Q15386 |  |
| UBE3D | Ubiquitin protein ligase E3D | | | Q7Z6J8 |  |
| UBE4A | Ubiquitination factor E4A | | | Q14139 |  |
| UBIAD1 | UbiA prenyltransferase domain containing 1 | | | Q9Y5Z9 |  |
| UBL3 | Ubiquitin like 3 | | | O95164 |  |
| UBL4A | Ubiquitin like 4A | | | P11441 |  |
| UBL5 | Ubiquitin like 5 | | | Q9BZL1 |  |
| UBL7 | Ubiquitin like 7 | | | Q96S82 |  |
| UBOX5 | U-box domain containing 5 | | | O94941 |  |
| UBP1 | Upstream binding protein 1 | | | Q9NZI7 |  |
| UBQLN1 | Ubiquilin 1 | | | Q9UMX0 |  |
| UBQLN2 | Ubiquilin 2 | | | Q9UHD9 |  |
| UBR2 | Ubiquitin protein ligase E3 component n-recognin 2 | | | Q8IWV8 |  |
| UBXN1 | UBX domain protein 1 | | | Q04323 |  |
| UBXN11 | UBX domain protein 11 | | | Q5T124 |  |
| UBXN2A | UBX domain protein 2A | | | P68543 |  |
| UBXN2B | UBX domain protein 2B | | | Q14CS0 |  |
| UBXN4 | UBX domain protein 4 | | | Q92575 |  |
| UBXN6 | UBX domain protein 6 | | | Q9BZV1 |  |
| UBXN7 | UBX domain protein 7 | | | O94888 |  |
| UBXN8 | UBX domain protein 8 | | | O00124 |  |
| UCHL3 | Ubiquitin C-terminal hydrolase L3 | | | P15374 |  |
| UCHL5 | Ubiquitin C-terminal hydrolase L5 | | | Q9Y5K5 |  |
| UCK1 | Uridine-cytidine kinase 1 | | | Q9HA47 |  |
| UCK2 | Uridine-cytidine kinase 2 | | | Q9BZX2 |  |
| UCKL1 | Uridine-cytidine kinase 1 like 1 | | | Q9NWZ5 |  |
| UCP2 | Uncoupling protein 2 | | | P55851 |  |
| UEVLD | UEV and lactate/malate dehyrogenase domains | | | Q8IX04 |  |
| UFC1 | Ubiquitin-fold modifier conjugating enzyme 1 | | | Q9Y3C8 |  |
| UFL1 | UFM1 specific ligase 1 | | | O94874 |  |
| UFM1 | Ubiquitin fold modifier 1 | | | P61960 |  |
| UFSP1 | UFM1 specific peptidase 1 (inactive) | | | Q6NVU6 |  |
| UFSP2 | UFM1 specific peptidase 2 | | | Q9NUQ7 |  |
| UGCG | UDP-glucose ceramide glucosyltransferase | | | Q16739 |  |
| UGDH | UDP-glucose 6-dehydrogenase | | | O60701 |  |
| UGGT1 | UDP-glucose glycoprotein glucosyltransferase 1 | | | Q9NYU2 |  |
| UGP2 | UDP-glucose pyrophosphorylase 2 | | | Q16851 |  |
| UHMK1 | U2AF homology motif kinase 1 | | | Q8TAS1 |  |
| UHRF2 | Ubiquitin like with PHD and ring finger domains 2 | | | Q96PU4 |  |
| UIMC1 | Ubiquitin interaction motif containing 1 | | | Q96RL1 |  |
| ULK3 | Unc-51 like kinase 3 | | | Q6PHR2 |  |
| ULK4 | Unc-51 like kinase 4 | | | Q96C45 |  |
| UMPS | Uridine monophosphate synthetase | | | P11172 |  |
| UNC119 | Unc-119 lipid binding chaperone | | | Q13432 |  |
| UNC13D | Unc-13 homolog D | | | Q70J99 |  |
| UNC45A | Unc-45 myosin chaperone A | | | Q9H3U1 |  |
| UNC50 | Unc-50 inner nuclear membrane RNA binding protein | | | Q53HI1 |  |
| UNG | Uracil DNA glycosylase | | | P13051 |  |
| UNKL | Unk like zinc finger | | | Q9H9P5 |  |
| UPF3A | UPF3A regulator of nonsense mediated mRNA decay | | | Q9H1J1 |  |
| UPP1 | Uridine phosphorylase 1 | | | Q16831 |  |
| UPRT | Uracil phosphoribosyltransferase homolog | | | Q96BW1 |  |
| UQCC1 | Ubiquinol-cytochrome c reductase complex assembly factor 1 | | | Q9NVA1 |  |
| UQCC2 | Ubiquinol-cytochrome c reductase complex assembly factor 2 | | | Q9BRT2 |  |
| UQCR10 | Ubiquinol-cytochrome c reductase, complex III subunit X | | | Q9UDW1 |  |
| UQCR11 | Ubiquinol-cytochrome c reductase, complex III subunit XI | | | O14957 |  |
| UQCRB | Ubiquinol-cytochrome c reductase binding protein | | | P14927 |  |
| UQCRC1 | Ubiquinol-cytochrome c reductase core protein 1 | | | P31930 |  |
| UQCRC2 | Ubiquinol-cytochrome c reductase core protein 2 | | | P22695 |  |
| UQCRFS1 | Ubiquinol-cytochrome c reductase, Rieske iron-sulfur polypeptide 1 | | | P47985 |  |
| UQCRH | Ubiquinol-cytochrome c reductase hinge protein | | | P07919 |  |
| UQCRQ | Ubiquinol-cytochrome c reductase complex III subunit VII | | | O14949 |  |
| URB2 | URB2 ribosome biogenesis homolog | | | Q14146 |  |
| URGCP | Upregulator of cell proliferation | | | Q8TCY9 |  |
| URI1 | URI1 prefoldin like chaperone | | | O94763 |  |
| URM1 | Ubiquitin related modifier 1 | | | Q9BTM9 |  |
| UROD | Uroporphyrinogen decarboxylase | | | P06132 |  |
| UROS | Uroporphyrinogen III synthase | | | P10746 |  |
| USB1 | U6 snRNA biogenesis phosphodiesterase 1 | | | Q9BQ65 |  |
| USE1 | Unconventional SNARE in the ER 1 | | | Q9NZ43 |  |
| USF1 | Upstream transcription factor 1 | | | P22415 |  |
| USF2 | Upstream transcription factor 2, c-fos interacting | | | Q15853 |  |
| USO1 | USO1 vesicle transport factor | | | O60763 |  |
| USP1 | Ubiquitin specific peptidase 1 | | | O94782 |  |
| USP10 | Ubiquitin specific peptidase 10 | | | Q14694 |  |
| USP11 | Ubiquitin specific peptidase 11 | | | P51784 |  |
| USP13 | Ubiquitin specific peptidase 13 | | | Q92995 |  |
| USP14 | Ubiquitin specific peptidase 14 | | | P54578 |  |
| USP15 | Ubiquitin specific peptidase 15 | | | Q9Y4E8 |  |
| USP16 | Ubiquitin specific peptidase 16 | | | Q9Y5T5 |  |
| USP18 | Ubiquitin specific peptidase 18 | | | Q9UMW8 |  |
| USP19 | Ubiquitin specific peptidase 19 | | | O94966 |  |
| USP20 | Ubiquitin specific peptidase 20 | | | Q9Y2K6 |  |
| USP21 | Ubiquitin specific peptidase 21 | | | Q9UK80 |  |
| USP22 | Ubiquitin specific peptidase 22 | | | Q9UPT9 |  |
| USP24 | Ubiquitin specific peptidase 24 | | | Q9UPU5 |  |
| USP25 | Ubiquitin specific peptidase 25 | | | Q9UHP3 |  |
| USP28 | Ubiquitin specific peptidase 28 | | | Q96RU2 |  |
| USP3 | Ubiquitin specific peptidase 3 | | | Q9Y6I4 |  |
| USP30 | Ubiquitin specific peptidase 30 | | | Q70CQ3 |  |
| USP32 | Ubiquitin specific peptidase 32 | | | Q8NFA0 |  |
| USP33 | Ubiquitin specific peptidase 33 | | | Q8TEY7 |  |
| USP36 | Ubiquitin specific peptidase 36 | | | Q9P275 |  |
| USP38 | Ubiquitin specific peptidase 38 | | | Q8NB14 |  |
| USP39 | Ubiquitin specific peptidase 39 | | | Q53GS9 |  |
| USP4 | Ubiquitin specific peptidase 4 | | | Q13107 |  |
| USP44 | Ubiquitin specific peptidase 44 | | | Q9H0E7 |  |
| USP44 | Ubiquitin specific peptidase 44 | | | Q9H0E7 |  |
| USP45 | Ubiquitin specific peptidase 45 | | | Q70EL2 |  |
| USP46 | Ubiquitin specific peptidase 46 | | | P62068 |  |
| USP47 | Ubiquitin specific peptidase 47 | | | Q96K76 |  |
| USP48 | Ubiquitin specific peptidase 48 | | | Q86UV5 |  |
| USP5 | Ubiquitin specific peptidase 5 | | | P45974 |  |
| USP53 | Ubiquitin specific peptidase 53 | | | Q70EK8 |  |
| USP7 | Ubiquitin specific peptidase 7 | | | Q93009 |  |
| USP8 | Ubiquitin specific peptidase 8 | | | P40818 |  |
| USP9X | Ubiquitin specific peptidase 9 X-linked | | | Q93008 |  |
| USPL1 | Ubiquitin specific peptidase like 1 | | | Q5W0Q7 |  |
| UTP14A | UTP14A small subunit processome component | | | Q9BVJ6 |  |
| UTP15 | UTP15 small subunit processome component | | | Q8TED0 |  |
| UTP18 | UTP18 small subunit processome component | | | Q9Y5J1 |  |
| UTP23 | UTP23 small subunit processome component | | | Q9BRU9 |  |
| UTP3 | UTP3 small subunit processome component | | | Q9NQZ2 |  |
| UTP4 | UTP4 small subunit processome component | | | Q969X6 |  |
| UTP6 | UTP6 small subunit processome component | | | Q9NYH9 |  |
| UTS2 | Urotensin 2 | | | O95399 |  |
| UVRAG | UV radiation resistance associated | | | Q9P2Y5 |  |
| UXS1 | UDP-glucuronate decarboxylase 1 | | | Q8NBZ7 |  |
| UXT | Ubiquitously expressed prefoldin like chaperone | | | Q9UBK9 |  |
| VAC14 | VAC14 component of PIKFYVE complex | | | Q08AM6 |  |
| VAMP1 | Vesicle associated membrane protein 1 | | | P23763 |  |
| VAMP2 | Vesicle associated membrane protein 2 | | | P63027 |  |
| VAMP3 | Vesicle associated membrane protein 3 | | | Q15836 |  |
| VAMP4 | Vesicle associated membrane protein 4 | | | O75379 |  |
| VAMP5 | Vesicle associated membrane protein 5 | | | O95183 |  |
| VAMP8 | Vesicle associated membrane protein 8 | | | Q9BV40 |  |
| VANGL1 | VANGL planar cell polarity protein 1 | | | Q8TAA9 |  |
| VAPA | VAMP associated protein A | | | Q9P0L0 |  |
| VAPB | VAMP associated protein B and C | | | O95292 |  |
| VASP | Vasodilator stimulated phosphoprotein | | | P50552 |  |
| VAT1 | Vesicle amine transport 1 | | | Q99536 |  |
| VBP1 | VHL binding protein 1 | | | P61758 |  |
| VCL | Vinculin | | | P18206 |  |
| VCP | Valosin containing protein | | | P55072 |  |
| VDAC1 | Voltage dependent anion channel 1 | | | P21796 |  |
| VDAC2 | Voltage dependent anion channel 2 | | | P45880 |  |
| VDAC3 | Voltage dependent anion channel 3 | | | Q9Y277 |  |
| VEZF1 | Vascular endothelial zinc finger 1 | | | Q14119 |  |
| VEZT | Vezatin, adherens junctions transmembrane protein | | | Q9HBM0 |  |
| VGLL4 | Vestigial like family member 4 | | | Q14135 |  |
| VHL | Von Hippel-Lindau tumor suppressor | | | P40337 |  |
| VILL | Villin like | | | O15195 |  |
| VIM | Vimentin | | | P08670 |  |
| VIPR1 | Vasoactive intestinal peptide receptor 1 | | | P32241 |  |
| VKORC1 | Vitamin K epoxide reductase complex subunit 1 | | | Q9BQB6 |  |
| VKORC1L1 | Vitamin K epoxide reductase complex subunit 1 like 1 | | | Q8N0U8 |  |
| VMA21 | Vacuolar ATPase assembly factor VMA21 | | | Q3ZAQ7 |  |
| VMAC | Vimentin type intermediate filament associated coiled-coil protein | | | Q2NL98 |  |
| VMP1 | Vacuole membrane protein 1 | | | Q96GC9 |  |
| VNN2 | Vanin 2 | | | O95498 |  |
| VOPP1 | VOPP1 WW domain binding protein | | | Q96AW1 |  |
| VPS16 | VPS16 core subunit of CORVET and HOPS complexes | | | Q9H269 |  |
| VPS18 | VPS18 core subunit of CORVET and HOPS complexes | | | Q9P253 |  |
| VPS25 | Vacuolar protein sorting 25 homolog | | | Q9BRG1 |  |
| VPS26A | VPS26 retromer complex component A | | | O75436 |  |
| VPS26B | VPS26 retromer complex component B | | | Q4G0F5 |  |
| VPS29 | VPS29 retromer complex component | | | Q9UBQ0 |  |
| VPS33A | VPS33A core subunit of CORVET and HOPS complexes | | | Q96AX1 |  |
| VPS33B | VPS33B late endosome and lysosome associated | | | Q9H267 |  |
| VPS35 | VPS35 retromer complex component | | | Q96QK1 |  |
| VPS36 | Vacuolar protein sorting 36 homolog | | | Q86VN1 |  |
| VPS37A | VPS37A subunit of ESCRT-I | | | Q8NEZ2 |  |
| VPS37B | VPS37B subunit of ESCRT-I | | | Q9H9H4 |  |
| VPS39 | VPS39 subunit of HOPS complex | | | Q96JC1 |  |
| VPS41 | VPS41 subunit of HOPS complex | | | P49754 |  |
| VPS45 | Vacuolar protein sorting 45 homolog | | | Q9NRW7 |  |
| VPS4A | Vacuolar protein sorting 4 homolog A | | | Q9UN37 |  |
| VPS4B | Vacuolar protein sorting 4 homolog B | | | O75351 |  |
| VPS50 | VPS50 subunit of EARP/GARPII complex | | | Q96JG6 |  |
| VPS52 | VPS52 subunit of GARP complex | | | Q8N1B4 |  |
| VPS53 | VPS53 subunit of GARP complex | | | Q5VIR6 |  |
| VPS72 | Vacuolar protein sorting 72 homolog | | | Q15906 |  |
| VRK1 | VRK serine/threonine kinase 1 | | | Q99986 |  |
| VRK2 | VRK serine/threonine kinase 2 | | | Q86Y07 |  |
| VRK3 | VRK serine/threonine kinase 3 | | | Q8IV63 |  |
| VSIG1 | V-set and immunoglobulin domain containing 1 | | | Q86XK7 |  |
| VSIG1 | V-set and immunoglobulin domain containing 1 | | | Q86XK7 |  |
| VTA1 | Vesicle trafficking 1 | | | Q9NP79 |  |
| VTI1A | Vesicle transport through interaction with t-SNAREs 1A | | | Q96AJ9 |  |
| VTI1B | Vesicle transport through interaction with t-SNAREs 1B | | | Q9UEU0 |  |
| VWA5A | Von Willebrand factor A domain containing 5A | | | O00534 |  |
| WAC | WW domain containing adaptor with coiled-coil | | | Q9BTA9 |  |
| WARS2 | Tryptophanyl tRNA synthetase 2, mitochondrial | | | Q9UGM6 |  |
| WASF2 | WASP family member 2 | | | Q9Y6W5 |  |
| WASL | WASP like actin nucleation promoting factor | | | O00401 |  |
| WBP1 | WW domain binding protein 1 | | | Q96G27 |  |
| WBP11 | WW domain binding protein 11 | | | Q9Y2W2 |  |
| WBP1L | WW domain binding protein 1 like | | | Q9NX94 |  |
| WBP2 | WW domain binding protein 2 | | | Q969T9 |  |
| WBP4 | WW domain binding protein 4 | | | O75554 |  |
| WDFY1 | WD repeat and FYVE domain containing 1 | | | Q8IWB7 |  |
| WDFY2 | WD repeat and FYVE domain containing 2 | | | Q96P53 |  |
| WDHD1 | WD repeat and HMG-box DNA binding protein 1 | | | O75717 |  |
| WDR1 | WD repeat domain 1 | | | O75083 |  |
| WDR11 | WD repeat domain 11 | | | Q9BZH6 |  |
| WDR12 | WD repeat domain 12 | | | Q9GZL7 |  |
| WDR13 | WD repeat domain 13 | | | Q9H1Z4 |  |
| WDR18 | WD repeat domain 18 | | | Q9BV38 |  |
| WDR20 | WD repeat domain 20 | | | Q8TBZ3 |  |
| WDR24 | WD repeat domain 24 | | | Q96S15 |  |
| WDR25 | WD repeat domain 25 | | | Q64LD2 |  |
| WDR27 | WD repeat domain 27 | | | A2RRH5 |  |
| WDR3 | WD repeat domain 3 | | | Q9UNX4 |  |
| WDR33 | WD repeat domain 33 | | | Q9C0J8 |  |
| WDR36 | WD repeat domain 36 | | |  |  |
| WDR37 | WD repeat domain 37 | | | Q9Y2I8 |  |
| WDR4 | WD repeat domain 4 | | | P57081 |  |
| WDR41 | WD repeat domain 41 | | | Q9HAD4 |  |
| WDR43 | WD repeat domain 43 | | | Q15061 |  |
| WDR45 | WD repeat domain 45 | | | Q9Y484 |  |
| WDR45B | WD repeat domain 45B | | | Q5MNZ6 |  |
| WDR47 | WD repeat domain 47 | | | O94967 |  |
| WDR48 | WD repeat domain 48 | | | Q8TAF3 |  |
| WDR5 | WD repeat domain 5 | | | P61964 |  |
| WDR53 | WD repeat domain 53 | | | Q7Z5U6 |  |
| WDR54 | WD repeat domain 54 | | | Q9H977 |  |
| WDR55 | WD repeat domain 55 | | | Q9H6Y2 |  |
| WDR59 | WD repeat domain 59 | | | Q6PJI9 |  |
| WDR5B | WD repeat domain 5B | | | Q86VZ2 |  |
| WDR6 | WD repeat domain 6 | | | Q9NNW5 |  |
| WDR70 | WD repeat domain 70 | | | Q9NW82 |  |
| WDR73 | WD repeat domain 73 | | | Q6P4I2 |  |
| WDR74 | WD repeat domain 74 | | | Q6RFH5 |  |
| WDR75 | WD repeat domain 75 | | | Q8IWA0 |  |
| WDR76 | WD repeat domain 76 | | | Q9H967 |  |
| WDR77 | WD repeat domain 77 | | | Q9BQA1 |  |
| WDR82 | WD repeat domain 82 | | | Q6UXN9 |  |
| WDR83 | WD repeat domain 83 | | | Q9BRX9 |  |
| WDR83OS | WD repeat domain 83 opposite strand | | | Q9Y284 |  |
| WDR89 | WD repeat domain 89 | | | Q96FK6 |  |
| WDR91 | WD repeat domain 91 | | | A4D1P6 |  |
| WDSUB1 | WD repeat, sterile alpha motif and U-box domain containing 1 | | | Q8N9V3 |  |
| WEE1 | WEE1 G2 checkpoint kinase | | | P30291 |  |
| WFS1 | Wolframin ER transmembrane glycoprotein | | | O76024 |  |
| WHAMM | WASP homolog associated with actin, golgi membranes and microtubules | | | Q8TF30 |  |
| WIPF1 | WAS/WASL interacting protein family member 1 | | | O43516 |  |
| WIPI1 | WD repeat domain, phosphoinositide interacting 1 | | | Q5MNZ9 |  |
| WIPI2 | WD repeat domain, phosphoinositide interacting 2 | | | Q9Y4P8 |  |
| WNK1 | WNK lysine deficient protein kinase 1 | | | Q9H4A3 |  |
| WNT1 | Wnt family member 1 | | | P04628 |  |
| WNT7A | Wnt family member 7A | | | O00755 |  |
| WRAP53 | WD repeat containing antisense to TP53 | | | Q9BUR4 |  |
| WRAP73 | WD repeat containing, antisense to TP73 | | | Q9P2S5 |  |
| WRNIP1 | WRN helicase interacting protein 1 | | | Q96S55 |  |
| WSB1 | WD repeat and SOCS box containing 1 | | | Q9Y6I7 |  |
| WSB2 | WD repeat and SOCS box containing 2 | | | Q9NYS7 |  |
| WTAP | WT1 associated protein | | | Q15007 |  |
| WWC3 | WWC family member 3 | | | Q9ULE0 |  |
| WWOX | WW domain containing oxidoreductase | | | Q9NZC7 |  |
| WWP1 | WW domain containing E3 ubiquitin protein ligase 1 | | | Q9H0M0 |  |
| WWP2 | WW domain containing E3 ubiquitin protein ligase 2 | | | O00308 |  |
| XAB2 | XPA binding protein 2 | | | Q9HCS7 |  |
| XAF1 | XIAP associated factor 1 | | | Q6GPH4 |  |
| XBP1 | X-box binding protein 1 | | | P17861 |  |
| XCL1 | X-C motif chemokine ligand 1 | | | P47992 |  |
| XCL2 | X-C motif chemokine ligand 2 | | | Q9UBD3 |  |
| XIAP | X-linked inhibitor of apoptosis | | | P98170 |  |
| XKR8 | XK related 8 | | | Q9H6D3 |  |
| XKRX | XK related X-linked | | | Q6PP77 |  |
| XPA | XPA, DNA damage recognition and repair factor | | | P23025 |  |
| XPC | XPC complex subunit, DNA damage recognition and repair factor | | | Q01831 |  |
| XPNPEP1 | X-prolyl aminopeptidase 1 | | | Q9NQW7 |  |
| XPNPEP3 | X-prolyl aminopeptidase 3 | | | Q9NQH7 |  |
| XPO1 | Exportin 1 | | | O14980 |  |
| XPO4 | Exportin 4 | | | Q9C0E2 |  |
| XPO5 | Exportin 5 | | | Q9HAV4 |  |
| XPO7 | Exportin 7 | | | Q9UIA9 |  |
| XPOT | Exportin for tRNA | | | O43592 |  |
| XPR1 | Xenotropic and polytropic retrovirus receptor 1 | | | Q9UBH6 |  |
| XRCC1 | X-ray repair cross complementing 1 | | | P18887 |  |
| XRCC4 | X-ray repair cross complementing 4 | | | Q13426 |  |
| XRCC5 | X-ray repair cross complementing 5 | | | P13010 |  |
| XRCC6 | X-ray repair cross complementing 6 | | | P12956 |  |
| XRN1 | 5'-3' exoribonuclease 1 | | | Q8IZH2 |  |
| XRN2 | 5'-3' exoribonuclease 2 | | | Q9H0D6 |  |
| XRRA1 | X-ray radiation resistance associated 1 | | | Q6P2D8 |  |
| XXYLT1 | Xyloside xylosyltransferase 1 | | | Q8NBI6 |  |
| YAF2 | YY1 associated factor 2 | | | Q8IY57 |  |
| YARS2 | Tyrosyl-tRNA synthetase 2 | | | Q9Y2Z4 |  |
| YBEY | YbeY metalloendoribonuclease | | | P58557 |  |
| YBX1 | Y-box binding protein 1 | | | P67809 |  |
| YEATS2 | YEATS domain containing 2 | | | Q9ULM3 |  |
| YEATS4 | YEATS domain containing 4 | | | O95619 |  |
| YES1 | YES proto-oncogene 1, Src family tyrosine kinase | | | P07947 |  |
| YIF1A | Yip1 interacting factor homolog A, membrane trafficking protein | | | O95070 |  |
| YIF1B | Yip1 interacting factor homolog B, membrane trafficking protein | | | Q5BJH7 |  |
| YIPF1 | Yip1 domain family member 1 | | | Q9Y548 |  |
| YIPF2 | Yip1 domain family member 2 | | | Q9BWQ6 |  |
| YIPF3 | Yip1 domain family member 3 | | | Q9GZM5 |  |
| YIPF4 | Yip1 domain family member 4 | | | Q9BSR8 |  |
| YIPF5 | Yip1 domain family member 5 | | | Q969M3 |  |
| YIPF6 | Yip1 domain family member 6 | | | Q96EC8 |  |
| YKT6 | YKT6 v-SNARE homolog | | | O15498 |  |
| YME1L1 | YME1 like 1 ATPase | | | Q96TA2 |  |
| YPEL2 | Yippee like 2 | | | Q96QA6 |  |
| YPEL3 | Yippee like 3 | | | P61236 |  |
| YPEL5 | Yippee like 5 | | | P62699 |  |
| YRDC | YrdC N6-threonylcarbamoyltransferase domain containing | | | Q86U90 |  |
| YTHDC1 | YTH domain containing 1 | | | Q96MU7 |  |
| YTHDF1 | YTH N6-methyladenosine RNA binding protein 1 | | | Q9BYJ9 |  |
| YTHDF2 | YTH N6-methyladenosine RNA binding protein 2 | | | Q9Y5A9 |  |
| YTHDF3 | YTH N6-methyladenosine RNA binding protein 3 | | | Q7Z739 |  |
| YWHAB | Tyrosine 3-monooxygenase/tryptophan 5-monooxygenase activation protein beta | | | P31946 |  |
| YWHAE | Tyrosine 3-monooxygenase/tryptophan 5-monooxygenase activation protein epsilon | | | P62258 |  |
| YWHAG | Tyrosine 3-monooxygenase/tryptophan 5-monooxygenase activation protein gamma | | | P61981 |  |
| YWHAH | Tyrosine 3-monooxygenase/tryptophan 5-monooxygenase activation protein eta | | | Q04917 |  |
| YWHAQ | Tyrosine 3-monooxygenase/tryptophan 5-monooxygenase activation protein theta | | | P27348 |  |
| YWHAZ | Tyrosine 3-monooxygenase/tryptophan 5-monooxygenase activation protein zeta | | | P63104 |  |
| YY1 | YY1 transcription factor | | | P25490 |  |
| YY1AP1 | YY1 associated protein 1 | | | Q9H869 |  |
| ZAP70 | Zeta chain of T cell receptor associated protein kinase 70 | | | P43403 |  |
| ZBED1 | Zinc finger BED-type containing 1 | | | O96006 |  |
| ZBED4 | Zinc finger BED-type containing 4 | | | O75132 |  |
| ZBED5 | Zinc finger BED-type containing 5 | | | Q49AG3 |  |
| ZBP1 | Z-DNA binding protein 1 | | | Q9H171 |  |
| ZBTB1 | Zinc finger and BTB domain containing 1 | | | Q9Y2K1 |  |
| ZBTB11 | Zinc finger and BTB domain containing 11 | | | O95625 |  |
| ZBTB14 | Zinc finger and BTB domain containing 14 | | | O43829 |  |
| ZBTB16 | Zinc finger and BTB domain containing 16 | | | Q05516 |  |
| ZBTB17 | Zinc finger and BTB domain containing 17 | | | Q13105 |  |
| ZBTB2 | Zinc finger and BTB domain containing 2 | | | Q8N680 |  |
| ZBTB20 | Zinc finger and BTB domain containing 20 | | | Q9HC78 |  |
| ZBTB21 | Zinc finger and BTB domain containing 21 | | | Q9ULJ3 |  |
| ZBTB24 | Zinc finger and BTB domain containing 24 | | | O43167 |  |
| ZBTB25 | Zinc finger and BTB domain containing 25 | | | P24278 |  |
| ZBTB3 | Zinc finger and BTB domain containing 3 | | | Q9H5J0 |  |
| ZBTB33 | Zinc finger and BTB domain containing 33 | | | Q86T24 |  |
| ZBTB38 | Zinc finger and BTB domain containing 38 | | | Q8NAP3 |  |
| ZBTB4 | Zinc finger and BTB domain containing 4 | | | Q9P1Z0 |  |
| ZBTB43 | Zinc finger and BTB domain containing 43 | | | O43298 |  |
| ZBTB44 | Zinc finger and BTB domain containing 44 | | | Q8NCP5 |  |
| ZBTB48 | Zinc finger and BTB domain containing 48 | | | P10074 |  |
| ZBTB49 | Zinc finger and BTB domain containing 49 | | | Q6ZSB9 |  |
| ZBTB6 | Zinc finger and BTB domain containing 6 | | | Q15916 |  |
| ZBTB8OS | Zinc finger and BTB domain containing 8 opposite strand | | | Q8IWT0 |  |
| ZBTB9 | Zinc finger and BTB domain containing 9 | | | Q96C00 |  |
| ZC2HC1A | Zinc finger C2HC-type containing 1A | | | Q96GY0 |  |
| ZC3H10 | Zinc finger CCCH-type containing 10 | | | Q96K80 |  |
| ZC3H11A | Zinc finger CCCH-type containing 11A | | | O75152 |  |
| ZC3H12A | Zinc finger CCCH-type containing 12A | | | Q5D1E8 |  |
| ZC3H12D | Zinc finger CCCH-type containing 12D | | | A2A288 |  |
| ZC3H13 | Zinc finger CCCH-type containing 13 | | | Q5T200 |  |
| ZC3H14 | Zinc finger CCCH-type containing 14 | | | Q6PJT7 |  |
| ZC3H15 | Zinc finger CCCH-type containing 15 | | | Q8WU90 |  |
| ZC3H18 | Zinc finger CCCH-type containing 18 | | | Q86VM9 |  |
| ZC3H3 | Zinc finger CCCH-type containing 3 | | | Q8IXZ2 |  |
| ZC3H7A | Zinc finger CCCH-type containing 7A | | | Q8IWR0 |  |
| ZC3H8 | Zinc finger CCCH-type containing 8 | | | Q8N5P1 |  |
| ZC3HAV1 | Zinc finger CCCH-type containing, antiviral 1 | | | Q7Z2W4 |  |
| ZC3HC1 | Zinc finger C3HC-type containing 1 | | | Q86WB0 |  |
| ZC4H2 | Zinc finger C4H2-type containing | | | Q9NQZ6 |  |
| ZCCHC10 | Zinc finger CCHC-type containing 10 | | | Q8TBK6 |  |
| ZCCHC17 | Zinc finger CCHC-type containing 17 | | | Q9NP64 |  |
| ZCCHC3 | Zinc finger CCHC-type containing 3 | | | Q9NUD5 |  |
| ZCCHC4 | Zinc finger CCHC-type containing 4 | | | Q9H5U6 |  |
| ZCCHC7 | Zinc finger CCHC-type containing 7 | | | Q8N3Z6 |  |
| ZCCHC8 | Zinc finger CCHC-type containing 8 | | | Q6NZY4 |  |
| ZCCHC9 | Zinc finger CCHC-type containing 9 | | | Q8N567 |  |
| ZCRB1 | Zinc finger CCHC-type and RNA binding motif containing 1 | | | Q8TBF4 |  |
| ZCWPW1 | Zinc finger CW-type and PWWP domain containing 1 | | | Q9H0M4 |  |
| ZDHHC12 | Zinc finger DHHC-type palmitoyltransferase 12 | | | Q96GR4 |  |
| ZDHHC13 | Zinc finger DHHC-type palmitoyltransferase 13 | | | Q8IUH4 |  |
| ZDHHC16 | Zinc finger DHHC-type palmitoyltransferase 16 | | | Q969W1 |  |
| ZDHHC17 | Zinc finger DHHC-type palmitoyltransferase 17 | | | Q8IUH5 |  |
| ZDHHC20 | Zinc finger DHHC-type palmitoyltransferase 20 | | | Q5W0Z9 |  |
| ZDHHC3 | Zinc finger DHHC-type palmitoyltransferase 3 | | | Q9NYG2 |  |
| ZDHHC4 | Zinc finger DHHC-type palmitoyltransferase 4 | | | Q9NPG8 |  |
| ZDHHC5 | Zinc finger DHHC-type palmitoyltransferase 5 | | | Q9C0B5 |  |
| ZDHHC6 | Zinc finger DHHC-type palmitoyltransferase 6 | | | Q9H6R6 |  |
| ZDHHC7 | Zinc finger DHHC-type palmitoyltransferase 7 | | | Q9NXF8 |  |
| ZDHHC9 | Zinc finger DHHC-type palmitoyltransferase 9 | | | Q9Y397 |  |
| ZEB2 | Zinc finger E-box binding homeobox 2 | | | O60315 |  |
| ZER1 | Zyg-11 related cell cycle regulator | | | Q7Z7L7 |  |
| ZFAND1 | Zinc finger AN1-type containing 1 | | | Q8TCF1 |  |
| ZFAND2A | Zinc finger AN1-type containing 2A | | | Q8N6M9 |  |
| ZFAND2B | Zinc finger AN1-type containing 2B | | | Q8WV99 |  |
| ZFAND3 | Zinc finger AN1-type containing 3 | | | Q9H8U3 |  |
| ZFAND5 | Zinc finger AN1-type containing 5 | | | O76080 |  |
| ZFAND6 | Zinc finger AN1-type containing 6 | | | Q6FIF0 |  |
| ZFC3H1 | Zinc finger C3H1-type containing | | | O60293 |  |
| ZFP1 | ZFP1 zinc finger protein | | | Q6P2D0 |  |
| ZFP14 | ZFP14 zinc finger protein | | | Q9HCL3 |  |
| ZFP3 | ZFP3 zinc finger protein | | | Q96NJ6 |  |
| ZFP30 | ZFP30 zinc finger protein | | | Q9Y2G7 |  |
| ZFP36 | ZFP36 ring finger protein | | | P26651 |  |
| ZFP36L1 | ZFP36 ring finger protein like 1 | | | Q07352 |  |
| ZFP36L2 | ZFP36 ring finger protein like 2 | | | P47974 |  |
| ZFP64 | ZFP64 zinc finger protein | | | Q9NTW7 |  |
| ZFP69B | ZFP69 zinc finger protein B | | | Q9UJL9 |  |
| ZFP82 | ZFP82 zinc finger protein | | | Q8N141 |  |
| ZFP91 | ZFP91 zinc finger protein, atypical E3 ubiquitin ligase | | | Q96JP5 |  |
| ZFPL1 | Zinc finger protein like 1 | | | O95159 |  |
| ZFY | Zinc finger protein Y-linked | | | P08048 |  |
| ZFYVE1 | Zinc finger FYVE-type containing 1 | | | Q9HBF4 |  |
| ZFYVE19 | Zinc finger FYVE-type containing 19 | | | Q96K21 |  |
| ZFYVE21 | Zinc finger FYVE-type containing 21 | | | Q9BQ24 |  |
| ZFYVE27 | Zinc finger FYVE-type containing 27 | | | Q5T4F4 |  |
| ZFYVE28 | Zinc finger FYVE-type containing 28 | | | Q9HCC9 |  |
| ZGPAT | Zinc finger CCCH-type and G-patch domain containing | | | Q8N5A5 |  |
| ZHX1 | Zinc fingers and homeoboxes 1 | | | Q9UKY1 |  |
| ZHX1-C8orf76 | ZHX1-C8orf76 readthrough | | | Q96EF9 |  |
| ZHX2 | Zinc fingers and homeoboxes 2 | | | Q9Y6X8 |  |
| ZIK1 | Zinc finger protein interacting with K protein 1 | | | Q3SY52 |  |
| ZKSCAN1 | Zinc finger with KRAB and SCAN domains 1 | | | P17029 |  |
| ZKSCAN3 | Zinc finger with KRAB and SCAN domains 3 | | | Q9BRR0 |  |
| ZKSCAN4 | Zinc finger with KRAB and SCAN domains 4 | | | Q969J2 |  |
| ZKSCAN5 | Zinc finger with KRAB and SCAN domains 5 | | | Q9Y2L8 |  |
| ZKSCAN8 | Zinc finger with KRAB and SCAN domains 8 | | | Q15776 |  |
| ZMAT2 | Zinc finger matrin-type 2 | | | Q96NC0 |  |
| ZMAT3 | Zinc finger matrin-type 3 | | | Q9HA38 |  |
| ZMAT5 | Zinc finger matrin-type 5 | | | Q9UDW3 |  |
| ZMPSTE24 | Zinc metallopeptidase STE24 | | | O75844 |  |
| ZMYM2 | Zinc finger MYM-type containing 2 | | | Q9UBW7 |  |
| ZMYM3 | Zinc finger MYM-type containing 3 | | | Q14202 |  |
| ZMYM4 | Zinc finger MYM-type containing 4 | | | Q5VZL5 |  |
| ZMYM5 | Zinc finger MYM-type containing 5 | | | Q9UJ78 |  |
| ZMYM6 | Zinc finger MYM-type containing 6 | | | O95789 |  |
| ZMYND11 | Zinc finger MYND-type containing 11 | | | Q15326 |  |
| ZMYND8 | Zinc finger MYND-type containing 8 | | | Q9ULU4 |  |
| ZNF10 | Zinc finger protein 10 | | | P21506 |  |
| ZNF101 | Zinc finger protein 101 | | | Q8IZC7 |  |
| ZNF106 | Zinc finger protein 106 | | | Q9H2Y7 |  |
| ZNF121 | Zinc finger protein 121 | | | P58317 |  |
| ZNF124 | Zinc finger protein 124 | | | Q15973 |  |
| ZNF131 | Zinc finger protein 131 | | | P52739 |  |
| ZNF132 | Zinc finger protein 132 | | | P52740 |  |
| ZNF133 | Zinc finger protein 133 | | | P52736 |  |
| ZNF134 | Zinc finger protein 134 | | | P52741 |  |
| ZNF135 | Zinc finger protein 135 | | | P52742 |  |
| ZNF136 | Zinc finger protein 136 | | | P52737 |  |
| ZNF138 | Zinc finger protein 138 | | | P52744 |  |
| ZNF14 | Zinc finger protein 14 | | | P17017 |  |
| ZNF140 | Zinc finger protein 140 | | | P52738 |  |
| ZNF143 | Zinc finger protein 143 | | | P52747 |  |
| ZNF146 | Zinc finger protein 146 | | | Q15072 |  |
| ZNF148 | Zinc finger protein 148 | | | Q9UQR1 |  |
| ZNF155 | Zinc finger protein 155 | | | Q12901 |  |
| ZNF16 | Zinc finger protein 16 | | | P17020 |  |
| ZNF160 | Zinc finger protein 160 | | | Q9HCG1 |  |
| ZNF165 | Zinc finger protein 165 | | | P49910 |  |
| ZNF169 | Zinc finger protein 169 | | | Q14929 |  |
| ZNF17 | Zinc finger protein 17 | | | P17021 |  |
| ZNF174 | Zinc finger protein 174 | | | Q15697 |  |
| ZNF18 | Zinc finger protein 18 | | | P17022 |  |
| ZNF180 | Zinc finger protein 180 | | | Q9UJW8 |  |
| ZNF181 | Zinc finger protein 181 | | | Q2M3W8 |  |
| ZNF182 | Zinc finger protein 182 | | | P17025 |  |
| ZNF184 | Zinc finger protein 184 | | | Q99676 |  |
| ZNF185 | Zinc finger protein 185 with LIM domain | | | O15231 |  |
| ZNF189 | Zinc finger protein 189 | | | O75820 |  |
| ZNF19 | Zinc finger protein 19 | | | P17023 |  |
| ZNF195 | Zinc finger protein 195 | | | O14628 |  |
| ZNF2 | Zinc finger protein 2 | | | Q9BSG1 |  |
| ZNF20 | Zinc finger protein 20 | | | P17024 |  |
| ZNF200 | Zinc finger protein 200 | | | P98182 |  |
| ZNF202 | Zinc finger protein 202 | | | O95125 |  |
| ZNF207 | Zinc finger protein 207 | | | O43670 |  |
| ZNF212 | Zinc finger protein 212 | | | Q9UDV6 |  |
| ZNF213 | Zinc finger protein 213 | | | O14771 |  |
| ZNF217 | Zinc finger protein 217 | | | O75362 |  |
| ZNF22 | Zinc finger protein 22 | | | P17026 |  |
| ZNF222 | Zinc finger protein 222 | | | Q9UK12 |  |
| ZNF223 | Zinc finger protein 223 | | | Q9UK11 |  |
| ZNF224 | Zinc finger protein 224 | | | Q9NZL3 |  |
| ZNF225 | Zinc finger protein 225 | | | Q9UK10 |  |
| ZNF226 | Zinc finger protein 226 | | | Q9NYT6 |  |
| ZNF23 | Zinc finger protein 23 | | | P17027 |  |
| ZNF230 | Zinc finger protein 230 | | | Q9UIE0 |  |
| ZNF232 | Zinc finger protein 232 | | | Q9UNY5 |  |
| ZNF234 | Zinc finger protein 234 | | | Q14588 |  |
| ZNF235 | Zinc finger protein 235 | | | Q14590 |  |
| ZNF239 | Zinc finger protein 239 | | | Q16600 |  |
| ZNF24 | Zinc finger protein 24 | | | P17028 |  |
| ZNF248 | Zinc finger protein 248 | | | Q8NDW4 |  |
| ZNF25 | Zinc finger protein 25 | | | P17030 |  |
| ZNF250 | Zinc finger protein 250 | | | P15622 |  |
| ZNF253 | Zinc finger protein 253 | | | O75346 |  |
| ZNF254 | Zinc finger protein 254 | | | O75437 |  |
| ZNF256 | Zinc finger protein 256 | | | Q9Y2P7 |  |
| ZNF257 | Zinc finger protein 257 | | | Q9Y2Q1 |  |
| ZNF26 | Zinc finger protein 26 | | | P17031 |  |
| ZNF260 | Zinc finger protein 260 | | | Q3ZCT1 |  |
| ZNF263 | Zinc finger protein 263 | | | O14978 |  |
| ZNF264 | Zinc finger protein 264 | | | O43296 |  |
| ZNF266 | Zinc finger protein 266 | | | Q14584 |  |
| ZNF267 | Zinc finger protein 267 | | | Q14586 |  |
| ZNF268 | Zinc finger protein 268 | | | Q14587 |  |
| ZNF274 | Zinc finger protein 274 | | | Q96GC6 |  |
| ZNF276 | Zinc finger protein 276 | | | Q8N554 |  |
| ZNF277 | Zinc finger protein 277 | | | Q9NRM2 |  |
| ZNF28 | Zinc finger protein 28 | | | P17035 |  |
| ZNF280B | Zinc finger protein 280B | | | Q86YH2 |  |
| ZNF280C | Zinc finger protein 280C | | | Q8ND82 |  |
| ZNF280D | Zinc finger protein 280D | | | Q6N043 |  |
| ZNF281 | Zinc finger protein 281 | | | Q9Y2X9 |  |
| ZNF283 | Zinc finger protein 283 | | | Q8N7M2 |  |
| ZNF284 | Zinc finger protein 284 | | | Q2VY69 |  |
| ZNF285 | Zinc finger protein 285 | | | Q96NJ3 |  |
| ZNF287 | Zinc finger protein 287 | | | Q9HBT7 |  |
| ZNF292 | Zinc finger protein 292 | | | O60281 |  |
| ZNF296 | Zinc finger protein 296 | | | Q8WUU4 |  |
| ZNF3 | Zinc finger protein 3 | | | P17036 |  |
| ZNF30 | Zinc finger protein 30 | | | P17039 |  |
| ZNF302 | Zinc finger protein 302 | | | Q9NR11 |  |
| ZNF304 | Zinc finger protein 304 | | | Q9HCX3 |  |
| ZNF317 | Zinc finger protein 317 | | | Q96PQ6 |  |
| ZNF319 | Zinc finger protein 319 | | | Q9P2F9 |  |
| ZNF32 | Zinc finger protein 32 | | | P17041 |  |
| ZNF320 | Zinc finger protein 320 | | | A2RRD8 |  |
| ZNF322 | Zinc finger protein 322 | | | Q6U7Q0 |  |
| ZNF324 | Zinc finger protein 324 | | | O75467 |  |
| ZNF329 | Zinc finger protein 329 | | | Q86UD4 |  |
| ZNF330 | Zinc finger protein 330 | | | Q9Y3S2 |  |
| ZNF331 | Zinc finger protein 331 | | | Q9NQX6 |  |
| ZNF333 | Zinc finger protein 333 | | | Q96JL9 |  |
| ZNF335 | Zinc finger protein 335 | | | Q9H4Z2 |  |
| ZNF337 | Zinc finger protein 337 | | | Q9Y3M9 |  |
| ZNF33A | Zinc finger protein 33A | | | Q06730 |  |
| ZNF34 | Zinc finger protein 34 | | | Q8IZ26 |  |
| ZNF343 | Zinc finger protein 343 | | | Q6P1L6 |  |
| ZNF345 | Zinc finger protein 345 | | | Q14585 |  |
| ZNF346 | Zinc finger protein 346 | | | Q9UL40 |  |
| ZNF350 | Zinc finger protein 350 | | | Q9GZX5 |  |
| ZNF354A | Zinc finger protein 354A | | | O60765 |  |
| ZNF354B | Zinc finger protein 354B | | | Q96LW1 |  |
| ZNF362 | Zinc finger protein 362 | | | Q5T0B9 |  |
| ZNF365 | Zinc finger protein 365 | | | Q70YC5 |  |
| ZNF37A | Zinc finger protein 37A | | | P17032 |  |
| ZNF382 | Zinc finger protein 382 | | | Q96SR6 |  |
| ZNF383 | Zinc finger protein 383 | | | Q8NA42 |  |
| ZNF384 | Zinc finger protein 384 | | | Q8TF68 |  |
| ZNF391 | Zinc finger protein 391 | | | Q9UJN7 |  |
| ZNF394 | Zinc finger protein 394 | | | Q53GI3 |  |
| ZNF395 | Zinc finger protein 395 | | | Q9H8N7 |  |
| ZNF396 | Zinc finger protein 396 | | | Q96N95 |  |
| ZNF397 | Zinc finger protein 397 | | | Q8NF99 |  |
| ZNF398 | Zinc finger protein 398 | | | Q8TD17 |  |
| ZNF408 | Zinc finger protein 408 | | | Q9H9D4 |  |
| ZNF41 | Zinc finger protein 41 | | | P51814 |  |
| ZNF410 | Zinc finger protein 410 | | | Q86VK4 |  |
| ZNF415 | Zinc finger protein 415 | | | Q09FC8 |  |
| ZNF416 | Zinc finger protein 416 | | | Q9BWM5 |  |
| ZNF417 | Zinc finger protein 417 | | | Q8TAU3 |  |
| ZNF418 | Zinc finger protein 418 | | | Q8TF45 |  |
| ZNF420 | Zinc finger protein 420 | | | Q8TAQ5 |  |
| ZNF425 | Zinc finger protein 425 | | | Q6IV72 |  |
| ZNF426 | Zinc finger protein 426 | | | Q9BUY5 |  |
| ZNF428 | Zinc finger protein 428 | | | Q96B54 |  |
| ZNF43 | Zinc finger protein 43 | | | P17038 |  |
| ZNF430 | Zinc finger protein 430 | | | Q9H8G1 |  |
| ZNF431 | Zinc finger protein 431 | | | Q8TF32 |  |
| ZNF432 | Zinc finger protein 432 | | | O94892 |  |
| ZNF433 | Zinc finger protein 433 | | | Q8N7K0 |  |
| ZNF438 | Zinc finger protein 438 | | | Q7Z4V0 |  |
| ZNF44 | Zinc finger protein 44 | | | P15621 |  |
| ZNF440 | Zinc finger protein 440 | | | Q8IYI8 |  |
| ZNF441 | Zinc finger protein 441 | | | Q8N8Z8 |  |
| ZNF443 | Zinc finger protein 443 | | | Q9Y2A4 |  |
| ZNF444 | Zinc finger protein 444 | | | Q8N0Y2 |  |
| ZNF446 | Zinc finger protein 446 | | | Q9NWS9 |  |
| ZNF449 | Zinc finger protein 449 | | | Q6P9G9 |  |
| ZNF45 | Zinc finger protein 45 | | | Q02386 |  |
| ZNF451 | Zinc finger protein 451 | | | Q9Y4E5 |  |
| ZNF460 | Zinc finger protein 460 | | | Q14592 |  |
| ZNF461 | Zinc finger protein 461 | | | Q8TAF7 |  |
| ZNF468 | Zinc finger protein 468 | | | Q5VIY5 |  |
| ZNF473 | Zinc finger protein 473 | | | Q8WTR7 |  |
| ZNF480 | Zinc finger protein 480 | | | Q8WV37 |  |
| ZNF483 | Zinc finger protein 483 | | | Q8TF39 |  |
| ZNF485 | Zinc finger protein 485 | | | Q8NCK3 |  |
| ZNF486 | Zinc finger protein 486 | | | Q96H40 |  |
| ZNF490 | Zinc finger protein 490 | | | Q9ULM2 |  |
| ZNF493 | Zinc finger protein 493 | | | Q6ZR52 |  |
| ZNF497 | Zinc finger protein 497 | | | Q6ZNH5 |  |
| ZNF502 | Zinc finger protein 502 | | | Q8TBZ5 |  |
| ZNF506 | Zinc finger protein 506 | | | Q5JVG8 |  |
| ZNF507 | Zinc finger protein 507 | | | Q8TCN5 |  |
| ZNF510 | Zinc finger protein 510 | | | Q9Y2H8 |  |
| ZNF511 | Zinc finger protein 511 | | | Q8NB15 |  |
| ZNF512 | Zinc finger protein 512 | | | Q96ME7 |  |
| ZNF514 | Zinc finger protein 514 | | | Q96K75 |  |
| ZNF518A | Zinc finger protein 518A | | | Q6AHZ1 |  |
| ZNF524 | Zinc finger protein 524 | | | Q96C55 |  |
| ZNF526 | Zinc finger protein 526 | | | Q8TF50 |  |
| ZNF527 | Zinc finger protein 527 | | | Q8NB42 |  |
| ZNF528 | Zinc finger protein 528 | | | Q3MIS6 |  |
| ZNF529 | Zinc finger protein 529 | | | Q6P280 |  |
| ZNF530 | Zinc finger protein 530 | | | Q6P9A1 |  |
| ZNF540 | Zinc finger protein 540 | | | Q8NDQ6 |  |
| ZNF543 | Zinc finger protein 543 | | | Q08ER8 |  |
| ZNF544 | Zinc finger protein 544 | | | Q6NX49 |  |
| ZNF546 | Zinc finger protein 546 | | | Q86UE3 |  |
| ZNF547 | Zinc finger protein 547 | | | Q8IVP9 |  |
| ZNF548 | Zinc finger protein 548 | | | Q8NEK5 |  |
| ZNF549 | Zinc finger protein 549 | | | Q6P9A3 |  |
| ZNF550 | Zinc finger protein 550 | | |  |  |
| ZNF551 | Zinc finger protein 551 | | | Q7Z340 |  |
| ZNF552 | Zinc finger protein 552 | | | Q9H707 |  |
| ZNF555 | Zinc finger protein 555 | | | Q8NEP9 |  |
| ZNF557 | Zinc finger protein 557 | | | Q8N988 |  |
| ZNF558 | Zinc finger protein 558 | | | Q96NG5 |  |
| ZNF559 | Zinc finger protein 559 | | | Q9BR84 |  |
| ZNF561 | Zinc finger protein 561 | | | Q8N587 |  |
| ZNF562 | Zinc finger protein 562 | | | Q6V9R5 |  |
| ZNF563 | Zinc finger protein 563 | | | Q8TA94 |  |
| ZNF564 | Zinc finger protein 564 | | | Q8TBZ8 |  |
| ZNF565 | Zinc finger protein 565 | | | Q8N9K5 |  |
| ZNF566 | Zinc finger protein 566 | | | Q969W8 |  |
| ZNF567 | Zinc finger protein 567 | | | Q8N184 |  |
| ZNF57 | Zinc finger protein 57 | | | Q68EA5 |  |
| ZNF571 | Zinc finger protein 571 | | | Q7Z3V5 |  |
| ZNF573 | Zinc finger protein 573 | | | Q86YE8 |  |
| ZNF574 | Zinc finger protein 574 | | | Q6ZN55 |  |
| ZNF575 | Zinc finger protein 575 | | | Q86XF7 |  |
| ZNF576 | Zinc finger protein 576 | | | Q9H609 |  |
| ZNF577 | Zinc finger protein 577 | | | Q9BSK1 |  |
| ZNF580 | Zinc finger protein 580 | | | Q9UK33 |  |
| ZNF581 | Zinc finger protein 581 | | | Q9P0T4 |  |
| ZNF582 | Zinc finger protein 582 | | | Q96NG8 |  |
| ZNF583 | Zinc finger protein 583 | | | Q96ND8 |  |
| ZNF584 | Zinc finger protein 584 | | | Q8IVC4 |  |
| ZNF585A | Zinc finger protein 585A | | | Q6P3V2 |  |
| ZNF585B | Zinc finger protein 585B | | | Q52M93 |  |
| ZNF586 | Zinc finger protein 586 | | | Q9NXT0 |  |
| ZNF589 | Zinc finger protein 589 | | | Q86UQ0 |  |
| ZNF592 | Zinc finger protein 592 | | | Q92610 |  |
| ZNF593 | Zinc finger protein 593 | | | O00488 |  |
| ZNF595 | Zinc finger protein 595 | | | Q8IYB9 |  |
| ZNF596 | Zinc finger protein 596 | | | Q8TC21 |  |
| ZNF597 | Zinc finger protein 597 | | | Q96LX8 |  |
| ZNF599 | Zinc finger protein 599 | | | Q96NL3 |  |
| ZNF606 | Zinc finger protein 606 | | | Q8WXB4 |  |
| ZNF607 | Zinc finger protein 607 | | | Q96SK3 |  |
| ZNF610 | Zinc finger protein 610 | | | Q8N9Z0 |  |
| ZNF613 | Zinc finger protein 613 | | | Q6PF04 |  |
| ZNF614 | Zinc finger protein 614 | | | Q8N883 |  |
| ZNF615 | Zinc finger protein 615 | | | Q8N8J6 |  |
| ZNF620 | Zinc finger protein 620 | | | Q6ZNG0 |  |
| ZNF621 | Zinc finger protein 621 | | | Q6ZSS3 |  |
| ZNF622 | Zinc finger protein 622 | | | Q969S3 |  |
| ZNF624 | Zinc finger protein 624 | | | Q9P2J8 |  |
| ZNF626 | Zinc finger protein 626 | | | Q68DY1 |  |
| ZNF627 | Zinc finger protein 627 | | | Q7L945 |  |
| ZNF638 | Zinc finger protein 638 | | | Q14966 |  |
| ZNF639 | Zinc finger protein 639 | | | Q9UID6 |  |
| ZNF641 | Zinc finger protein 641 | | | Q96N77 |  |
| ZNF644 | Zinc finger protein 644 | | | Q9H582 |  |
| ZNF646 | Zinc finger protein 646 | | | O15015 |  |
| ZNF649 | Zinc finger protein 649 | | | Q9BS31 |  |
| ZNF652 | Zinc finger protein 652 | | | Q9Y2D9 |  |
| ZNF654 | Zinc finger protein 654 | | | Q8IZM8 |  |
| ZNF655 | Zinc finger protein 655 | | | Q8N720 |  |
| ZNF658 | Zinc finger protein 658 | | | Q5TYW1 |  |
| ZNF662 | Zinc finger protein 662 | | | Q6ZS27 |  |
| ZNF668 | Zinc finger protein 668 | | | Q96K58 |  |
| ZNF669 | Zinc finger protein 669 | | | Q96BR6 |  |
| ZNF670 | Zinc finger protein 670 | | | Q9BS34 |  |
| ZNF671 | Zinc finger protein 671 | | | Q8TAW3 |  |
| ZNF672 | Zinc finger protein 672 | | | Q499Z4 |  |
| ZNF675 | Zinc finger protein 675 | | | Q8TD23 |  |
| ZNF677 | Zinc finger protein 677 | | | Q86XU0 |  |
| ZNF680 | Zinc finger protein 680 | | | Q8NEM1 |  |
| ZNF681 | Zinc finger protein 681 | | | Q96N22 |  |
| ZNF682 | Zinc finger protein 682 | | | O95780 |  |
| ZNF683 | Zinc finger protein 683 | | | Q8IZ20 |  |
| ZNF684 | Zinc finger protein 684 | | | Q5T5D7 |  |
| ZNF688 | Zinc finger protein 688 | | | P0C7X2 |  |
| ZNF689 | Zinc finger protein 689 | | | Q96CS4 |  |
| ZNF69 | Zinc finger protein 69 | | | Q9UC07 |  |
| ZNF691 | Zinc finger protein 691 | | | Q5VV52 |  |
| ZNF692 | Zinc finger protein 692 | | | Q9BU19 |  |
| ZNF696 | Zinc finger protein 696 | | | Q9H7X3 |  |
| ZNF699 | Zinc finger protein 699 | | | Q32M78 |  |
| ZNF7 | Zinc finger protein 7 | | | P17097 |  |
| ZNF701 | Zinc finger protein 701 | | | Q9NV72 |  |
| ZNF706 | Zinc finger protein 706 | | | Q9Y5V0 |  |
| ZNF708 | Zinc finger protein 708 | | | P17019 |  |
| ZNF709 | Zinc finger protein 709 | | | Q8N972 |  |
| ZNF71 | Zinc finger protein 71 | | | Q9NQZ8 |  |
| ZNF714 | Zinc finger protein 714 | | |  |  |
| ZNF718 | Zinc finger protein 718 | | | Q3SXZ3 |  |
| ZNF726 | Zinc finger protein 726 | | | A6NNF4 |  |
| ZNF738 | Zinc finger protein 738 | | | Q8NE65 |  |
| ZNF74 | Zinc finger protein 74 | | | Q16587 |  |
| ZNF747 | Zinc finger protein 747 | | | Q9BV97 |  |
| ZNF749 | Zinc finger protein 749 | | | O43361 |  |
| ZNF75A | Zinc finger protein 75a | | | Q96N20 |  |
| ZNF75D | Zinc finger protein 75D | | | P51815 |  |
| ZNF76 | Zinc finger protein 76 | | | P36508 |  |
| ZNF761 | Zinc finger protein 761 | | | Q86XN6 |  |
| ZNF763 | Zinc finger protein 763 | | | Q0D2J5 |  |
| ZNF764 | Zinc finger protein 764 | | | Q96H86 |  |
| ZNF765 | Zinc finger protein 765 | | | Q7L2R6 |  |
| ZNF766 | Zinc finger protein 766 | | | Q5HY98 |  |
| ZNF77 | Zinc finger protein 77 | | | Q15935 |  |
| ZNF770 | Zinc finger protein 770 | | | Q6IQ21 |  |
| ZNF771 | Zinc finger protein 771 | | | Q7L3S4 |  |
| ZNF772 | Zinc finger protein 772 | | | Q68DY9 |  |
| ZNF773 | Zinc finger protein 773 | | | Q6PK81 |  |
| ZNF776 | Zinc finger protein 776 | | | Q68DI1 |  |
| ZNF778 | Zinc finger protein 778 | | | Q96MU6 |  |
| ZNF780A | Zinc finger protein 780A | | | O75290 |  |
| ZNF785 | Zinc finger protein 785 | | | A8K8V0 |  |
| ZNF786 | Zinc finger protein 786 | | | Q8N393 |  |
| ZNF789 | Zinc finger protein 789 | | | Q5FWF6 |  |
| ZNF79 | Zinc finger protein 79 | | | Q15937 |  |
| ZNF790 | Zinc finger protein 790 | | | Q6PG37 |  |
| ZNF791 | Zinc finger protein 791 | | | Q3KP31 |  |
| ZNF792 | Zinc finger protein 792 | | | Q3KQV3 |  |
| ZNF793 | Zinc finger protein 793 | | | Q6ZN11 |  |
| ZNF800 | Zinc finger protein 800 | | | Q2TB10 |  |
| ZNF808 | Zinc finger protein 808 | | | Q8N4W9 |  |
| ZNF81 | Zinc finger protein 81 | | | P51508 |  |
| ZNF814 | Zinc finger protein 814 | | | B7Z6K7 |  |
| ZNF816 | Zinc finger protein 816 | | | Q0VGE8 |  |
| ZNF821 | Zinc finger protein 821 | | | O75541 |  |
| ZNF823 | Zinc finger protein 823 | | | P16415 |  |
| ZNF829 | Zinc finger protein 829 | | | Q3KNS6 |  |
| ZNF83 | Zinc finger protein 83 | | | P51522 |  |
| ZNF830 | Zinc finger protein 830 | | | Q96NB3 |  |
| ZNF835 | Zinc finger protein 835 | | | Q9Y2P0 |  |
| ZNF84 | Zinc finger protein 84 | | | P51523 |  |
| ZNF846 | Zinc finger protein 846 | | | Q147U1 |  |
| ZNF85 | Zinc finger protein 85 | | | Q03923 |  |
| ZNF880 | Zinc finger protein 880 | | | Q6PDB4 |  |
| ZNF92 | Zinc finger protein 92 | | | Q03936 |  |
| ZNF93 | Zinc finger protein 93 | | | P35789 |  |
| ZNFX1 | Zinc finger NFX1-type containing 1 | | | Q9P2E3 |  |
| ZNHIT1 | Zinc finger HIT-type containing 1 | | | O43257 |  |
| ZNHIT2 | Zinc finger HIT-type containing 2 | | | Q9UHR6 |  |
| ZNHIT3 | Zinc finger HIT-type containing 3 | | | Q15649 |  |
| ZNHIT6 | Zinc finger HIT-type containing 6 | | | Q9NWK9 |  |
| ZP3 | Zona pellucida glycoprotein 3 | | | P21754 |  |
| ZPR1 | ZPR1 zinc finger | | | O75312 |  |
| ZRANB1 | Zinc finger RANBP2-type containing 1 | | | Q9UGI0 |  |
| ZRANB2 | Zinc finger RANBP2-type containing 2 | | | O95218 |  |
| ZRSR2 | Zinc finger CCCH-type, RNA binding motif and serine/arginine rich 2 | | | Q15696 |  |
| ZSCAN12 | Zinc finger and SCAN domain containing 12 | | | O43309 |  |
| ZSCAN16 | Zinc finger and SCAN domain containing 16 | | | Q9H4T2 |  |
| ZSCAN2 | Zinc finger and SCAN domain containing 2 | | | Q7Z7L9 |  |
| ZSCAN21 | Zinc finger and SCAN domain containing 21 | | | Q9Y5A6 |  |
| ZSCAN22 | Zinc finger and SCAN domain containing 22 | | | P10073 |  |
| ZSCAN25 | Zinc finger and SCAN domain containing 25 | | | Q6NSZ9 |  |
| ZSCAN26 | Zinc finger and SCAN domain containing 26 | | | Q16670 |  |
| ZSCAN30 | Zinc finger and SCAN domain containing 30 | | | Q86W11 |  |
| ZSCAN31 | Zinc finger and SCAN domain containing 31 | | | Q96LW9 |  |
| ZSCAN32 | Zinc finger and SCAN domain containing 32 | | | Q9NX65 |  |
| ZSCAN5A | Zinc finger and SCAN domain containing 5A | | | Q9BUG6 |  |
| ZSCAN9 | Zinc finger and SCAN domain containing 9 | | | O15535 |  |
| ZSWIM1 | Zinc finger SWIM-type containing 1 | | | Q9BR11 |  |
| ZSWIM7 | Zinc finger SWIM-type containing 7 | | | Q19AV6 |  |
| ZW10 | Zw10 kinetochore protein | | | O43264 |  |
| ZWILCH | Zwilch kinetochore protein | | | Q9H900 |  |
| ZWINT | ZW10 interacting kinetochore protein | | | O95229 |  |
| ZYX | Zyxin | | | Q15942 |  |
| ZZZ3 | Zinc finger ZZ-type containing 3 | | | Q8IYH5 |  |
|  | |  |  | | |
|  | |  |  | | |
|  | |  |  | | |
|  | |  |  | | |
|  | |  |  | | |
|  | |  |  | | |
|  | |  |  | | |
|  | |  |  | | |
|  | |  |  | | |
|  | |  |  | | |
|  | |  |  | | |
|  | |  |  | | |
|  | |  |  | | |
|  | |  |  | | |
|  | |  |  | | |
|  | |  |  | | |
|  | |  |  | | |
|  | |  |  | | |
|  | |  |  | | |
|  | |  |  | | |
|  | |  |  | | |
|  | |  |  | | |
|  | |  |  | | |
|  | |  |  | | |
|  | |  |  | | |
|  | |  |  | | |
|  | |  |  | | |
|  | |  |  | | |
|  | |  |  | | |
|  | |  |  | | |
|  | |  |  | | |
